# No evidence for maintenance of a sympatric *Heliconius* species barrier by chromosomal inversions

**DOI:** 10.1002/evl3.12

**Published:** 2017-06-14

**Authors:** John W. Davey, Sarah L. Barker, Pasi M. Rastas, Ana Pinharanda, Simon H. Martin, Richard Durbin, W. Owen McMillan, Richard M. Merrill, Chris D. Jiggins

**Affiliations:** ^1^ Department of Zoology University of Cambridge Downing Street Cambridge CB2 3EJ United Kingdom; ^2^ Smithsonian Tropical Research Institute Gamboa Panama; ^3^ Wellcome Trust Sanger Institute Cambridge CB10 1SA United Kingdom

**Keywords:** Chromosomal evolution, evolutionary genomics, insects, speciation

## Abstract

Mechanisms that suppress recombination are known to help maintain species barriers by preventing the breakup of coadapted gene combinations. The sympatric butterfly species *Heliconius melpomene* and *Heliconius cydno* are separated by many strong barriers, but the species still hybridize infrequently in the wild, and around 40% of the genome is influenced by introgression. We tested the hypothesis that genetic barriers between the species are maintained by inversions or other mechanisms that reduce between‐species recombination rate. We constructed fine‐scale recombination maps for Panamanian populations of both species and their hybrids to directly measure recombination rate within and between species, and generated long sequence reads to detect inversions. We find no evidence for a systematic reduction in recombination rates in F1 hybrids, and also no evidence for inversions longer than 50 kb that might be involved in generating or maintaining species barriers. This suggests that mechanisms leading to global or local reduction in recombination do not play a significant role in the maintenance of species barriers between *H. melpomene* and *H. cydno*.

**Impact Summary**

It is now possible to study the process of species formation by sequencing the genomes of multiple closely related species. *Heliconius melpomene* and *H. cydno* are two butterfly species that have diverged over the past two million years. These species have different color patterns, mate preferences, and host plants, traits that involve variants of multiple genes spread across the genome. However, the species still hybridize infrequently in the wild and exchange large parts of their genomes. Typically, when genomes are exchanged, chromosomes are recombined and gene combinations are broken up, preventing species from forming. Theory predicts that gene variants that define species might be linked together because of structural differences in their genomes, such as chromosome inversions, that will not be broken up when the species hybridize. We sequenced large crosses of butterflies to show that there are almost certainly no megabase‐long chromosome regions that are not broken up during hybridization, and while we find evidence for some small chromosome inversions (on the order of tens of kilobases in size), it is unlikely that these are necessary to keep gene combinations together. This suggests that hybridization is rare enough and mate preference is strong enough that inversions are not necessary to maintain the species barrier.

## Introduction

It is now widely appreciated that the evolution and maintenance of new species is constrained by genetic as well as ecological and geographical factors (Seehausen et al. 2014). A classic problem for speciation is that if combinations of divergently selected alleles arise in populations that remain in contact, recombination is expected to break down the associations between alleles and prevent speciation from proceeding (Felsenstein 1981). A large body of work has invoked genetic mechanisms that couple species‐specific alleles and so reduce the homogenizing effects of gene flow (Smadja and Butlin 2011; Nachman and Payseur 2012), including assortative mating, one‐allele mechanisms (Felsenstein 1981), tight physical linkage, pleiotropy, and multiple (or "magic") traits (Servedio et al. 2011). Here, we focus on the role of chromosomal inversions in suppressing recombination of divergently selected alleles in hybrids.

Inversions have frequently been implicated in speciation (White 1978; King 1993; Ayala and Coluzzi 2005; Hoffmann and Rieseberg 2008; Kirkpatrick 2010). Traits associated with reproductive isolation are often linked to inversions (e.g., Noor et al. 2001; Ayala et al. 2013; Fishman et al. 2013) and genetic divergence between species can increase within inverted regions through reduction of gene flow (Navarro and Barton 2003b; Jones et al. 2012; McGaugh and Noor 2012; Lohse et al. 2015). Theory predicts that inversions can spread by reducing recombination between locally adapted alleles (Butlin 2005; Kirkpatrick and Barton 2006; Feder and Nosil 2009; Ortíz‐Barrientos et al. 2016), which can either establish or reinforce species barriers by capturing loci for isolating traits such as mating preferences and epistatic incompatibilities (Dagilis and Kirkpatrick 2016) or allow adaptive cassettes to spread between species via hybridization (Kirkpatrick and Barrett 2015). Reduced recombination within and around inversions has been confirmed in several species (Stevison et al. 2011; Farré et al. 2013), although it is unlikely that gene flow is entirely suppressed within inversions, due to double crossovers and gene conversion (Korunes and Noor 2017), factors addressed in some recent models (Guerrero et al. 2012; Feder et al. 2014).

Several authors have predicted that inversions can enable the formation and maintenance of species barriers in sympatry or parapatry by favoring the accumulation of barrier loci in the presence of gene flow (Noor et al. 2001; Rieseberg 2001; Navarro and Barton 2003a; Faria and Navarro 2010), as opposed to older models where inversions have direct effects on hybrid fertility or viability (discussed in Rieseberg 2001). Especially striking is the fact that most sympatric *Drosophila* species pairs differ by one or more inversions, whereas allopatric pairs are virtually all homosequential (Noor et al. 2001). In the particular case of *Drosophila pseudoobscura* and *D. persimilis*, three chromosomes differ by large, fixed inversions and a fourth chromosome has many varied arrangements (Machado et al. 2007; Noor et al. 2007), genome differentiation is greater within and near inversions (Noor et al. 2007; McGaugh and Noor 2012) and sterility factors are associated with inversions in a sympatric species pair, but with collinear regions in an allopatric pair (Brown et al. 2004). In rodents, sympatric sister species typically have more autosomal karyotypic differences than allopatric sister species (Castiglia 2014). Sympatric sister species of passerine birds are significantly more likely to differ by an inversion than allopatric sister species, with the number of inversion differences best explained by whether the species ranges overlap (Hooper 2016). Although inversions are not the only mechanism by which recombination rate can be modified during speciation, and more recently attention has been drawn to the potential role of genic recombination modifiers (Ortíz‐Barrientos et al. 2016), the very strong effect of inversions on recombination rate, and the fact that they are completely linked to the locus at which recombination is reduced, means that they are perhaps the most likely mechanism of recombination rate evolution during speciation.

We set out to test the role of inversions in the maintenance of species barriers in *Heliconius* butterflies. The 46 species of *Heliconius* have been the focus of a wide range of speciation research (Merrill et al. 2011a; Supple et al. 2013; Kozak et al. 2015; Merrill et al. 2015). Chromosomal inversions are known to play an important role in the maintenance of a complex color pattern polymorphism in *Heliconius numata* (Joron et al. 2011). However, no other *Heliconius* inversions have been identified with traditional methods, as *Heliconius* chromosomes typically appear as dots in chromosome squashes, at least in male tissues (Brown et al. 1992).

Here, we systematically searched for inversions between populations of two *Heliconius* species, *H. melpomene rosina* and *H. cydno chioneus*, which are sympatric in the lowland tropical forests of Panama. These species differ by many traits (Jiggins 2008) including color pattern (Naisbit et al. 2003), mate preference (Jiggins et al. 2001; Naisbit et al. 2001; Merrill et al. 2011b), host plant choice (Merrill et al. 2013), pollen load, and microhabitat (Estrada and Jiggins 2002). Hybrid color pattern phenotypes are attacked more frequently than parental forms, indicating disruptive selection against hybrids (Merrill et al. 2012). Assortative mating between the species is strong, and genetic differences in mate preference are linked to different color pattern loci (Merrill et al. 2011b). Matings between *H. cydno* females and *H. melpomene* males produce sterile female offspring, but male offspring are fertile, and female offspring of backcrosses show a range of sterility phenotypes (Naisbit et al. 2002). Hybrids are extremely rare in the wild, but many natural hybrids have been documented in museum collections (Mallet et al. 2007) and examination of present‐day genomic sequences indicate that gene flow has been pervasive, affecting around 40% of the genome (Martin et al. 2013; Arias et al. 2014). Modeling suggests that the species diverged around 1.5 million years ago, with hybridization rare or absent for one million years, followed by a period of more abundant gene flow in the last half a million years (Kronforst et al. 2013; Martin et al. 2015b), suggesting that the species originated in parapatry, but have been broadly sympatric and hybridizing during their recent history. Although the species are closely related, they are not sister species; several other species such as *Heliconius timareta* and *Heliconius heurippa* are more closely related to *H. cydno* than *H. melpomene*.

Models predict that inversions, or other modifiers of recombination, can be established during both sympatric speciation and secondary contact (Noor et al. 2001; Rieseberg 2001; Feder and Nosil 2009; Feder et al. 2011; Feder et al. 2014). Furthermore, the genetic basis for species differences between *H. melpomene* and *H. cydno* is well understood and would seem to favor the establishment of inversions. Wing pattern differences are controlled by a few loci of major effect (Naisbit et al. 2003), some of which consist of clusters of linked elements. There is also evidence for linkage between genes controlling wing pattern and those underlying assortative mating (Merrill et al. 2011b). The existing evidence for clusters of linked loci of major effect would therefore seem to favor the evolution of mechanisms to reduce recombination between such loci, and hold species differences in tighter association.

We therefore set out to investigate patterns of recombination and test for the presence of inversions between *H. melpomene rosina* and *H. cydno chioneus*. *H. melpomene melpomene* has a high‐quality genome assembly with 99% of the genome placed on chromosomes and 84% ordered and oriented (Heliconius Genome Consortium 2012; Davey et al. 2016). Whole genome resequencing has shown that *F_ST_* between *H. melpomene melpomene* and *H. melpomene rosina* is consistently low across the genome, with only a few small, narrow peaks of divergence, but *F_ST_* between *H. melpomene rosina* and *H. cydno chioneus* is substantially higher and heterogeneous (Martin et al. 2013), and many gene duplications have been identified between the two species (Pinharanda et al. 2017).

**Figure 1:**
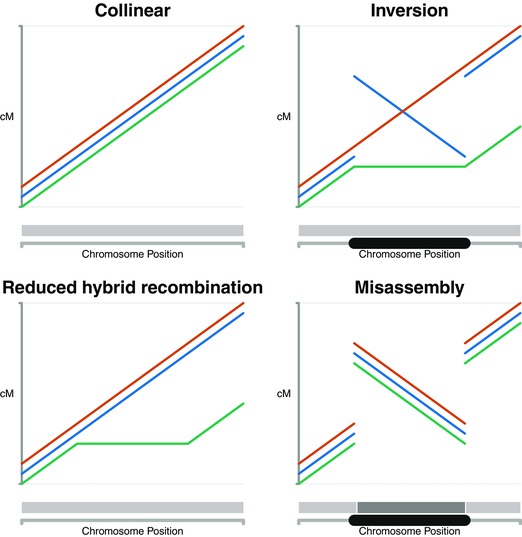
Diagram of expected patterns for collinear, inverted, reduced hybrid recombination, and misassembled genome regions. *Heliconius melpomene*, red; *H. cydno*, blue; *H. cydno* × *H. melpomene* hybrids, green. Gray strip, *H. melpomene* contigs (dark/light gray shows different contigs). Black lozenge, inverted region.

However, *H. melpomene* and *H. cydno* have not yet been examined for evidence of large differences in genome structure such as inversions. To test for this, we constructed fine‐scale linkage maps for *H. melpomene*, *H. cydno*, and *H. cydno* x *H. melpomene* hybrids to test for the presence of reduced recombination in hybrids and inverted regions between the species (Fig. 1). Our linkage maps are based on tens of thousands of new single nucleotide polymorphisms (SNPs) discovered and genotyped using RAD Sequencing data from just under 1000 individuals from 24 crosses. We also generated long‐read sequencing data and new genome assemblies for both species to test for inversions on smaller scales. This is the first systematic survey of genome structure and recombination at a fine scale in a lepidopteran species, and also one of very few such surveys of both parent species and their hybrids (Ortíz‐Barrientos et al. 2016), which we hope will be a valuable test case for the role of inversions in speciation.

## Methods

### LINKAGE MAPS

Full details of methods for our crosses, library preparations, sequencing, and linkage map construction can be found in Supporting Information. In brief, for the within‐species crosses of *H. melpomene rosina* and *H. cydno chioneus*, wild males were mated to virgin stock females and linkage maps were constructed from F1 offspring, whereas for hybrids, *H. cydno* stock females were mated to wild *H. melpomene* males, and F1 males were backcrossed to *H. cydno* stock females, with linkage maps constructed from backcross offspring (Fig. S1). Grandparents, parents, and offspring were RAD sequenced using the PstI restriction enzyme on Illumina HiSeq 2500 and 4000 machines using 100 bp paired end reads, except for one *H. melpomene* and one *H. cydno* trio which were whole genome sequenced with 125 bp paired end Illumina HiSeq 2500 sequencing (previously reported in Malinsky et al. 2016), and 58 hybrid individuals that were sequenced on a HiSeq 2000 using 50 bp single‐end sequencing. RAD sequences were demultiplexed with Stacks (Catchen et al. 2013) and Illumina RAD and whole genome reads were aligned to version 2 of the *H. melpomene* genome (Hmel2; Davey *et al*. 2016) with Stampy (Lunter and Goodson 2011), Picard (http://broadinstitute.github.io/picard/), and GATK (dePristo et al. 2011) and genotype posteriors called with SAMtools mpileup (Li 2011).

Linkage maps were constructed from genotype posteriors using Lep‐MAP. Within‐species linkage maps for *H. melpomene* and *H. cydno* were built with Lep‐MAP2 (Rastas et al. 2016) and some additional bespoke scripts. Due to the more complex cross structure of backcross populations, smaller cross sizes, and lower sequence quality for some crosses, different methods and thresholds were used to construct linkage maps for the *H. cydno* x *H. melpomene* hybrid crosses, now incorporated into Lep‐MAP3 (https://sourceforge.net/projects/lep-map3/). Most notably, separate linkage maps were built for each large within‐species cross, but only one linkage map was constructed for all hybrids, given the small size of the backcross families. The hybrid linkage map was then divided into four separate maps for each pair of grandparents. Full details can be found in Supporting Information.

In brief, SNPs were filtered to ensure each genotype was supported by multiple reads in the majority of individuals, excluding SNPs with rare alleles and segregation distortion. Missing parental genotypes were called based on related parent and offspring genotypes. Markers were identified by clustering together SNPs with almost identical patterns and filtering candidate markers with low support. Markers were separated into linkage groups, setting parameters empirically to identify 21 linkage groups for the expected 21 *H. melpomene/H. cydno* chromosomes, and markers for each linkage group were ordered. As females do not recombine, maternal markers were easy to identify and unchanging, so we could also make use of thousands of SNPs where both parents were heterozygous, by removing the maternal alleles and so converting the SNPs to paternal‐only markers (Jiggins et al. 2005). Initial marker orderings were manually reviewed and edited, and all SNPs were reassigned to the final set of cleaned markers to improve coverage of the genome.

### GENOME SCAFFOLDING

Hmel2 scaffolds were manually ordered according to the linkage maps for each of the three *Heliconius melpomene* crosses wherever possible. A small number of misassemblies in Hmel2 were corrected, with scaffolds being split and reoriented where necessary. Not all scaffolds could be ordered based on the linkage maps alone, so Pacific Biosciences reads were also used. PacBio reads were aligned to Hmel2 scaffolds using BWA mem with ‐x pacbio option (Li 2013). Scaffolds were ordered by manual inspection of spanning reads between scaffolds, identified and summarized by script find_pacbio_scaffold_overlaps.py. Chromosomal positions were assigned by inserting dummy 100 bp gaps between each pair of remaining scaffolds. Although PacBio sequencing could fill gaps between scaffolds, we chose not to do this for these analyses to avoid disrupting Hmel2 linkage map and annotation feature coordinates.

### RECOMBINATION RATE MEASUREMENT AND PERMUTATION TESTING

**Figure 2:**
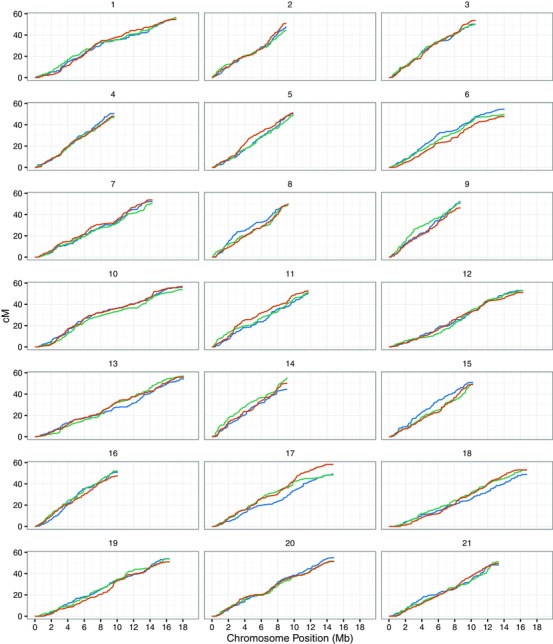
Marey maps of within‐ and between‐species recombination. *Heliconius melpomene*, red; *H. cydno*, blue; *H. cydno* × *H. melpomene* hybrids, green. Chromosomes 1–21 of *H. melpomene* genome assembly version 2 (Hmel2) with improved scaffold ordering shown against cumulative centiMorgan (cM) values.

CentiMorgan values were calculated using the recombination fraction alone, as the maps were sufficiently fine‐scale that mapping functions were not necessary (Ziegler and König 2001); see Supporting Information note on crossover detection for further details. Per‐cross maps (Fig. S3) and map statistics (Table 1) were calculated for F1 parents within‐species and for grandparents for hybrids. Marey maps (Figs. 2 and S3) and total map lengths were calculated using centiMorgan values. Chromosomes were tested for reductions in chromosome‐wide recombination rate in the hybrids compared to *H. melpomene* or *H. cydno* using a bootstrapped Kolmogorov–Smirnov test suitable for discrete data with ties such as recombination counts (ks.boot in the R Matching package; Sekhon 2011), using a one‐tailed test for reduced rates in hybrids with 10,000 bootstrap samples, declaring significance at a 0.05 false discovery rate with control for multiple testing (42 tests, with two comparisons for each of 21 chromosomes).

**Table 1:** Cross information for each species. Summary values for each species shown in bold; mean map lengths and sequencing depths shown in italics

Species	Cross	Offspring	Total map length (cM)	Mean offspring sequencing depth (reads per RAD locus)
*Heliconius melpomene*	**1**	111	1048	22
	**2**	122	1065	23
	**3**	102	1135	31
	**Total/*Mean***	**335**	***1083***	***25***
*Heliconius cydno*	**1**	95	1076	15
	**2**	77	1076	17
	**3**	125	1070	17
	**Total/*Mean***	**297**	***1074***	***16***
*H. melpomene* × *H. cydno* hybrids	**1**	170	1090	19
	**2**	88	1069	30
	**3**	68	1158	21
	**4**	5	1040	28
	**Total/*Mean***	**331**	***1089***	***25***

Fine‐scale recombination rates (Fig. S4) were calculated in windows of 1 Mb with 100 kb steps, counting individual crossovers in each window (see Supporting Information note on crossover detection for further details). One megabase windows were tested for differences in recombination rate by calculating null distributions of rate differences by permutation of species labels across all offspring, testing at a 0.05 false discovery rate over 270,000 permutations, controlling for multiple tests with three comparisons for each of 2549 windows. Ninety‐five percent confidence intervals in Figure S4 were calculated by bootstrap, sampling offspring for each species by replacement 10,000 times and calculating centiMorgan values, plotting 2.5 and 97.5% quantiles for each window.

### INVERSION DISCOVERY

PBHoney (in PBSuite version 15.8.24 (English et al. 2014)) was used to call candidate inversions between *H. melpomene* and *H. cydno*, using alignments of PacBio data to ordered Hmel2 scaffolds made with BWA mem with ‐x pacbio option (Li 2013), retaining only primary alignments, and accepting alignments with minimum mapping quality of 30 in Honey.py tails, running separately on each of four samples (*H. cydno* females, *H. cydno* males, *H. melpomene* females, *H. melpomene* males). Break point candidate sets were compiled together into one file and scaffold positions converted to chromosome positions using script compile_tails.py. PBHoney was run with default options, requiring a minimum of three overlapping reads from three unique zero‐mode waveguides to call a breakpoint candidate. As the *H. cydno* male sample had low coverage, we also ran PBHoney requiring a minimum of two reads from one zero‐mode waveguide and included these tentative candidates where they overlapped with candidates from other samples called with the default settings.

PBHoney was tested for false positives by simulating PacBio reads with pbsim 1.0.3 (Ono et al. 2013), generating a sample profile using the *H. melpomene* female sample and simulating 15 "SMRT cells" at 5x coverage each. Simulated data were then aligned with BWA and inversions called with PBHoney as above.

Trio assemblies were aligned to the ordered Hmel2 genome using NUCmer from the MUMmer suite (Kurtz et al. 2004; version 3.23), followed by show‐coords with show‐Tlcd options, to produce tab‐separated output including scaffold lengths, percentage identities, and directions of hits.

Script detect_inversion_gaps.py was used to integrate the PBHoney inversion candidates with the linkage maps, trio alignments, and *H. melpomene* annotation (from Hmel2). As these data are being used to rule out inversions in regions without recombinations, PBHoney inversion candidates were rejected if at least one recombination for the same species as the candidate was contained within the inversion. PBHoney candidates were also rejected if there was a trio scaffold alignment spanning the candidate inversion, with spanning defined as extending more than half the length of the candidate inversion in either direction. Finally, candidates shorter than 1 kb were rejected, as linkage disequilibrium between SNPs separated by 1 kb or less in *H. melpomene* is significantly higher than background levels (Martin et al. 2016) and so inversions below this size are unlikely to be required to maintain linkage. The retained inversion candidates were then combined into groups by overlap.

**Figure 3:**
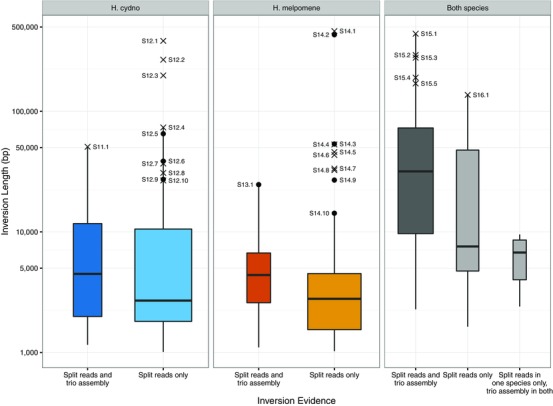
Lengths of candidate inversion groups classified by species and status. See Methods for status definitions. *Heliconius cydno*, blue; *H. melpomene*, red; both species, gray. Dark boxes, evidence from both split reads and trio assemblies; lighter boxes, evidence from either split reads or trio assemblies. Boxes span first and third quartiles; midline shows mean; width represents number of inversions in each category; whiskers extend to the highest value within 1.5 times of the height of the boxes from the edge of the box. Outlier points are shown with crosses if contig gaps fall near inversion breakpoints, circles if not. Labels refer to pages of Figures S11–S17 where full details of each inversion are given.

Each group of overlapping inversion candidates was classified as follows (Fig. 3; Table 4; Figs. S11–S17): *Split reads and trio assembly*, group has at least one PBHoney inversion candidate and at least one trio scaffold with forward and reverse alignments either side of an inversion breakpoint; *Split reads only*, group has at least one PBHoney inversion candidate in at least one sex, but no matching inverted trio scaffolds; *Split reads in one species*, *trio assembly in both*, group has at least one PBHoney inversion candidate in at least one sex of only one species, but trio assembly has inverted scaffolds in at least one sex in both species. These classifications do not cover whether there are multiple contigs across the candidate inversion (see Table 4; Figs. S11–S17) or whether there are trio scaffolds with alignments that span whole PBHoney inversion candidates or single candidate breakpoints (see Figs. S11–S17).

### POPULATION GENETICS STATISTICS

To look for evidence of variation in gene flow, *F_ST_*, *d_XY_*, and *f_d_* were calculated within and around candidate inversions following Martin et al. (2013, 2015a), using all four *H. melpomene rosina*, four *H. cydno chioneus*, four *H. melpomene* French Guiana, and two *H. pardalinus* samples from Martin et al. (2013). Samples were aligned to Hmel2 using bwa mem version 0.7.12 using default parameters and genotypes were called GATK version 3.4 HaplotypeCaller using default parameters except for setting heterozygosity to 0.02. For each candidate inversion, 11 windows equal to the size of the inversion were generated, one for the inversion itself and five either side of the inversion, except where candidates were at the ends of chromosomes. Statistics were calculated for each window with scripts popgenWindows.py and ABBABABAwindows.py in GitHub repository genomics_general (https://github.com/simonhmartin/genomics_general).

### *H. erato* ANALYSIS

The *H. erato* version 1 genome assembly was downloaded from LepBase (http://ensembl.lepbase.org/Heliconius_erato_v1) and aligned to the ordered Hmel2 scaffolds with LAST version 744 (Kiełbasa et al. 2011). Scaffolds and linkage maps were compared with bespoke scripts Hmel2_Herato_maf.py, compile_Herato_maps.py, and Hmel2_Herato_dotplot.R.

## Results

### SUMMARY OF SEQUENCED CROSSES, LINKAGE MAPS DATA, IMPROVED ORDERING OF *H. melpomene* ASSEMBLY

We raised crosses within *H. melpomene* (three F_1_ crosses, 335 offspring), within *H. cydno* (3 F_1_ crosses, 297 offspring) and between *H. cydno* and *H. melpomene* (18 backcrosses of 18 separate F_1_ hybrid fathers to 18 separate *H. cydno* females from four pairs of grandparents, 331 offspring; see Table 1 and Fig. S1 for cross designs and Tables S1–S3 for full sample information) and generated PstI RAD sequencing data for a total of 963 offspring as well as whole genome sequencing for parent–offspring trios from *H. melpomene* cross 2 and *H. cydno* cross 1 (Tables S1–S3). Linkage maps were constructed from tens of thousands of SNPs discovered and genotyped in RAD sequencing and whole genome trio sequencing data (Fig. S2, Tables S4 and S5); separate maps were constructed for each within‐species cross, but, due to the varying size and complexity of the hybrid crosses and heterogeneity of hybrid sequencing data, one single linkage map was constructed for all hybrid crosses using more conservative filters, and the single map was divided into separate F1 crosses *post hoc* (Table 1; Fig. S2; see Methods and Supporting Information for full details). We also generated Pacific Biosciences long‐read data for pools of male and female larvae from *H. melpomene* cross 2 and *H. cydno* cross 1 (Table 2).

**Table 2:** Summary of Pacific Biosciences sequencing and trio assemblies used to identify inversions

		*Heliconius melpomene*		*Heliconius cydno*	
		Females	Males	Females	Males
Pacific Biosciences sequences	SMRT cells	15	10	15	10
	Reads	3,138,554	2,079,617	3,022,815	1,218,186
	Reads mapped	3,006,793	1,985,294	2,745,477	1,089,370
	Reads mapped percentage	95.8	95.5	90.8	89.4
	Bases	16,172,976,632	11,157,098,567	15,277,034,979	4,457,967,153
	Bases mapped	15,842,062,694	10,864,609,062	14,285,479,974	4,170,115,456
	Bases mapped percentage	98	97.4	93.5	93.5
	Depth mode for mapped bases	43	27	38	10
	Bases mapped for PBHoney (primary alignments + tails)	10,848,364,007	7,275,050,641	9,173,633,875	2,725,704,499
	Based mapped for PBHoney %	67	65.2	60	61.1
	Mode of base depth for PBHoney bases	37	24	33	8
Trio assemblies	Scaffolds	49,035	46,134	32,548	34,566
	Total length	267.8	276.8	257.9	270.3
	Mean scaffold length (kb)	5.4	6.0	7.9	7.8
	Scaffold N50 (kb)	16.9	20.1	27.0	25.7
	Max scaffold length (kb)	140	165	234	267

To improve the accuracy of our recombination rate measurements, we first used the new linkage maps and Pacific Biosciences long‐read data for *Heliconius melpomene* to improve the scaffolding of version 2 of the *H. melpomene* genome assembly (Hmel2; 795 scaffolds, 275.2 Mb total length, 641 scaffolds placed on chromosomes (274.0 Mb), 2.1 Mb scaffold N50 length; Davey et al. 2016). This resulted in an updated genome assembly with 13 complete chromosomes, the remaining eight chromosomes having one long central scaffold with short unconnected scaffolds at either end (272.6 Mb placed on 21 chromosomes in 38 scaffolds, including 17 minor scaffolds at chromosome ends totaling 1.3 Mb; 294 additional scaffolds [2.6 Mb] were not placed on chromosomes and unused in further analyses). This updated reference genome assembly (referred to as ordered Hmel2) was used for all further analyses.

We transferred our existing linkage maps to the new *H. melpomene* chromosomal assembly. Density of SNPs in the final map varies by species and chromosome position (Tables S4 and S5; Figs. S2 and S3; mean paternal SNP density for *H. melpomene*, 6101.1 bp; *H. cydno*, 9043.8 bp; hybrids, 13,642.4 bp). The variation is largely due to variation in sequencing depth and PstI site occurrence, which are both related to GC content (Fig. S3; Benjamini and Speed 2012; see Supporting Information note for full discussion). However, SNP density is not correlated with recombination rate, final map lengths, or crossover resolution, and final map lengths are consistent across all crosses (see below), so we do not believe variation in SNP density has affected our results (see Supporting Information note for further details).

Crossing over has previously been shown to be absent in *Heliconius* females (Turner and Sheppard 1975; Jiggins et al. 2005; Pringle et al. 2007; Davey et al. 2016), and we could find no evidence to the contrary in any of our crosses (Fig. S2), so we focus on paternal crossovers throughout (see Supporting Information note for a discussion and defense of this point). The paternal linkage maps have a mean genetic length of 51 cM and mean recombination rate of 4.2 cM/Mb per chromosome for both species and hybrids (Table 3). Mean crossovers per offspring across 21 chromosomes were 10.8 in *H. melpomene* (SD 2.4, from 335 offspring) and 10.7 in *H. cydno* (SD 2.2, from 297 offspring). This is consistent with an expectation of one crossover per chromosome per offspring and a 50% chance of inheritance of one of the 2 recombined gametes (from 4 total gametes).

**Table 3:** Physical and genetic map information for each chromosome and species

			*Heliconius melpomene*		*Heliconius cydno*		*H. cydno x H. melpomene*	
Chromosome	Physical length (bp)	Predicted recombination rate (cM/Mb)	Genetic length (cM)	Rate (cM/Mb)	Genetic length (cM)	Rate (cM/Mb)	Genetic length (cM)	Rate (cM/Mb)
**1**	17,206,585	5.8	54.6	3.17	54.5	3.17	56.2	3.27
**2**	9,045,316	11.1	50.7	5.61	47.5	5.25	44.4	4.91
**3**	10,541,528	9.5	53.7	5.10	50.2	4.76	49.9	4.73
**4**	9,662,098	10.3	48.1	4.97	50.5	5.23	46.9	4.85
**5**	9,908,586	10.1	51.0	5.15	50.2	5.06	48.7	4.91
**6**	14,054,175	7.1	47.8	3.40	54.5	3.88	49.9	3.55
**7**	14,308,859	7.0	53.7	3.76	52.2	3.65	50.2	3.51
**8**	9,320,449	10.7	49.3	5.28	49.8	5.35	49.6	5.32
**9**	8,708,747	11.5	46.3	5.31	50.8	5.84	52.3	6.00
**10**	17,965,481	5.6	56.7	3.16	55.9	3.11	53.8	3.00
**11**	11,759,272	8.5	52.5	4.47	49.8	4.24	51.4	4.37
**12**	16,327,298	6.1	51.0	3.13	52.9	3.24	52.9	3.24
**13**	18,127,314	5.5	55.8	3.08	54.2	2.99	56.8	3.13
**14**	9,174,305	10.9	50.2	5.47	44.4	4.84	55.3	6.03
**15**	10,235,750	9.8	49.0	4.78	50.8	4.97	49.3	4.81
**16**	10,083,215	9.9	47.5	4.71	50.8	5.04	52.0	5.16
**17**	14,773,299	6.8	58.2	3.94	49.2	3.33	48.3	3.27
**18**	16,803,890	6.0	53.1	3.16	48.8	2.91	52.9	3.15
**19**	16,399,344	6.1	51.0	3.11	53.9	3.29	54.1	3.30
**20**	14,871,695	6.7	51.3	3.45	54.9	3.69	51.7	3.48
**21**	13,359,691	7.5	49.6	3.71	48.1	3.60	51.1	3.82
Genome	**272,636,897**	**7.7**	**1081.2**	**3.97**	**1074.1**	**3.94**	**1077.5**	**3.95**
Chromosome		**8.2**	**51.5**	**4.2**	**51.1**	**4.2**	**51.3**	**4.2**

### DIFFERENCES IN RECOMBINATION RATE BETWEEN SPECIES AND HYBRIDS

To identify potential genomic regions that may influence the maintenance of the species barrier, we examined our linkage maps for evidence of reduced recombination in the hybrids compared to the within‐species crosses at the genome‐wide scale, the chromosome scale, and at fine scale (1 Mb windows). Figure 2 shows Marey maps (Chakravarti 1991) for each of the 21 *Heliconius melpomene* chromosomes for *H. melpomene*, *H. cydno*, and *H. cydno* x *H. melpomene* hybrids, with crossovers from all crosses per species combined (see Fig. S4 and Table 1 for per‐cross Marey maps and map lengths). Mean broad scale recombination rates and total genome‐wide map lengths were almost identical across *H. melpomene*, *H. cydno*, and the hybrids (Tables 1 and 3; mean genome‐wide recombination rates were all 3.9 cM; mean chromosome‐wide recombination rates were all 4.2 cM; total map lengths were *H. melpomene*, 1081 cM; *H. cydno*, 1074.1 cM; hybrids, 1077 cM).

Some differences in chromosome‐scale recombination rate between the species maps are visible; for example, on chromosome 17, the *H. melpomene* map is 9.1 cM longer than *H. cydno*; on chromosome 6, *H. cydno* is 6.8 cM longer than *H. melpomene* (Fig. 2; Table 3). However, we are primarily interested in recombination suppression in hybrids, and only chromosome 2 has a significantly reduced chromosome‐wide recombination rate in the hybrid crosses, and only when compared to *H. melpomene*, not *H. cydno* (one‐tailed Kolmogorov–Smirnov tests; see Methods).

At the fine scale, measuring recombination rate in sliding 1 Mb windows across chromosomes, regions with reduced recombination in the hybrids can be observed (Fig. S5; for example, chromosome 17, 11–13 Mb and chromosome 19, 13.5–14 Mb), but none of these regions are statistically significant (permutation test for fine‐scale variation in recombination in 1 Mb sliding windows at a 5% false discovery rate; see Methods).

### RECOMBINATION MAPS SHOW NO MAJOR INVERSIONS BETWEEN SPECIES

We also examined our recombination maps for evidence of inversions between species (Fig. 1). There are no regions of any map with a detectable reversed region in *H. cydno* or the hybrids with respect to *H. melpomene* (Fig. 2). This is true for the species maps and for all individual cross maps (Fig. S4). This indicates there are no large fixed inversions between *H. melpomene* and *H. cydno*.

Known or predicted chromosome inversions involved in the maintenance of species barriers are typically megabases long, and models indicate that inversions may have to be very large to become fixed in a population (Feder et al. 2014). Our maps are sufficiently fine scale to rule out the presence of inversions on the megabase scale (*H. melpomene* mean gap between markers, 115 kb, median 87 kb, maximum 1.38 Mb; *H. cydno* mean 135 kb, median 101 kb, maximum 1.14 Mb; see Figs. S6 and S7, and Supporting Information notes on our ability to detect and resolve crossovers). Simulation of random inversions indicates that our existing maps give us power to detect ∼98% of 500 kb inversions, ∼90% of 250 kb inversions and ∼75% of 100 kb inversions (Fig. S8). These sizes are smaller than most inversions known to be associated with adaptive traits or species barriers, which are typically on the megabase scale; however, they are on the order of the sizes of the known inversions involved in within‐species polymorphism in *H. numata* (see Introduction). The recombination maps alone do not rule out the presence of an inversion in any remaining gap between markers within *H. melpomene* or *H. cydno*.

### DETECTION OF SMALL INVERSIONS WITH LONG SEQUENCE READS AND TRIO ASSEMBLIES

To test for the presence of smaller fixed inversions between *H. melpomene* and *H. cydno* that were undetectable using our recombination maps, we generated Pacific Biosciences long‐read sequence data for pools of male and female larvae from one each of the *H. melpomene* and *H. cydno* crosses used to generate recombination maps (Table 2; Figs. S9 and S10). We called candidate inversions from the long‐read data using PBHoney to identify reads with clipped alignments, realign the clipped read ends, and detect such split reads with inverted alignments.

We also generated Illumina short‐read assemblies of the maternal and paternal genomes of one offspring from the same crosses used to generate the linkage maps and PBHoney candidates. These assemblies were constructed using a trio assembly method that separates maternal and paternal reads from one offspring and constructs haplotypic assemblies of each parental genome, providing longer and more accurate contigs compared to standard Illumina assemblies of heterogenous genomes such as those of *Heliconius* species (Malinsky et al. 2016; Table 2). We aligned these trio assemblies to the *H. melpomene* genome and assessed whether the resulting alignments supported or conflicted with candidate split read inversions.

In total, 1494 raw PBHoney split read candidates were identified across the four samples (two sexes for each of two species; Tables 2 and S6). As we consider our linkage maps to be reliable, and we are concerned with regions of the genome where our linkage maps do not contain recombinations, we rejected 438 split read candidates (30%) that spanned recombinations in the linkage maps (Table S6), of which 294 (20%) were longer than 1 Mb, with 36 (2.5%) longer than 10 Mb. The remaining candidates were all in regions that may contain crossovers but where crossover location could not be resolved, or in regions where multiple SNPs showed that there were no crossovers and so recombination could not be used to detect inversions (see Supporting Information note for discussion).

A further 344 candidates (23%) were removed because the candidate was spanned by a trio scaffold from the same species by 50% of the inversion length on either side (Table S6). These rejected candidates are likely to be mostly false positives; when we simulated PacBio reads directly from the ordered Hmel2 reference genome, PBHoney called 49 "false‐positive" inversions. Alternatively, they may be generated by polymorphic inversions that are not present in the two parental haplotypes in the trio assemblies, but are present in at least one of the other two parental haplotypes and so detectable in the PacBio data, but as we expect only fixed inversions to contribute to species barriers, we have not considered these candidates any further.

A further 199 of the 1494 candidates (13%) were removed because they were shorter than 1 kb (Table S6) on the grounds that there is already above‐background linkage disequilibrium between SNPs separated by 1 kb or less in *H. melpomene* (Martin et al. 2016). The remaining 463 split read candidate inversions from the four samples were merged into 185 candidate groups based on their overlaps. We expect fixed inversions to be present in both sexes for each species, but the four samples were sequenced with variable coverage, with particularly low coverage for the *H. cydno* males (Table 2). Given this, 173 additional candidates with less robust support that overlapped with the 185 merged groups were included in the dataset (Table S6). Each of the merged groups was then classified based on their presence in either or both species and their support by split read and trio assembly evidence (Table 4; Figs. 3 and S11–S17; Table S7; see Methods for full criteria).

**Table 4:** Classification of candidate inversions

Species	Evidence	Candidate inversions	Breakpoint near contig boundaries (%)	Supporting Information figure
*Heliconius cydno*	Split reads and trio assembly	13	3 (23%)	S11
	Split reads only	52	17 (33%)	S12
	Total	**65**	**20 (31%)**	
*Heliconius melpomene*	Split reads and trio assembly	9	4 (44%)	S13
	Split reads only	46	15 (33%)	S14
	Total	**55**	**19 (35%)**	
Both species	Split reads and trio assembly	42	39 (92%)	S15
	Split reads only	17	11 (64%)	S16
	Split reads in one species, trio assembly in both	6	3 (50%)	S17
	Total	**65**	**53 (82%)**	
Grand total		**185**	**92 (50%)**	

Despite the high rate of likely false positives, PBHoney does appear to detect some genuine inverted sequences relative to the reference genome. Where candidate inversions are identified in the same location from both *H. melpomene* and *H. cydno* sequence data, it is likely that these candidates are accurately reflecting a misassembly in the reference genome. This is especially the case where the inversion breakpoints fall at contig boundaries in the assembly, as local misassembly can prevent neighboring contigs from being assembled. There were 59 candidate groups where PBHoney found overlapping inversions in both *H. cydno* and *H. melpomene*, 50 (85%) of which span multiple contigs, with most inversion breakpoints falling at or near to the end of a contig (Table 4; Figs. 3 and S15–S17). This indicates either that some whole contigs are inverted, or that the ends of contigs have inverted regions that need to be reassembled (which perhaps explains the failure to fill the contig gaps during assembly). In contrast, candidate inversions specific to one or other species were less likely to span multiple contigs (20 of 65 *H. cydno* candidates (31%), and 19 of 56 *H. melpomene* candidates (35%); Figs. S11–S14). We suggest that while some of these species‐specific inversions could be explained by misassemblies and incomplete PacBio coverage across both species, many of them could be genuine inversions.

### CANDIDATE INVERSIONS ASSESSED USING TRIO ASSEMBLIES AND POPULATION GENETICS

As the false positive rate for PBHoney is high, we made further use of the trio assemblies to find support for the remaining PBHoney candidate inversion groups (Tables 4 and S7, Figs. S11–S17). Thirteen *H. cydno* and nine *H. melpomene* groups were further supported by trio scaffolds aligning with inverted hits within inversion breakpoints (Fig. 3, “Split reads and trio assembly”; Figs. S11 and S13). Of these, eight *H. cydno* and three *H. melpomene* candidates did not have inversion breakpoints near contig boundaries, suggesting that they are less likely to be due to genome misassemblies. If these inversions are species‐specific, as indicated by the PBHoney output, we expect support for the reference genome order in the species that does not possess the inversion candidate. Indeed, six of these *H. cydno* and all three *H. melpomene* candidates have trio scaffolds of the other species spanning the whole inversion or one of the breakpoints, supporting the inversion as being species‐specific (Figs. S11.2, S11.4, S11.6, S11.8, S11.10, S11.11; Fig S13.6, S13.8, S13.9). Hence, we have likely detected a small number of species‐specific inversions. However, the longest of these candidates is Figure S11.2 at 20,247 bp, far shorter than any known inversion relevant for speciation and shorter than is expected to become fixed in simulations (Feder et al. 2014). Furthermore, this is only slightly larger than the distance at which linkage disequilibrium in *H. melpomene* reaches background levels (∼10 kb; Martin et al. 2016), such that any effect of reduced recombination would be slight in population genetic terms. We conclude that there are a small number of likely species‐specific inversions, but that these are too small to play a role in speciation via reduced recombination. Notably, none of these candidate inversions were located near loci known or suspected to determine species differences in wing pattern or any other trait with known locations (Nadeau et al. 2014, Davey et al. 2016; we have transferred the *H. melpomene* loci to positions in the ordered Hmel2 genome in Table S8 and also included a table of all candidate inversion positions for comparison in Table S7; the BD region has been narrowed based on the results of Wallbank et al. 2016).

We also calculated *F_ST_*, *d_XY_*, and *f_d_* (Cruickshank and Hahn 2014; Martin et al. 2015a) across inverted regions (Figs. S11–S17) to look for evidence of variation in gene flow at the inversion relative to surrounding regions. An inversion acting as a species barrier typically produces a signal of elevated *F_ST_* and reduced admixture (here estimated using *f_d_*; Aulard et al. 2002; Deng et al. 2008; Huynh et al. 2011; Nachman and Payseur 2012; Fontaine et al. 2015; Love et al. 2016), and an inversion enabling the spread of an adaptive cassette between species (Kirkpatrick and Barrett 2015) might produce a signal of elevated *f_d_*. However, we see very little evidence for deviations in these statistics within the handful of candidate inversions compared to the surrounding regions, with only one *H. cydno* inversion (Fig. S11.4, 11,719 bp long) showing a noticeable localized increase in *F_ST_* and small increase in *d_XY_*. This region contains no annotated features, although of course this does not rule out some functional importance of this region.

Some candidates with only split read evidence, many in only one sex, are hundreds of kilobases long (outliers labeled in Fig. 3, particularly those marked with circles, where breakpoints are not near contig boundaries), which, if real, may be relevant to speciation. However, given the large number of false positives produced by PBHoney, the lack of supporting evidence from trio assemblies, and the lack of clear, localized deviations in *F_ST_*, *d_XY_*, and *f_d_* signals at these candidates, it is unlikely these candidates, even if they are real, are substantial species barriers.

### THE *H. melpomene* AND *H. erato* GENOMES ARE MOSTLY COLLINEAR, BUT DO CONTAIN INVERTED REGIONS

We used the recently completed *H. erato* genome assembly (Van Belleghem et al. 2017) to investigate the incidence of inversions between more divergent genomes in the *Heliconius* genus. *Heliconius melpomene* and *H. erato* diverged 10 million years ago (Kozak et al. 2015; Fig. S18), considerably more than the ∼1.5 million years between *H. melpomene* and *H. cydno*. Despite the substantial divergence time, the chromosomes of the two species are collinear throughout at the large scale, with a few exceptions. There are many regions of the *H. erato* genome assembly that are inverted relative to the ordered Hmel2 assembly, but they fall within regions where the *H. erato* or *H. melpomene* linkage maps were not informative and so may be due to genome misassemblies. For example, *H. erato* scaffolds Herato0201, Herato0202, and Herato0203 on chromosome 2, and the first 300 kb of *H. melpomene* chromosome 3, may be misoriented rather than genuinely inverted.

However, three large inverted and/or translocated regions are well supported by linkage map markers in both species, and so are likely to be genuine inversions (Fig. S18; chromosome 2, *H. erato* 7–10 Mb, *H. melpomene* 4–7 Mb; chromosome 6, *H. erato* 16–18 Mb, *H. melpomene* 12–13 Mb; chromosome 20, *H. erato* 13–15 Mb, *H. melpomene* 11–12 Mb). The chromosome 2 rearrangement is particularly striking, spanning four *H. erato* scaffolds (Herato0211, Herato0212, Herato0213, and Herato0214) and multiple linkage map markers in both species. On current scaffold ordering, this rearrangement appears to be an inversion followed by a translocation (for scaffold Herato0214), but it is likely to be a single inversion; as scaffolds Herato0212, Herato0213, and Herato0214 are all found at the same marker on the linkage map, it may be that these scaffolds need to be reoriented and reordered, inserting scaffold Herato0212 at the start of the inversion in Herato0211 and inverting Herato0214. Nevertheless, this large region deserves further attention, especially as some pairs of *H. erato* subspecies appear to have elevated *F_st_* in the center of chromosome 2 (see Fig. 2 of Van Belleghem et al. 2017). It is unclear whether this inversion is polymorphic only in *H. erato* or whether it is present in other *Heliconius* species.

## Discussion

We have systematically tested the hypothesis that inversions causing reduced recombination rates in hybrids might maintain species barriers with gene flow (Ortíz‐Barrientos et al. 2016). High‐density linkage maps and high‐coverage long‐read sequence data give us considerable power to both measure recombination rate and detect structural rearrangements. We find evidence for some small inversions, but not for inversion differences between *H. melpomene* and *H. cydno* at a scale that is likely to influence the speciation process.

Our data have some limitations that might have prevented us from identifying genuine inversions between *H. melpomene* and *H. cydno*. First, we have only sequenced crosses from three or four pairs of parents per species, and so may have missed polymorphic inversions absent from our sampling of wild individuals. However, any inversion important for speciation is expected to be fixed between the species, so it should have been detected even in small samples. Second, our ability to detect inversions and differences in recombination is limited by the size of our crosses (roughly 300 individuals for each species and for the hybrids), and the maps contain regions of the genome up to a maximum of 1.3 Mb without crossovers that might conceivably harbor inversions (see Results, recombination maps show no major inversions between species); further crosses could improve resolution in these areas. Third, we have used RAD sequencing data to measure recombinations, which is limited to ∼10 kb resolution (using the PstI restriction enzyme); some of the smaller candidate inversions could be confirmed by developing further markers within them at narrower resolution, but this would not change our conclusion that inversions are unlikely to be involved in speciation between *H. melpomene* and *H. cydno*.

One important aspect of our experimental design is that we have measured recombination in hybrids as well as investigating gene order in the parental species. This gives power to detect reduced hybrid recombination rate more generally as well as specifically the presence of inversions. We have found no evidence for significantly reduced recombination in hybrids, at the broad (chromosome) scale or megabase scale, suggesting that genic modifiers of recombination are unlikely to have widespread effects in these species. Larger crosses would give greater resolution to this test, and might detect smaller regions of reduced recombination. Nevertheless, we can decisively rule out the presence of any multimegabase rearrangements among these samples.

We complemented the linkage maps with PacBio sequencing and trio assemblies to detect candidate inverted regions at a smaller scale. This approach also has challenges and generated a high rate of false positives. One potential source of difficulties is reliance on alignment to the *H. melpomene* reference genome assembly. The existing assembly has 25% transposable element content (Lavoie et al. 2013) and is likely missing around 6% of true genome sequence, mostly due to collapsed repeats (Davey et al. 2016). Inversion breakpoints are typically repeat‐rich, which increases the likelihood that reads or scaffolds will not align correctly, and that the breakpoint regions could be misassembled or absent in the reference genome and in the trio assemblies. This problem may be worse for *H. cydno*, where more divergent sequence may align incorrectly or not align at all, and unique *H. cydno* sequence will not be present in the reference (an additional ∼5% of *H. cydno* sequence did not map to the *H. melpomene* genome compared to *H. melpomene* samples; Table 2). These issues may explain the high observed rate of false positives in our data.

Nonetheless, the detection of likely genome misassemblies indicates that our methods do indeed have the power to detect real rearrangements. These are supported by multiple lines of evidence in both species and fall near contig boundaries. These misassemblies could be due to whole inverted contigs, or to misassembled inverted regions at the ends of contigs, which may be preventing the contigs being joined by spanning reads. Misassemblies demonstrate that our methods are capable of detecting large rearrangements in the sampled reads relative to the genome assembly.

In contrast, our candidate species‐specific inversions are typically smaller than the misassemblies, and are mostly not supported by multiple lines of evidence. Indeed, we can find no compelling fixed candidate inversions supported by both the split read and trio assembly datasets that also show evidence of an increase in *F_ST_* or *d_XY_*, except for the 11.7 kb inversion shown in Figure S11.4, which is probably too small to substantially increase linkage across this locus beyond that expected by normal decay of linkage disequilibrium (Martin et al. 2016). It is possible that some of the candidates with less robust evidence are genuine, given the limitations described above, but on the existing evidence we cannot identify any inversions that are likely to be involved in maintaining species barriers between *H. melpomene* and *H. cydno*.

Although existing models identify situations where chromosome inversions can spread to fixation between two species and maintain a species barrier, they do not show that inversions always spread during speciation with gene flow. For example, in the model of Feder et al. (2014), inversions only fix when the strength of selection on the loci captured by the inversion is considerably lower than migration between the species. Similarly, Dagilis and Kirkpatrick (2016), modeling the spread of inversions that capture a mate preference locus and one or more epistatic hybrid viability genes, show that inversions are unlikely to spread where pre‐ and post‐zygotic reproductive isolation is already strong. In a recent review, Ortíz‐Barrientos et al. (2016) also highlight that during reinforcement, assortative mating and recombination modifiers such as inversions are antagonistic; if strong assortative mating arises first, there is only weak selection for reduced recombination.

We considered *H. melpomene* and *H. cydno* to be good candidates for the spread of inversions because there are linked loci causing reproductive isolation, because hybridization has been ongoing for much of their history, because an inversion is known to maintain color pattern polymorphism in *H. numata* (Joron et al. 2011), and because they are a parallel case to that of *D. pseudoobscura* and *D. persimilis*, where inversions do appear to maintain the species barrier (Noor et al. 2007). Comparisons between sympatric and allopatric populations of the two *Heliconius* species have shown that almost a third of the genome is admixed in sympatry and that hybridization has been ongoing for a long time (Martin et al. 2013), perhaps at a low rate.

However, strong selection on species differences and assortative mating are not conducive to the spread of inversions. Aposematic warning patterns are strongly selected (Mallet and Barton 1989) with F_1_ hybrids twice as likely to be attacked as parental phenotypes (Merrill et al. 2012), and prezygotic isolation in the form of mate preference is almost complete (Jiggins et al. 2001). Therefore, inversions may not be necessary for divergent loci to accumulate between the species. Thus, in this case, the evolution of strong assortative mating may have been favored by reinforcement selection and close physical linkage between preference and wing‐patterning loci (Merrill et al. 2011b), and it is likely that the species barrier between *H. melpomene* and *H. cydno* has persisted with gene flow, but without the suppression of recombination by chromosome inversions.

An alternative and complementary explanation is that the rate of production of inversions may simply be low in *Heliconius*. This is suggested by the low background rate of fixation of inversions in *Heliconius* genomes. We have shown that, not only is there little evidence for substantial, fixed inversions between *H. melpomene* and *H. cydno*, but also that *H. melpomene* and *H. erato*, which last shared a common ancestor over 10 million years ago, have largely collinear genomes, and it is also known that there is substantial chromosomal synteny across the Nymphalids (Ahola et al. 2014). The association of multiple inversions with the wing pattern polymorphism in *H. numata* is all the more remarkable given the low background rate of inversions in these butterflies. This contrasts with, for example, the many fixed or polymorphic inversions in the genomes of *Drosophila* (Krimbas and Powell 1992), *Anopheles* (Ayala et al. 2014), and primates (Samonte and Eichler 2002), and especially with the solid case for the influence of inversions on speciation between sympatric populations of *D. pseudoobscura* and *D. persimilis* (Noor et al. 2001; Machado et al. 2007; Noor et al. 2007), where major fixed inversions occur on most chromosomes. Although *H. melpomene* and *H. cydno* have similarly divergent genomes overall compared to the *Drosophila* species pair, we do not find evidence for a similar role for inversions in maintaining the species barrier. Although inversions are clearly involved in speciation in many taxa studied to date, they appear to be absent in *H. melpomene* and *H. cydno* and in the flycatcher species pair *Ficedula albicollis* and *F. hypoleuca* (Ellegren et al. 2012), so the possibility of speciation without inversions should be kept in mind. We conclude that species barriers can persist during speciation with gene flow without substantial suppression of between‐species recombination.

Associate Editor: Z. Gompert

## Supporting information

Supporting information

**Figure S1**. Cross design.

**Figure S2**. Genetic and physical maps for each ordered Hmel2 chromosome.

**Figure S3**. SNP density, PstI site density, and GC content for each ordered Hmel2 chromosome.

**Figure S4**. Marey maps of recombinations for each cross separately.

**Figure S5**. Recombination rates in cM/Mb for each ordered Hmel2 chromosome.

**Figure S6**. Gaps in linkage maps where lack of precise recombination data cannot rule out presence of inversions.

**Figure S7**. Histograms of gap lengths for *Heliconius cydno* and *H. melpomene*.

**Figure S8**. Probability of detecting random inversions of sizes from 10 kb to 1.5 Mb given existing linkage maps for *Heliconius melpomene* and *H. cydno*.

**Figure S9**. Histograms of raw read lengths for Pacific Biosciences sequencing.

**Figure S10**. Histograms of base depths across the genome after alignment of raw PacBio reads to *Heliconius melpomene* genome assembly Hmel2.

**Figures S11–S17**. Full evidence for each candidate inversion group, separated into the classes shown in Figure 3 and Table 4.
**S11,** *H. cydno*, split reads and trio assembly.

**S12,** *H. cydno*, split reads only.

**S13,** *H. melpomene*, split reads and trio assembly.

**S14,** *H. melpomene*, split reads only.

**S15,** Both species, split reads and trio assembly.

**S16,** Both species, split reads only.

**S17,** Both species, split reads in one species, trio assembly in both.

**Figure S18**. Oxford grids for ordered Hmel2 scaffolds and *H. erato* scaffolds.

**Table S1**. Full sample details for *Heliconius melpomene* crosses.
**Table S2**. Full sample details for *Heliconius cydno* crosses.
**Table S3**. Full sample details for *Heliconius cydno* x *H. melpomene* hybrid crosses.
**Table S4**. SNP and marker information for within‐species crosses.
**Table S5**. SNP and marker information for hybrid crosses.
**Table S6**. Counts of raw and filtered PBHoney candidate inversions.
**Table S7**. Locations of candidate inversions.
**Table S8**. Locations of known trait loci.

## References

[evl312-bib-0001] Ahola, V. , R. Lehtonen , P. Somervuo , L. Salmela , P. Koskinen , P. Rastas , et al. 2014 The Glanville fritillary genome retains an ancient karyotype and reveals selective chromosomal fusions in Lepidoptera. Nat. Commun. 5:4737 Available at 10.1038/ncomms5737. 25189940 PMC4164777

[evl312-bib-0002] Arias, C. F. , C. Salazar , C. Rosales , M. R. Kronforst , M. Linares , E. Bermingham , and W. O. McMillan . 2014 Phylogeography of *Heliconius cydno* and its closest relatives: disentangling their origin and diversification. Mol. Ecol. 23:4137–4152. 24962067 10.1111/mec.12844

[evl312-bib-0003] Aulard, S. , J. R. David , and F. Lemeunier . 2002 Chromosomal inversion polymorphism in Afrotropical populations of *Drosophila melanogaster* . Genet. Res. Camb. 79:49–63. 10.1017/s0016672301005407 11974603

[evl312-bib-0004] Ayala, D. , R. F. Guerrero , and M. Kirkpatrick . 2013 Reproductive isolation and local adaptation quantified for a chromosome inversion in a malaria mosquito. Evolution 67:946–958. 23550747 10.1111/j.1558-5646.2012.01836.x

[evl312-bib-0005] Ayala, D. , A. Ullastres , and J. González . 2014 Adaptation through chromosomal inversions in *Anopheles* . Front. Genet. 5:129. 24904633 10.3389/fgene.2014.00129 PMC4033225

[evl312-bib-0006] Ayala, F. J. , and M. Coluzzi . 2005 Chromosome speciation: humans, *Drosophila*, and mosquitoes. Proc. Natl. Acad. Sci. USA. 102:6535–6542. 15851677 10.1073/pnas.0501847102 PMC1131864

[evl312-bib-0007] Benjamini, Y. , and T. P. Speed . 2012 Summarizing and correcting the GC content bias in high‐throughput sequencing. Nucleic Acids Res. 40:e72. 22323520 10.1093/nar/gks001 PMC3378858

[evl312-bib-0008] Brown, K. M. , L. M. Burk , L. M. Henagan , and M. A. F. Noor . 2004 A test of the chromosomal rearrangement model of speciation in *Drosophila pseudoobscura* . Evolution 58:1856–1860. 15446438 10.1111/j.0014-3820.2004.tb00469.x

[evl312-bib-0009] Brown, K. S. , T. C. Emmel , P. J. Eliazar , and E. Suomalainen . 1992 Evolutionary patterns in chromosome numbers in neotropical Lepidoptera. Hereditas 117:109–125. 1459855 10.1111/j.1601-5223.1992.tb00165.x

[evl312-bib-0010] Butlin, R. K. 2005 Recombination and speciation. Mol. Ecol. 14:2621–2635. 16029465 10.1111/j.1365-294X.2005.02617.x

[evl312-bib-0011] Castiglia, R. 2014 Sympatric sister species in rodents are more chromosomally differentiated than allopatric ones: implications for the role of chromosomal rearrangements in speciation. Mammal Rev. 44:1–4.

[evl312-bib-0012] Catchen, J. , P. A. Hohenlohe , S. Bassham , A. Amores , and W. A. Cresko . 2013 Stacks: an analysis tool set for population genomics. Mol. Ecol. 22:3124–3140. 23701397 10.1111/mec.12354 PMC3936987

[evl312-bib-0013] Chakravarti, A. 1991 A graphical representation of genetic and physical maps: the Marey map. Genomics 11:219–222. 1765381 10.1016/0888-7543(91)90123-v

[evl312-bib-0014] Cruickshank, T. E. , and M. W. Hahn . 2014 Reanalysis suggests that genomic islands of speciation are due to reduced diversity, not reduced gene flow. Mol. Ecol. 23:3133–3157. 24845075 10.1111/mec.12796

[evl312-bib-0015] Dagilis, A. J. , and M. Kirkpatrick . 2016 Prezygotic isolation, mating preferences, and the evolution of chromosomal inversions. Evolution 70:1465–1472. 27174252 10.1111/evo.12954

[evl312-bib-0016] Davey, J. W. , M. Chouteau , S. L. Barker , L. Maroja , S. W. Baxter , F. Simpson , et al. 2016 Major improvements to the *Heliconius melpomene* genome assembly used to confirm 10 chromosome fusion events in 6 million years of butterfly evolution. G3 6:695–708. 26772750 10.1534/g3.115.023655 PMC4777131

[evl312-bib-0017] Deng, L. , Y. Zhang , J. Kang , T. Liu , H. Zhao , Y. Gao , et al. 2008 An unusual haplotype structure on human chromosome 8p23 derived from the inversion polymorphism. Hum. Mutat. 29:1209–1216. 18473345 10.1002/humu.20775

[evl312-bib-0018] DePristo, M. A. , E. Banks , R. Poplin , K. V. Garimella , J. R. Maguire , C. Hartl , et al. 2011 A framework for variation discovery and genotyping using next‐generation DNA sequencing data. Nat Genet 43:491–498. 21478889 10.1038/ng.806 PMC3083463

[evl312-bib-0019] Ellegren, H. , L. Smeds , R. Burri , P. I. Olason , N. Backström , T. Kawakami , et al. 2012 The genomic landscape of species divergence in Ficedula flycatchers. Nature 491:756–760. 23103876 10.1038/nature11584

[evl312-bib-0020] English, A. C. , W. J. Salerno , and J. G. Reid . 2014 PBHoney: identifying genomic variants via long‐read discordance and interrupted mapping. BMC Bioinform. 15:180. 10.1186/1471-2105-15-180 PMC4082283 24915764

[evl312-bib-0021] Estrada, C. , and C. D. Jiggins . 2002 Patterns of pollen feeding and habitat preference among *Heliconius* species. Ecol. Entomol. 27:448–456.

[evl312-bib-0022] Faria, R. , and A. Navarro . 2010 Chromosomal speciation revisited: rearranging theory with pieces of evidence. Trends Ecol. Evol. 25:660–669. 20817305 10.1016/j.tree.2010.07.008

[evl312-bib-0094] Farré, M. , Micheletti, D. , & A. Ruiz‐Herrera . 2013 Recombination rates and genomic shuffling in human and chimpanzee–a new twist in the chromosomal speciation theory. Evol. 30:853–864. 10.1093/molbev/mss272 PMC3603309 23204393

[evl312-bib-0023] Feder, J. L. , and P. Nosil . 2009 Chromosomal inversions and species differences: when are genes affecting adaptive divergence and reproductive isolation expected to reside within inversions? Evolution 63:3061–3075. 19656182 10.1111/j.1558-5646.2009.00786.x

[evl312-bib-0024] Feder, J. L. , R. Gejji , T. H. Q. Powell , and P. Nosil . 2011 Adaptive chromosomal divergence driven by mixed geographic mode of evolution. Evolution 65:2157–2170. 21790566 10.1111/j.1558-5646.2011.01321.x

[evl312-bib-0025] Feder, J. L. , P. Nosil , and S. M. Flaxman . 2014 Assessing when chromosomal rearrangements affect the dynamics of speciation: implications from computer simulations. Front Genet. 5:295. 25206365 10.3389/fgene.2014.00295 PMC4144205

[evl312-bib-0026] Felsenstein, J. 1981 Skepticism towards Santa Rosalia, or why are there so few kinds of animals? Evolution 35:124–138. 28563447 10.1111/j.1558-5646.1981.tb04864.x

[evl312-bib-0027] Fishman, L. , A. Stathos , P. M. Beardsley , C. F. Williams , and J. P. Hill . 2013 Chromosomal rearrangements and the genetics of reproductive barriers in *Mimulus* (monkey flowers). Evolution 67:2547–2560. 24033166 10.1111/evo.12154

[evl312-bib-0028] Fontaine, M. C. , J. B. Pease , A. Steele , R. M. Waterhouse , D. E. Neafsey , I. V. Sharakhov , et al. 2015 Extensive introgression in a malaria vector species complex revealed by phylogenomics. Science 347:1258524‐1–1258524‐6. 25431491 10.1126/science.1258524 PMC4380269

[evl312-bib-0029] Guerrero, R. F. , F. Rousset , and M. Kirkpatrick . 2012 Coalescent patterns for chromosomal inversions in divergent populations. Phil. Trans. R. Soc. B 367:430–438. 22201172 10.1098/rstb.2011.0246 PMC3233714

[evl312-bib-0030] Heliconius Genome Consortium . 2012 Butterfly genome reveals promiscuous exchange of mimicry adaptations among species. Nature 487:94–98. 22722851 10.1038/nature11041 PMC3398145

[evl312-bib-0031] Hoffmann, A. A. , and L. H. Rieseberg . 2008 Revisiting the impact of inversions in evolution: from population genetic markers to drivers of adaptive shifts and speciation? Annu. Rev. Ecol. Syst. 39:21–42. 10.1146/annurev.ecolsys.39.110707.173532 PMC2858385 20419035

[evl312-bib-0032] Hooper, D. M. 2016 Range overlap drives chromosome inversion fixation in passerine birds. bioRxiv. 10.1101/053371.

[evl312-bib-0033] Huynh, L. Y. , D. L. Maney , and J. W. Thomas . 2011 Chromosome‐wide linkage disequilibrium caused by an inversion polymorphism in the white‐throated sparrow (*Zonotrichia albicollis*). Heredity 106:537–546. 20571514 10.1038/hdy.2010.85 PMC2950911

[evl312-bib-0034] Jiggins, C. D. 2008 Ecological speciation in mimetic butterflies. BioScience 58:541–548.

[evl312-bib-0035] Jiggins, C. D. , R. E. Naisbit , R. L. Coe , and J. L. B. Mallet . 2001 Reproductive isolation caused by colour pattern mimicry. Nature 411:302–305. 11357131 10.1038/35077075

[evl312-bib-0036] Jiggins, C. D. , J. Mavarez , M. Beltrán , W. O. McMillan , J. S. Johnston , and E. Bermingham . 2005 A genetic linkage map of the mimetic butterfly *Heliconius melpomene* . Genetics 171:557–570. 15489522 10.1534/genetics.104.034686 PMC1456771

[evl312-bib-0037] Jones, F. C. , M. G. Grabherr , Y. F. Chan , P. Russell , E. Mauceli , J. Johnson , et al. 2012 The genomic basis of adaptive evolution in threespine sticklebacks. Nature 484:55–61. 22481358 10.1038/nature10944 PMC3322419

[evl312-bib-0038] Joron, M. , L. Frezal , R. T. Jones , N. L. Chamberlain , S. F. Lee , C. R. Haag , et al. 2011 Chromosomal rearrangements maintain a polymorphic supergene controlling butterfly mimicry. Nature 477:203–206. 21841803 10.1038/nature10341 PMC3717454

[evl312-bib-0039] Kiełbasa, S. M. , R. Wan , K. Sato , P. Horton , and M. C. Frith . 2011 Adaptive seeds tame genomic sequence comparison. Genome Res 21:487–493. 21209072 10.1101/gr.113985.110 PMC3044862

[evl312-bib-0040] King, M. 1993 Species evolution: the role of chromosome change. Cambridge Univ. Press, Cambridge, U.K.

[evl312-bib-0041] Kirkpatrick, M. 2010 How and why chromosome inversions evolve. PLOS Biol 8:e1000501. 20927412 10.1371/journal.pbio.1000501 PMC2946949

[evl312-bib-0042] Kirkpatrick, M. , and B. Barrett . 2015 Chromosome inversions, adaptive cassettes and the evolution of species' ranges. Mol. Ecol. 24:2046–2055. 25583098 10.1111/mec.13074

[evl312-bib-0043] Kirkpatrick, M. , and N. Barton . 2006 Chromosome inversions, local adaptation and speciation. Genetics 173:419–434. 16204214 10.1534/genetics.105.047985 PMC1461441

[evl312-bib-0044] Korunes, K. L. , and M. A. F. Noor . 2017 Gene Conversion and Linkage: effects on genome evolution and speciation. Mol. Ecol. 26:351–364. 27337640 10.1111/mec.13736

[evl312-bib-0045] Kozak, K. M. , N. Wahlberg , A. F. E. Neild , K. K. Dasmahapatra , J. L. B. Mallet , and C. D. Jiggins . 2015 Multilocus species trees show the recent adaptive radiation of the mimetic *Heliconius* butterflies. Syst. Biol. 64:505–524. 25634098 10.1093/sysbio/syv007 PMC4395847

[evl312-bib-0046] Krimbas, C. B. and J. R. Powell . 1992 Drosophila inversion polymorphism. CRC Press, Boca Raton, FL.

[evl312-bib-0047] Kronforst, M. R. , M. E. B. Hansen , N. G. Crawford , J. R. Gallant , W. Zhang , R. J. Kulathinal , et al. 2013 Hybridization reveals the evolving genomic architecture of speciation. Cell Rep. 5:666–677. 24183670 10.1016/j.celrep.2013.09.042 PMC4388300

[evl312-bib-0048] Kurtz, S. , A. Phillippy , A. L. Delcher , M. Smoot , M. Shumway , C. Antonescu , and S. L. Salzberg . 2004 Versatile and open software for comparing large genomes. Genome Biol. 5:R12. 14759262 10.1186/gb-2004-5-2-r12 PMC395750

[evl312-bib-0049] Lavoie, C. A. , R. N. Platt , P. A. Novick , B. A. Counterman , and D. A. Ray . 2013 Transposable element evolution in *Heliconius* suggests genome diversity within Lepidoptera. Mobile DNA 4:21. 24088337 10.1186/1759-8753-4-21 PMC4016481

[evl312-bib-0050] Li, H. 2011 A statistical framework for SNP calling, mutation discovery, association mapping and population genetical parameter estimation from sequencing data. Bioinformatics 27:2987–2993. 21903627 10.1093/bioinformatics/btr509 PMC3198575

[evl312-bib-0051] Li, H. 2013 Aligning sequence reads, clone sequences and assembly contigs with BWA‐MEM. arXiv 1303.3997v2

[evl312-bib-0052] Lohse, K. , M. Clarke , M. G. Ritchie , and W. J. Etges . 2015 Genome‐wide tests for introgression between cactophilic *Drosophila* implicate a role of inversions during speciation. Evolution 69:1178–1190. 25824653 10.1111/evo.12650 PMC5029762

[evl312-bib-0053] Love, R. R. , A. M. Steele , M. B. Coulibaly , S. F. Traorè , S. J. Emrich , M. C. Fontaine , and N. J. Besansky . 2016 Chromosomal inversions and ecotypic differentiation in *Anopheles gambiae*: the perspective from whole‐genome sequencing. Mol. Ecol. 25:5889–5906. 27759895 10.1111/mec.13888 PMC5130611

[evl312-bib-0054] Lunter, G. , and M. Goodson . 2011 Stampy: a statistical algorithm for sensitive and fast mapping of Illumina sequence reads. Genome Res. 21:936–939. 20980556 10.1101/gr.111120.110 PMC3106326

[evl312-bib-0055] Machado, C. A. , T. S. Haselkorn , and M. A. F. Noor . 2007 Evaluation of the genomic extent of effects of fixed inversion differences on intraspecific variation and interspecific gene flow in *Drosophila pseudoobscura* and *D. persimilis* . Genetics 175:1289–1306. 17179068 10.1534/genetics.106.064758 PMC1840060

[evl312-bib-0056] Malinsky, M. , J. T. Simpson , and R. Durbin . 2016 trio‐sga: facilitating de novo assembly of highly heterozygous genomes with parent‐child trios. bioRxiv. 10.1101/051516

[evl312-bib-0057] Mallet, J. L. B. , and N. H. Barton . 1989 Strong natural selection in a warning‐color hybrid zone. Evolution 43:421–431. 28568556 10.1111/j.1558-5646.1989.tb04237.x

[evl312-bib-0058] Mallet, J. L. B. , M. Beltrán , W. Neukirchen , and M. Linares . 2007 Natural hybridization in heliconiine butterflies: the species boundary as a continuum. BMC Evol. Biol. 7:28. 17319954 10.1186/1471-2148-7-28 PMC1821009

[evl312-bib-0059] Martin, S. H. , K. K. Dasmahapatra , N. J. Nadeau , C. Salazar , J. R. Walters , F. Simpson , et al. 2013 Genome‐wide evidence for speciation with gene flow in *Heliconius* butterflies. Genome Res. 23:1817–1828. 24045163 10.1101/gr.159426.113 PMC3814882

[evl312-bib-0060] Martin, S. H. , J. W. Davey , and C. D. Jiggins . 2015a Evaluating the use of ABBA‐BABA statistics to locate introgressed loci. Mol. Biol. Ecol. 32:244–257. 10.1093/molbev/msu269 PMC4271521 25246699

[evl312-bib-0061] Martin, S. H. , A. Eriksson , K. M. Kozak , and A. Manica . 2015b Speciation in Heliconius butterflies: minimal contact followed by millions of generations of hybridisation. bioRxiv. 10.1101/015800

[evl312-bib-0062] Martin, S. H. , M. Möst , W. J. Palmer , C. Salazar , W. O. McMillan , F. M. Jiggins , and C. D. Jiggins . 2016 Natural Selection and Genetic Diversity in the Butterfly *Heliconius melpomene* . Genetics 203:525–541. 27017626 10.1534/genetics.115.183285 PMC4858797

[evl312-bib-0063] McGaugh, S. E. , and M. A. F. Noor . 2012 Genomic impacts of chromosomal inversions in parapatric *Drosophila* species. Phil. Trans. R. Soc. B 367:422–429. 22201171 10.1098/rstb.2011.0250 PMC3233717

[evl312-bib-0064] Merrill, R. M. , Z. Gompert , L. M. Dembeck , M. R. Kronforst , W. O. McMillan , and C. D. Jiggins . 2011a Mate preference across the speciation continuum in a clade of mimetic butterflies. Evolution 65:1489–1500. 21521198 10.1111/j.1558-5646.2010.01216.x

[evl312-bib-0065] Merrill, R. M. , B. Van Schooten , J. A. Scott , and C. D. Jiggins . 2011b Pervasive genetic associations between traits causing reproductive isolation in *Heliconius* butterflies. Proc. R. Soc. B 278:511–518. 10.1098/rspb.2010.1493 PMC3025683 20810445

[evl312-bib-0066] Merrill, R. M. , R. W. R. Wallbank , V. Bull , P. C. A. Salazar , J. L. B. Mallet , M. Stevens , and C. D. Jiggins . 2012 Disruptive ecological selection on a mating cue. Proc. R. Soc. B. 279:4907–4913. 10.1098/rspb.2012.1968 PMC3497240 23075843

[evl312-bib-0067] Merrill, R. M. , R. E. Naisbit , J. L. B. Mallet , and C. D. Jiggins . 2013 Ecological and genetic factors influencing the transition between host‐use strategies in sympatric *Heliconius* butterflies. J. Evol. Biol. 26:1959–1967. 23961921 10.1111/jeb.12194

[evl312-bib-0068] Merrill, R. M. , K. K. Dasmahapatra , J. W. Davey , D. D. Dell'Aglio , J. J. Hanly , B. Huber , et al. 2015 The diversification of *Heliconius* butterflies: what have we learned in 150 years? J. Evol. Biol. 28:1417–1438. 26079599 10.1111/jeb.12672

[evl312-bib-0069] Nachman, M. W. , and B. A. Payseur . 2012 Recombination rate variation and speciation: theoretical predictions and empirical results from rabbits and mice. Phil. Trans. R. Soc. B 367:409–421. 22201170 10.1098/rstb.2011.0249 PMC3233716

[evl312-bib-0070] Nadeau, N. J. , M. Ruiz , P. Salazar , B. Counterman , J. A. Medina , H. Ortiz‐Zuazaga , et al. 2014 Population genomics of parallel hybrid zones in the mimetic butterflies, *H. melpomene* and *H. erato* . Genome Res. 24:1316–1333. 24823669 10.1101/gr.169292.113 PMC4120085

[evl312-bib-0071] Naisbit, R. E. , C. D. Jiggins , and J. L. B. Mallet . 2001 Disruptive sexual selection against hybrids contributes to speciation between *Heliconius cydno* and *Heliconius melpomene* . Proc. R. Soc. B 268:1849–1854. 10.1098/rspb.2001.1753 PMC1088818 11522205

[evl312-bib-0072] Naisbit, R. E. , C. D. Jiggins , M. Linares , C. Salazar , and J. L. B. Mallet . 2002 Hybrid sterility, Haldane's rule and speciation in *Heliconius cydno* and *H. melpomene* . Genetics 161:1517–1526. 12196397 10.1093/genetics/161.4.1517 PMC1462209

[evl312-bib-0073] Naisbit, R. E. , C. D. Jiggins , and J. L. B. Mallet . 2003 Mimicry: developmental genes that contribute to speciation. Evol. Dev. 5:269–280. 12752766 10.1046/j.1525-142x.2003.03034.x

[evl312-bib-0074] Navarro, A. , and N. H. Barton . 2003a Accumulating postzygotic isolation genes in parapatry: a new twist on chromosomal speciation. Evolution 57:447–459. 12703935 10.1111/j.0014-3820.2003.tb01537.x

[evl312-bib-0075] Navarro, A. , and N. H. Barton 2003b Chromosomal speciation and molecular divergence—accelerated evolution in rearranged chromosomes. Science 300:321–324. 12690198 10.1126/science.1080600

[evl312-bib-0076] Noor, M. A. F. , K. L. Grams , L. A. Bertucci , and J. Reiland . 2001 Chromosomal inversions and the reproductive isolation of species. Proc. Natl. Acad. Sci. USA 98:12084–12088. 11593019 10.1073/pnas.221274498 PMC59771

[evl312-bib-0077] Noor, M. A. F. , D. A. Garfield , S. W. Schaeffer , and C. A. Machado . 2007 Divergence between the *Drosophila pseudoobscura* and *D. persimilis* genome sequences in relation to chromosomal inversions. Genetics 177:1417–1428. 18039875 10.1534/genetics.107.070672 PMC2147956

[evl312-bib-0078] Ono, Y. , K. Asai , and M. Hamada . 2013 PBSIM: PacBio reads simulator–toward accurate genome assembly. Bioinformatics 29:119–121. 23129296 10.1093/bioinformatics/bts649

[evl312-bib-0079] Ortíz‐Barrientos, D. , J. Engelstädter , and L. H. Rieseberg . 2016 Recombination rate evolution and the origin of species. Trends Ecol. Evol. 31:226–236. 26831635 10.1016/j.tree.2015.12.016

[evl312-bib-0080] Pinharanda, A. , S. H. Martin , S. L. Barker , J. W. Davey , and C. D. Jiggins . 2017 The comparative landscape of duplications in *Heliconius melpomene* and *Heliconius cydno* . Heredity 118:78–87. 27925618 10.1038/hdy.2016.107 PMC5176112

[evl312-bib-0081] Pringle, E. G. , S. W. Baxter , C. L. Webster , A. Papanicolaou , S. F. Lee , and C. D. Jiggins . 2007 Synteny and chromosome evolution in the Lepidoptera: evidence from mapping in *Heliconius melpomene* . Genetics 177:417–426. 17603110 10.1534/genetics.107.073122 PMC2013725

[evl312-bib-0082] Rastas, P. , F. C. F. Calboli , B. Guo , T. Shikano , and J. Merilä . 2016 Construction of ultradense linkage maps with Lep‐MAP2: stickleback F2 recombinant crosses as an example. Genome Biol. Evol. 8:78–93. 10.1093/gbe/evv250 PMC4758246 26668116

[evl312-bib-0083] Rieseberg, L. H. 2001 Chromosomal rearrangements and speciation. Trends Ecol. Evol. 16:351–358. 11403867 10.1016/s0169-5347(01)02187-5

[evl312-bib-0084] Samonte, R. V. , and E. E. Eichler . 2002 Segmental duplications and the evolution of the primate genome. Nat. Rev. Genet. 3:65–72. 11823792 10.1038/nrg705

[evl312-bib-0085] Seehausen, O. , R. K. Butlin , I. Keller , C. E. Wagner , J. W. Boughman , P. A. Hohenlohe , et al. 2014 Genomics and the origin of species. Nat. Rev. Genet. 15:176–192. 24535286 10.1038/nrg3644

[evl312-bib-0086] Sekhon, J. S. 2011 Multivariate and propensity score matching software with automated balance optimization: the matching package for R. J. Stat. Softw. 42:1–52.

[evl312-bib-0087] Servedio, M. R. , G. S. Van Doorn , M. Kopp , A. M. Frame , and P. Nosil . 2011 Magic traits in speciation: “magic” but not rare? Trends Ecol. Evol. 26:389–397. 21592615 10.1016/j.tree.2011.04.005

[evl312-bib-0088] Smadja, C. M. , and R. K. Butlin . 2011 A framework for comparing processes of speciation in the presence of gene flow. Mol. Ecol. 20:5123–5140. 22066935 10.1111/j.1365-294X.2011.05350.x

[evl312-bib-0089] Stevison, L. S. , K. B. Hoehn , and M. A. F. Noor . 2011 Effects of inversions on within‐ and between‐species recombination and divergence. Genome Biol. Evol. 3:830–841. 21828374 10.1093/gbe/evr081 PMC3171675

[evl312-bib-0090] Supple, M. , R. Papa , B. Counterman , and W. O. McMillan . 2013 The genomics of an adaptive radiation: insights across the *Heliconius* speciation continuum. Adv. Exp. Med. Biol. 781:249–271. 10.1007/978-94-007-7347-9_13 24277304

[evl312-bib-0091] Turner, J. R. G. , and P. M. Sheppard . 1975 Absence of crossing‐over in female butterflies (*Heliconius*). Heredity 34:265–269. 1055712 10.1038/hdy.1975.29

[evl312-bib-0092] Van Belleghem, S. M. , P. Rastas , A. Papanicolaou , S. H. Martin , C. F. Arias , M. A. Supple , et al. 2017 Complex modular architecture around a simple toolkit of wing pattern genes. Nat. Ecol. Evol. 1. 10.1038/s41559-016-0052 PMC5432014 28523290

[evl312-bib-0093] Wallbank, R. W. R. , S. W. Baxter , C. Pardo‐Diaz , J. J. Hanly , S. H. Martin , J. L. B. Mallet , et al. 2016 Evolutionary novelty in a butterfly wing pattern through enhancer shuffling. PLOS Biol. 14:e1002353. 26771987 10.1371/journal.pbio.1002353 PMC4714872

[evl312-bib-0095] White, M. J. D. 1978 Modes of speciation. Freeman & Co, San Francisco, CA.

[evl312-bib-0096] Ziegler, A. , and I. R. König . 2001 Genetic distance and mapping functions. eLS. 10.1038/npg.els.0005399

