# Supplementary material for: No evidence for maintenance of a sympatric Heliconius species barrier by chromosomal inversions
Source: Evol Lett. 2017 Jun 14;1(3):138–54. doi: 10.1002/evl3.12 (PMC6122123; doi:10.1002/evl3.12)
Supplement: Supplementary file 1 — Supporting information [file EVL3-1-138-s001.docx]

**No evidence for maintenance of a sympatric *Heliconius* species barrier by chromosomal inversions**

John W. Davey^1,2*^, Sarah L. Barker^1^, Pasi M. Rastas^1^, Ana Pinharanda^1,2^, Simon H. Martin^1^, Richard Durbin^3^, W. Owen McMillan^2^, Richard M. Merrill^1,2^, Chris D. Jiggins^1,2*^

**Supporting Information**

*Supporting Figure and Table Legends*

*Supporting Methods*

Crosses

Dissection and DNA extraction

RAD Sequencing library preparation

RAD library sequencing and alignment

Pacific Biosciences library prep and sequencing

Linkage mapping: within-species crosses

Linkage mapping: hybrid crosses

*Supporting Notes*

Variation in SNP density

Crossover detection

Absence of female recombination

**Supporting Figure and Table Legends**

**Figure S1. Cross design.** Within-species crosses for *H. melpomene* (red) and *H. cydno* (blue) were each 3 F_1_ crosses of stock females to wild males. *H. cydno* x *H. melpomene* hybrid crosses (green) were 18 backcrosses of F_1_ hybrid males to stock *H. cydno* females, with F_1_ hybrids produced from four matings of stock *H. cydno* females to wild *H. melpomene* males. PacBio+Trio, cross sequencing was supplemented with PacBio sequencing of pools of male and female larvae and a parent-offspring trio assembly (see Methods). N/A, parent was lost or could not be sequenced.

**Figure S2. Genetic and physical maps for each ordered Hmel2 chromosome.** Thick green, red and orange bars show Hmel2 scaffolds ordered on chromosomes, labelled with names. Green scaffolds are ordered and oriented, orange are ordered but not oriented, red are placed but not ordered or oriented. Vertical lines show SNPs placed on scaffolds; maternal SNPs, above scaffolds in black; paternal SNPs, below scaffolds in blue and orange. Paternal SNPs are connected by dotted lines to the genetic marker they are assigned to, with alternating markers coloured blue and orange.

**Figure S3. SNP density, PstI site density and GC content for each ordered Hmel2 chromosome.** SNP density for all crosses combined for each species.

**Figure S4.** **Marey maps of recombinations for each cross separately.** Crosses listed in Table 1, Tables S1-S3 and Figure S1. *H. melpomene*, red; *H. cydno*, blue; *H. cydno* x *H. melpomene* hybrids, green. Chromosomes 1-21 of *H. melpomene* genome assembly version 2 (Hmel2) shown against cumulative cM values for each set of crosses.

**Figure S5.** **Recombination rates in cM/Mb for each ordered Hmel2 chromosome.** Rates in 1 Mb windows sliding by 100 kb. *H. melpomene*, red; *H. cydno*, blue; hybrids, green. Lines show true values; shaded areas show 95% bootstrap confidence intervals, 10 000 iterations.

**Figure S6.** **Gaps in linkage maps where lack of precise recombination data cannot rule out presence of inversions.** Each page shows data for one chromosome with *H. cydno* in blue and *H. melpomene* in red. Heights and lengths of lines show gap lengths.

**Figure S7.** **Histograms of gap lengths for *H. cydno* and *H. melpomene*.**

**Figure S8. Probability of detecting random inversions of sizes from 10 kb to 1.5 Mb given existing linkage maps for *H. melpomene* and *H. cydno*.**

**Figure S9. Histograms of raw read lengths for Pacific Biosciences sequencing.** *H. cydno* females, blue; *H. cydno* males, light blue; *H. melpomene* females, red; *H. melpomene* males, orange.

**Figure S10.** **Histograms of base depths across the genome after alignment of raw PacBio reads to *H. melpomene* genome assembly Hmel2.** Colours as for Figure S9.

**Figures S11-17.** **Full evidence for each candidate inversion group, separated into the classes shown in Figure 3 and Table 4.** **S11**, *H. cydno*, Split reads and trio assembly. **S12**, *H. cydno*, Split reads only. **S13**, *H. melpomene*, Split reads and trio assembly. **S14**, *H. melpomene*, Split reads only. **S15**, Both species, Split reads and trio assembly. **S16**, Both species, Split reads only. **S17**, Both species, Split reads in one species, trio assembly in both.

Each page shows linkage map, split read, trio assembly and population genetics evidence for each candidate inversion group, across a region of the Hmel2 genome assembly. Black lozenge, candidate inverted region (white text in lozenge shows length of region). **Genome**, contigs from Hmel1 and Hmel2 shown in alternating dark and light grey (labels show Hmel1 scaffold names and Hmel2 contig names). Thin black lines in Hmel2 contigs show repeat-masked regions of the genome. Red bars show features from the Hmel2 annotation. **Linkage map**, SNPs from linkage maps shown as circles connected by Marey map lines. Thick lines show regions with no recombination; thin lines span regions between SNPs with different markers, indicating a recombination happens somewhere along the line but the position cannot be resolved any further. *H. cydno*, blue; *H. melpomene*, red; hybrids, green. **PBHoney Candidates**, rounded lines show candidate inversions called by PBHoney; number to the right shows number of reads supporting each candidate. Light shaded rectangles show ranges of these inversions across the plot for comparison with trio alignments and contig boundaries. *H. cydno* females, blue; *H. cydno* males, light blue; *H. melpomene* females, red; *H. melpomene* males, orange. **Trio** strips, alignments of trio scaffolds to candidate inversion regions. Arrowed lines show individual alignments; shaded rectangles enclosing arrowed lines show all alignments for a particular scaffold, with scaffold name to the right of each shaded rectangle. Colours as per PBHoney candidates. **Trio Inverted**, trio scaffolds with multiple alignments across the candidate inversion, with forward and reverse alignments either side of candidate breakpoints. **Trio Spanning**, trio scaffolds with single alignments spanning any one PBHoney candidate. **Trio Edges**, trio scaffolds with alignments spanning one candidate breakpoint, but not the whole inversion. **Population genetics**, *F_ST_* (magenta), *f_d_* (green) and *d_XY_* (yellow) for windows across the candidate inversion. Black lozenge, inverted region, matching lozenge in main plot. Length and number label of grey lozenges at the bottom show number of sites used for calculations in each window. Open circles show windows were calculation is not possible (either no sites present, or D<0 for *f_d_*).

**Figure S18.** **Oxford grids for ordered Hmel2 scaffolds and *H. erato* scaffolds.** Collinear regions in red; inverted regions in blue. Scaffold boundaries separated by grey lines. Black and dark grey lines on right and lower axes show alternating markers from linkage maps; light grey shaded rectangles show these markers across the plot.

**Table S1. Full sample details for *H. melpomene* crosses.** All samples were RAD sequenced except for underlined samples, which were whole genome sequenced and trio assembled. Samples shaded in grey were rejected due to low coverage or many erroneous genotypes. Estimated coverage is for read 1 RAD stacks, assuming 27,000 PstI cut sites in *Heliconius melpomene,* or, for whole genome samples, assuming a 292 Mb genome for 125bp read pairs. ENA = European Nucleotide Archive, <http://www.ebi.ac.uk/ena>.

**Table S2. Full sample details for *H. cydno* crosses.** Key as for Table S1.

**Table S3. Full sample details for *H. cydno* x *H. melpomene* hybrid crosses.** Each cross involved multiple backcrosses. Many parents were not preserved or sequenced. All samples were sequenced with 100bp paired end RAD sequencing except for those in bold, sequenced with 50bp single end RAD sequencing. Otherwise, key as for Table S1.

**Table S4. SNP and marker information for within-species crosses.** Method Steps refer to numbered cross-references in Supplementary Methods.

**Table S5. SNP and marker information for hybrid crosses.** Method Steps refer to numbered cross-references in Supplementary Methods.

**Table S6.** **Counts of raw and filtered PBHoney candidate inversions.**

**Table S7. Locations of candidate inversions.**

**Table S8. Locations of known trait loci.**

**Supporting Methods**

*Crosses*

Crosses were prepared in Smithsonian Tropical Research Institute insectaries in Gamboa, Panama. Individuals used to establish stock and for crosses were collected from Gamboa (987.4˚ N, 79842.2˚ W, elevation 60 m) and the nearby Soberania National Park, República de Panamá. For the within-species crosses of *H. melpomene rosina* and *H. cydno chioneus*, wild males were mated to virgin stock females. Females were placed alone in a cage for laying with access to *Psiguria* flowers, *Lantana camara*, and artificial feeders containing a 20% sugar-water solution with 5% added commercial pollen, changed every other day. All laying females were provided with *Passiflora* plants (*H. melpomene*, *P. menispermifolia*; *H. cydno*, *P. edulis*, *P. vitifolia*, *P. triloba*, *P. quadragulata*) with fresh shootings for laying. Eggs were collected every day, and separated into individual plastic pots to prevent cannibalism. Larvae were reared individually on fresh *Passiflora* leaves until late 3rd or early 4th instar, preserved in 2ml of 20% DMSO and 0.25M EDTA (pH 8.0) and stored at -20ºC. Adults were preserved and stored in the same way after dissecting wings. Adult males were preserved after mating; adult females were preserved when egg laying ceased. Interspecific crosses were achieved by placing pupae from a *H. cydno* stock in a cage of wild *H. melpomene* males. Hybrid individuals used to produce linkage maps were then obtained from backcrosses produced by mating F1 males to females from the *H. cydno* stock. As with the intraspecific crosses, eggs were collected daily, and larvae were then raised individually; however, unlike the intraspecific crosses, individuals were raised to adults. Some parents were lost in the insectaries, most often due to ants or other predators, or sufficient DNA could not be extracted for successful sequencing (Figure S1).

*Dissection and DNA extraction*

All dissections were performed with a new sterile scalpel, fresh Parafilm, and tweezers washed in 80% ethanol. For adults, the thorax was dissected away from the head and abdomen and cut in half along the median plane. One half of thorax was used for DNA extraction with the remaining tissue returned to storage. Larvae were cut in half along the median plane and gut contents removed, with one half used for extraction and the other returned to storage. Some larvae were too small to be cut along the median plane and were cut along a transverse plane instead, whereas some halves of large larvae were cut into smaller pieces to fit within kit tissue size limits.

DNA for all RAD sequenced samples was extracted with the QIAGEN DNeasy Blood & Tissue kit (69504) following manufacturer’s instructions for animal tissue. DNA for Pacific Biosciences sequenced samples was extracted with the QIAGEN MagAttract HMW DNA kit (67563) following manufacturer’s instructions for fresh or frozen tissue, with overnight lysis of tissue. DNA quantity, purity and size distribution were measured using a Qubit Fluorometer (Q32857), Nanodrop Spectrophotometer (ND-1000) and Agilent 2100 Bioanalyzer (G2939A).

*RAD Sequencing library preparation*

RAD libraries were prepared to contain 96 samples in 6 batches of 16, with each sample ligated to one of 16 P1 adapters and each batch ligated to one of 8 P2 adapters, with each adapter featuring an 8bp barcode (adapter primers provided by IDT). 500ng of DNA for each sample was diluted to 35uL and digested with NEB high fidelity PstI enzyme (1.0uL PstI-HF, 5.0uL NEB Cutsmart buffer, 9.0uL dH_2_O), incubating for 60 minutes at 37°C and denaturing for 20 minutes at 80°C. Samples were ligated to P1 adapters (add 4ul of 100 nM P1 adapter, 1uL NEB Buffer 2, 0.6uL 100mM rATP, 1.0uL T4 Quick Ligase and 3.4uL dH2O to 50ul DNA solution, incubate for 60 minutes at 22°C and denature for 10 minutes at 65°C), pooled, sheared using a Diagenode Bioruptor (UCD 200 TM) for 8 minutes (30s on, 30s off) and cleaned up with the Nucleospin kit following manufacturer’s instructions (Machery Nagel 740609.50). Libraries were size selected to 250-700bp using a Sage Science BluePippin system (BLU0001) with 1.5% agarose cassettes (BDF1510). 38uL of the resulting library was blunted with the NEB Quick Blunting kit (NEB E1201L) (adding 5uL 10x Blunting Buffer, 5uL 1mM dNTPs, 2uL Blunting Enzyme and incubating for 30 minutes at 30°C) and purified (mix 0.8x Ampure XP beads (Beckman Coulter A63880) to 1x DNA, vortex briefly, incubate at room temperature for 5 minutes and place in magnetic rack for 3-5 minutes; remove supernatant and wash in 250uL 80% ethanol twice; dry beads, add 42uL elution buffer and resuspend; return to magnetic rack and remove 41uL of eluate).

Libraries were A-tailed (add 5uL 10x NEB Buffer 2, 1uL 100mM dATP, 3uL exo-Klenow fragment (NEB E6044) and incubate for 30 minutes at 37°C, then purify as before to 40.5uL eluate) and P2 adapters were ligated (add 3uL 10uM P2 adapter, 5uL 10x NEB Buffer 2, 0.5uL 100mM rATP, 2uL T4 Quick Ligase (NEB M2200L) and leave for 60 minutes at room temperature); samples were purified as above and quantified, to a final yield of 5-20 ng/uL. Libraries were amplified by PCR using P1 and P2 adapter primers for 12-16 cycles depending on initial yield, and using P2 primers with different barcodes for each batch of 16 P1-ligated samples. Each batch was amplified in 8 separate reactions (to increase diversity of initial samples and reduce sequencing of PCR duplicates) and pooled. Amplified samples were purified as above and eluted in EB-Tween 0.1%.

*RAD library sequencing and alignment*

RAD libraries were sequenced by BGI using the Illumina HiSeq 2500 (hybrids and *H. cydno* samples) and HiSeq 4000 (*H. melpomene* samples) to produce 100 bp reads. FASTQ files were demultiplexed using process_radtags in Stacks v1.34 (Catchen *et al.* 2013) allowing 2 mismatches between barcodes. Sequences were aligned to version 2 of the *H. melpomene* genome (Hmel2 (Davey *et al.* 2016)) using Stampy (Lunter *et al.* 2011) v1.0.23 with options --substitutionrate=0.01, --gatkcigarworkaround, --baq and --alignquals, and then processed by Picard MarkDuplicates (v1.135 (<http://broadinstitute.github.io/picard/>)) and GATK IndelRealigner (v3.4.0 (dePristo *et al.* 2011)). Genotype posteriors were called with SAMtools mpileup (v1.2 (Li 2011)) requiring minimum mapping quality of 10 and minimum base quality of 10. A small number of individuals were removed during linkage map construction due to low coverage or large numbers of erroneous genotype calls in markers (light grey samples in Tables S1-S3).

*Pacific Biosciences library prep and sequencing*

Larvae from H. cydno cross 1 and H. melpomene cross 2 were sexed by examining the segregation of the Z chromosome where linkage maps were available at time of sequencing, or the ratio of average autosomal RAD sequencing read coverage to sex chromosome read coverage. Pools of 12 males and 12 females were constructed, using larvae with the highest DNA yields. 12 individuals were used per pool to achieve sufficient yield for Pacific Biosciences sequencing and to approach even coverage of the four parental genomes (insufficient DNA was available to generate long read sequences from parents directly). Each pool was size selected to fragments greater than 6 kb using the SageELF system. Libraries were prepared following the standard 10 kb protocol (SMRTbell Template Prep Kit 1.0, 100-259-100) but with only two bead clean ups at the end rather than three. Libraries were sequenced on a PacBio RSII machine with P6/C4 reagents for 3-4 hour run times. Subreads were used for analyses.

*Linkage mapping: within-species crosses*

Within-species linkage maps for *H. melpomene* and *H. cydno* were built with Lep-MAP2 (Rastas *et al.* 2016; downloaded from <https://sourceforge.net/p/lepmap2>, commit d91aa7, 18 January 2016) and some modules from Lep-MAP3 (noted below; <https://sourceforge.net/projects/lep-map3/>). The number of SNPs and/or markers produced by each step of the process are reported in Table S4, according to the step numbers listed here. For each cross, sites were filtered with Lep-MAP2 pileupParser2.awk and Lep-MAP2 pileup2posterior.awk, requiring each SNP to be sequenced at least 3 times for 90% of individuals, and requiring each allele to be present in at least 5% of individuals (step 1). Missing parental genotypes were called with the Lep-MAP2 ParentCall module with options ZLimit=2.0 and removeNonInformative=1 (step 2).

Sites were clustered into markers by filtering for segregation distortion with the Lep-MAP3 Filtering module with option dataTolerance=0.01 (distortion by chance 1:100; step 3) and then processing by Lep-MAP3 SeparateIdenticals (step 4), setting lodLimit options to 20 for maternal markers, log_10_ 2^(*n*-4) for paternal markers and log_10_ 3^(*n*-4) for intercross markers (*n* = number of individuals in the cross; calculation based on 2 possible genotypes for paternal markers, 3 for intercross markers, and allowing for 4 missing individuals), betweenSameType=1, lod3Mode=2, keepRate=1, numParts=2. Markers were then passed through Lep-MAP2 OutputData with option sizeLimit=3 (retaining only those markers found at at least three sites; step 5). Filtered, clustered markers were then separated into linkage groups using Lep-MAP2 SeparateChromosomes, setting lodLimit empirically for each cross to produce 21 linkage groups (step 6). Markers for each linkage group were then ordered with Lep-MAP2 OrderMarkers, setting maxDistance=0.1, initRecombination=0.05,0.000001, and learnRecombinationParameters=1,0 (step 7).

Initial marker orderings were manually reviewed and edited to correct misorderings, including removal of low quality markers causing disorder, and to ensure linkage groups were syntenic with Hmel2 chromosomes, using script clean_Lep-MAP_output.pl (step 8; this script and all others mentioned below are available; see Data availability section for details). Maps were then improved in two directions by script assign_snps_to_markers.pl (step 9): i) using Lep-MAP3 module JoinSingles2, all SNPs were reassigned to the set of cleaned markers, to extend the coverage of the linkage map across Hmel2 scaffolds; ii) where two consecutive markers differed by multiple recombinations, all possible paths between the two markers were generated and checked against JoinSingles2 output, with the most likely path being included in the map.

*Linkage mapping: hybrid crosses*

Due to the more complex cross structure of backcross populations (four pairs of grandparents and 18 pairs of F_1_ fathers and F_0_ mothers; Figure S1), smaller cross sizes for each backcross compared to within-species crosses, and varying sequence quality for some crosses, different methods were used to construct linkage maps for the *H. cydno* x *H. melpomene* hybrid crosses, now incorporated into Lep-MAP3 (<https://sourceforge.net/projects/lep-map3/>). The number of SNPs and/or markers produced by each step of the process are listed in Table S5, according to the step numbers listed here. SNPs were required to be sequenced at least 3 times for 80% of individuals, requiring 1200 total reads across all individuals and at least two alleles with minimum read coverage of 60 each (step 1). Lep-MAP3 module ParentCallGrandparents was called on posterior data with parameters ZLimit=2 and removeNonInformative=1 to impute missing parental calls (step 2). Families with less than 3 offspring were removed. Markers with high segregation distortion (p<0.001, distortion by chance 1:1000) were removed with Lep-MAP3 module Filtering2, with parameter dataTolerance=0.001 (step 3). Markers were first separated to 21 chromosomes with Lep-MAP3 module SeparateChromosomes2 (step 4) with recombination rate 3% (parameter theta=0.03) and LOD score limit 35 (lodLimit=35). The 21 largest linkage groups were matched to *H. melpomene* chromosomes and additional markers included using Lep-MAP3 module JoinSingles2All with LOD score limit 30.

Marker positions for each chromosome were constructed using Lep-MAP3 module OrderMarkers2, using only paternally informative markers with recombination1 and interference1 parameters set to 0.000001 (step 5). The map inflation due to the dependency of adjacent posteriors was reduced by scaling the posteriors by 0.5 in log space (taking the square root) and limiting the minimum posterior probability to 0.1 (scale=0.5 minError=0.1). Maps for each family were examined manually, removing erroneous markers including some distorted markers that passed filtering due to small family sizes.

**Supporting Notes**

*Variation in SNP density*

The recognition site for PstI (CTGCAG) occurs 27,279 times in the 275.2 Mb ordered Hmel2 genome. As PstI is a symmetric cutter, we expect to find two RAD loci for every PstI site. Heterozygosity in *Heliconius* is sufficiently high that we expect the vast majority of RAD loci to contain at least one SNP, and so we expected our linkage maps to have resolution of approximately 10 kilobases. Density of SNPs in the final map is broadly consistent with this expectation, but varies by species and chromosome position (Figure S3, Table S4; mean paternal SNP density for *H. melpomene*, 6101.1 bp; *H. cydno*, 9043.8 bp; hybrids, 13642.4 bp). Sequencing depth strongly influences SNP density by cross; the total number of paternal SNPs contributing to the within-species crosses (Table S4) is significantly correlated with estimated coverage per RAD locus for each cross (Table 2, Table S1, Table S2; r=0.87, n=6, p<0.05). This effect may be exaggerated because we have used paired end RAD sequencing. SNPs can rarely be called reliably from the regions downstream of the RAD site covered by the second reads in each pair, because coverage is spread over a region of a few hundred bases. However, as coverage increases, it becomes possible to call more read 2 SNPs reliably.

The variation in SNP density by chromosome position is largely due to PstI site locations. The number of paternal SNPs per 1 Mb sliding window (100 kb steps) is correlated with number of PstI sites in both pure species and hybrids (Figure S3; *H. melpomene*, r=0.36; *H. cydno*, r=0.34; hybrids, r=0.55; all correlation tests n=2528, p<0.05 (Bonferroni-corrected for 3 tests)). Number of PstI sites is in turn correlated with GC content (Figure S3; r=0.35, n=2528, p<0.05), as expected for a GC-rich restriction site.

Illumina sequencing has a GC content bias, with read depths of regions with 40% GC content roughly double those of regions with 30% GC content (Benjamini and Speed 2012), largely due to differential PCR amplification. The *H.* melpomene genome has an average GC content of 32% and so our sequencing is expected to be affected by this bias. We minimised the number of PCR cycles during our library preparations but it is not possible to avoid PCR altogether for RAD sequencing, as the PCR step is required to amplify restriction-site-containing fragments to out-compete other genomic DNA fragments remaining in the library. Therefore we expect to find that higher sequencing depths not only increase SNP density overall but also have an exaggerated bias in SNP density in regions with high GC content and so increased density of restriction sites; this effect is visible when comparing *H. melpomene* to *H. cydno*, due to the higher coverage of the *H. melpomene* crosses (Figure S3).

However, we do not believe variation in SNP density has affected our results. If SNP density affected our ability to accurately identify crossovers, map length should increase with SNP density. But Table 2, Table 4 and Figure 2 show that total map lengths are highly consistent across all species, crosses and chromosomes. SNP density in 1 Mb sliding windows (100 kb steps as above) is either not significantly correlated with recombination rate (*H. cydno*, hybrids; p>0.05) or significantly but very weakly correlated (*H. melpomene*, r=0.08, p<0.05; all tests, n=2528, Bonferroni-corrected for 3 tests). While the mean distance between crossovers does vary between within-species crosses (Table S4), this variation is not correlated with SNP density or sequencing depth (both tests p>0.05, n=6). Therefore we believe our overall recombination rates are not biased by sequencing depth, PstI site occurrence or the resulting SNP density.

There are many other reasons why SNP density may vary. The more conservative methods used for our hybrid crosses compared to within-species crosses appears to have reduced overall SNP density and positional variation in SNP density (Figure S3). It is also likely that number of offspring, biological variation between species and individual parents, and different sequencing strategies (whole genome sequencing of trios, 100bp paired end RAD sequencing, and 50bp single end RAD sequencing) have also influenced SNP and marker density. But as we do not see an effect of SNP density on recombination rate, we have not attempted to model the influence of these other features, as we do not believe it will change our conclusions.

*Crossover detection*

*i) Crossover resolution and inversion resolution*

Two aspects of the power of our study need to be carefully distinguished. Firstly, there is the ability to precisely identify the location of a crossover in a particular individual. In our data, these locations will be defined by two consecutive SNPs that differ by at least one genotype. As these SNPs are highly unlikely to be found at consecutive bases in the genome, we can only associate the crossover with the region defined by the distance between the SNPs, and cannot identify the crossover location with any more precision. The mean distance between paternal SNPs that differ by at least one crossover is between 51 and 79 kilobases per cross (Table S4); we call this *crossover resolution* and discuss this result further below.

Secondly, there is the ability to rule out the presence of inversions in a region due to the presence of crossovers in a region and so the demonstrable absence of a reversal in the linkage map. This relates to the regions identified in Figure S6 and S7, where no crossovers are found and so the presence of inversions cannot be confirmed or rejected. This is our *inversion resolution*, which is distinct from crossover resolution because it also includes regions where we have many accurate SNPs but no crossovers; it is a factor of not just our SNP density but also the numbers of offspring in our crosses. These gaps do not necessarily reflect uncertainty; for any one of these gaps, and for any horizontal line on our Marey maps (Figures 2, S1), we can be confident that we have no recombinations in this region, because we have on average several tens of SNPs for each of these paternal markers (Figure S2) - unless we have systematically underestimated double crossovers, which is discussed below. But we cannot claim that recombination would never happen in these regions if we increased our sample sizes, and we cannot be sure there are no inversions in these regions from the linkage maps alone because without crossovers we cannot observe any possible reversal in the linkage maps.

*ii) Crossover resolution*

The crossover resolutions reported above are higher than our estimated SNP densities, due to the difficulty of distinguishing genuine variation from sequencing, genotyping or alignment errors in such regions. Regions where we are unable to resolve crossovers precisely are visible on the Marey maps (Figures 2, S1) as sloping lines; a precise breakpoint resolution would appear vertical. We took two steps to alleviate the lack of resolution in our data. Firstly, when calculating number of crossovers within windows for estimates of fine-scale recombination rate (Figure S4), we identified crossover regions that overlapped either end of the window and assigned a proportion of the crossover to each window according to the proportion of the region that overlapped the window. For example, if a region containing 1 crossover was 100 kb in size and overlapped a window by 10kb, we assigned 0.1 of a crossover to that window. Secondly, we have restricted ourselves to a window size of 1 Mb, more than an order of magnitude higher than average crossover resolution and two orders of magnitude higher than average SNP density, to minimise the impact of the lack of precision of our crossover locations.

*iii) Double crossovers*

Given the steps described above, we believe that our estimates of recombination rate are accurate given the limitations of our data, and that it is highly unlikely that single crossovers have been missed given the density of our SNPs. However, it is possible that we have missed double crossovers in our survey. We believe we are at considerably lower risk of this than in older surveys where single markers may have been separated by Megabases. With hundreds of SNPs genotyped per Megabase, crossover resolutions in the tens of kilobases, and overall SNP densities in the kilobases, double crossovers would also have had to occur within these narrow kilobase ranges to have been missed. Given the widespread existence of crossover interference, this seems very unlikely, although we have no direct evidence for crossover interference in *Heliconius*.

Nevertheless, we cannot completely rule out the existence of narrow double crossovers, due to the lack of resolution in our RAD data, where loci are ~10 kb apart. We manually omitted many apparent double crossovers from the maps because investigation showed them to be due to sequencing error, genotyping error due to low coverage, or alignment errors. But it is possible we excluded some genuine double crossovers. However, we do not believe it would have been possible with our data to distinguish these double crossovers from gene conversion events, and so we do not feel it would have been appropriate to include them in our maps without further support.

Therefore, it is possible our overall map lengths are lower than the true map lengths, which could be addressed by additional, more thorough sequencing; whole genome sequencing combined with technical replicates would be required to distinguish narrow double crossovers from gene conversion events. But as our overall map lengths are highly consistent between species, crosses and chromosomes regardless of SNP density (see above), we do not believe conclusions drawn from comparisons of our maps to each other are affected by this issue.

*Absence of female recombination*

It has long been reported that crossing over is absent in *Heliconius* females (Turner and Sheppard 1975) and no evidence for female crossing over has been found in several previous linkage maps of *H. melpomene* (Jiggins *et al.* 2005, Pringle *et al.* 2007, Davey *et al.* 2016). Here again we could identify unique chromosome prints for each chromosome, each supported by hundreds or thousands of SNPs spanning the length of the reference chromosome sequences (Figure S2, Table S4), and could not identify any crossovers in females that could be distinguished from sequencing error or potential gene conversion events, and so have continued to focus on paternal crossovers throughout.

There are a small number of chromosomes with large regions with no identifiable chromosome print (e.g. *H. melpomene* cross 1 (MEL1) chromosomes 3, 14 and 15); these regions are sequenced well and have many valid RAD sites, but they have several times fewer raw SNPs than regions with an identifiable print, and our filters rejected all of these raw SNPs as genotyping errors. On further inspection of the raw SNPs, we could find no evidence for alternative or recombined chromosome prints in these regions, so we believe the lack of SNPs in these regions is due to homozygosity of these chromosomes in the cross 1 mother, which is not unusual in stocks. (There are similar regions in some of the fathers, for example *H. melpomene* cross 3 chromosome 8 (Figure S2).)

Nevertheless, despite the high density of our markers, we still cannot rule out the possibility that females recombine at the ends of chromosomes, either because the sequence at the end of the chromosomes is absent or misassembled, or because we have erroneously rejected a marker supported by a small number of SNPs at the end of a linkage map. It is also possible that females recombine via narrow double crossovers like those discussed above. But until extremely fine-scale mapping and accurate assembly of chromosome ends is available to settle the matter, we continue to believe crossing over is absent in *Heliconius* females.
