# Supplementary material for: No evidence for maintenance of a sympatric Heliconius species barrier by chromosomal inversions
Source: Evol Lett. 2017 Jun 14;1(3):138–54. doi: 10.1002/evl3.12 (PMC6122123; doi:10.1002/evl3.12)
Supplement: Supplementary file 2 — Figure S1. Cross design. [file EVL3-1-138-s002.pdf]

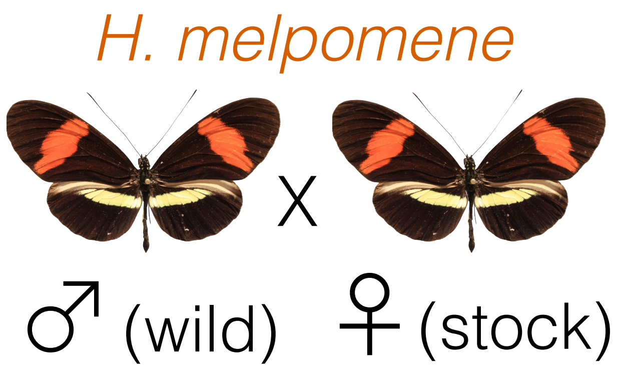

Cross	Internal name	Mother (stock)	Father (wild)	Offspring	PacBio+Trio
1	MEL1	JDMo1	JDFa1	111	No
2	MEL3	JDMo3	JDMo3	122	Yes
3	MEL4	JDMo4	JDMo4	102	No

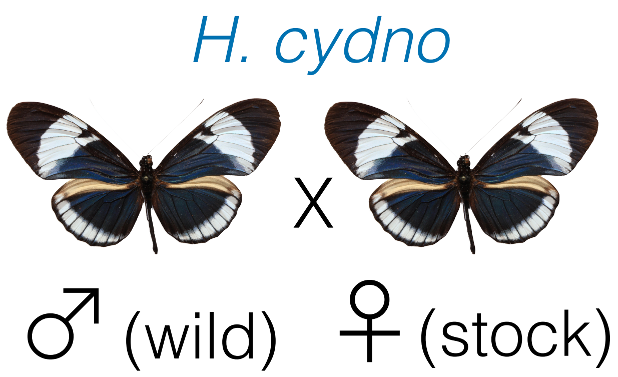

Cross	Internal name	Mother (stock)	Father (wild)	Offspring	PacBio+Trio
1	CYDA	C3 / CYDAMo	C1 / CYDAFa	95	Yes
2	CYDB	C2	C4	77	No
3	CYDE	C8	C7	125	No

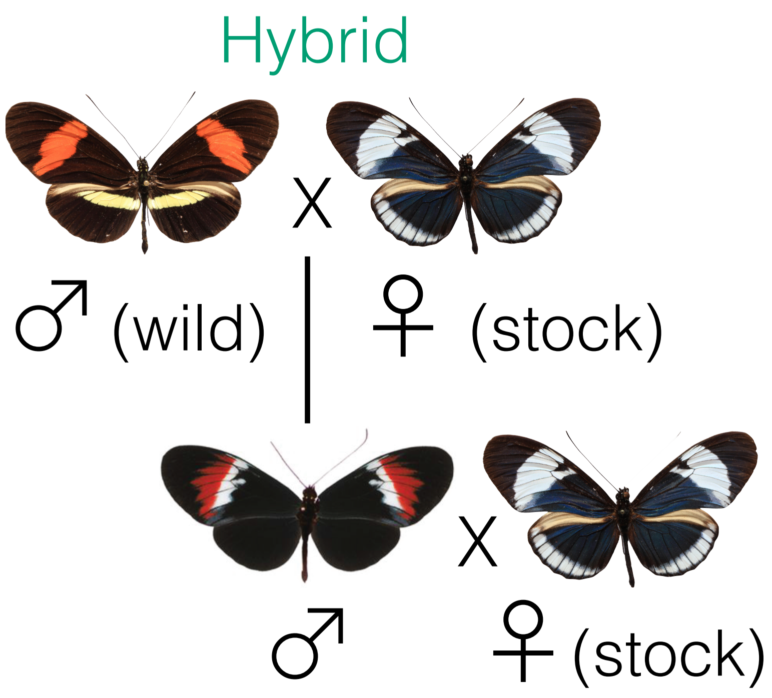

Cross	Internal name	Grandmother (stock *H. cydno*)	Grandfather (wild *H. melpomene*)	Total Backcross Offspring	Backcross name	Backcross mother (stock *H. cydno*)	F1 hybrid father	Offspring
1	C6	13040	13024	170	C8	N/A	N/A	52
					C10	13231	N/A	20
					C11	13118	13043	5
					C14	13120	N/A	6
					C18	N/A	13115	47
					C20	13256	N/A	3
					C23	13363	13255	30
					C24	13270	N/A	7
2	C26	13411	13334	88	C32	13453	N/A	34
					C36	13547	N/A	10
					C37	13635	13444	16
					C38	13541	N/A	6
					C51	13742	13550	22
3	C29	13428	13376	68	C70	13911	N/A	22
					C71	13897	N/A	13
					C72	13910	N/A	27
					C94	13935	N/A	6
4	C1	13005	13006	5	C3	13031	13020	5
