# Supplementary material for: No evidence for maintenance of a sympatric Heliconius species barrier by chromosomal inversions
Source: Evol Lett. 2017 Jun 14;1(3):138–54. doi: 10.1002/evl3.12 (PMC6122123; doi:10.1002/evl3.12)
Supplement: Supplementary file 3 — Figure S2. Genetic and physical maps for each ordered Hmel2 chromosome. [file EVL3-1-138-s003.pdf]

Chromosome 1

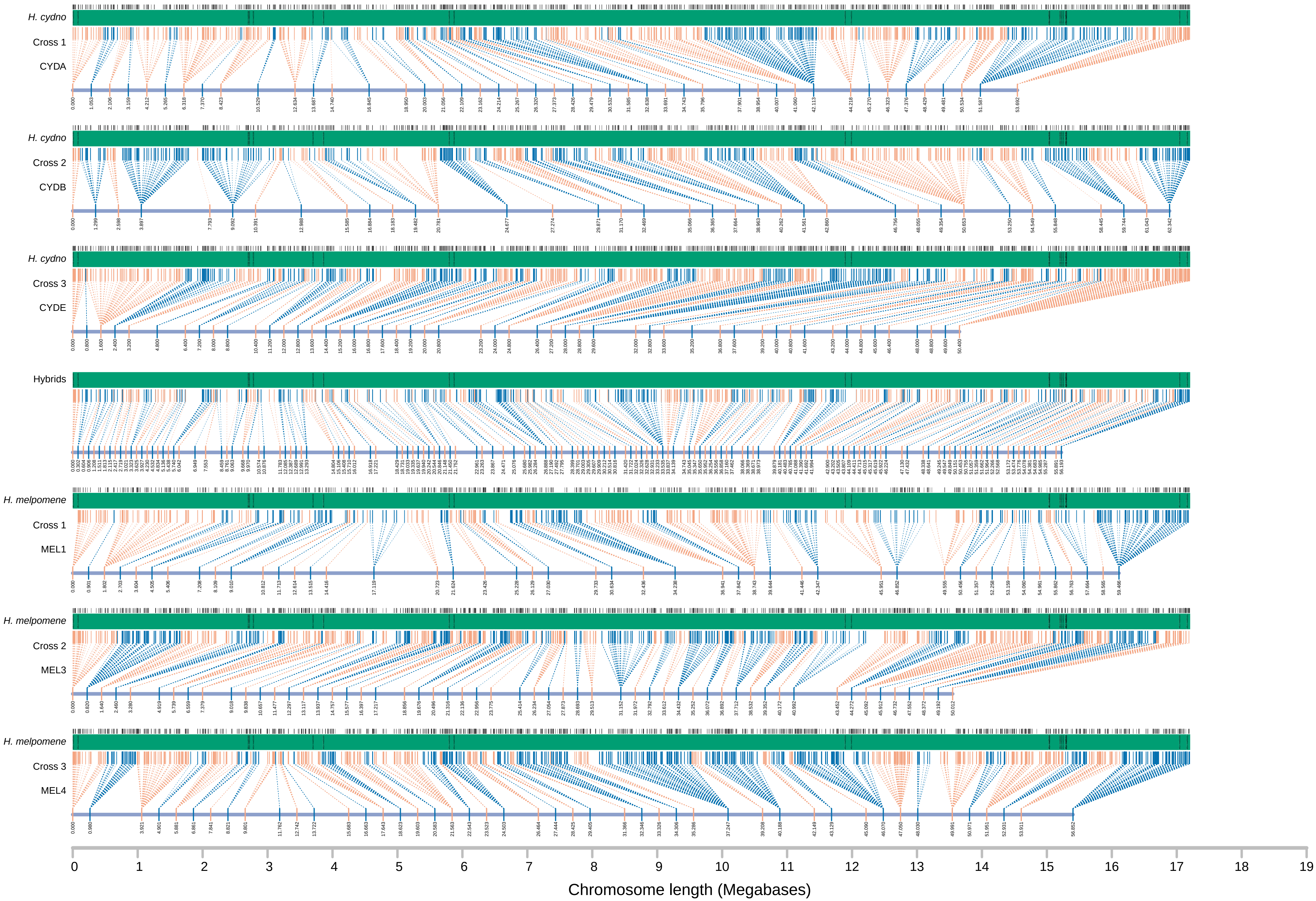

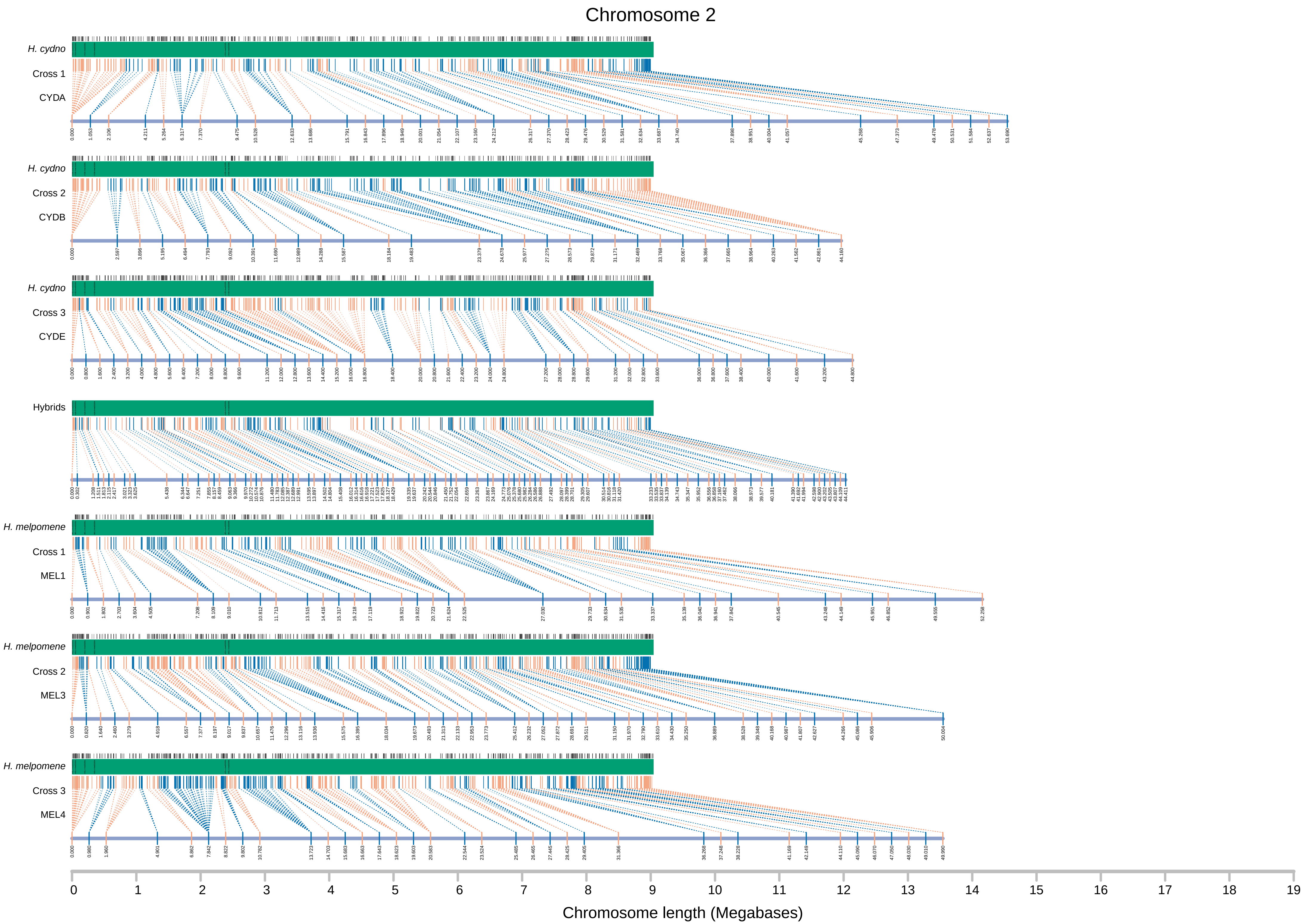
Chromosome 2
H. cydno
Cross 1
CYDA
H. cydno
Cross 2
CYDB
H. cydno
Cross 3
CYDE
Hybrids
H. melpomene
Cross 1
MEL1
H. melpomene
Cross 2
MEL3
H. melpomene
Cross 3
MEL4
0
1
2
3
4
5
6
7
8
9
10
11
12
13
14
15
16
17
18
19
Chromosome length (Megabases)

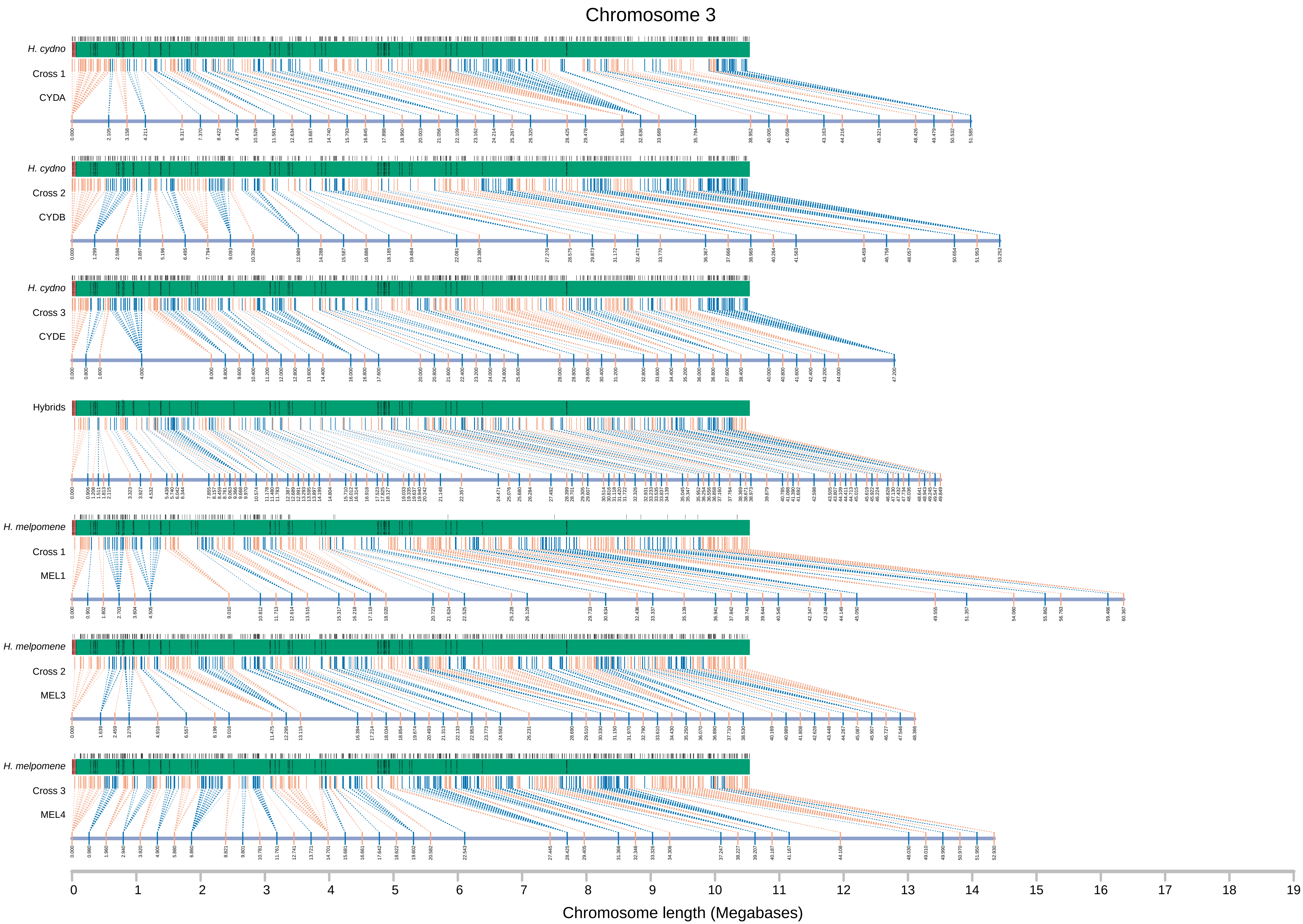

Chromosome 3
H. cydno
Cross 1
CYDA
H. cydno
Cross 2
CYDB
H. cydno
Cross 3
CYDE
Hybrids
H. melpomene
Cross 1
MEL1
H. melpomene
Cross 2
MEL3
H. melpomene
Cross 3
MEL4
0
1
2
3
4
5
6
7
8
9
10
11
12
13
14
15
16
17
18
19
Chromosome length (Megabases)

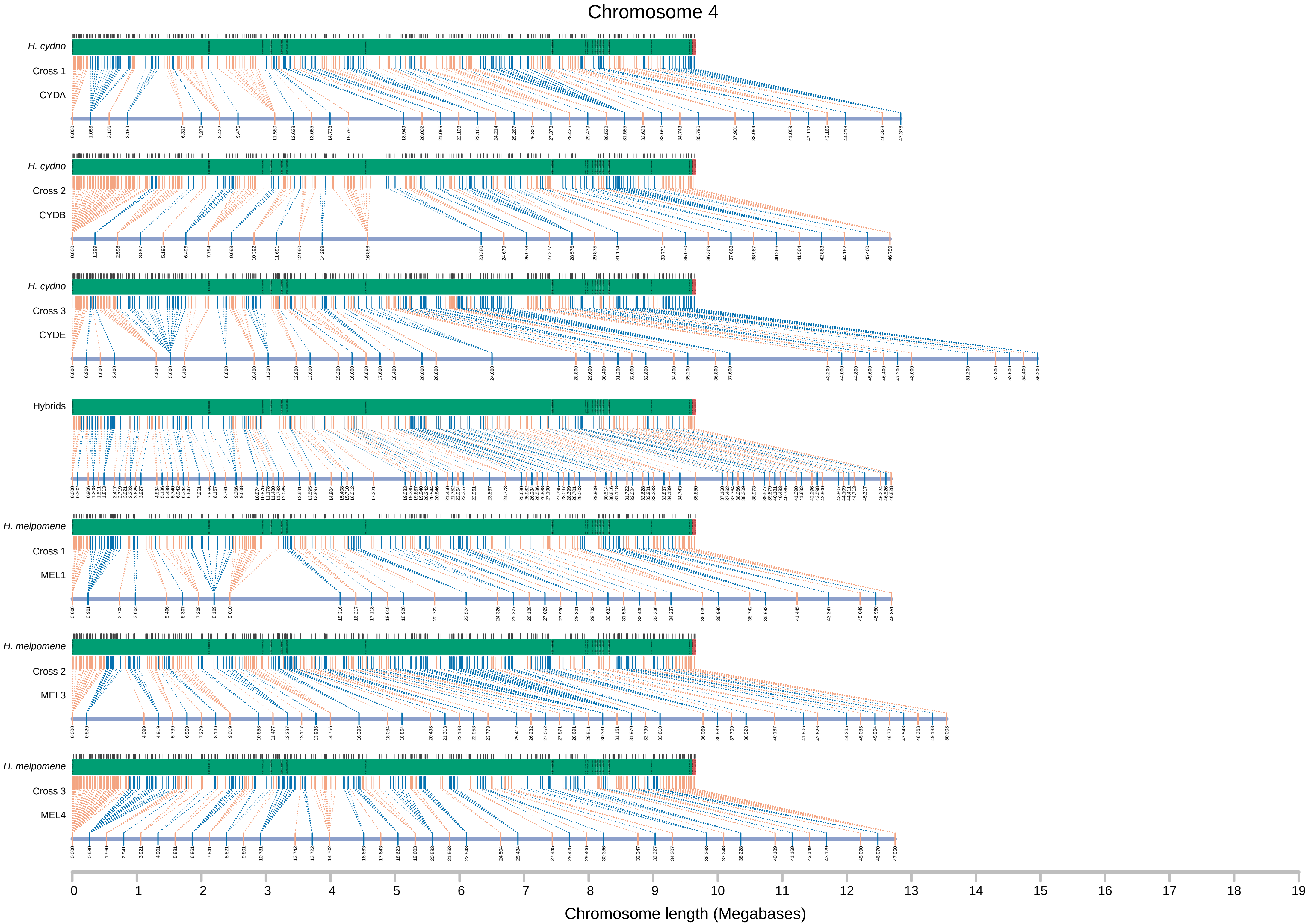
Chromosome 4
H. cydno
Cross 1
CYDA
H. cydno
Cross 2
CYDB
H. cydno
Cross 3
CYDE
Hybrids
H. melpomene
Cross 1
MEL1
H. melpomene
Cross 2
MEL3
H. melpomene
Cross 3
MEL4
0
1
2
3
4
5
6
7
8
9
10
11
12
13
14
15
16
17
18
19
Chromosome length (Megabases)

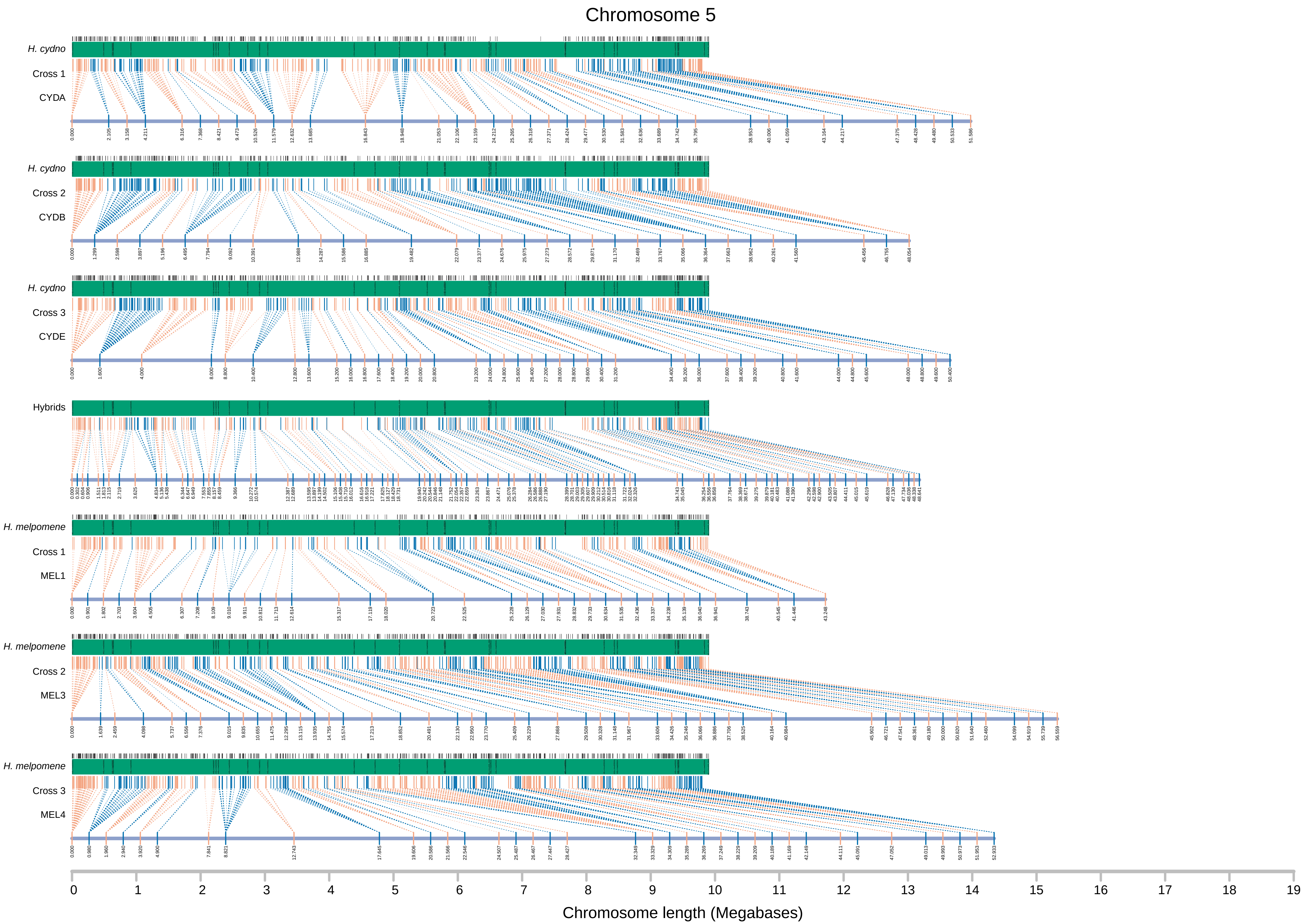
Chromosome 5
H. cydno
Cross 1
CYDA
H. cydno
Cross 2
CYDB
H. cydno
Cross 3
CYDE
Hybrids
H. melpomene
Cross 1
MEL1
H. melpomene
Cross 2
MEL3
H. melpomene
Cross 3
MEL4
0
1
2
3
4
5
6
7
8
9
10
11
12
13
14
15
16
17
18
19
Chromosome length (Megabases)

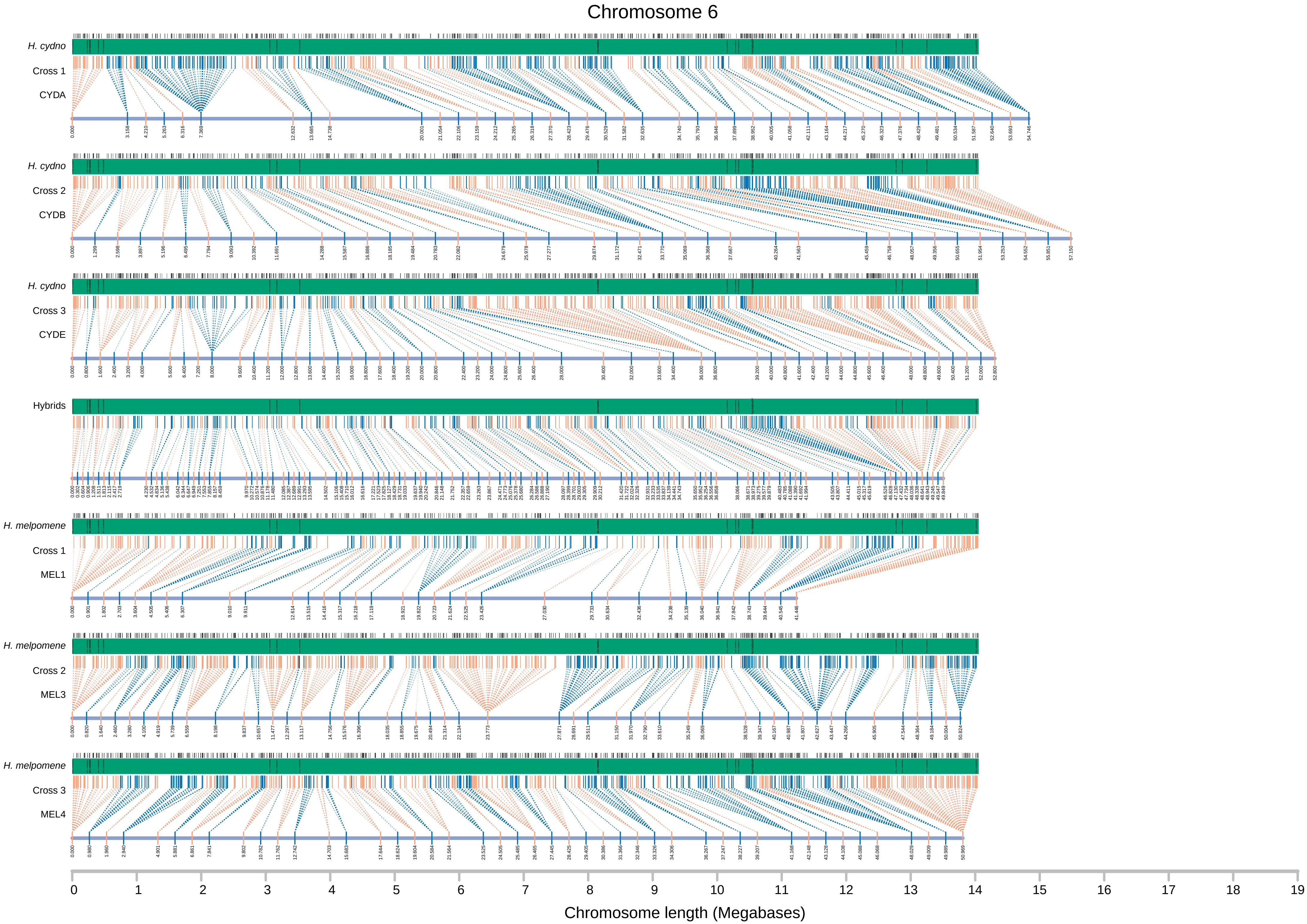

Chromosome 6
H. cydno
Cross 1
CYDA
H. cydno
Cross 2
CYDB
H. cydno
Cross 3
CYDE
Hybrids
H. melpomene
Cross 1
MEL1
H. melpomene
Cross 2
MEL3
H. melpomene
Cross 3
MEL4
0
1
2
3
4
5
6
7
8
9
10
11
12
13
14
15
16
17
18
19
Chromosome length (Megabases)

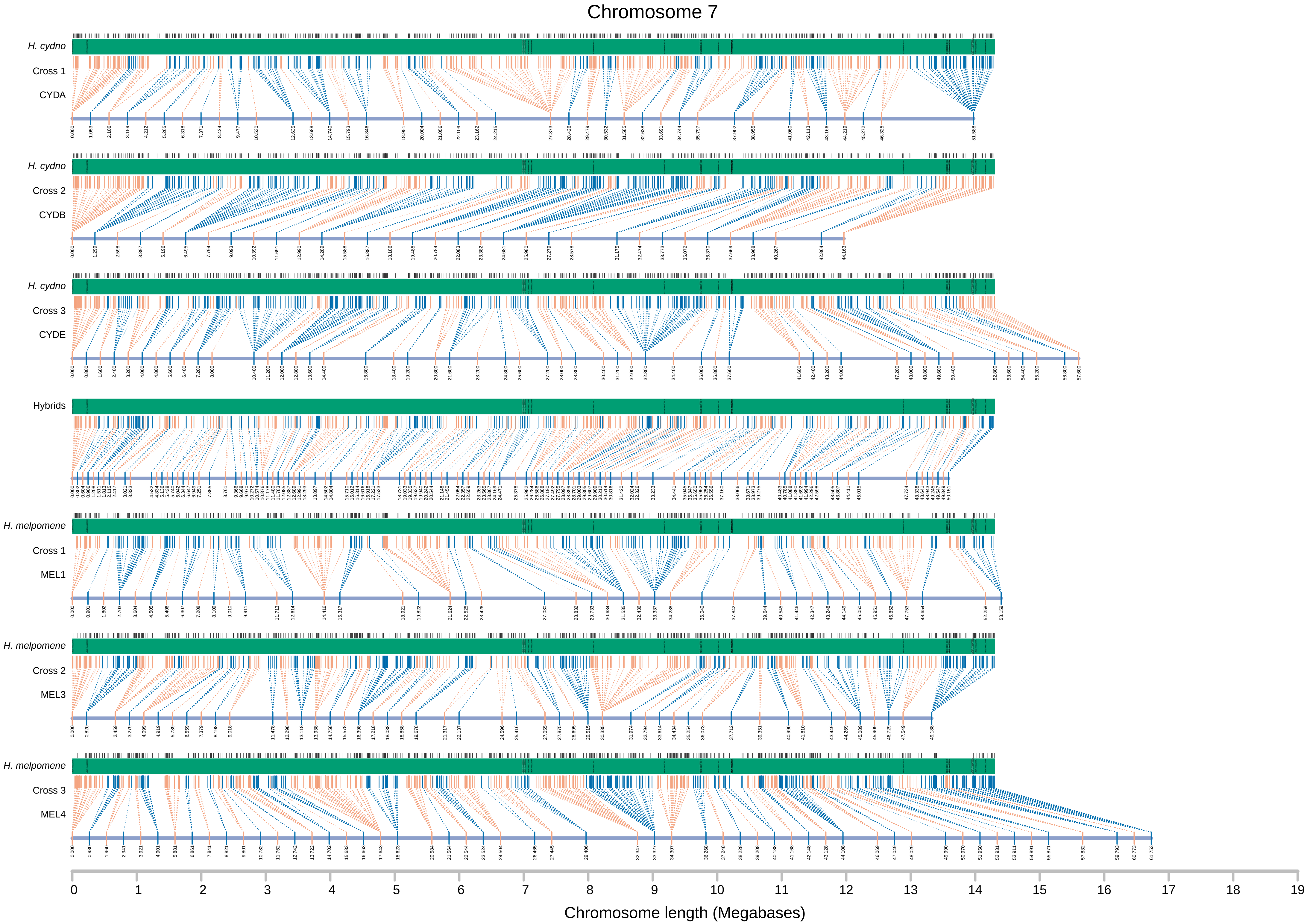

Chromosome 7
H. cydno
Cross 1
CYDA
H. cydno
Cross 2
CYDB
H. cydno
Cross 3
CYDE
Hybrids
H. melpomene
Cross 1
MEL1
H. melpomene
Cross 2
MEL3
H. melpomene
Cross 3
MEL4
0
1
2
3
4
5
6
7
8
9
10
11
12
13
14
15
16
17
18
19
Chromosome length (Megabases)

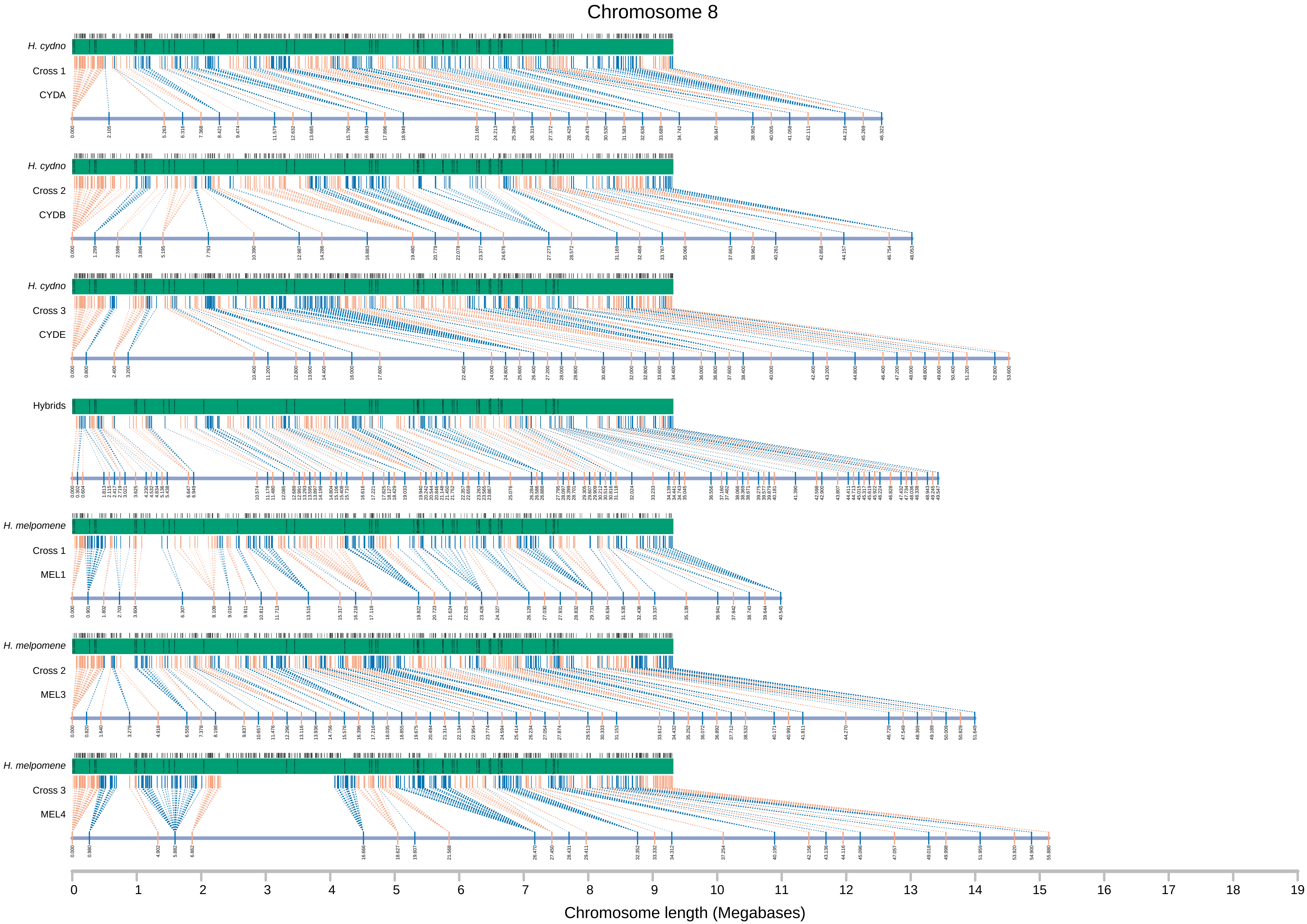
Chromosome 8
H. cydno
Cross 1
CYDA
H. cydno
Cross 2
CYDB
H. cydno
Cross 3
CYDE
Hybrids
H. melpomene
Cross 1
MEL1
H. melpomene
Cross 2
MEL3
H. melpomene
Cross 3
MEL4
0
1
2
3
4
5
6
7
8
9
10
11
12
13
14
15
16
17
18
19
Chromosome length (Megabases)

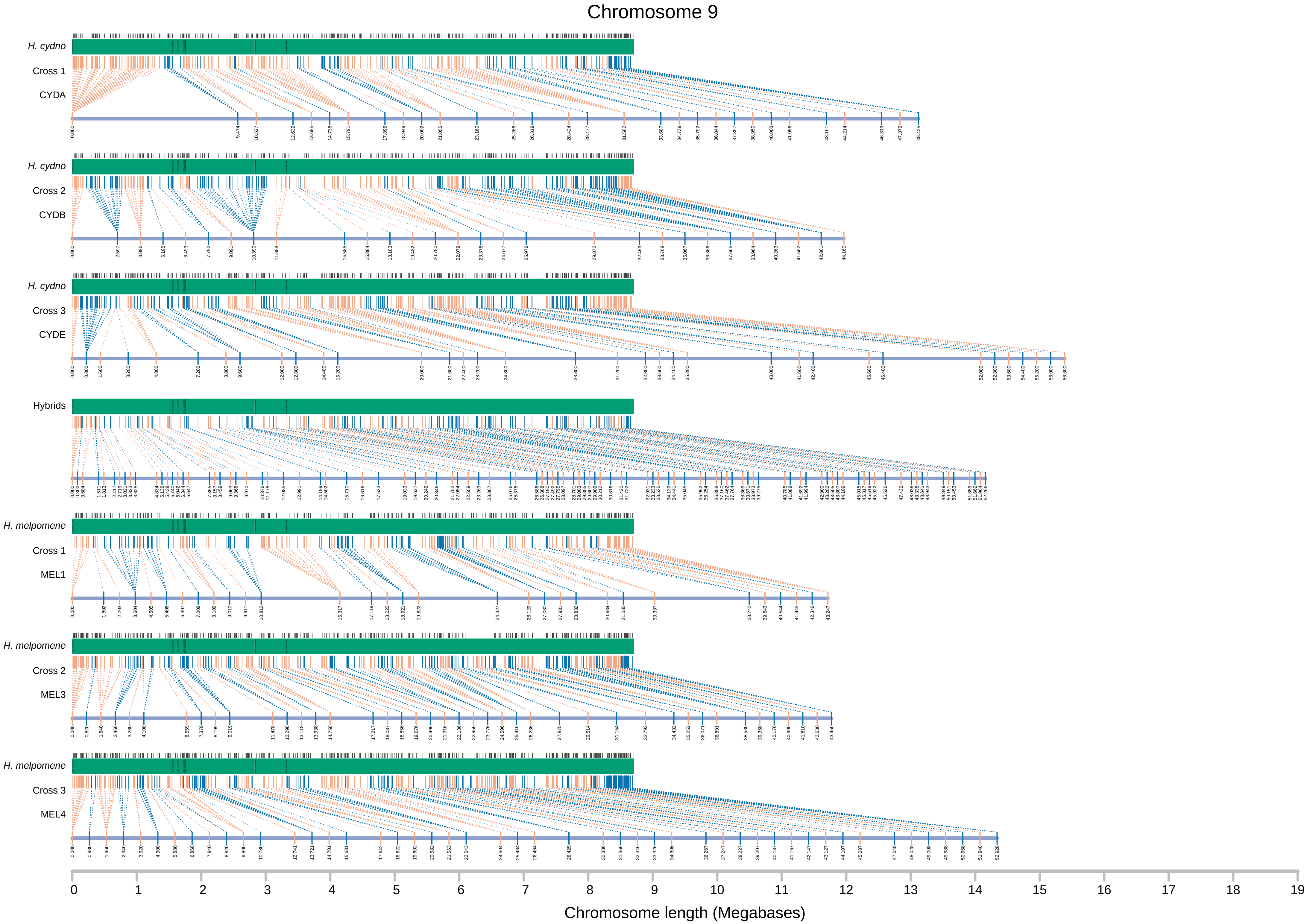

Chromosome 9
H. cydno
Cross 1
CYDA
H. cydno
Cross 2
CYDB
H. cydno
Cross 3
CYDE
Hybrids
H. melpomene
Cross 1
MEL1
H. melpomene
Cross 2
MEL3
H. melpomene
Cross 3
MEL4
0
1
2
3
4
5
6
7
8
9
10
11
12
13
14
15
16
17
18
19
Chromosome length (Megabases)

Chromosome 10

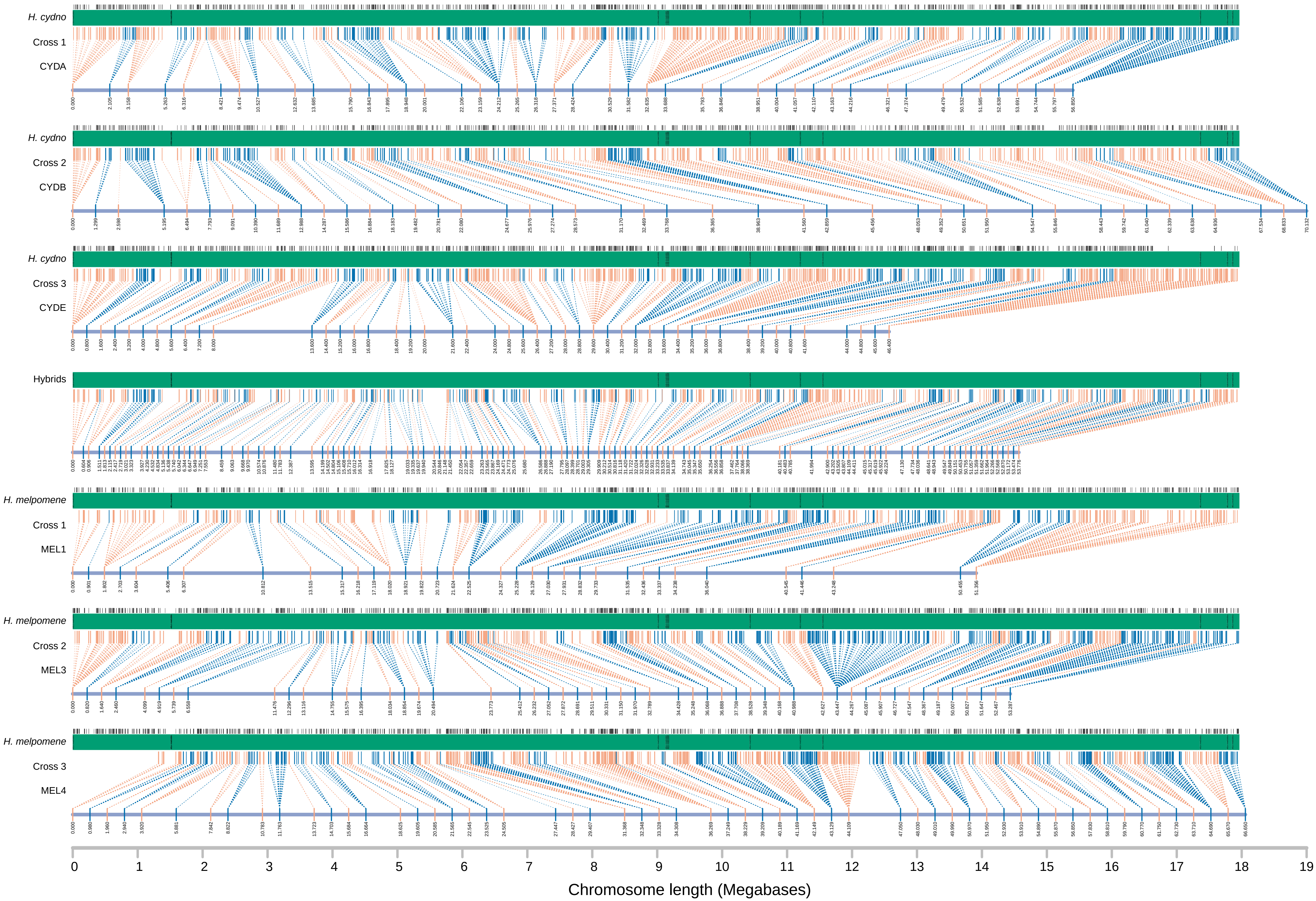

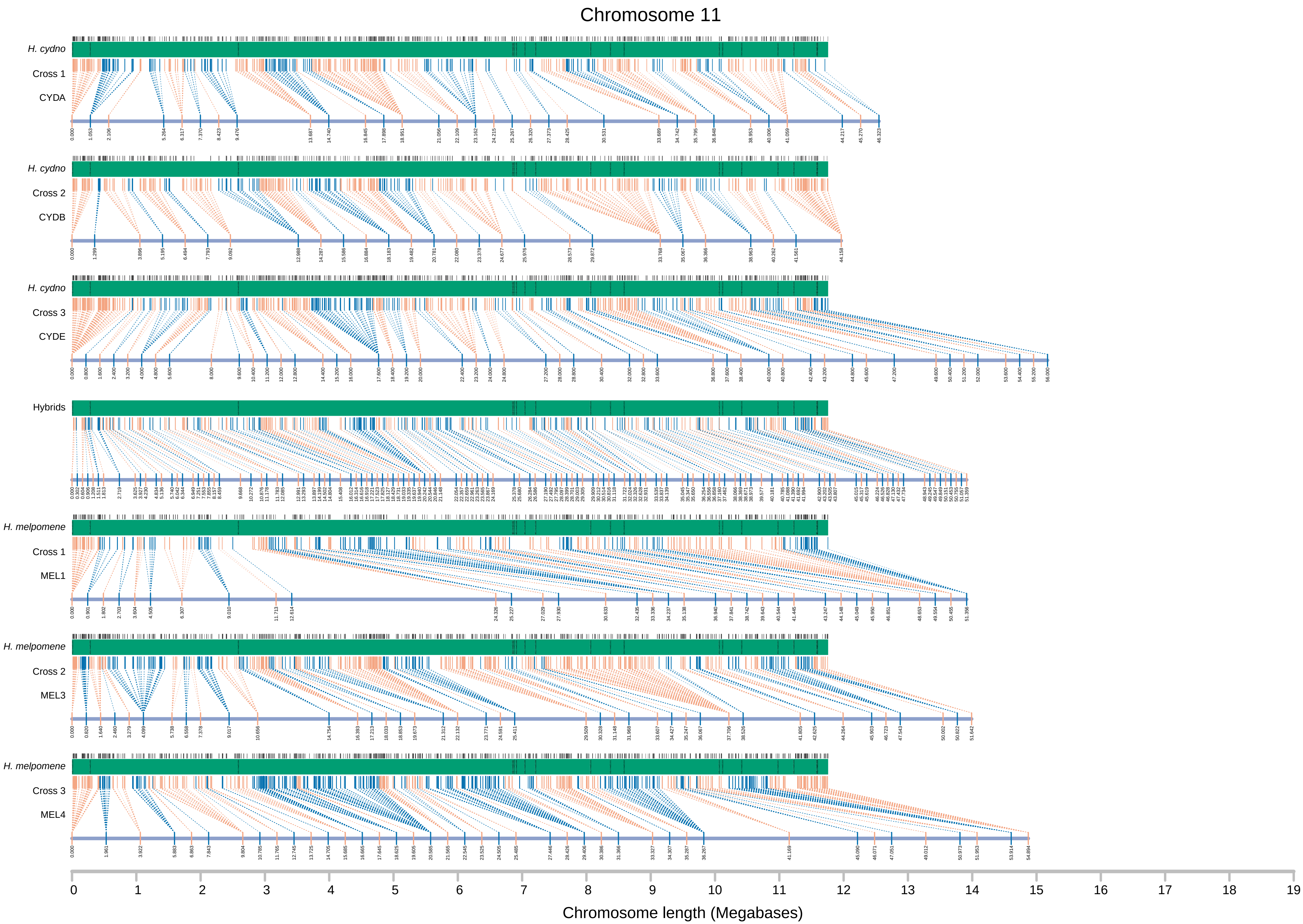
Chromosome 11
H. cydno
Cross 1
CYDA
H. cydno
Cross 2
CYDB
H. cydno
Cross 3
CYDE
Hybrids
H. melpomene
Cross 1
MEL1
H. melpomene
Cross 2
MEL3
H. melpomene
Cross 3
MEL4
0
1
2
3
4
5
6
7
8
9
10
11
12
13
14
15
16
17
18
19
Chromosome length (Megabases)

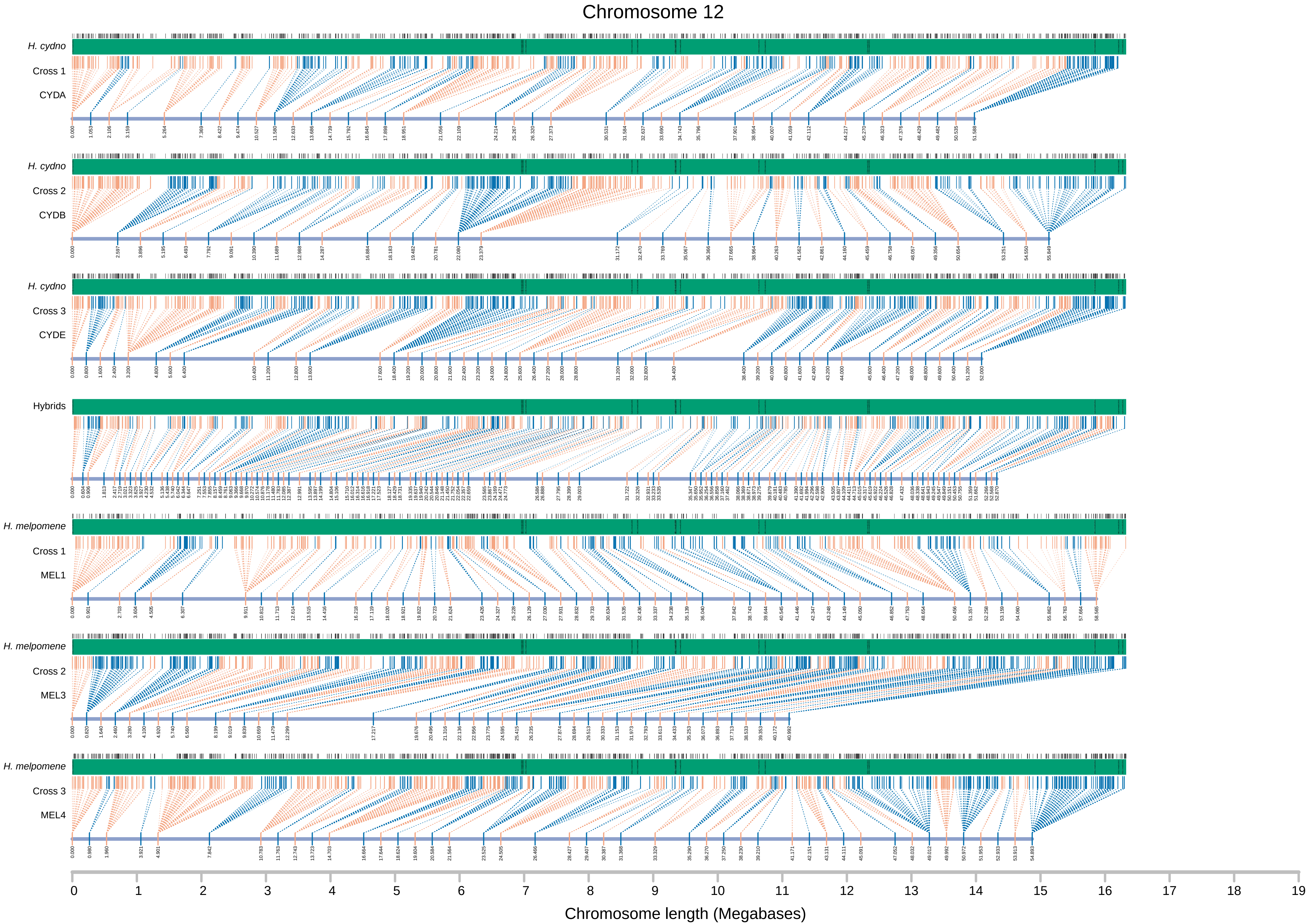
Chromosome 12
H. cydno
Cross 1
CYDA
H. cydno
Cross 2
CYDB
H. cydno
Cross 3
CYDE
Hybrids
H. melpomene
Cross 1
MEL1
H. melpomene
Cross 2
MEL3
H. melpomene
Cross 3
MEL4
0
1
2
3
4
5
6
7
8
9
10
11
12
13
14
15
16
17
18
19
Chromosome length (Megabases)

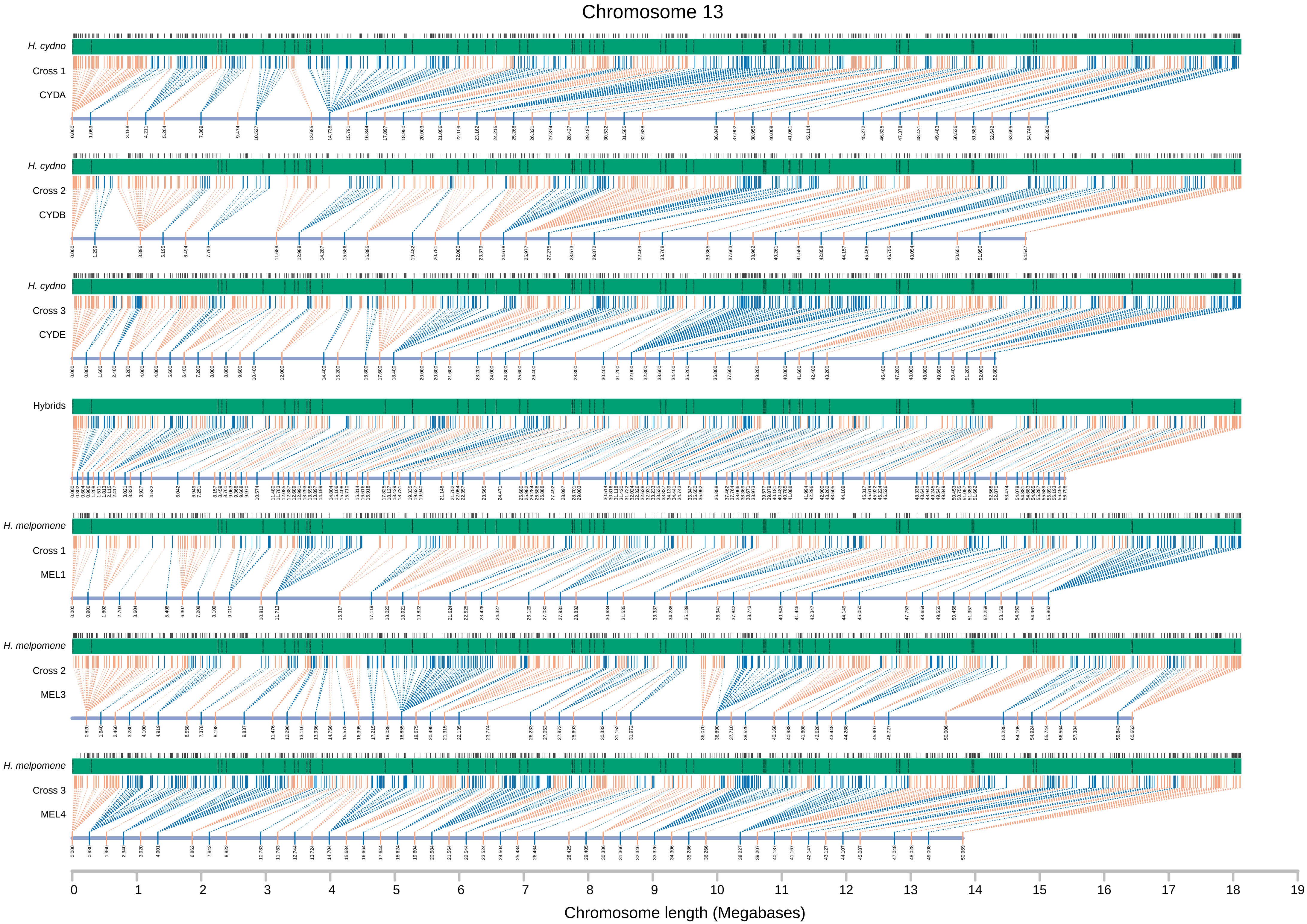
Chromosome 13
H. cydno
Cross 1
CYDA
H. cydno
Cross 2
CYDB
H. cydno
Cross 3
CYDE
Hybrids
H. melpomene
Cross 1
MEL1
H. melpomene
Cross 2
MEL3
H. melpomene
Cross 3
MEL4
0
1
2
3
4
5
6
7
8
9
10
11
12
13
14
15
16
17
18
19
Chromosome length (Megabases)

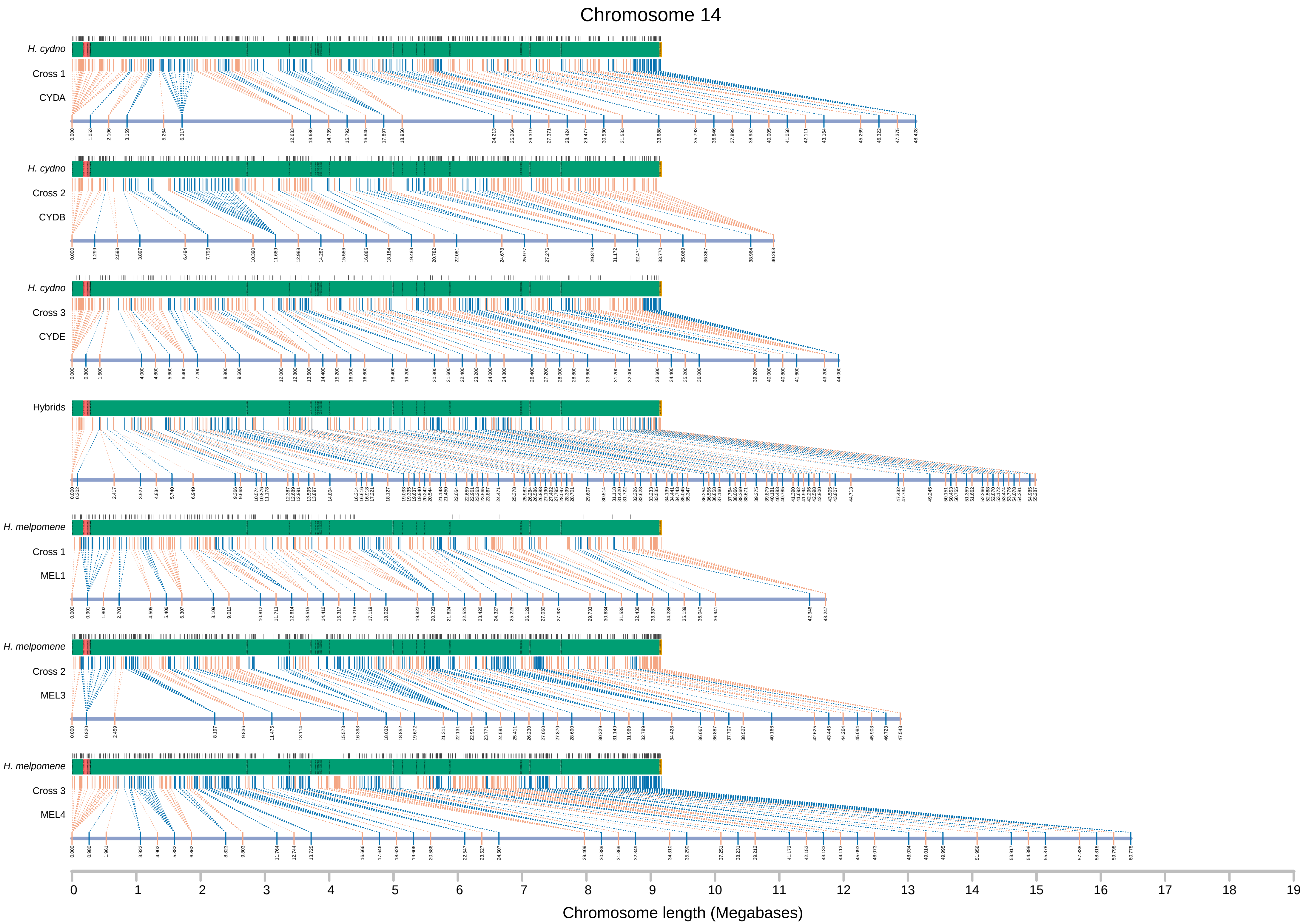

Chromosome 14
H. cydno
Cross 1
CYDA
H. cydno
Cross 2
CYDB
H. cydno
Cross 3
CYDE
Hybrids
H. melpomene
Cross 1
MEL1
H. melpomene
Cross 2
MEL3
H. melpomene
Cross 3
MEL4
0
1
2
3
4
5
6
7
8
9
10
11
12
13
14
15
16
17
18
19
Chromosome length (Megabases)

Chromosome 15

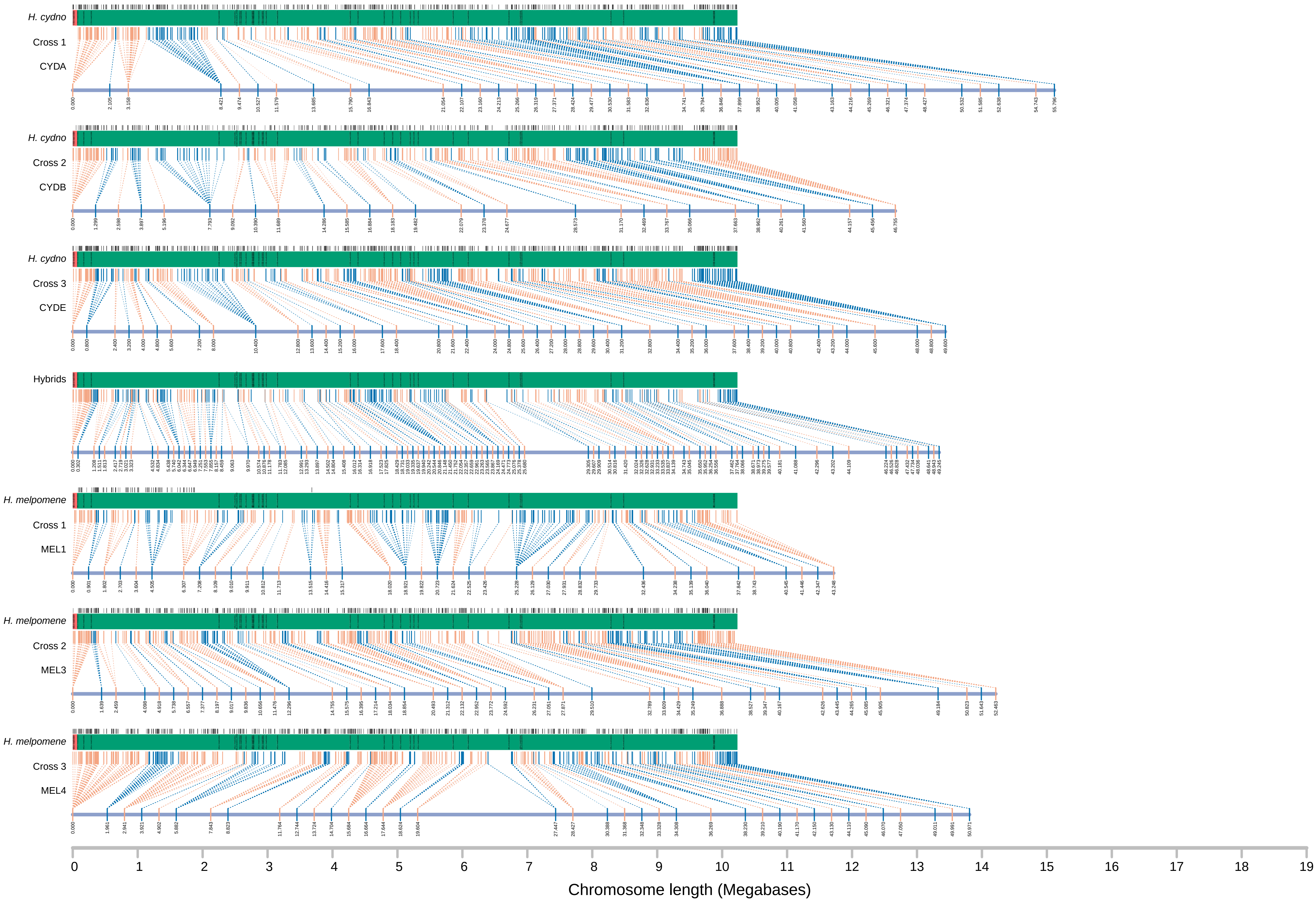

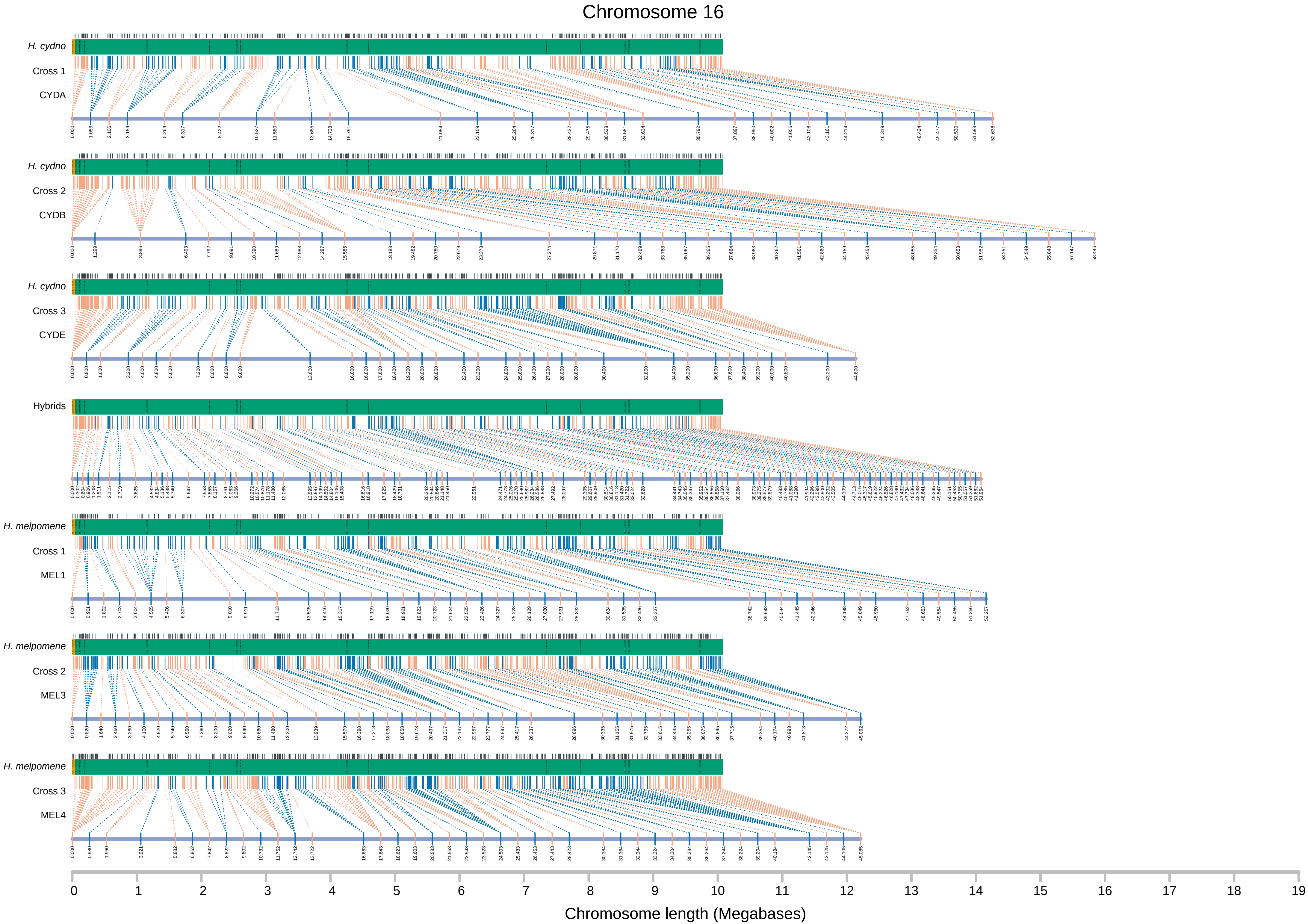
Chromosome 16
H. cydno
Cross 1
CYDA
H. cydno
Cross 2
CYDB
H. cydno
Cross 3
CYDE
Hybrids
H. melpomene
Cross 1
MEL1
H. melpomene
Cross 2
MEL3
H. melpomene
Cross 3
MEL4
0
1
2
3
4
5
6
7
8
9
10
11
12
13
14
15
16
17
18
19
Chromosome length (Megabases)

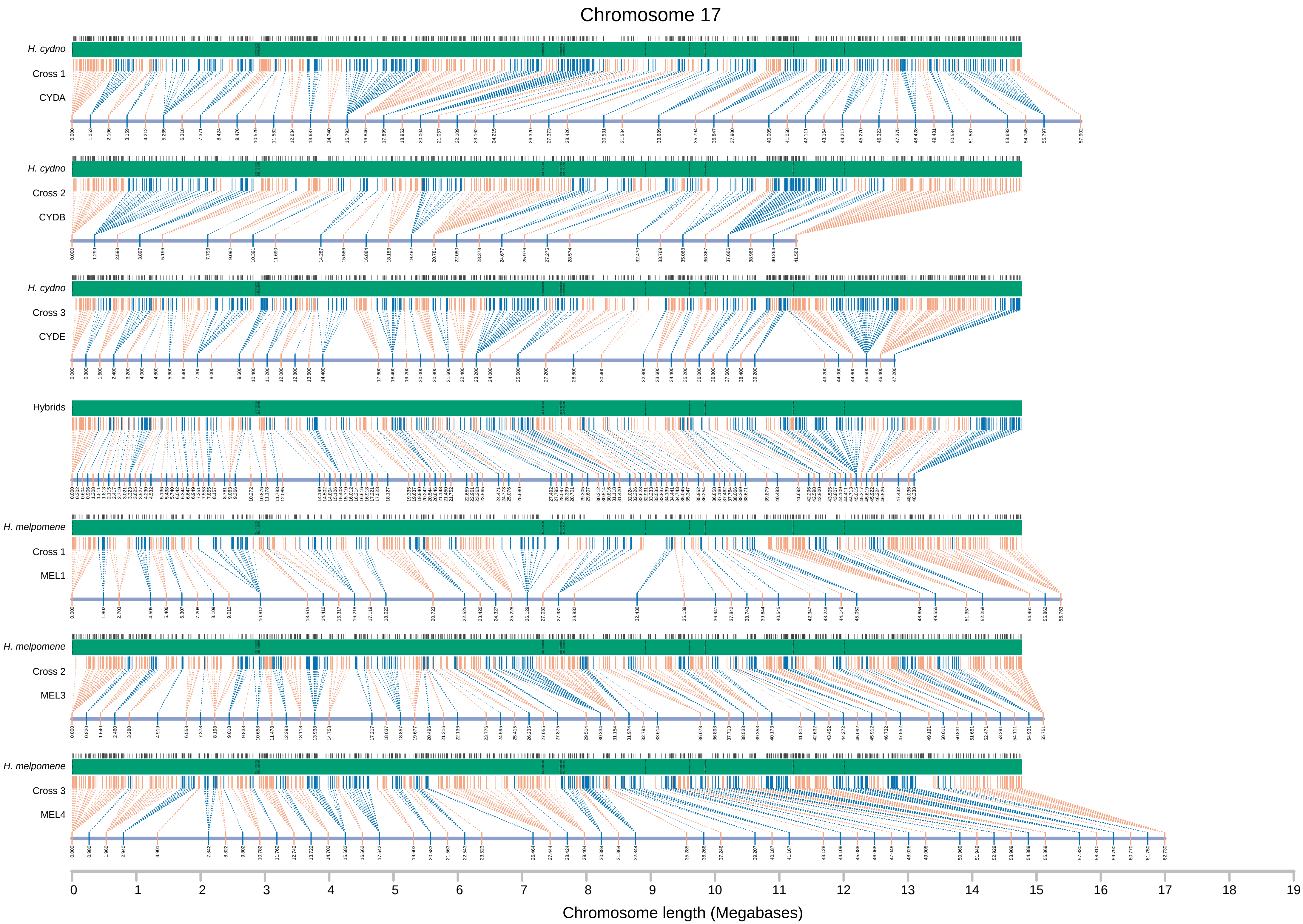
Chromosome 17
H. cydno
Cross 1
CYDA
H. cydno
Cross 2
CYDB
H. cydno
Cross 3
CYDE
Hybrids
H. melpomene
Cross 1
MEL1
H. melpomene
Cross 2
MEL3
H. melpomene
Cross 3
MEL4
0 1 2 3 4 5 6 7 8 9 10 11 12 13 14 15 16 17 18 19
Chromosome length (Megabases)

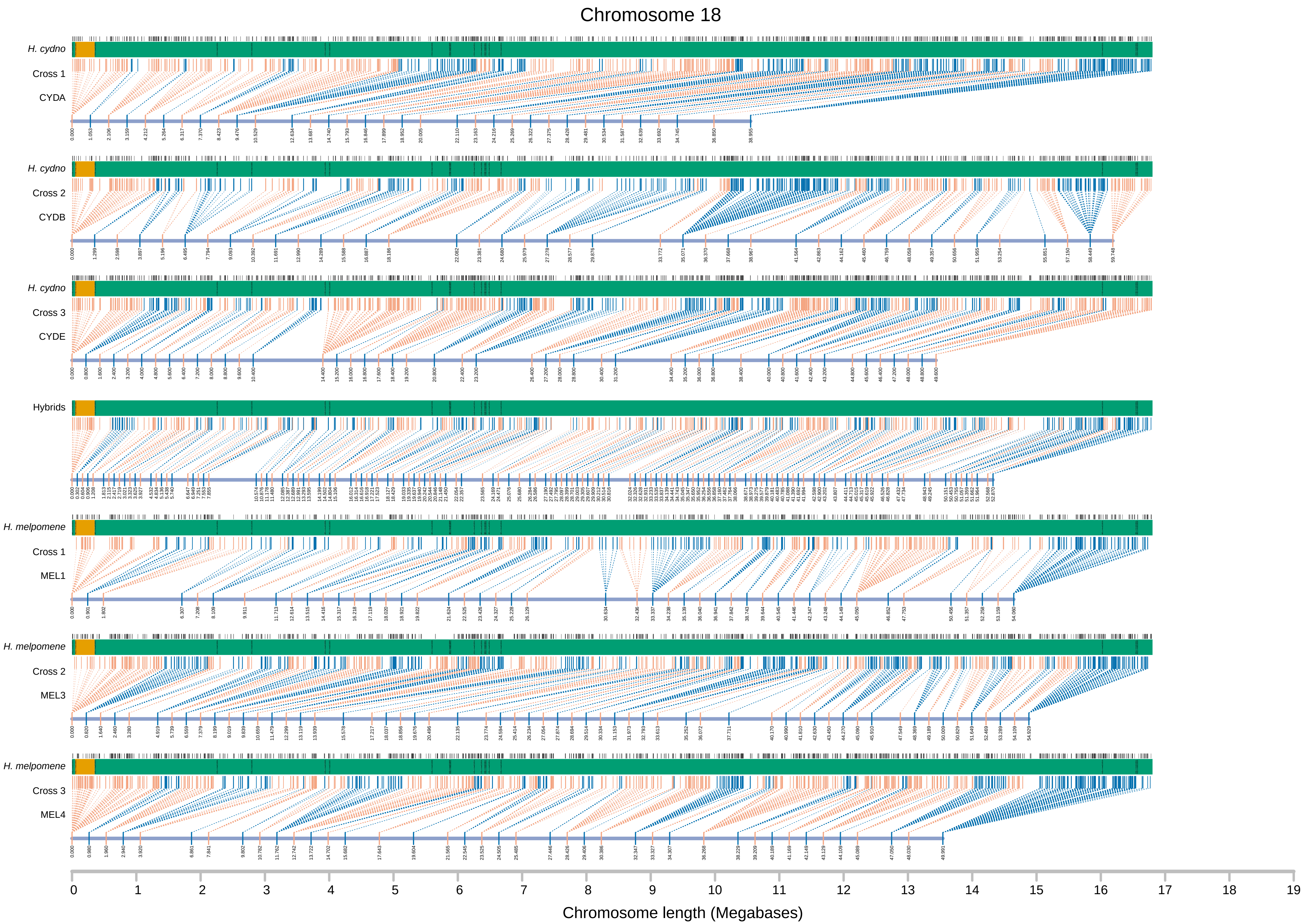
Chromosome 18
H. cydno
Cross 1
CYDA
H. cydno
Cross 2
CYDB
H. cydno
Cross 3
CYDE
Hybrids
H. melpomene
Cross 1
MEL1
H. melpomene
Cross 2
MEL3
H. melpomene
Cross 3
MEL4
0
1
2
3
4
5
6
7
8
9
10
11
12
13
14
15
16
17
18
19
Chromosome length (Megabases)

Chromosome 19

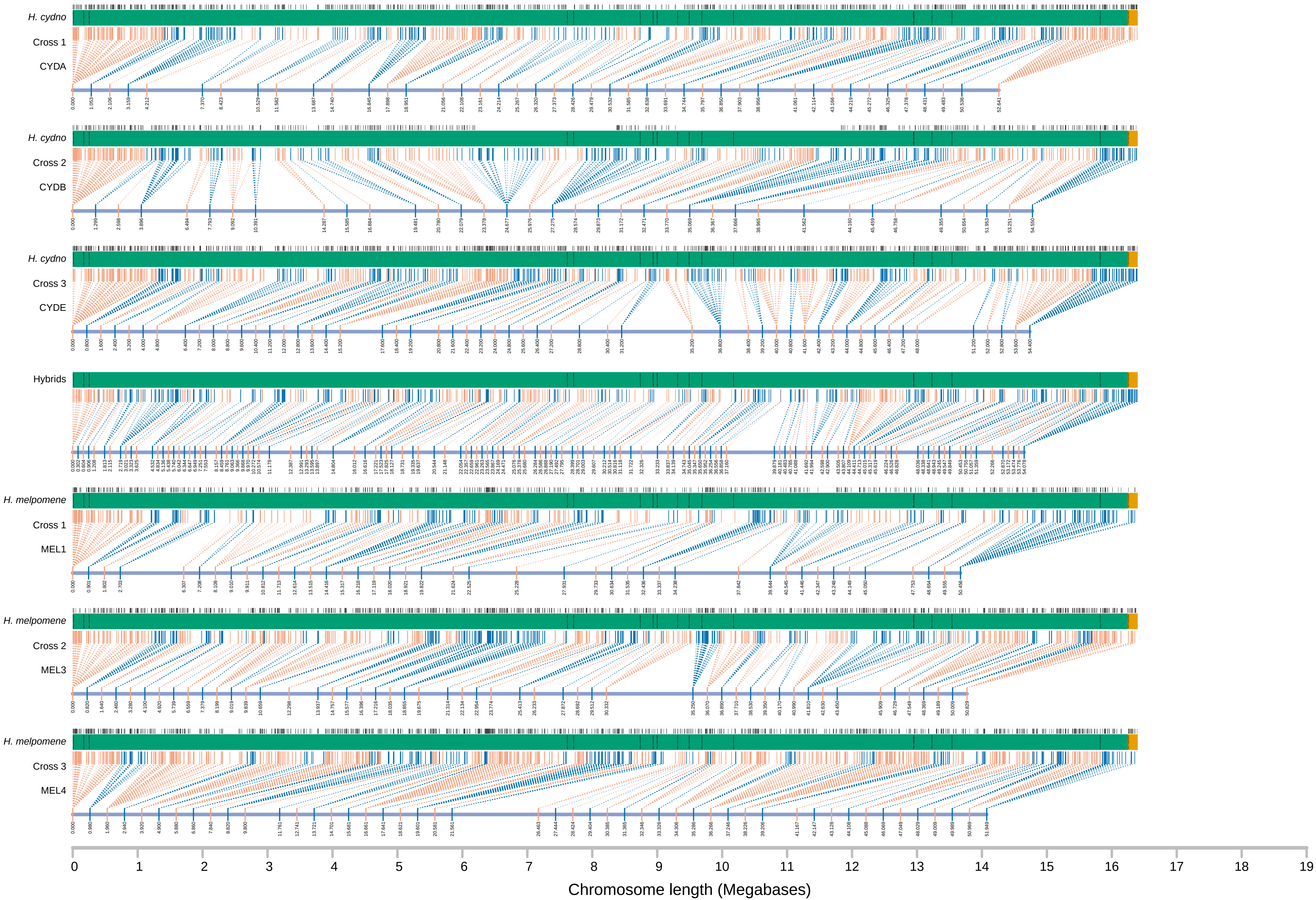

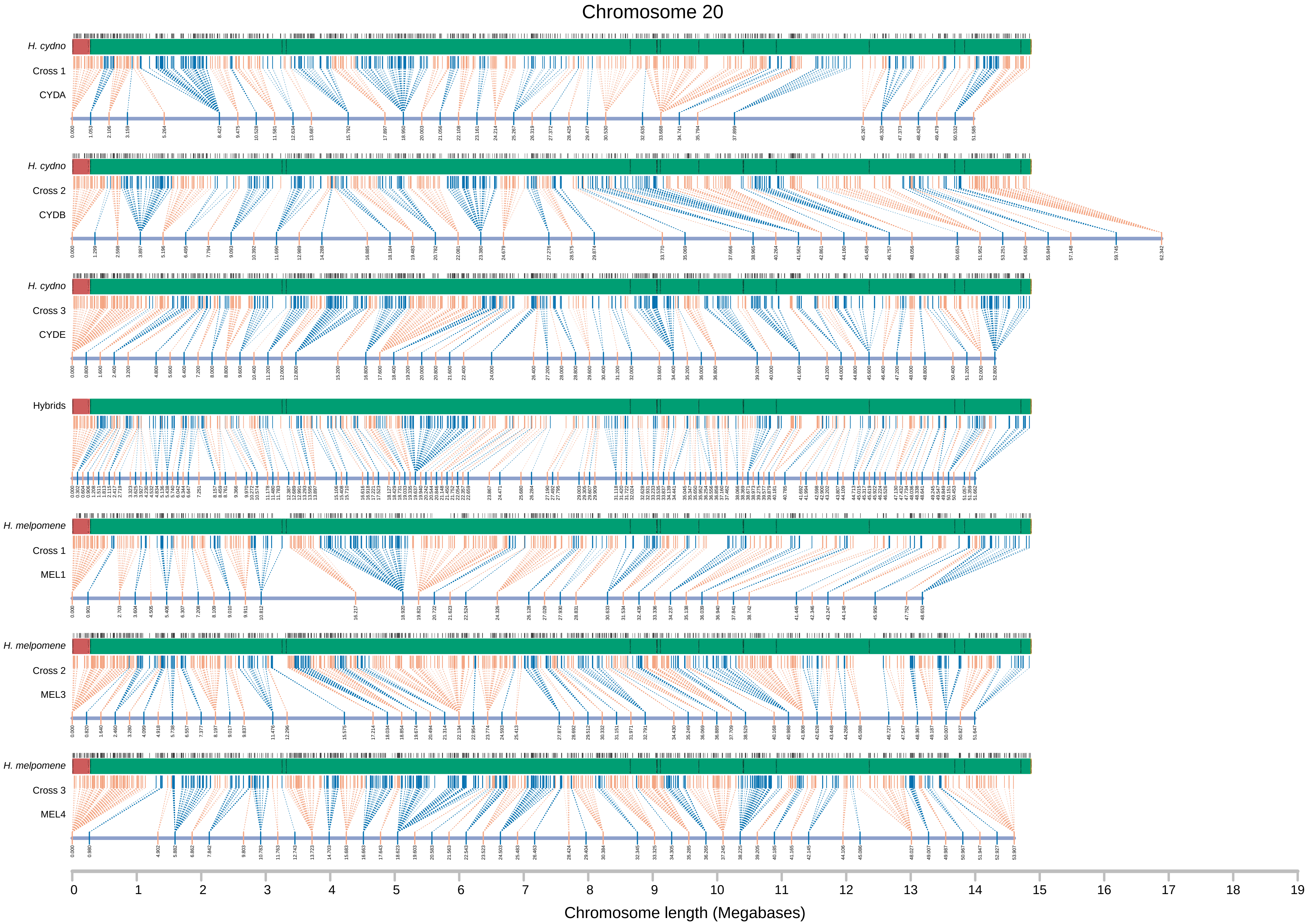
Chromosome 20
H. cydno
Cross 1
CYDA
H. cydno
Cross 2
CYDB
H. cydno
Cross 3
CYDE
Hybrids
H. melpomene
Cross 1
MEL1
H. melpomene
Cross 2
MEL3
H. melpomene
Cross 3
MEL4
0
1
2
3
4
5
6
7
8
9
10
11
12
13
14
15
16
17
18
19
Chromosome length (Megabases)

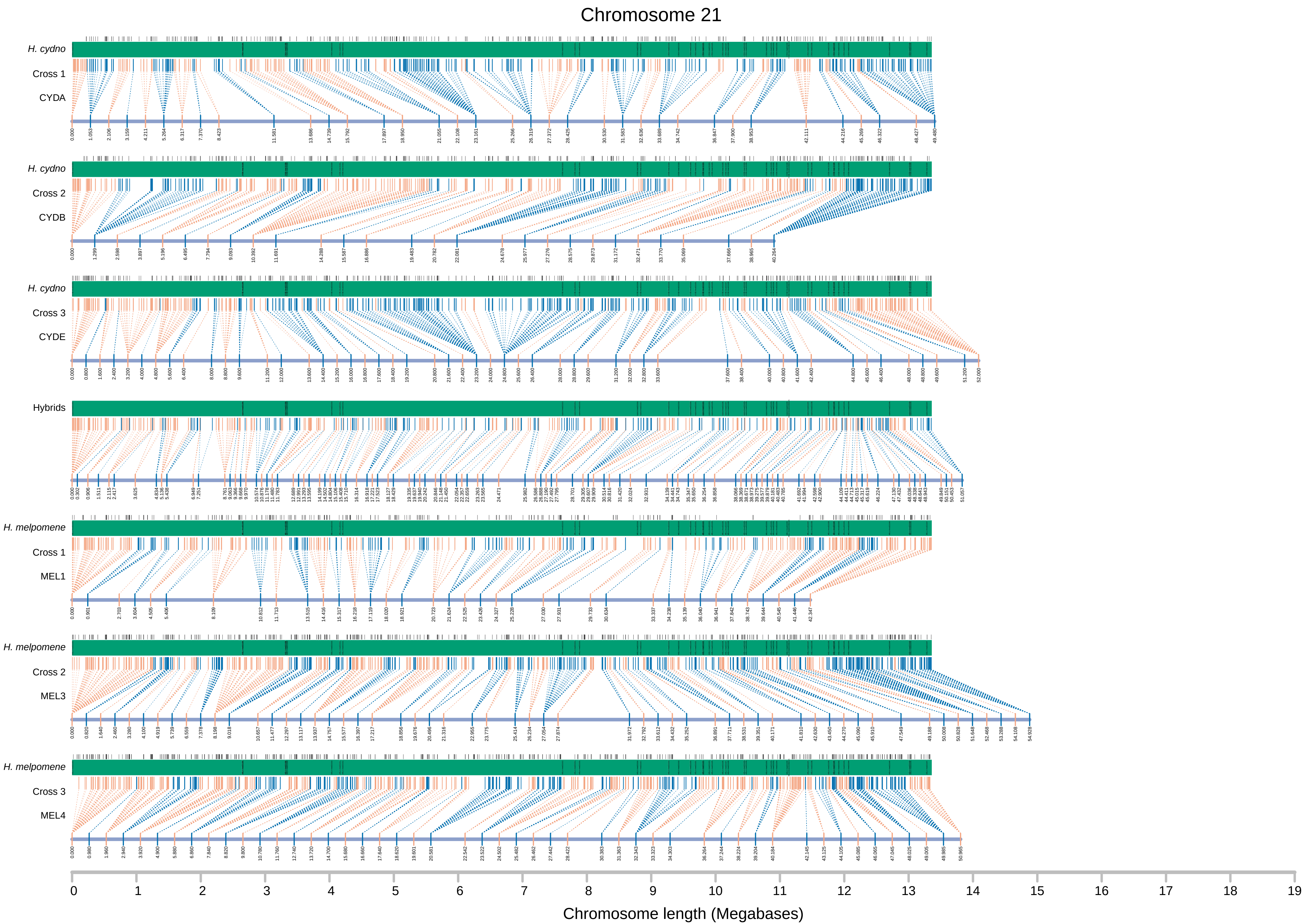
Chromosome 21
H. cydno
Cross 1
CYDA
H. cydno
Cross 2
CYDB
H. cydno
Cross 3
CYDE
Hybrids
H. melpomene
Cross 1
MEL1
H. melpomene
Cross 2
MEL3
H. melpomene
Cross 3
MEL4
0 1 2 3 4 5 6 7 8 9 10 11 12 13 14 15 16 17 18 19
Chromosome length (Megabases)
