# Supplementary material for: No evidence for maintenance of a sympatric Heliconius species barrier by chromosomal inversions
Source: Evol Lett. 2017 Jun 14;1(3):138–54. doi: 10.1002/evl3.12 (PMC6122123; doi:10.1002/evl3.12)
Supplement: Supplementary file 4 — Figure S3. SNP density, PstI site density, and GC content for each ordered Hmel2 chromosome. [file EVL3-1-138-s004.pdf]

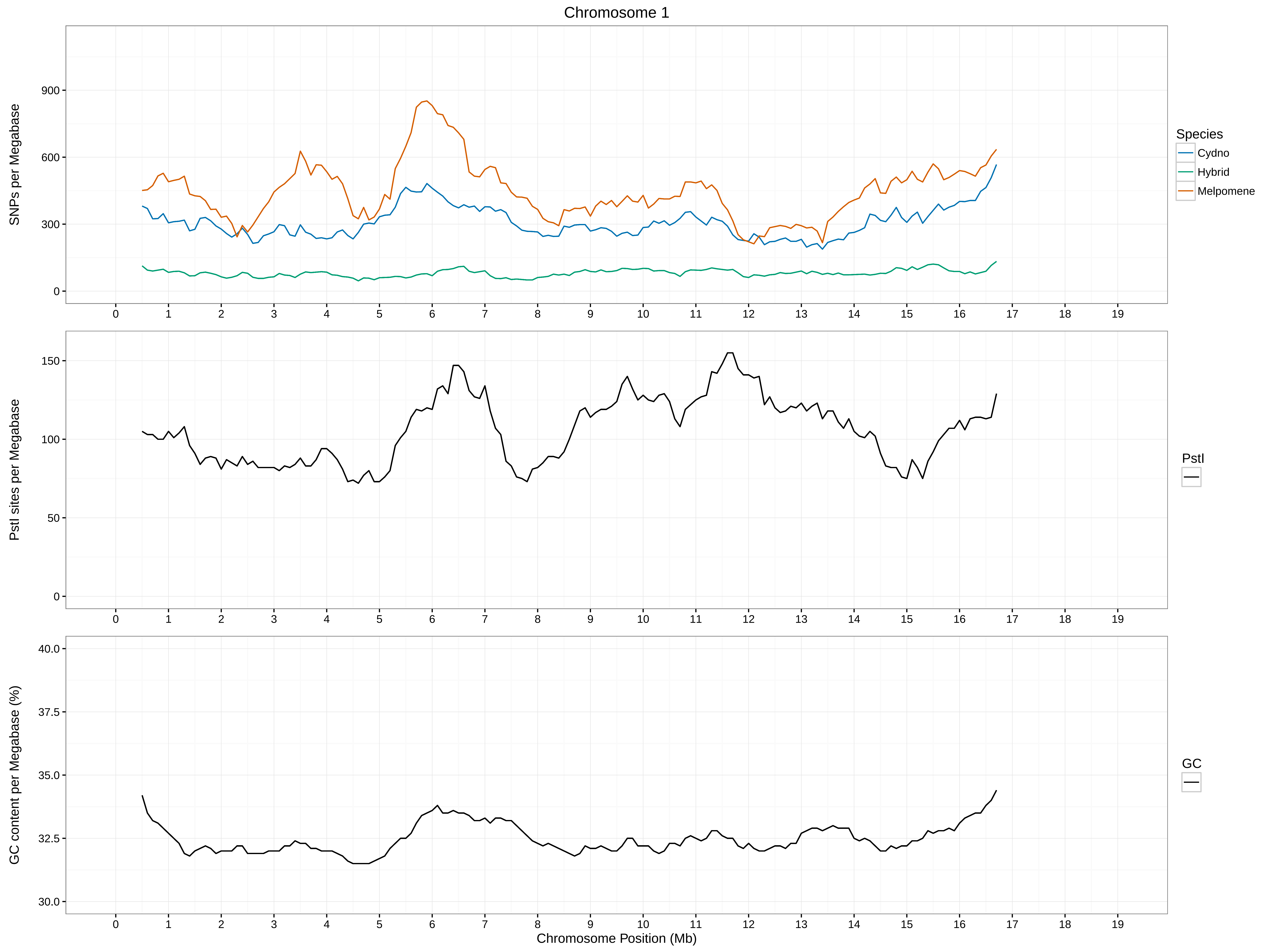
Chromosome 1
SNPs per Megabase
900
600
300
0
Species
Cydno
Hybrid
Melpomene
PstI sites per Megabase
150
100
50
0
PstI
GC content per Megabase (%)
40.0
37.5
35.0
32.5
30.0
GC
0 1 2 3 4 5 6 7 8 9 10 11 12 13 14 15 16 17 18 19
Chromosome Position (Mb)

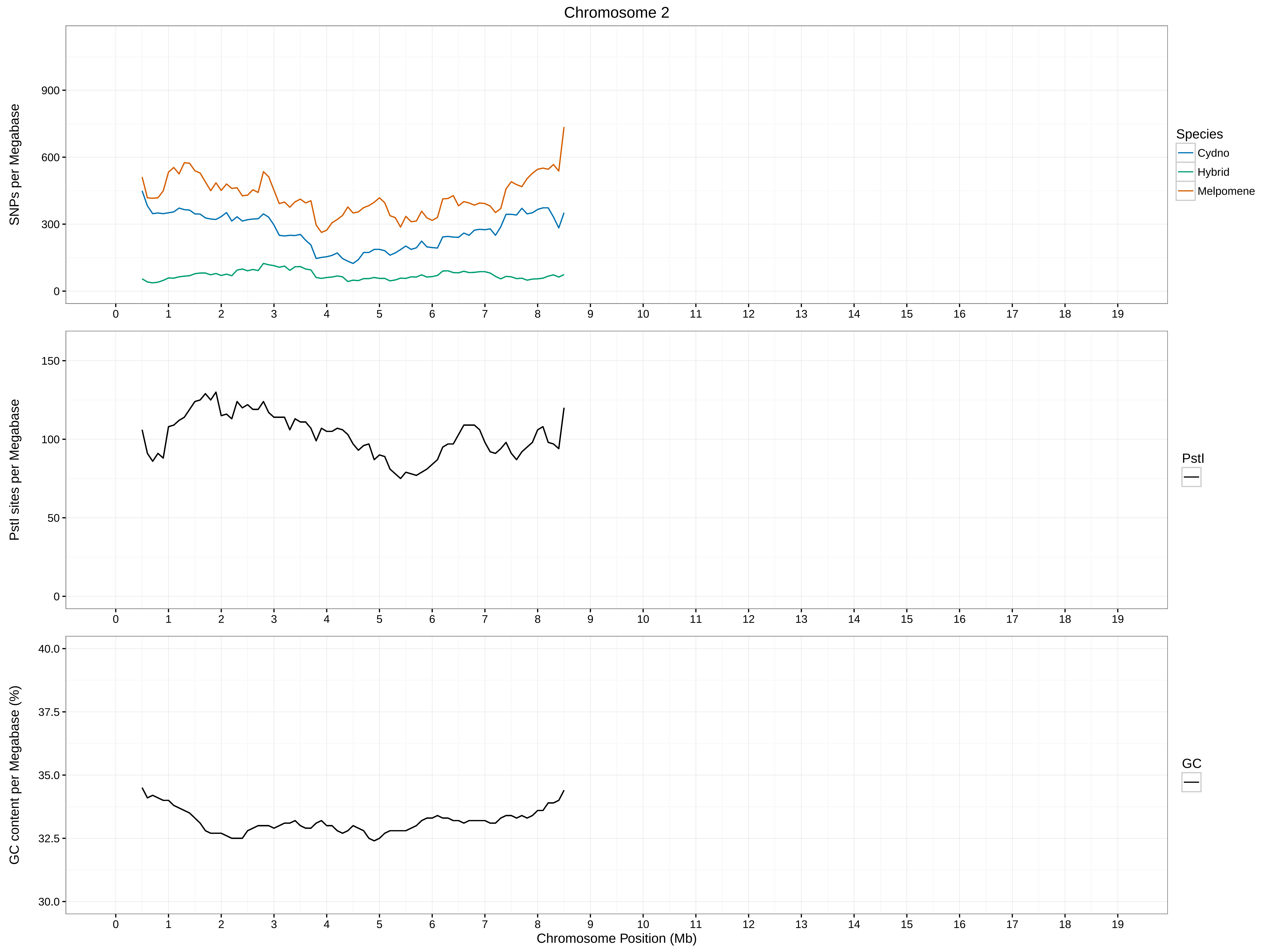

Chromosome 2
SNPs per Megabase
900
600
300
0
Species
Cydno
Hybrid
Melpomene
0 1 2 3 4 5 6 7 8 9 10 11 12 13 14 15 16 17 18 19
PstI sites per Megabase
150
100
50
0
PstI
GC content per Megabase (%)
40.0
37.5
35.0
32.5
30.0
GC
Chromosome Position (Mb)

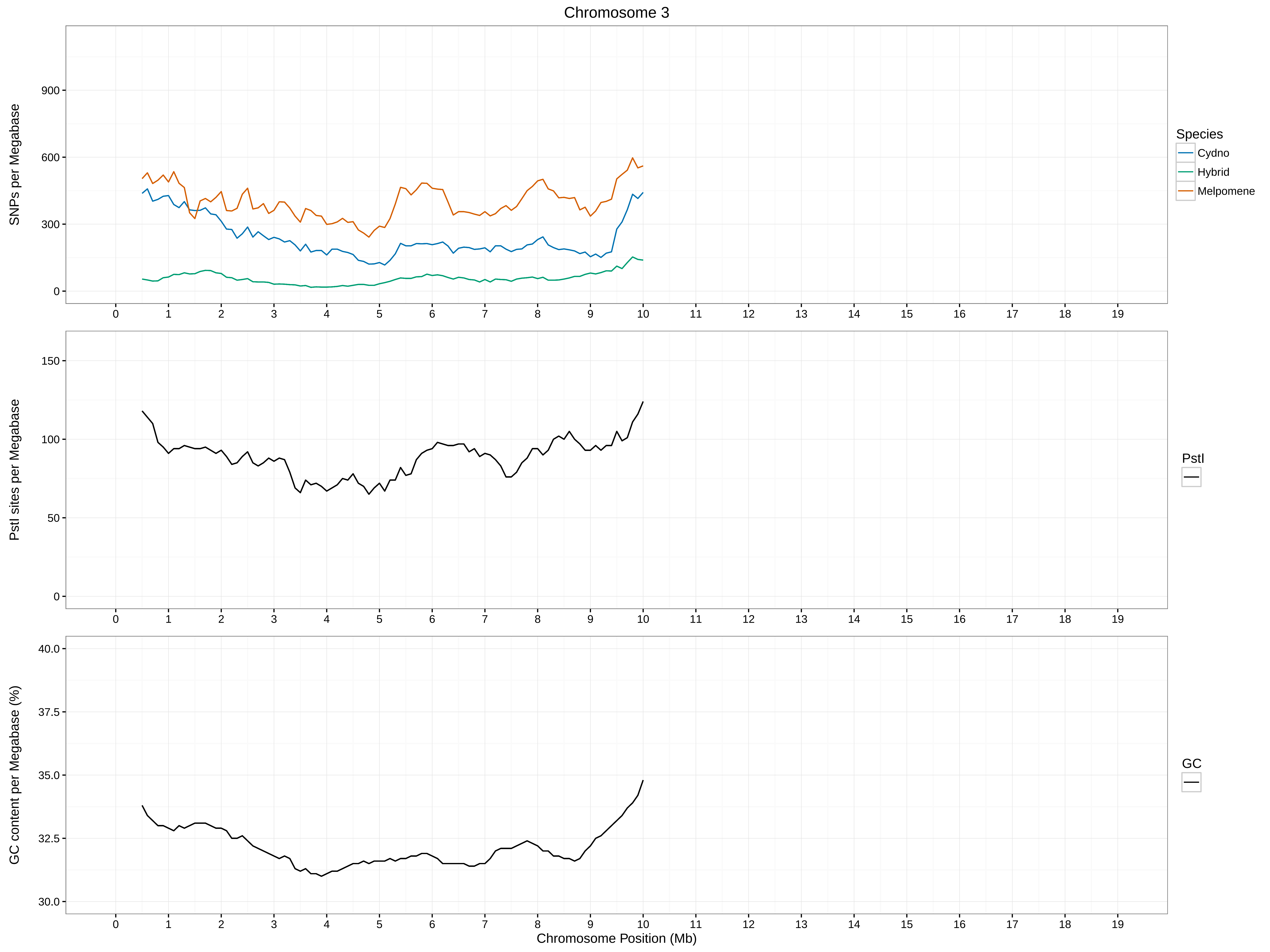
Chromosome 3
SNPs per Megabase
0
300
600
900
Species
Cydno
Hybrid
Melpomene
PstI sites per Megabase
0
50
100
150
PstI
GC content per Megabase (%)
30.0
32.5
35.0
37.5
40.0
GC
0 1 2 3 4 5 6 7 8 9 10 11 12 13 14 15 16 17 18 19
Chromosome Position (Mb)

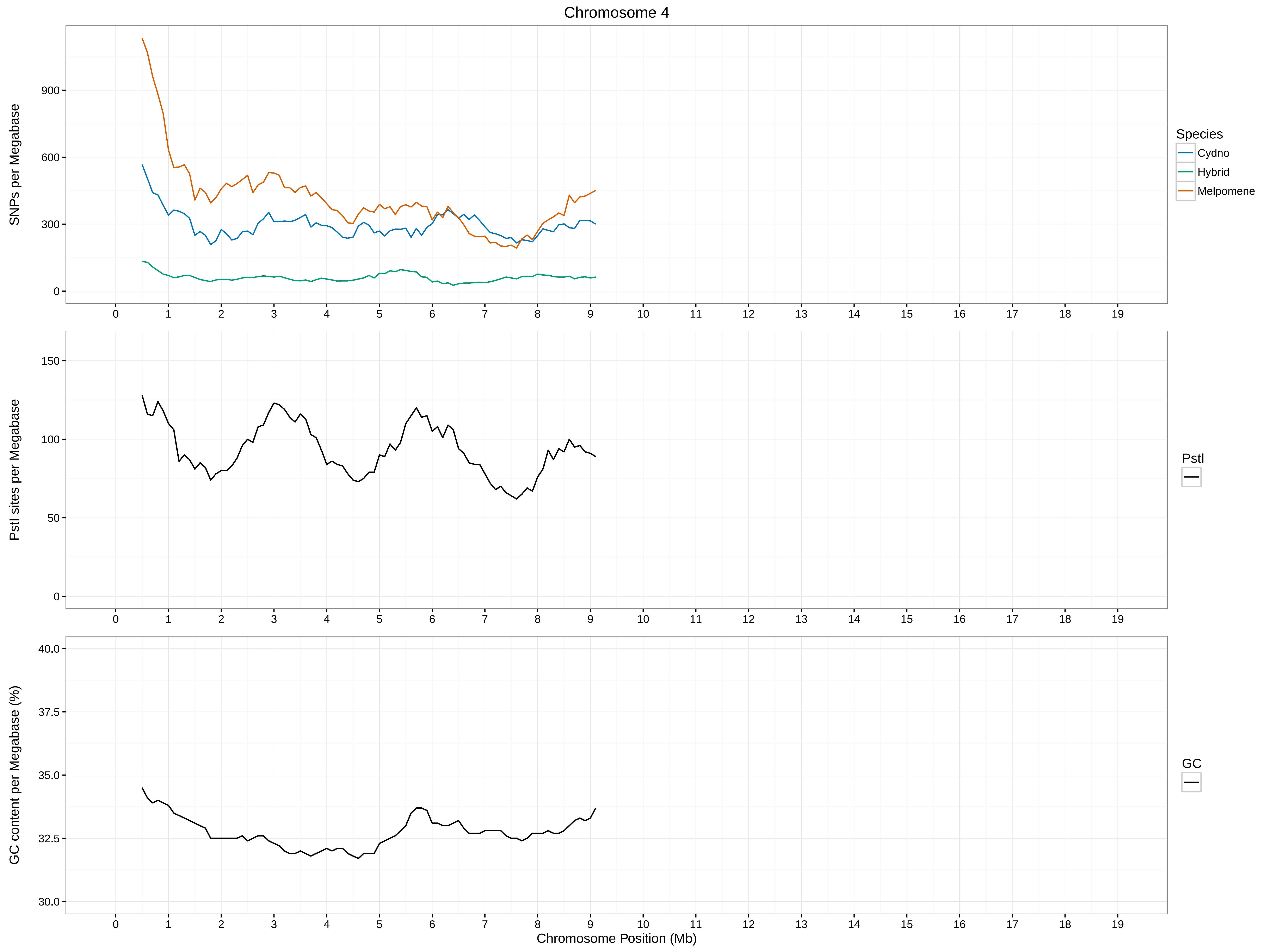
Chromosome 4
SNPs per Megabase
900
600
300
0
Species
Cydno
Hybrid
Melpomene
PstI sites per Megabase
150
100
50
0
PstI
GC content per Megabase (%)
40.0
37.5
35.0
32.5
30.0
GC
0 1 2 3 4 5 6 7 8 9 10 11 12 13 14 15 16 17 18 19
Chromosome Position (Mb)

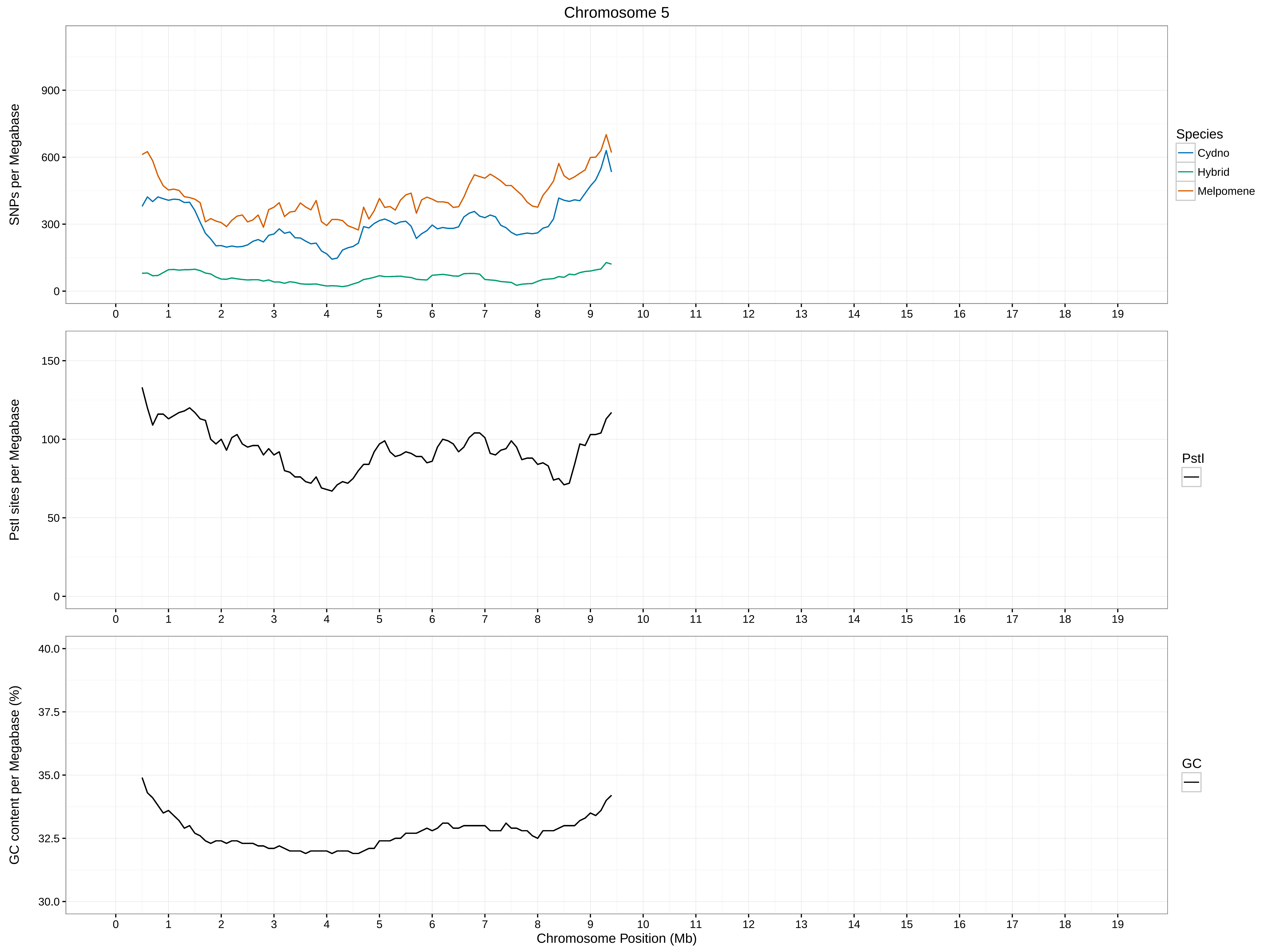

Chromosome 5
SNPs per Megabase
900
600
300
0
Species
Cydno
Hybrid
Melpomene
PstI sites per Megabase
150
100
50
0
PstI
GC content per Megabase (%)
40.0
37.5
35.0
32.5
30.0
GC
0
1
2
3
4
5
6
7
8
9
10
11
12
13
14
15
16
17
18
19
Chromosome Position (Mb)

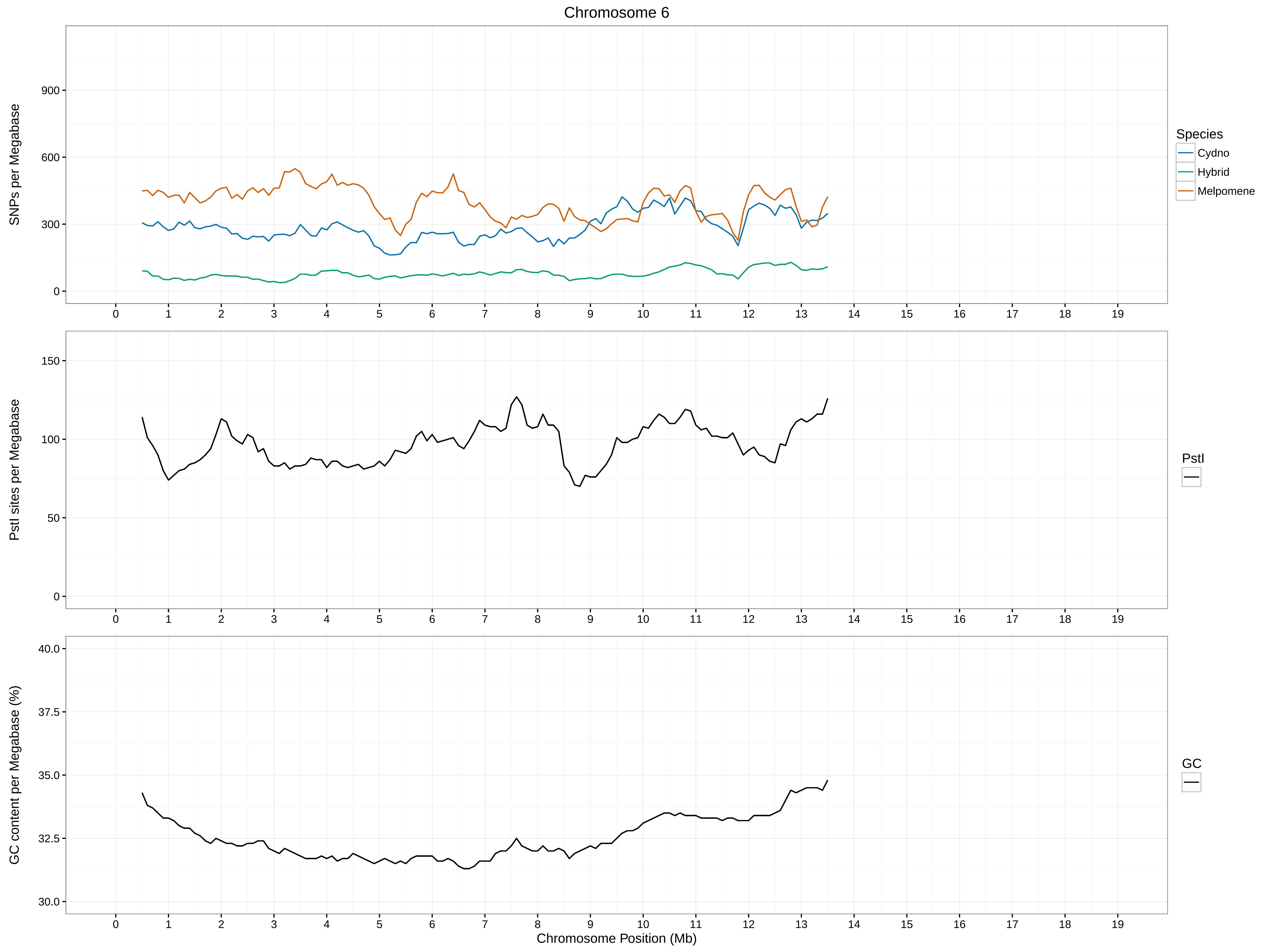
Chromosome 6
SNPs per Megabase
900
600
300
0
Species
Cydno
Hybrid
Melpomene
0
1
2
3
4
5
6
7
8
9
10
11
12
13
14
15
16
17
18
19
PstI sites per Megabase
150
100
50
0
PstI
GC content per Megabase (%)
40.0
37.5
35.0
32.5
30.0
GC
Chromosome Position (Mb)

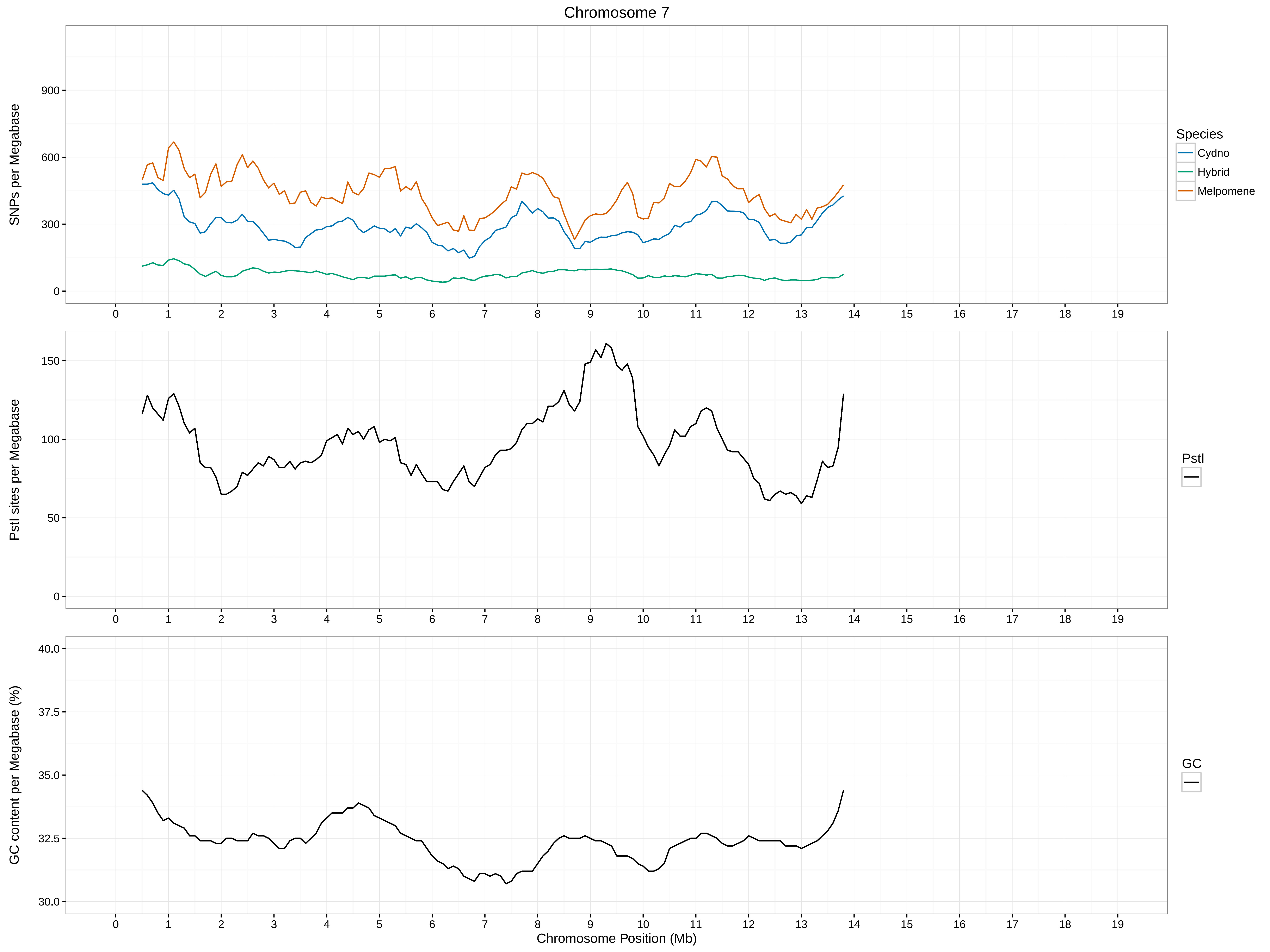
Chromosome 7
SNPs per Megabase
900
600
300
0
Species
Cydno
Hybrid
Melpomene
PstI sites per Megabase
150
100
50
0
PstI
GC content per Megabase (%)
40.0
37.5
35.0
32.5
30.0
GC
0 1 2 3 4 5 6 7 8 9 10 11 12 13 14 15 16 17 18 19
Chromosome Position (Mb)

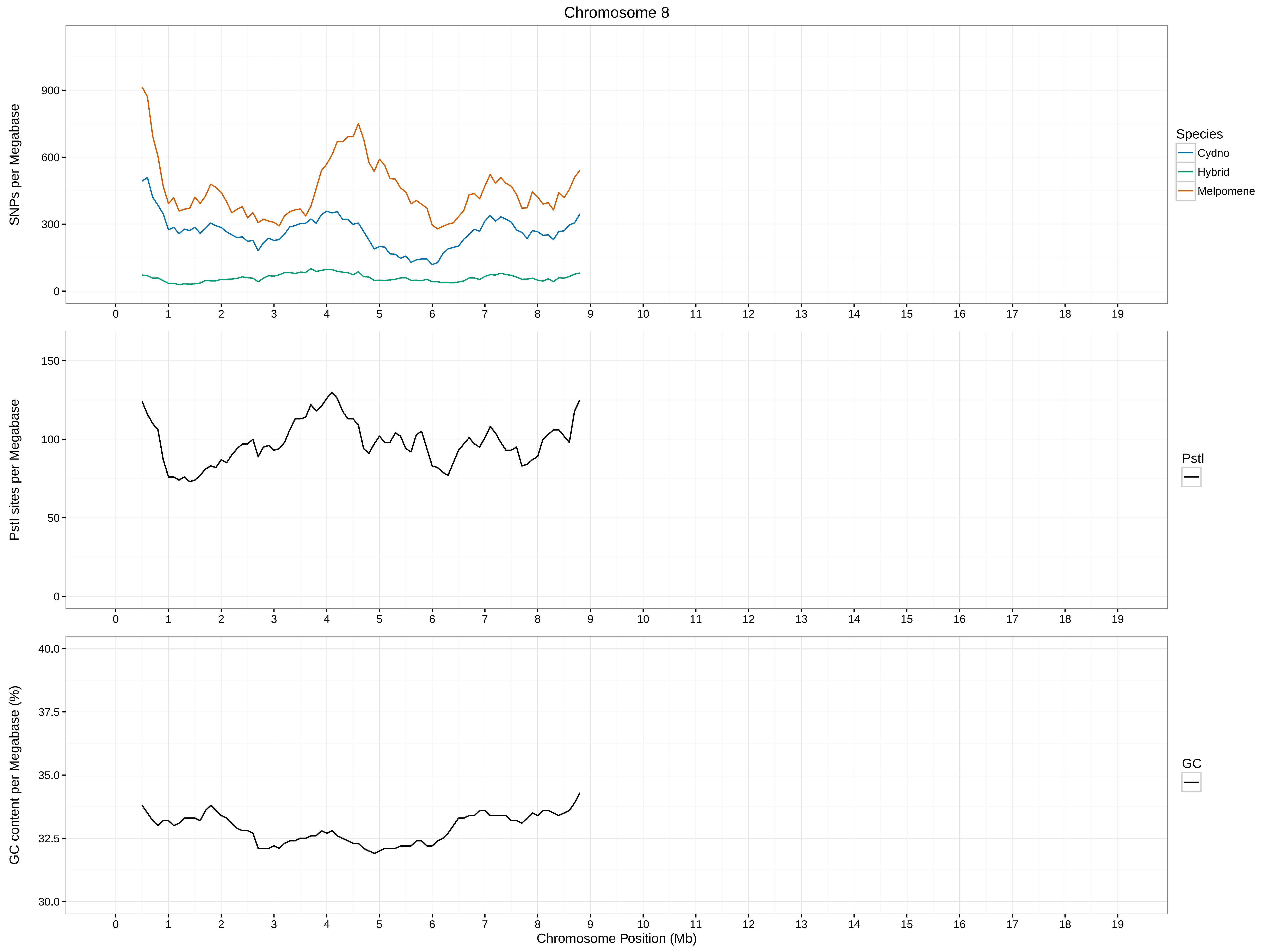
Chromosome 8
SNPs per Megabase
Species
Cydno
Hybrid
Melpomene
PstI sites per Megabase
PstI
GC content per Megabase (%)
GC
Chromosome Position (Mb)

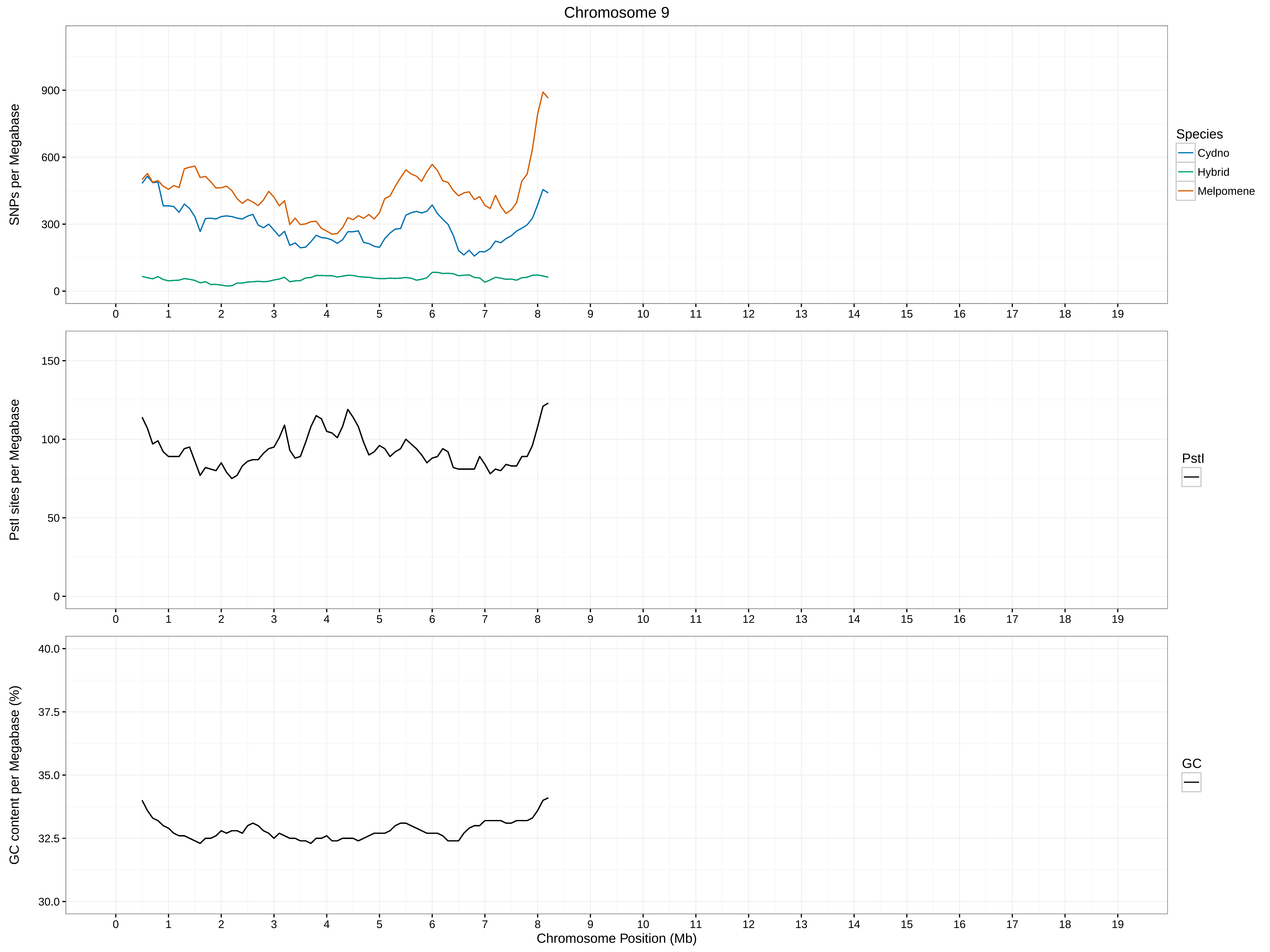

Chromosome 9
SNPs per Megabase
900
600
300
0
Species
Cydno
Hybrid
Melpomene
PstI sites per Megabase
150
100
50
0
PstI
GC content per Megabase (%)
40.0
37.5
35.0
32.5
30.0
GC
0 1 2 3 4 5 6 7 8 9 10 11 12 13 14 15 16 17 18 19
Chromosome Position (Mb)

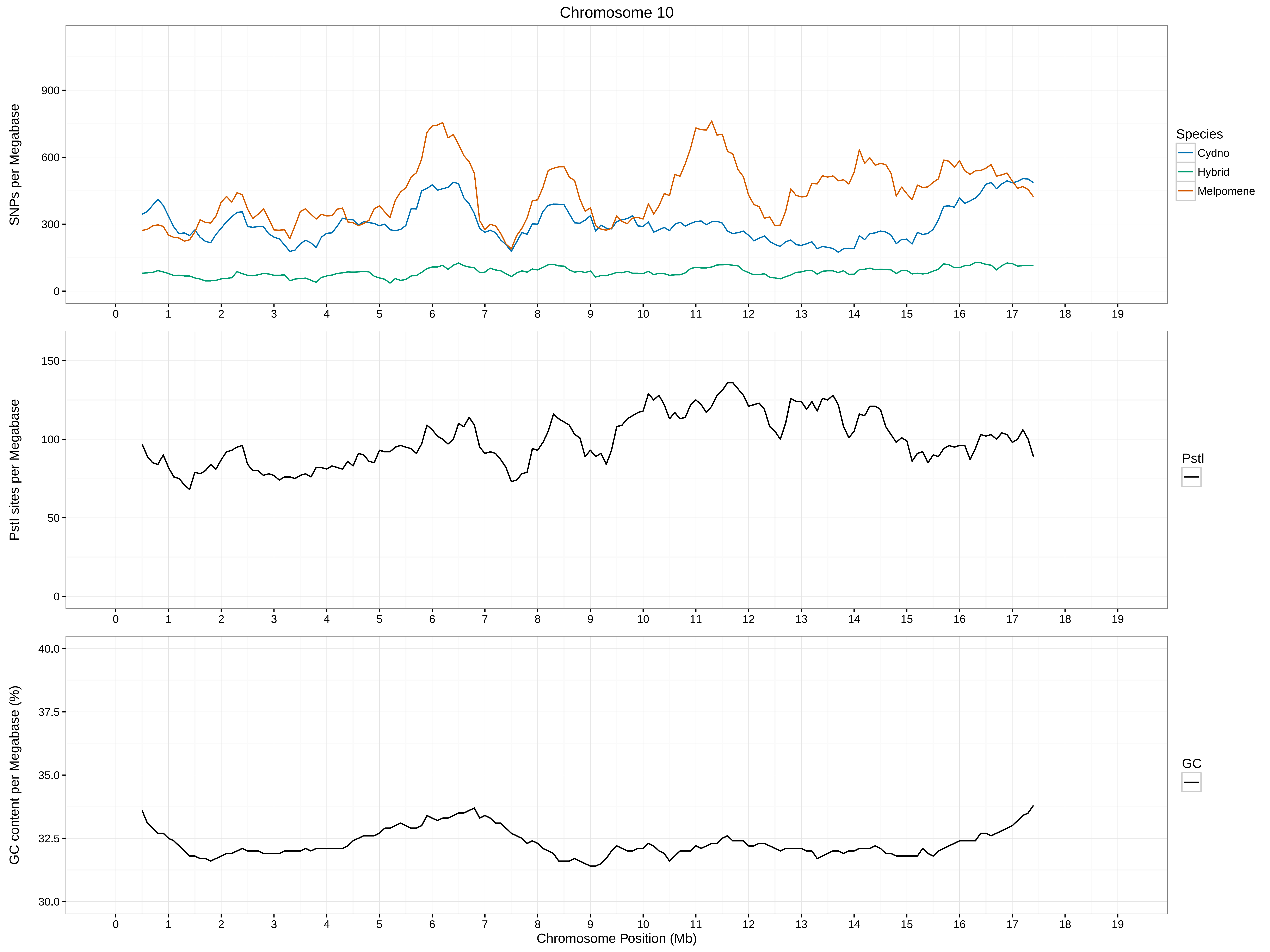

Chromosome 10
SNPs per Megabase
900
600
300
0
Species
Cydno
Hybrid
Melpomene
0
1
2
3
4
5
6
7
8
9
10
11
12
13
14
15
16
17
18
19
PstI sites per Megabase
150
100
50
0
PstI
GC content per Megabase (%)
40.0
37.5
35.0
32.5
30.0
GC
Chromosome Position (Mb)

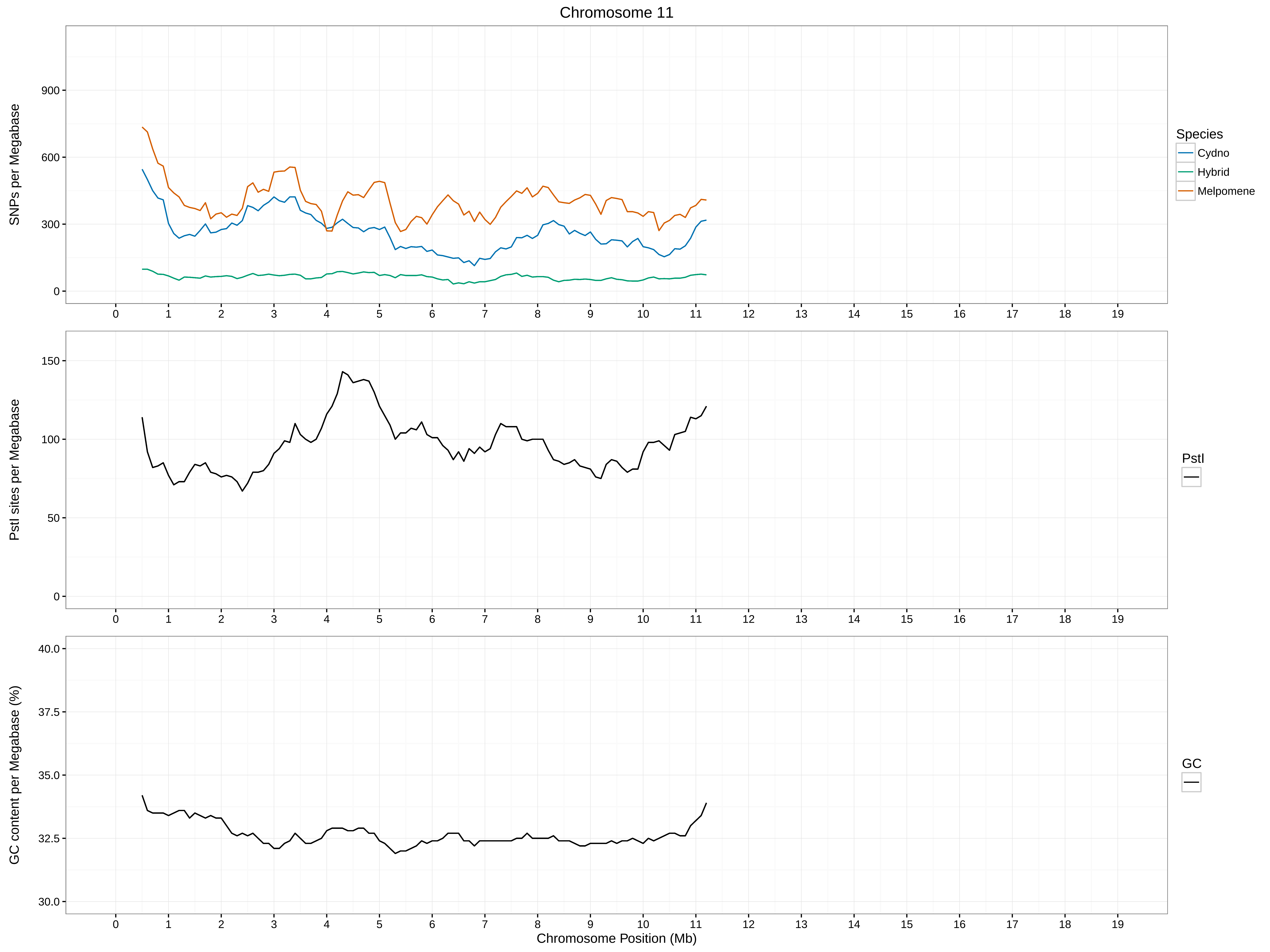

Chromosome 11
SNPs per Megabase
900
600
300
0
Species
Cydno
Hybrid
Melpomene
0
1
2
3
4
5
6
7
8
9
10
11
12
13
14
15
16
17
18
19
PstI sites per Megabase
150
100
50
0
PstI
GC content per Megabase (%)
40.0
37.5
35.0
32.5
30.0
GC
Chromosome Position (Mb)

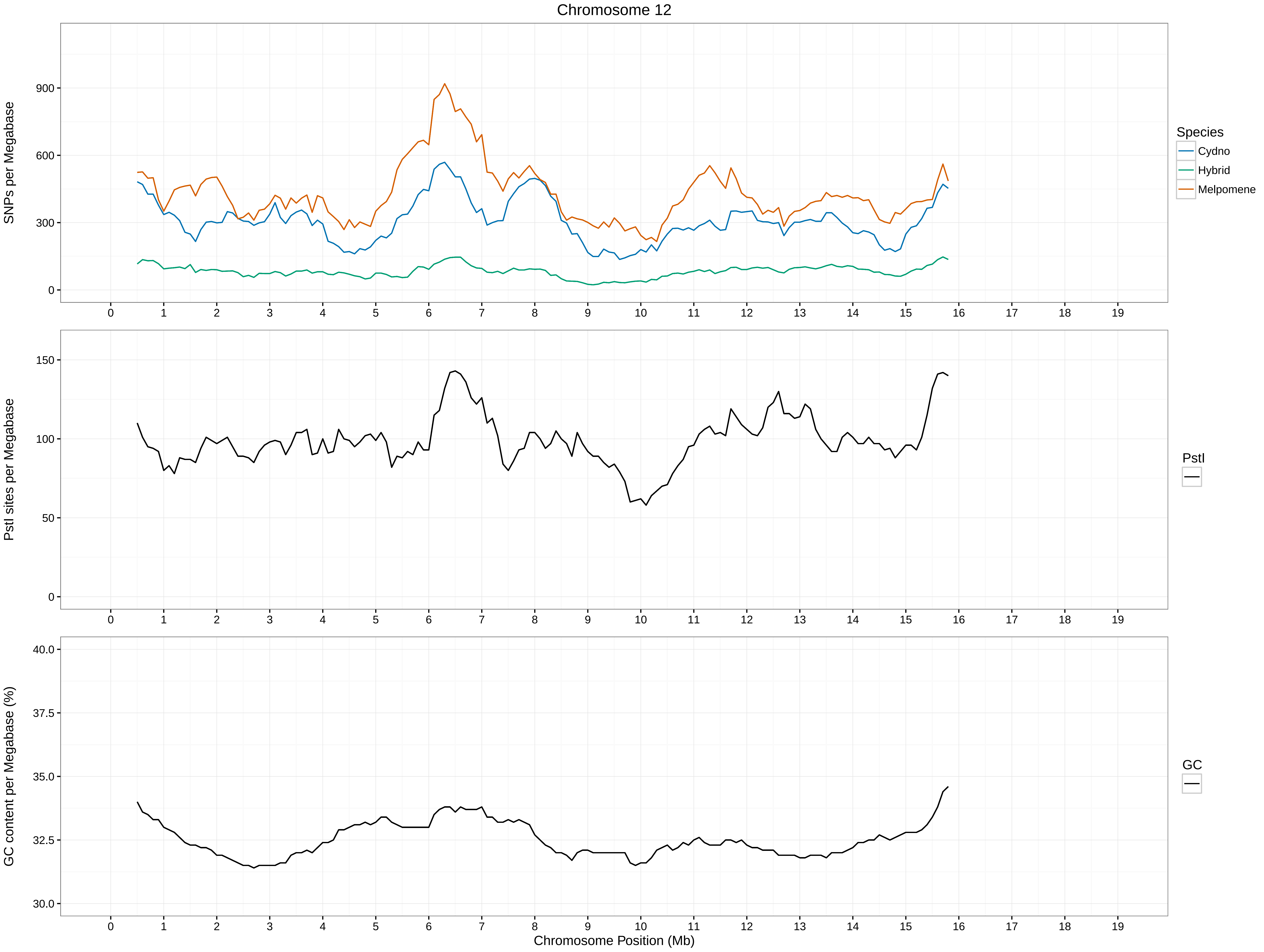
Chromosome 12
SNPs per Megabase
900
600
300
0
Species
Cydno
Hybrid
Melpomene
PstI sites per Megabase
150
100
50
0
PstI
GC content per Megabase (%)
40.0
37.5
35.0
32.5
30.0
GC
0 1 2 3 4 5 6 7 8 9 10 11 12 13 14 15 16 17 18 19
Chromosome Position (Mb)

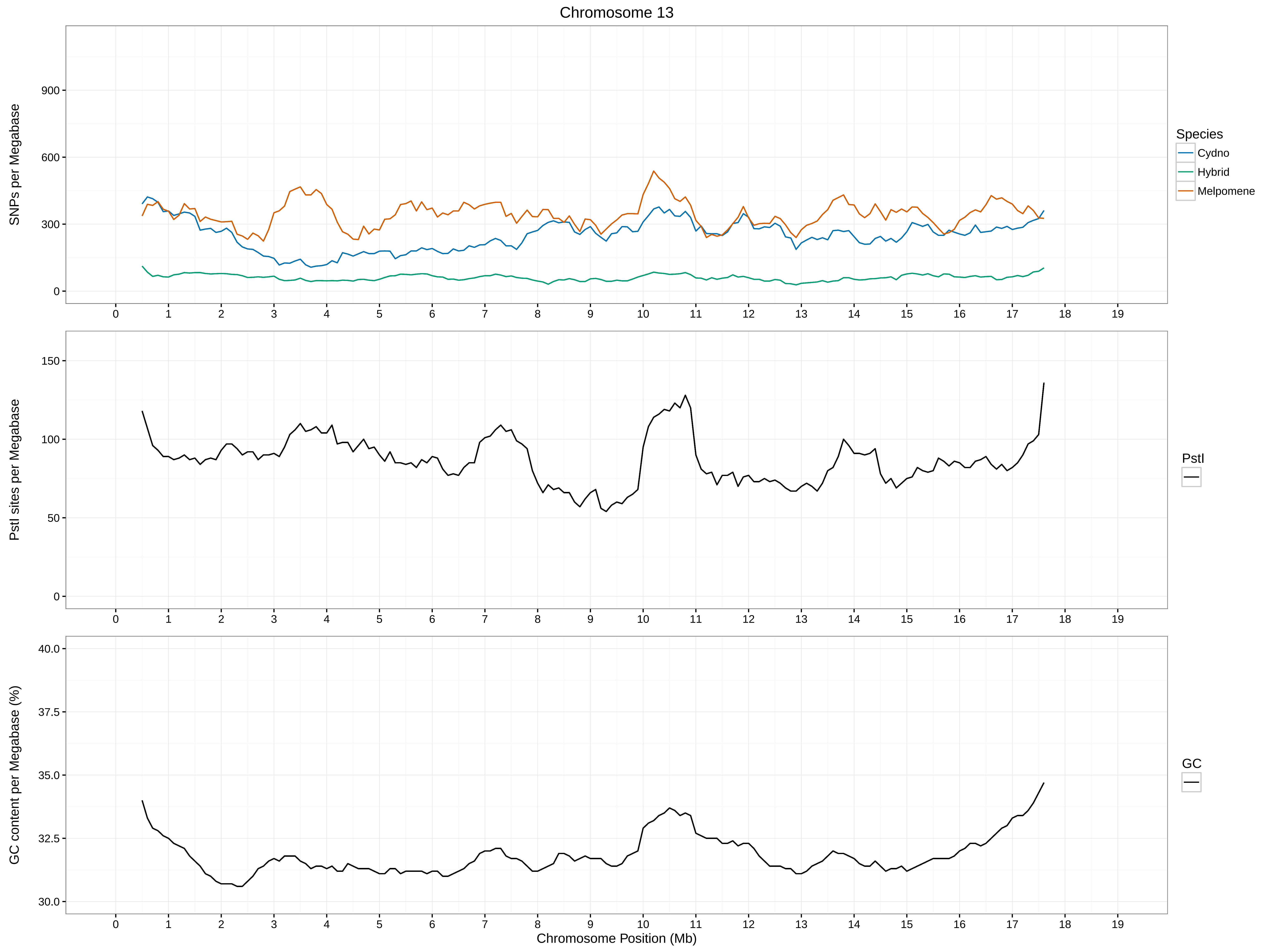
Chromosome 13
SNPs per Megabase
900
600
300
0
Species
Cydno
Hybrid
Melpomene
PstI sites per Megabase
150
100
50
0
PstI
GC content per Megabase (%)
40.0
37.5
35.0
32.5
30.0
GC
0
1
2
3
4
5
6
7
8
9
10
11
12
13
14
15
16
17
18
19
Chromosome Position (Mb)

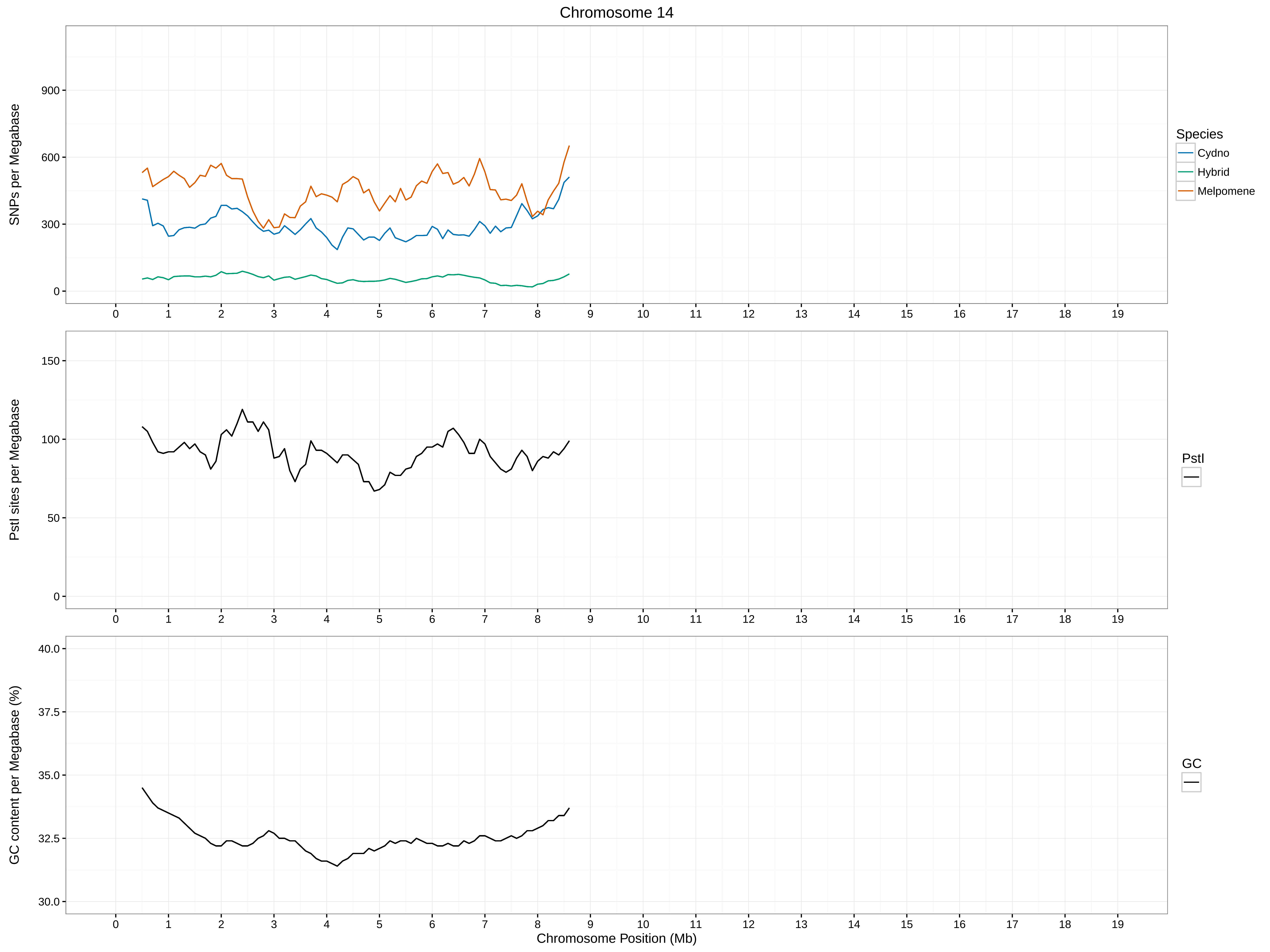
Chromosome 14
SNPs per Megabase
900
600
300
0
Species
Cydno
Hybrid
Melpomene
0 1 2 3 4 5 6 7 8 9 10 11 12 13 14 15 16 17 18 19
PstI sites per Megabase
150
100
50
0
PstI
0 1 2 3 4 5 6 7 8 9 10 11 12 13 14 15 16 17 18 19
GC content per Megabase (%)
40.0
37.5
35.0
32.5
30.0
GC
0 1 2 3 4 5 6 7 8 9 10 11 12 13 14 15 16 17 18 19
Chromosome Position (Mb)

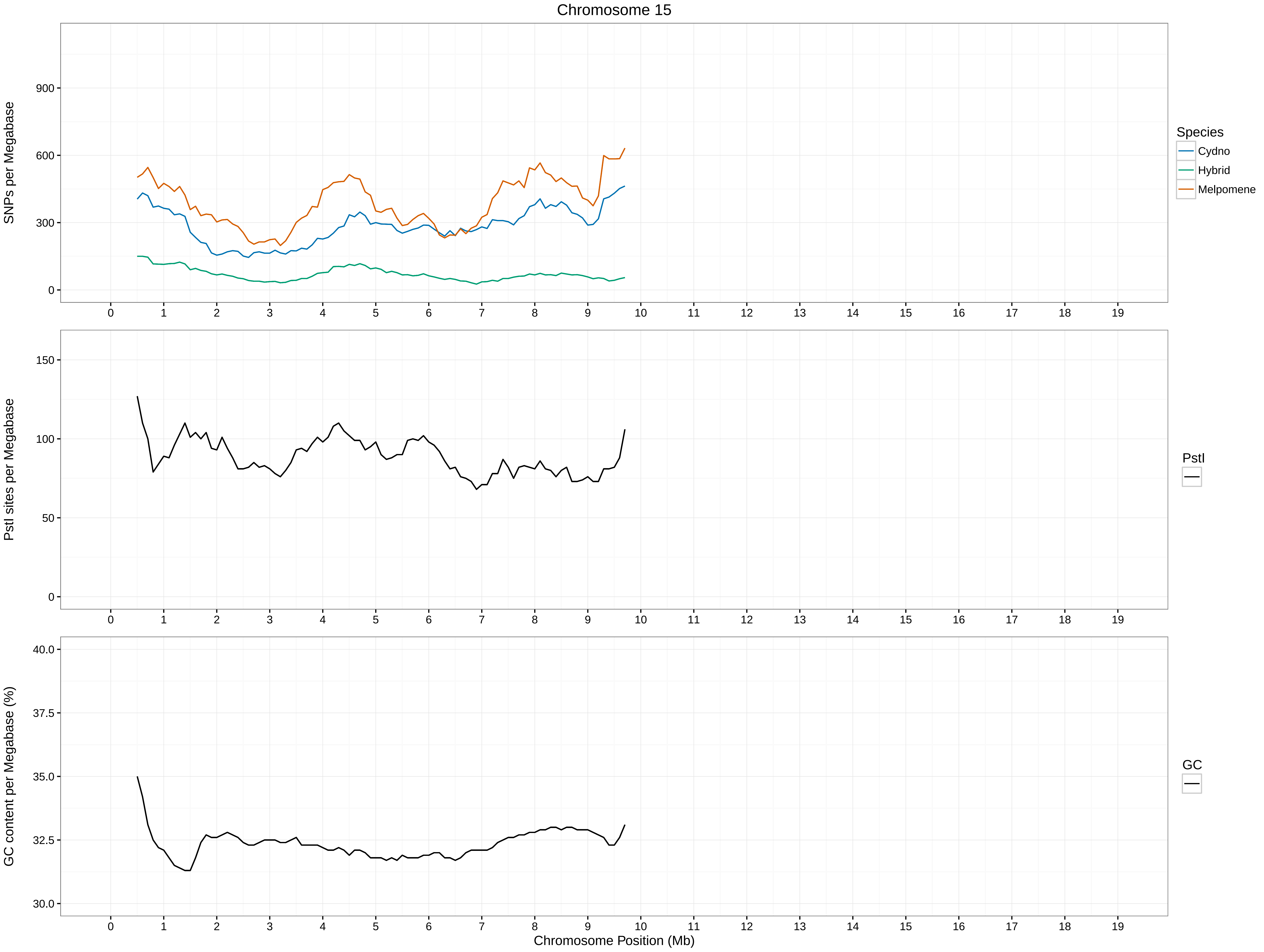
Chromosome 15
SNPs per Megabase
900
600
300
0
Species
Cydno
Hybrid
Melpomene
PstI sites per Megabase
150
100
50
0
PstI
GC content per Megabase (%)
40.0
37.5
35.0
32.5
30.0
GC
0 1 2 3 4 5 6 7 8 9 10 11 12 13 14 15 16 17 18 19
Chromosome Position (Mb)

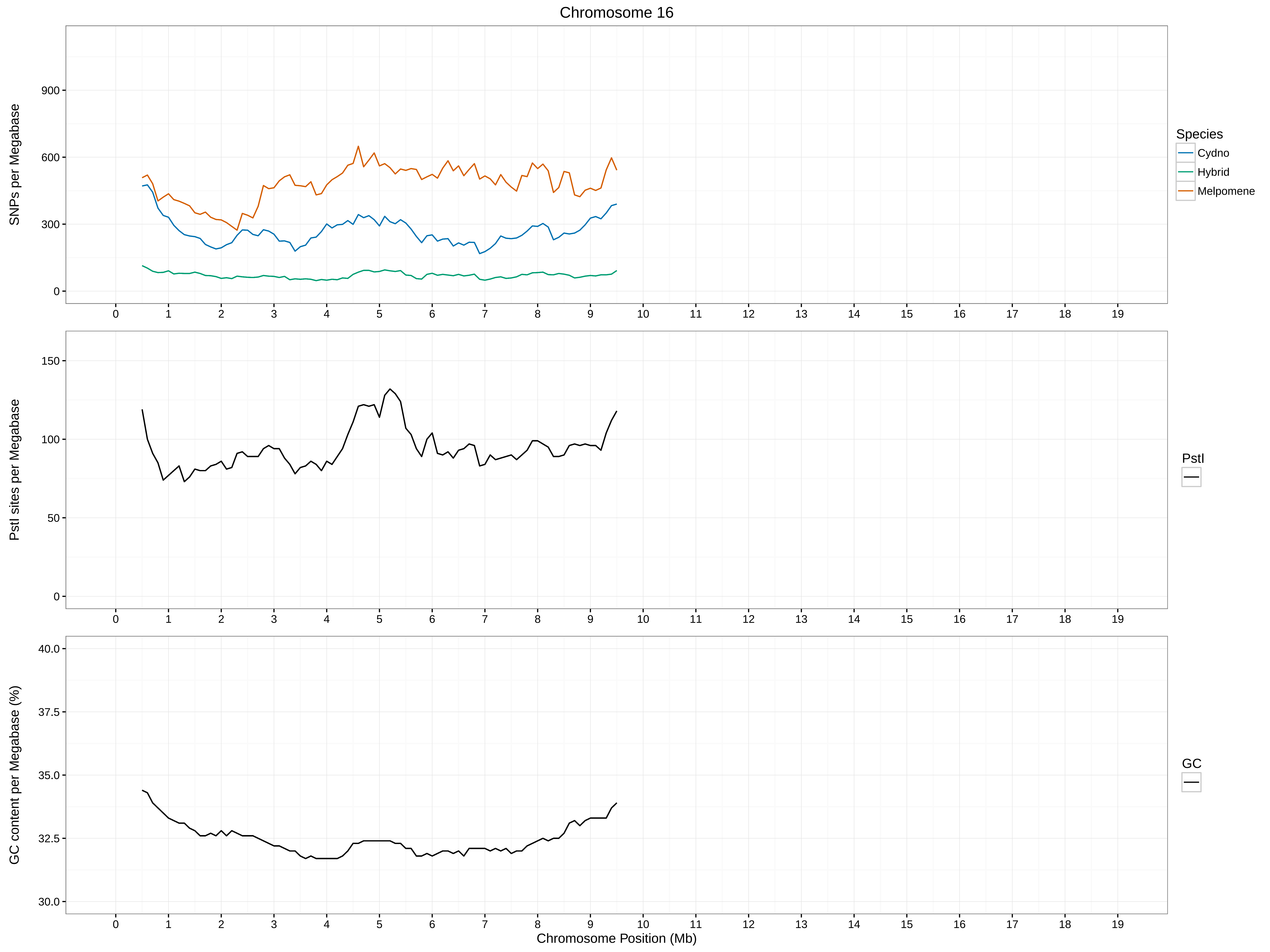

Chromosome 16
SNPs per Megabase
900
600
300
0
Species
Cydno
Hybrid
Melpomene
PstI sites per Megabase
150
100
50
0
PstI
GC content per Megabase (%)
40.0
37.5
35.0
32.5
30.0
GC
0 1 2 3 4 5 6 7 8 9 10 11 12 13 14 15 16 17 18 19
Chromosome Position (Mb)

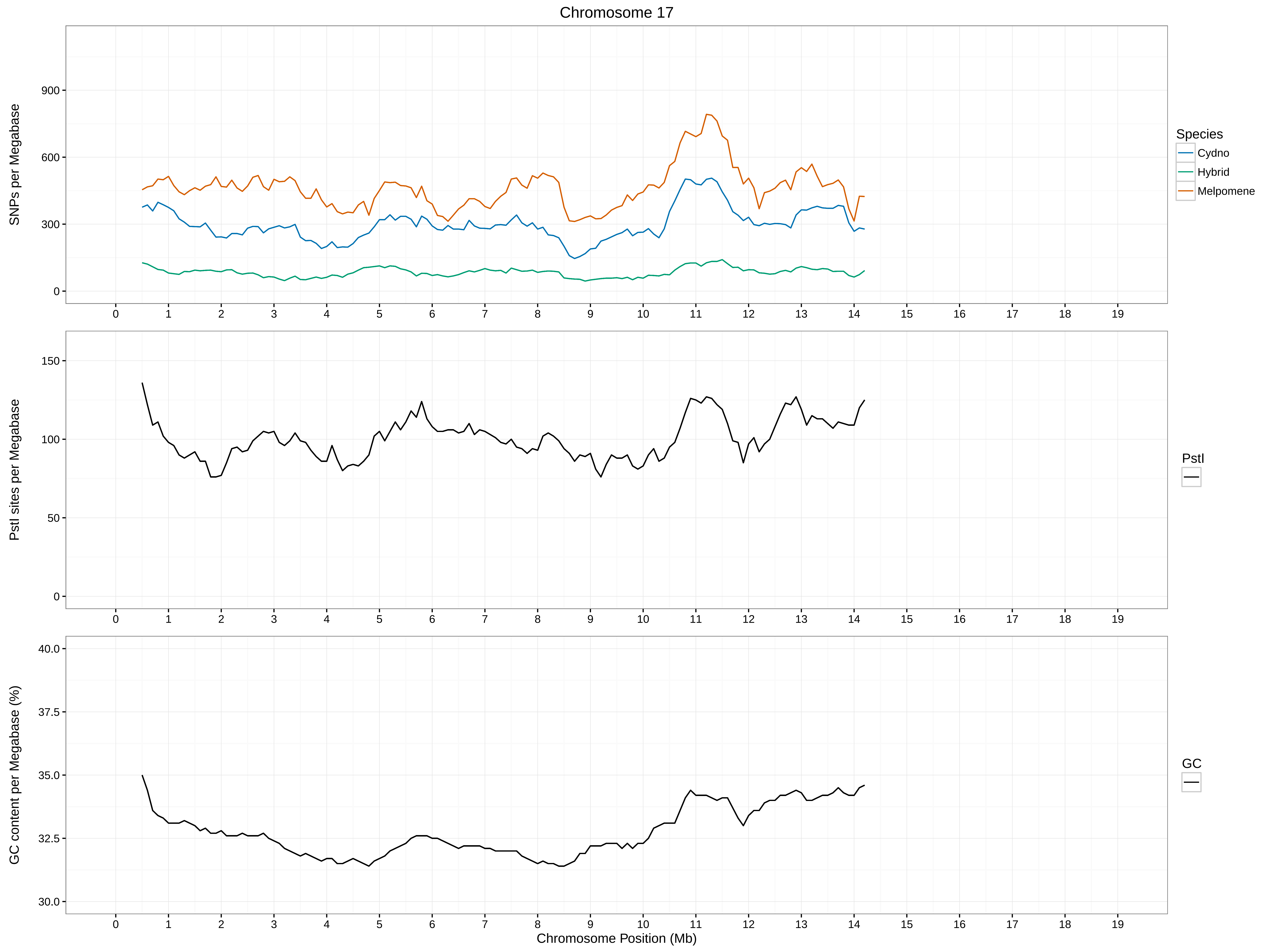

Chromosome 17
SNPs per Megabase
900
600
300
0
Species
Cydno
Hybrid
Melpomene
PstI sites per Megabase
150
100
50
0
PstI
GC content per Megabase (%)
40.0
37.5
35.0
32.5
30.0
GC
0 1 2 3 4 5 6 7 8 9 10 11 12 13 14 15 16 17 18 19
Chromosome Position (Mb)

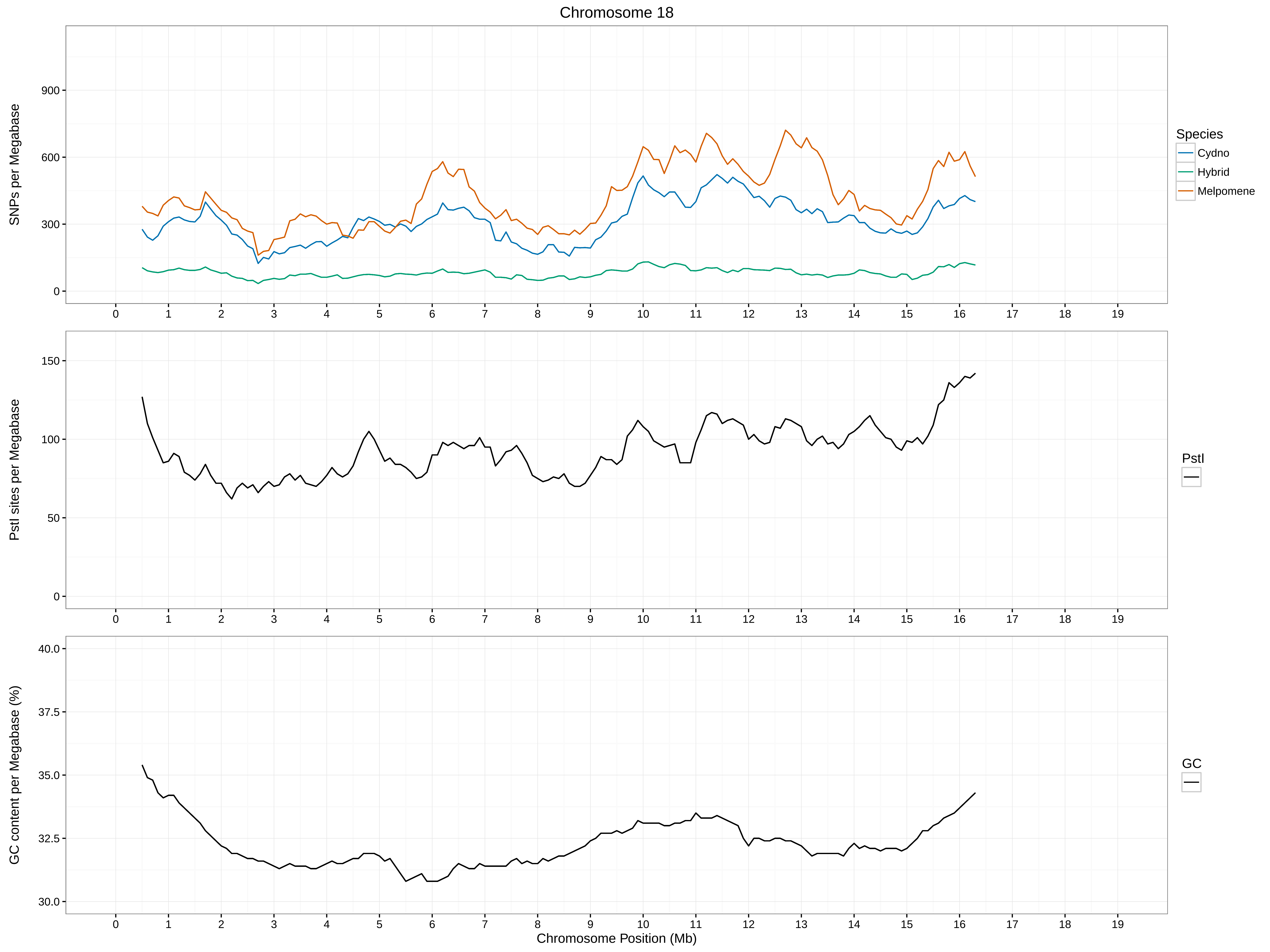

Chromosome 18
SNPs per Megabase
0
300
600
900
Species
Cydno
Hybrid
Melpomene
PstI sites per Megabase
0
50
100
150
PstI
GC content per Megabase (%)
30.0
32.5
35.0
37.5
40.0
GC
0 1 2 3 4 5 6 7 8 9 10 11 12 13 14 15 16 17 18 19
Chromosome Position (Mb)

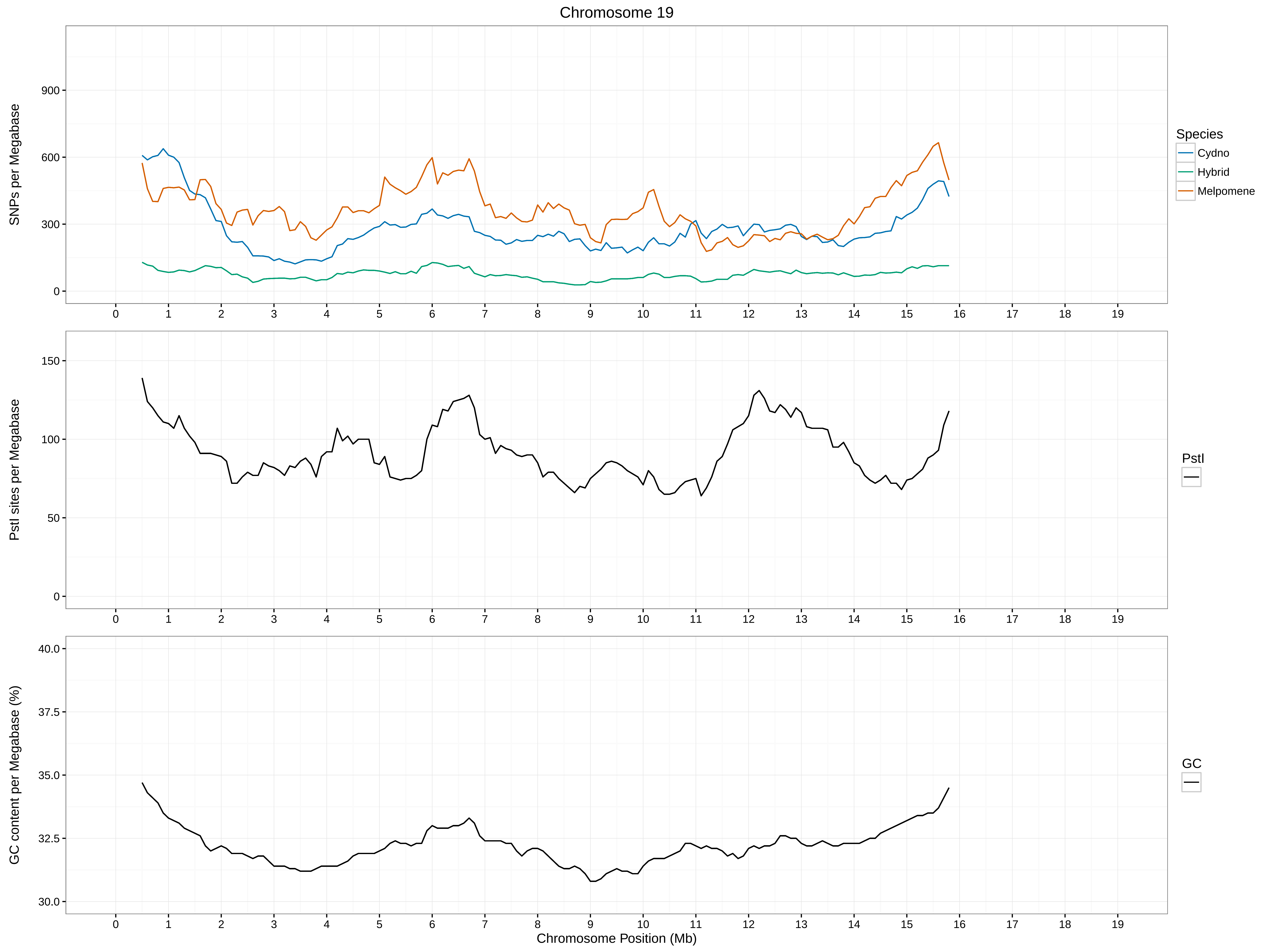

Chromosome 19
SNPs per Megabase
Species
Cydno
Hybrid
Melpomene
PstI sites per Megabase
PstI
GC content per Megabase (%)
GC
Chromosome Position (Mb)

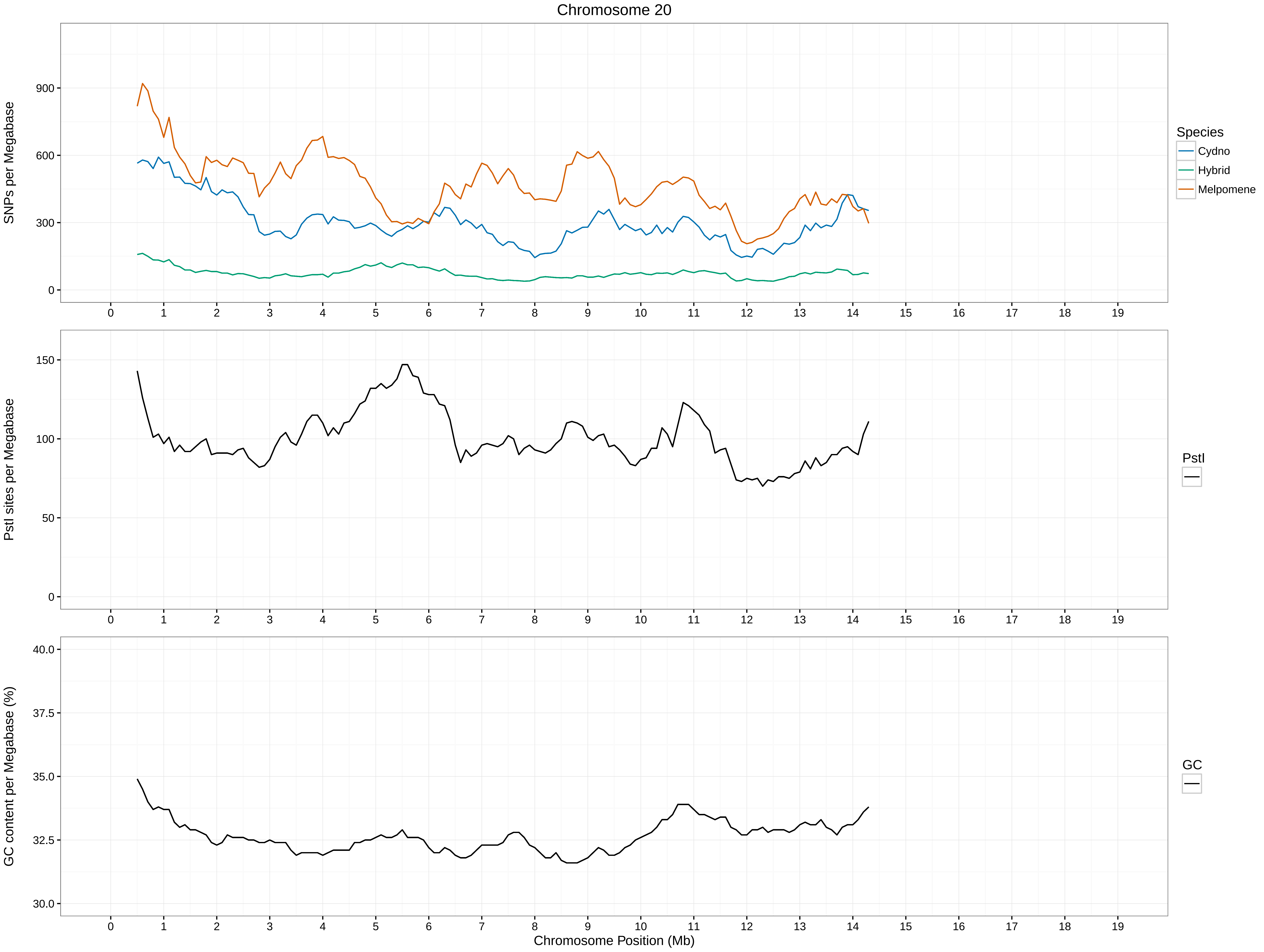
Chromosome 20
SNPs per Megabase
900
600
300
0
Species
Cydno
Hybrid
Melpomene
PstI sites per Megabase
150
100
50
0
PstI
GC content per Megabase (%)
40.0
37.5
35.0
32.5
30.0
GC
0 1 2 3 4 5 6 7 8 9 10 11 12 13 14 15 16 17 18 19
Chromosome Position (Mb)

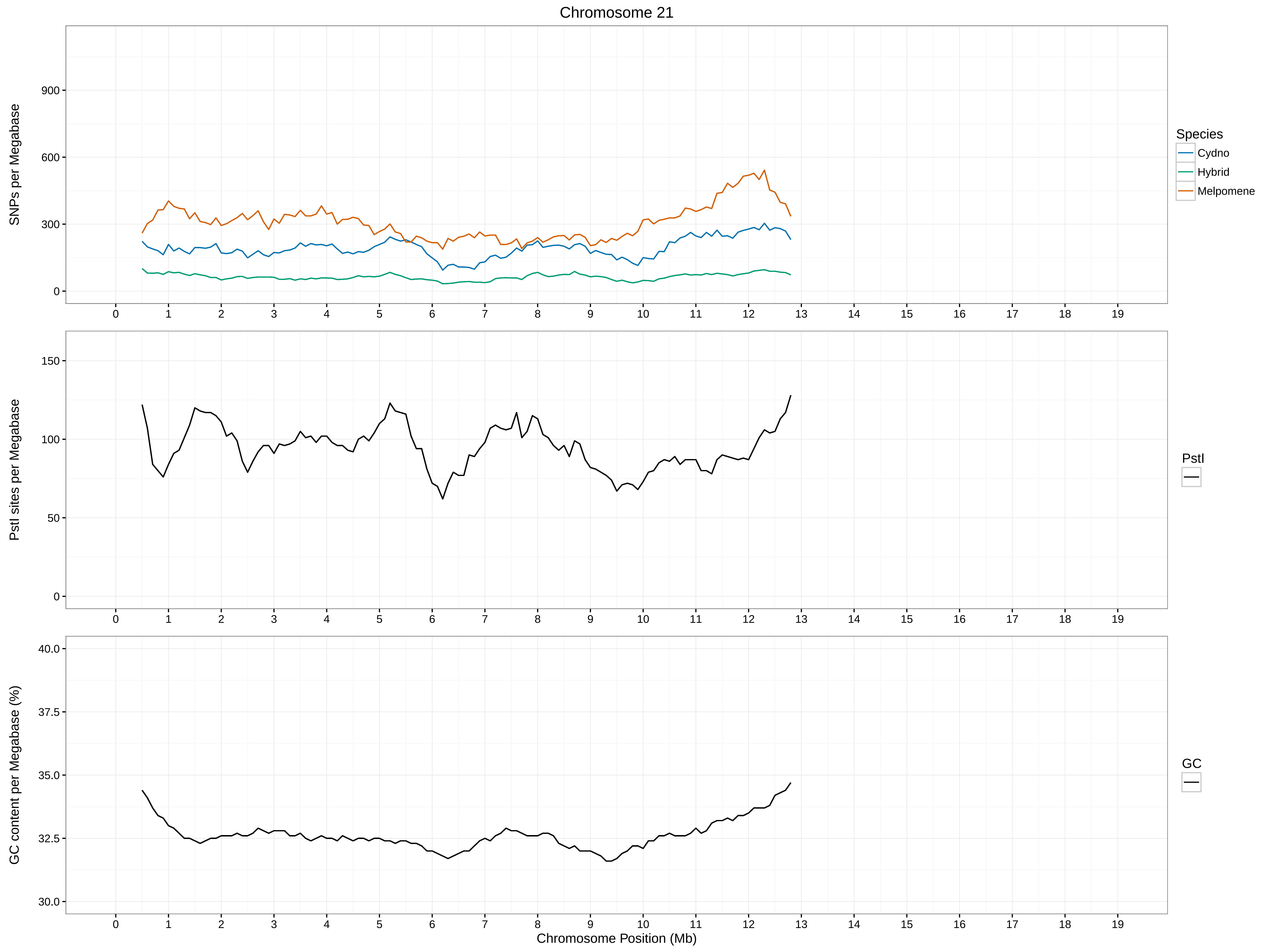

Chromosome 21
SNPs per Megabase
900
600
300
0
Species
Cydno
Hybrid
Melpomene
PstI sites per Megabase
150
100
50
0
PstI
GC content per Megabase (%)
40.0
37.5
35.0
32.5
30.0
GC
0
1
2
3
4
5
6
7
8
9
10
11
12
13
14
15
16
17
18
19
Chromosome Position (Mb)
