# Supplementary figures and images for: No evidence for maintenance of a sympatric Heliconius species barrier by chromosomal inversions
Source: Evol Lett. 2017 Jun 14;1(3):138–54. doi: 10.1002/evl3.12 (PMC6122123; doi:10.1002/evl3.12)

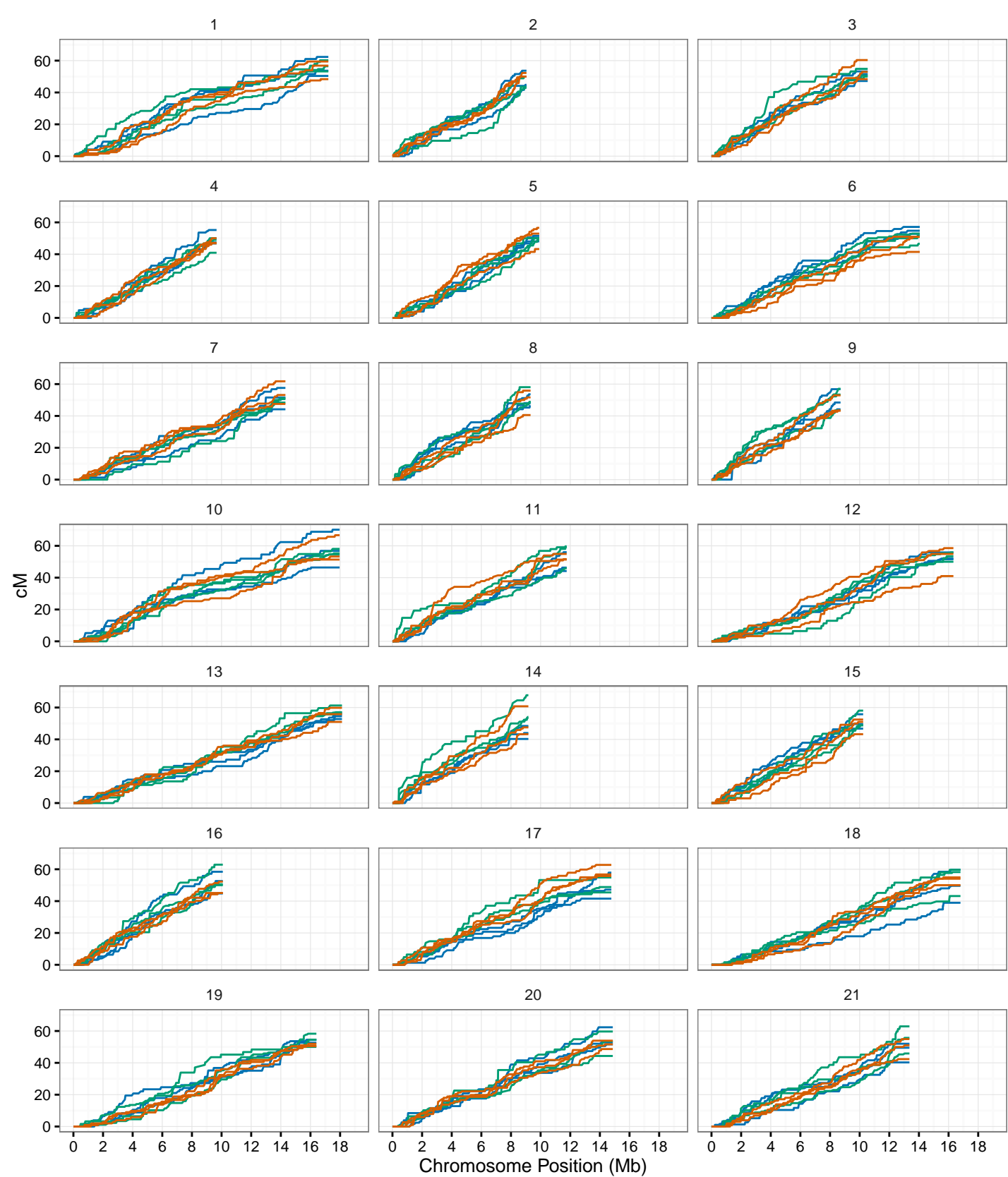

Supplement: Supplementary file 5 — Figure S4. Marey maps of recombinations for each cross separately. [file EVL3-1-138-s005.pdf]

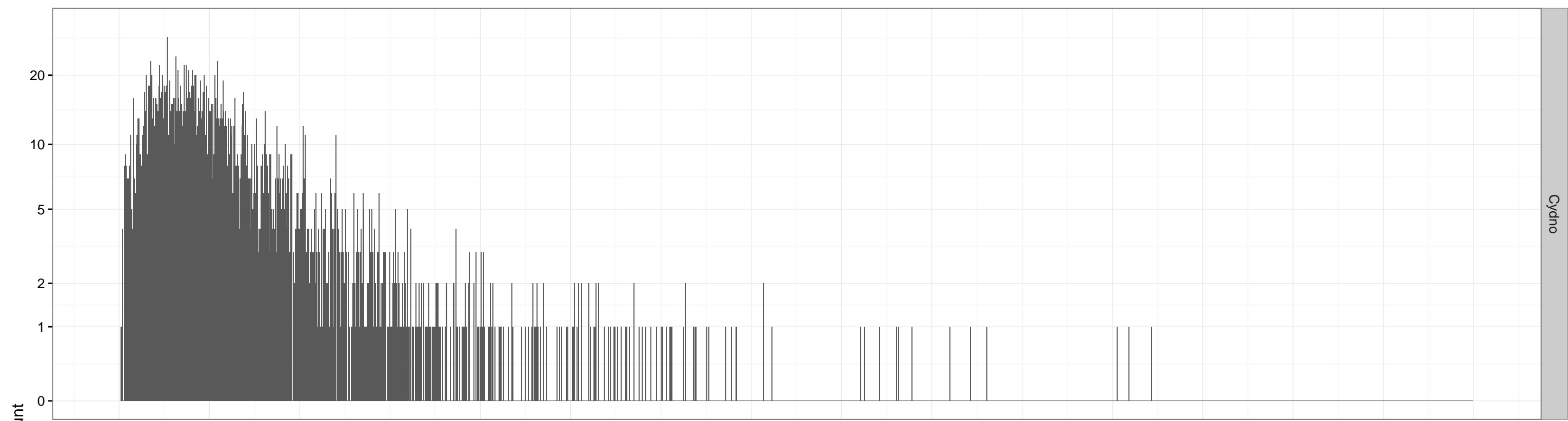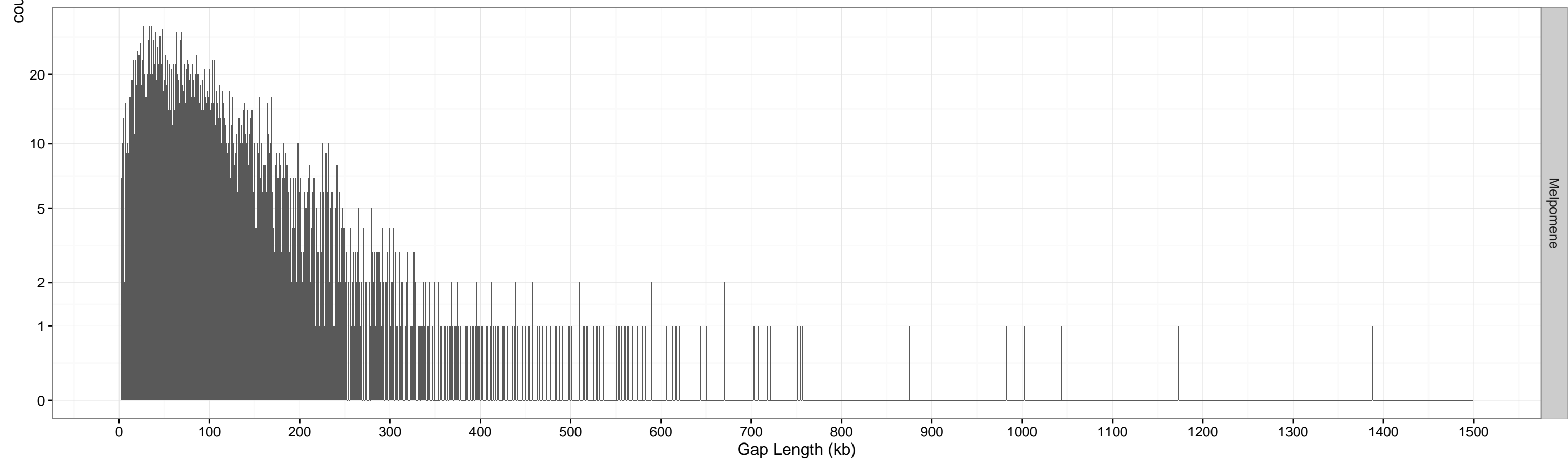

Supplement: Supplementary file 8 — Figure S7. Histograms of gap lengths for Heliconius cydno and H. melpomene. [file EVL3-1-138-s008.pdf]

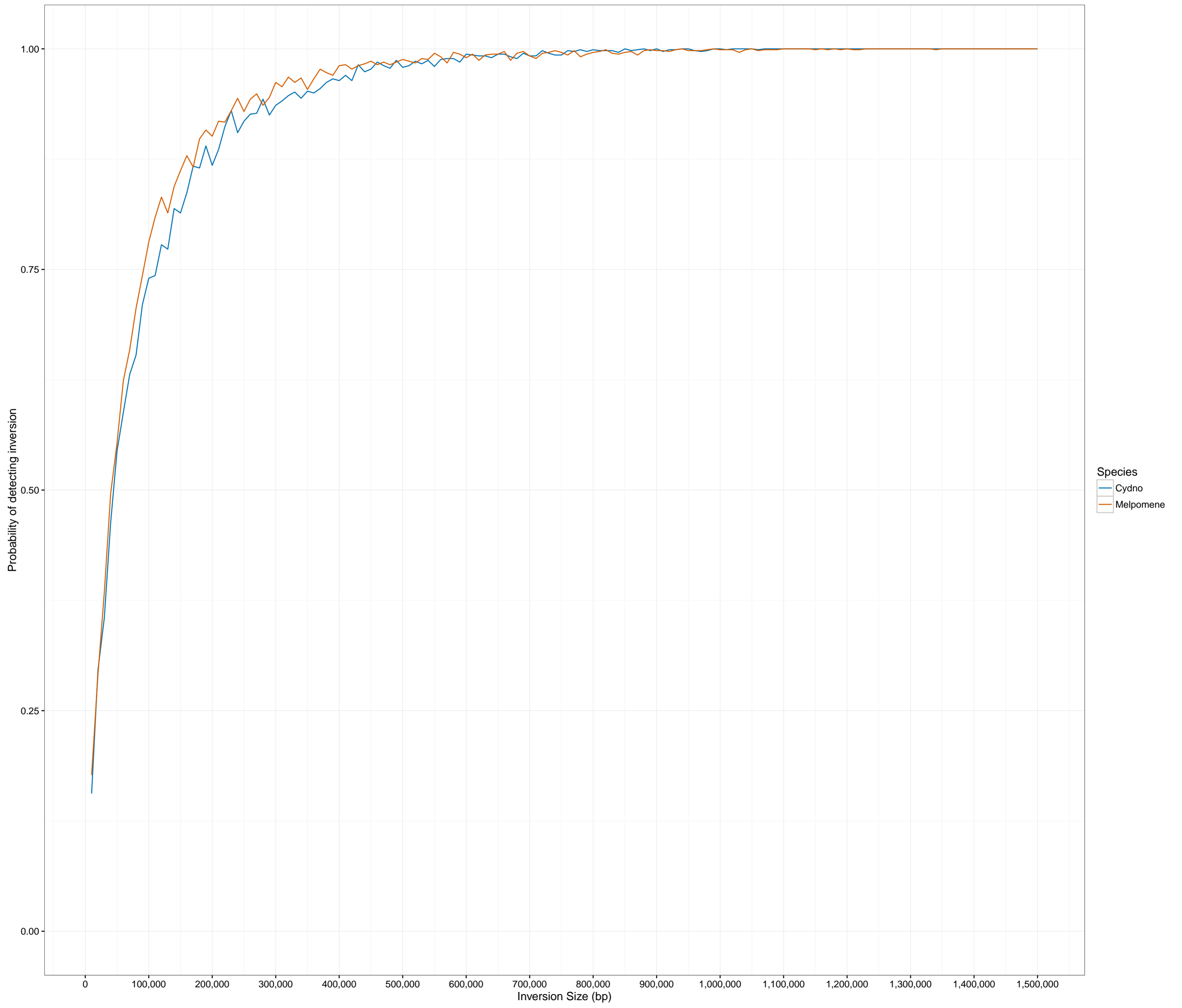

Supplement: Supplementary file 9 — Figure S8. Probability of detecting random inversions of sizes from 10 kb to 1.5 Mb given existing linkage maps for Heliconius melpomene and H. cydno. [file EVL3-1-138-s009.pdf]

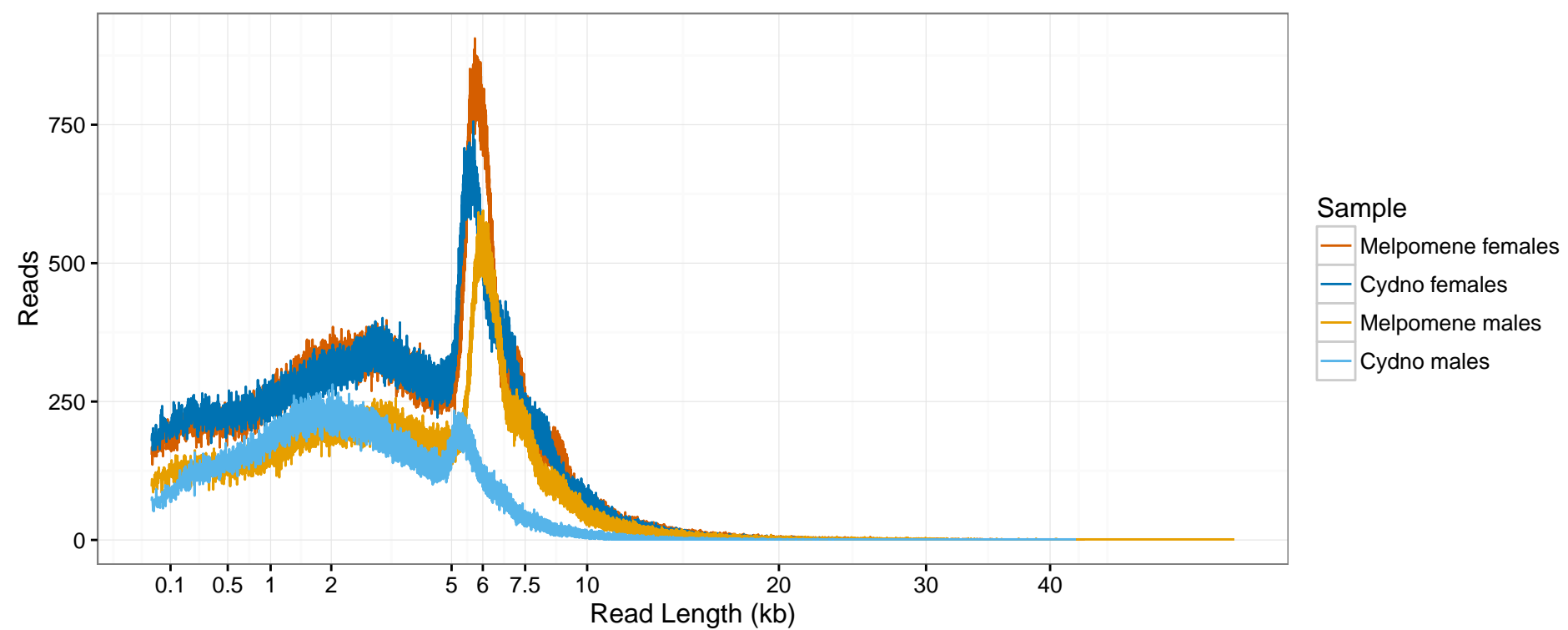

Supplement: Supplementary file 10 — Figure S9. Histograms of raw read lengths for Pacific Biosciences sequencing. [file EVL3-1-138-s010.pdf]

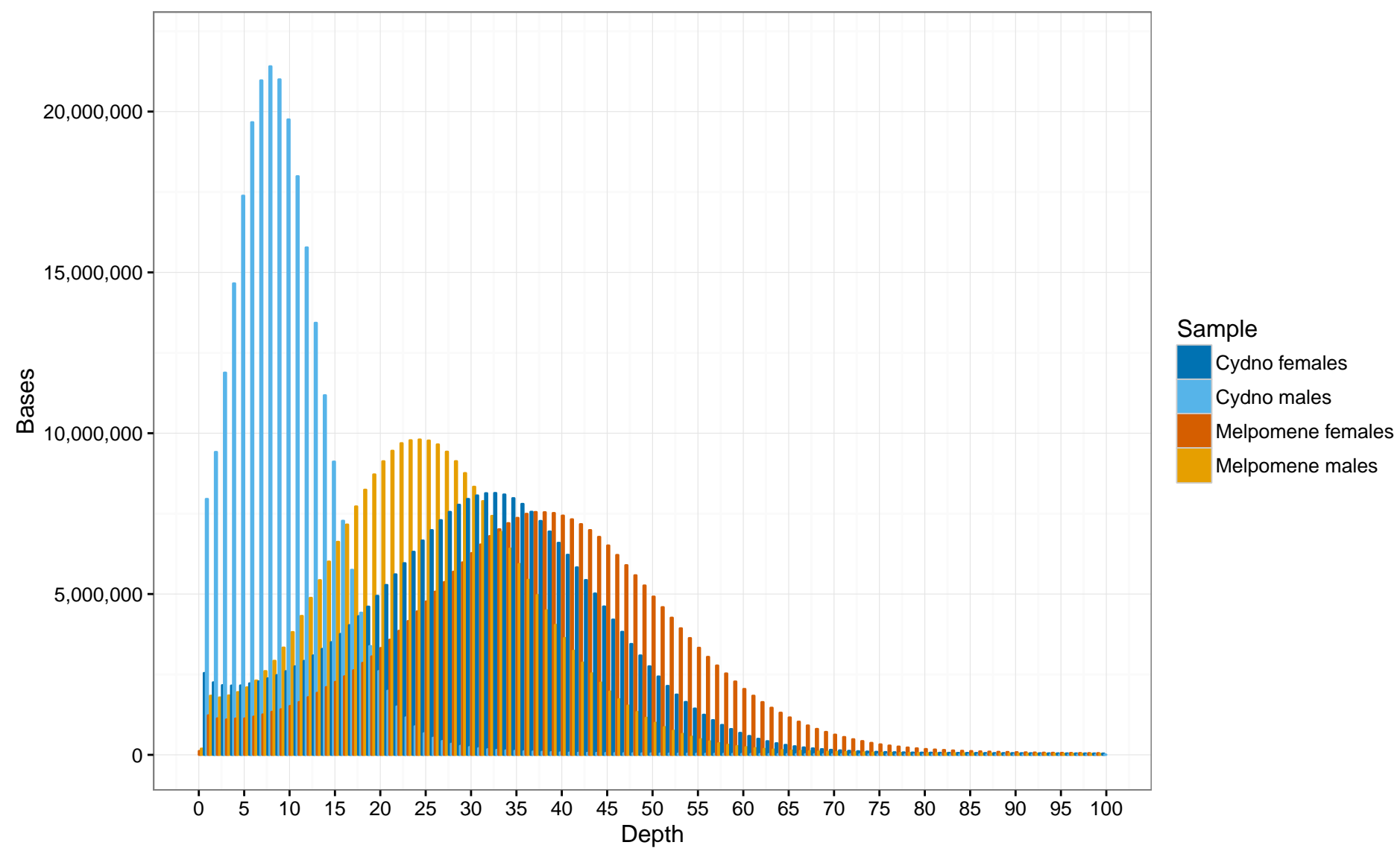

Supplement: Supplementary file 11 — Figure S10. Histograms of base depths across the genome after alignment of raw PacBio reads to Heliconius melpomene genome assembly Hmel2. [file EVL3-1-138-s011.pdf]
