# Supplementary material for: No evidence for maintenance of a sympatric Heliconius species barrier by chromosomal inversions
Source: Evol Lett. 2017 Jun 14;1(3):138–54. doi: 10.1002/evl3.12 (PMC6122123; doi:10.1002/evl3.12)
Supplement: Supplementary file 6 — Figure S5. Recombination rates in cM/Mb for each ordered Hmel2 chromosome. [file EVL3-1-138-s006.pdf]

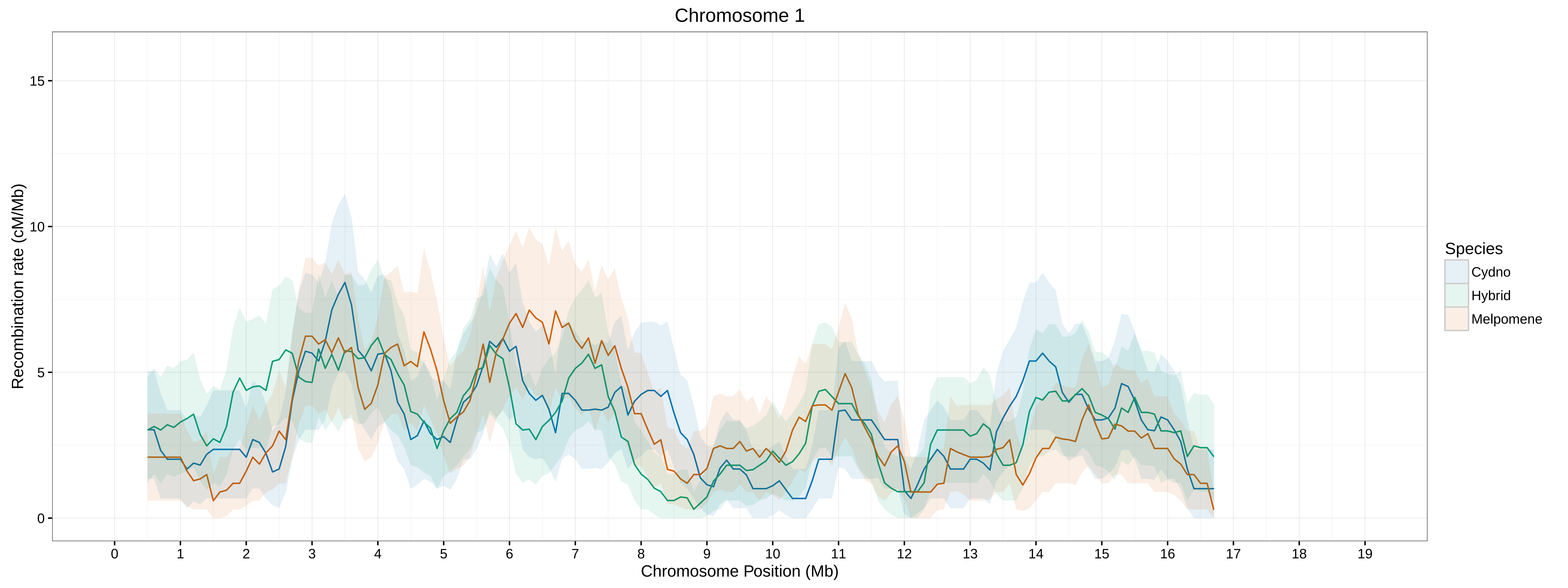

Chromosome 1
Recombination rate (cM/Mb)
0
5
10
15
0
1
2
3
4
5
6
7
8
9
10
11
12
13
14
15
16
17
18
19
Chromosome Position (Mb)
Species
Cydno
Hybrid
Melpomene

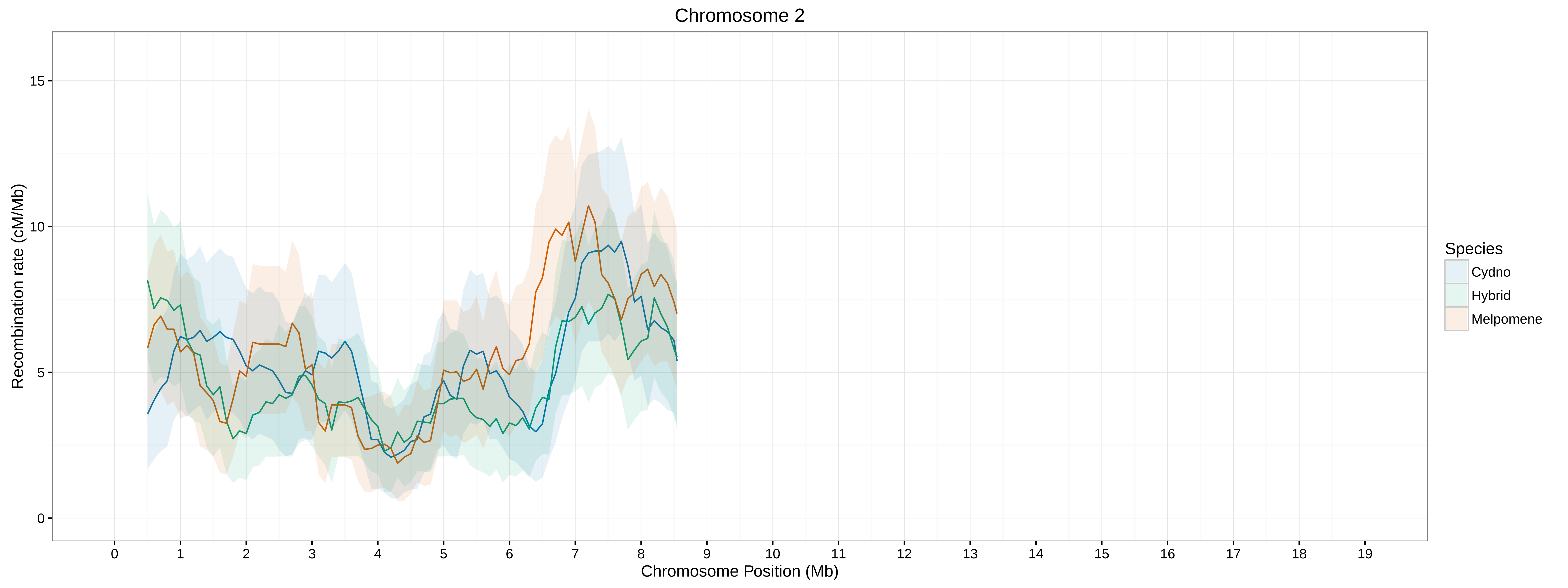

Chromosome 2
Recombination rate (cM/Mb)
0
5
10
15
0
1
2
3
4
5
6
7
8
9
10
11
12
13
14
15
16
17
18
19
Chromosome Position (Mb)
Species
Cydno
Hybrid
Melpomene

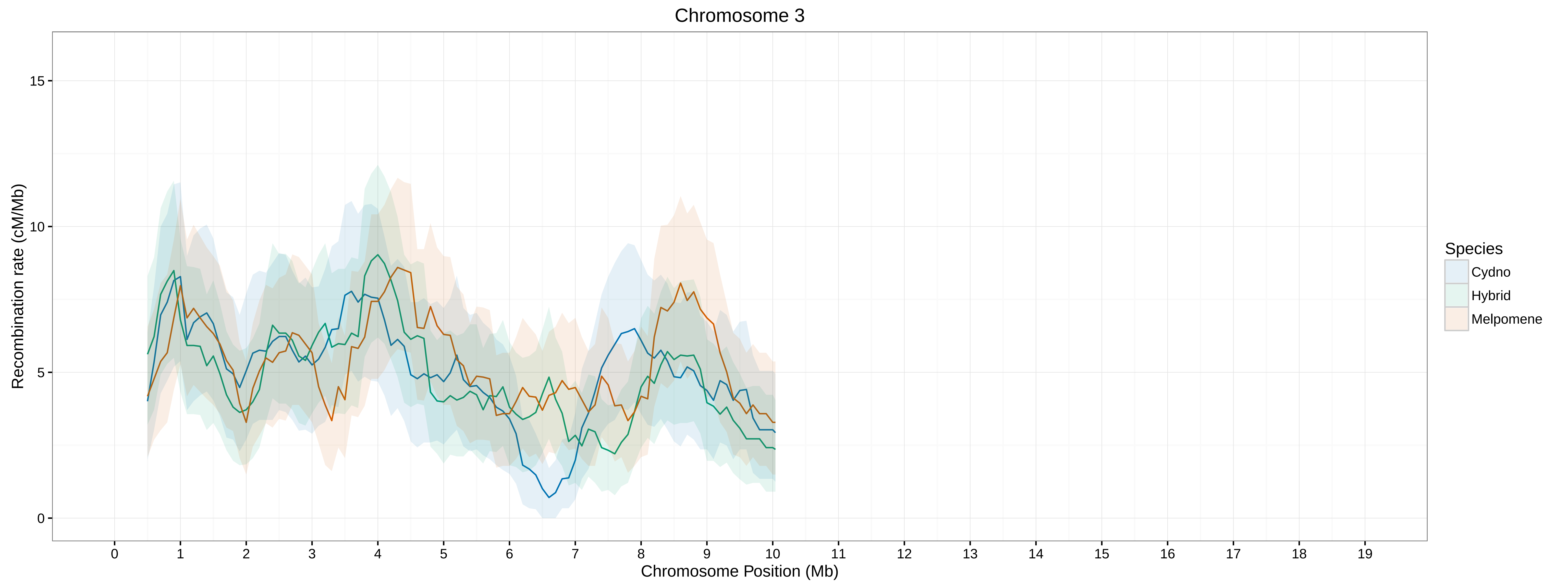

Chromosome 3
Recombination rate (cM/Mb)
0
5
10
15
Chromosome Position (Mb)
0
1
2
3
4
5
6
7
8
9
10
11
12
13
14
15
16
17
18
19
Species
Cydno
Hybrid
Melpomene

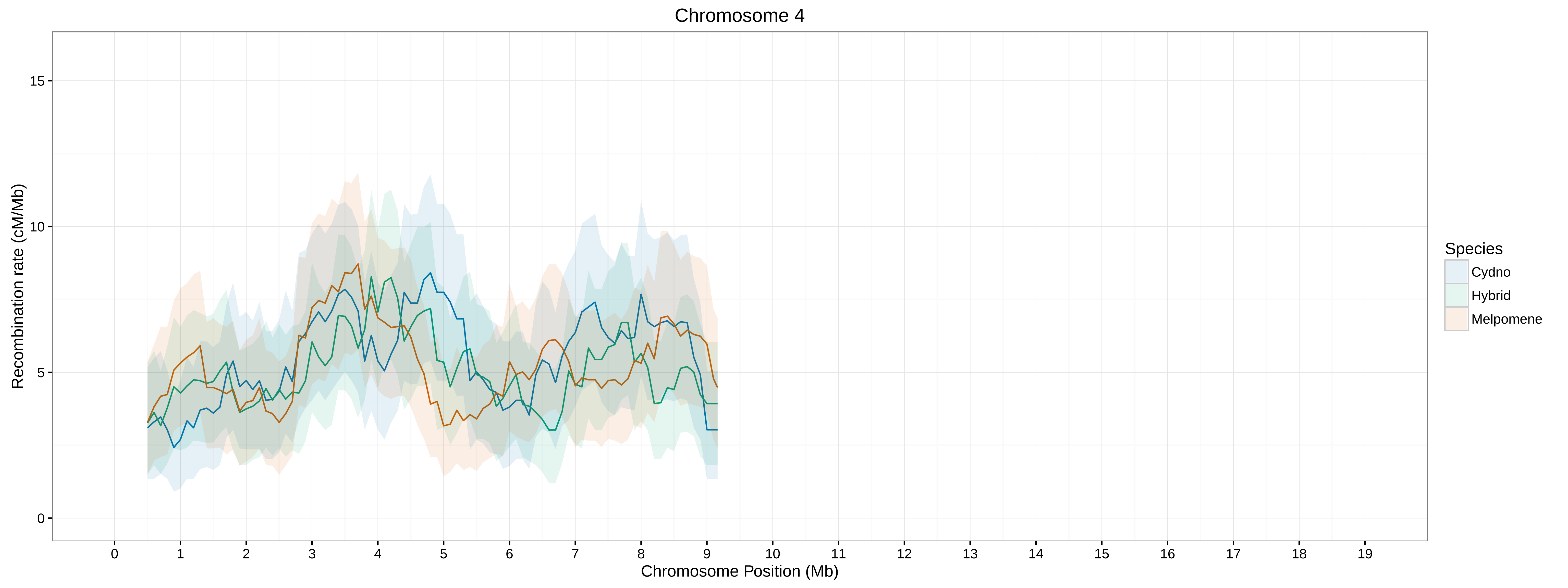
Chromosome 4
Recombination rate (cM/Mb)
15
10
5
0
0
1
2
3
4
5
6
7
8
9
10
11
12
13
14
15
16
17
18
19
Chromosome Position (Mb)
Species
Cydno
Hybrid
Melpomene

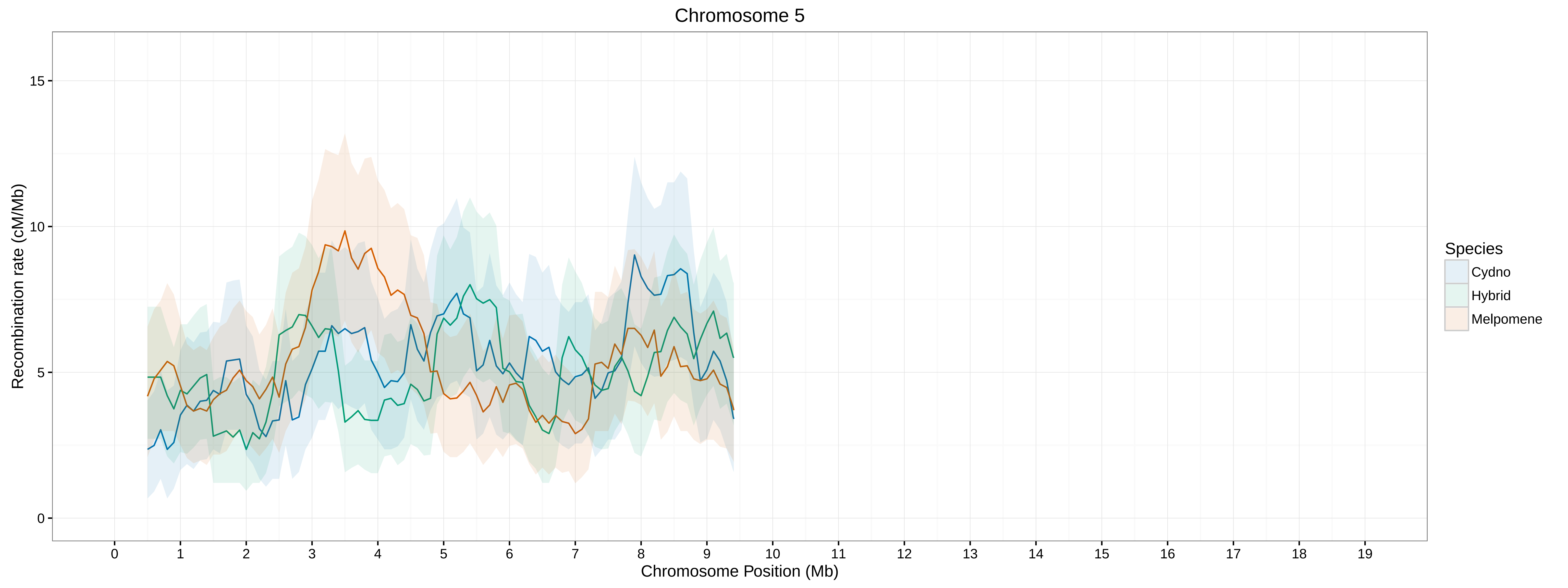

Chromosome 5
Recombination rate (cM/Mb)
0
5
10
15
Chromosome Position (Mb)
0
1
2
3
4
5
6
7
8
9
10
11
12
13
14
15
16
17
18
19
Species
Cydno
Hybrid
Melpomene

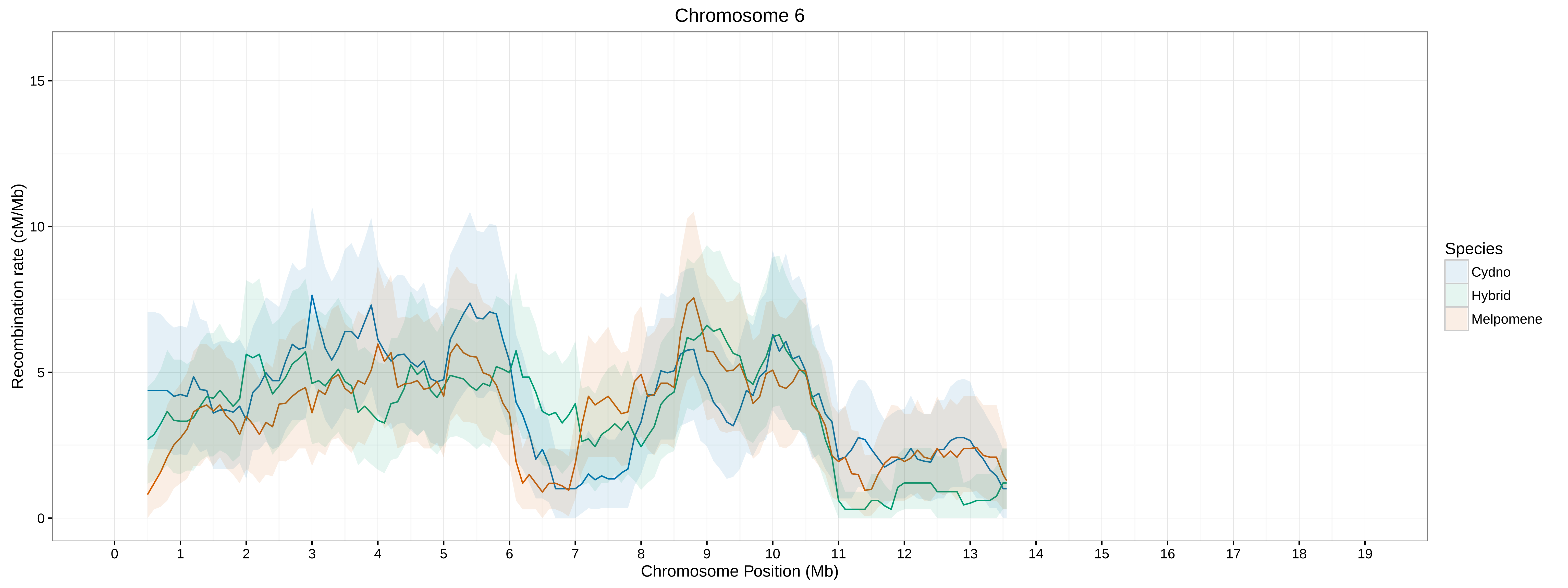
Chromosome 6
Recombination rate (cM/Mb)
0
5
10
15
0
1
2
3
4
5
6
7
8
9
10
11
12
13
14
15
16
17
18
19
Chromosome Position (Mb)
Species
Cydno
Hybrid
Melpomene

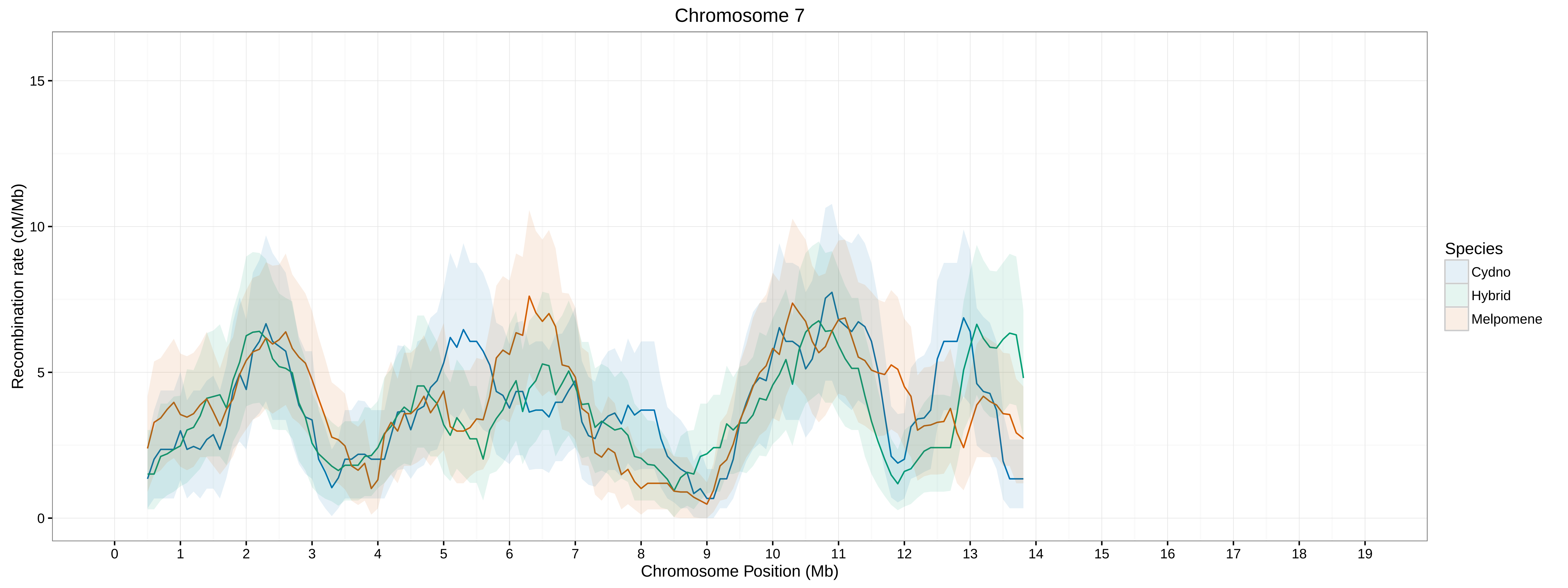
Chromosome 7
Recombination rate (cM/Mb)
0
5
10
15
Chromosome Position (Mb)
0
1
2
3
4
5
6
7
8
9
10
11
12
13
14
15
16
17
18
19
Species
Cydno
Hybrid
Melpomene

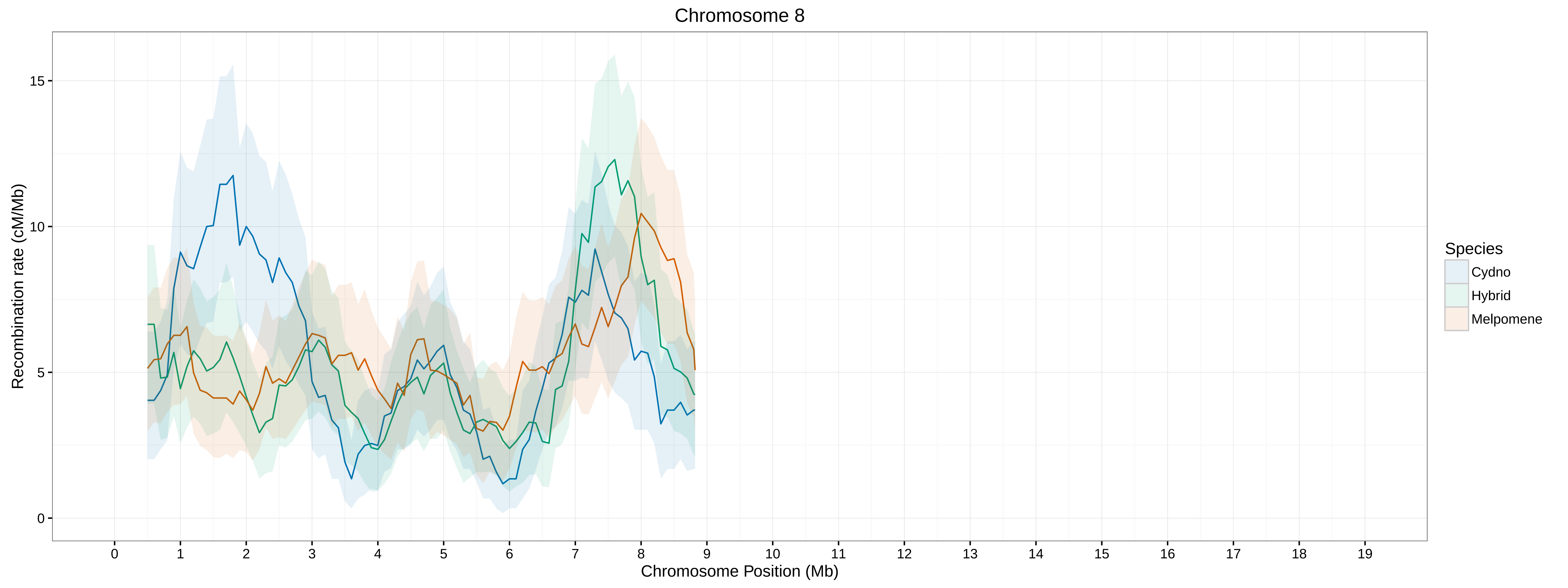

Chromosome 8
Recombination rate (cM/Mb)
15
10
5
0
0
1
2
3
4
5
6
7
8
9
10
11
12
13
14
15
16
17
18
19
Chromosome Position (Mb)
Species
Cydno
Hybrid
Melpomene

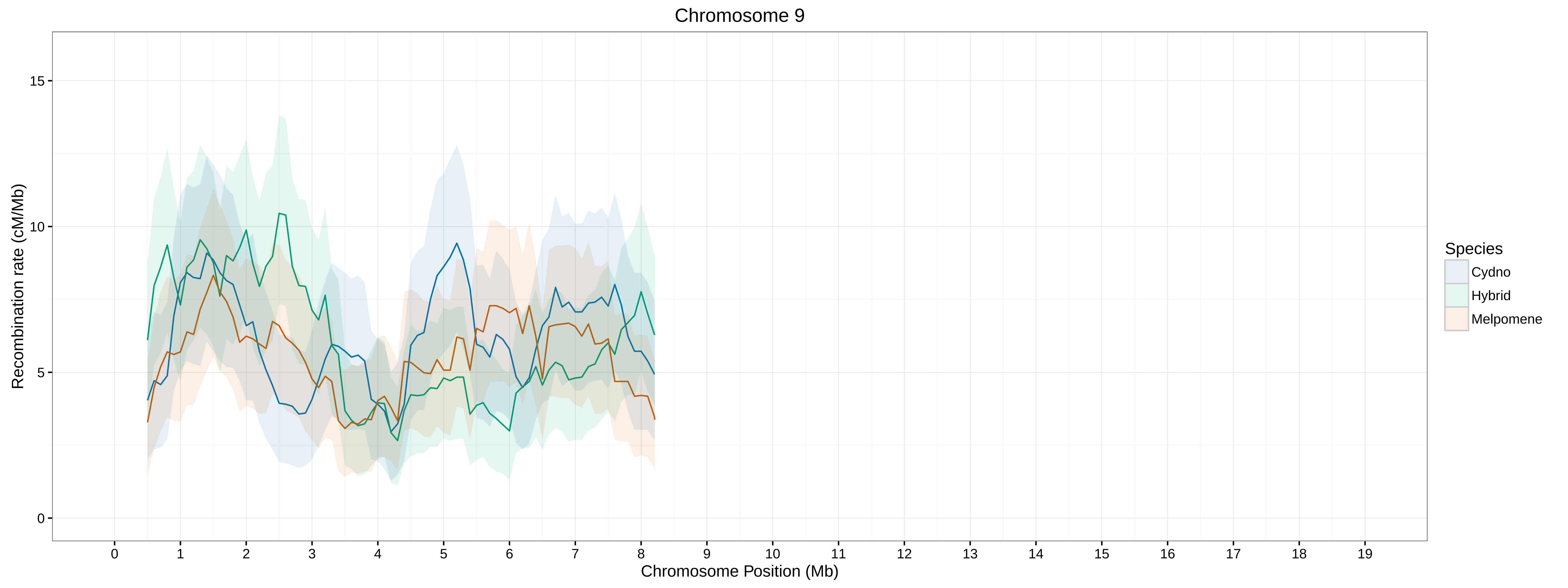

Chromosome 9
Recombination rate (cM/Mb)
0
5
10
15
0
1
2
3
4
5
6
7
8
9
10
11
12
13
14
15
16
17
18
19
Chromosome Position (Mb)
Species
Cydno
Hybrid
Melpomene

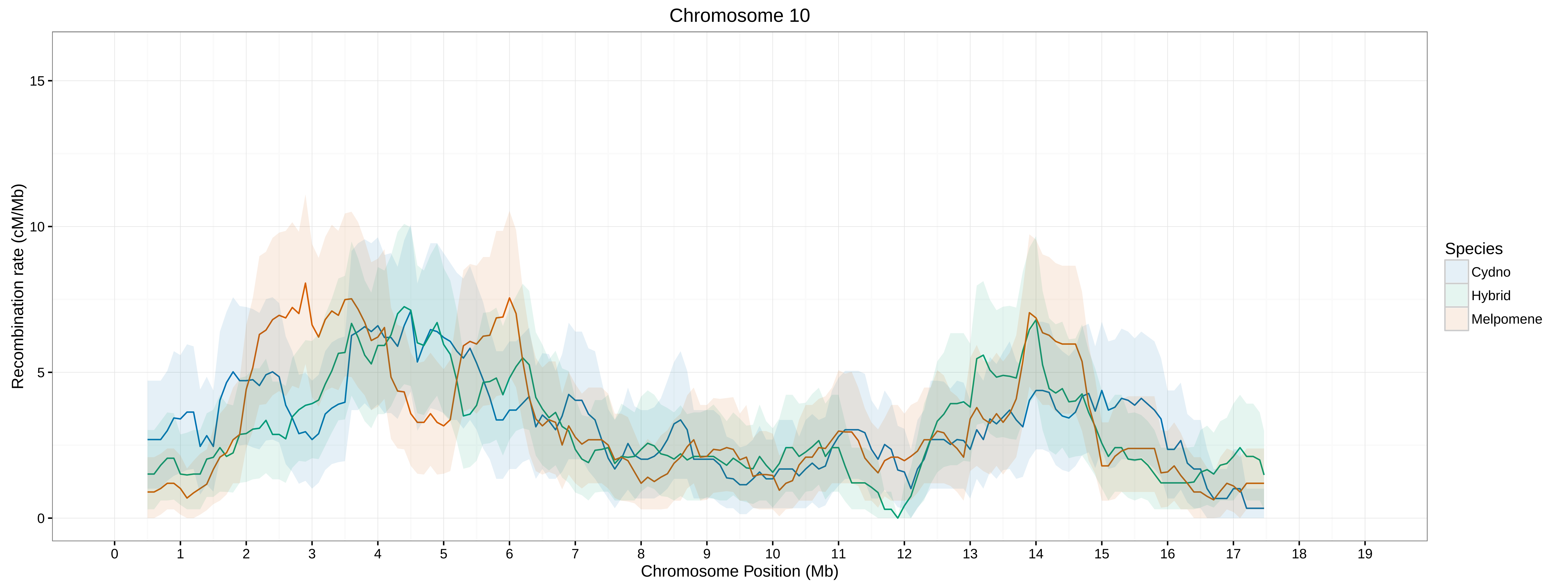

Chromosome 10
Recombination rate (cM/Mb)
0
5
10
15
0
1
2
3
4
5
6
7
8
9
10
11
12
13
14
15
16
17
18
19
Chromosome Position (Mb)
Species
Cydno
Hybrid
Melpomene

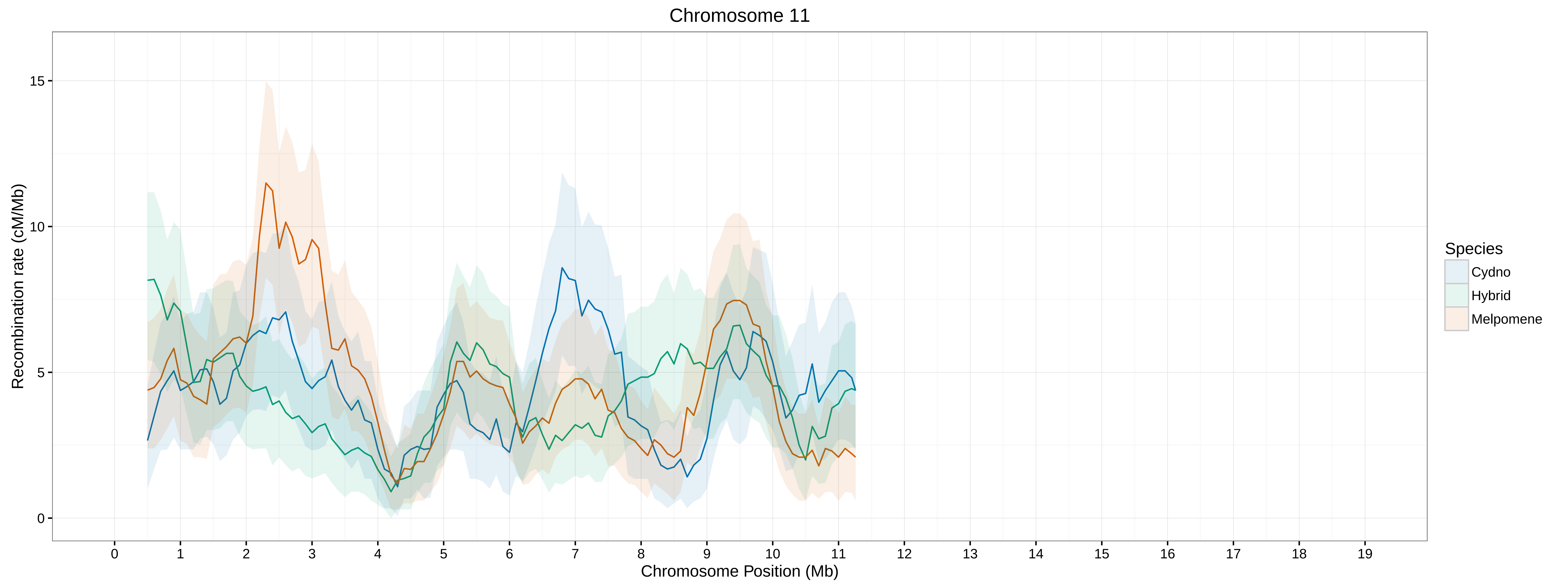

Chromosome 11
Recombination rate (cM/Mb)
15
10
5
0
0
1
2
3
4
5
6
7
8
9
10
11
12
13
14
15
16
17
18
19
Chromosome Position (Mb)
Species
Cydno
Hybrid
Melpomene

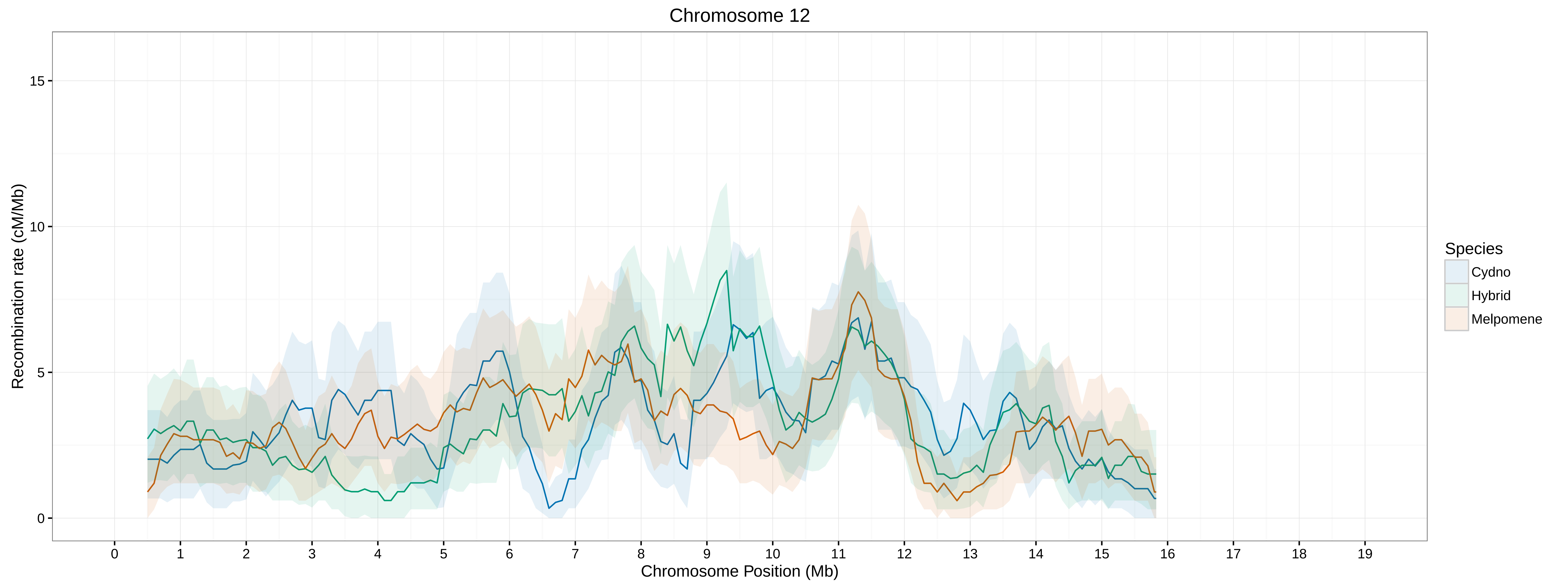

Chromosome 12
Recombination rate (cM/Mb)
0
5
10
15
0
1
2
3
4
5
6
7
8
9
10
11
12
13
14
15
16
17
18
19
Chromosome Position (Mb)
Species
Cydno
Hybrid
Melpomene

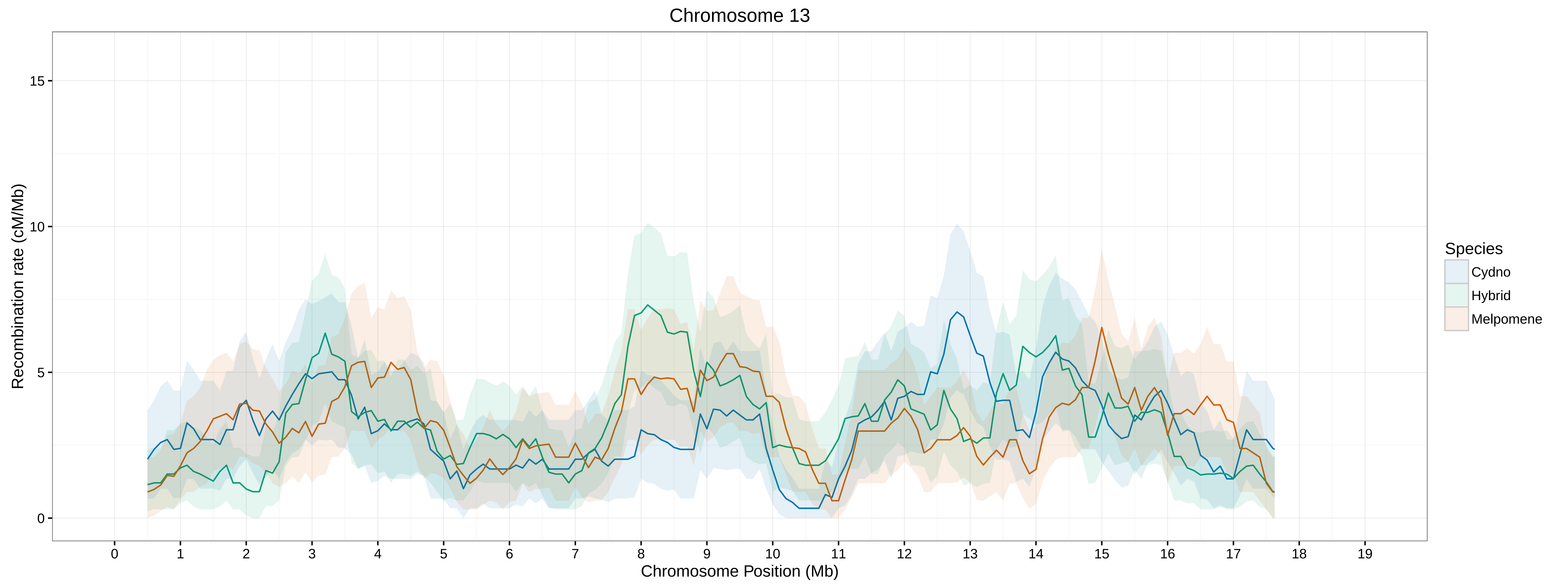
Chromosome 13
Recombination rate (cM/Mb)
0
5
10
15
0
1
2
3
4
5
6
7
8
9
10
11
12
13
14
15
16
17
18
19
Chromosome Position (Mb)
Species
Cydno
Hybrid
Melpomene

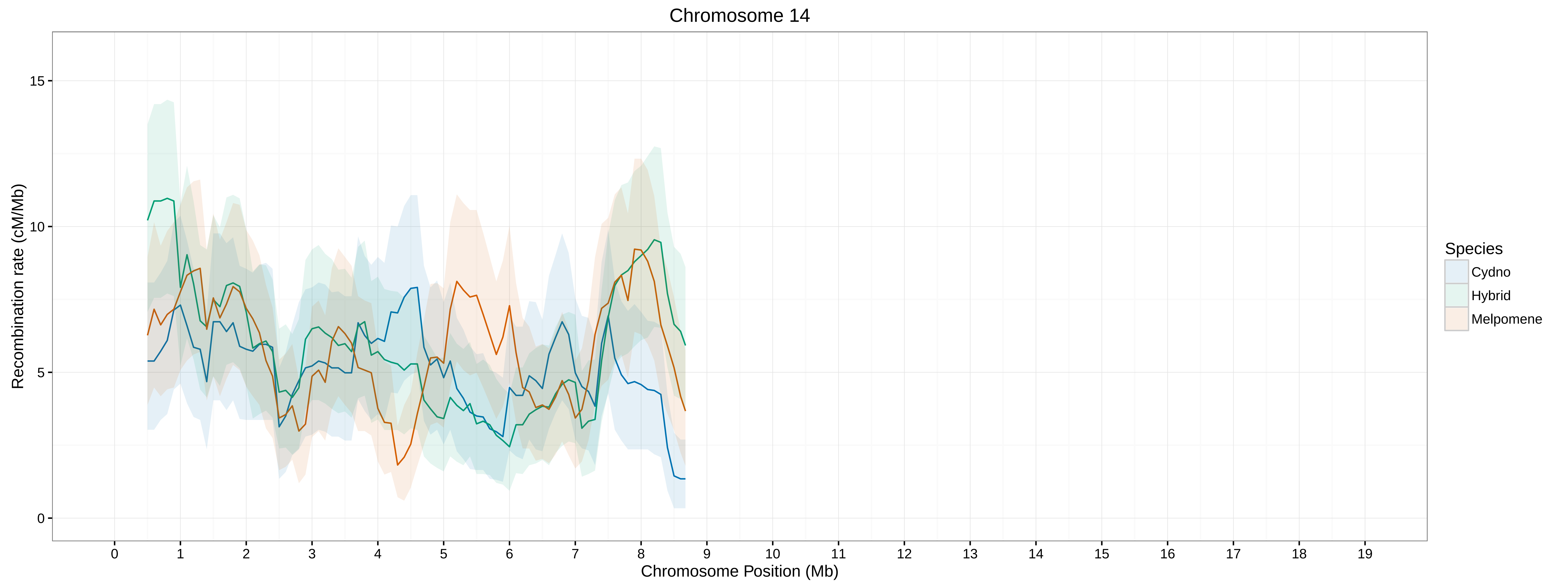

Chromosome 14
Recombination rate (cM/Mb)
0
5
10
15
Chromosome Position (Mb)
0
1
2
3
4
5
6
7
8
9
10
11
12
13
14
15
16
17
18
19
Species
Cydno
Hybrid
Melpomene

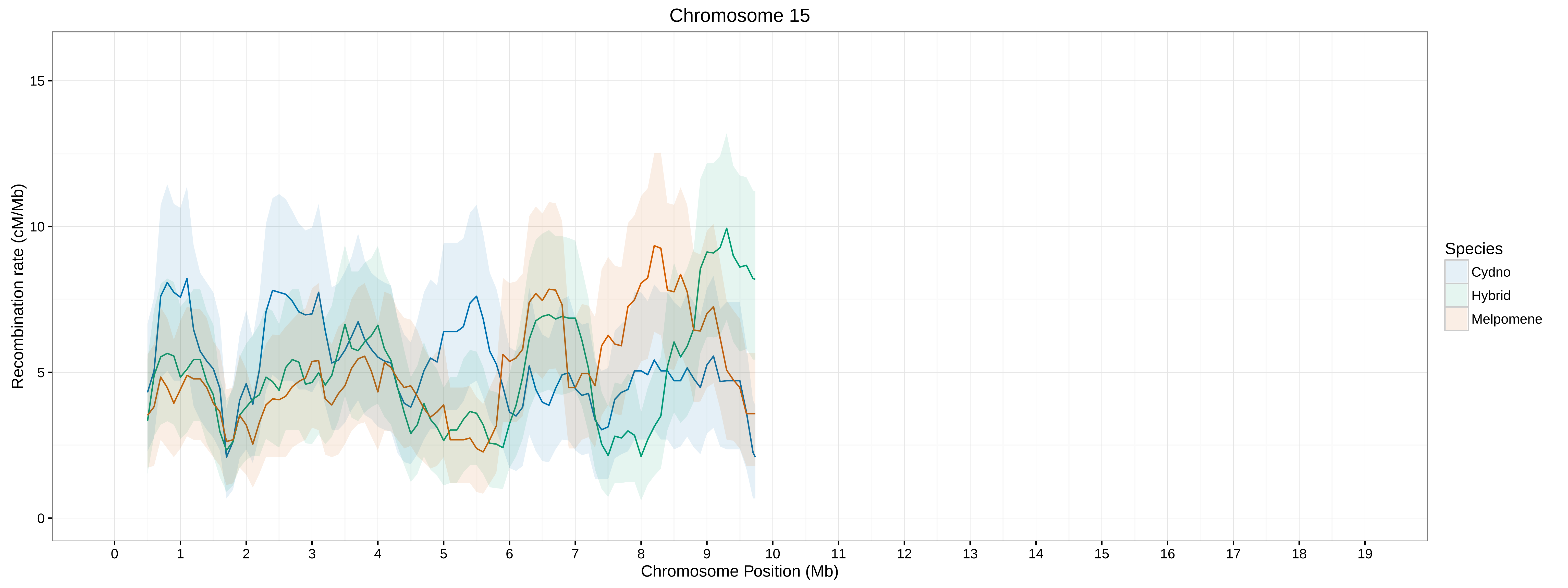

Chromosome 15
Recombination rate (cM/Mb)
0
5
10
15
0
1
2
3
4
5
6
7
8
9
10
11
12
13
14
15
16
17
18
19
Chromosome Position (Mb)
Species
Cydno
Hybrid
Melpomene

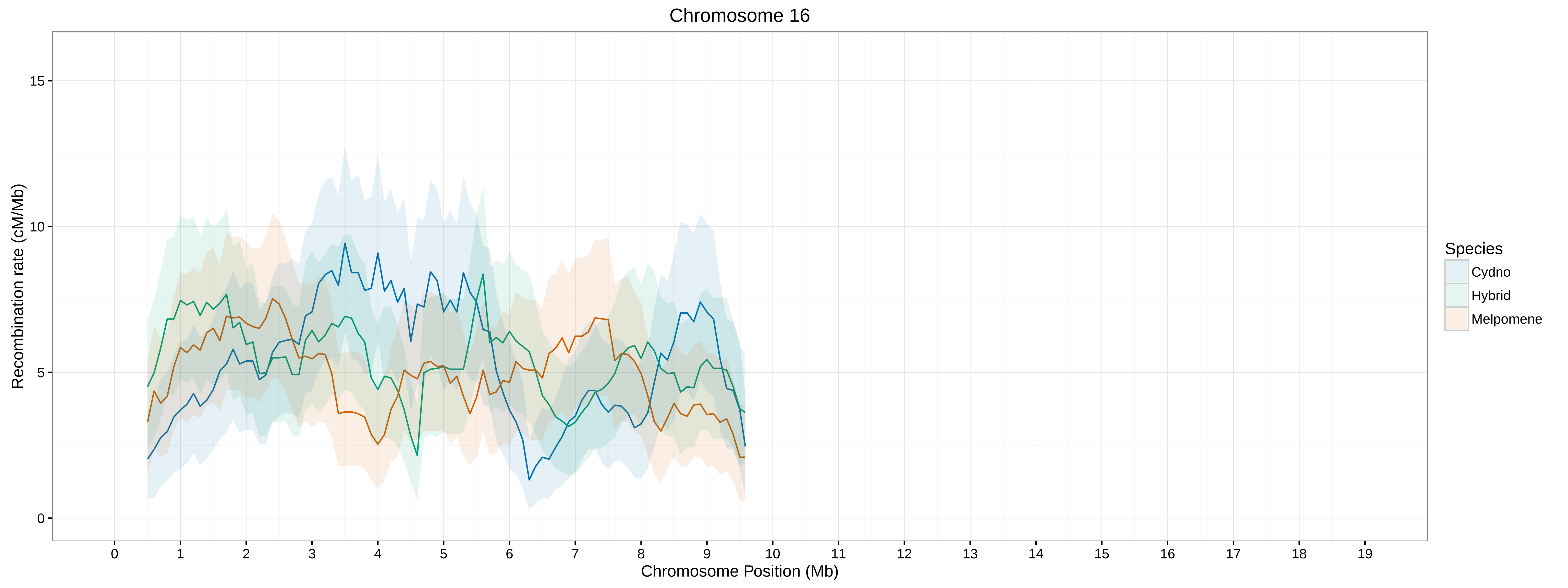

Chromosome 16
Recombination rate (cM/Mb)
0
5
10
15
Chromosome Position (Mb)
0
1
2
3
4
5
6
7
8
9
10
11
12
13
14
15
16
17
18
19
Species
Cydno
Hybrid
Melpomene

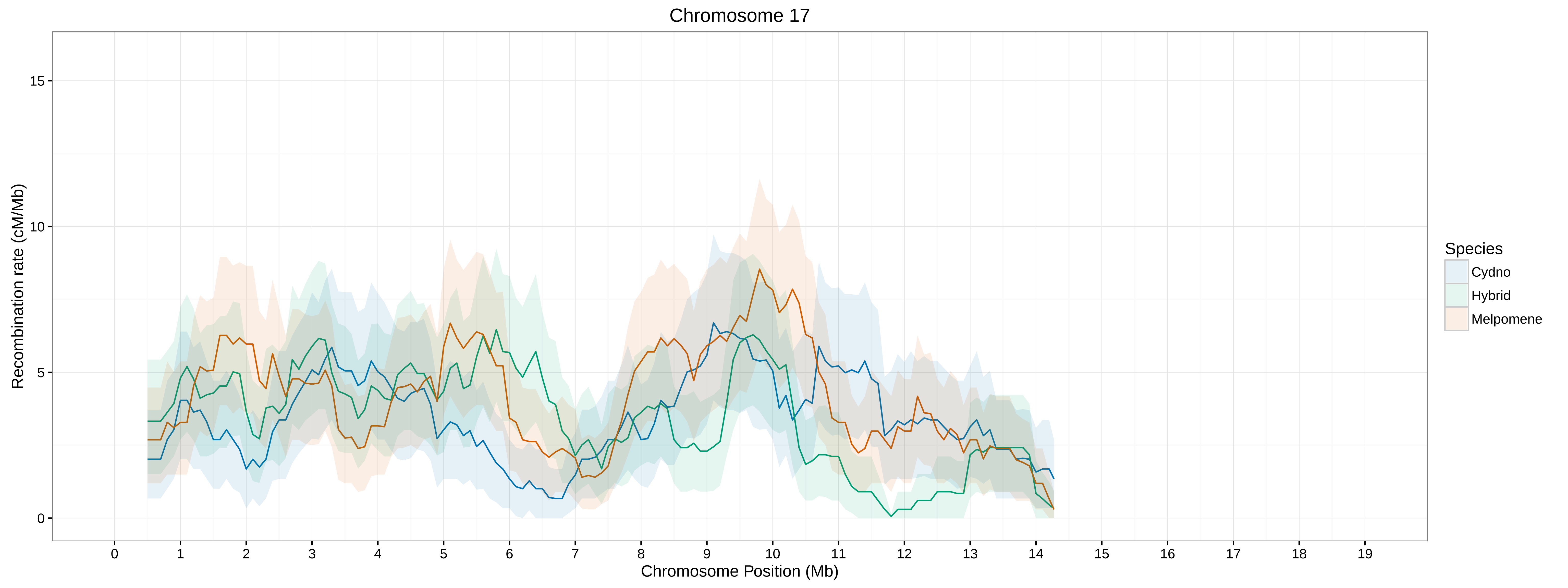

Chromosome 17
Recombination rate (cM/Mb)
0
5
10
15
0
1
2
3
4
5
6
7
8
9
10
11
12
13
14
15
16
17
18
19
Chromosome Position (Mb)
Species
Cydno
Hybrid
Melpomene

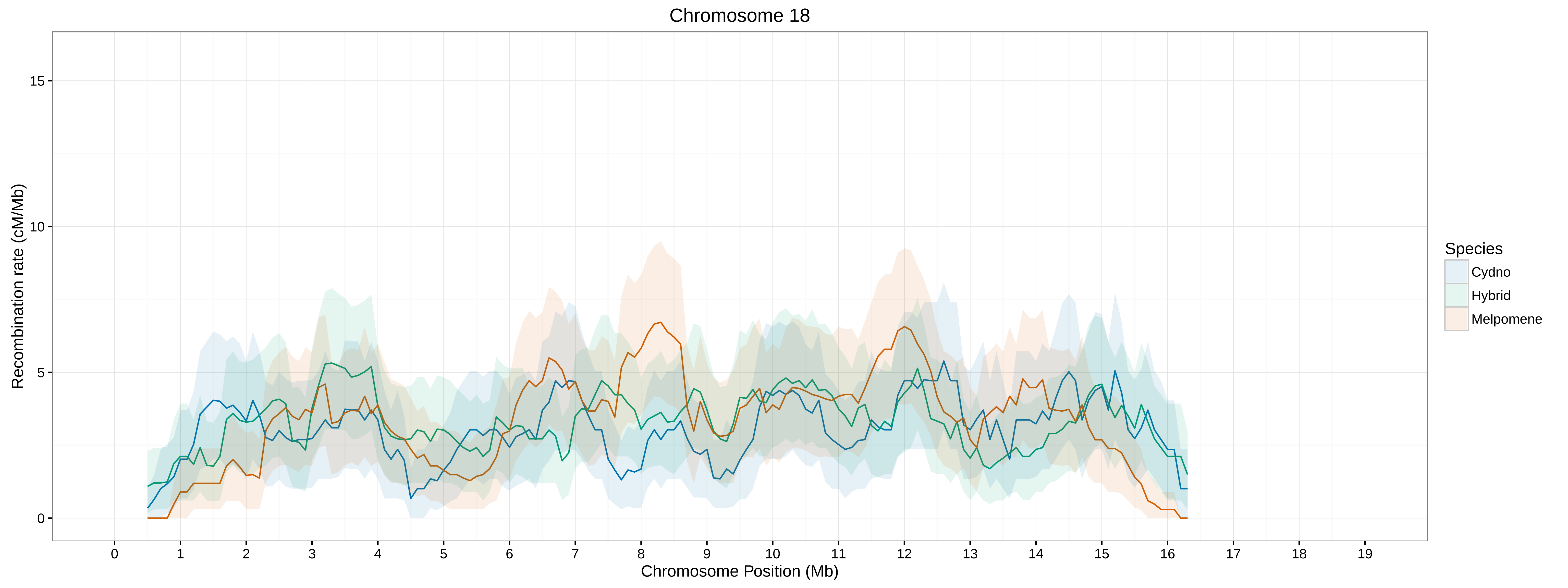

Chromosome 18
Recombination rate (cM/Mb)
0
5
10
15
0
1
2
3
4
5
6
7
8
9
10
11
12
13
14
15
16
17
18
19
Chromosome Position (Mb)
Species
Cydno
Hybrid
Melpomene

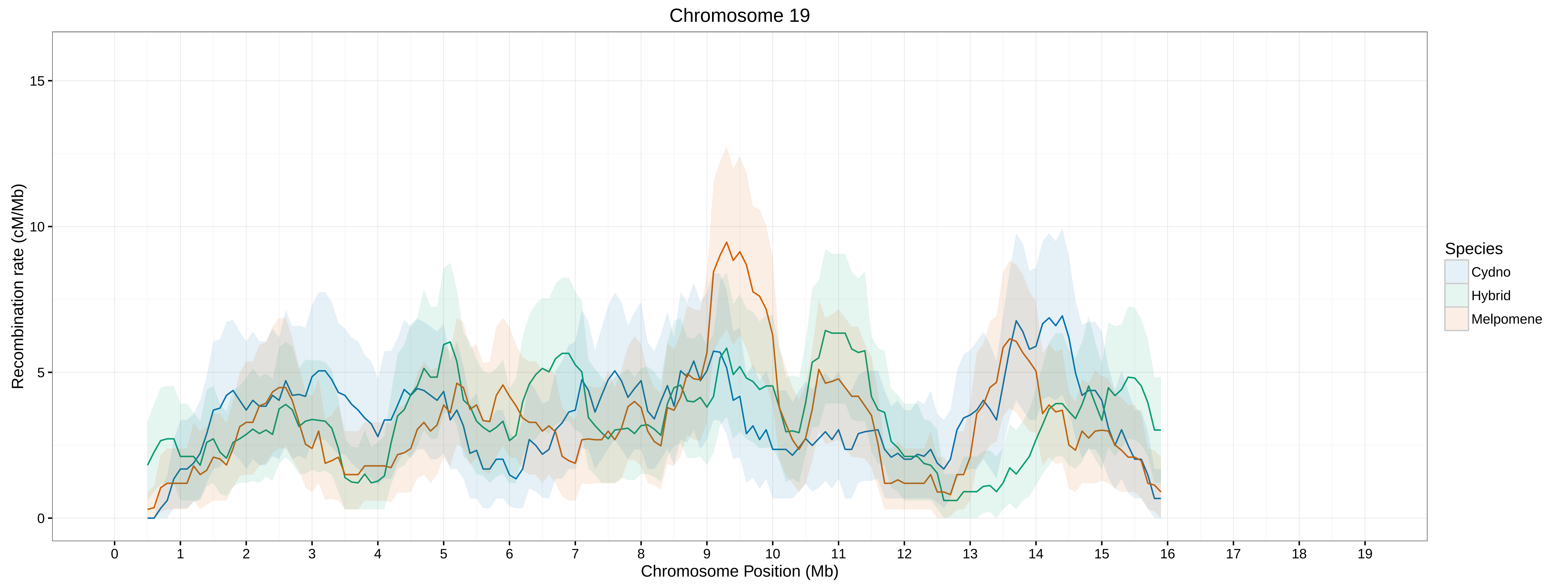

Chromosome 19
Recombination rate (cM/Mb)
0
5
10
15
0
1
2
3
4
5
6
7
8
9
10
11
12
13
14
15
16
17
18
19
Chromosome Position (Mb)
Species
Cydno
Hybrid
Melpomene

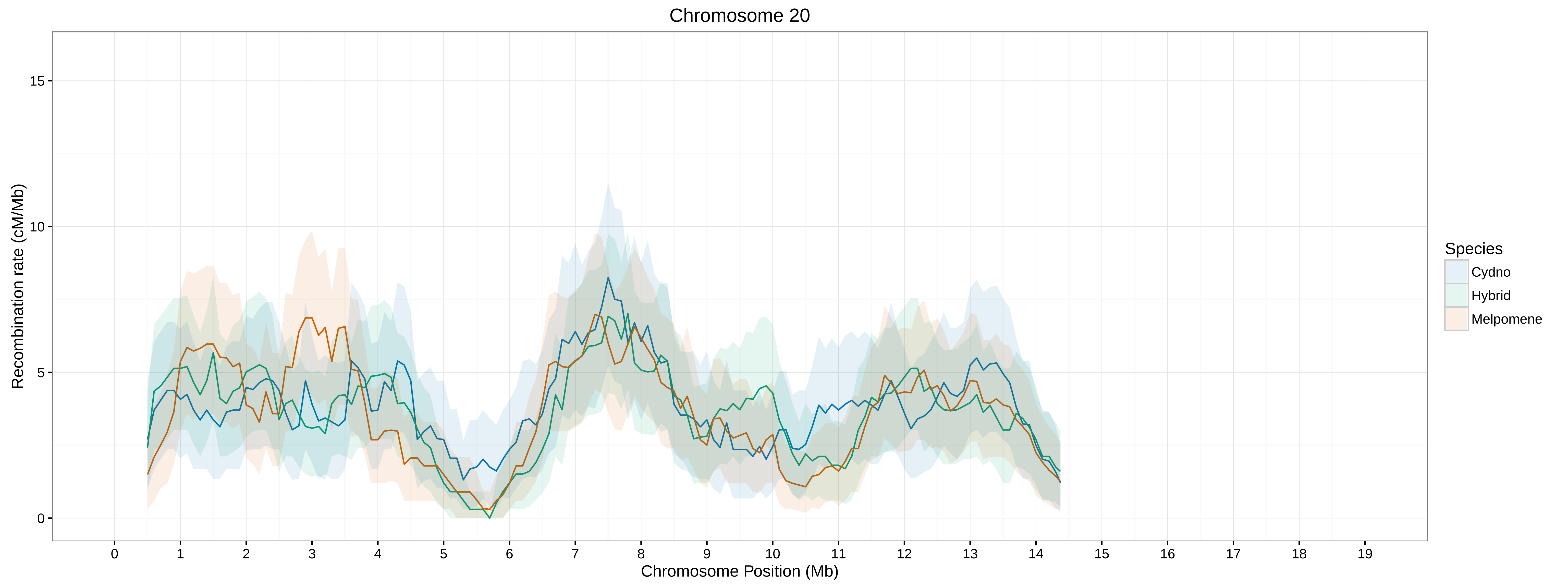

Chromosome 20
Recombination rate (cM/Mb)
0
5
10
15
Chromosome Position (Mb)
0
1
2
3
4
5
6
7
8
9
10
11
12
13
14
15
16
17
18
19
Species
Cydno
Hybrid
Melpomene

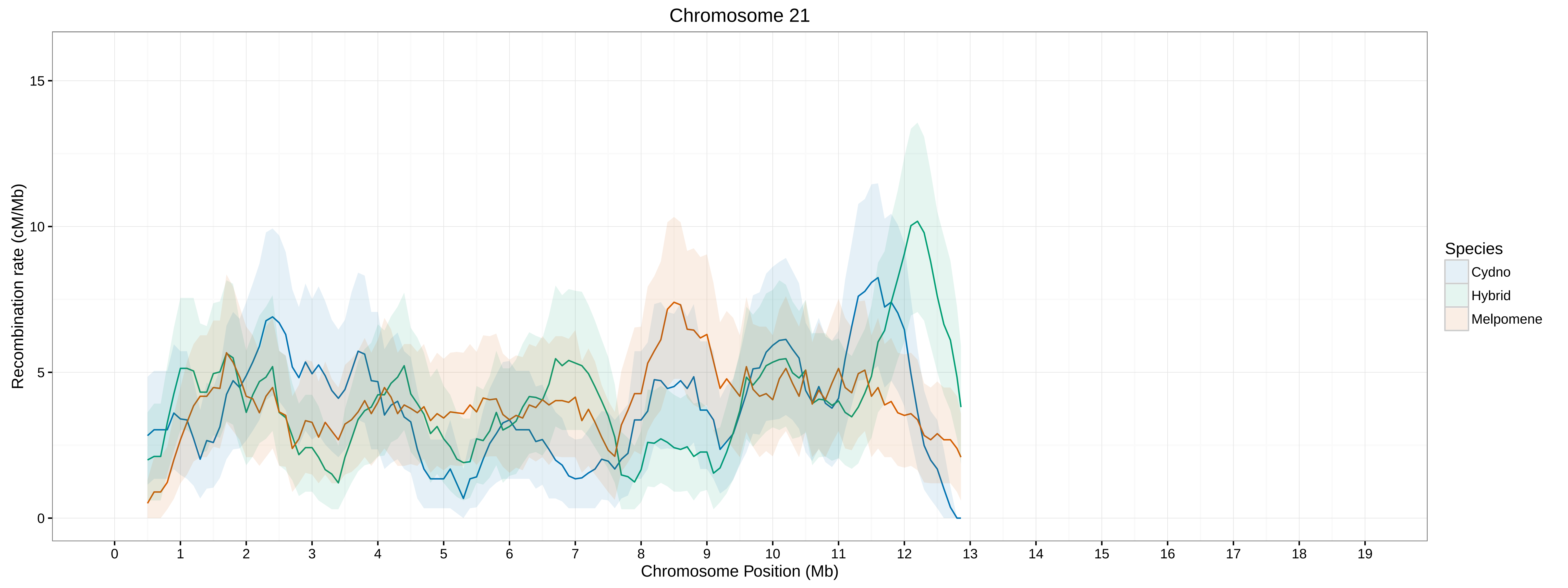

Chromosome 21
Recombination rate (cM/Mb)
0
5
10
15
Chromosome Position (Mb)
0
1
2
3
4
5
6
7
8
9
10
11
12
13
14
15
16
17
18
19
Species
Cydno
Hybrid
Melpomene
