# Supplementary material for: No evidence for maintenance of a sympatric Heliconius species barrier by chromosomal inversions
Source: Evol Lett. 2017 Jun 14;1(3):138–54. doi: 10.1002/evl3.12 (PMC6122123; doi:10.1002/evl3.12)
Supplement: Supplementary file 7 — Figure S6. Gaps in linkage maps where lack of precise recombination data cannot rule out presence of inversions. [file EVL3-1-138-s007.pdf]

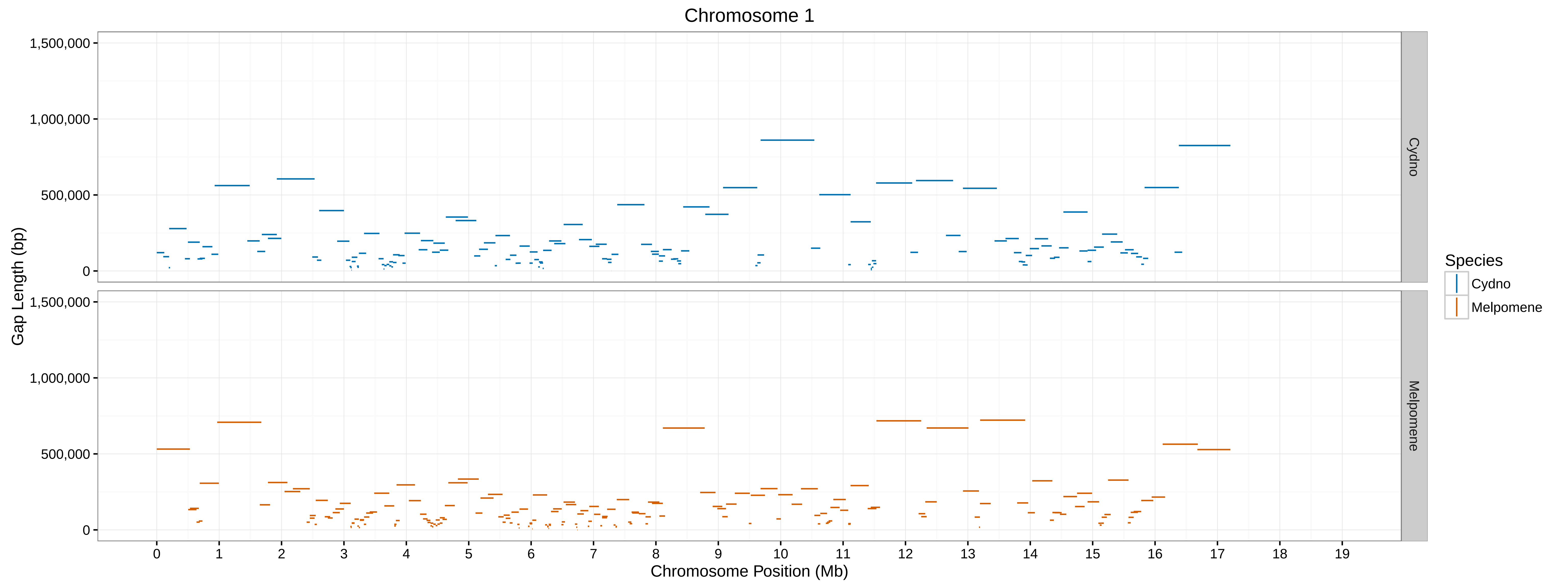

Chromosome 1
1,500,000
1,000,000
500,000
0
Cydno
Melpomene
Gap Length (bp)
Species
Cydno
Melpomene
0
1
2
3
4
5
6
7
8
9
10
11
12
13
14
15
16
17
18
19
Chromosome Position (Mb)

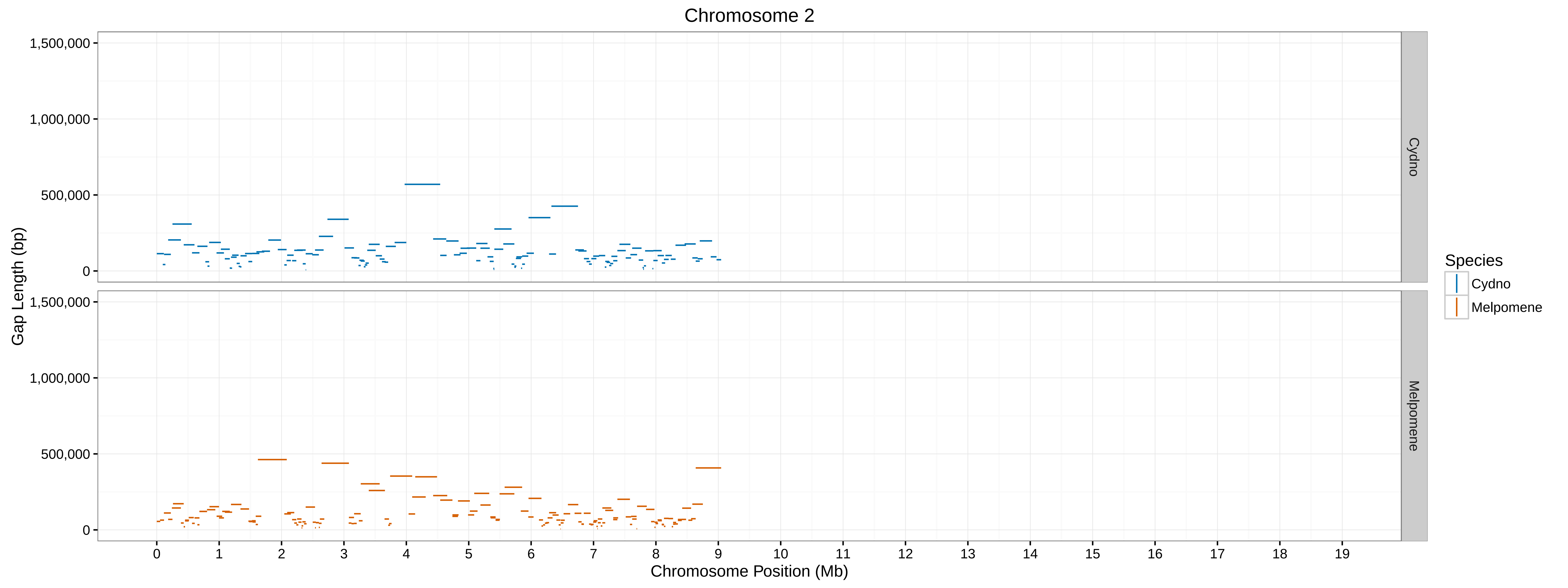

Chromosome 2
Gap Length (bp)
1,500,000
1,000,000
500,000
0
Cydno
Melpomene
0
1
2
3
4
5
6
7
8
9
10
11
12
13
14
15
16
17
18
19
Chromosome Position (Mb)
Species
Cydno
Melpomene

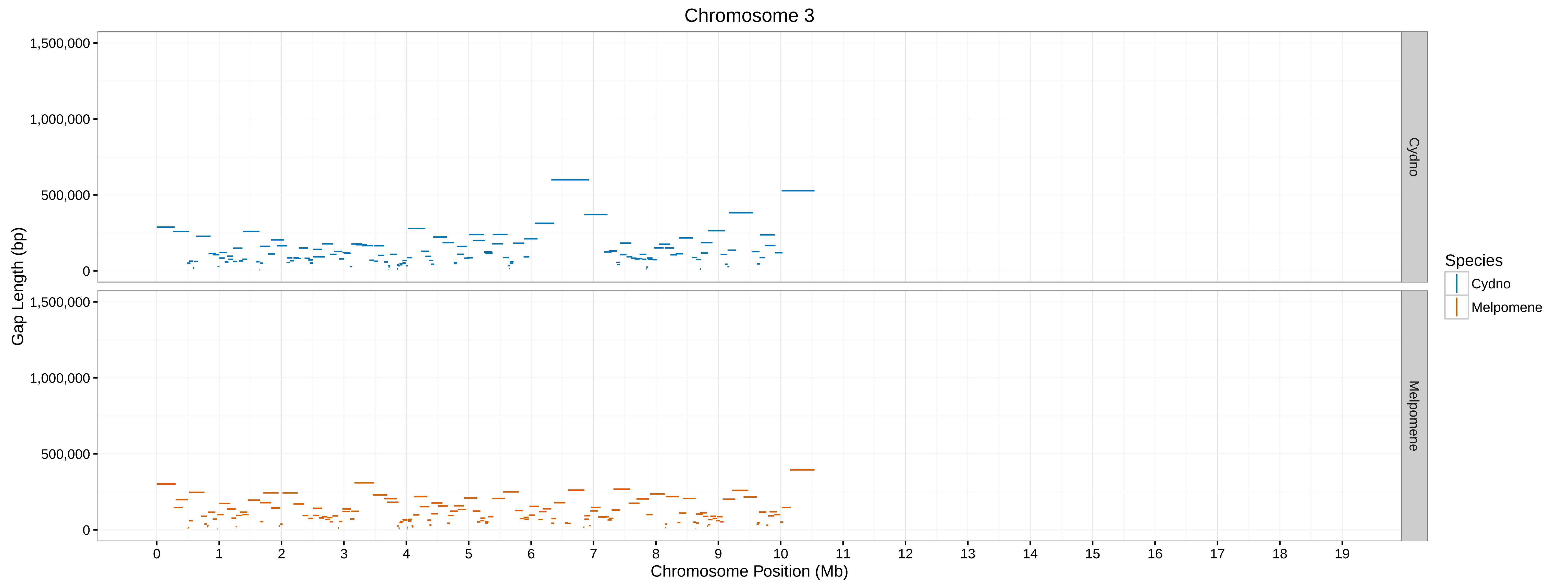

Chromosome 3
Gap Length (bp)
1,500,000
1,000,000
500,000
0
Cydno
Melpomene
0
1
2
3
4
5
6
7
8
9
10
11
12
13
14
15
16
17
18
19
Chromosome Position (Mb)
Species
Cydno
Melpomene

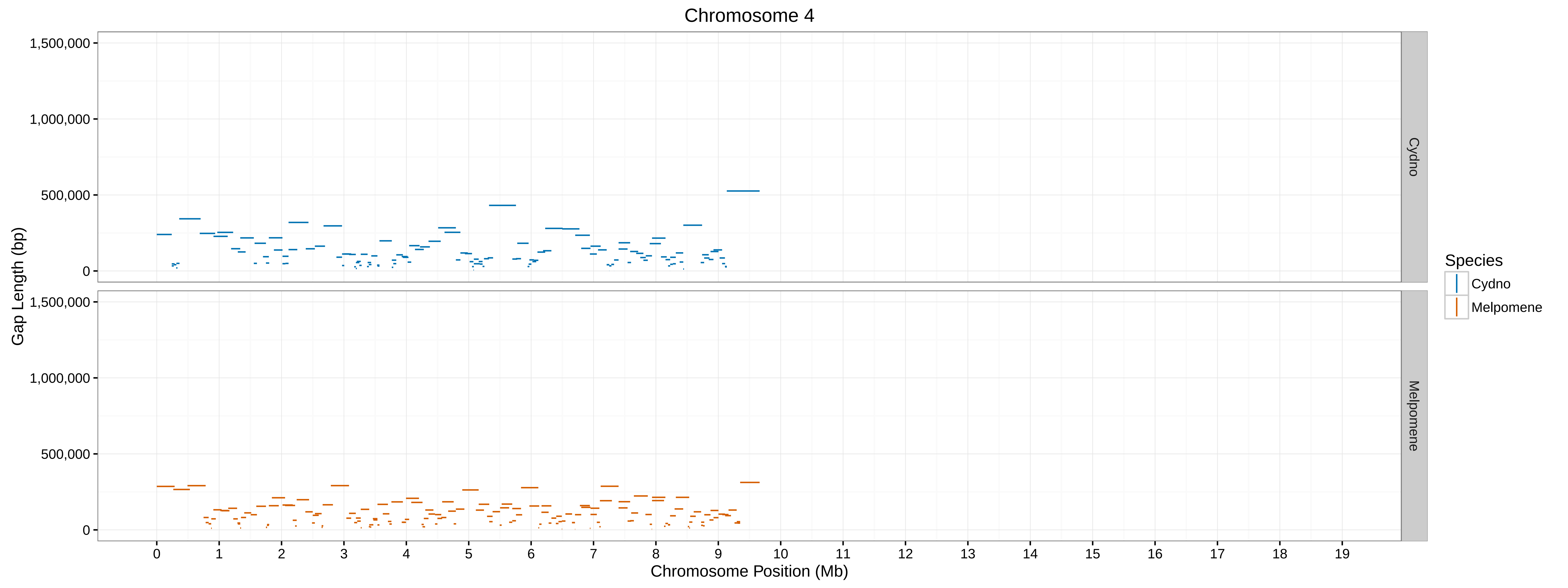

Chromosome 4
1,500,000
1,000,000
500,000
0
Gap Length (bp)
Cydno
Melpomene
0
1
2
3
4
5
6
7
8
9
10
11
12
13
14
15
16
17
18
19
Chromosome Position (Mb)
Species
Cydno
Melpomene

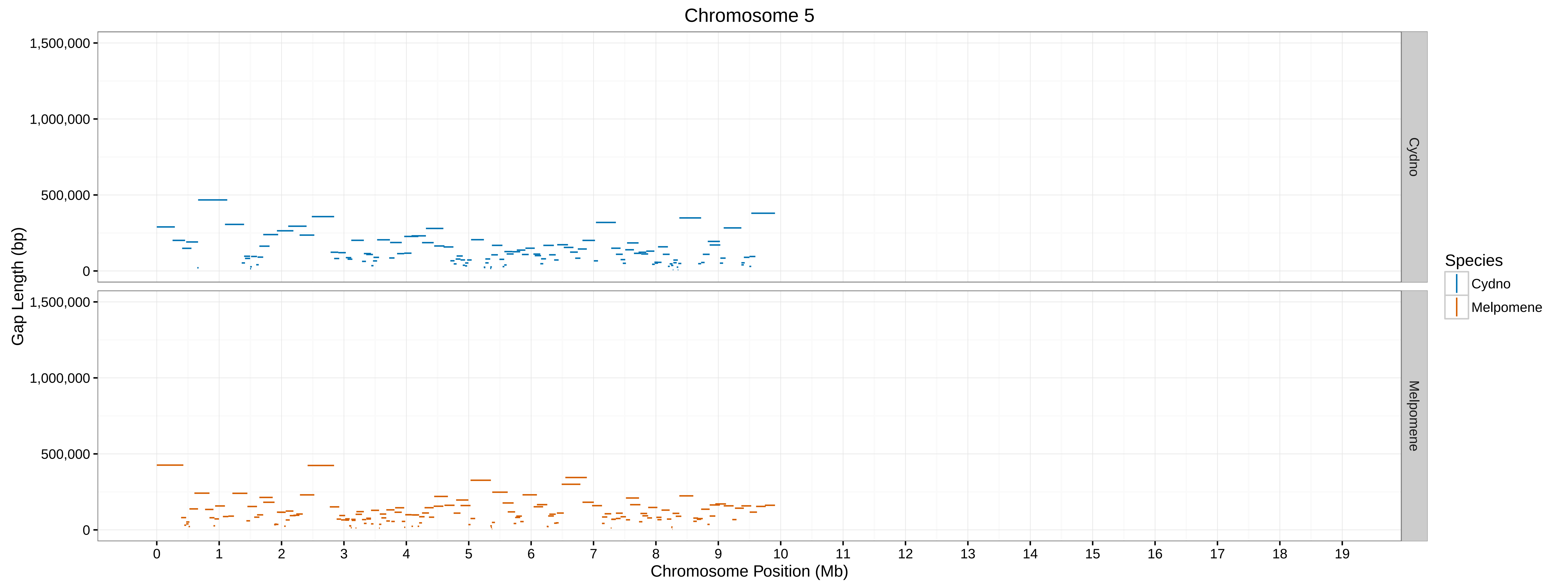
Chromosome 5
Gap Length (bp)
1,500,000
1,000,000
500,000
0
Cydno
Melpomene
Chromosome Position (Mb)
0
1
2
3
4
5
6
7
8
9
10
11
12
13
14
15
16
17
18
19
Species
Cydno
Melpomene

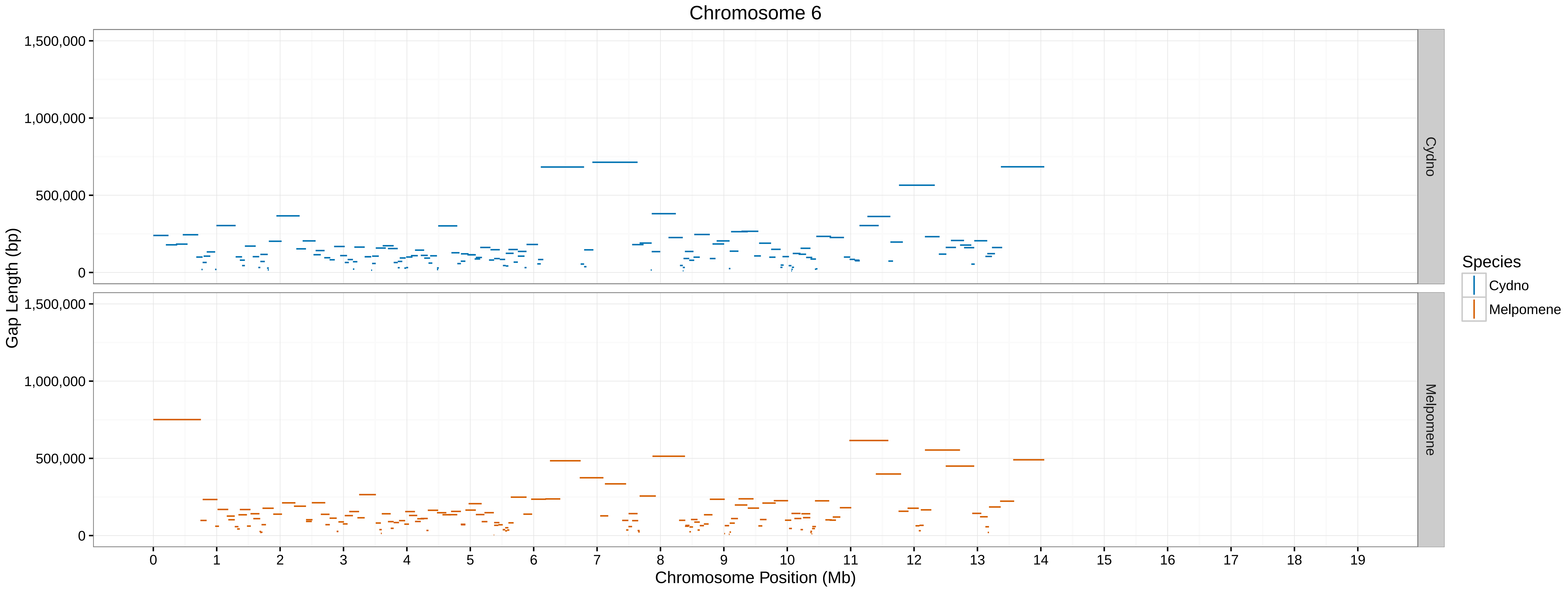

Chromosome 6
Gap Length (bp)
1,500,000
1,000,000
500,000
0
Cydno
Melpomene
Species
Cydno
Melpomene
0
1
2
3
4
5
6
7
8
9
10
11
12
13
14
15
16
17
18
19
Chromosome Position (Mb)

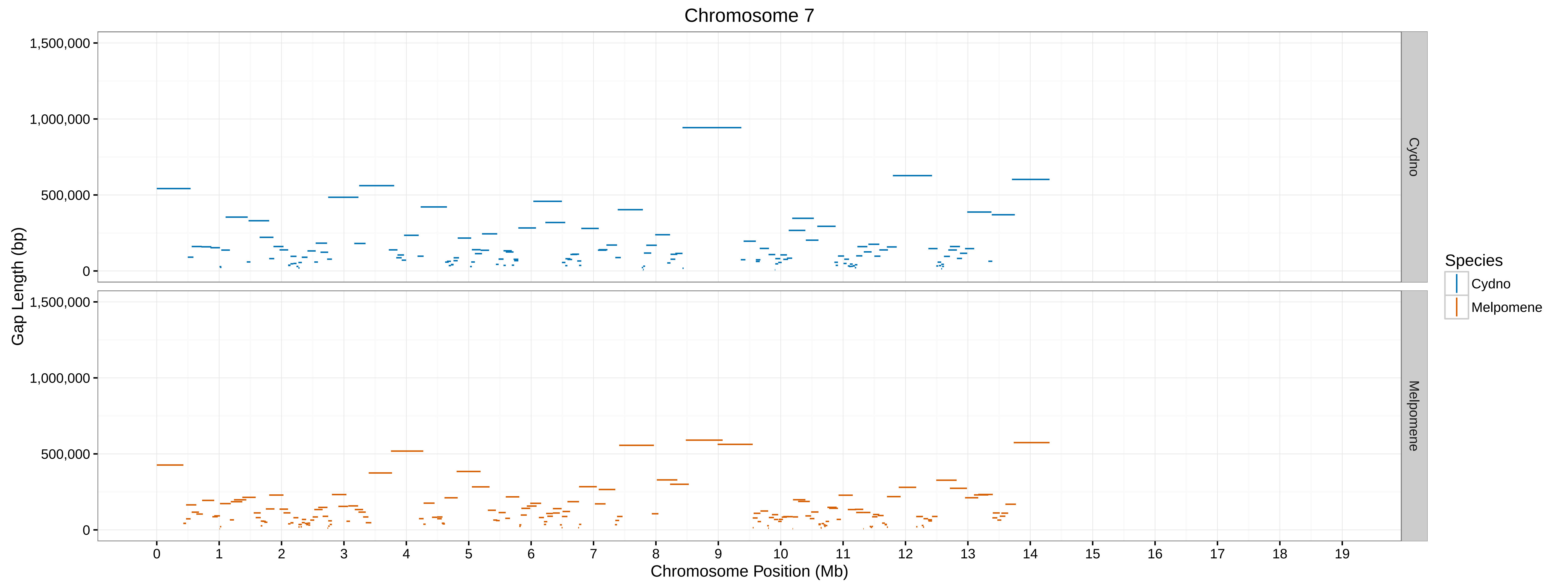

Chromosome 7
1,500,000
1,000,000
500,000
0
Gap Length (bp)
Cydno
Melpomene
0
1
2
3
4
5
6
7
8
9
10
11
12
13
14
15
16
17
18
19
Chromosome Position (Mb)
Species
Cydno
Melpomene

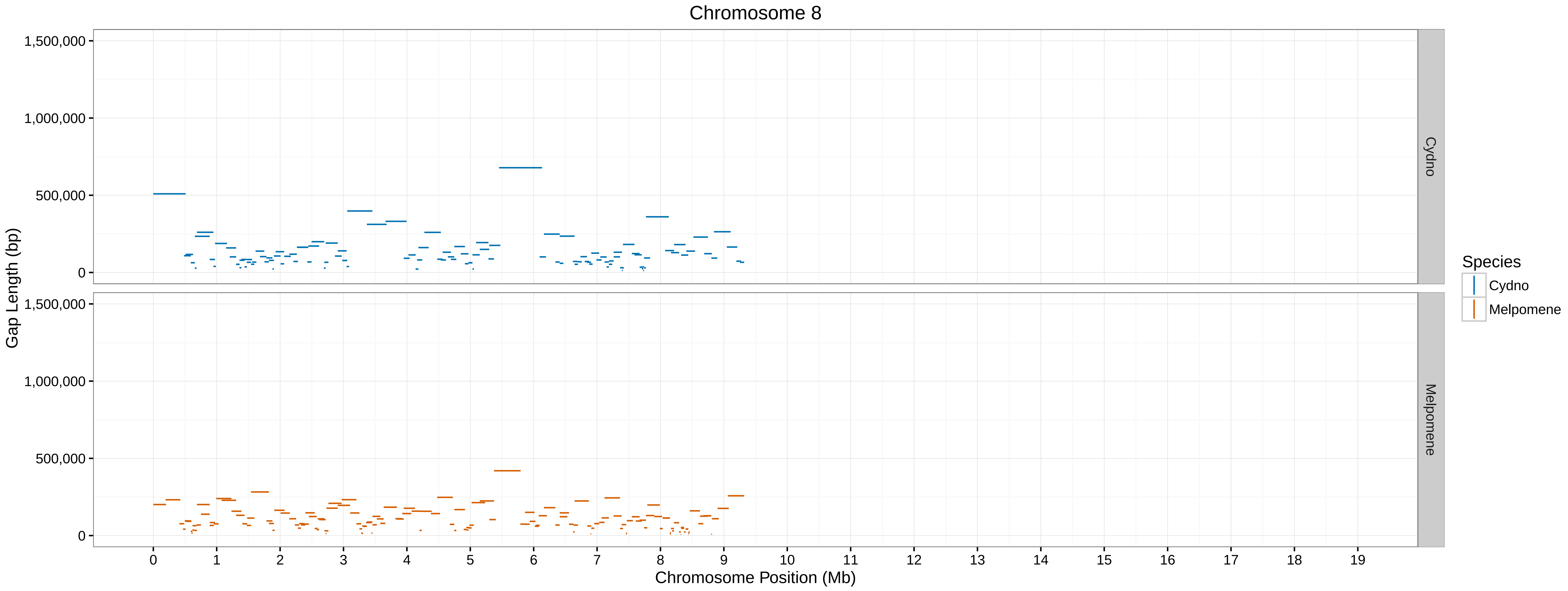

Chromosome 8
1,500,000
1,000,000
500,000
0
Gap Length (bp)
Cydno
Melpomene
0
1
2
3
4
5
6
7
8
9
10
11
12
13
14
15
16
17
18
19
Chromosome Position (Mb)
Species
Cydno
Melpomene

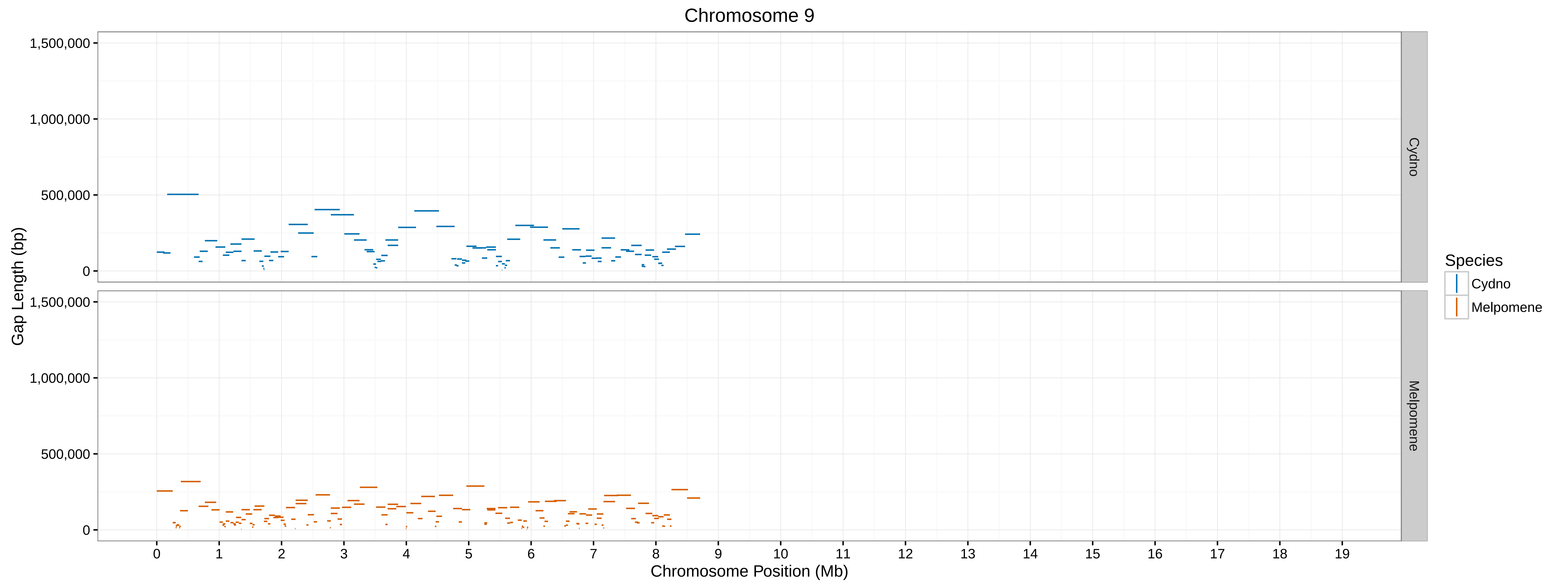

Chromosome 9
Gap Length (bp)
1,500,000
1,000,000
500,000
0
Cydno
Melpomene
Species
Cydno
Melpomene
0
1
2
3
4
5
6
7
8
9
10
11
12
13
14
15
16
17
18
19
Chromosome Position (Mb)

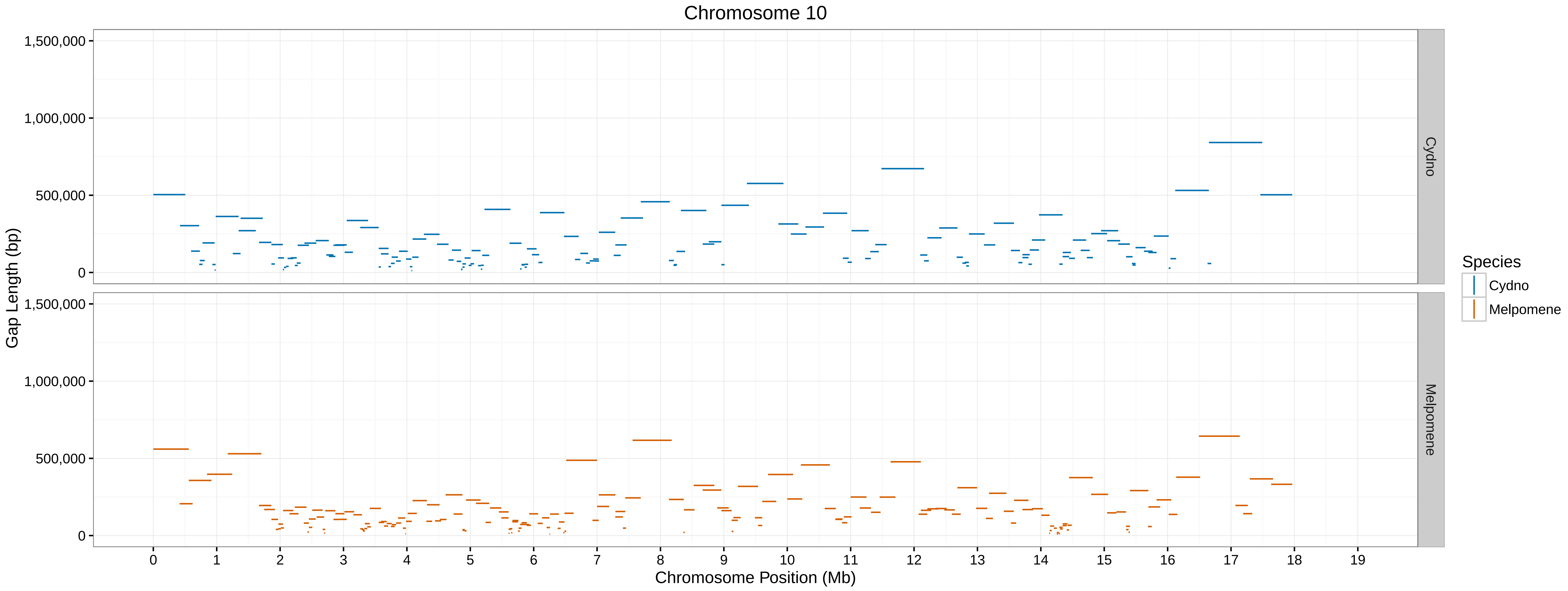

Chromosome 10
Gap Length (bp)
1,500,000
1,000,000
500,000
0
Cydno
Melpomene
0
1
2
3
4
5
6
7
8
9
10
11
12
13
14
15
16
17
18
19
Chromosome Position (Mb)
Species
Cydno
Melpomene

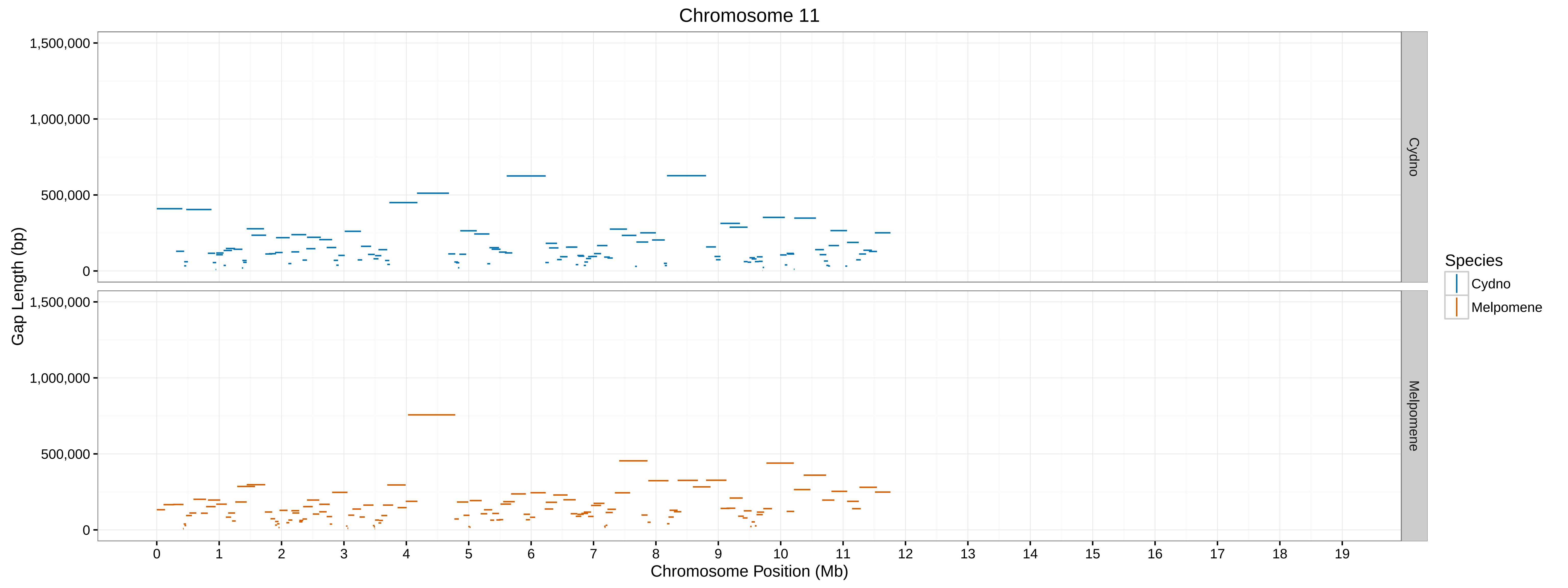

Chromosome 11
Gap Length (bp)
1,500,000
1,000,000
500,000
0
Cydno
Melpomene
Chromosome Position (Mb)
0
1
2
3
4
5
6
7
8
9
10
11
12
13
14
15
16
17
18
19
Species
Cydno
Melpomene

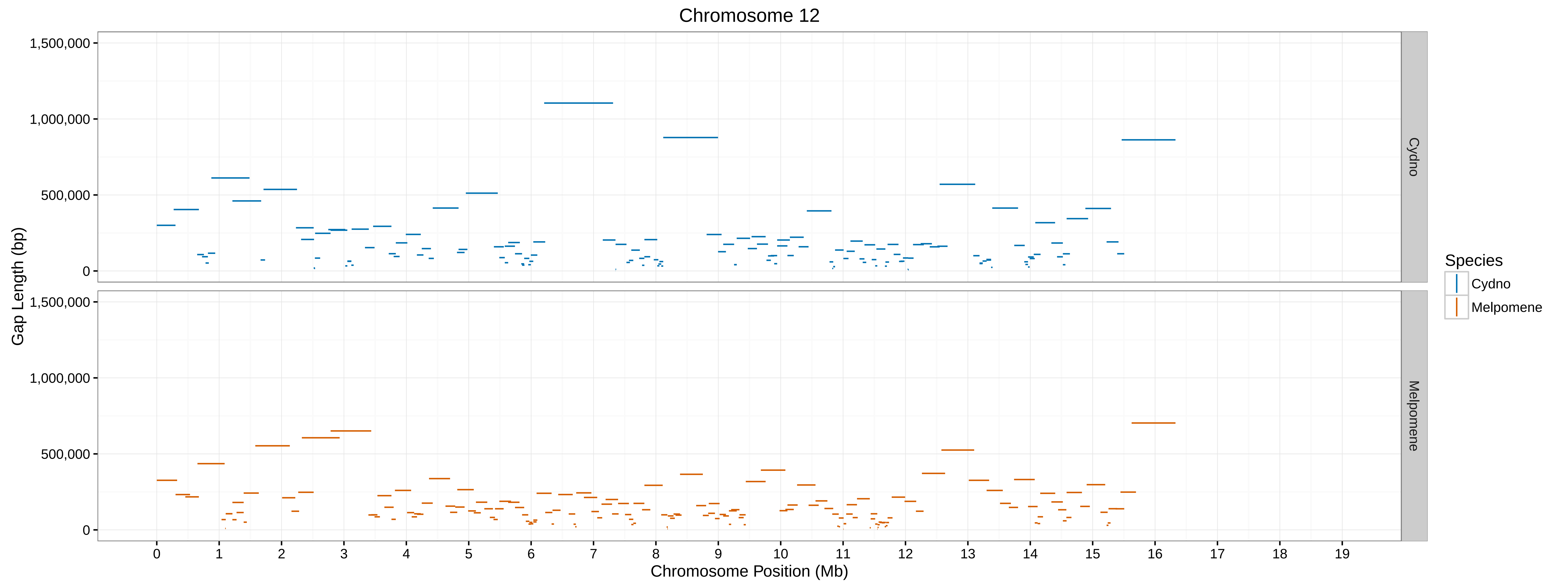

Chromosome 12
Gap Length (bp)
1,500,000
1,000,000
500,000
0
Cydno
Melpomene
Species
Cydno
Melpomene
0
1
2
3
4
5
6
7
8
9
10
11
12
13
14
15
16
17
18
19
Chromosome Position (Mb)

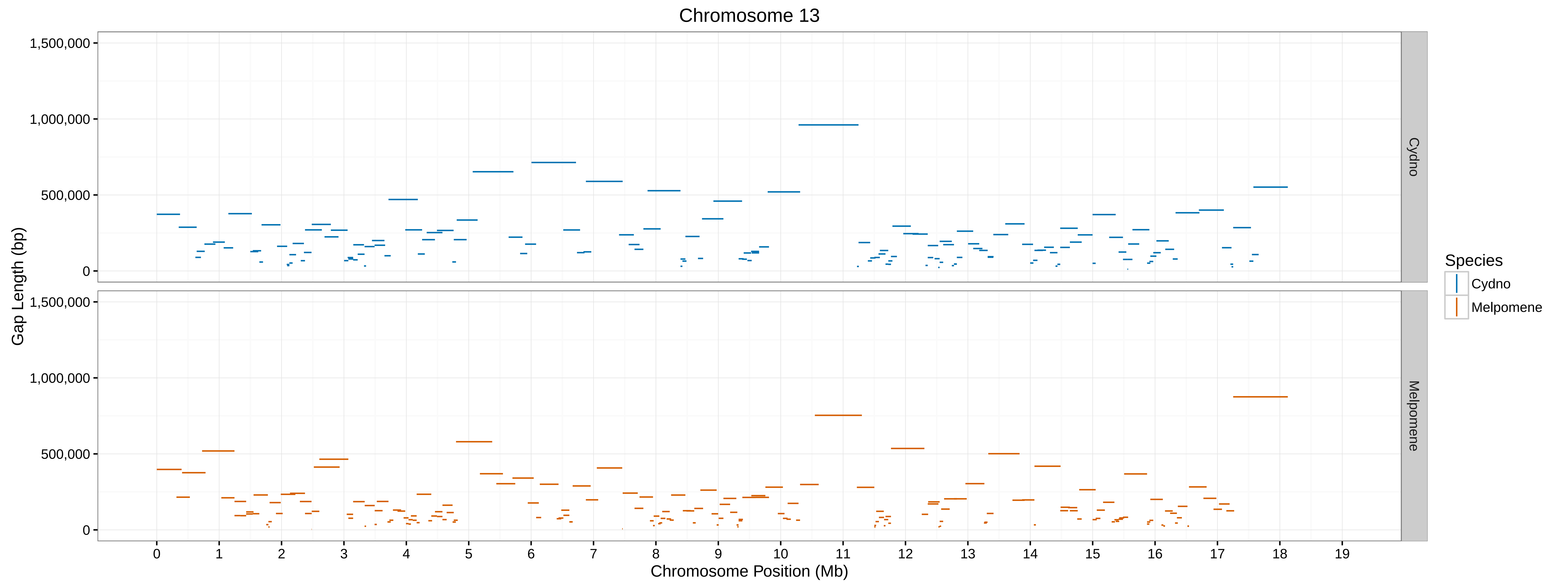

Chromosome 13
Gap Length (bp)
1,500,000
1,000,000
500,000
0
Cydno
Melpomene
0
1
2
3
4
5
6
7
8
9
10
11
12
13
14
15
16
17
18
19
Chromosome Position (Mb)
Species
Cydno
Melpomene

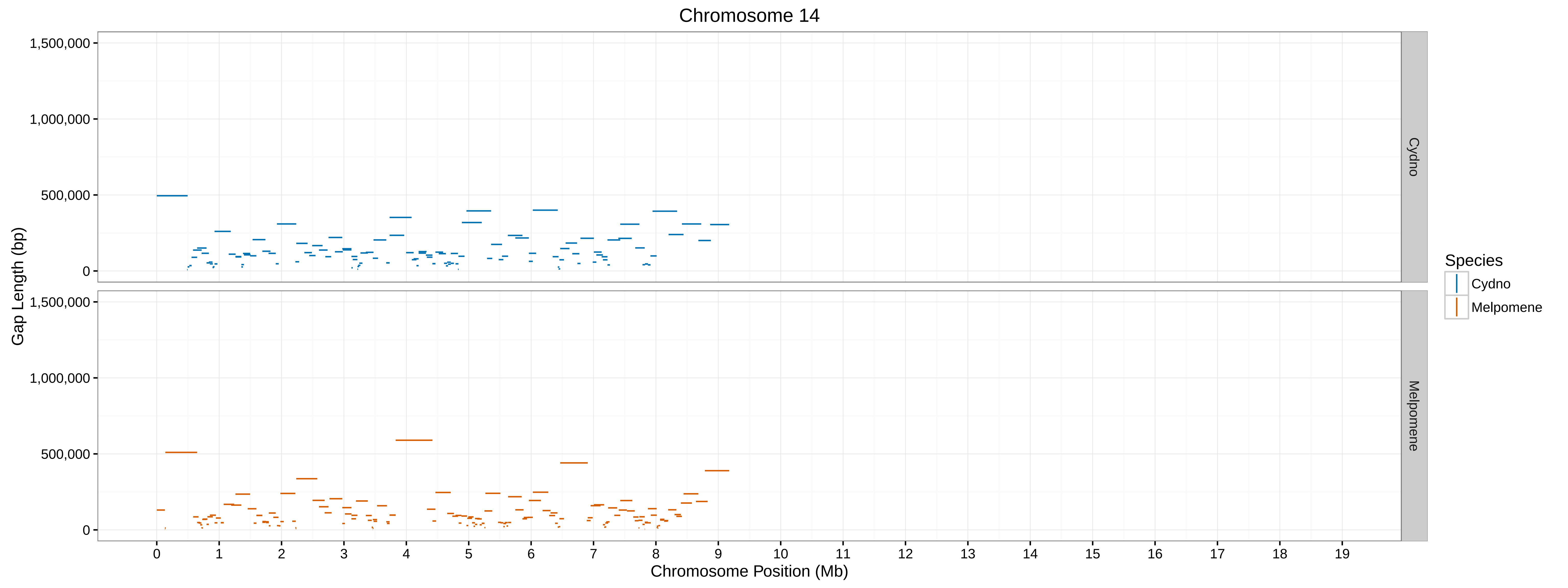

Chromosome 14
Gap Length (bp)
1,500,000
1,000,000
500,000
0
Cydno
Melpomene
Chromosome Position (Mb)
0
1
2
3
4
5
6
7
8
9
10
11
12
13
14
15
16
17
18
19
Species
Cydno
Melpomene

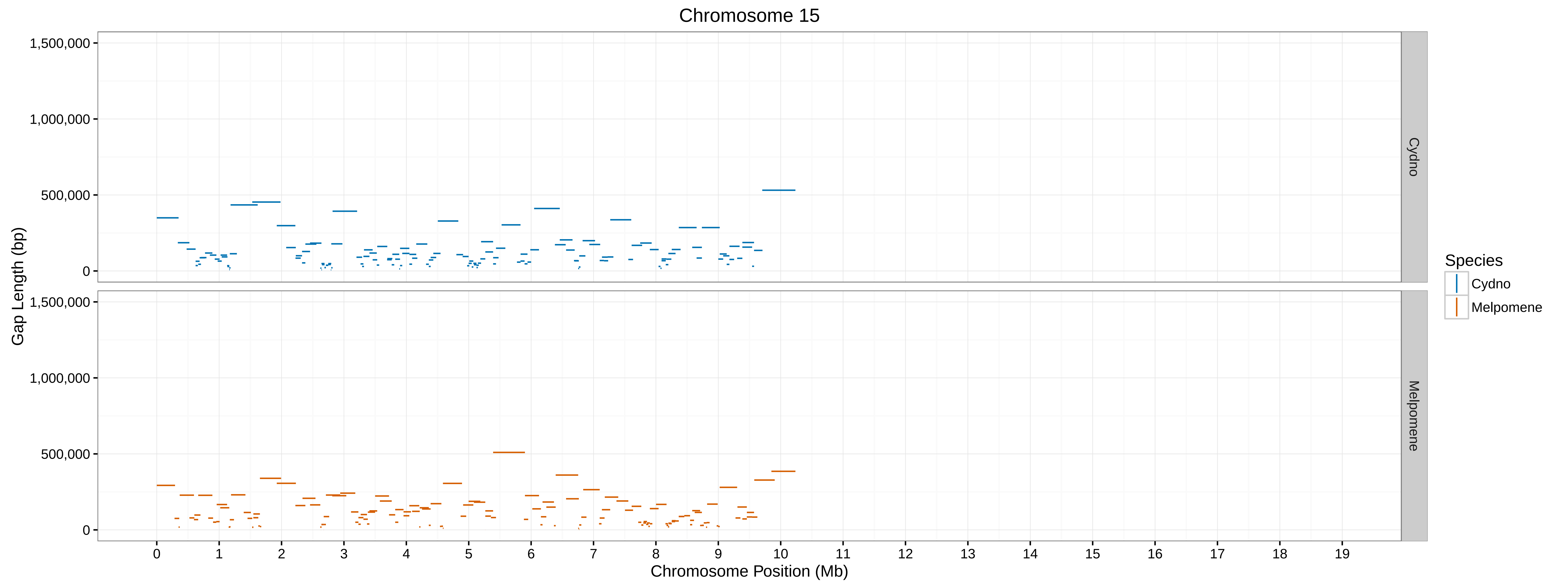

Chromosome 15
Gap Length (bp)
1,500,000
1,000,000
500,000
0
Cydno
Melpomene
Species
Cydno
Melpomene
0
1
2
3
4
5
6
7
8
9
10
11
12
13
14
15
16
17
18
19
Chromosome Position (Mb)

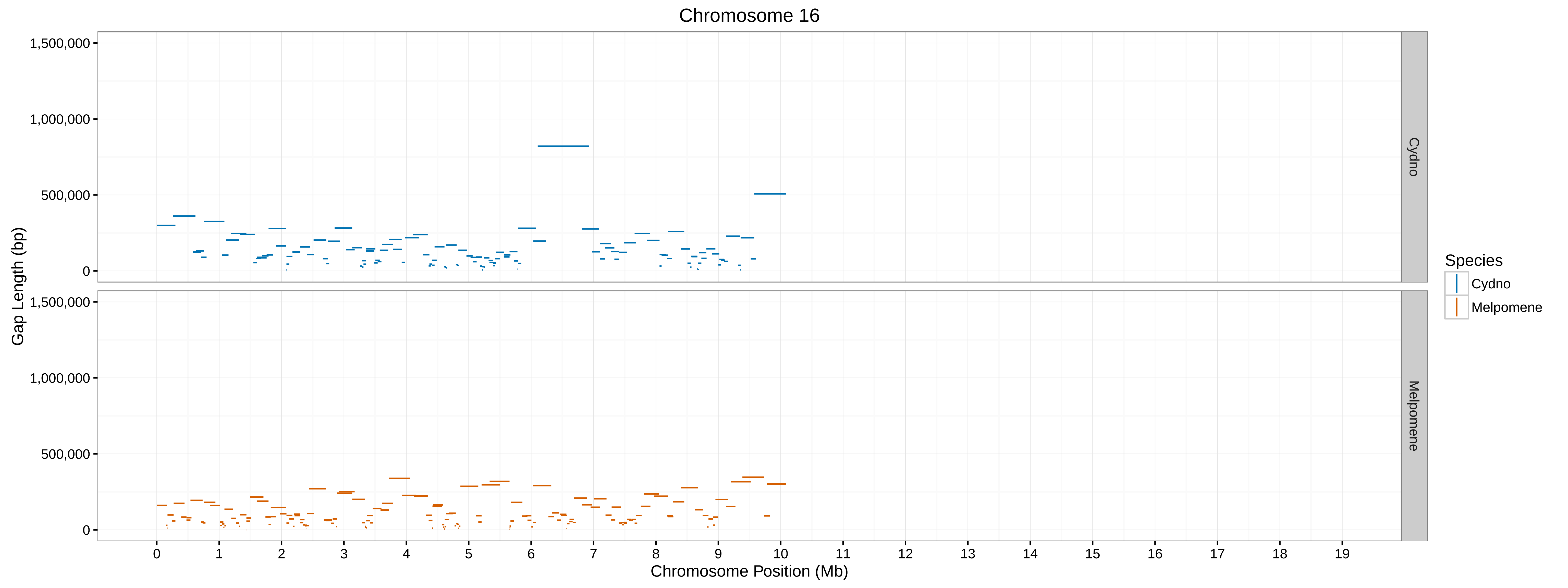

Chromosome 16
1,500,000
1,000,000
500,000
0
Gap Length (bp)
Cydno
Melpomene
Species
Cydno
Melpomene
0
1
2
3
4
5
6
7
8
9
10
11
12
13
14
15
16
17
18
19
Chromosome Position (Mb)

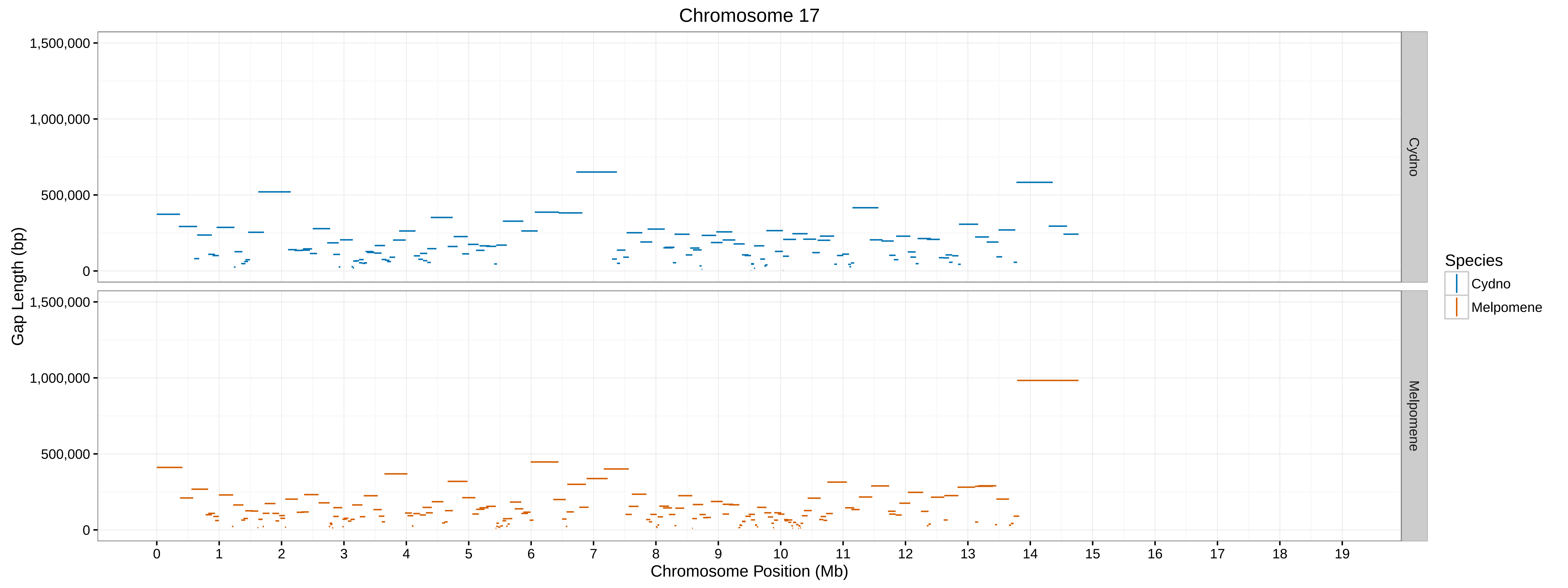

Chromosome 17
Gap Length (bp)
1,500,000
1,000,000
500,000
0
Cydno
Melpomene
0
1
2
3
4
5
6
7
8
9
10
11
12
13
14
15
16
17
18
19
Chromosome Position (Mb)
Species
Cydno
Melpomene

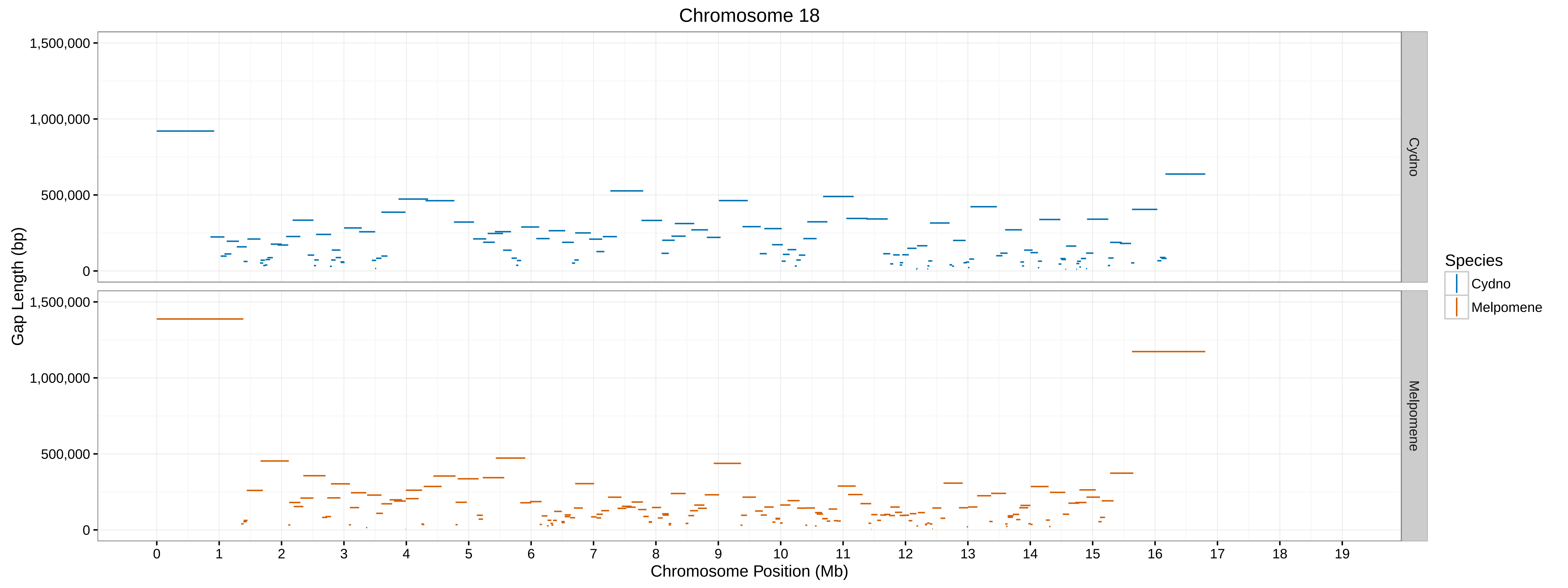

Chromosome 18
Gap Length (bp)
1,500,000
1,000,000
500,000
0
Cydno
Melpomene
Chromosome Position (Mb)
0
1
2
3
4
5
6
7
8
9
10
11
12
13
14
15
16
17
18
19
Species
Cydno
Melpomene

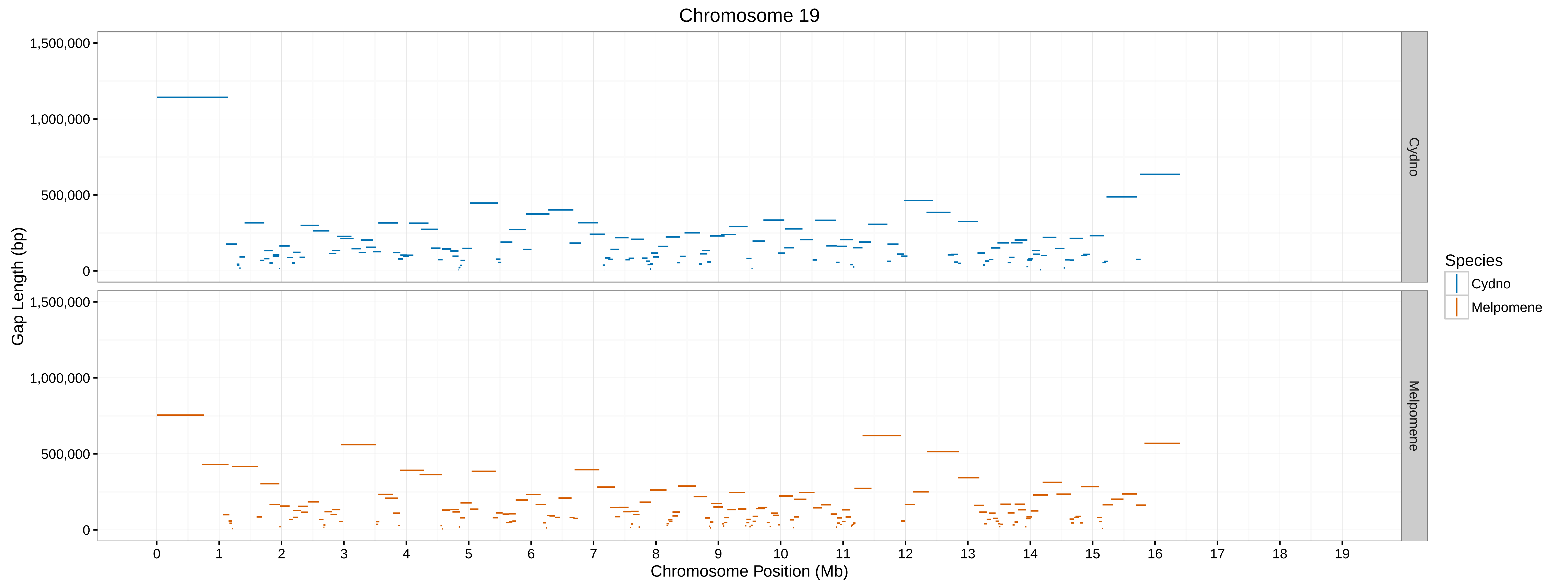

Chromosome 19
1,500,000
1,000,000
500,000
0
Gap Length (bp)
Cydno
Melpomene
0
1
2
3
4
5
6
7
8
9
10
11
12
13
14
15
16
17
18
19
Chromosome Position (Mb)
Species
Cydno
Melpomene

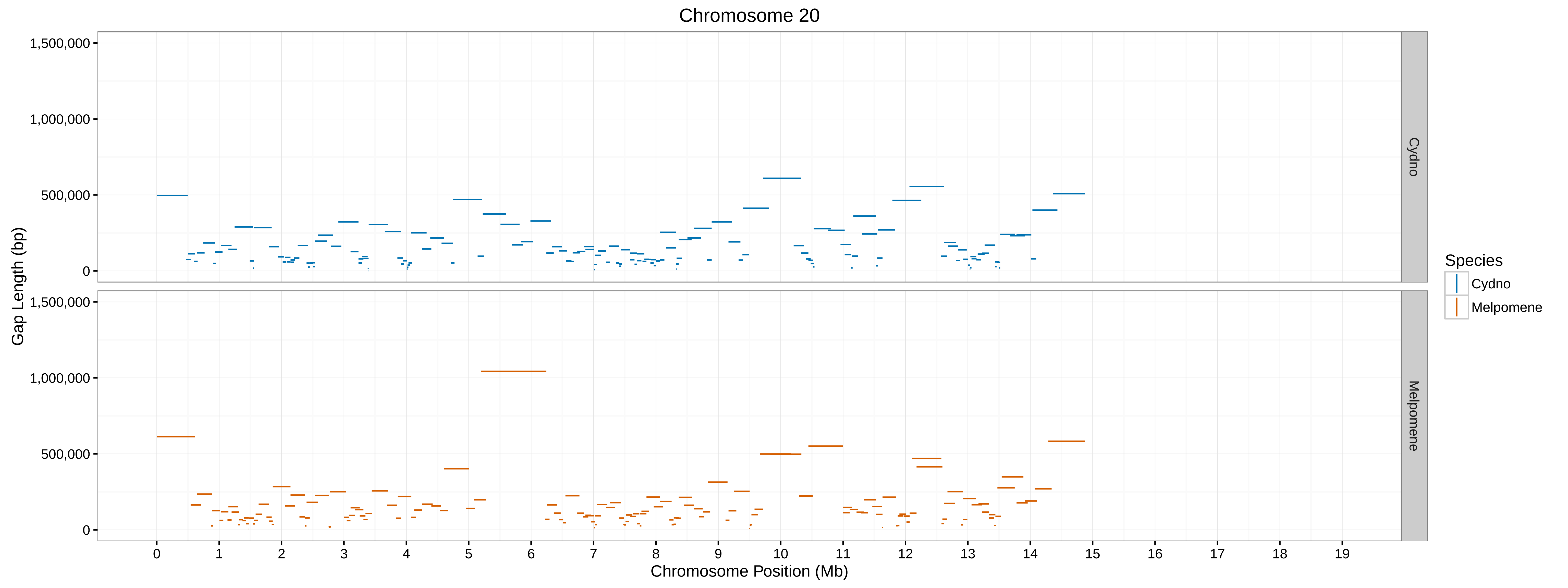
Chromosome 20
Gap Length (bp)
1,500,000
1,000,000
500,000
0
Cydno
Melpomene
Chromosome Position (Mb)
0
1
2
3
4
5
6
7
8
9
10
11
12
13
14
15
16
17
18
19
Species
Cydno
Melpomene

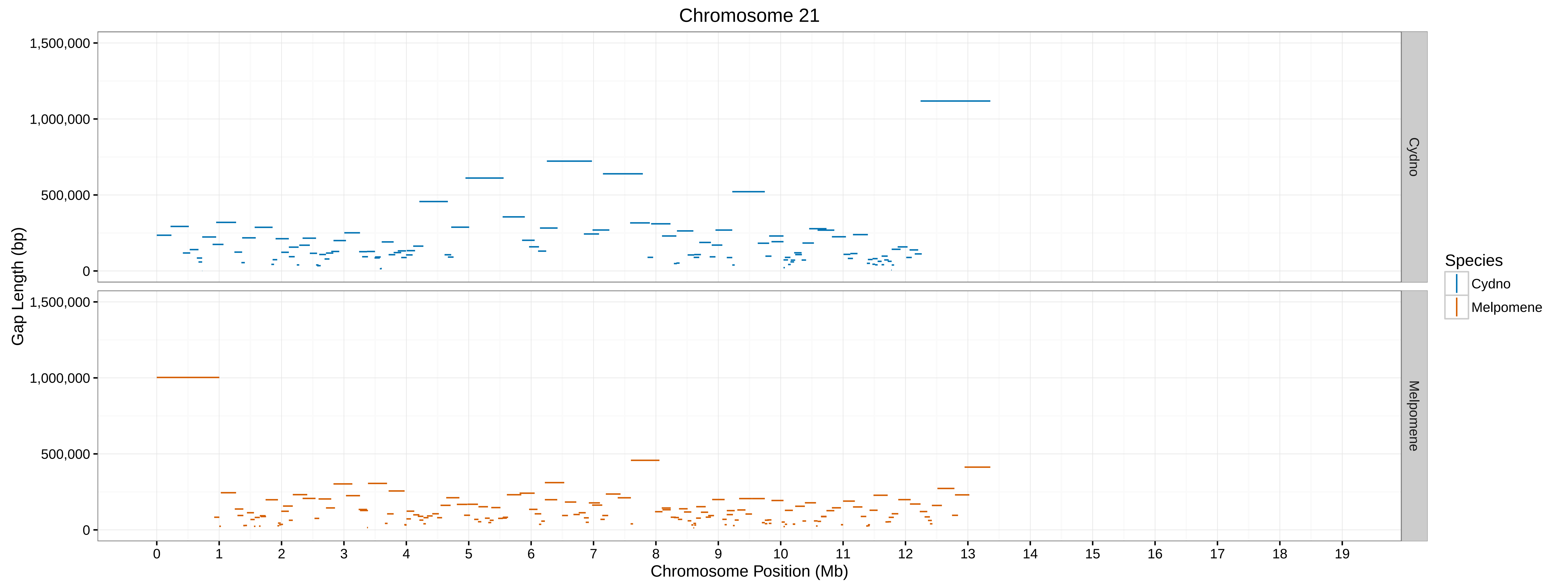

Chromosome 21
1,500,000
1,000,000
500,000
0
Gap Length (bp)
Cydno
Melpomene
0
1
2
3
4
5
6
7
8
9
10
11
12
13
14
15
16
17
18
19
Chromosome Position (Mb)
Species
Cydno
Melpomene
