# Supplementary material for: No evidence for maintenance of a sympatric Heliconius species barrier by chromosomal inversions
Source: Evol Lett. 2017 Jun 14;1(3):138–54. doi: 10.1002/evl3.12 (PMC6122123; doi:10.1002/evl3.12)

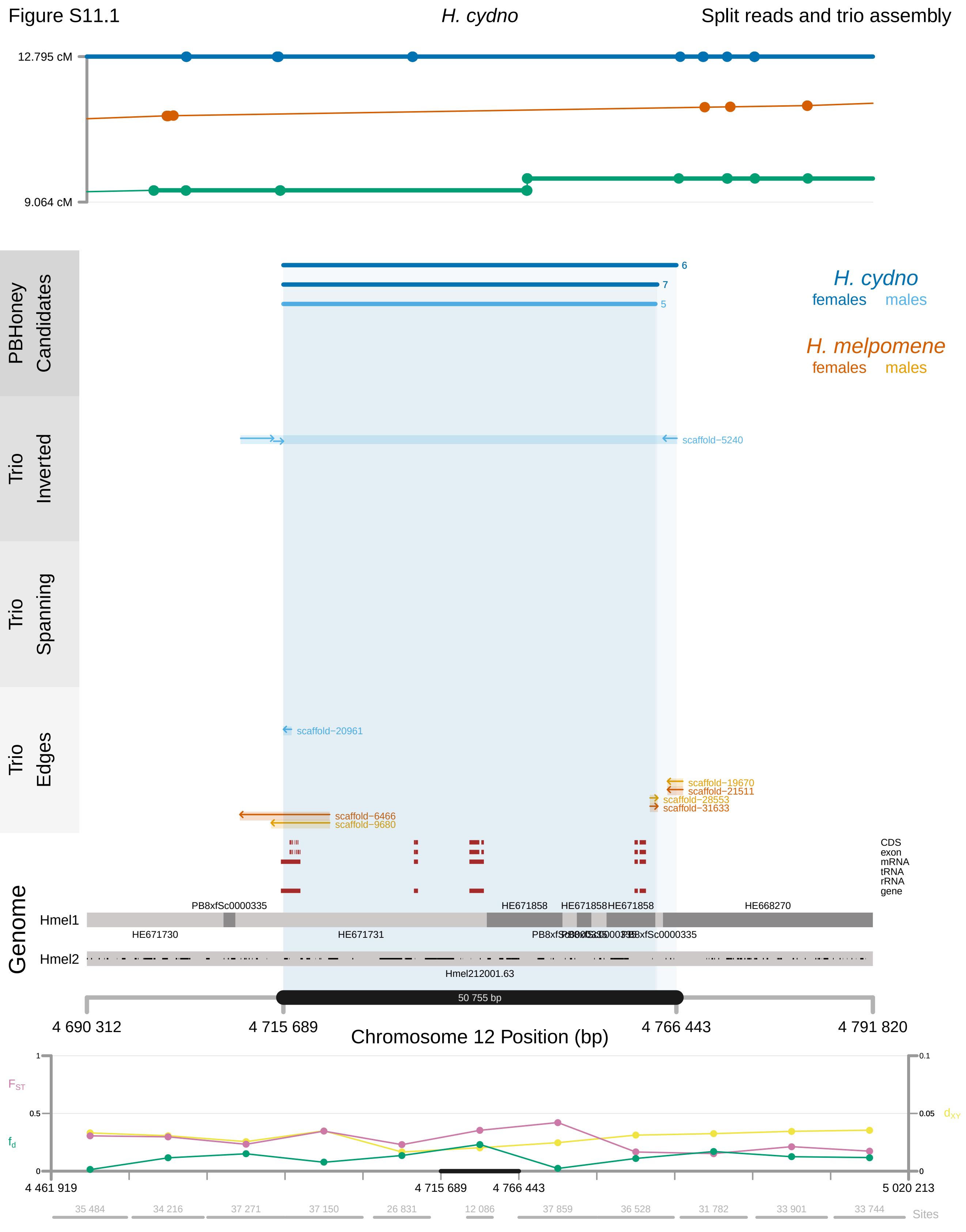

Figure S11.1
H. cydno
Split reads and trio assembly
12.795 cM
9.064 cM
PBHoney Candidates
Trio Inverted
Trio Spanning
Trio Edges
Genome
H. cydno
females males
H. melpomene
females males
6
7
5
scaffold−5240
scaffold−20961
scaffold−19670
scaffold−21511
scaffold−28553
scaffold−31633
scaffold−6466
scaffold−9680
CDS
exon
mRNA
tRNA
rRNA
gene
PB8xfSc0000335
HE671858
HE671858
HE671858
HE668270
Hmel1
HE671730
HE671731
PB8xfSc0000335
Hmel2
Hmel212001.63
50 755 bp
4 690 312
4 715 689
4 766 443
4 791 820
Chromosome 12 Position (bp)
1
0.5
0
F_{ST}
f_d
0.1
0.05
0
d_{XY}
4 461 919
4 715 689
4 766 443
5 020 213
35 484
34 216
37 271
37 150
26 831
12 086
37 859
36 528
31 782
33 901
33 744
Sites

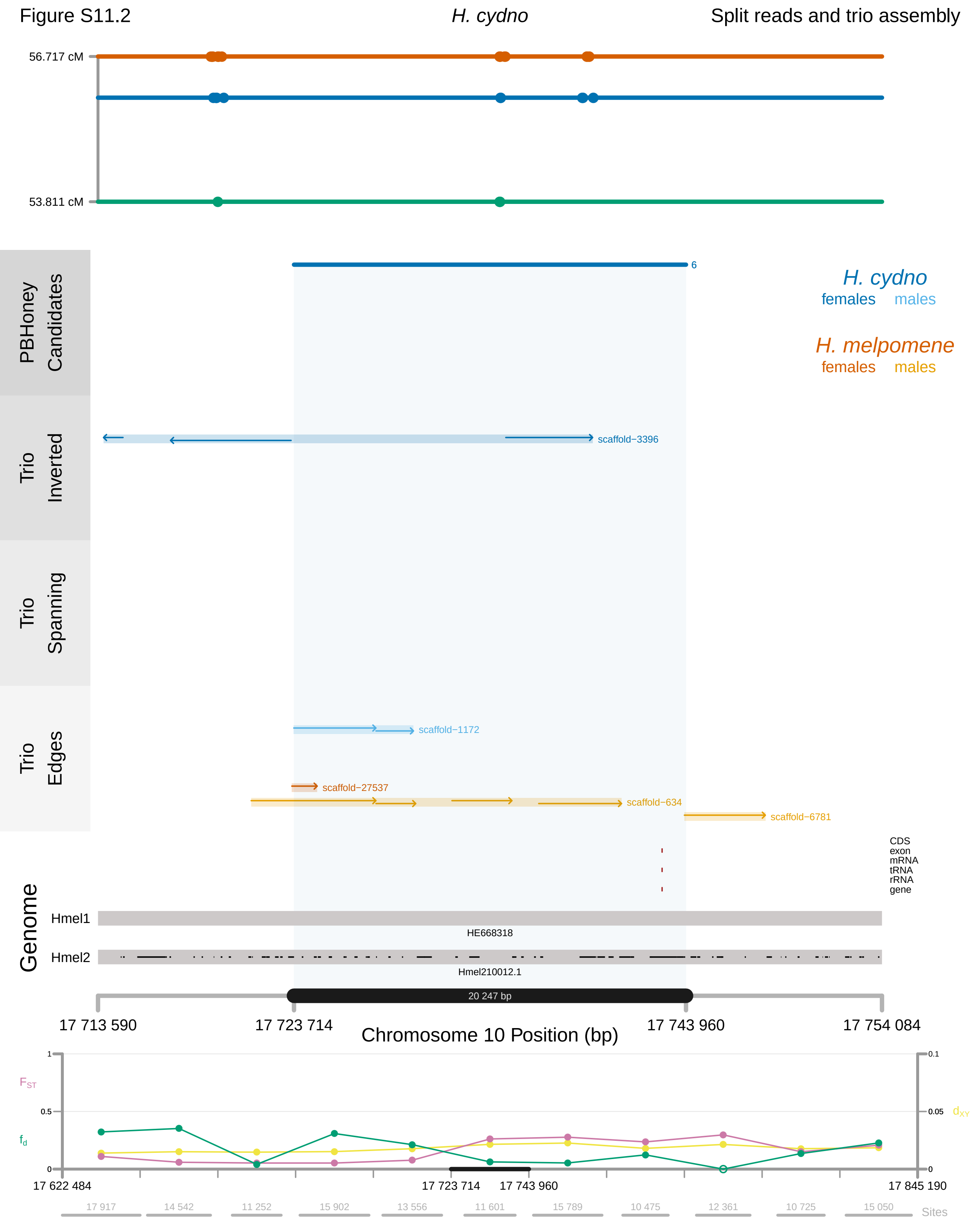

Figure S11.2
H. cydno
Split reads and trio assembly
56.717 cM
53.811 cM
PBHoney Candidates
6
H. cydno
females males
H. melpomene
females males
Trio Inverted
scaffold−3396
Trio Spanning
Trio Edges
scaffold−1172
scaffold−27537
scaffold−634
scaffold−6781
CDS
exon
mRNA
tRNA
rRNA
gene
Genome
Hmel1
HE668318
Hmel2
Hmel210012.1
20 247 bp
17 713 590
17 723 714
17 743 960
17 754 084
Chromosome 10 Position (bp)
1
0.5
0
F_ST
f_d
0.1
0.05
0
d_XY
17 622 484
17 723 714
17 743 960
17 845 190
17 917
14 542
11 252
15 902
13 556
11 601
15 789
10 475
12 361
10 725
15 050
Sites

Figure S11.3 *H. cydno* Split reads and trio assembly

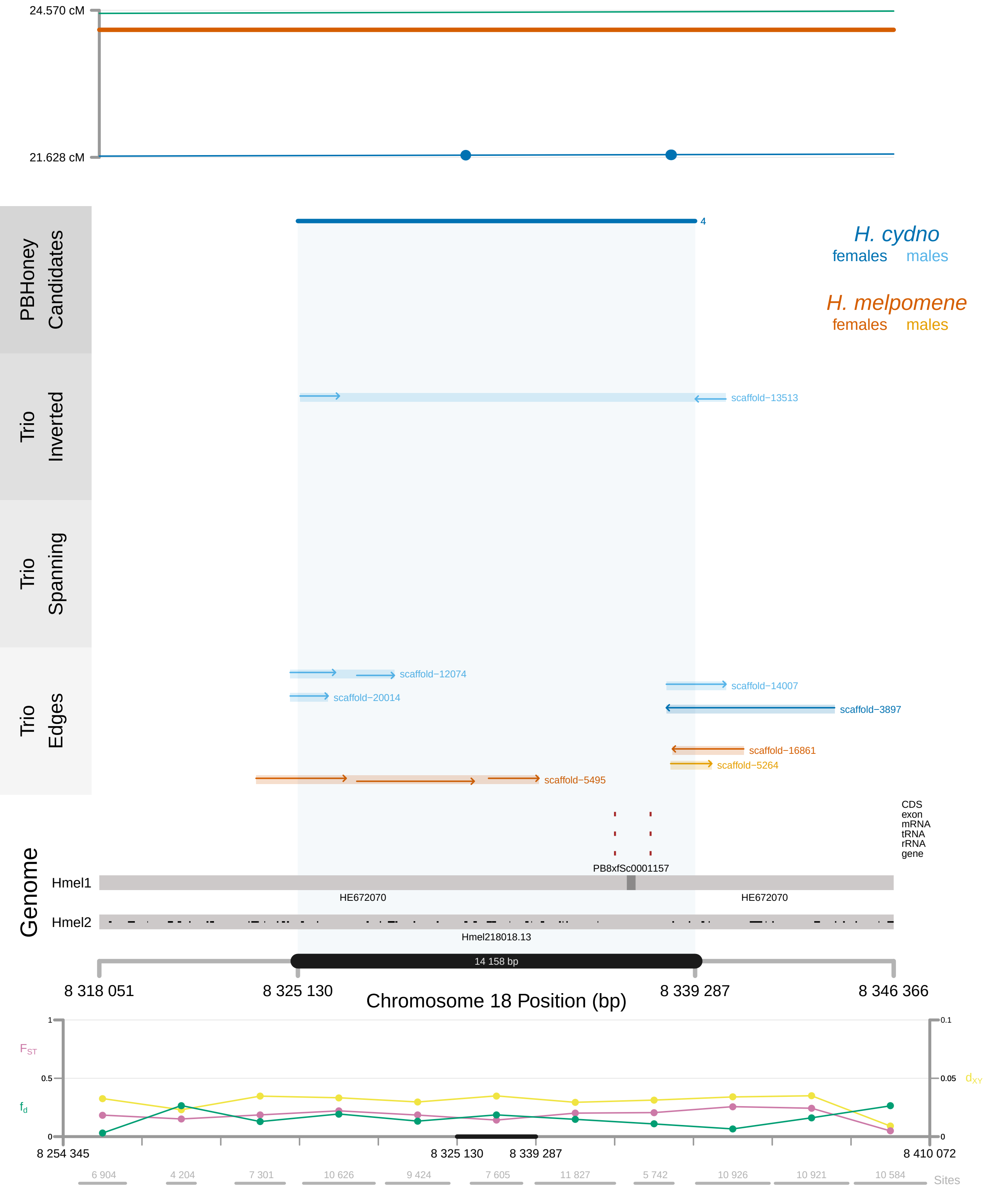

Figure S11.4 *H. cydno* Split reads and trio assembly

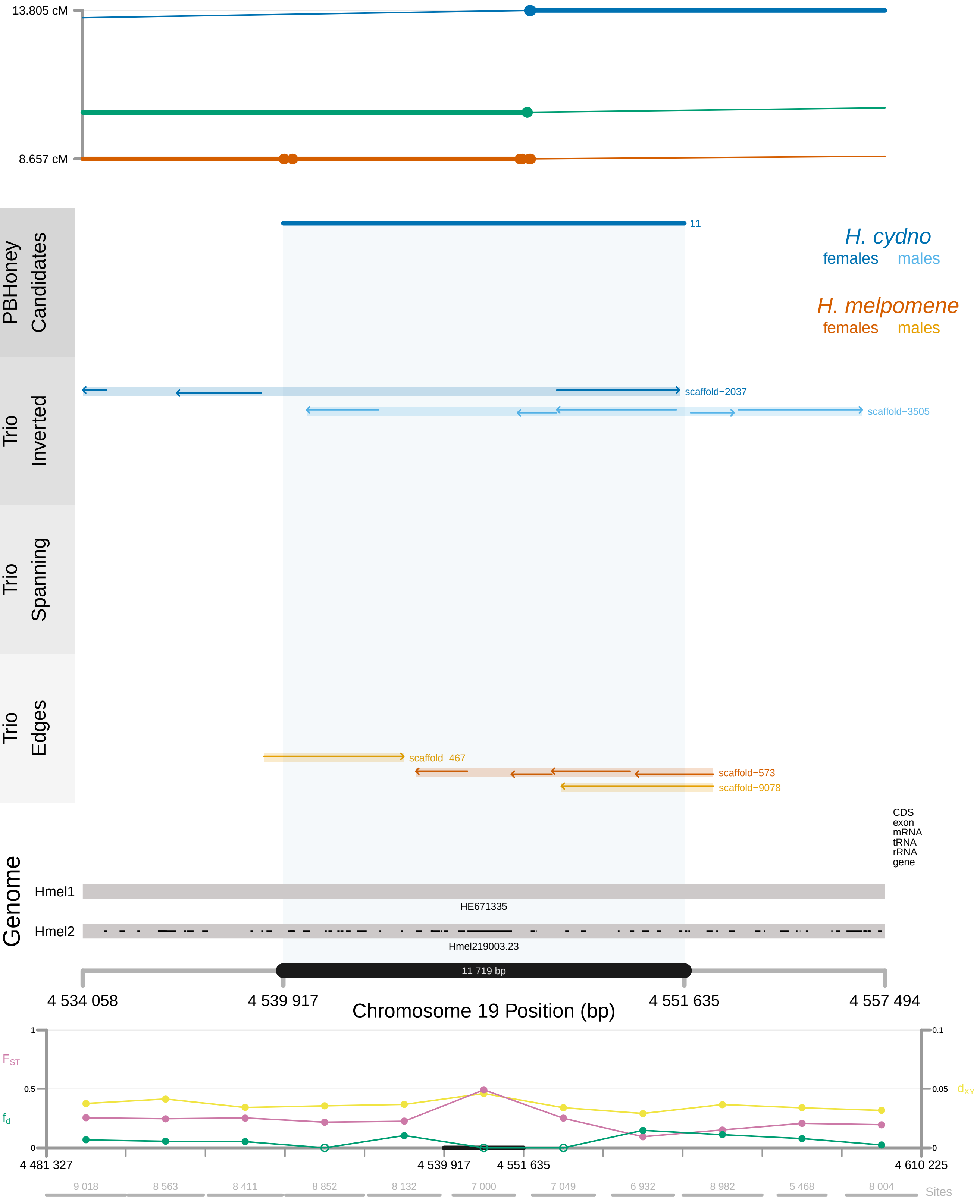

Figure S11.5 *H. cydno* Split reads and trio assembly

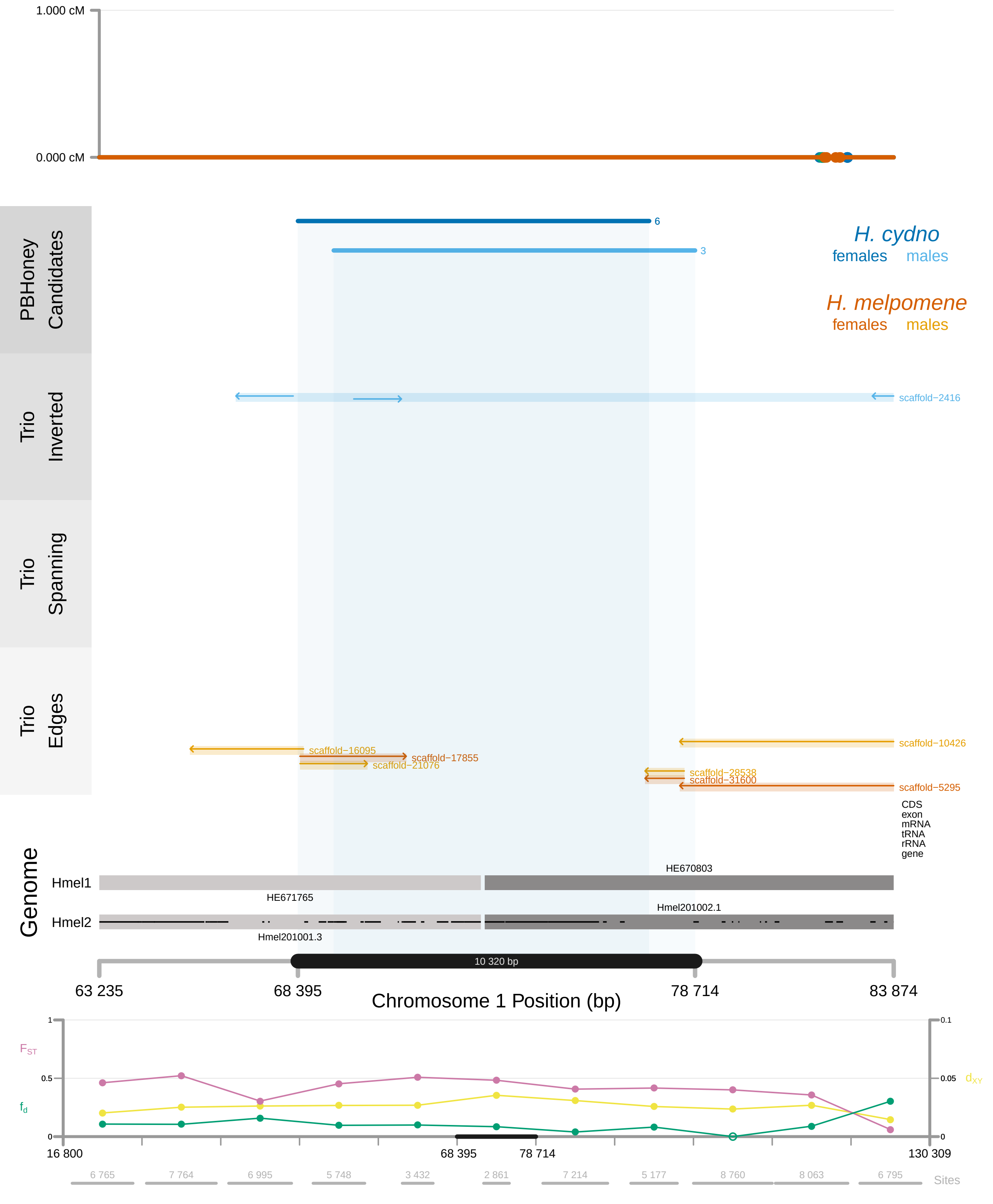

Figure S11.6 *H. cydno* Split reads and trio assembly

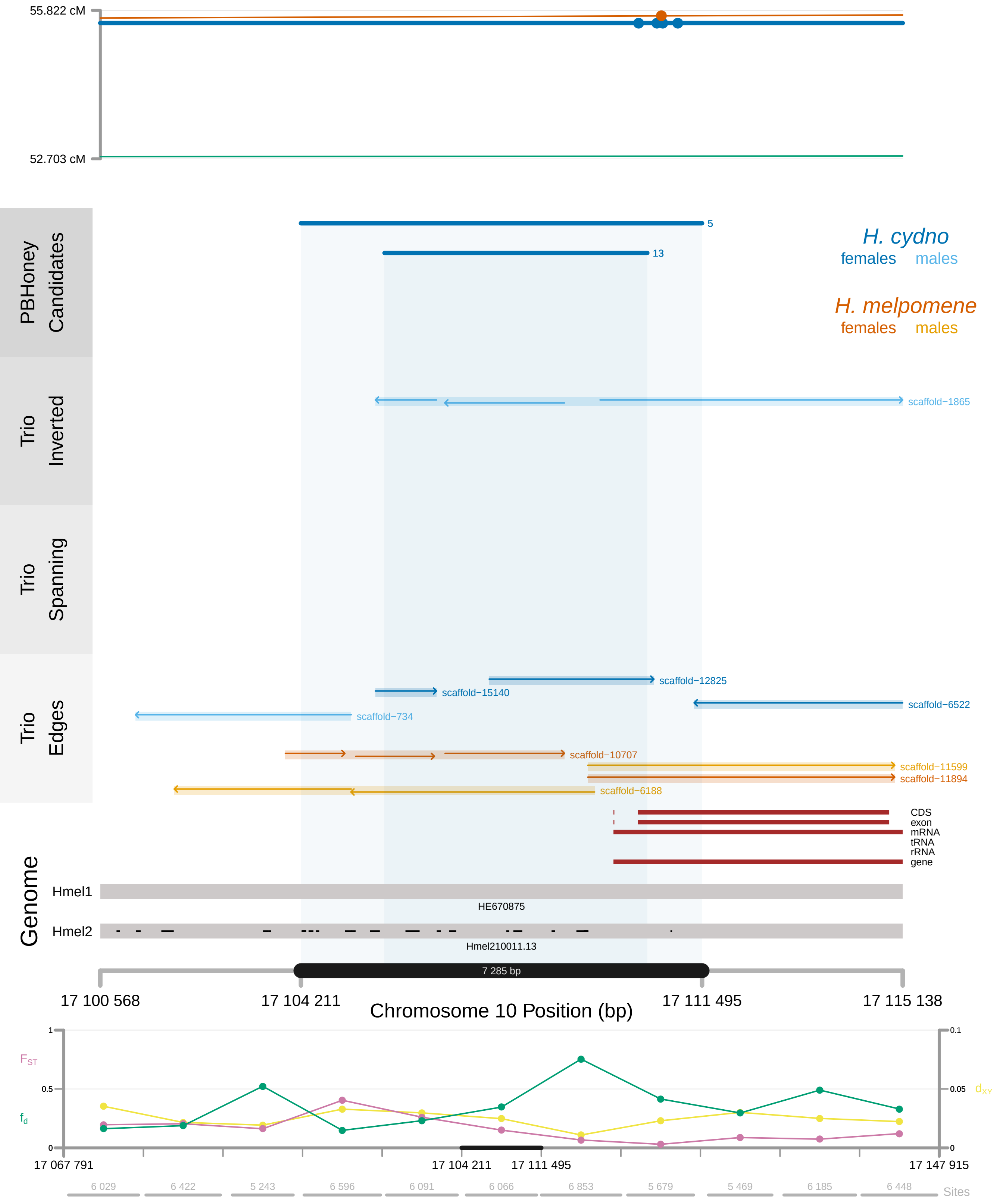

Figure S11.7 *H. cydno* Split reads and trio assembly

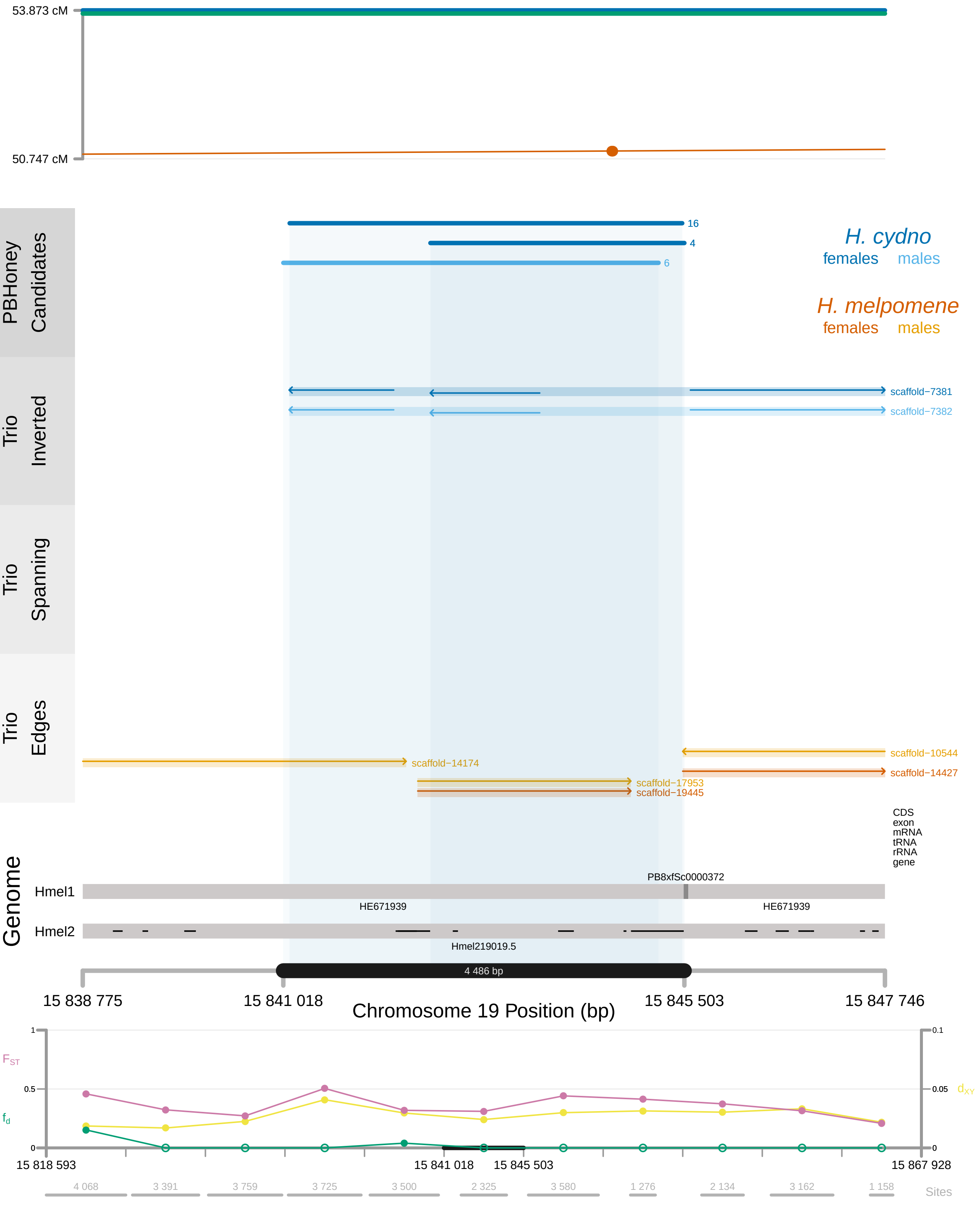

Figure S11.8 *H. cydno* Split reads and trio assembly

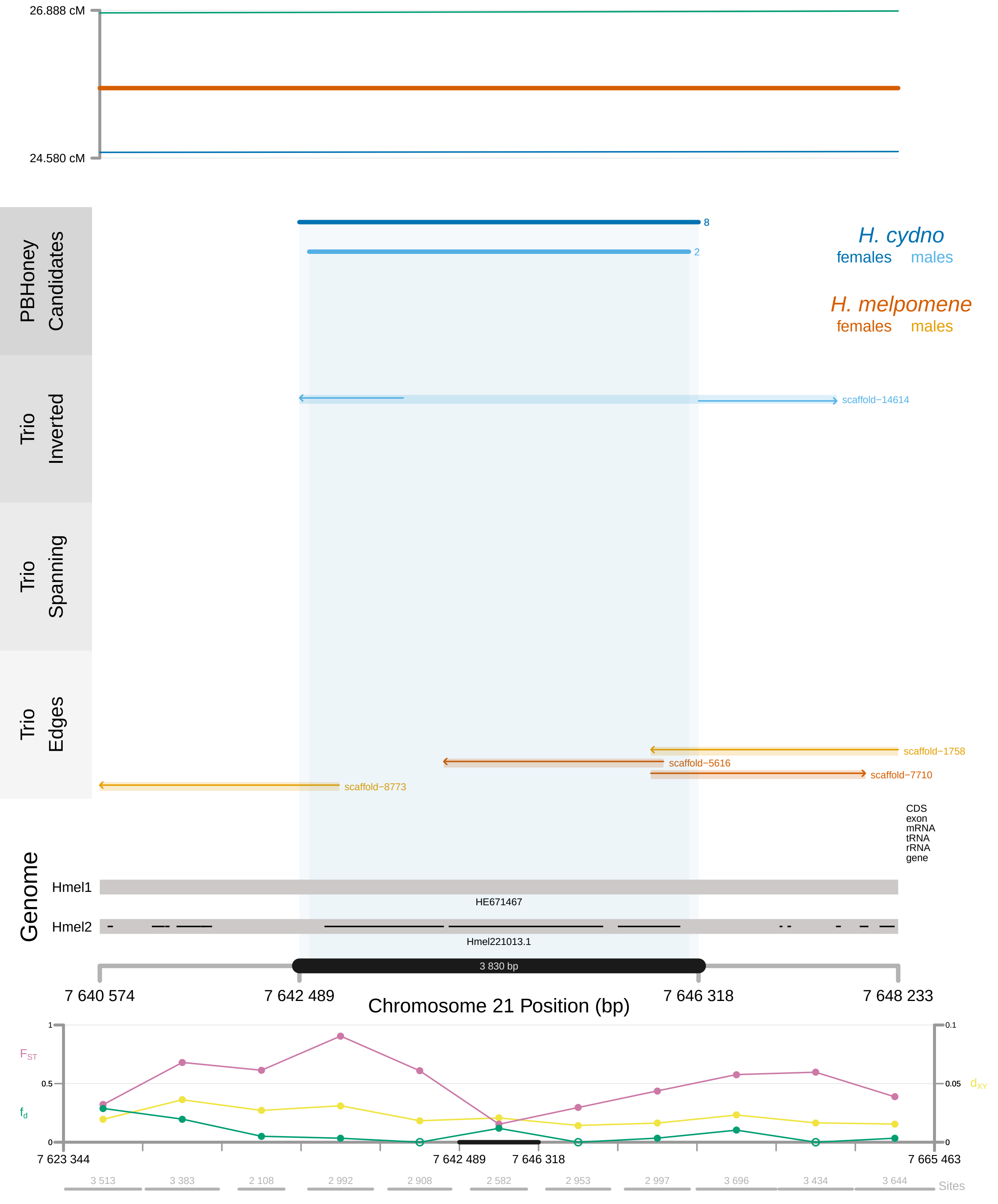

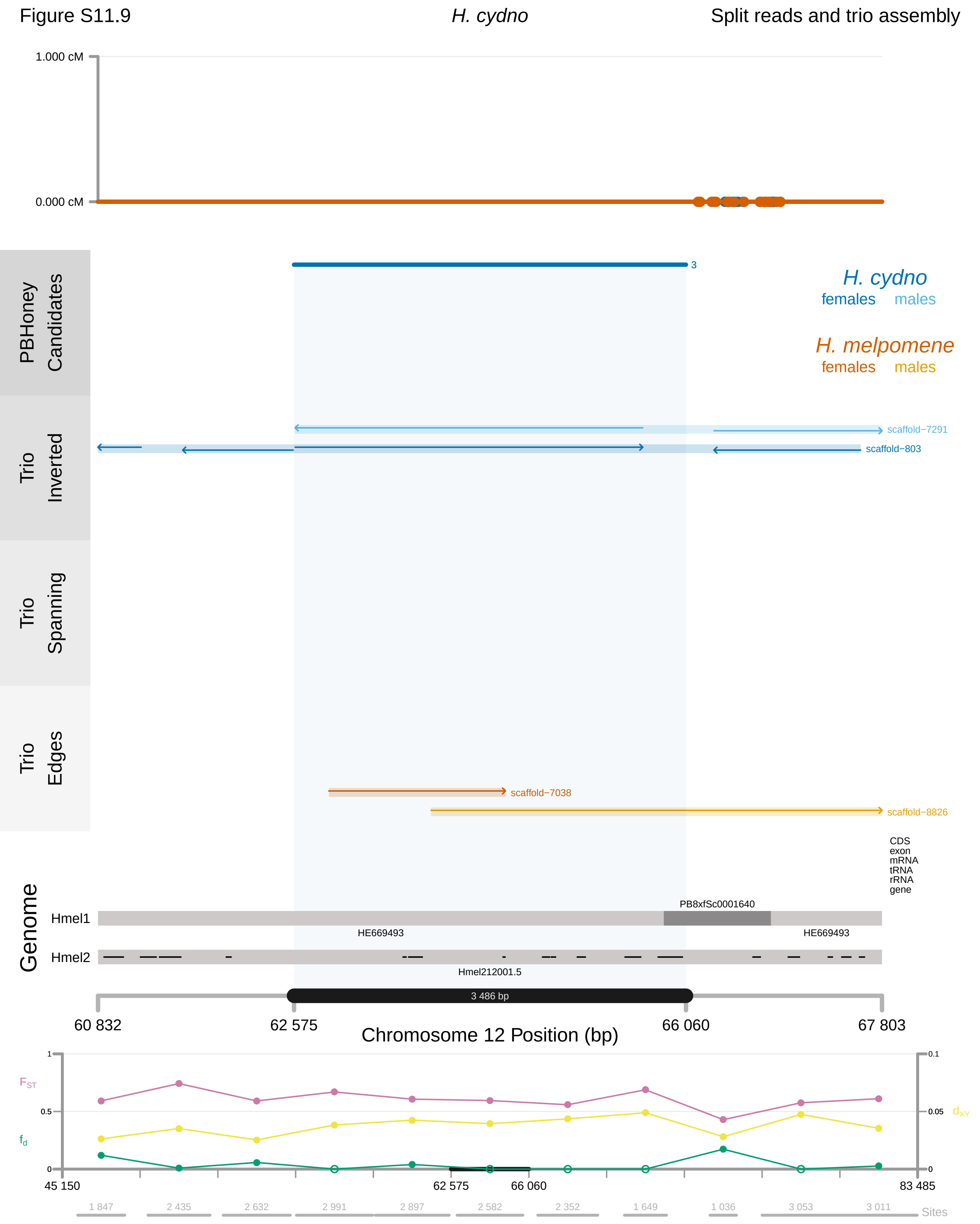

Figure S11.9
H. cydno
Split reads and trio assembly
1.000 cM
0.000 cM
PBHoney Candidates
3
H. cydno
females
males
H. melpomene
females
males
Trio Inverted
scaffold−7291
scaffold−803
Trio Spanning
Trio Edges
scaffold−7038
scaffold−8826
CDS
exon
mRNA
tRNA
rRNA
gene
Genome
PB8xfSc0001640
Hmel1
HE669493
HE669493
Hmel2
Hmel212001.5
3 486 bp
60 832
62 575
66 060
67 803
Chromosome 12 Position (bp)
1
0.5
0
FST
fd
0.1
0.05
0
dXY
45 150
62 575
66 060
83 485
1 847
2 435
2 632
2 991
2 897
2 582
2 352
1 649
1 036
3 053
3 011
Sites

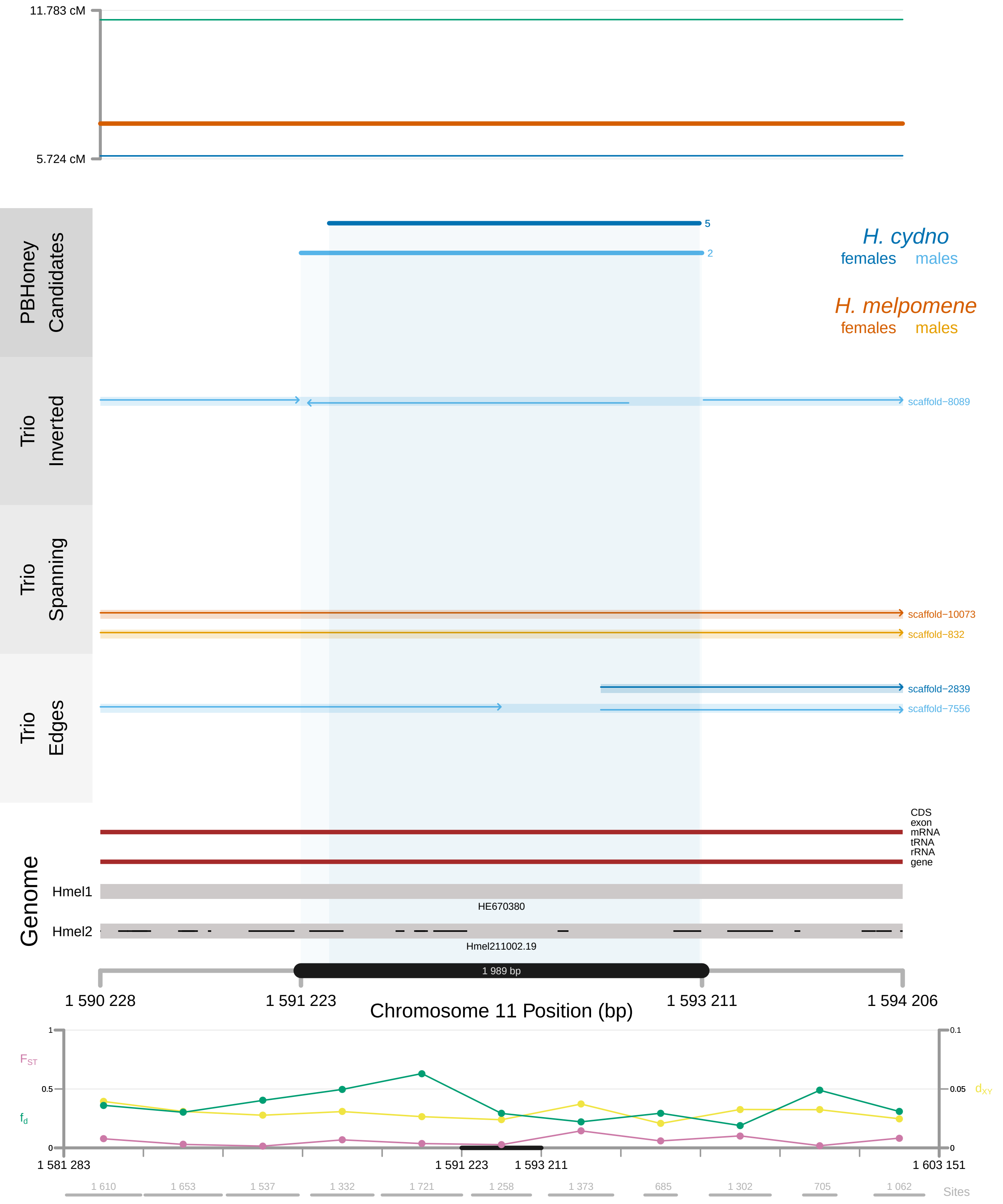
11.783 cM
5.724 cM
PBHoney Candidates
5
2
H. cydno
females males
H. melpomene
females males
Trio Inverted
scaffold−8089
Trio Spanning
scaffold−10073
scaffold−832
Trio Edges
scaffold−2839
scaffold−7556
Genome
CDS
exon
mRNA
tRNA
rRNA
gene
Hmel1
HE670380
Hmel2
Hmel211002.19
1 989 bp
1 590 228
1 591 223
1 593 211
1 594 206
Chromosome 11 Position (bp)
1
0.5
0
F_ST
f_d
0.1
0.05
0
d_XY
1 581 283
1 591 223
1 593 211
1 603 151
1 610
1 653
1 537
1 332
1 721
1 258
1 373
685
1 302
705
1 062
Sites

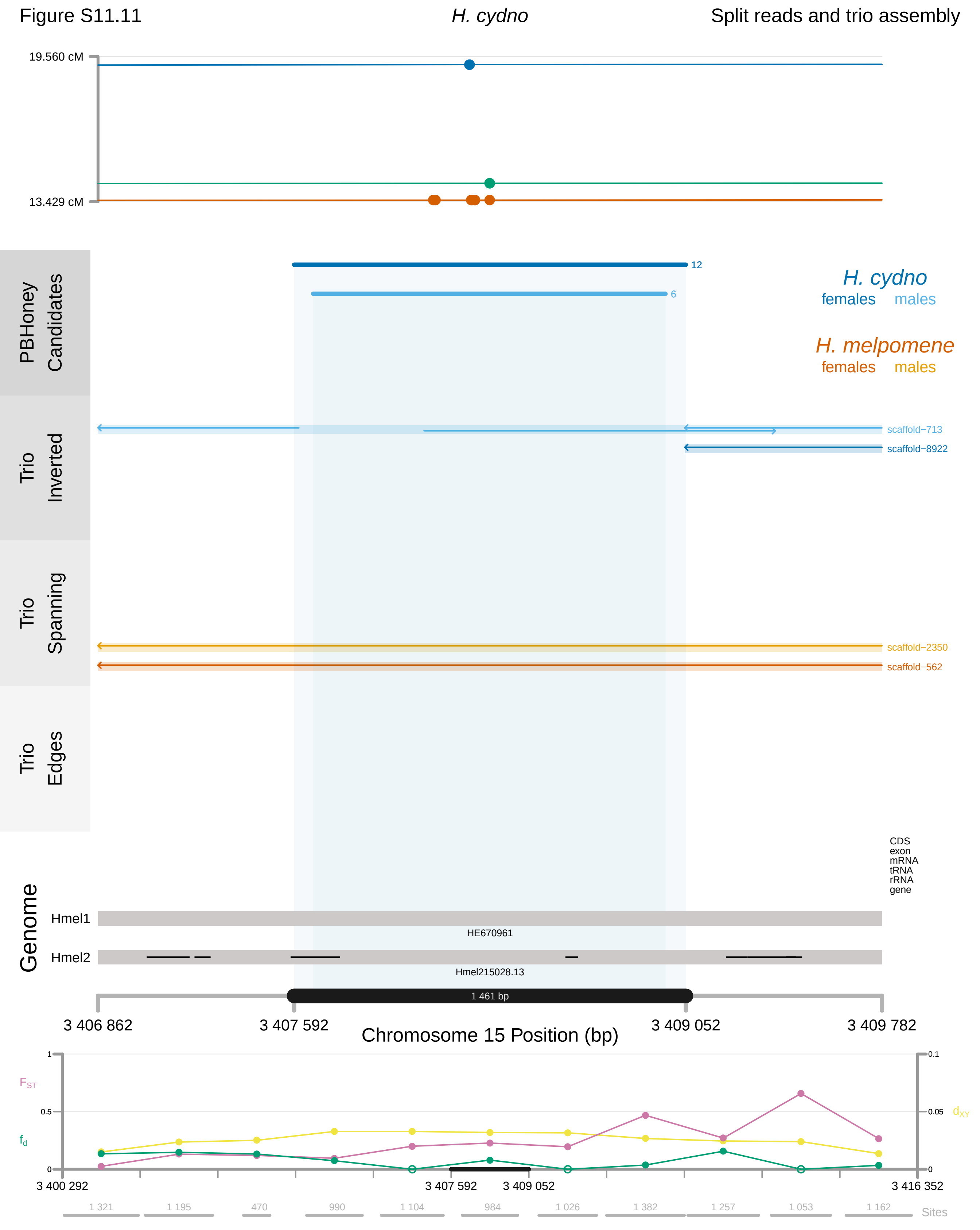

Figure S11.11
H. cydno
Split reads and trio assembly
19.560 cM
13.429 cM
PBHoney Candidates
12
6
H. cydno
females males
H. melpomene
females males
Trio Inverted
scaffold−713
scaffold−8922
Trio Spanning
scaffold−2350
scaffold−562
Trio Edges
CDS
exon
mRNA
tRNA
rRNA
gene
Genome
Hmel1
HE670961
Hmel2
Hmel215028.13
1 461 bp
3 406 862
3 407 592
3 409 052
3 409 782
Chromosome 15 Position (bp)
1
0.5
0
0.1
0.05
0
FST
fd
dXY
3 400 292
3 407 592
3 409 052
3 416 352
1 321
1 195
470
990
1 104
984
1 026
1 382
1 257
1 053
1 162
Sites

Figure S11.12 *H. cydno* Split reads and trio assembly

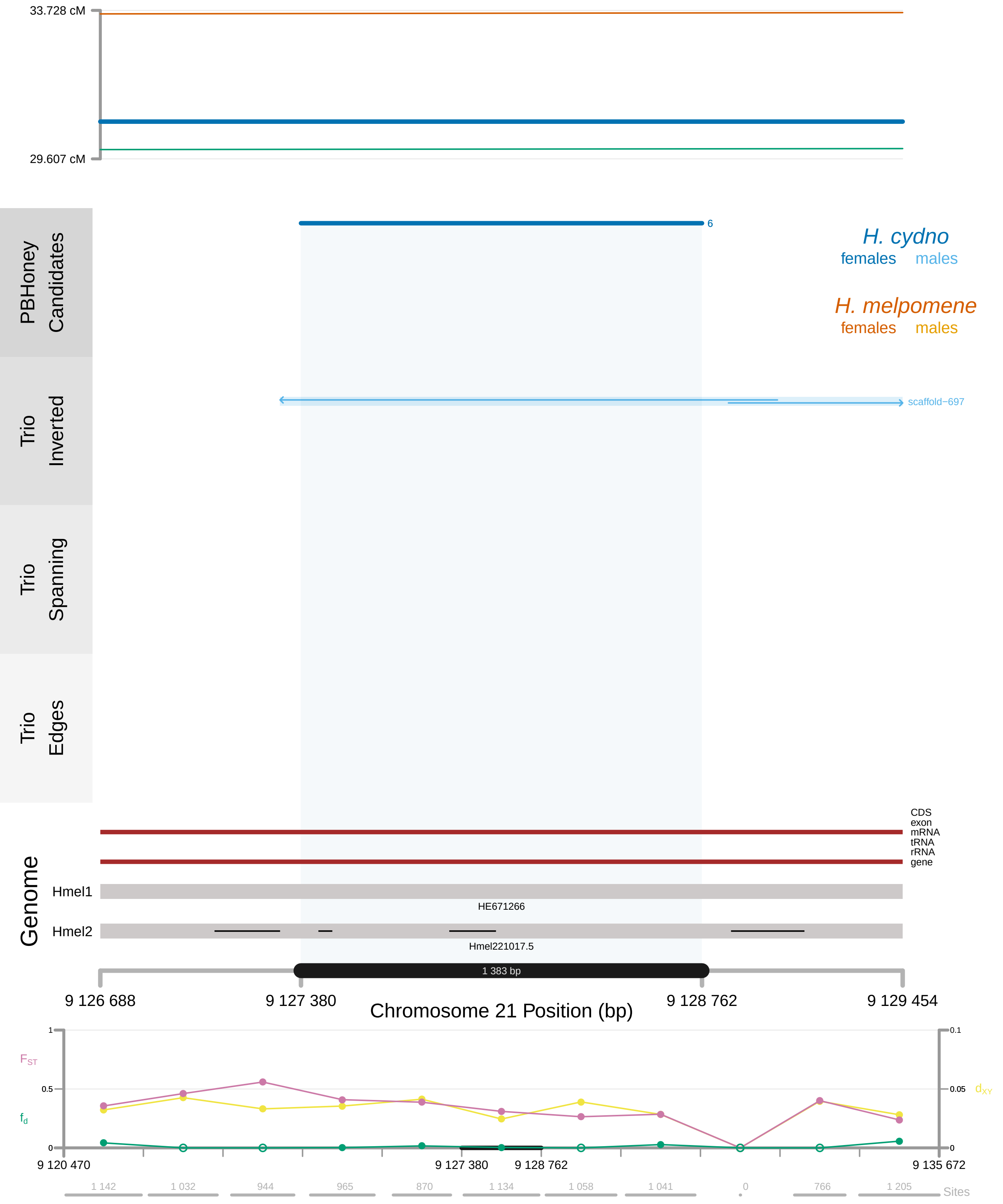

Figure S11.13 *H. cydno* Split reads and trio assembly

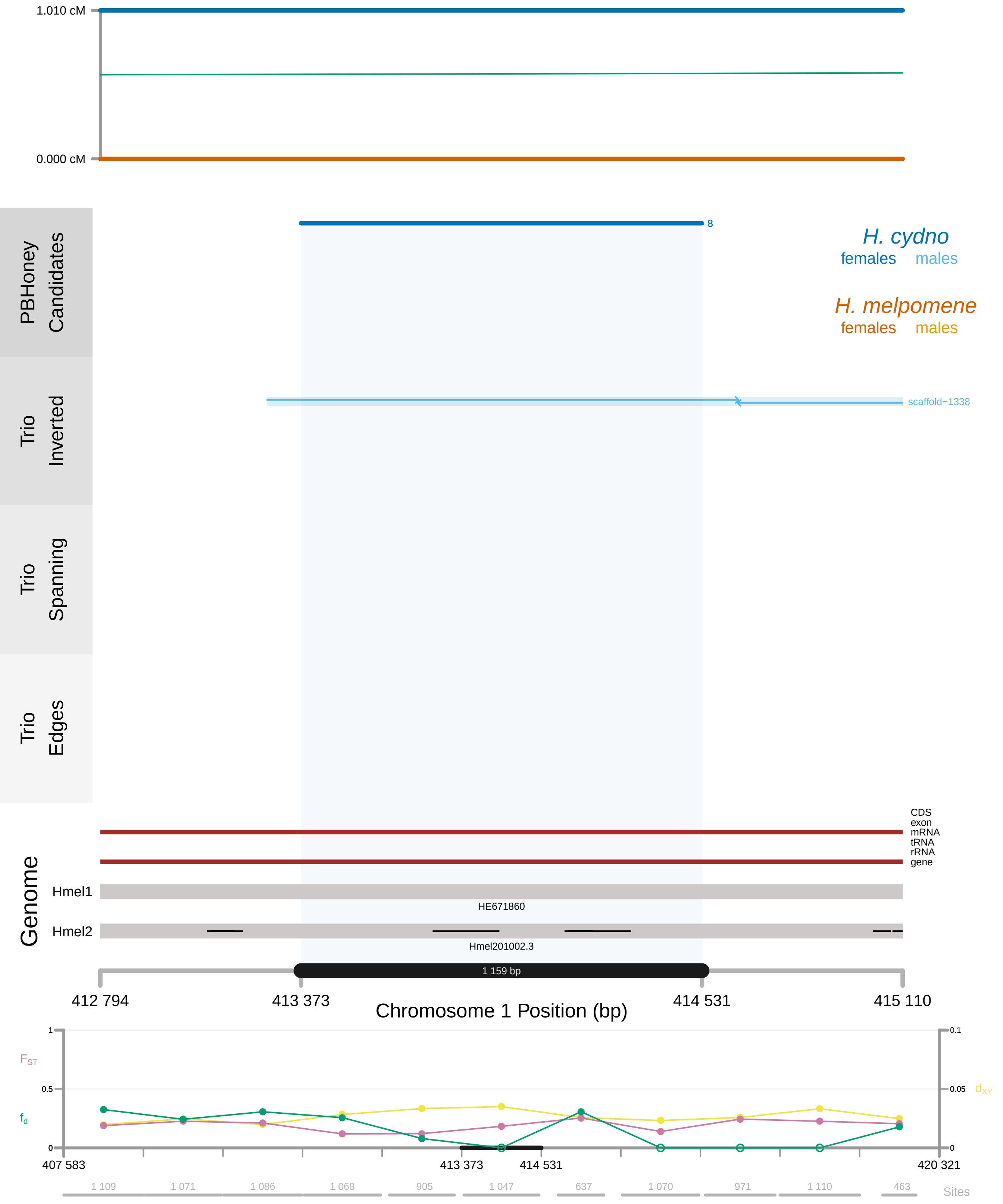

Supplement: Supplementary file 12 — Figures S11–S17. Full evidence for each candidate inversion group, separated into the classes shown in Figure 3 and Table 4. S11, H. cydno, split reads and trio assembly. [file EVL3-1-138-s012.pdf]
