# Supplementary material for: No evidence for maintenance of a sympatric Heliconius species barrier by chromosomal inversions
Source: Evol Lett. 2017 Jun 14;1(3):138–54. doi: 10.1002/evl3.12 (PMC6122123; doi:10.1002/evl3.12)

Figure S12.1

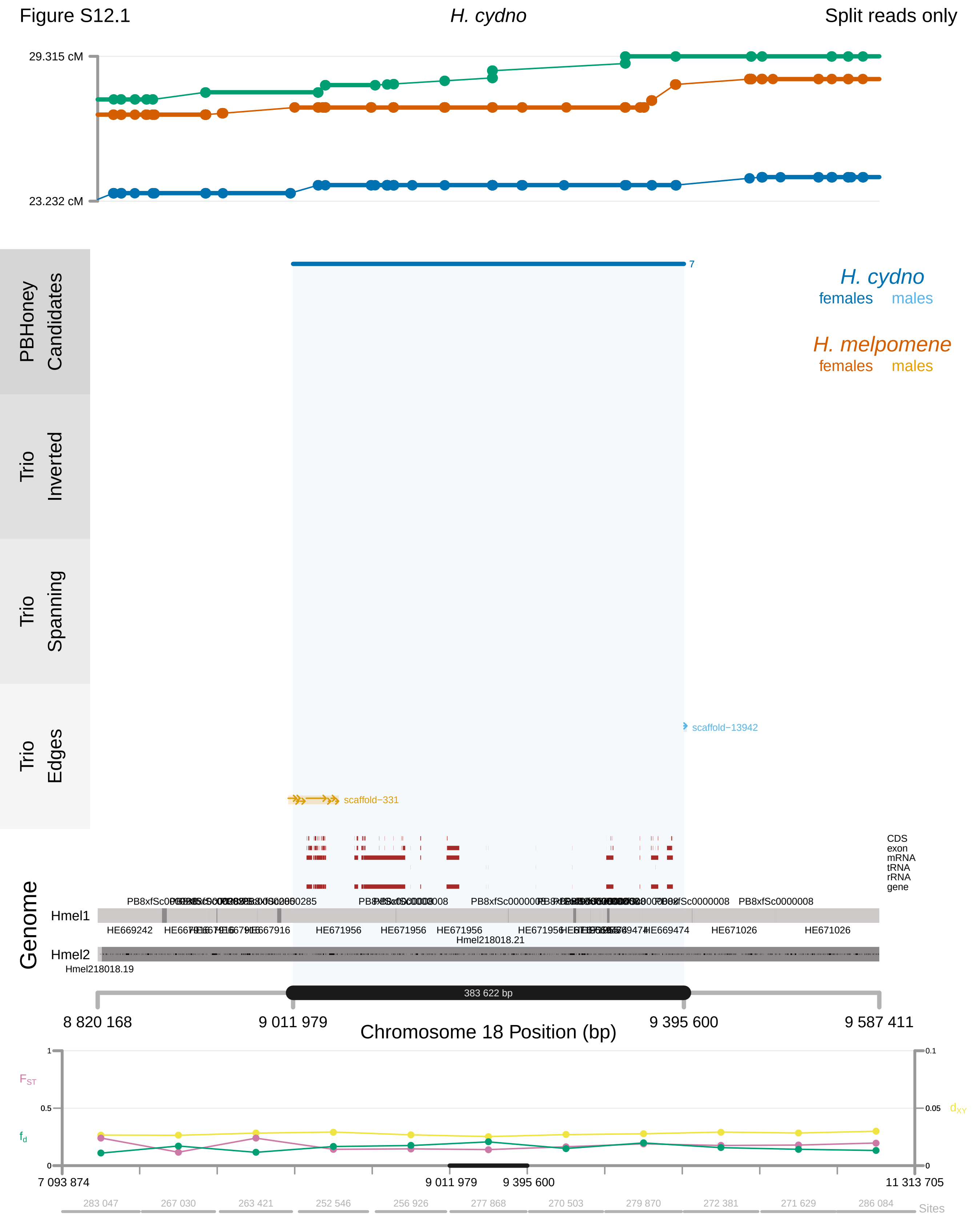
H. cydno
Split reads only
29.315 cM
23.232 cM
7
H. cydno
females
males
H. melpomene
females
males
PBHoney Candidates
Trio Inverted
Trio Spanning
Trio Edges
scaffold−13942
scaffold−331
CDS
exon
mRNA
tRNA
rRNA
gene
Genome
Hmel1
Hmel2
PB8xfSc0000008
HE669242
HE671956
HE671026
HE669474
Hmel218018.21
Hmel218018.19
383 622 bp
8 820 168
9 011 979
9 395 600
9 587 411
Chromosome 18 Position (bp)
FST
fd
dXY
1
0.5
0
0.1
0.05
7 093 874
11 313 705
283 047
267 030
263 421
252 546
256 926
277 868
270 503
279 870
272 381
271 629
286 084
Sites

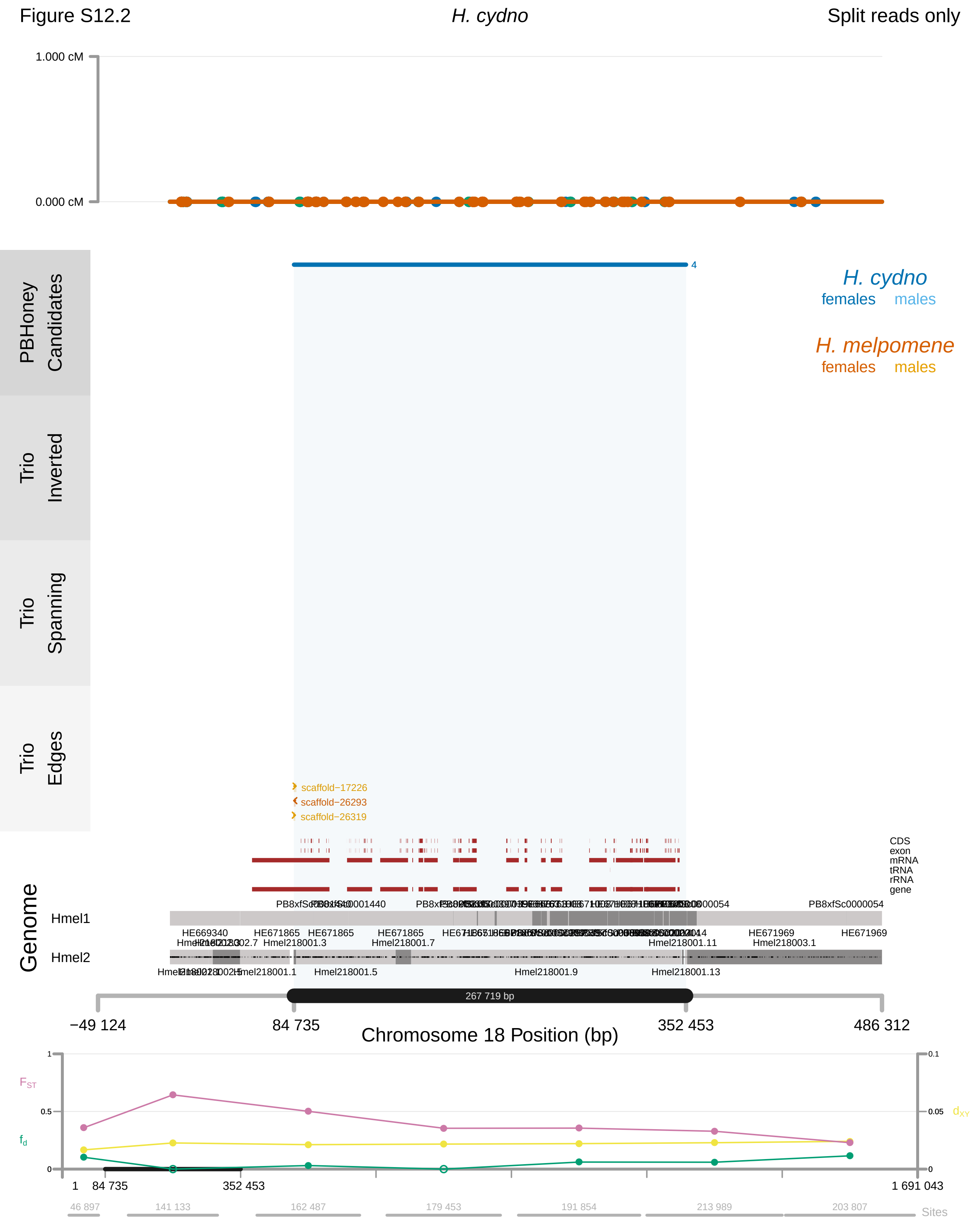
Figure S12.2
H. cydno
Split reads only
1.000 cM
0.000 cM
PBHoney Candidates
Trio Inverted
Trio Spanning
Trio Edges
H. cydno
females males
H. melpomene
females males
scaffold−17226
scaffold−26293
scaffold−26319
CDS
exon
mRNA
tRNA
rRNA
gene
Genome
Hmel1
Hmel2
PB8xfSc0000054
HE669340
HE671865
HE671969
Hmel218001.3
Hmel218001.7
Hmel218001.11
Hmel218003.1
Hmel218001.1
Hmel218001.5
Hmel218001.9
Hmel218001.13
267 719 bp
−49 124
84 735
352 453
486 312
Chromosome 18 Position (bp)
F_ST
f_d
d_XY
Sites
1
1 691 043
46 897
141 133
162 487
179 453
191 854
213 989
203 807

Figure S12.3 *H. cydno* Split reads only

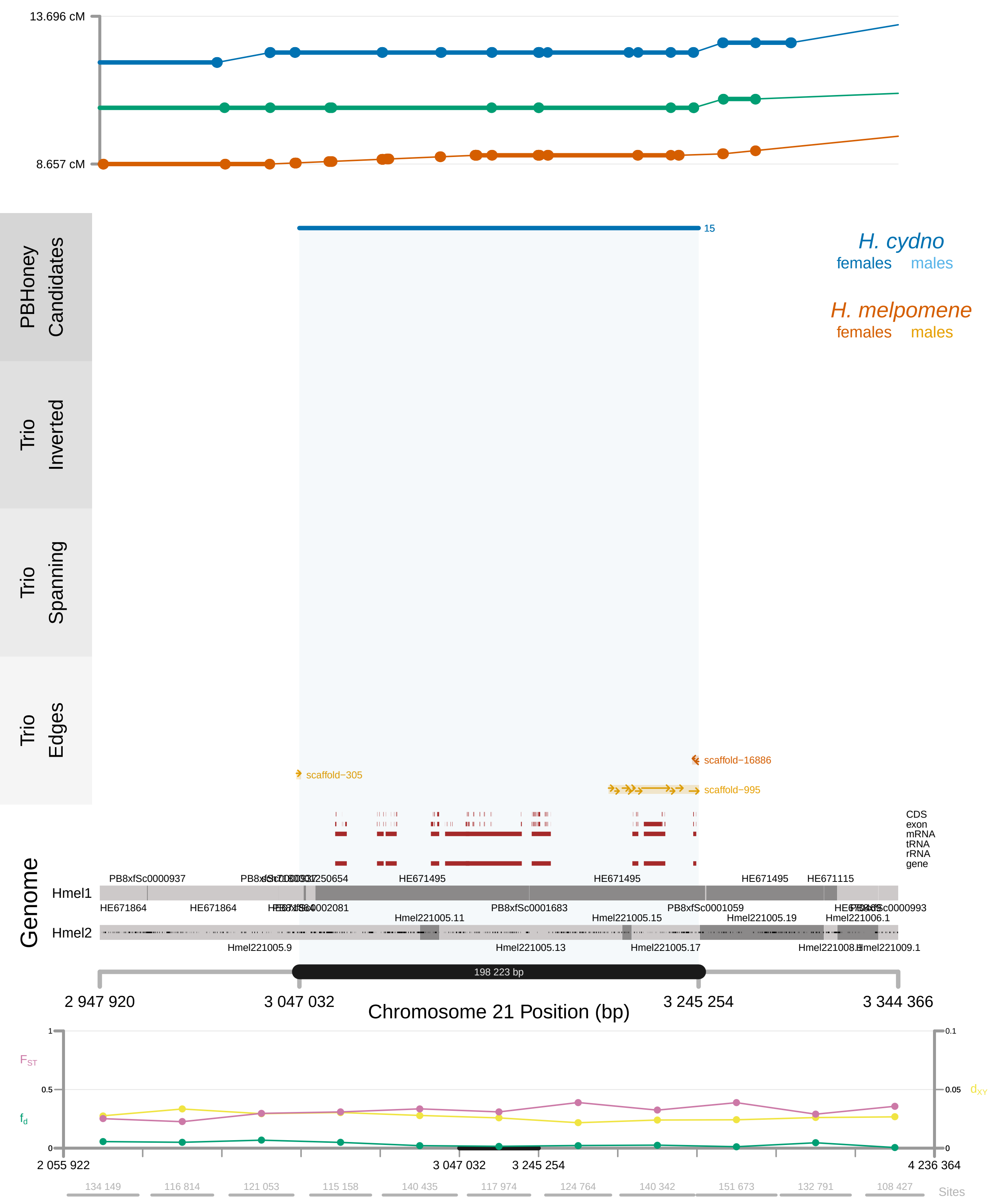

Figure S12.4 *H. cydno* Split reads only

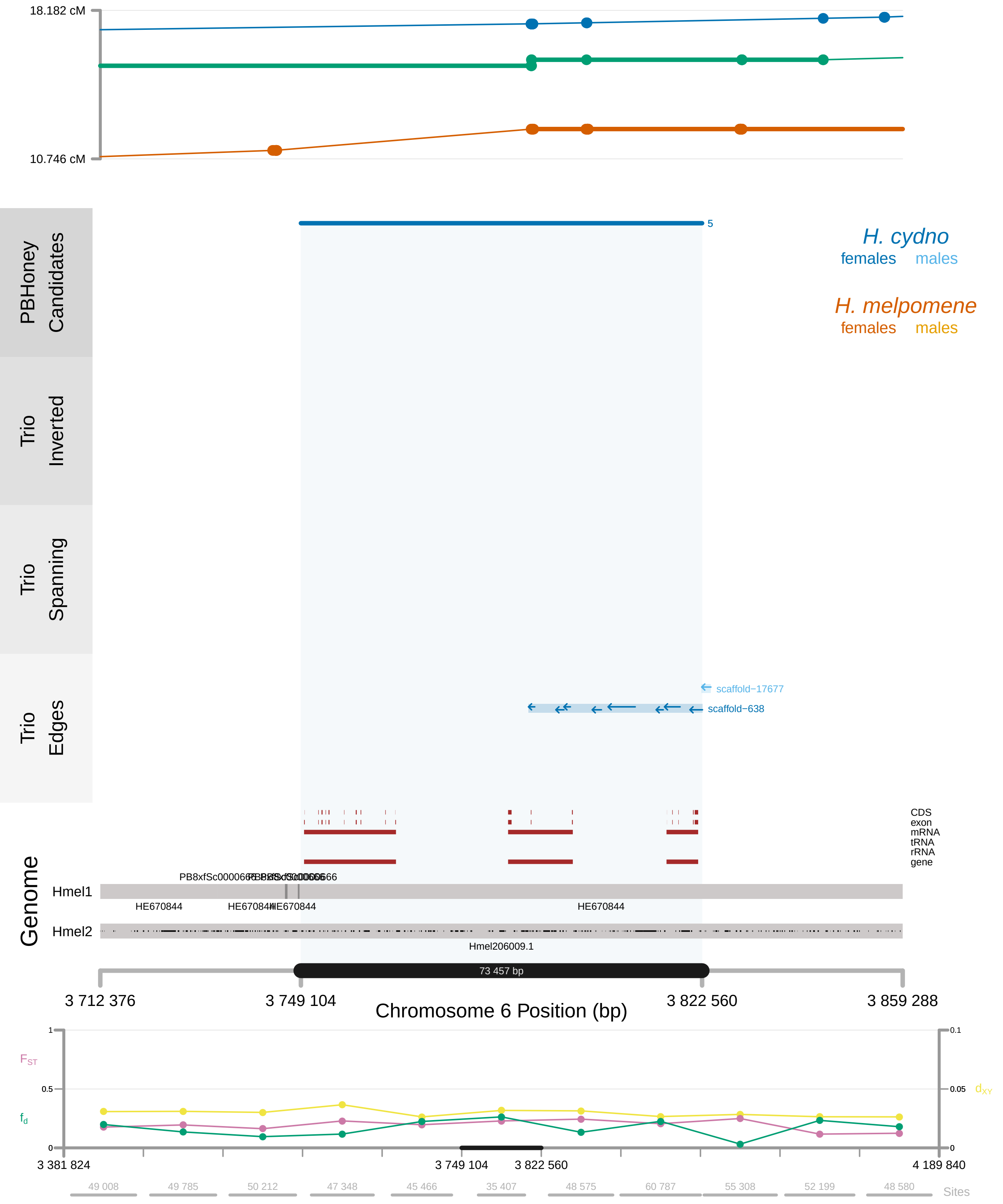

Figure S12.5 *H. cydno* Split reads only

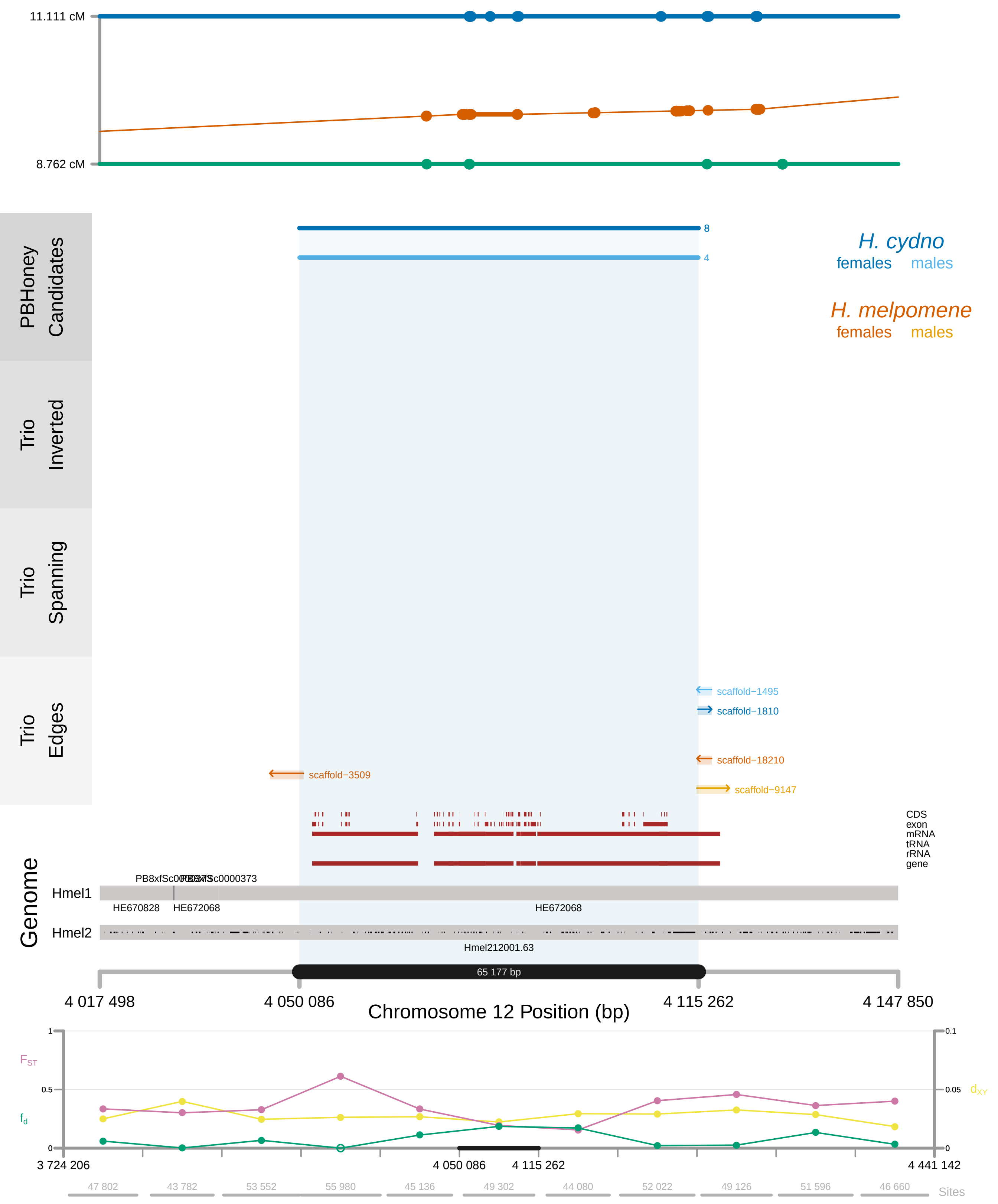

Figure S12.6

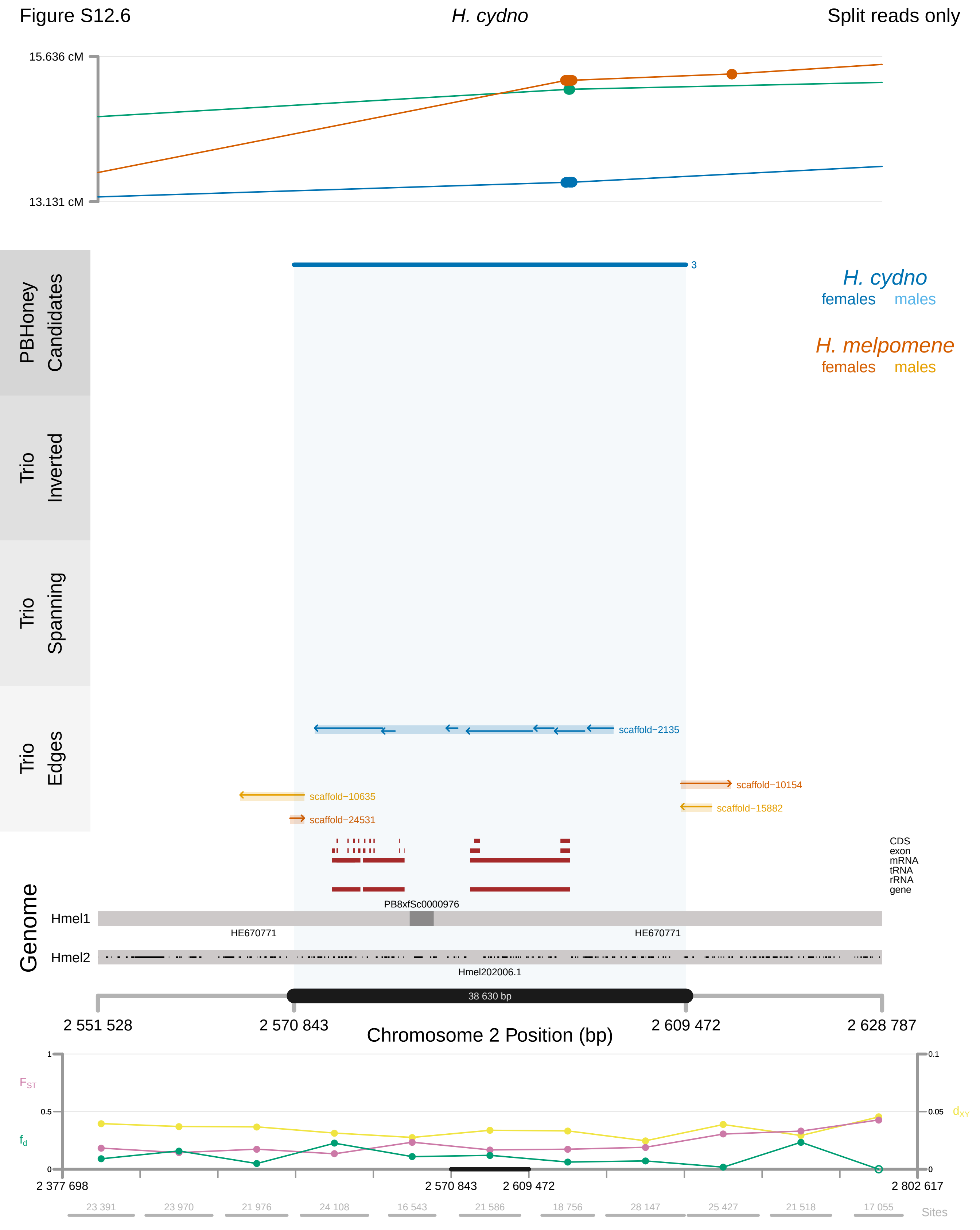
H. cydno
Split reads only
15.636 cM
13.131 cM
PBHoney Candidates
Trio Inverted
Trio Spanning
Trio Edges
3
H. cydno
females males
H. melpomene
females males
scaffold−2135
scaffold−10154
scaffold−10635
scaffold−15882
scaffold−24531
CDS
exon
mRNA
tRNA
rRNA
gene
Genome
PB8xfSc0000976
Hmel1
HE670771
HE670771
Hmel2
Hmel202006.1
38 630 bp
2 551 528
2 570 843
2 609 472
2 628 787
Chromosome 2 Position (bp)
1
0.5
0
F_{ST}
f_d
0.1
0.05
0
d_{XY}
2 377 698
2 570 843
2 609 472
2 802 617
23 391
23 970
21 976
24 108
16 543
21 586
18 756
28 147
25 427
21 518
17 055
Sites

Figure S12.7

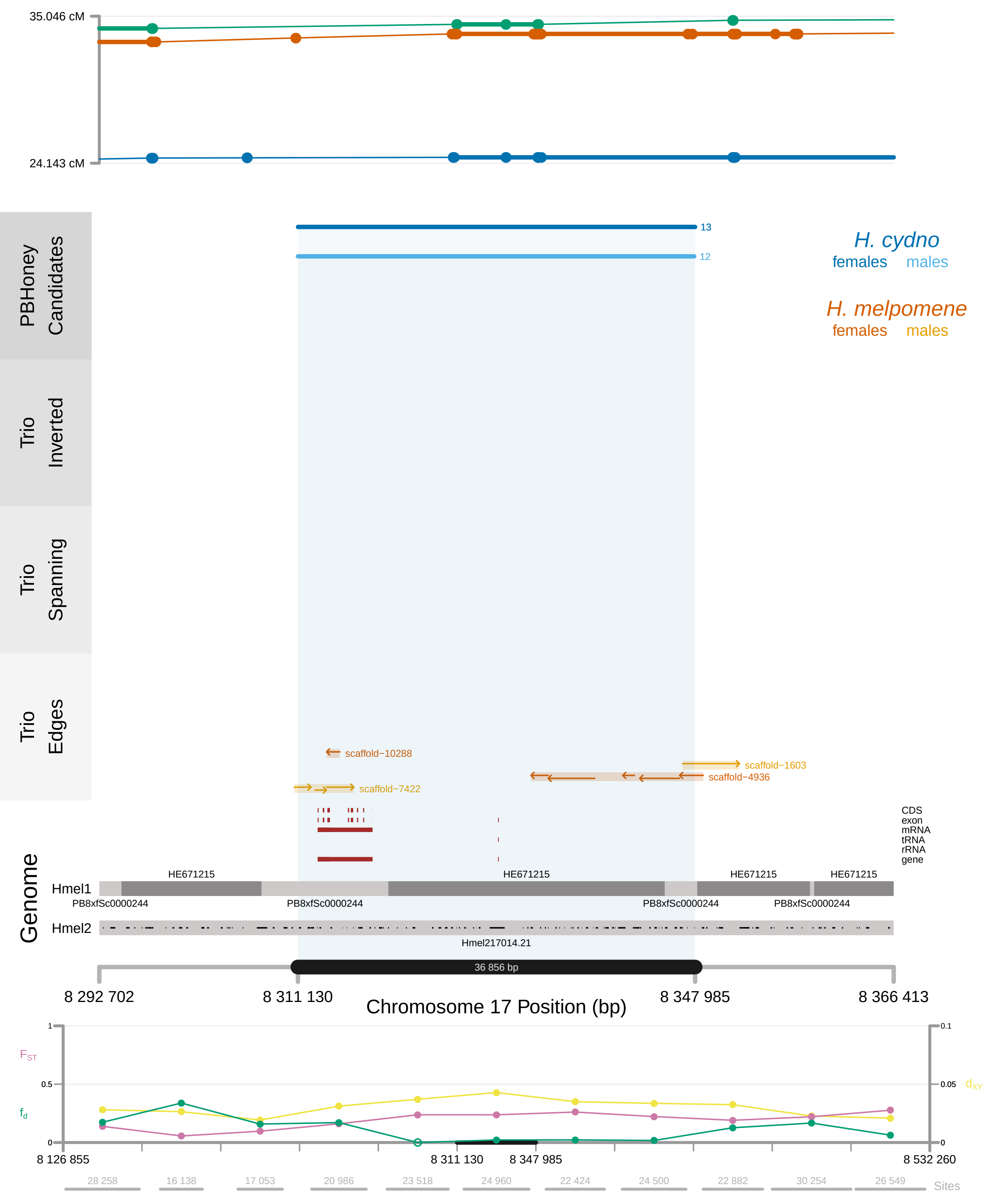

H. cydno
Split reads only
35.046 cM
24.143 cM
PBHoney Candidates
Trio Inverted
Trio Spanning
Trio Edges
Genome
13
12
H. cydno
females
males
H. melpomene
females
males
scaffold−10288
scaffold−7422
scaffold−1603
scaffold−4936
CDS
exon
mRNA
tRNA
rRNA
gene
HE671215
Hmel1
PB8xfSc0000244
Hmel2
Hmel217014.21
36 856 bp
8 292 702
8 311 130
8 347 985
8 366 413
Chromosome 17 Position (bp)
1
0.5
0
F_{ST}
f_d
0.1
0.05
d_{XY}
8 126 855
8 311 130
8 347 985
8 532 260
28 258
16 138
17 053
20 986
23 518
24 960
22 424
24 500
22 882
30 254
26 549
Sites

Figure S12.8 *H. cydno* Split reads only

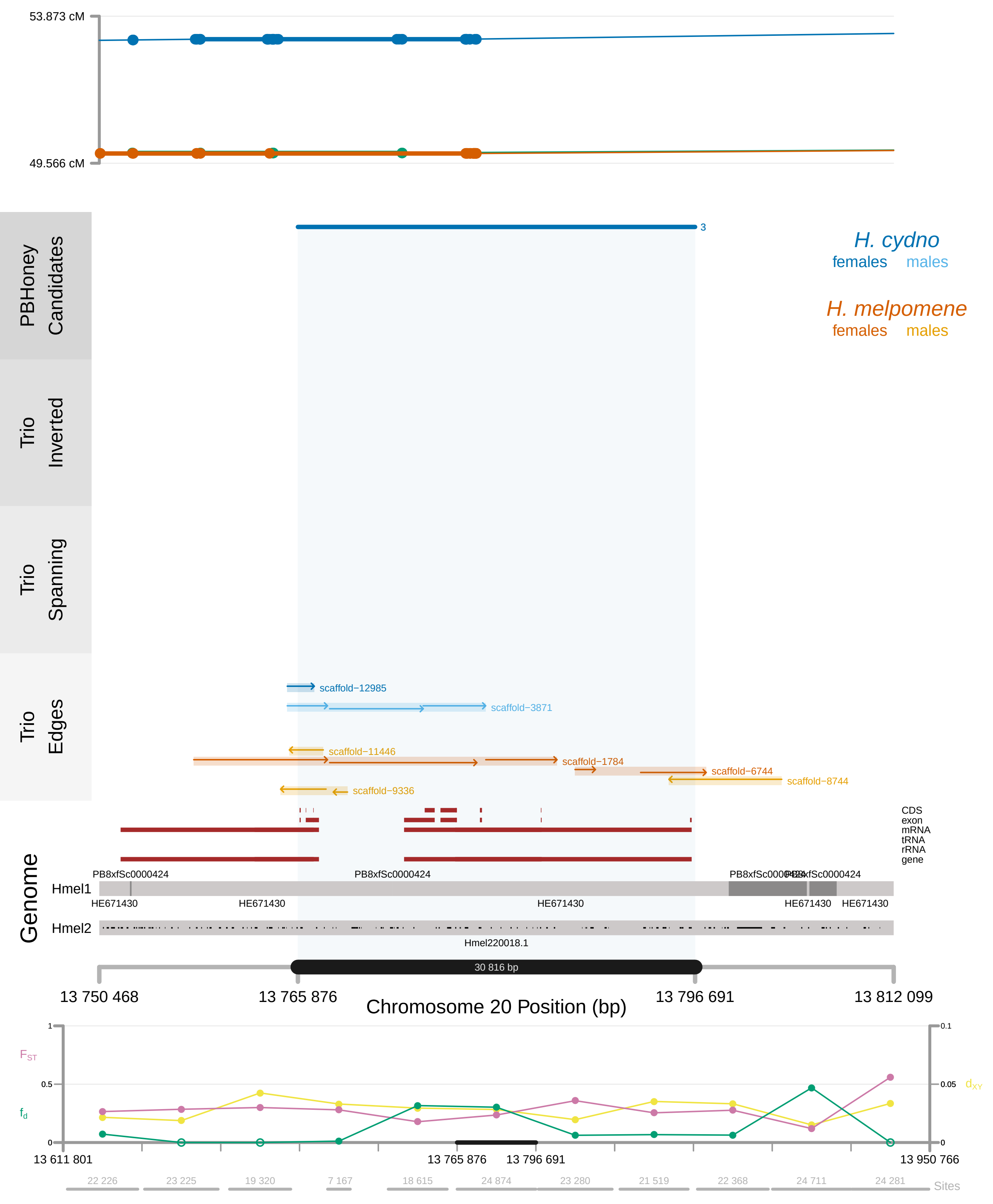

Figure S12.9 *H. cydno* Split reads only

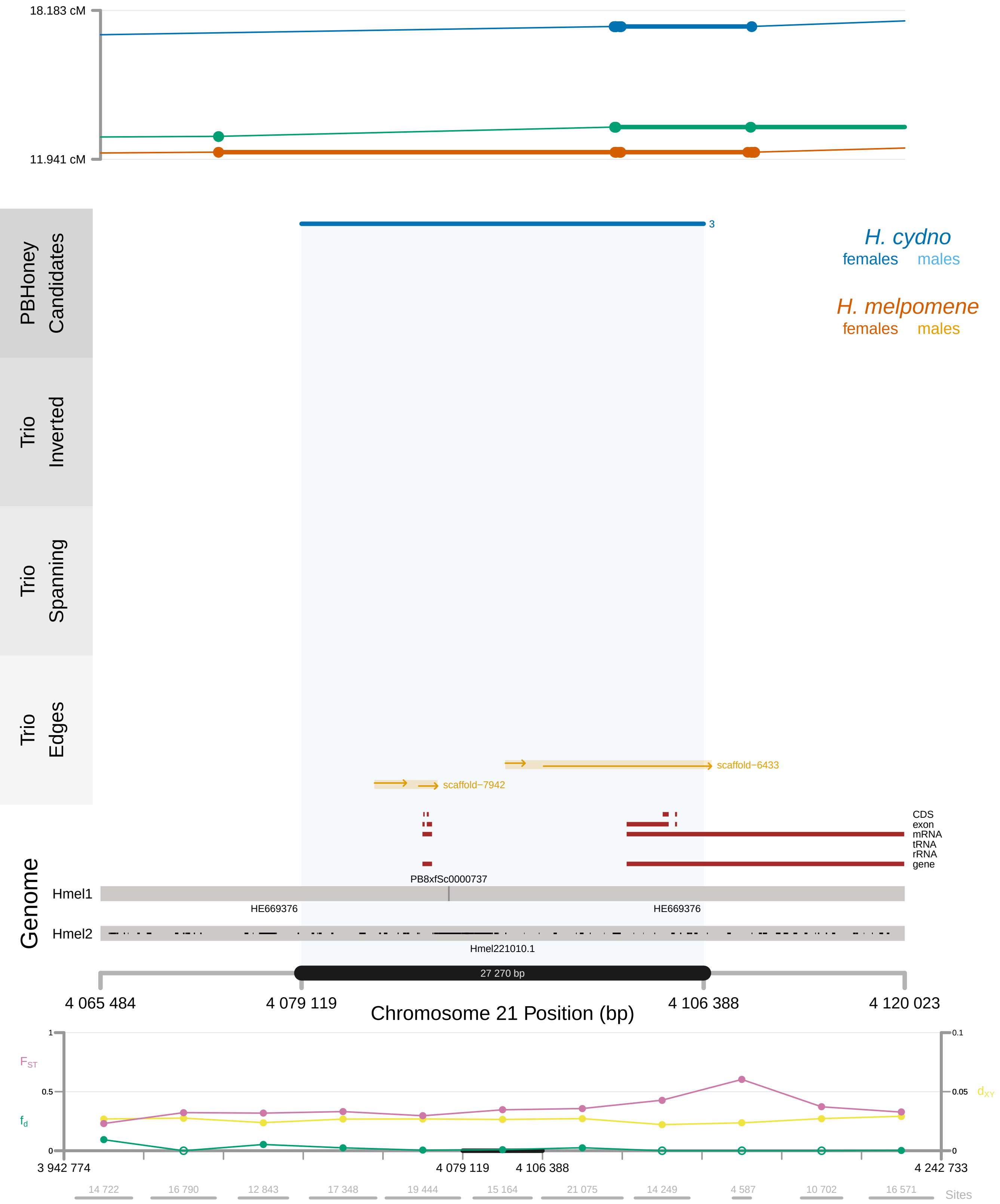

Figure S12.10

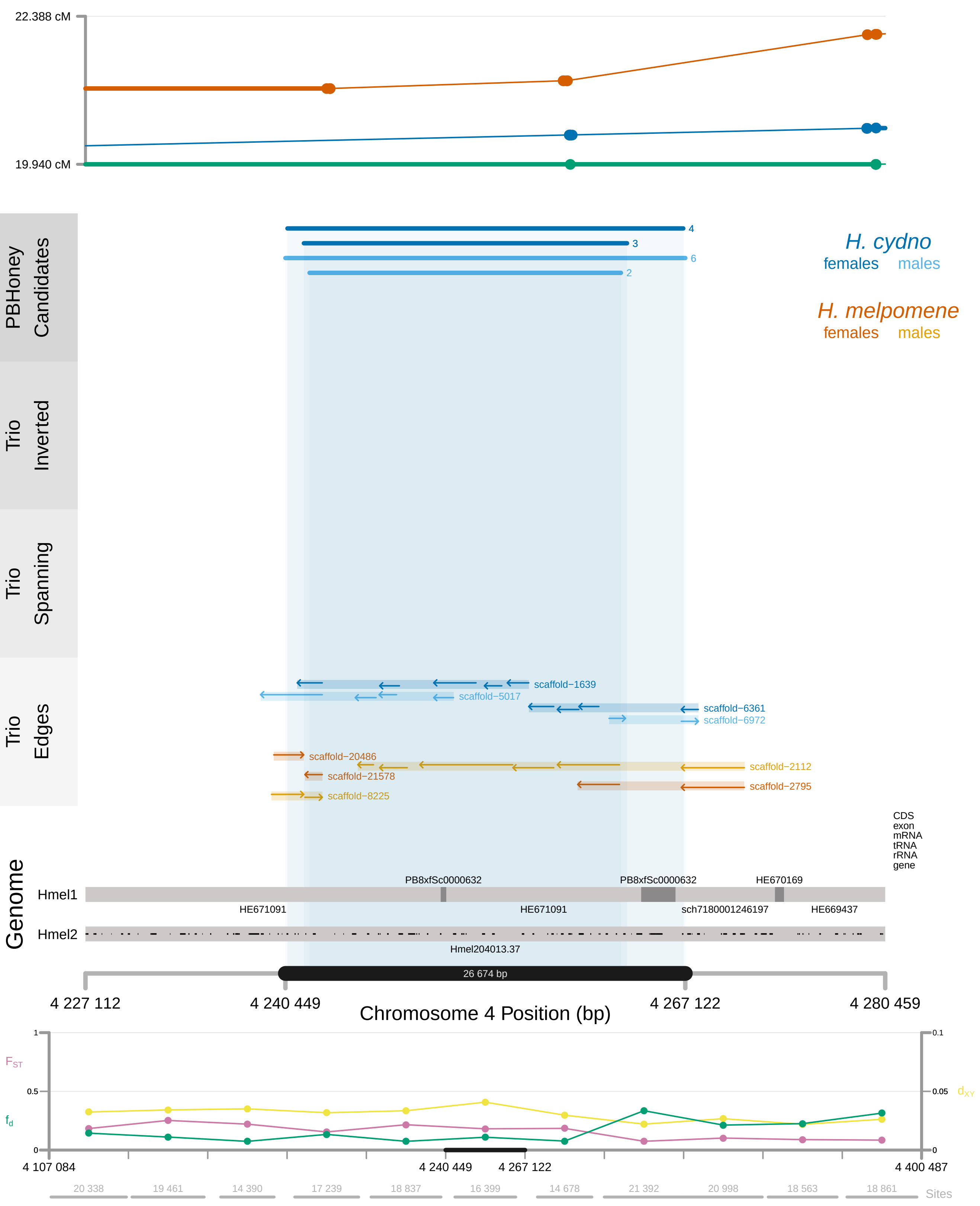

Figure S12.11

H. cydno

Split reads only

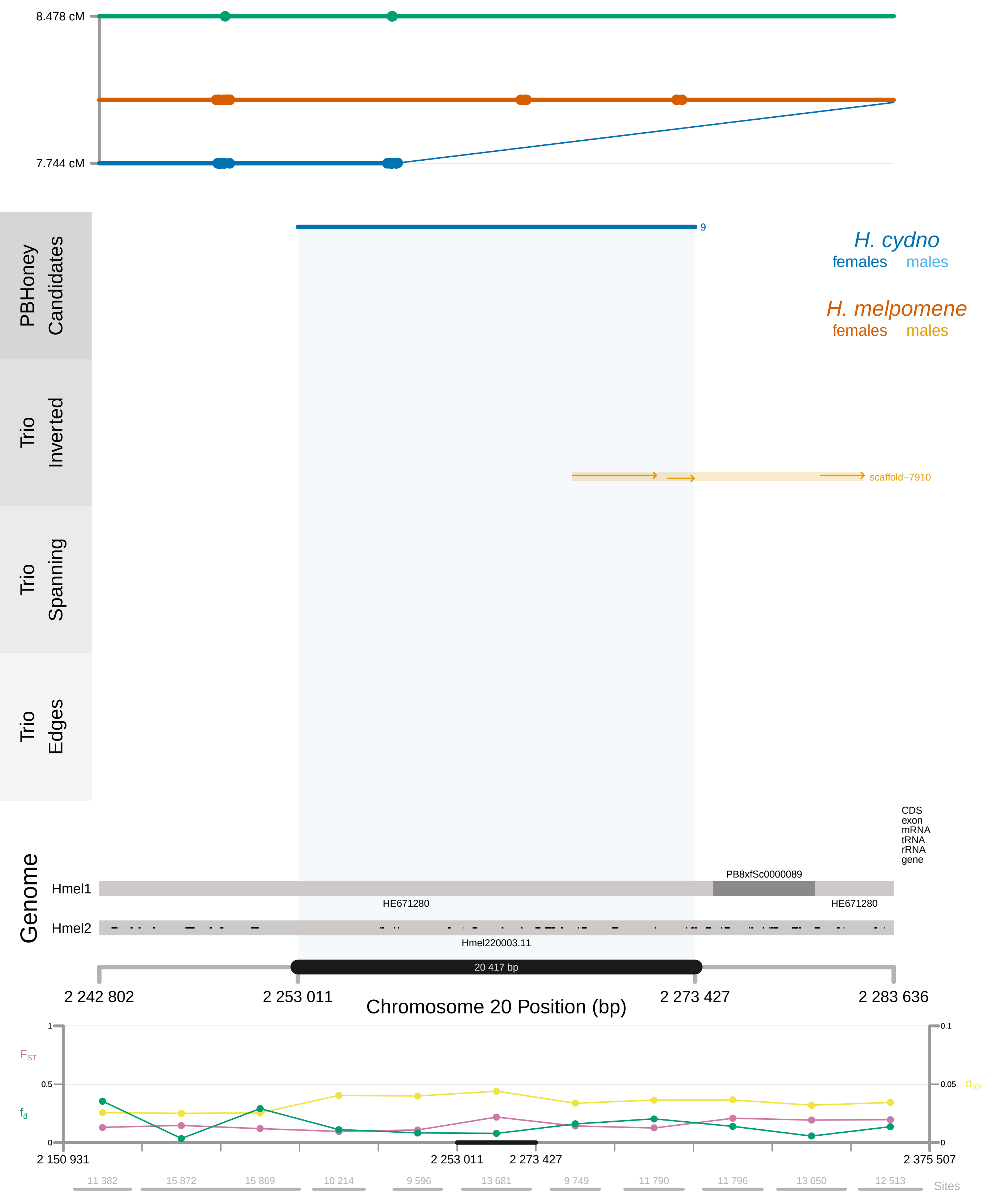

Figure S12.12

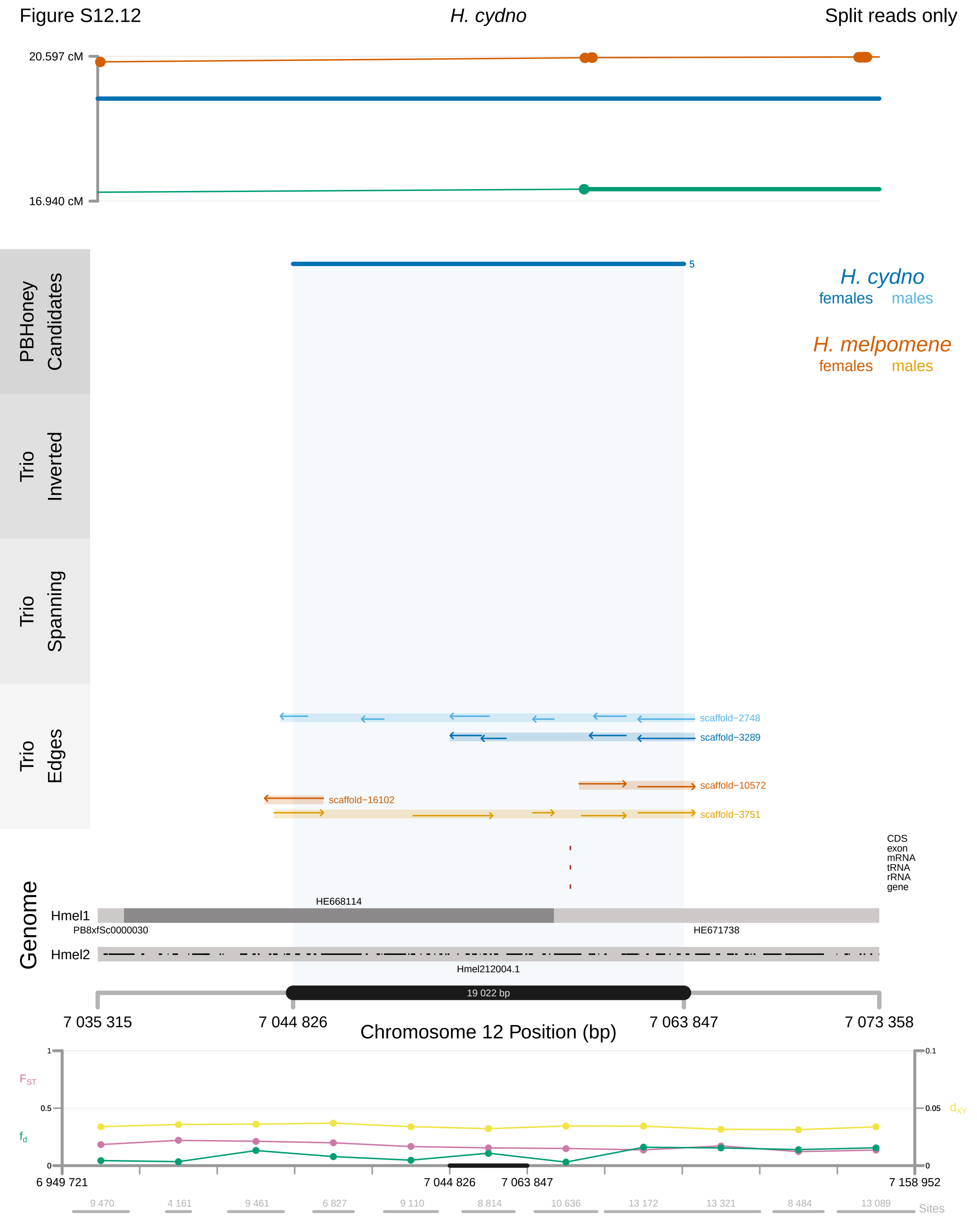

Figure S12.13

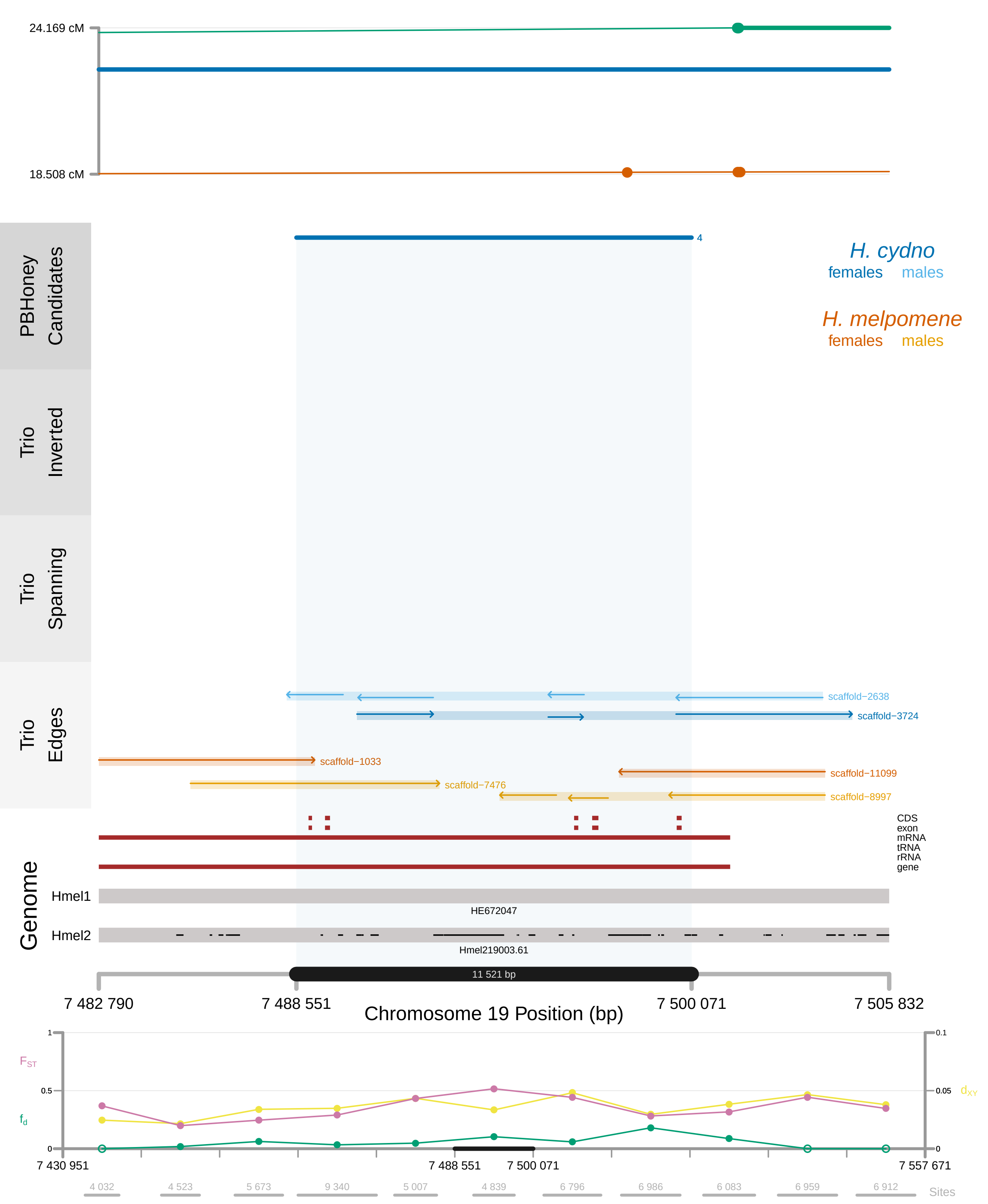

Figure S12.14

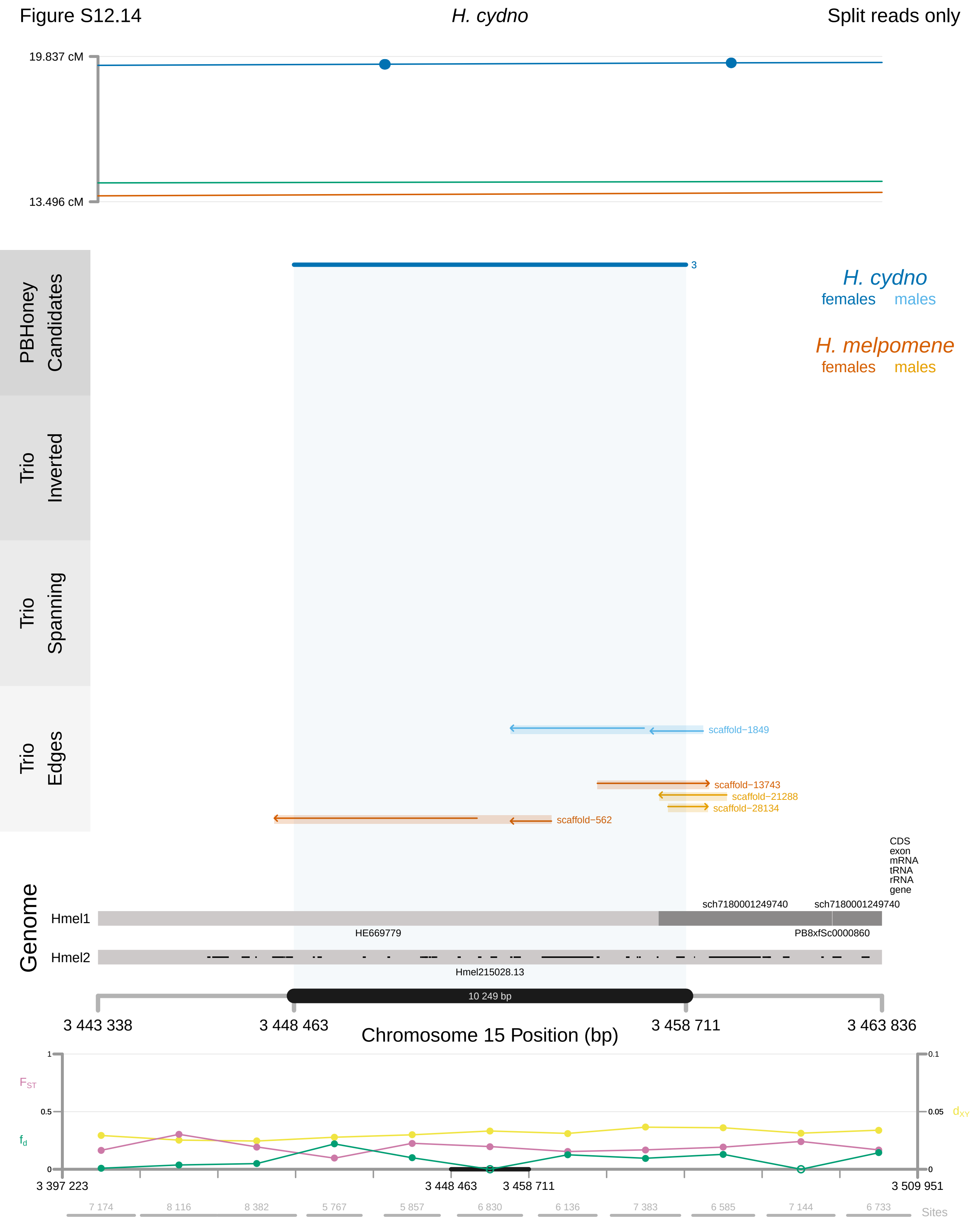

Figure S12.15 *H. cydno* Split reads only

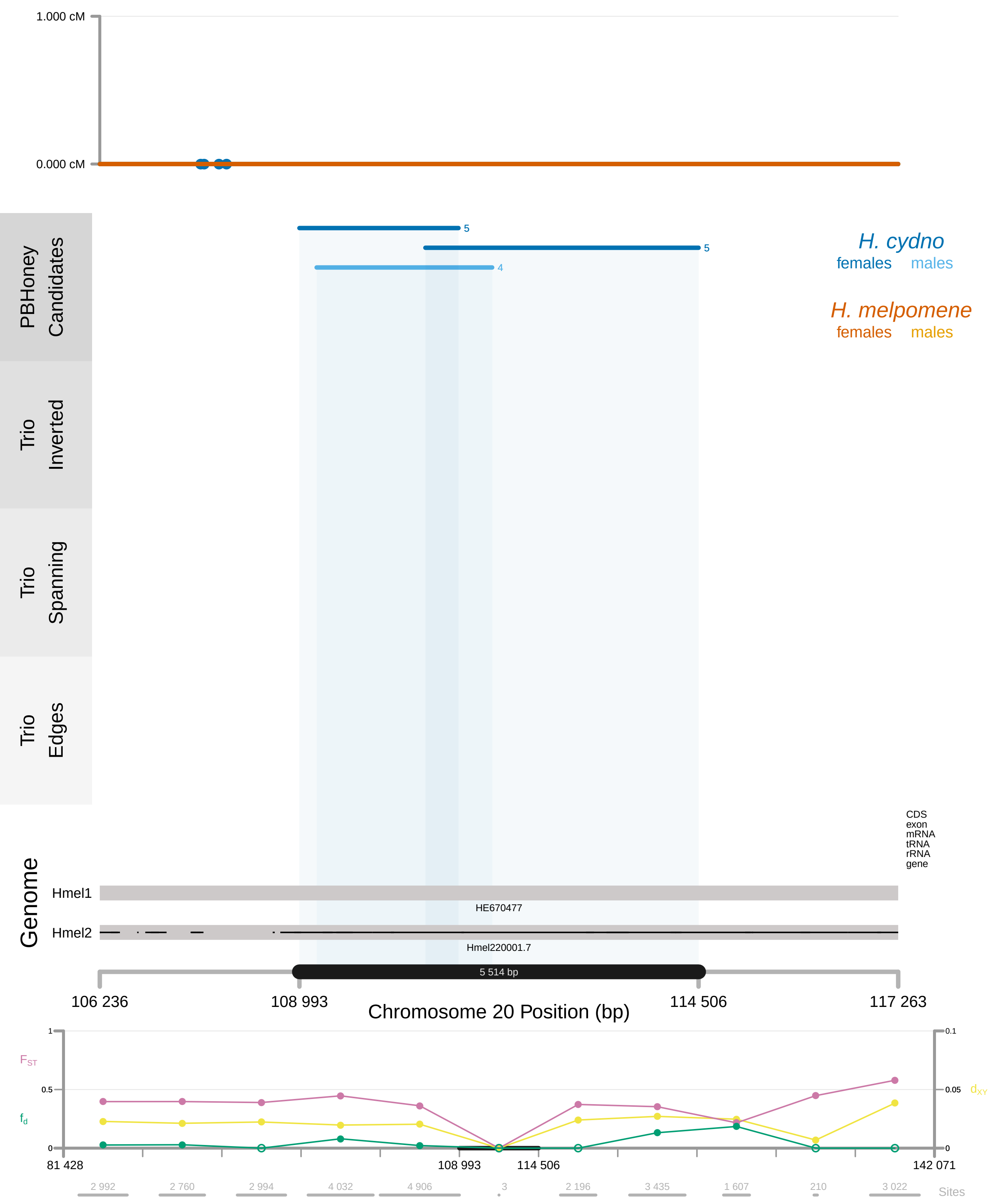

Figure S12.16

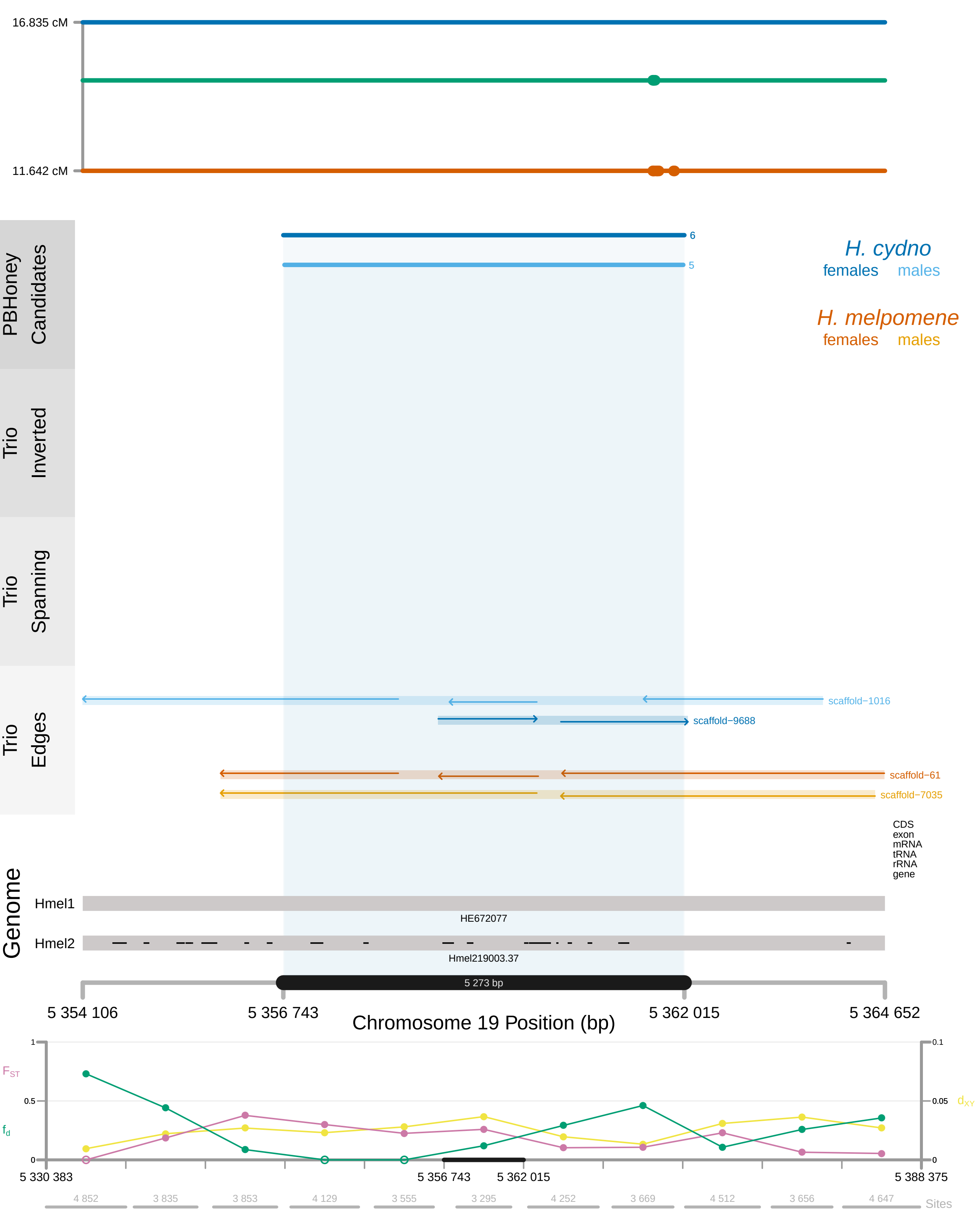

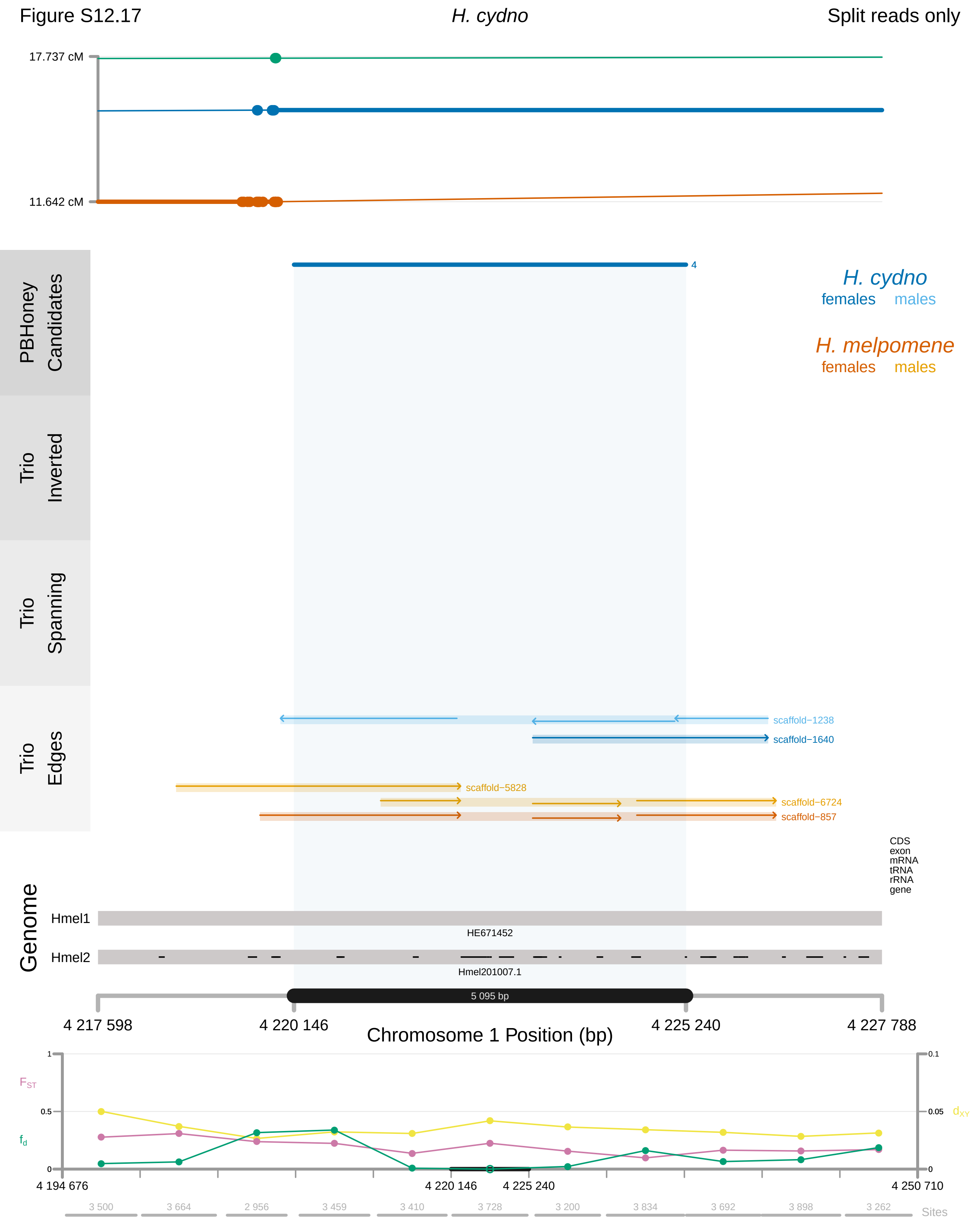

Figure S12.17
H. cydno
Split reads only
17.737 cM
11.642 cM
PBHoney Candidates
Trio Inverted
Trio Spanning
Trio Edges
Genome
4
H. cydno
females
males
H. melpomene
females
males
scaffold−1238
scaffold−1640
scaffold−5828
scaffold−6724
scaffold−857
CDS
exon
mRNA
tRNA
rRNA
gene
Hmel1
HE671452
Hmel2
Hmel201007.1
5 095 bp
4 217 598
4 220 146
4 225 240
4 227 788
Chromosome 1 Position (bp)
1
0.5
0
F_{ST}
f_d
0.1
0.05
0
d_{XY}
4 194 676
4 220 146
4 225 240
4 250 710
3 500
3 664
2 956
3 459
3 410
3 728
3 200
3 834
3 692
3 898
3 262
Sites

Figure S12.18 *H. cydno* Split reads only

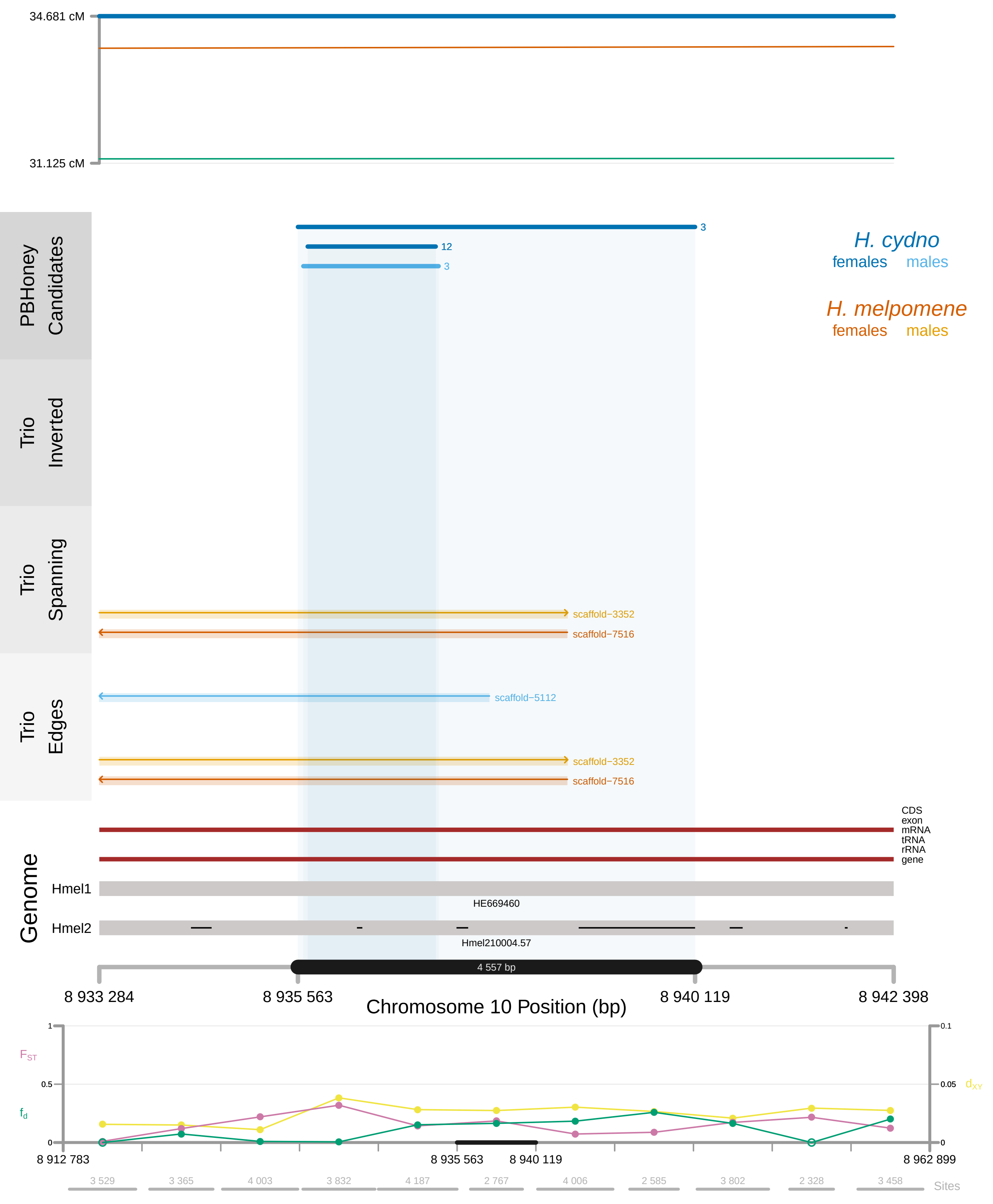

Figure S12.19 *H. cydno* Split reads only

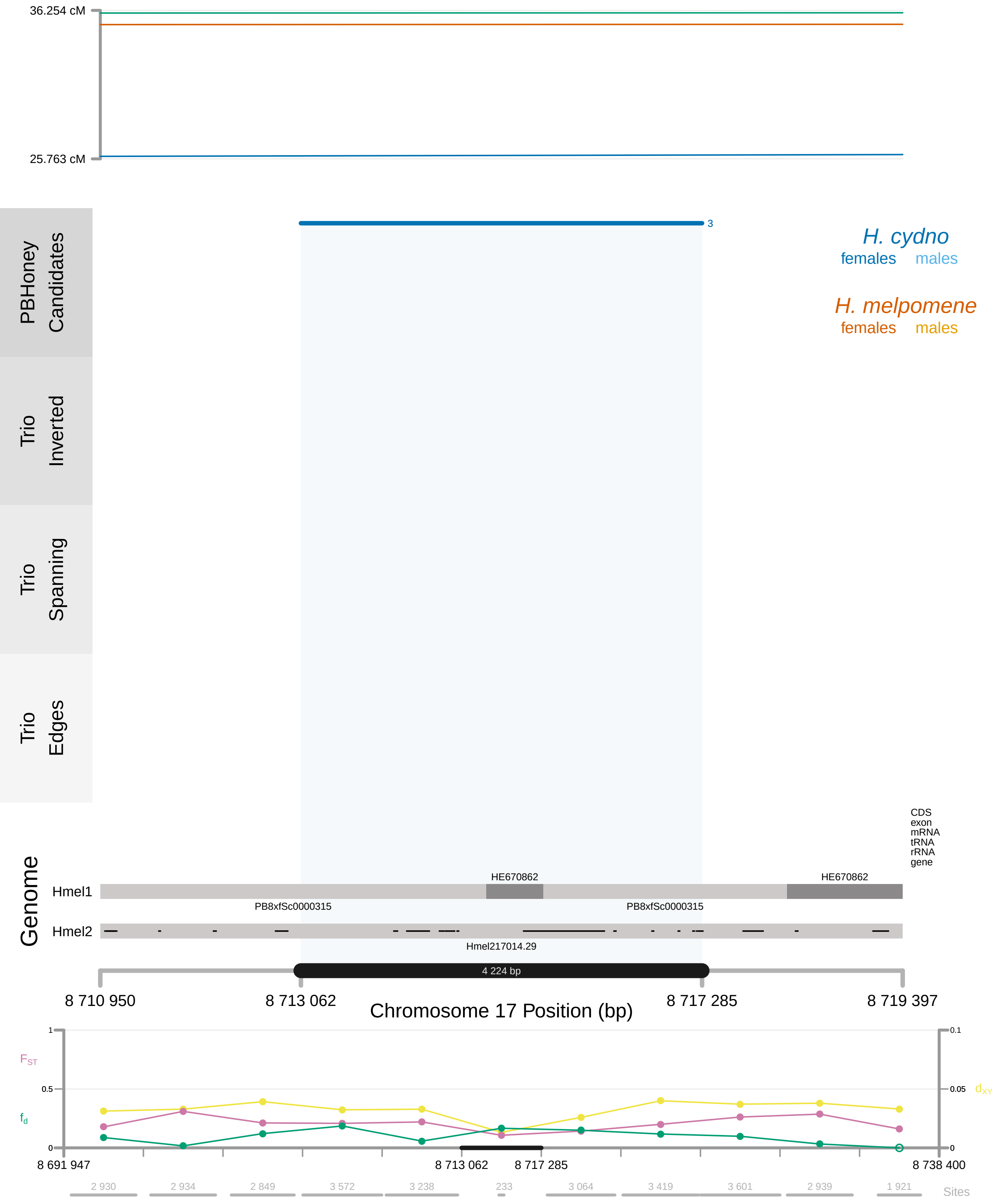

Figure S12.20 *H. cydno* Split reads only

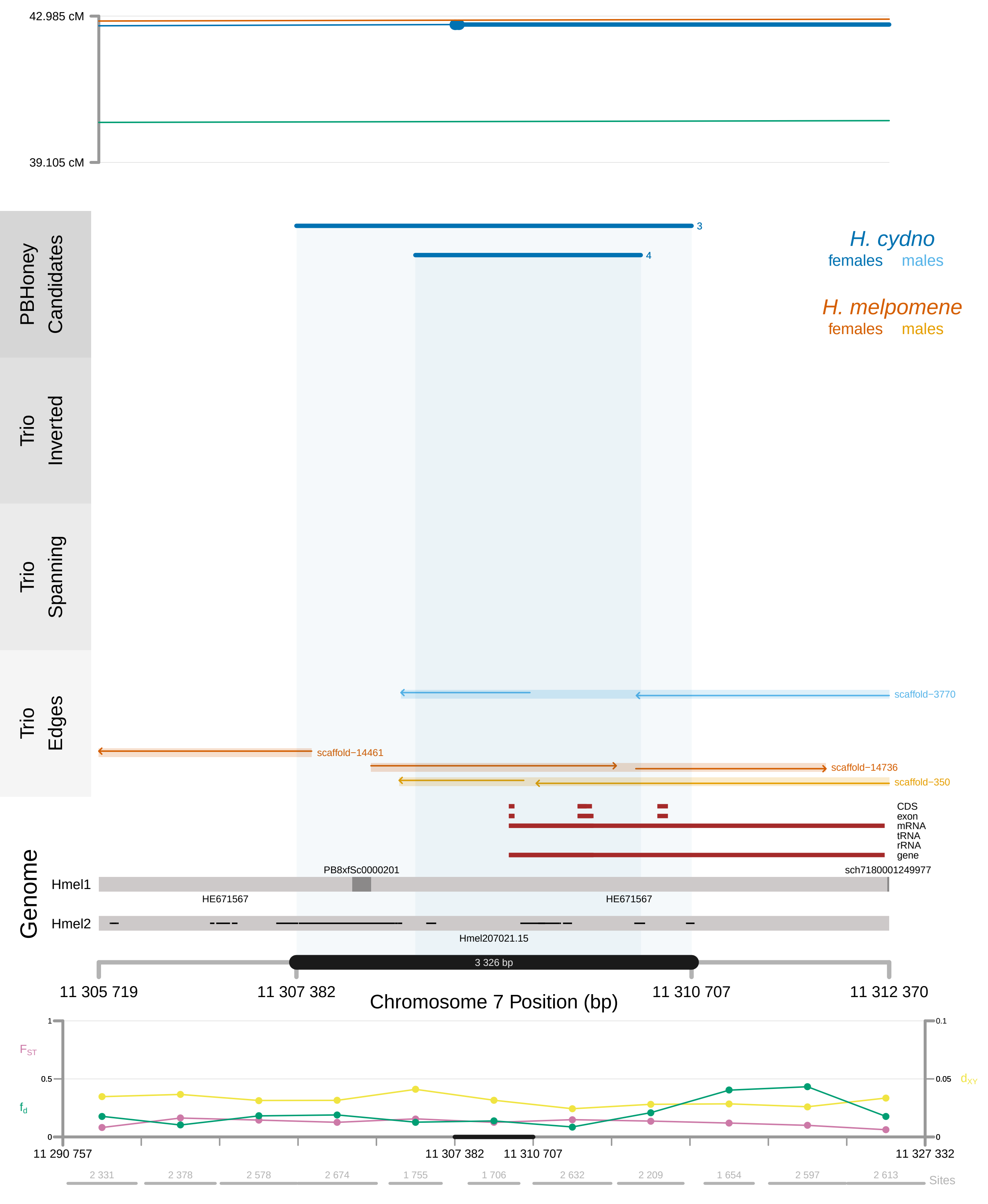

Figure S12.21 *H. cydno* Split reads only

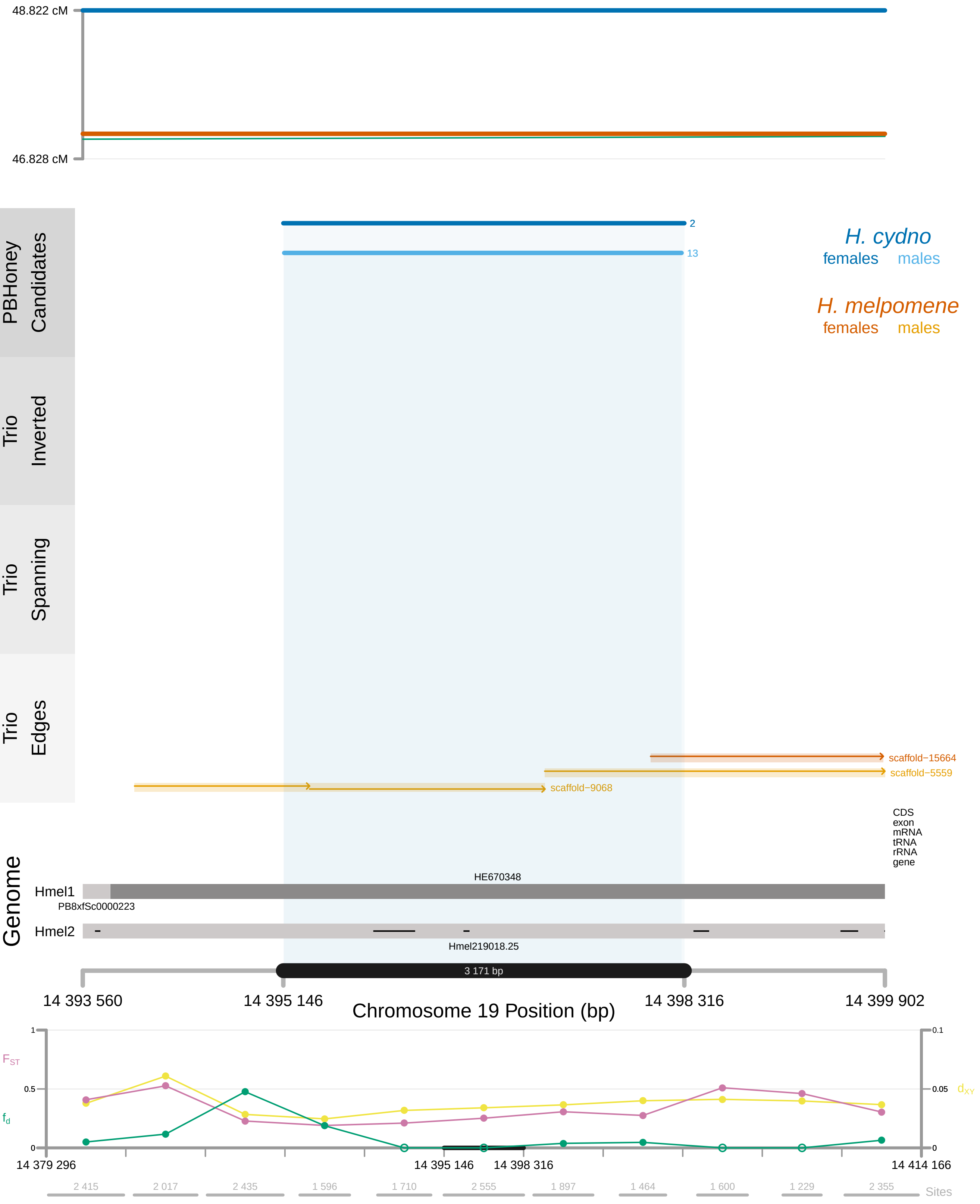

Figure S12.22 *H. cydno* Split reads only

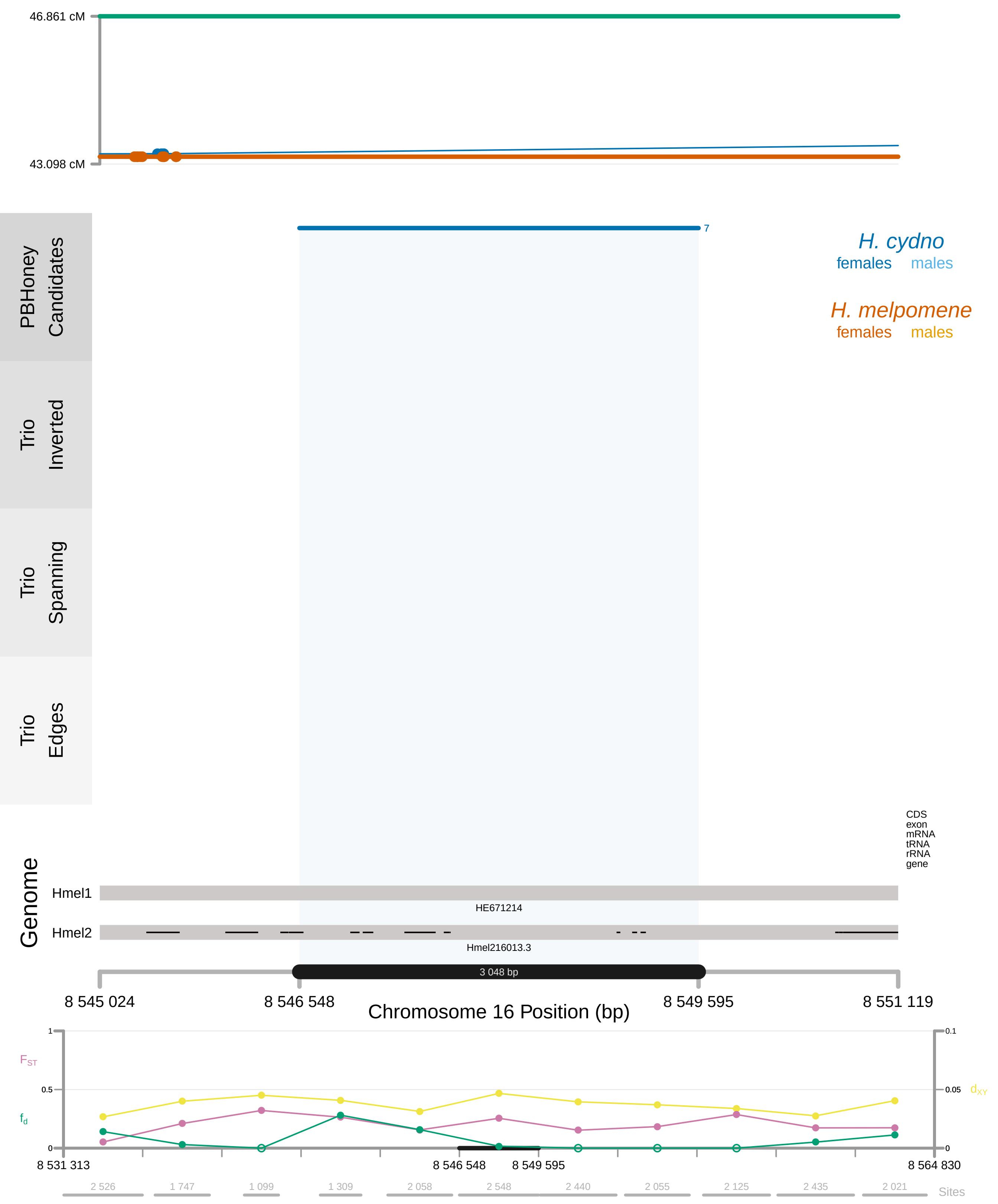

Figure S12.23 *H. cydno* Split reads only

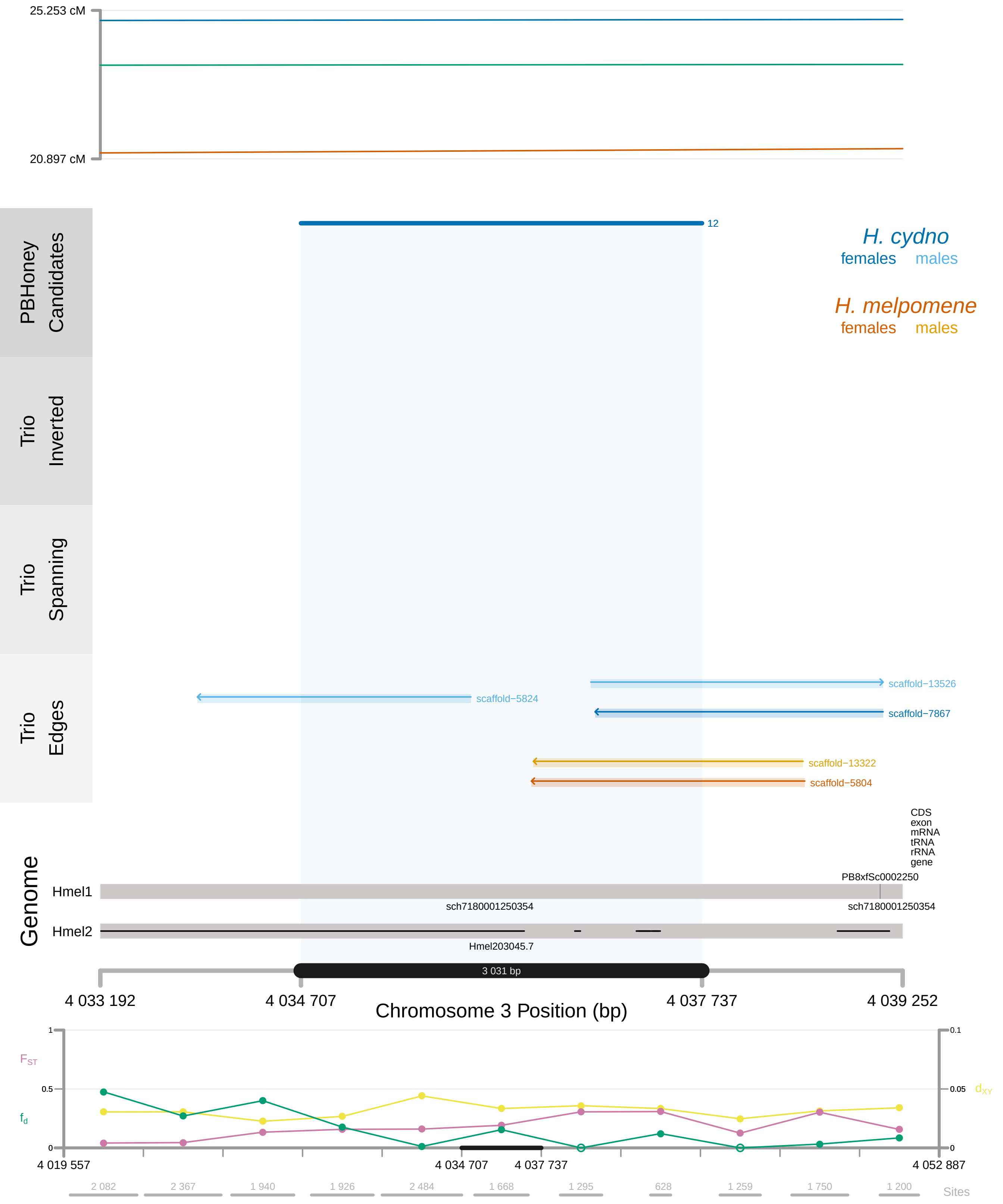

Figure S12.24 *H. cydno* Split reads only

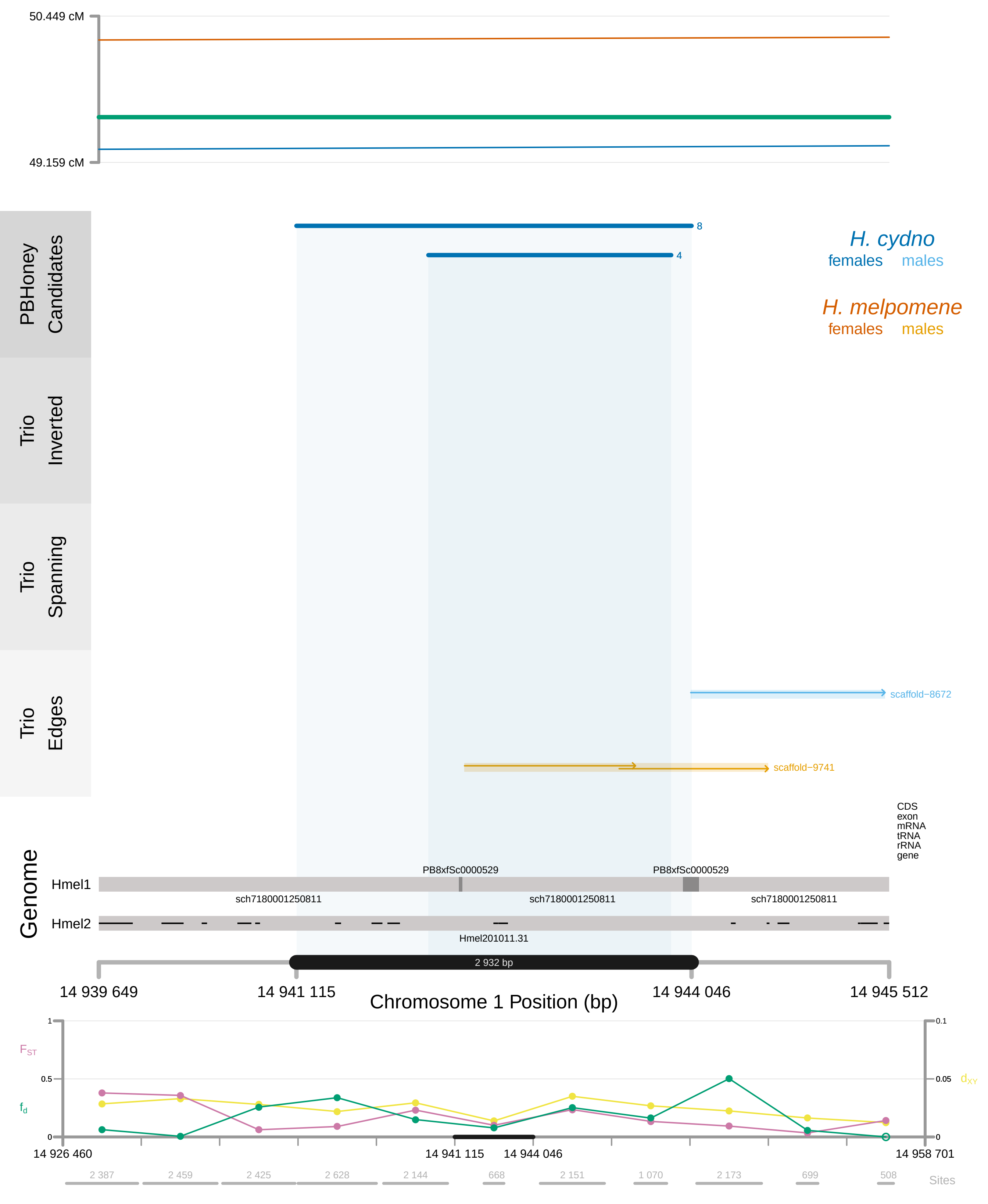

Figure S12.25 *H. cydno* Split reads only

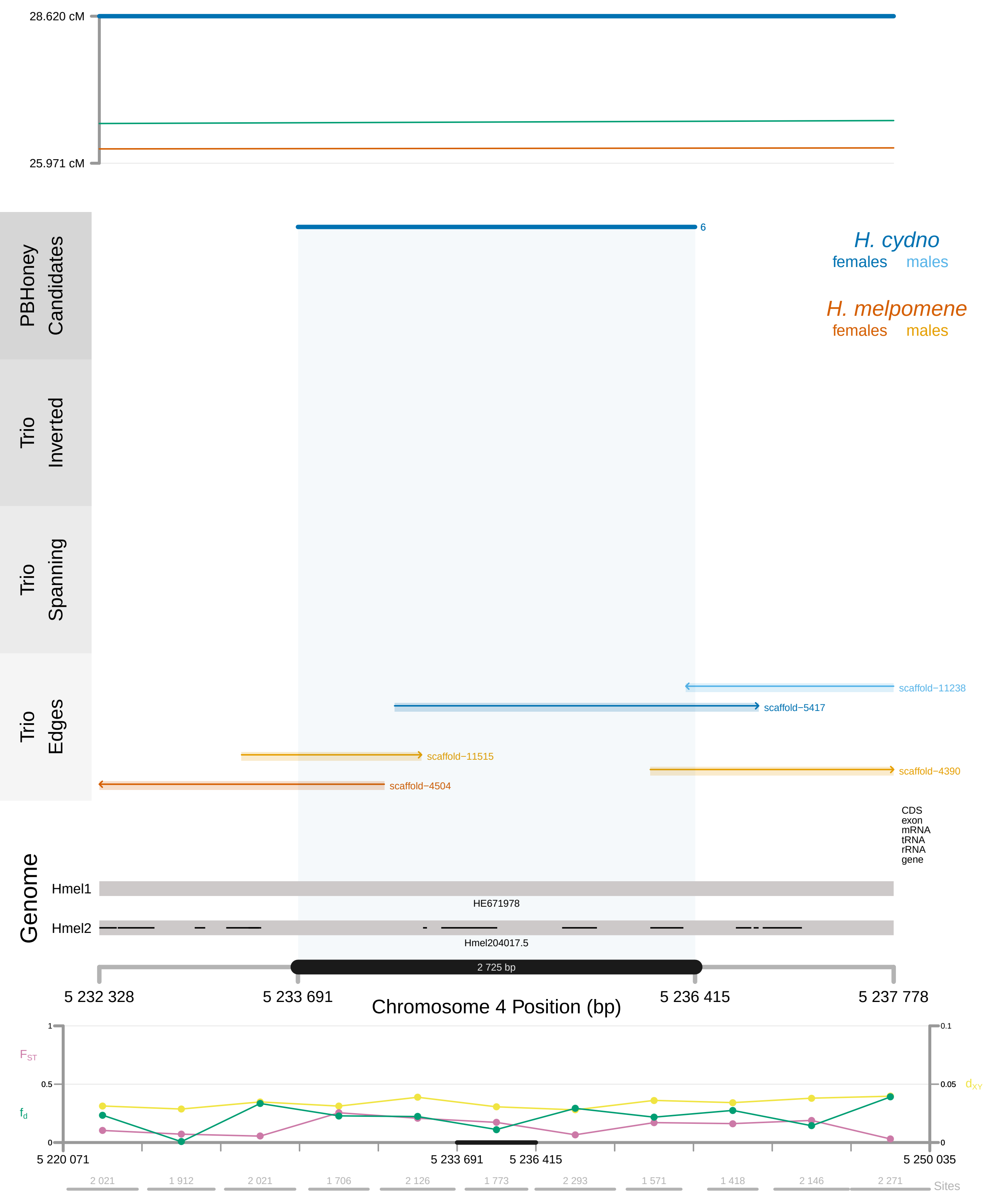

Figure S12.26 *H. cydno* Split reads only

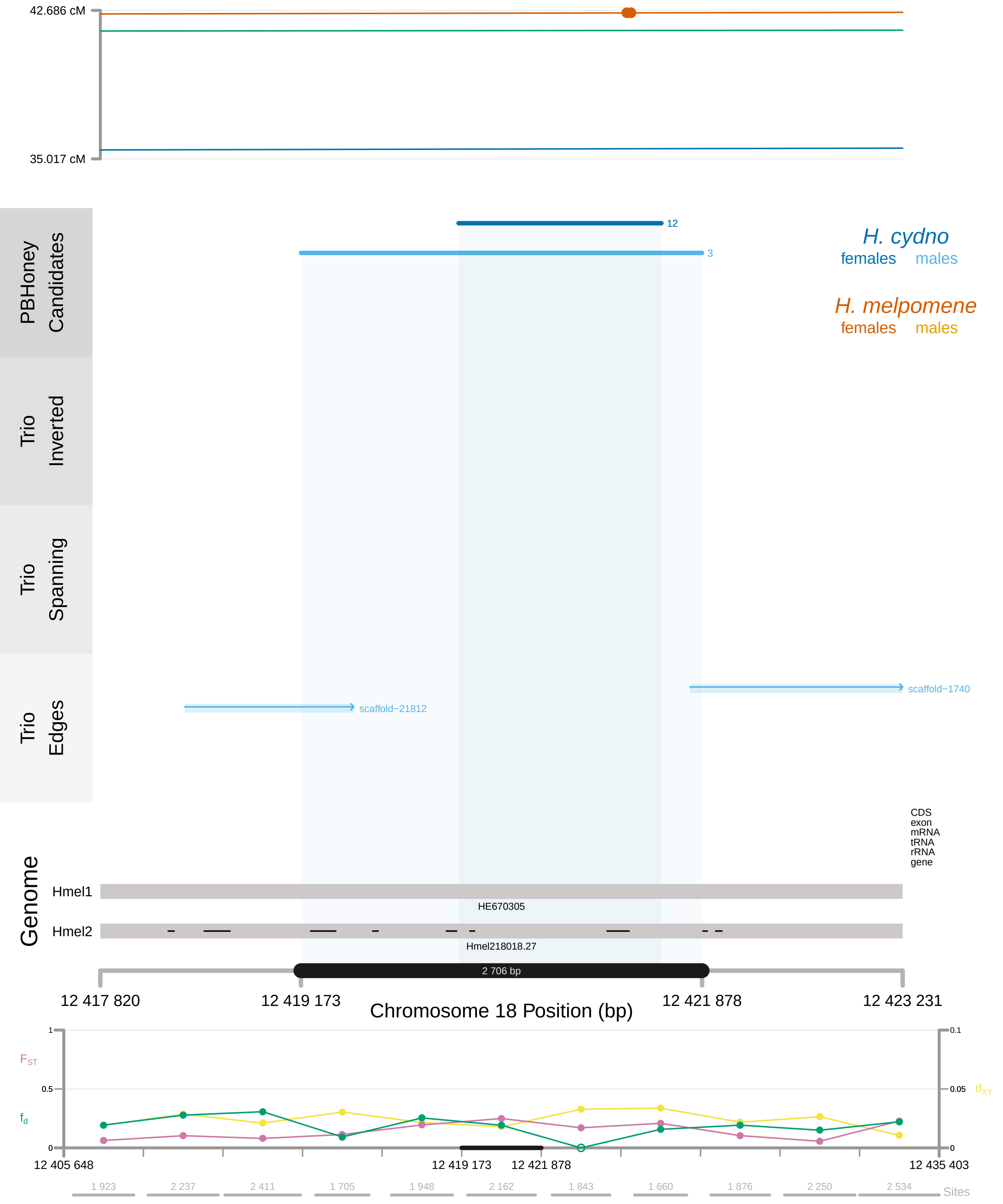

Figure S12.27

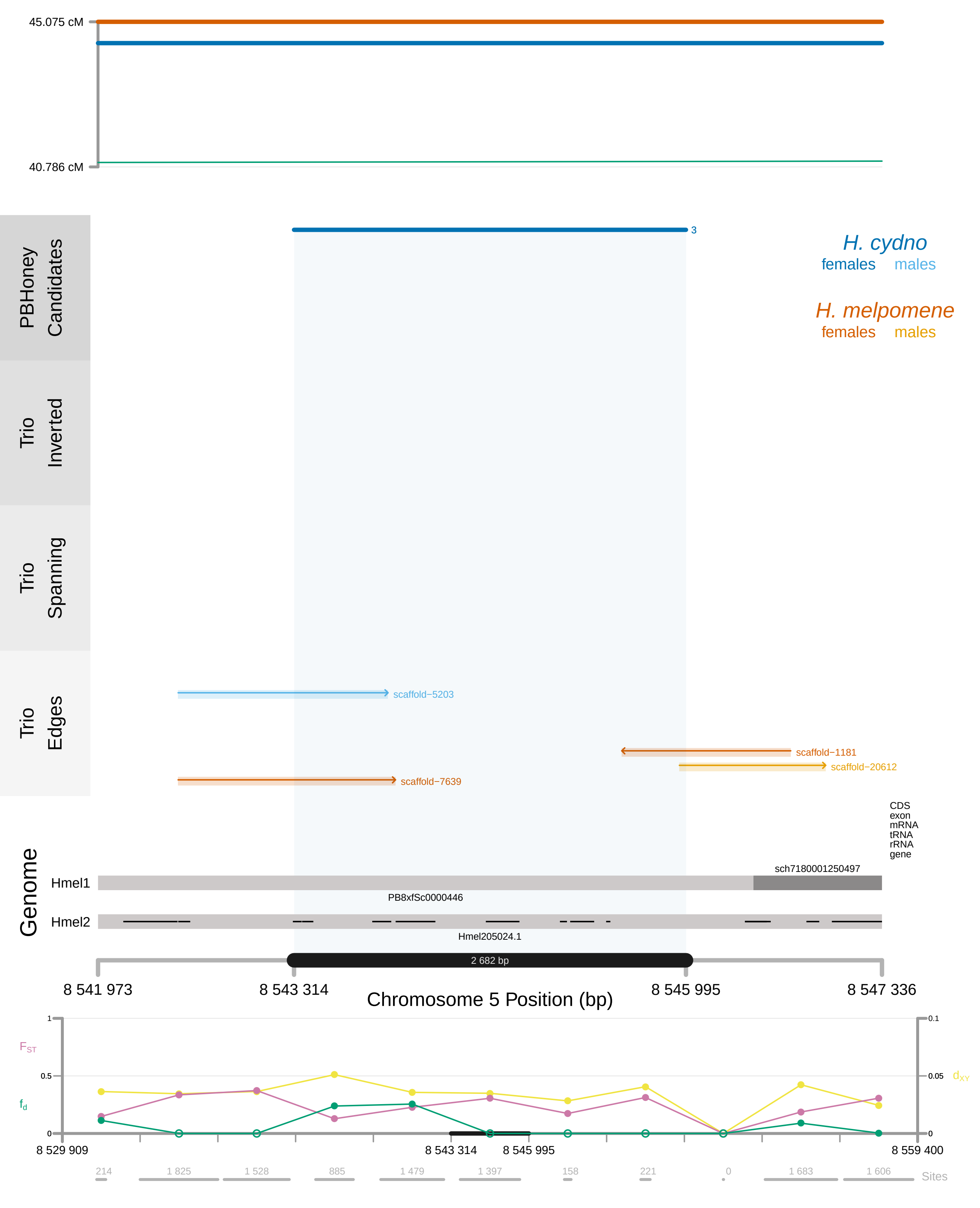

Figure S12.28

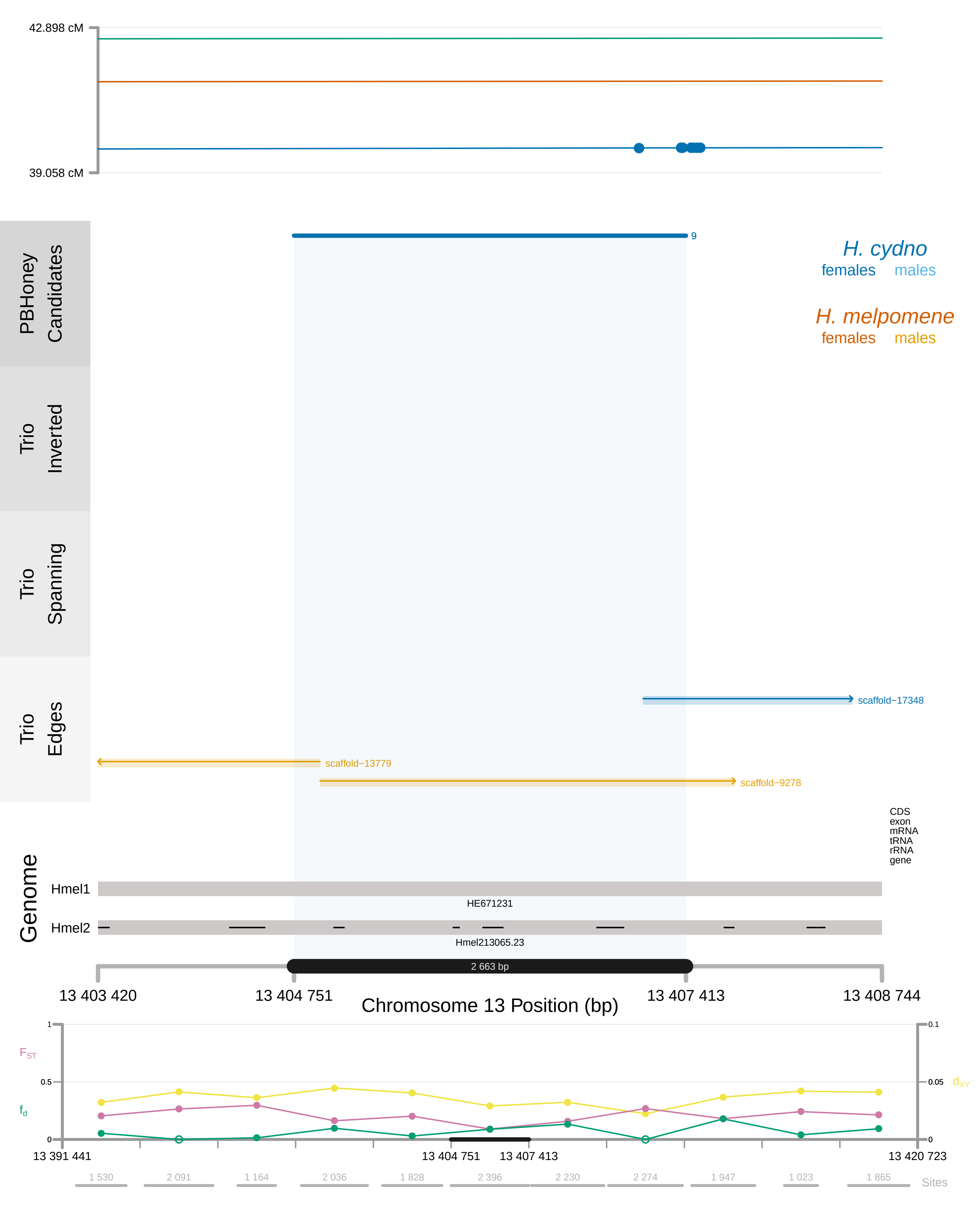

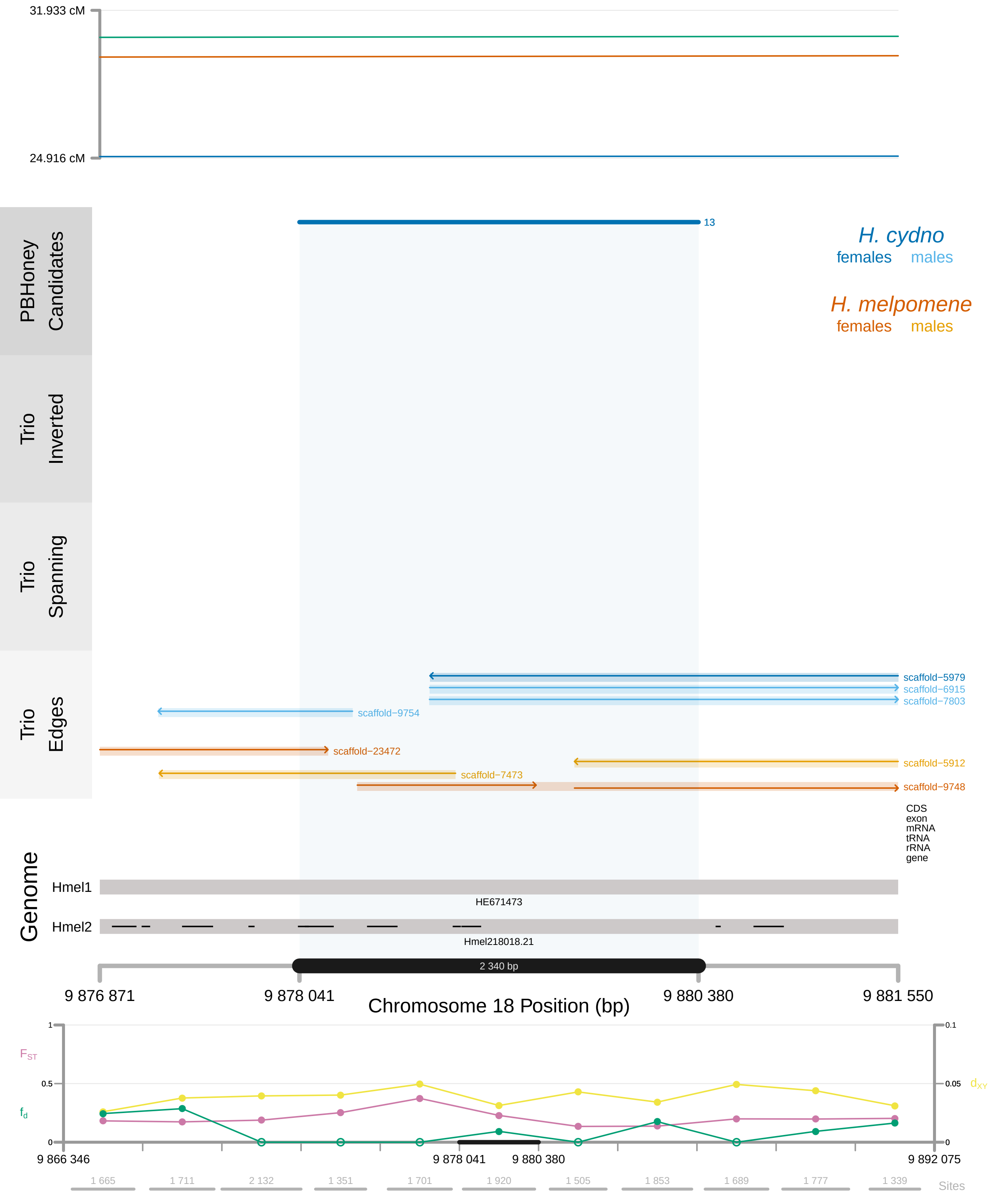
31.933 cM
24.916 cM
PBHoney Candidates
13
H. cydno
females males
H. melpomene
females males
Trio Inverted
Trio Spanning
Trio Edges
scaffold−5979
scaffold−6915
scaffold−7803
scaffold−9754
scaffold−23472
scaffold−5912
scaffold−7473
scaffold−9748
CDS
exon
mRNA
tRNA
rRNA
gene
Genome
Hmel1
HE671473
Hmel2
Hmel218018.21
2 340 bp
9 876 871
9 878 041
9 880 380
9 881 550
Chromosome 18 Position (bp)
F_ST
f_d
d_XY
1
0.5
0
0.1
0.05
9 866 346
9 878 041
9 880 380
9 892 075
1 665
1 711
2 132
1 351
1 701
1 920
1 505
1 853
1 689
1 777
1 339
Sites

Figure S12.30 *H. cydno* Split reads only

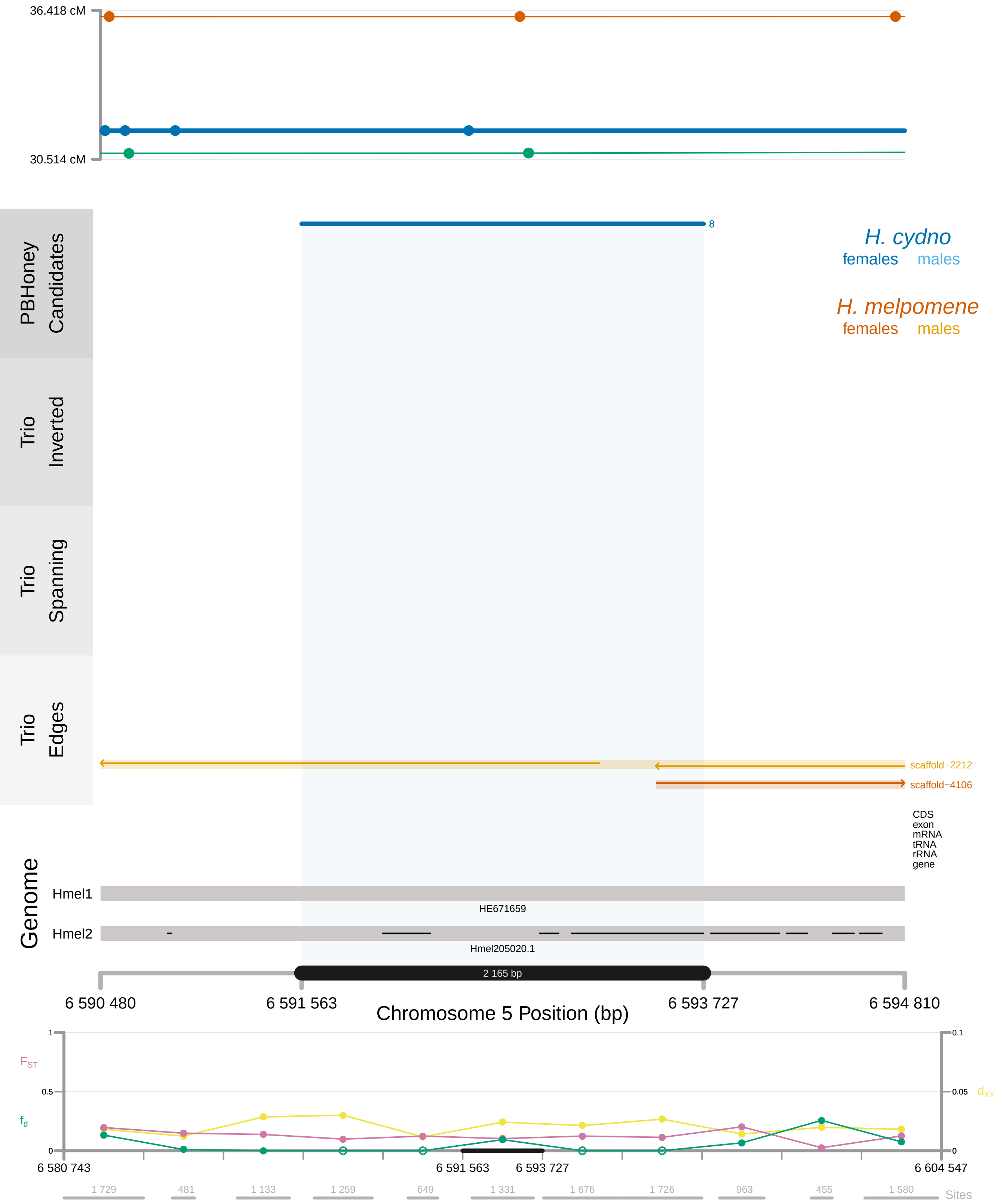

Figure S12.31 *H. cydno* Split reads only

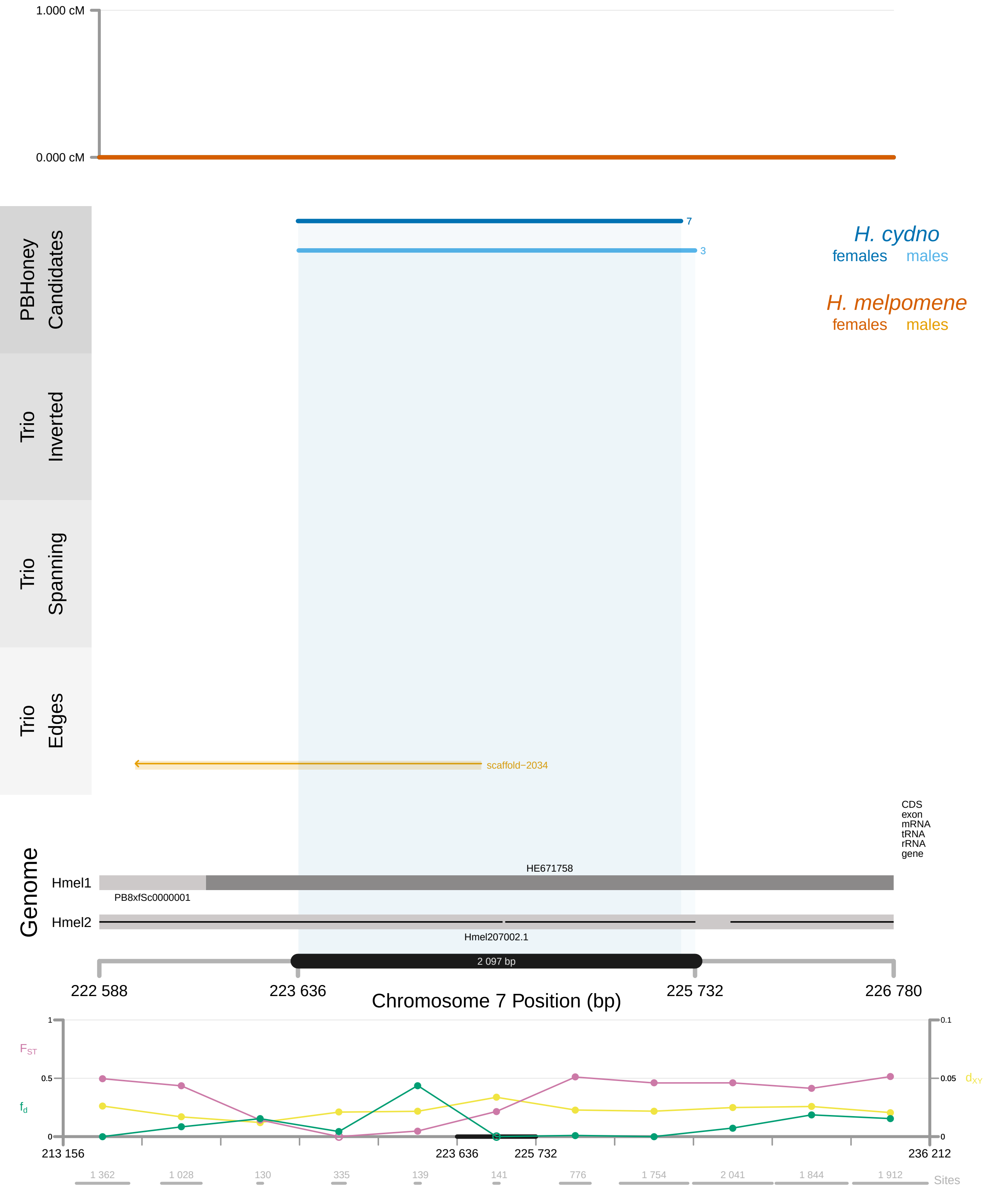

Figure S12.32 *H. cydno* Split reads only

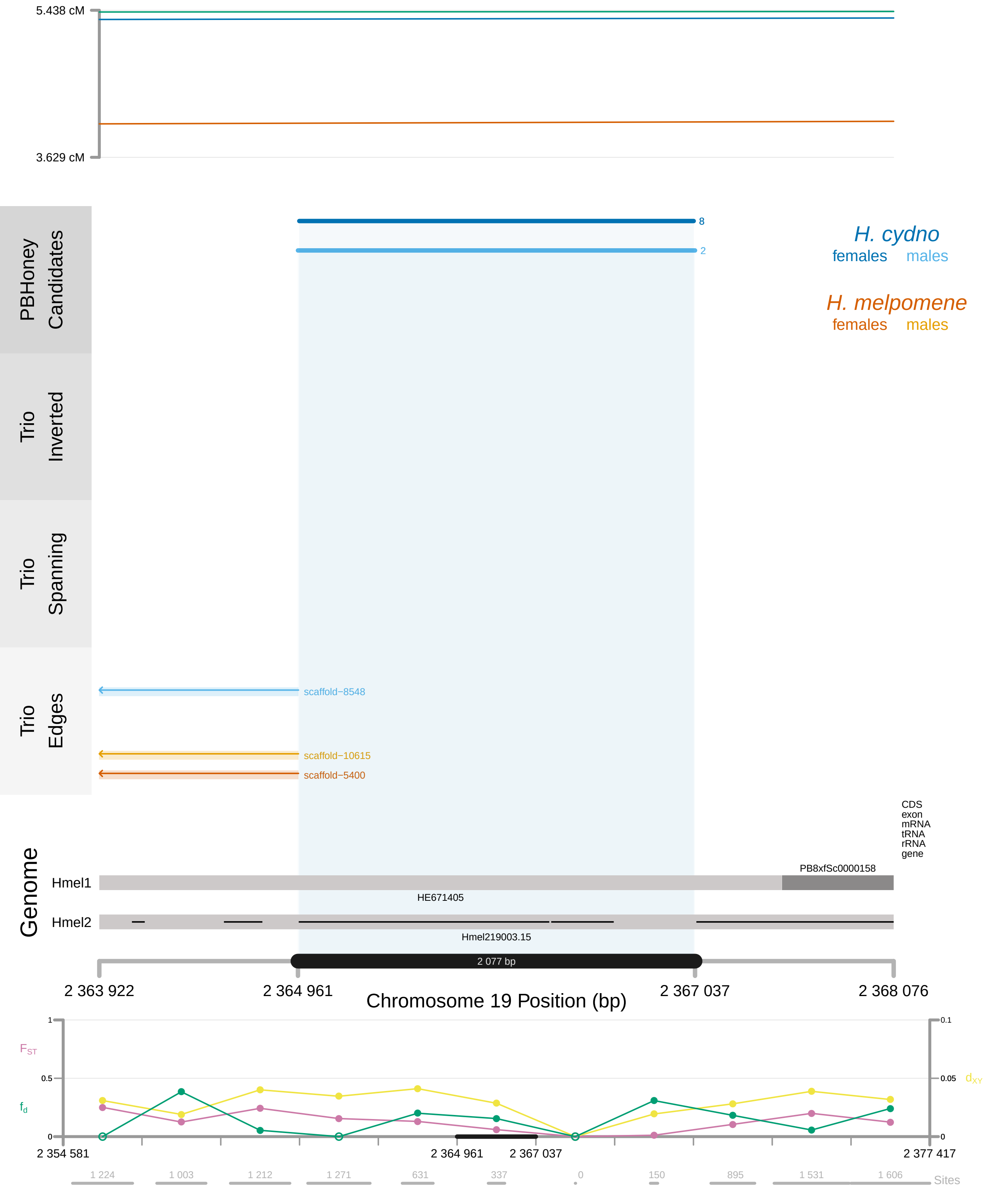

Figure S12.33 *H. cydno* Split reads only

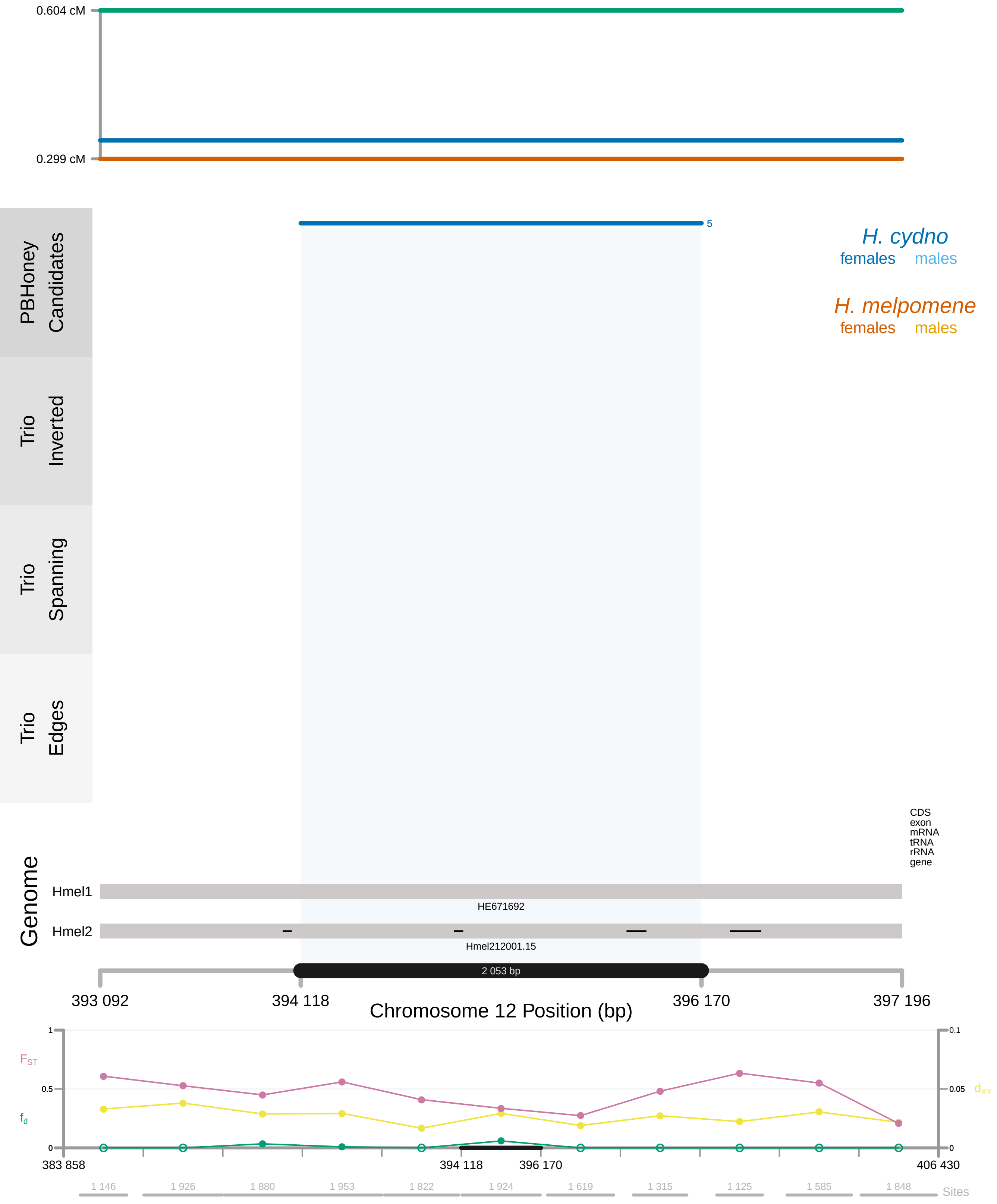

Figure S12.34 *H. cydno* Split reads only

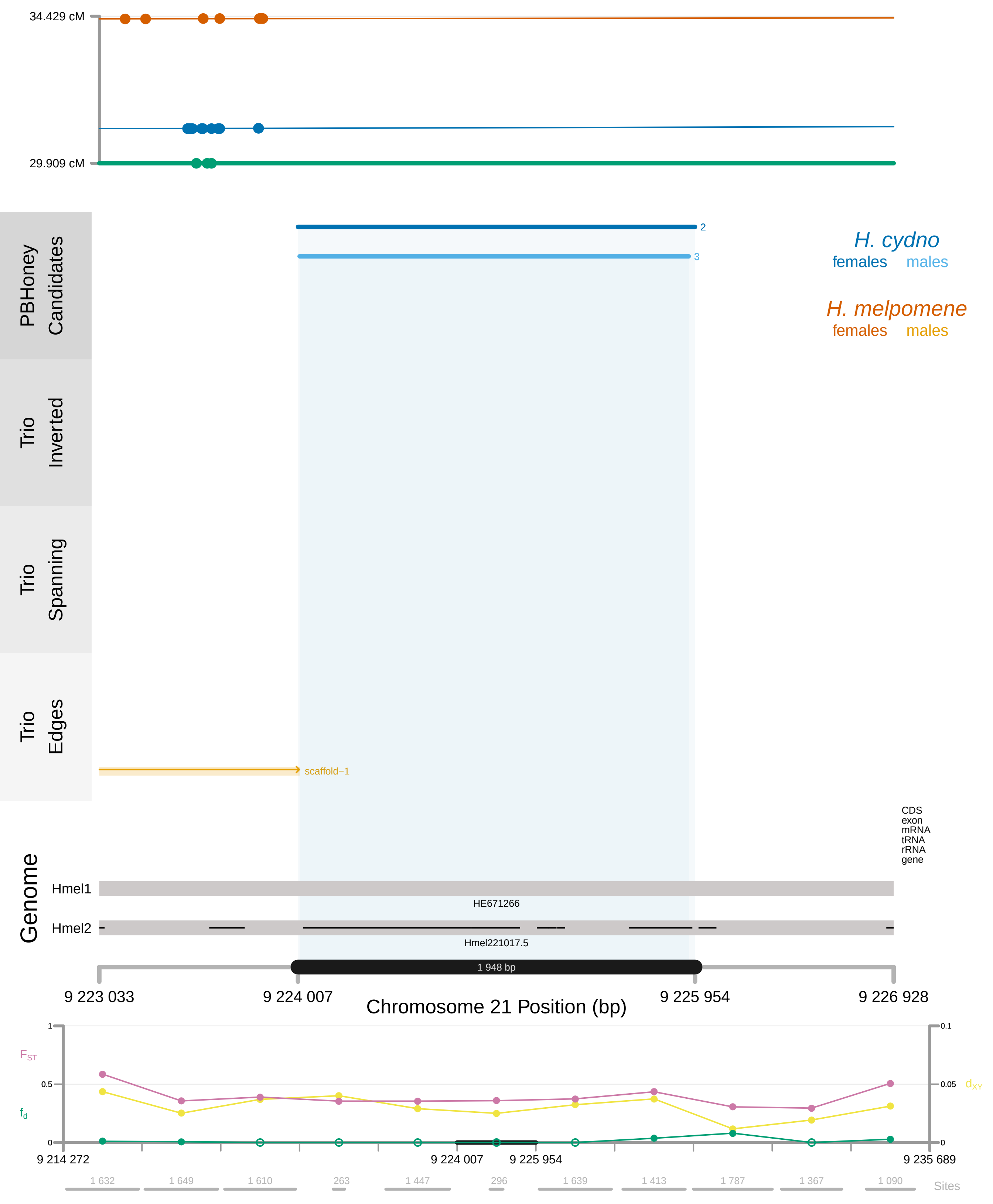

Figure S12.35

H. cydno

Split reads only

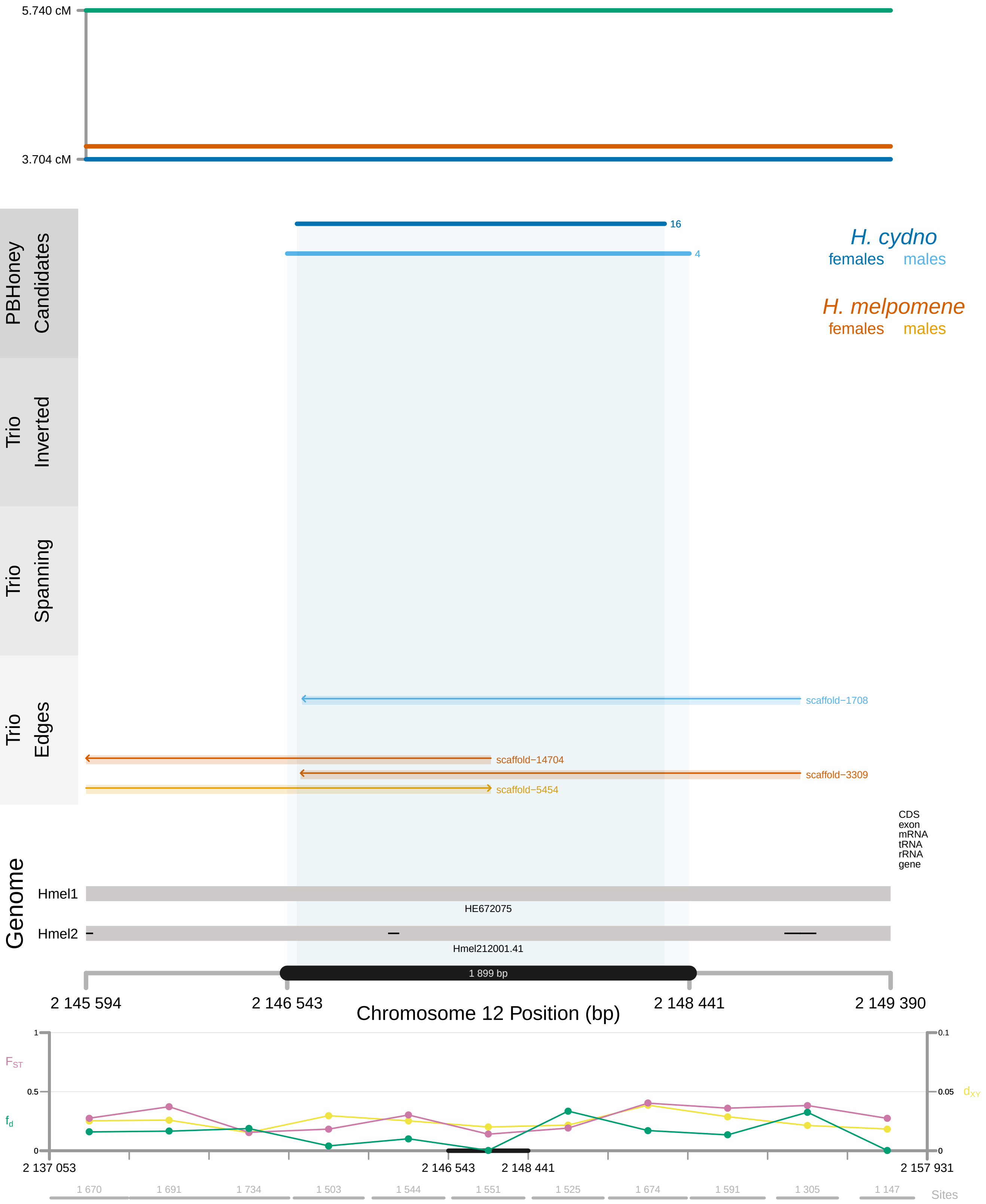

Figure S12.36

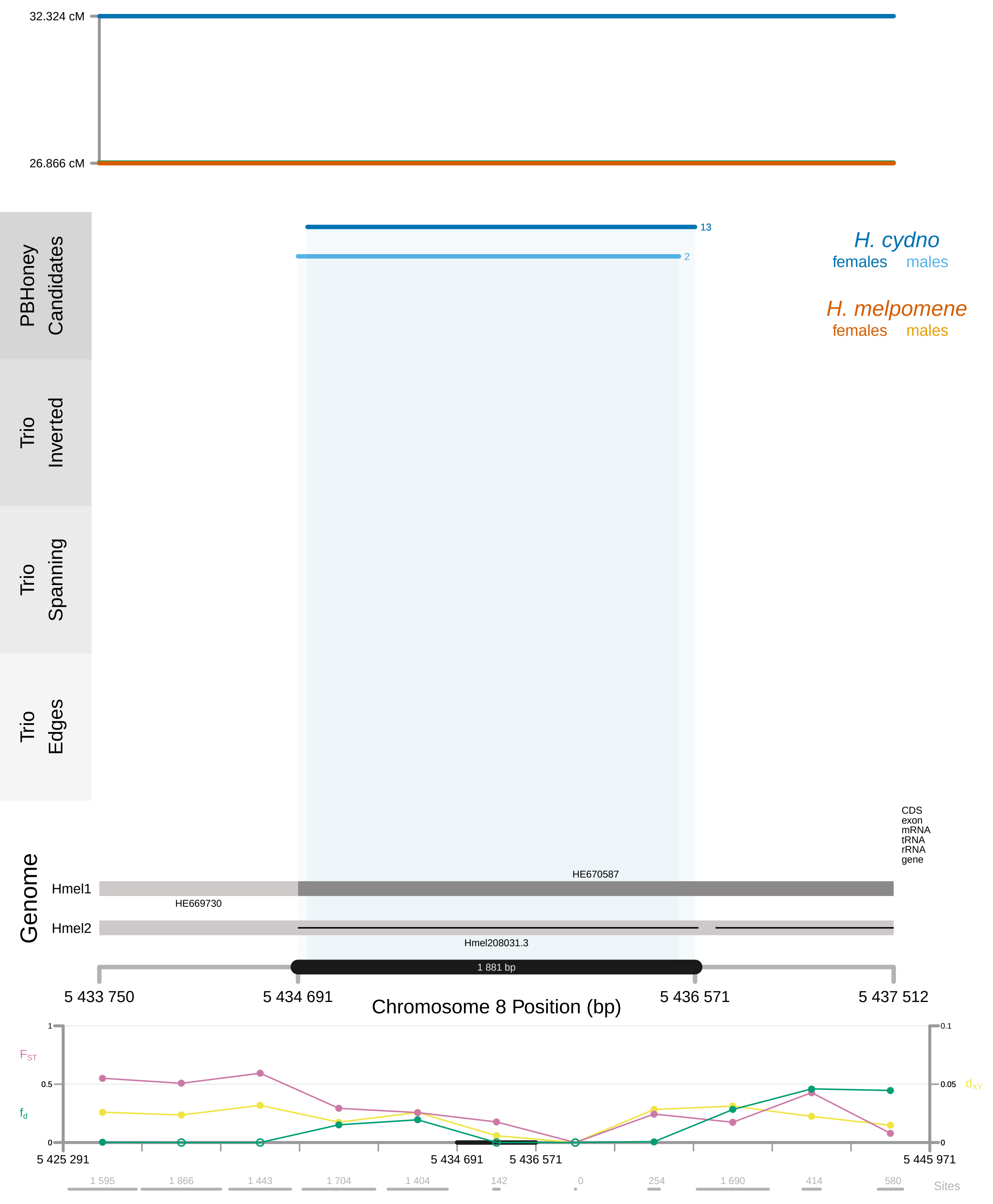

Figure S12.37 *H. cydno* Split reads only

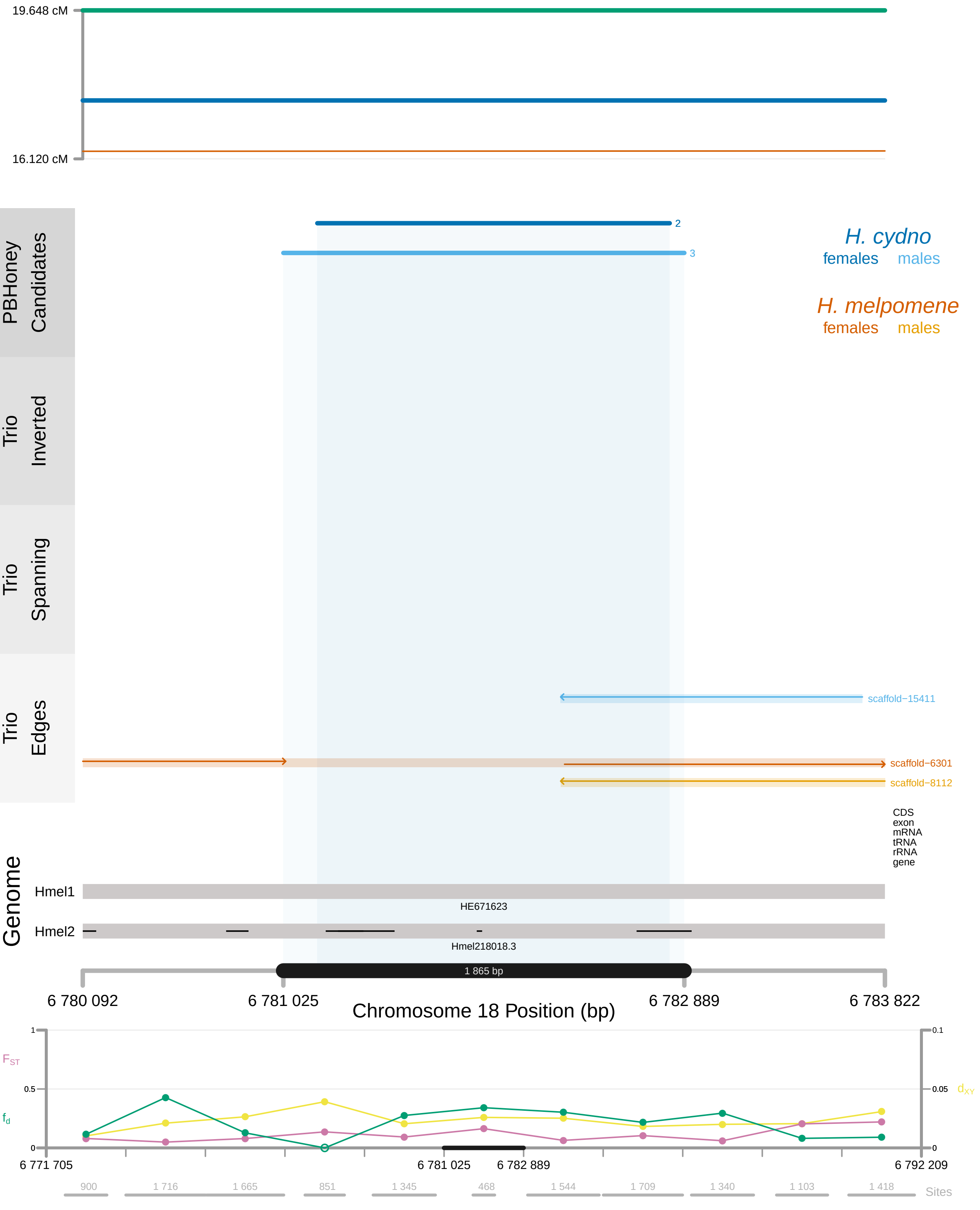

Figure S12.38 *H. cydno* Split reads only

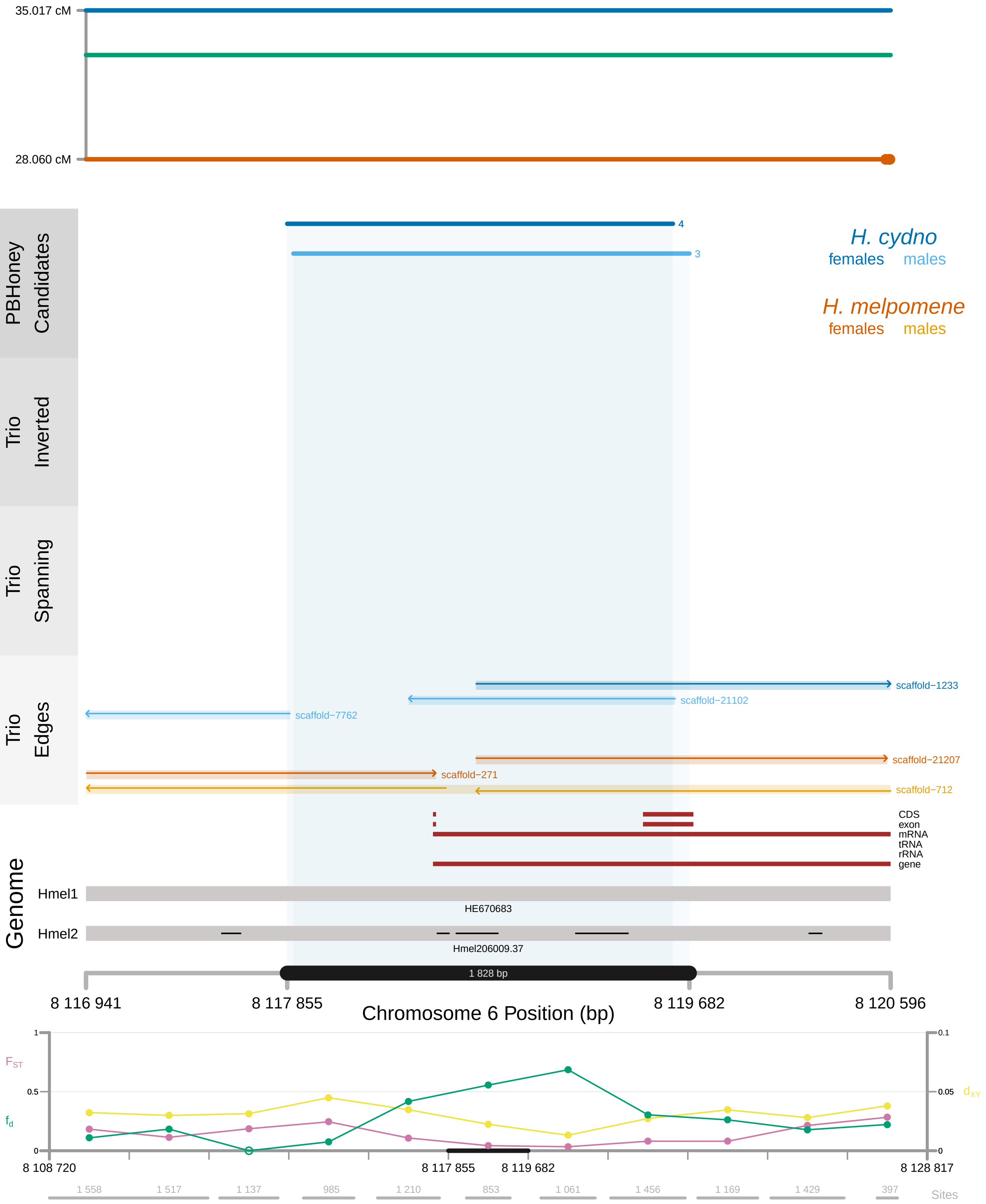

Figure S12.39 *H. cydno* Split reads only

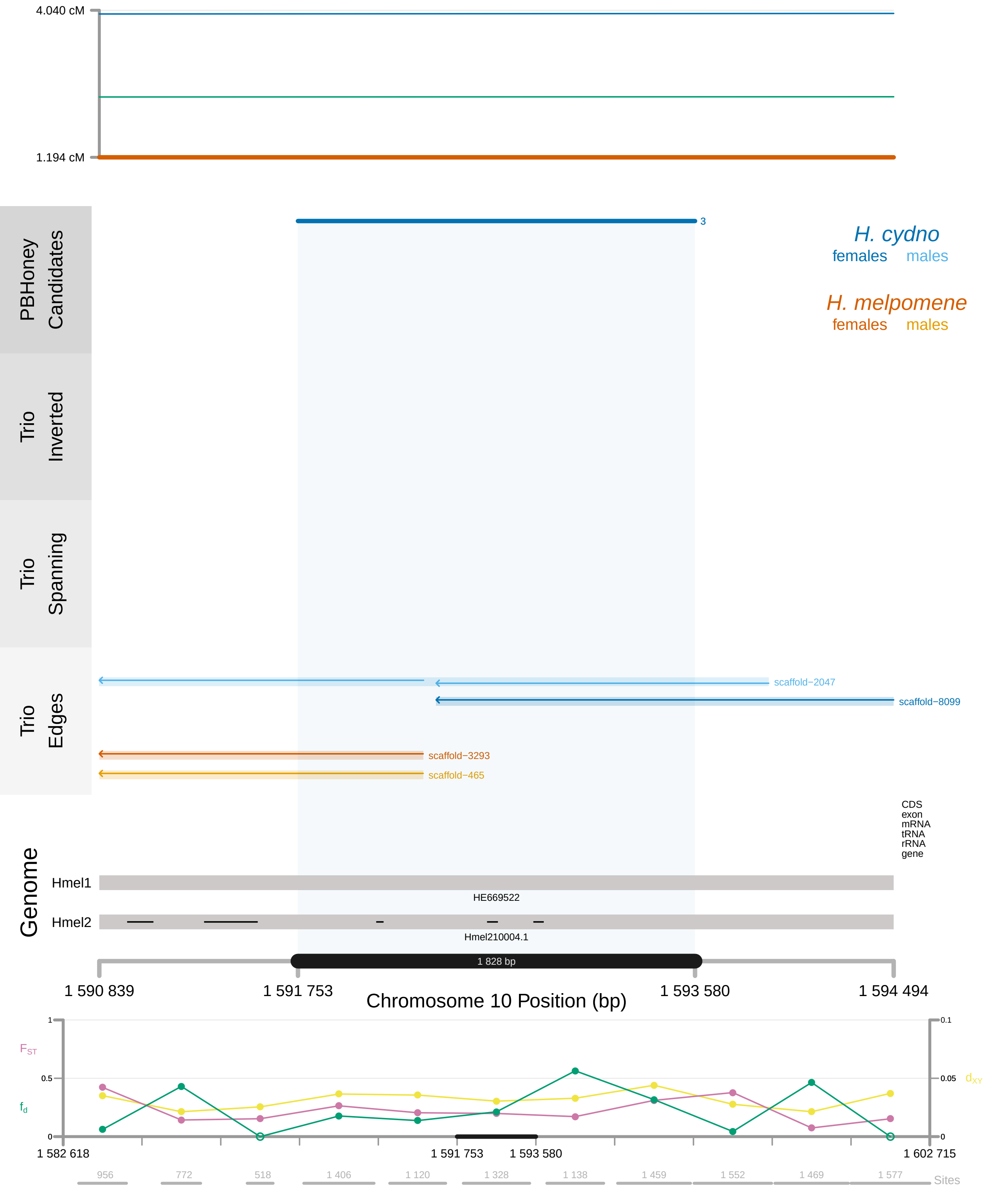

Figure S12.40 *H. cydno* Split reads only

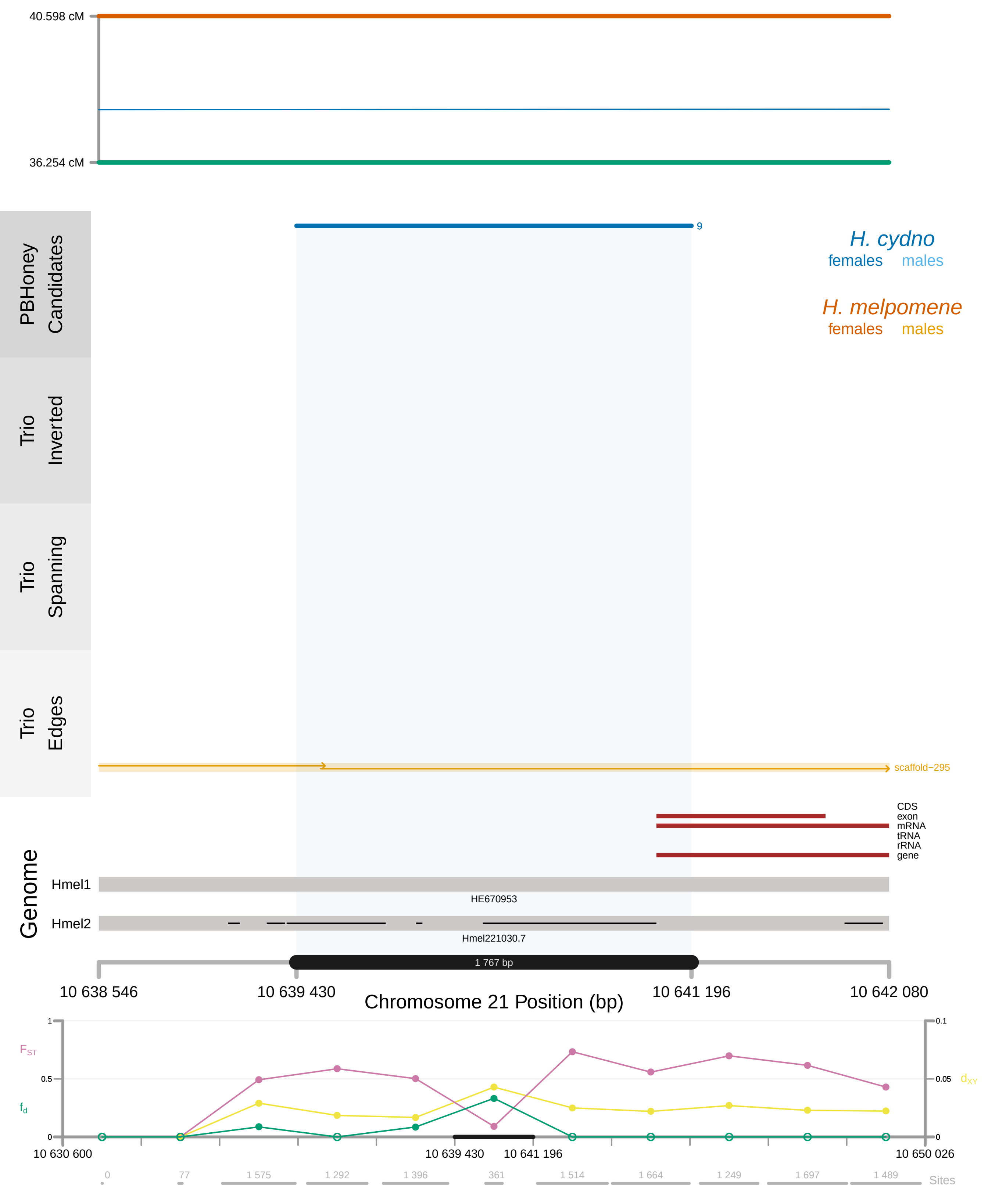

Figure S12.41 *H. cydno* Split reads only

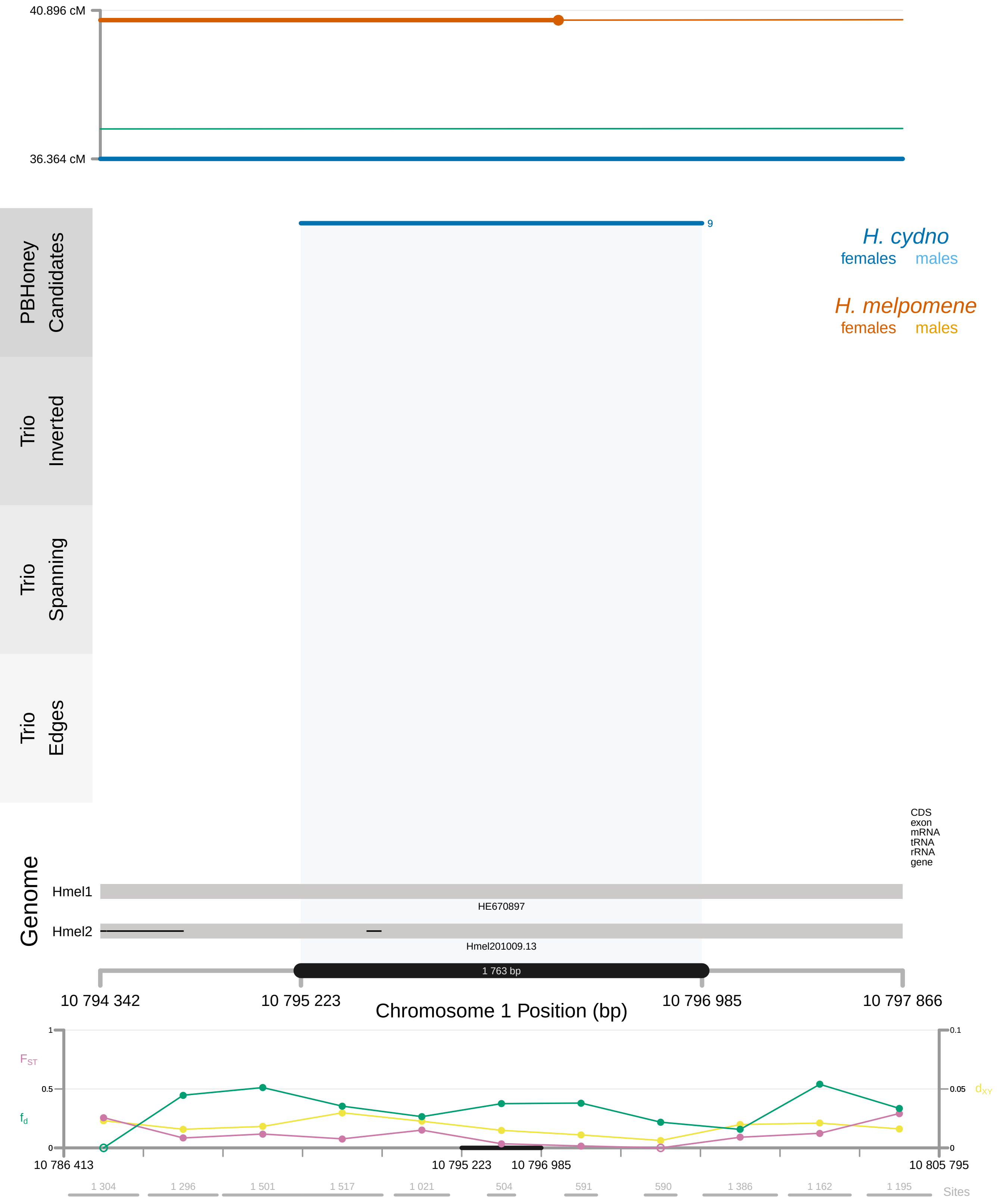

Figure S12.42 *H. cydno* Split reads only

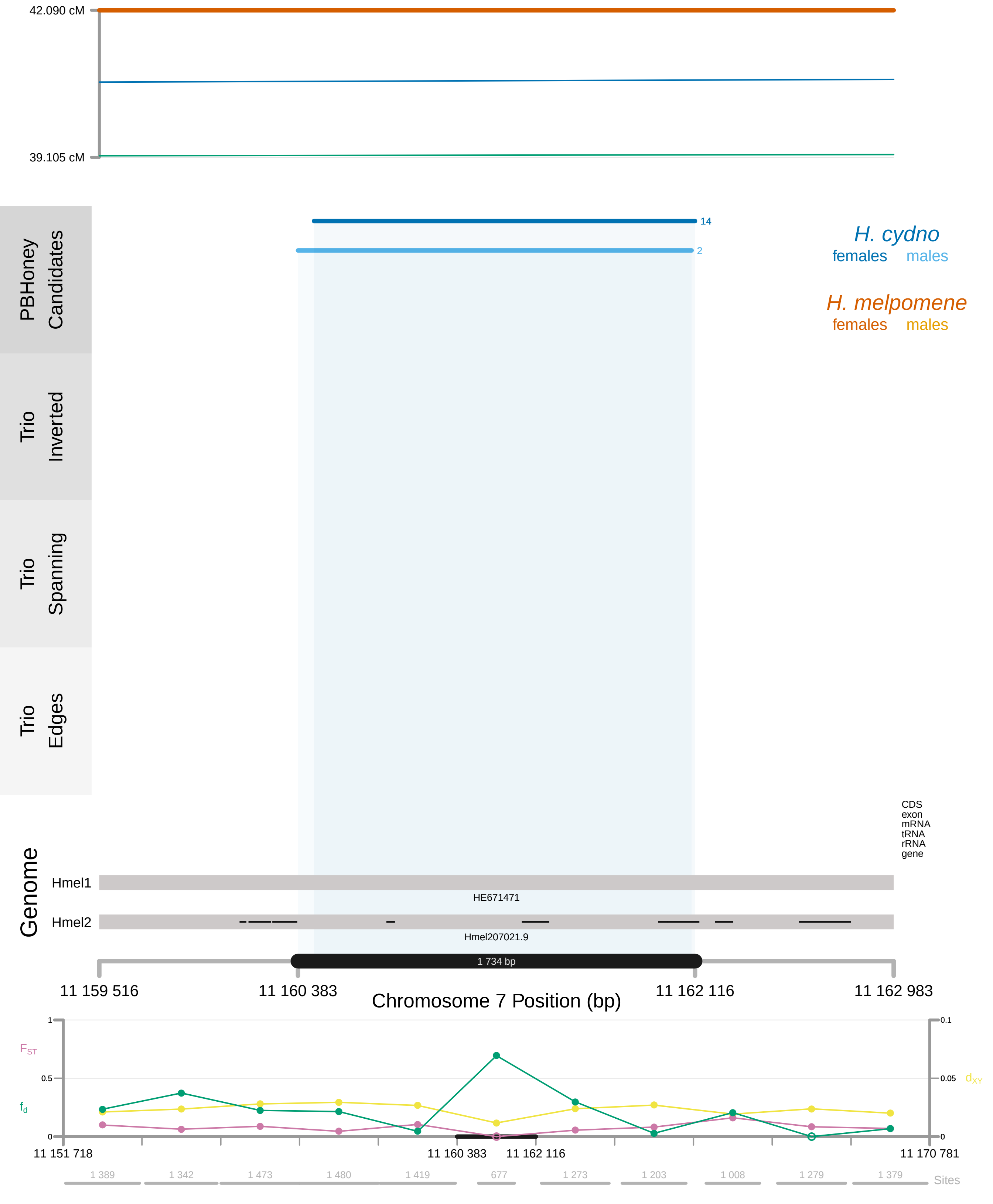

Figure S12.43 *H. cydno* Split reads only

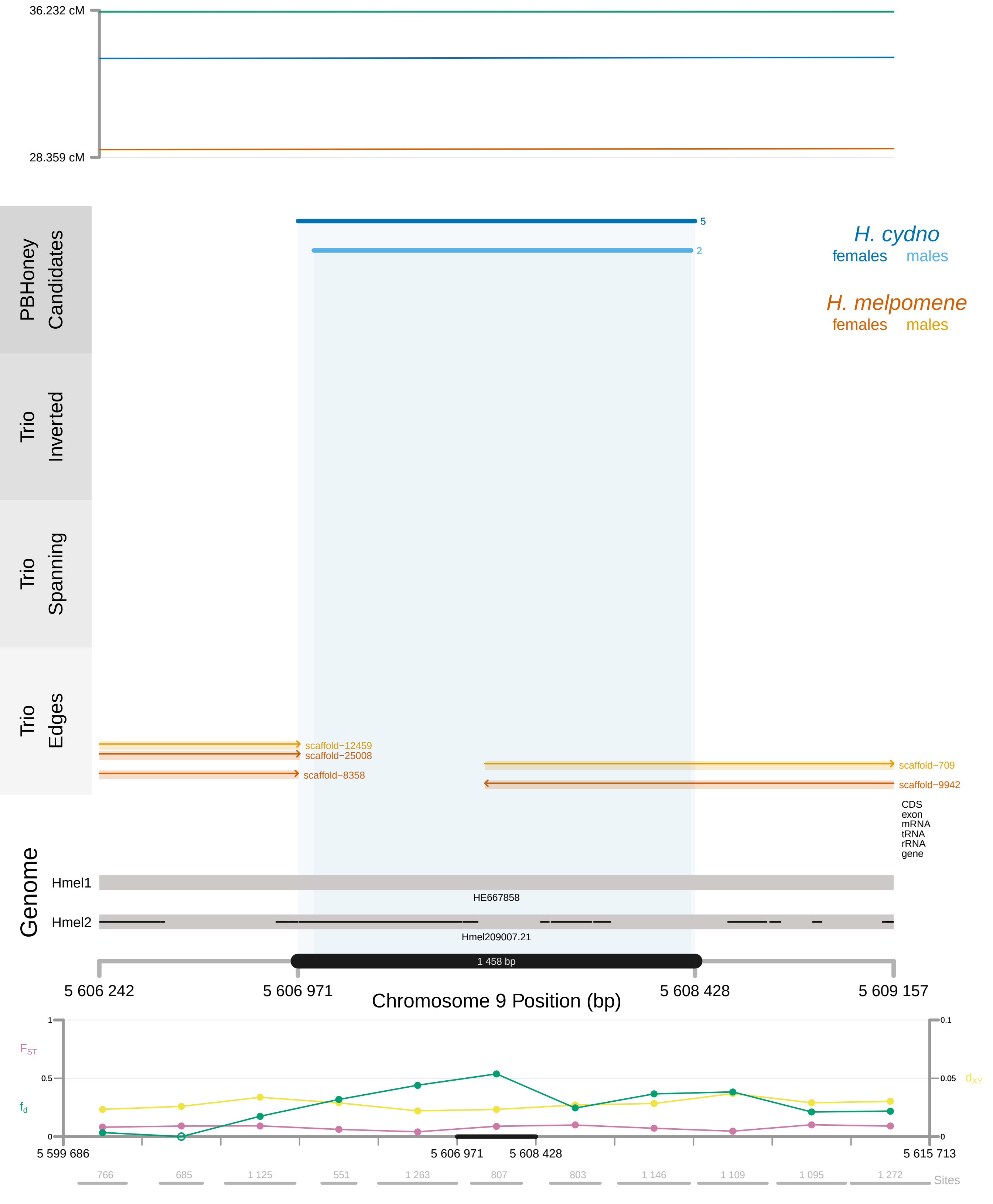

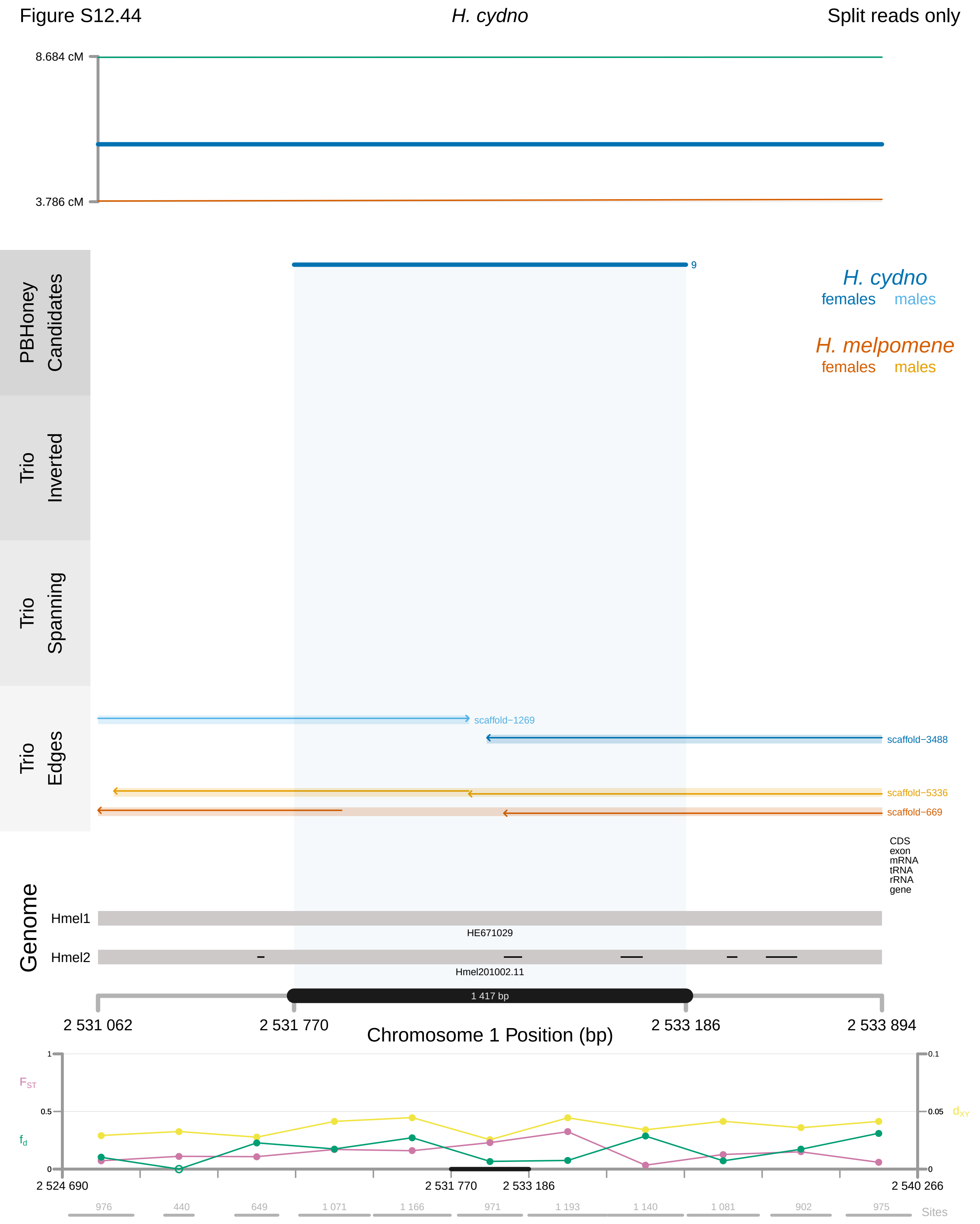

Figure S12.44
H. cydno
Split reads only
8.684 cM
3.786 cM
PBHoney Candidates
9
H. cydno
females males
H. melpomene
females males
Trio Inverted
Trio Spanning
Trio Edges
scaffold−1269
scaffold−3488
scaffold−5336
scaffold−669
CDS
exon
mRNA
tRNA
rRNA
gene
Genome
Hmel1
HE671029
Hmel2
Hmel201002.11
1 417 bp
2 531 062
2 531 770
2 533 186
2 533 894
Chromosome 1 Position (bp)
F_ST
f_d
d_XY
1
0.5
0
0.1
0.05
0
2 524 690
2 531 770
2 533 186
2 540 266
976
440
649
1 071
1 166
971
1 193
1 140
1 081
902
975
Sites

Figure S12.45 *H. cydno* Split reads only

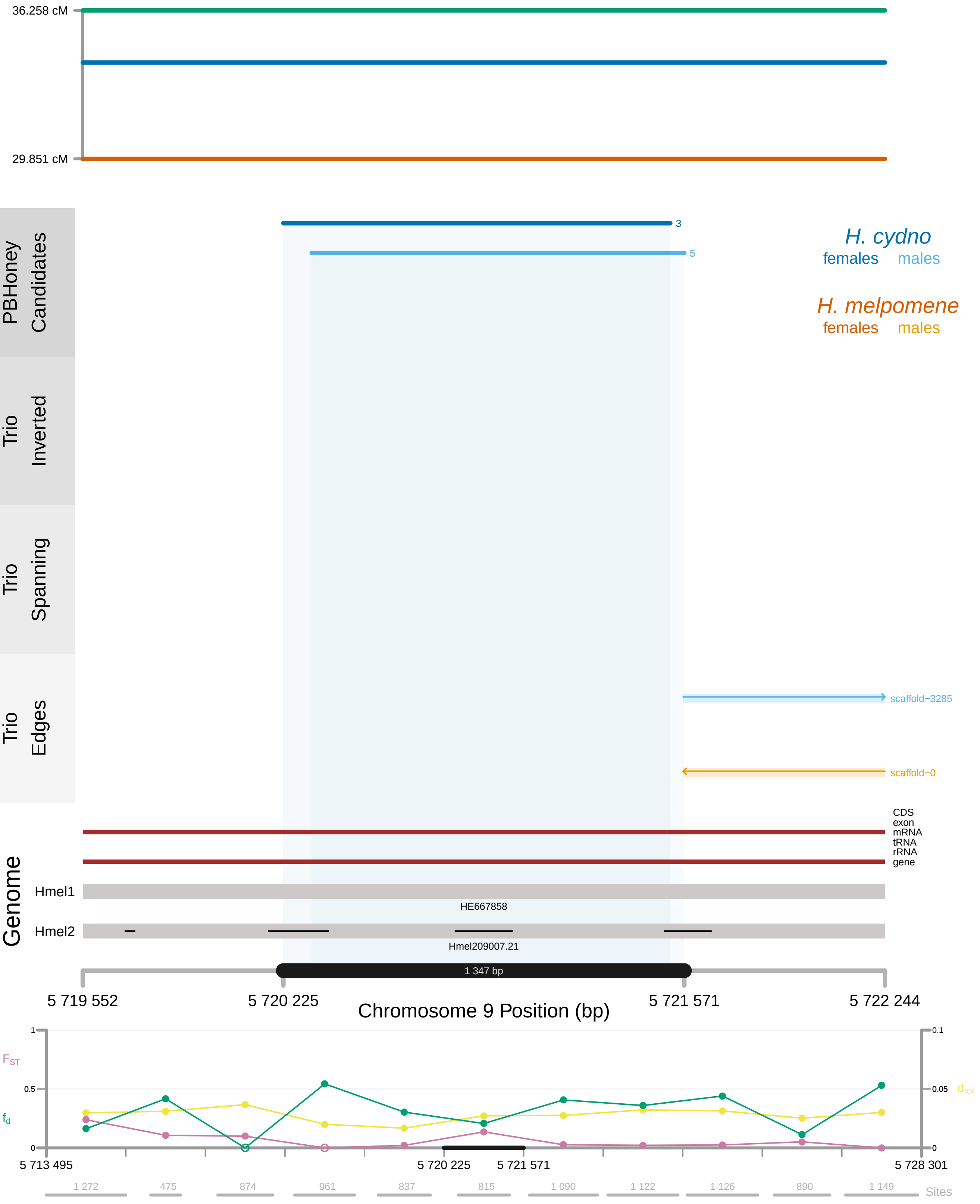

Figure S12.46

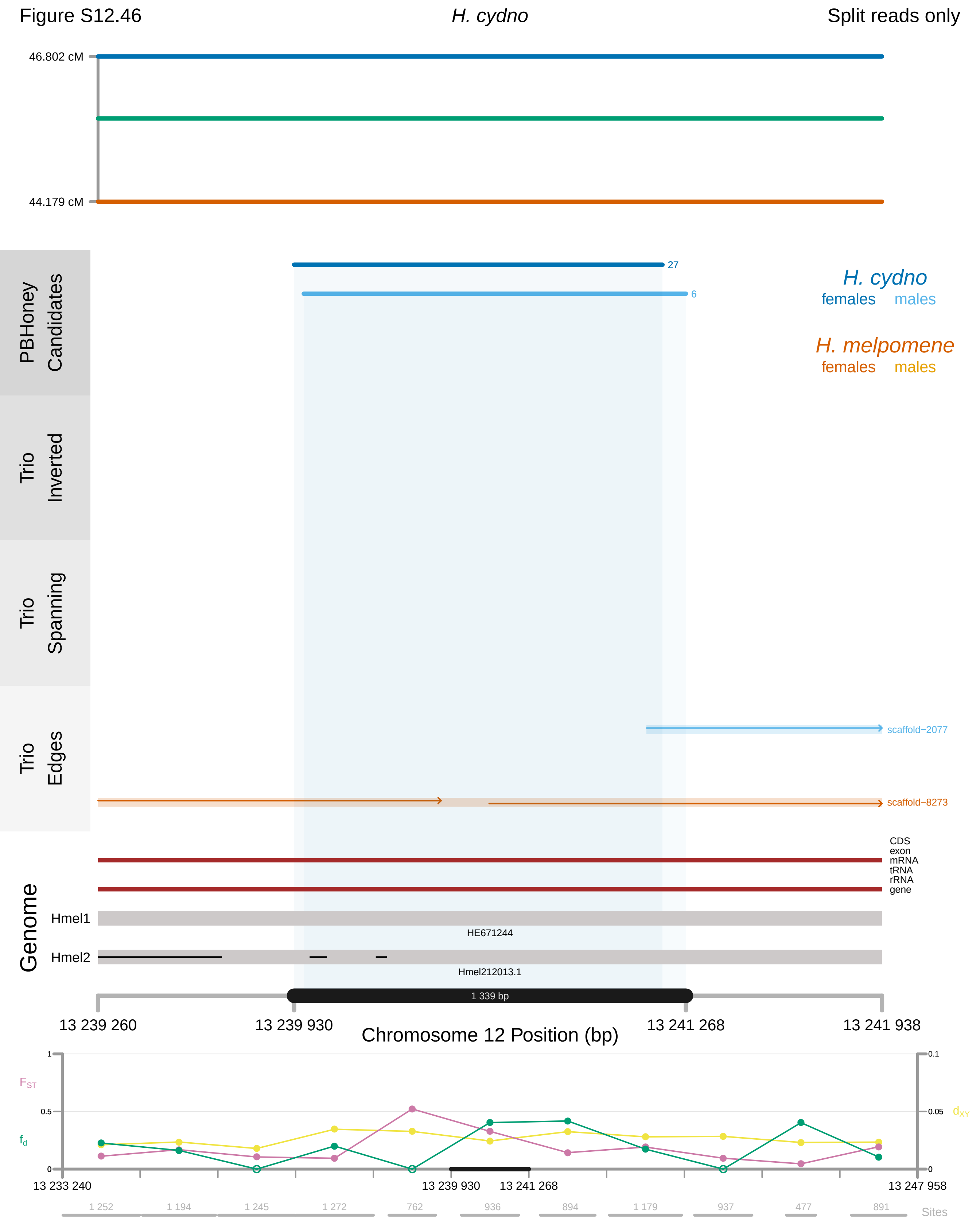

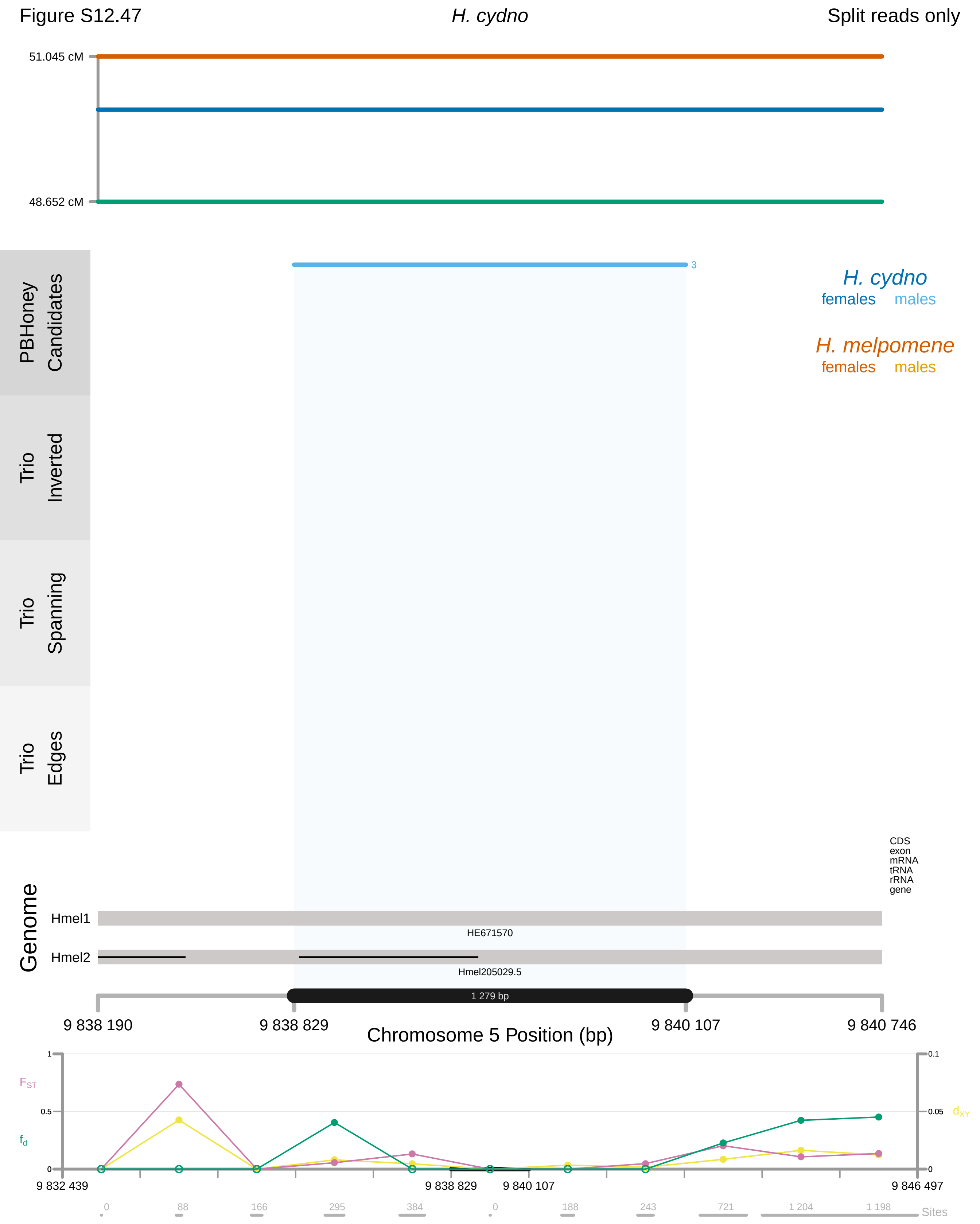

Figure S12.47
H. cydno
Split reads only
51.045 cM
48.652 cM
3
H. cydno
females
males
H. melpomene
females
males
PBHoney Candidates
Trio Inverted
Trio Spanning
Trio Edges
Genome
CDS
exon
mRNA
tRNA
rRNA
gene
Hmel1
HE671570
Hmel2
Hmel205029.5
1 279 bp
9 838 190
9 838 829
9 840 107
9 840 746
Chromosome 5 Position (bp)
1
0.5
0
FST
fd
0.1
0.05
dXY
9 832 439
9 838 829
9 840 107
9 846 497
0
88
166
295
384
0
188
243
721
1 204
1 198
Sites

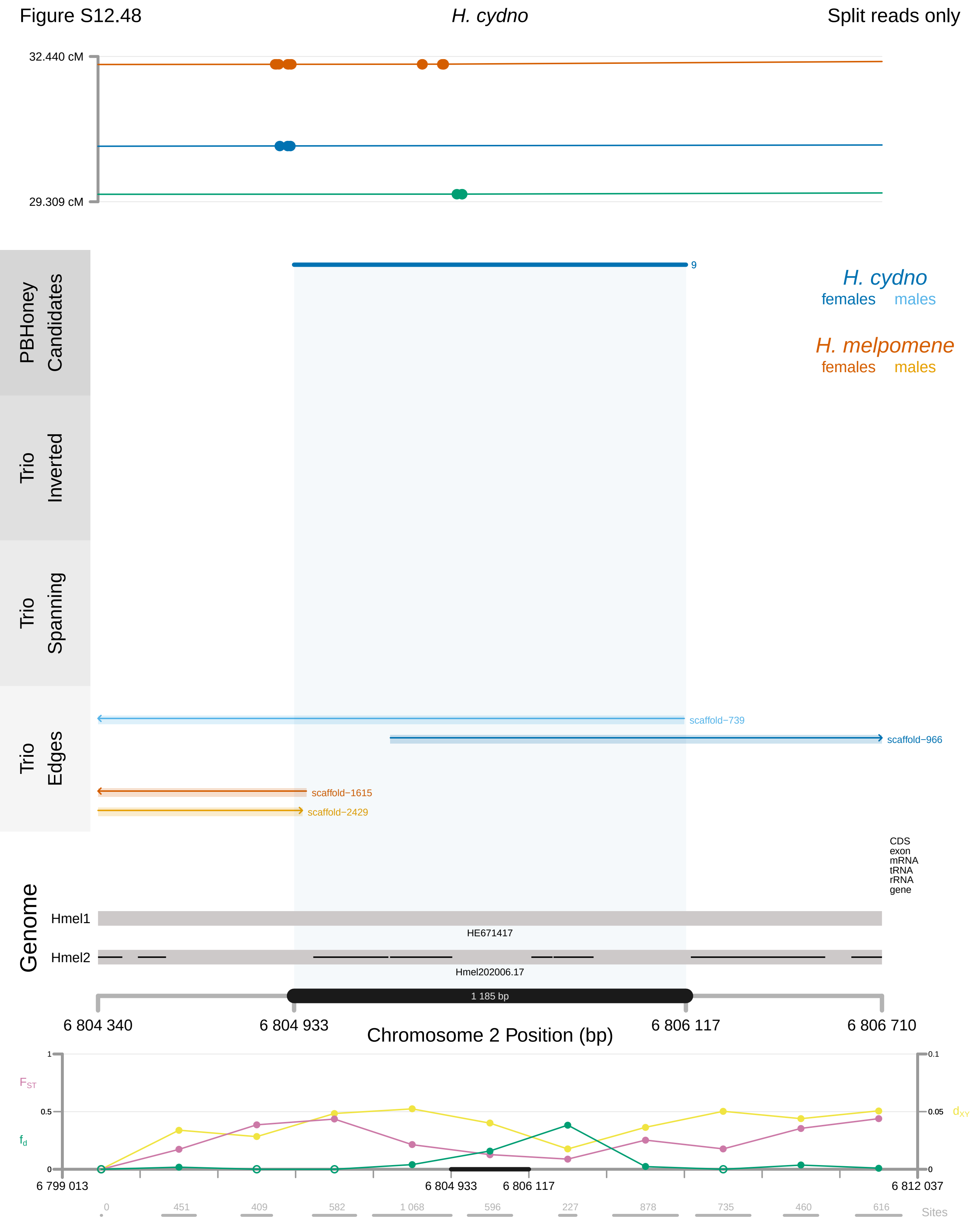

Figure S12.48
H. cydno
Split reads only
32.440 cM
29.309 cM
PBHoney Candidates
Trio Inverted
Trio Spanning
Trio Edges
Genome
9
H. cydno
females males
H. melpomene
females males
scaffold−739
scaffold−966
scaffold−1615
scaffold−2429
CDS
exon
mRNA
tRNA
rRNA
gene
Hmel1
HE671417
Hmel2
Hmel202006.17
1 185 bp
6 804 340
6 804 933
6 806 117
6 806 710
Chromosome 2 Position (bp)
F_ST
f_d
d_XY
1
0.5
0
0.1
0.05
0
6 799 013
6 804 933
6 806 117
6 812 037
0
451
409
582
1 068
596
227
878
735
460
616
Sites

Figure S12.49

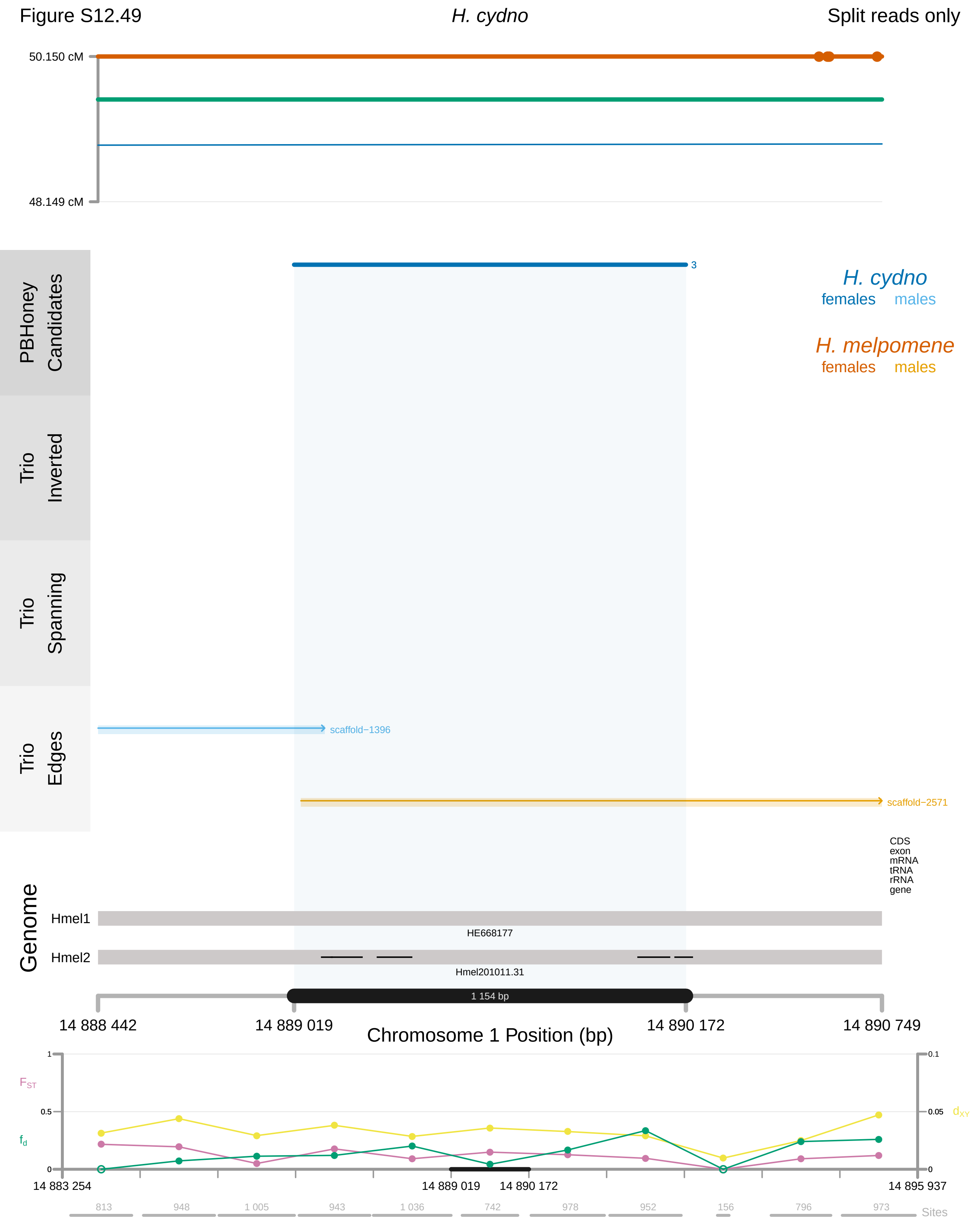

Figure S12.50 *H. cydno* Split reads only

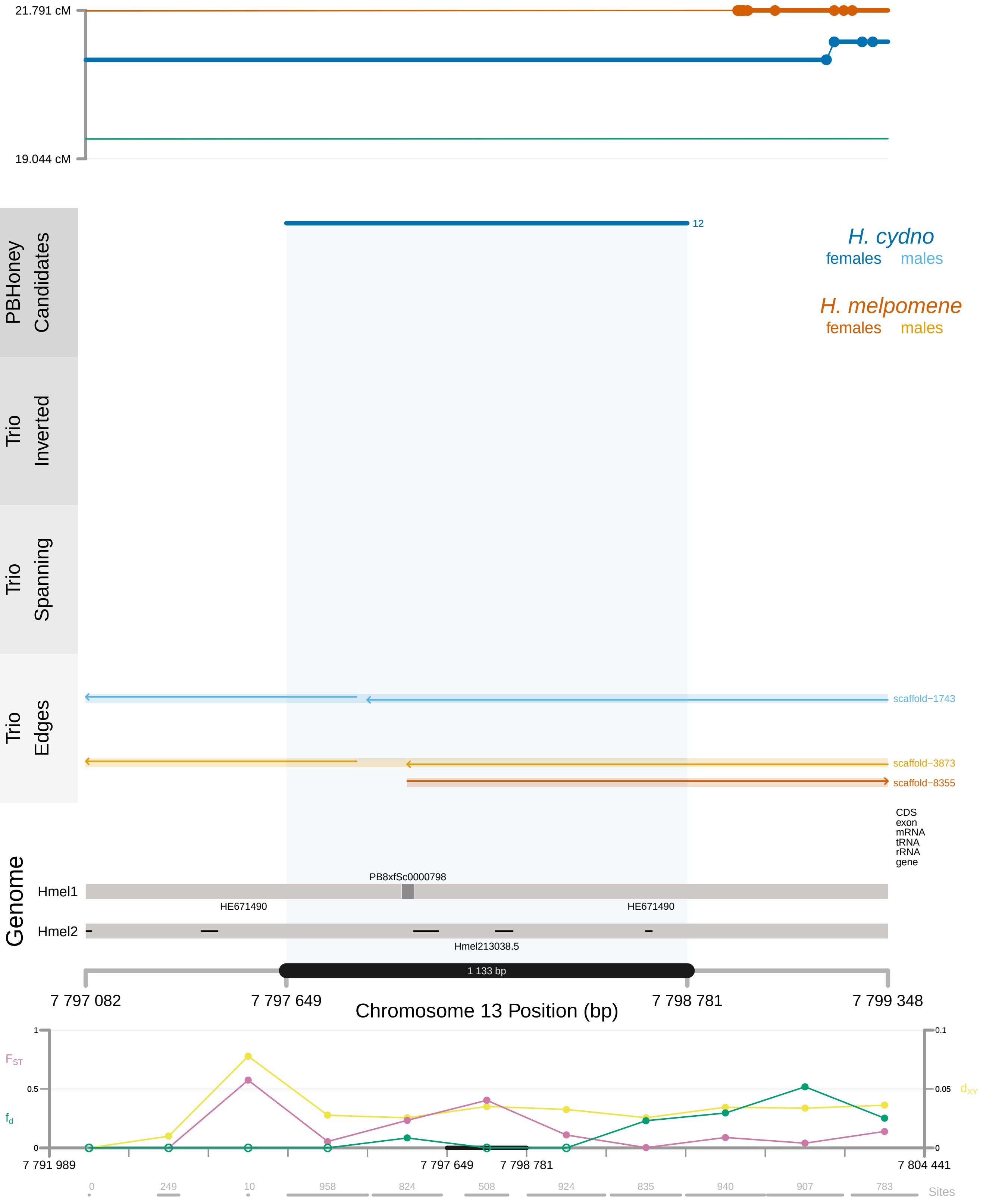

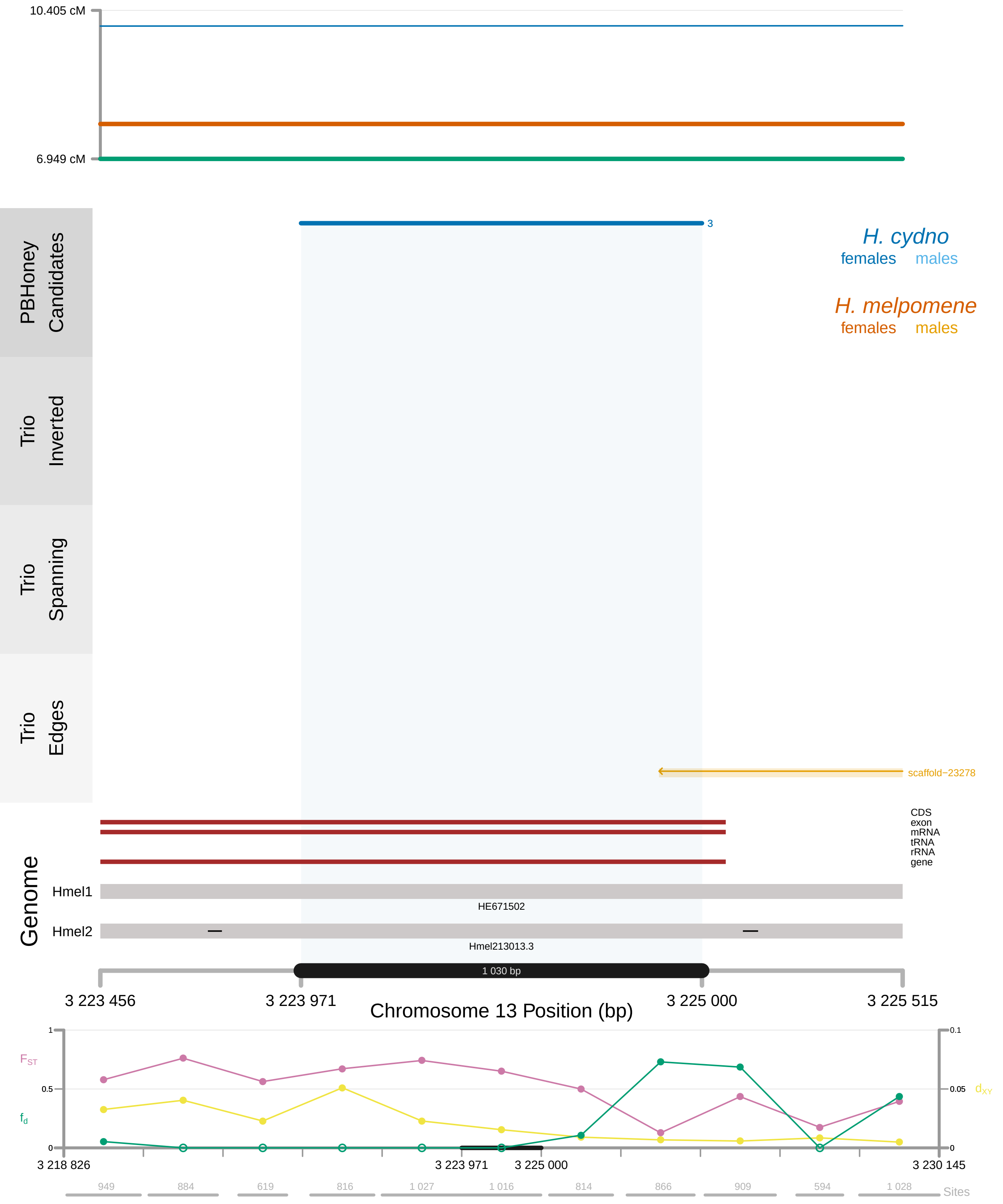
10.405 cM
6.949 cM
PBHoney Candidates
Trio Inverted
Trio Spanning
Trio Edges
Genome
3
H. cydno
females males
H. melpomene
females males
scaffold−23278
CDS
exon
mRNA
tRNA
rRNA
gene
Hmel1
HE671502
Hmel2
Hmel213013.3
1 030 bp
3 223 456
3 223 971
3 225 000
3 225 515
Chromosome 13 Position (bp)
FST
fd
dXY
1
0.5
0
0.1
0.05
0
3 218 826
3 223 971
3 225 000
3 230 145
949
884
619
816
1 027
1 016
814
866
909
594
1 028
Sites

Figure S12.52 *H. cydno* Split reads only

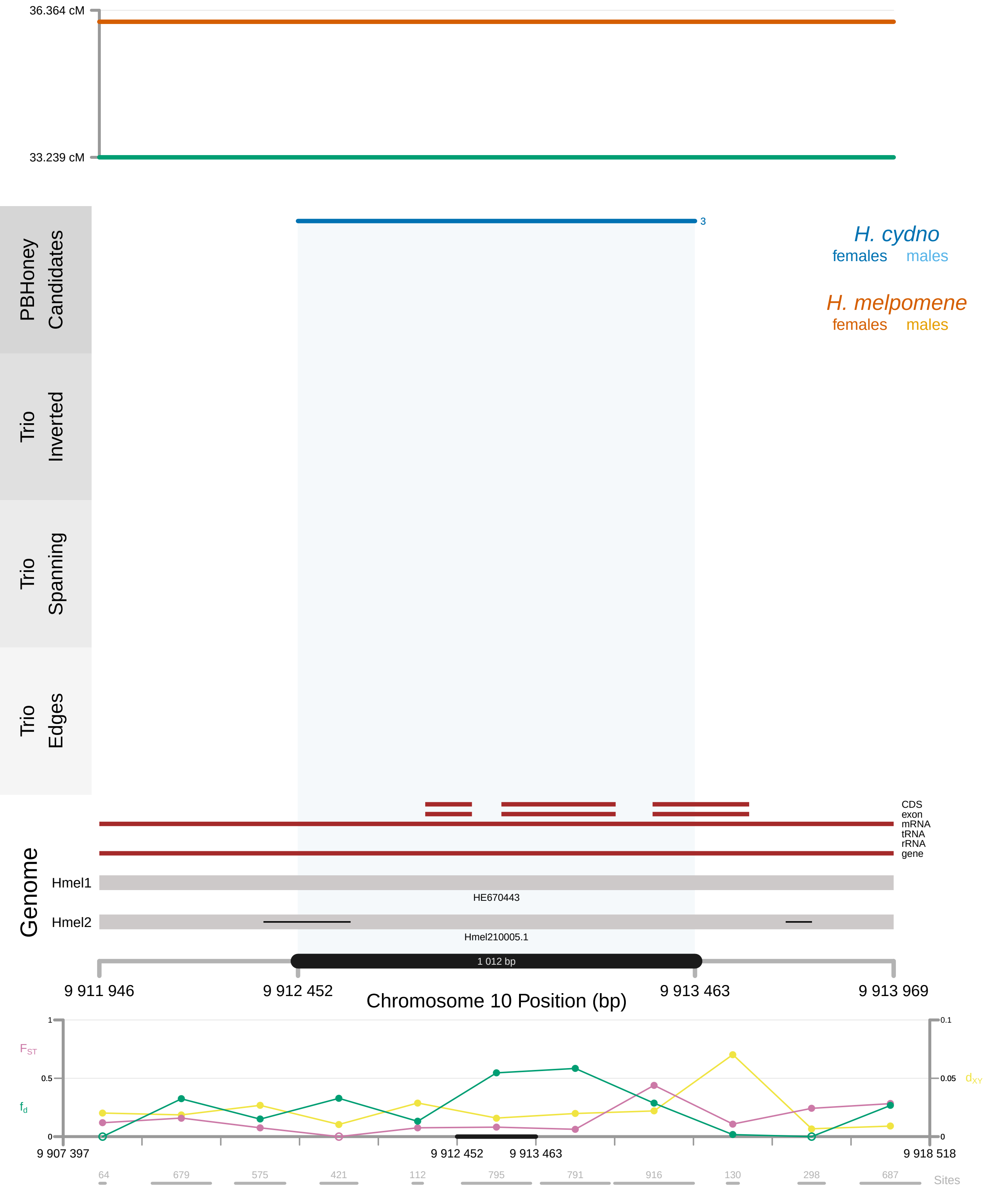

Supplement: Supplementary file 13 — S12, H. cydno, split reads only. [file EVL3-1-138-s013.pdf]
