# Supplementary material for: No evidence for maintenance of a sympatric Heliconius species barrier by chromosomal inversions
Source: Evol Lett. 2017 Jun 14;1(3):138–54. doi: 10.1002/evl3.12 (PMC6122123; doi:10.1002/evl3.12)

Figure S13.1 *H. melpomene* Split reads and trio assembly

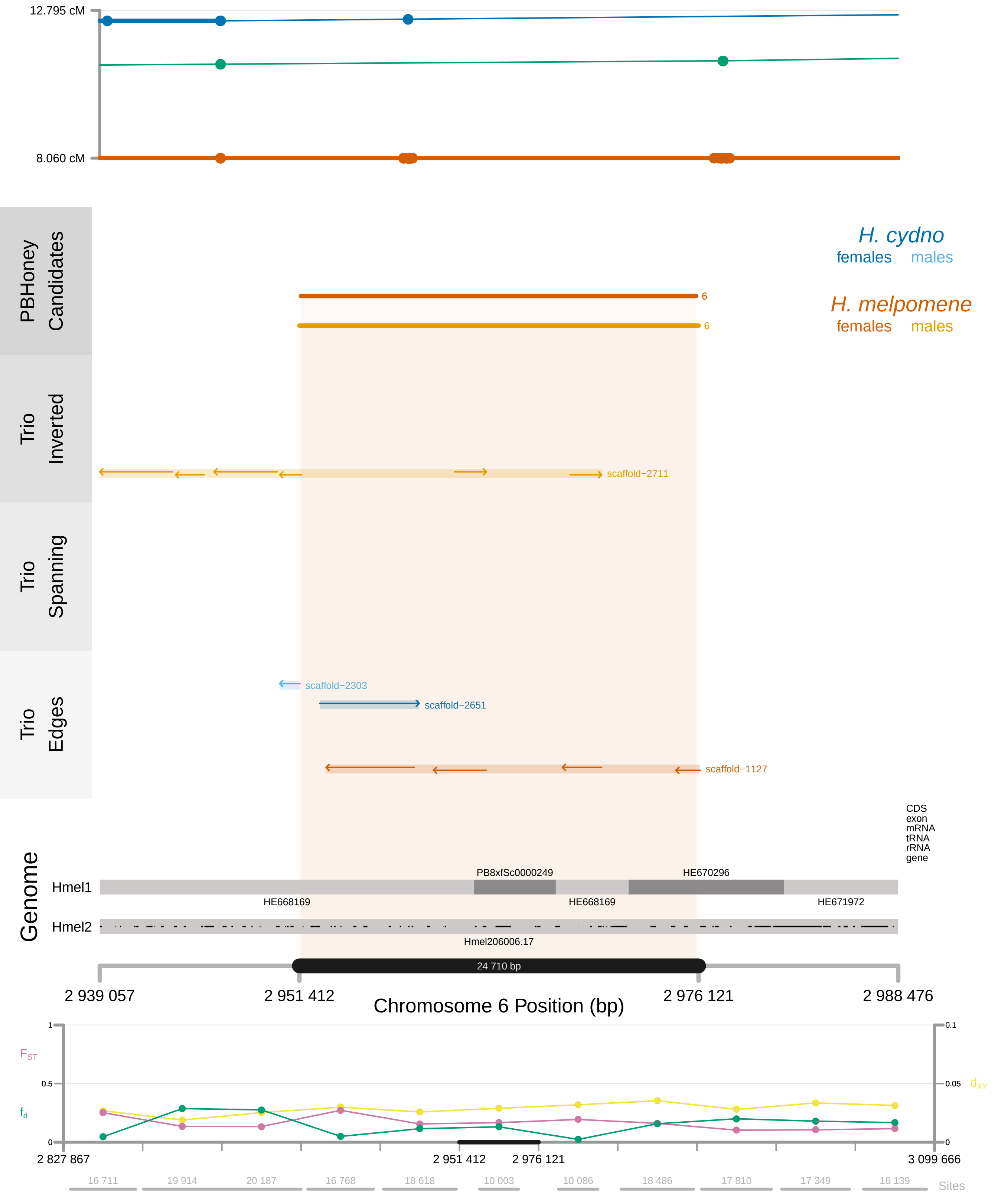

Figure S13.2 *H. melpomene* Split reads and trio assembly

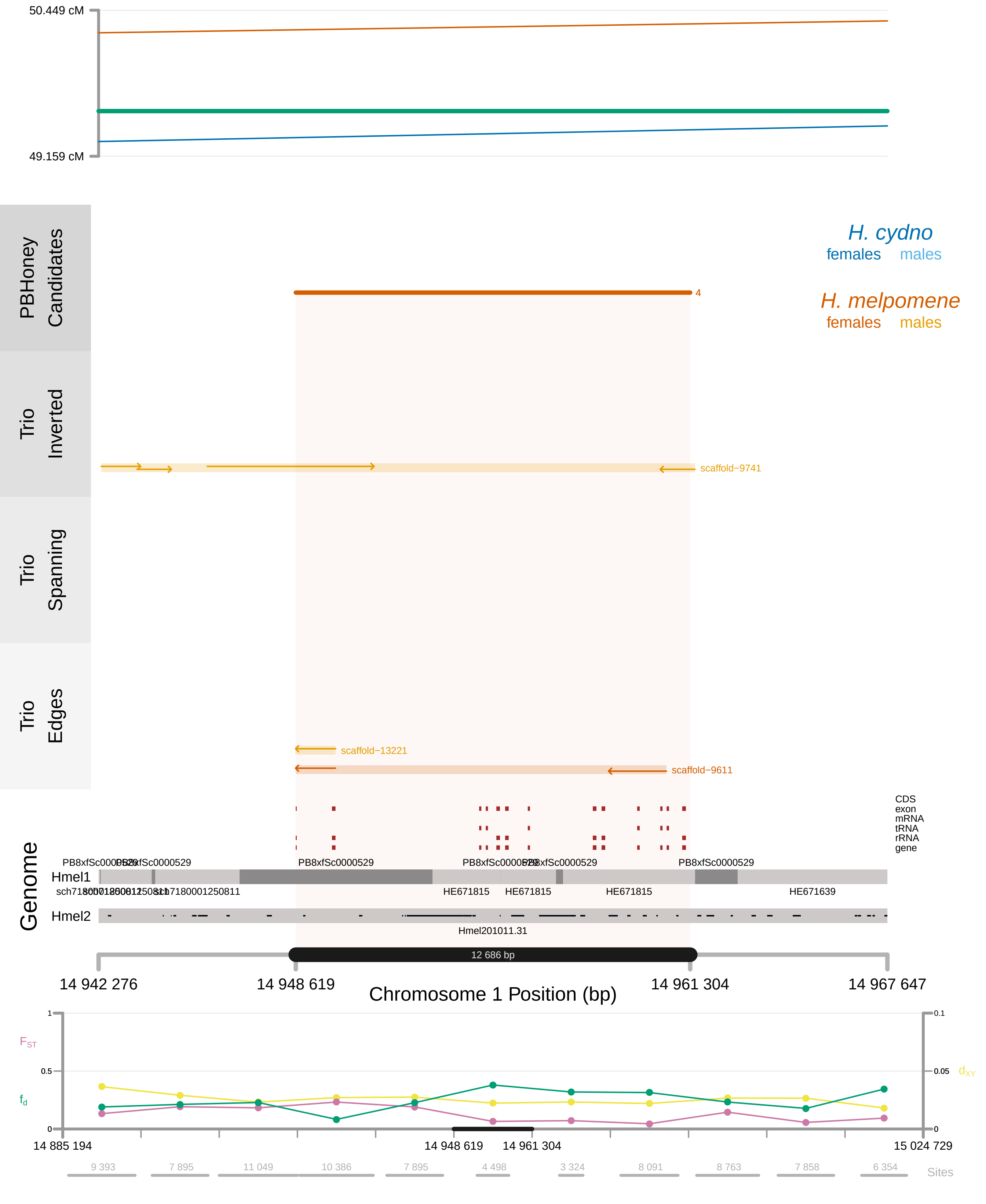

Figure S13.3 *H. melpomene* Split reads and trio assembly

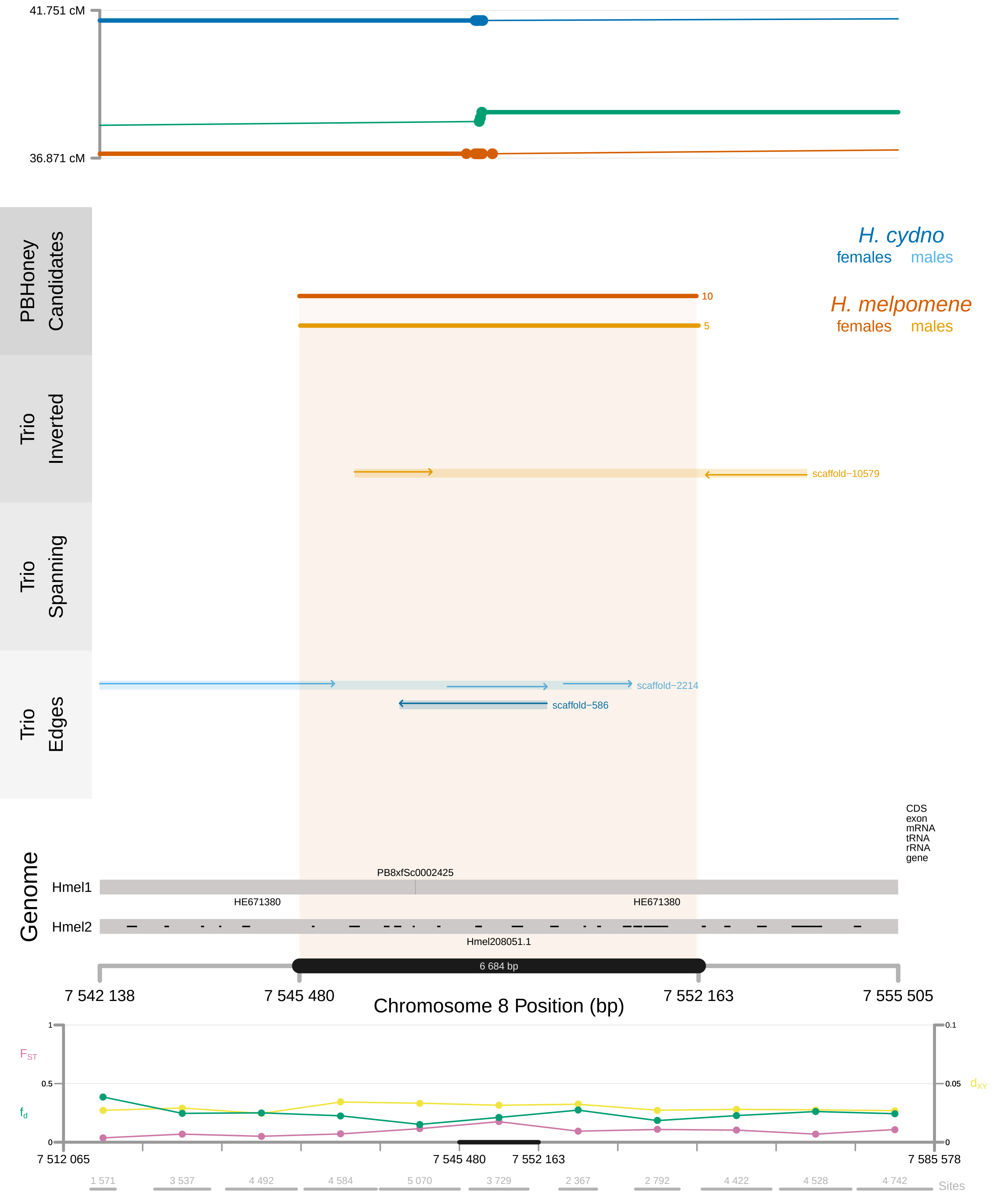

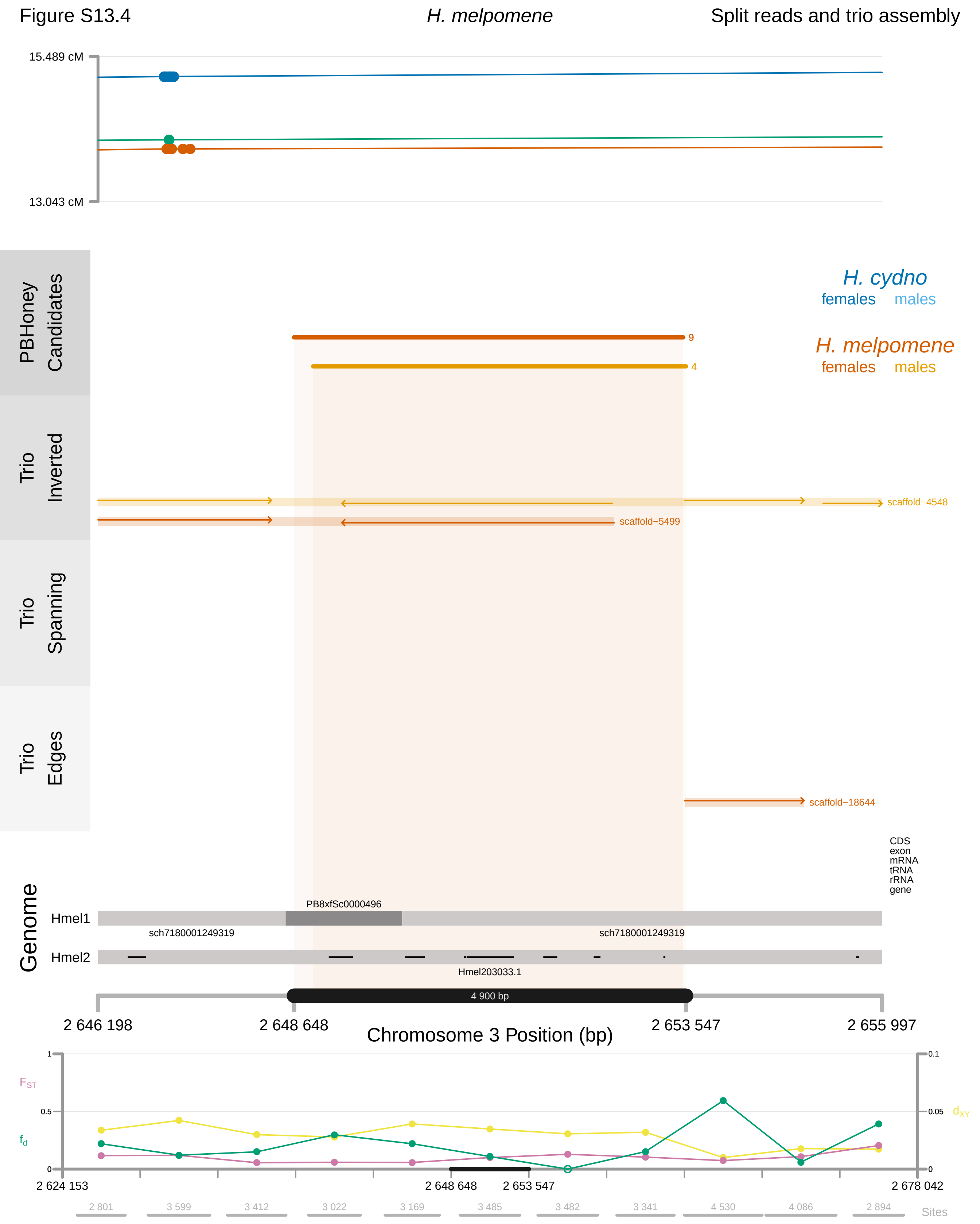

Figure S13.4
H. melpomene
Split reads and trio assembly
15.489 cM
13.043 cM
PBHoney Candidates
Trio Inverted
Trio Spanning
Trio Edges
H. cydno
females
males
H. melpomene
females
males
9
4
scaffold−4548
scaffold−5499
scaffold−18644
CDS
exon
mRNA
tRNA
rRNA
gene
Genome
PB8xfSc0000496
Hmel1
sch7180001249319
sch7180001249319
Hmel2
Hmel203033.1
4 900 bp
2 646 198
2 648 648
2 653 547
2 655 997
Chromosome 3 Position (bp)
F_ST
f_d
d_XY
1
0.5
0
0.1
0.05
0
2 624 153
2 648 648
2 653 547
2 678 042
2 801
3 599
3 412
3 022
3 169
3 485
3 482
3 341
4 530
4 086
2 894
Sites

Figure S13.5

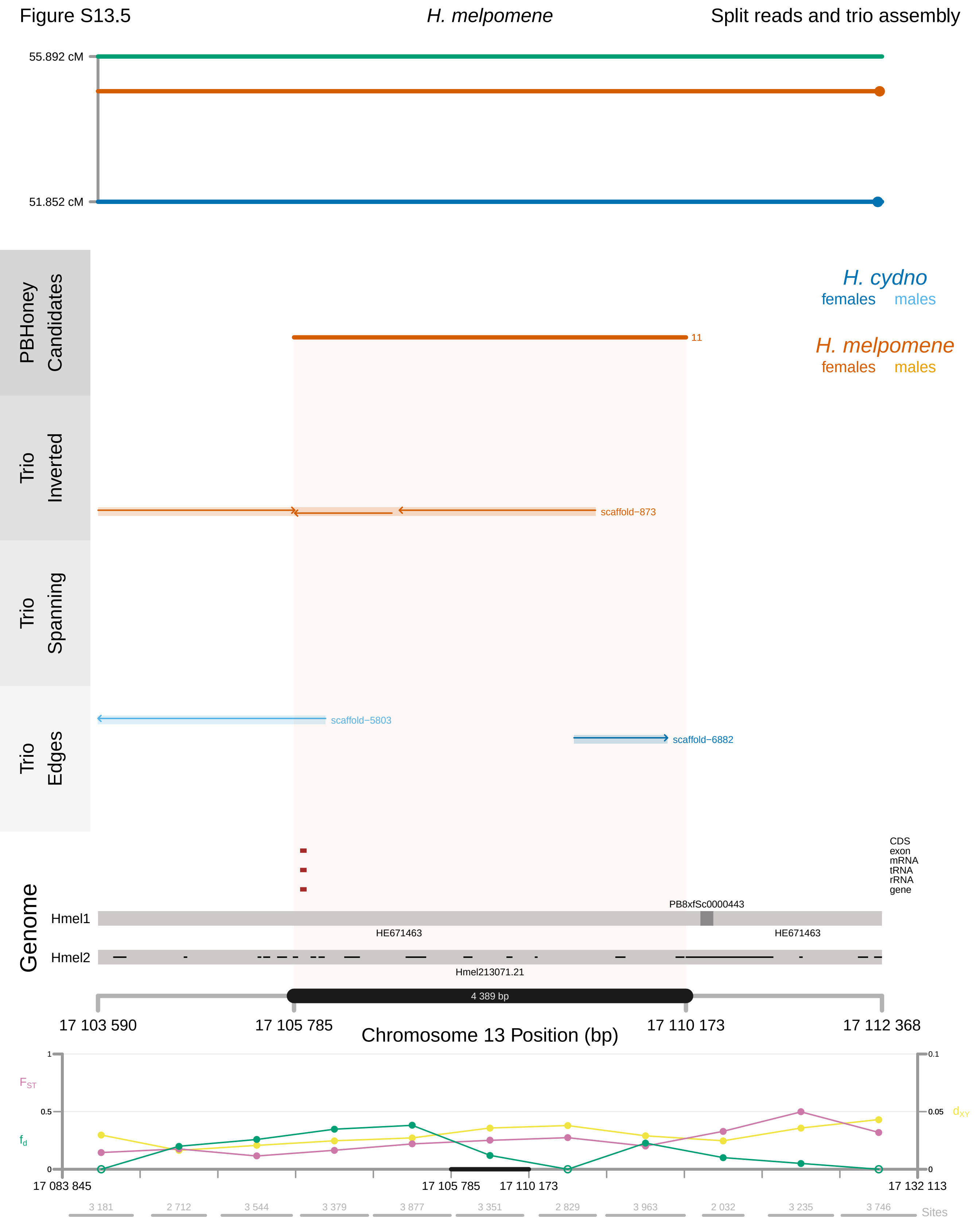
H. melpomene
Split reads and trio assembly
55.892 cM
51.852 cM
PBHoney Candidates
Trio Inverted
Trio Spanning
Trio Edges
Genome
H. cydno
females
males
H. melpomene
females
males
11
scaffold−873
scaffold−5803
scaffold−6882
CDS
exon
mRNA
tRNA
rRNA
gene
PB8xfSc0000443
Hmel1
HE671463
HE671463
Hmel2
Hmel213071.21
4 389 bp
17 103 590
17 105 785
17 110 173
17 112 368
Chromosome 13 Position (bp)
F_ST
f_d
d_XY
1
0.5
0
0.1
0.05
0
17 083 845
17 105 785
17 110 173
17 132 113
3 181
2 712
3 544
3 379
3 877
3 351
2 829
3 963
2 032
3 235
3 746
Sites

Figure S13.6 *H. melpomene* Split reads and trio assembly

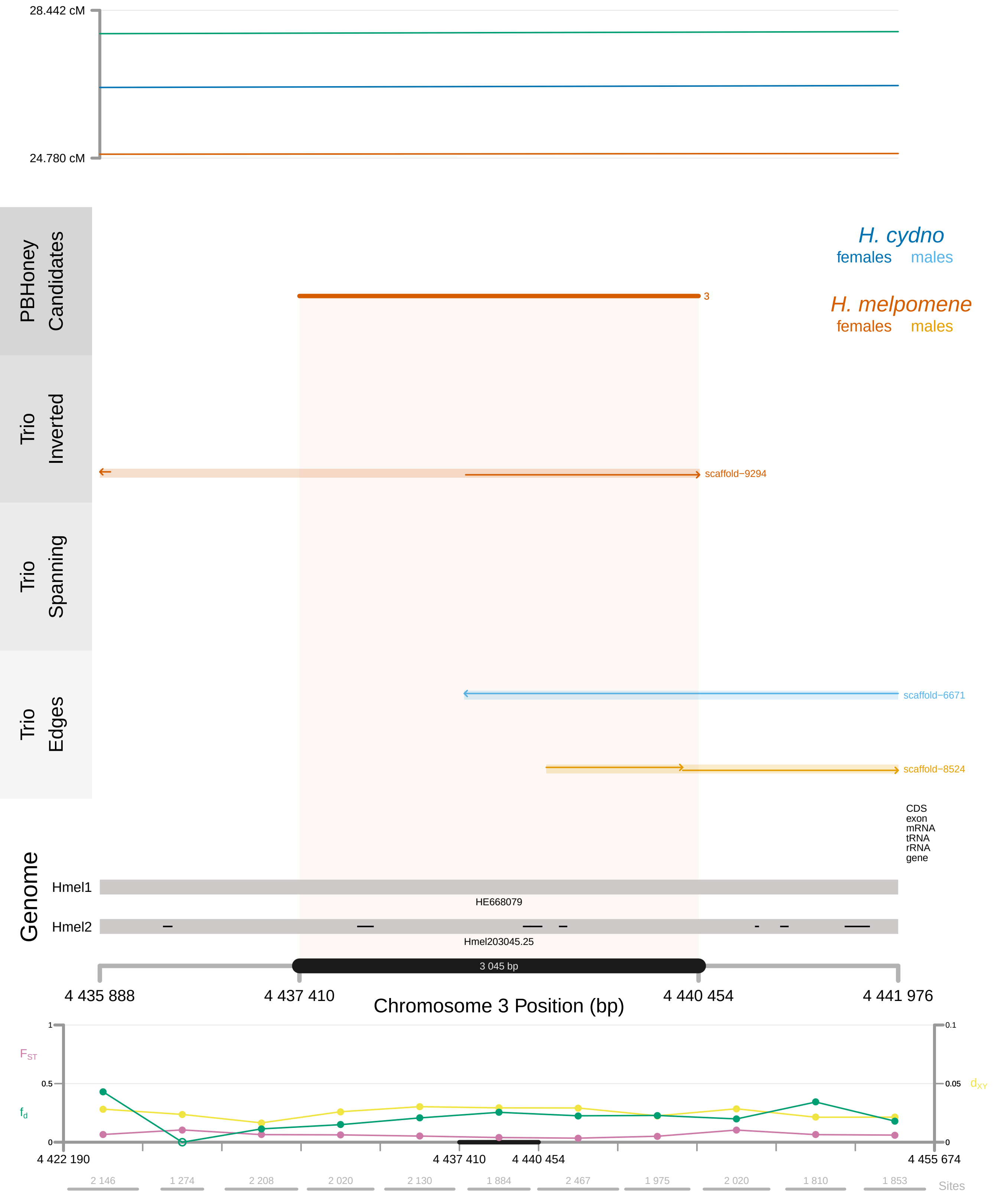

Figure S13.7 *H. melpomene* Split reads and trio assembly

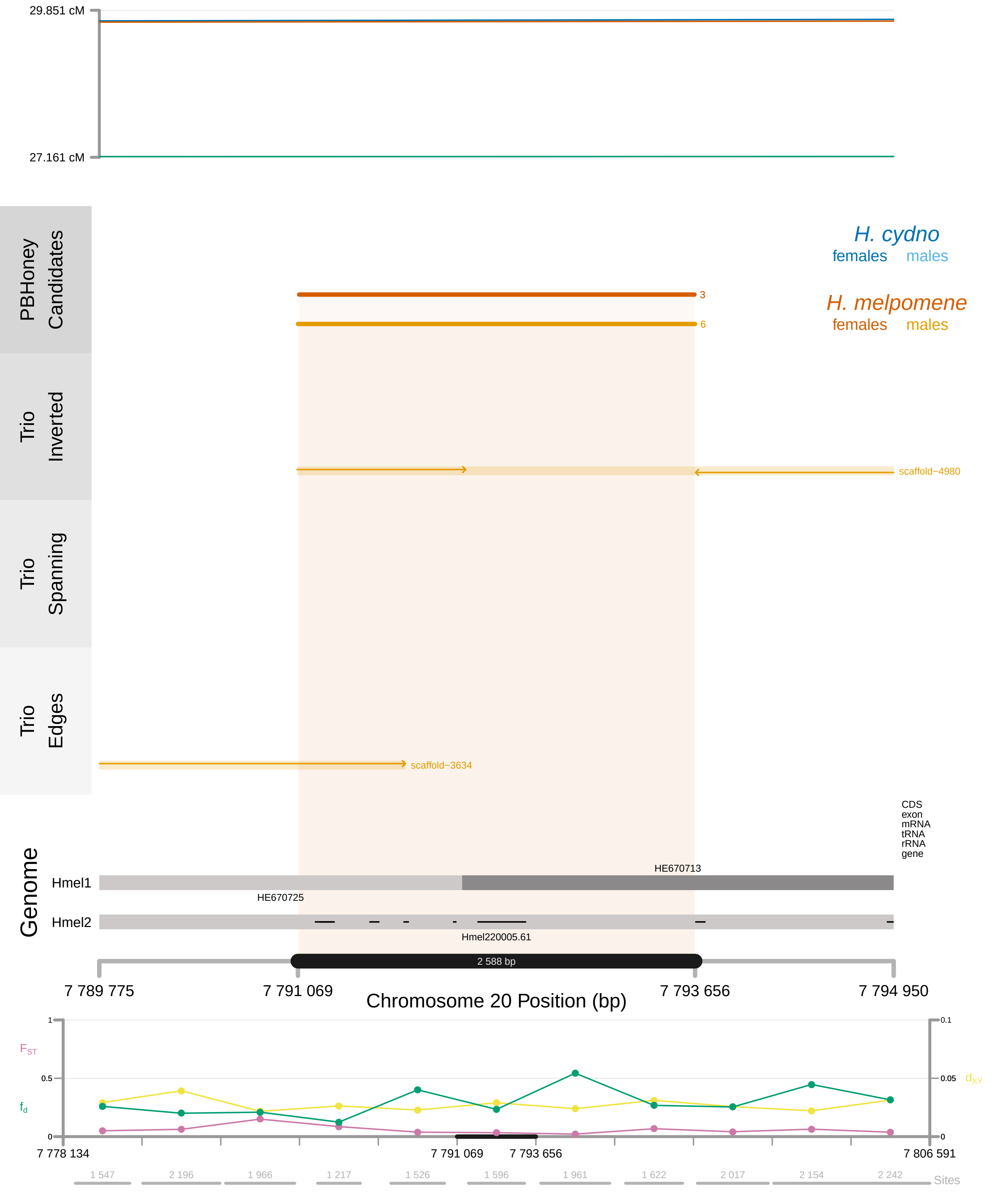

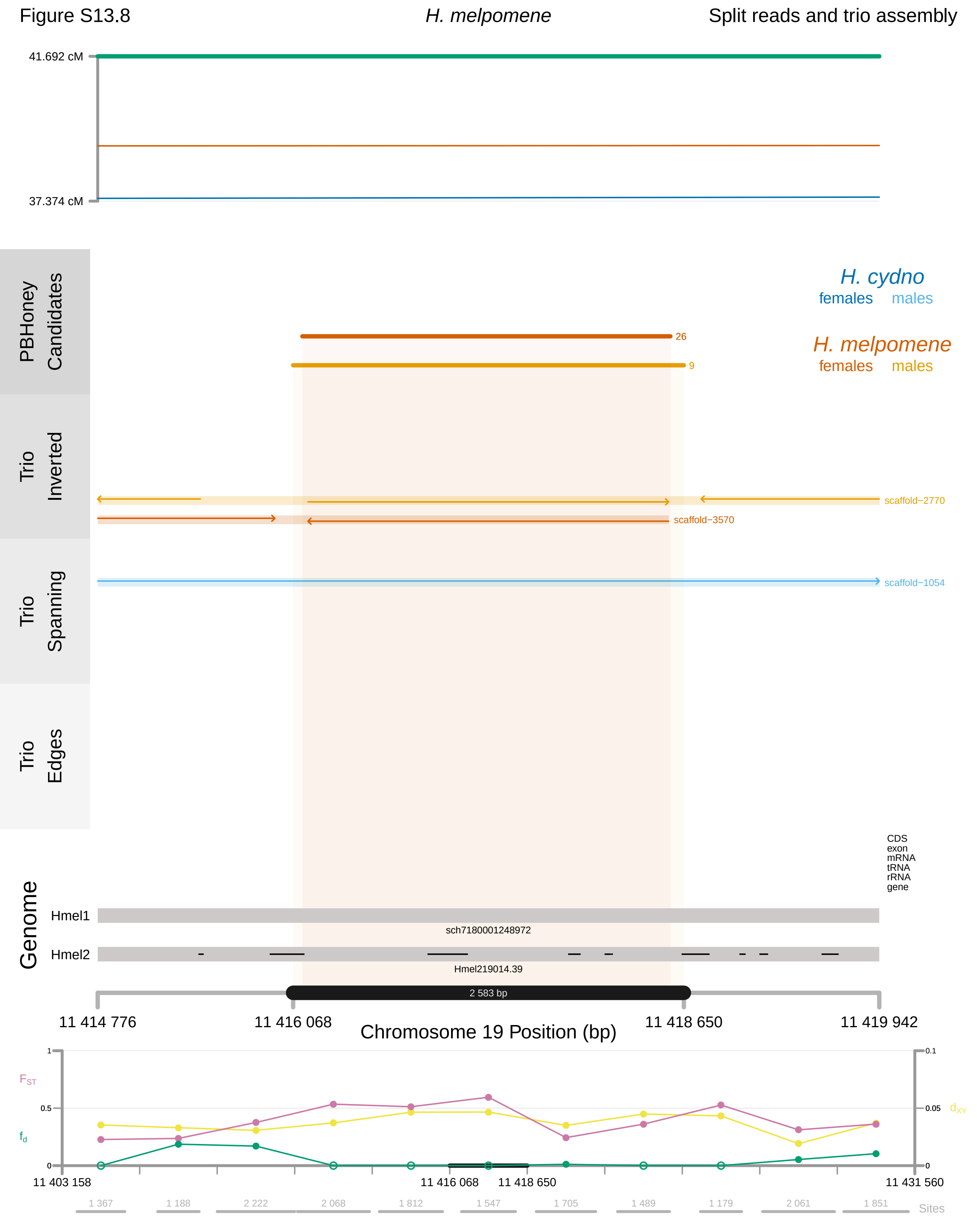
Figure S13.8
H. melpomene
Split reads and trio assembly
41.692 cM
37.374 cM
PBHoney Candidates
Trio Inverted
Trio Spanning
Trio Edges
Genome
H. cydno
females males
H. melpomene
females males
26
9
scaffold−2770
scaffold−3570
scaffold−1054
CDS
exon
mRNA
tRNA
rRNA
gene
Hmel1
sch7180001248972
Hmel2
Hmel219014.39
2 583 bp
11 414 776
11 416 068
11 418 650
11 419 942
Chromosome 19 Position (bp)
FST
fd
dXY
1
0.5
0
0.1
0.05
0
11 403 158
11 416 068
11 418 650
11 431 560
1 367
1 188
2 222
2 068
1 812
1 547
1 705
1 489
1 179
2 061
1 851
Sites

Figure S13.9 *H. melpomene* Split reads and trio assembly

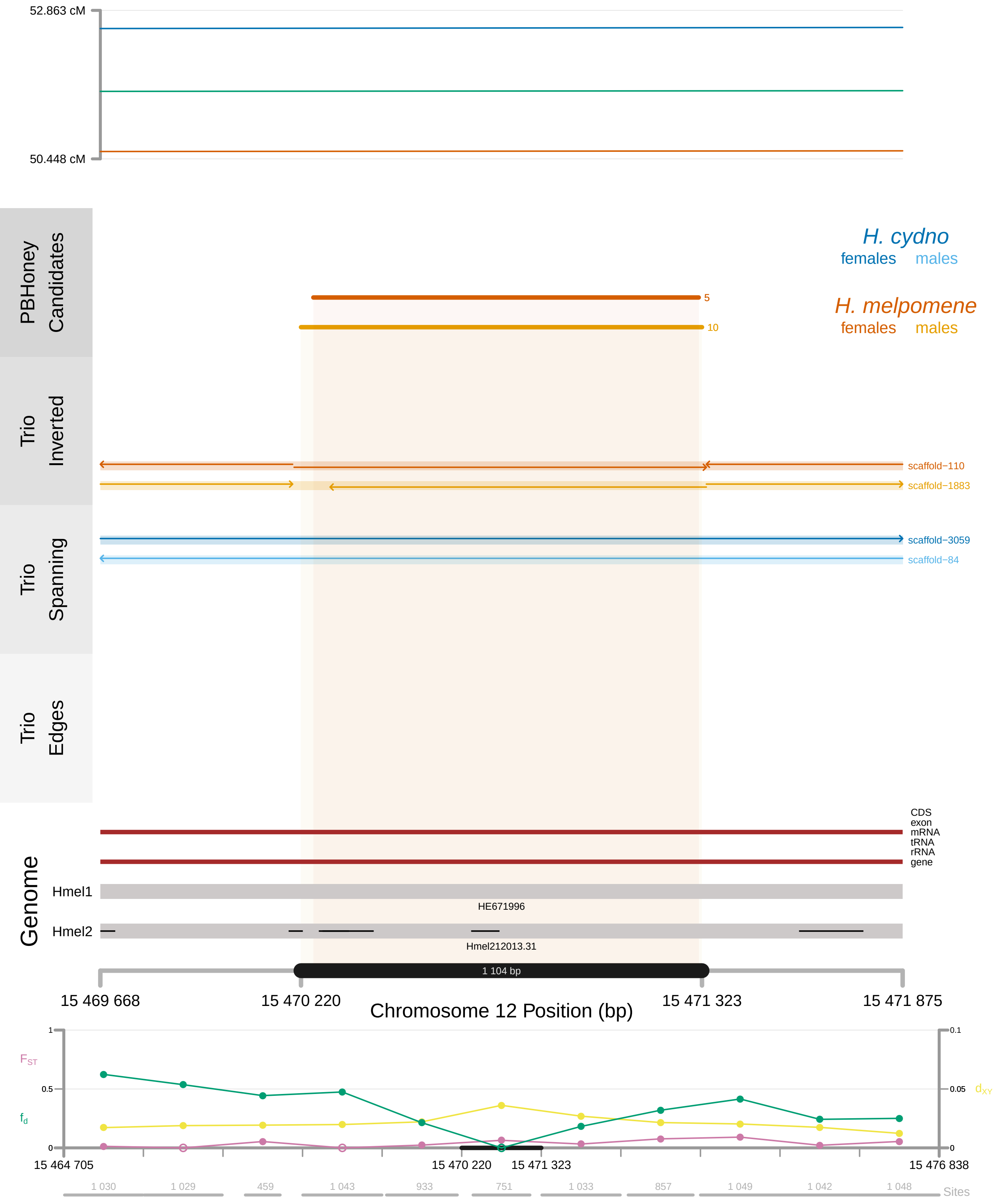

Supplement: Supplementary file 14 — S13, H. melpomene, split reads and trio assembly. [file EVL3-1-138-s014.pdf]
