# Supplementary material for: No evidence for maintenance of a sympatric Heliconius species barrier by chromosomal inversions
Source: Evol Lett. 2017 Jun 14;1(3):138–54. doi: 10.1002/evl3.12 (PMC6122123; doi:10.1002/evl3.12)

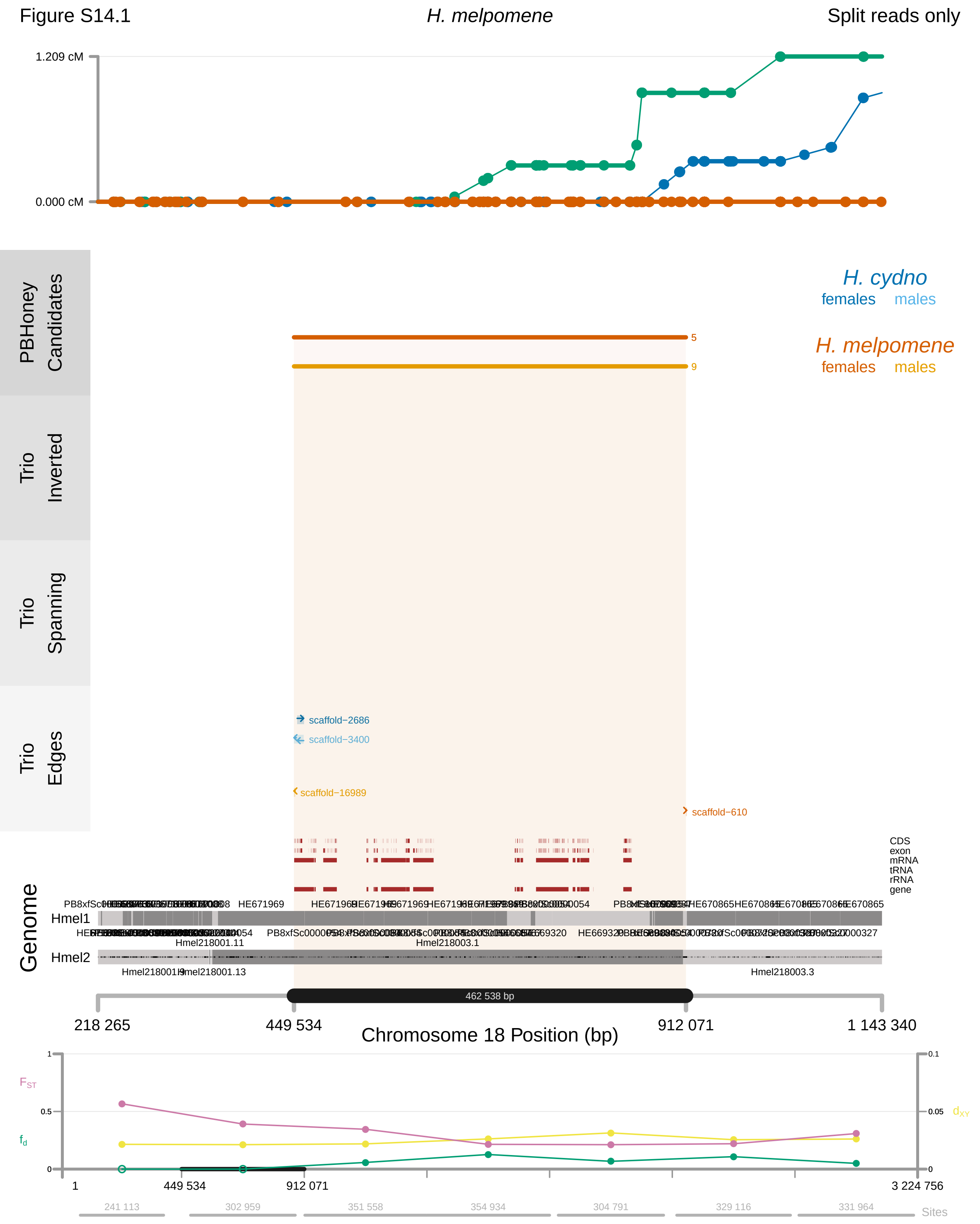

Figure S14.1
H. melpomene
Split reads only
1.209 cM
0.000 cM
PBHoney Candidates
Trio Inverted
Trio Spanning
Trio Edges
Genome
H. cydno
females males
H. melpomene
females males
5
9
scaffold−2686
scaffold−3400
scaffold−16989
scaffold−610
CDS
exon
mRNA
tRNA
rRNA
gene
Hmel1
Hmel2
Hmel218001.11
Hmel218003.1
Hmel218001.13
Hmel218003.3
462 538 bp
218 265
449 534
912 071
1 143 340
Chromosome 18 Position (bp)
1
0.5
0
0.1
0.05
F_{ST}
f_d
d_{XY}
1
449 534
912 071
3 224 756
241 113
302 959
351 558
354 934
304 791
329 116
331 964
Sites

Figure S14.2 *H. melpomene* Split reads only

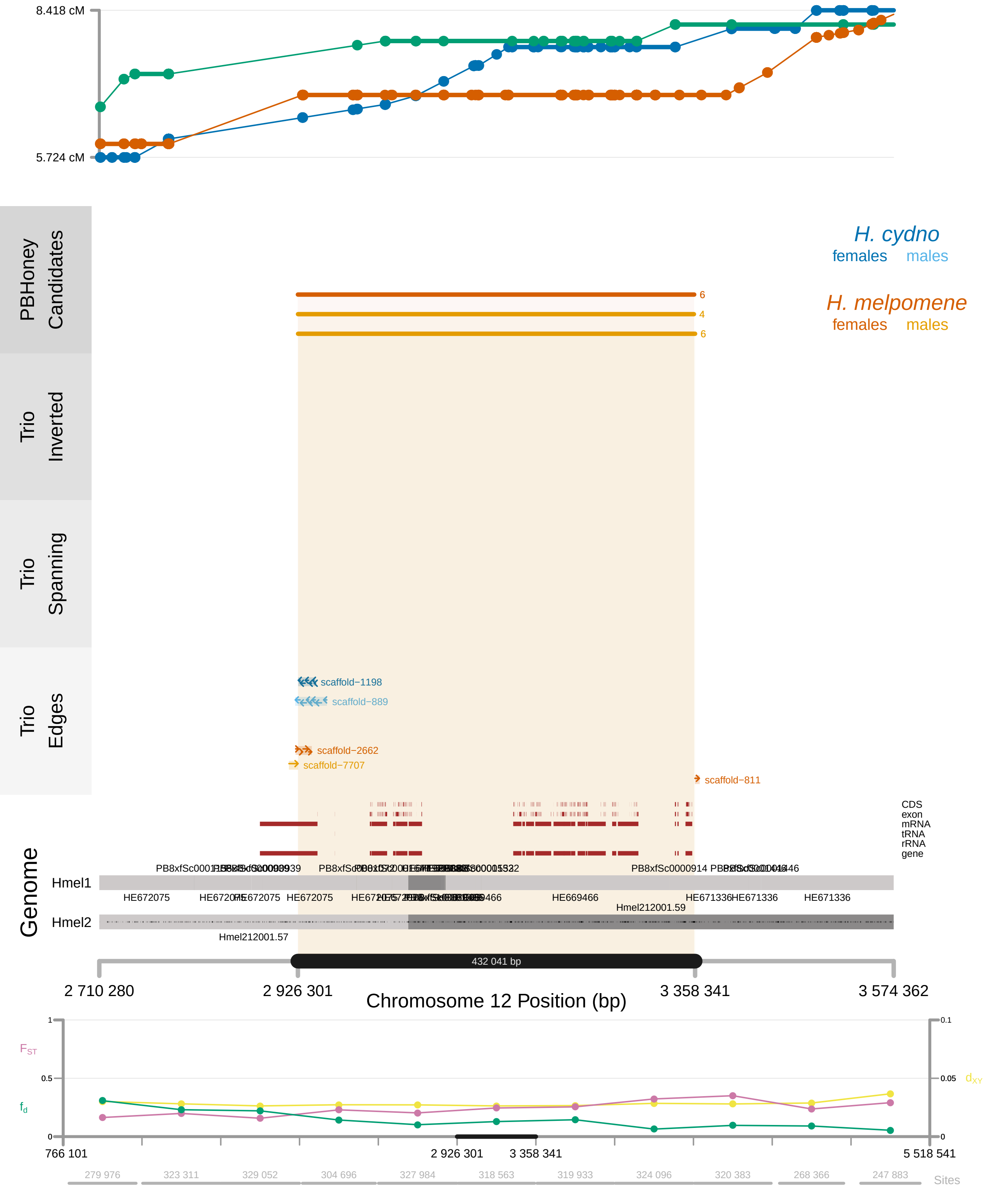

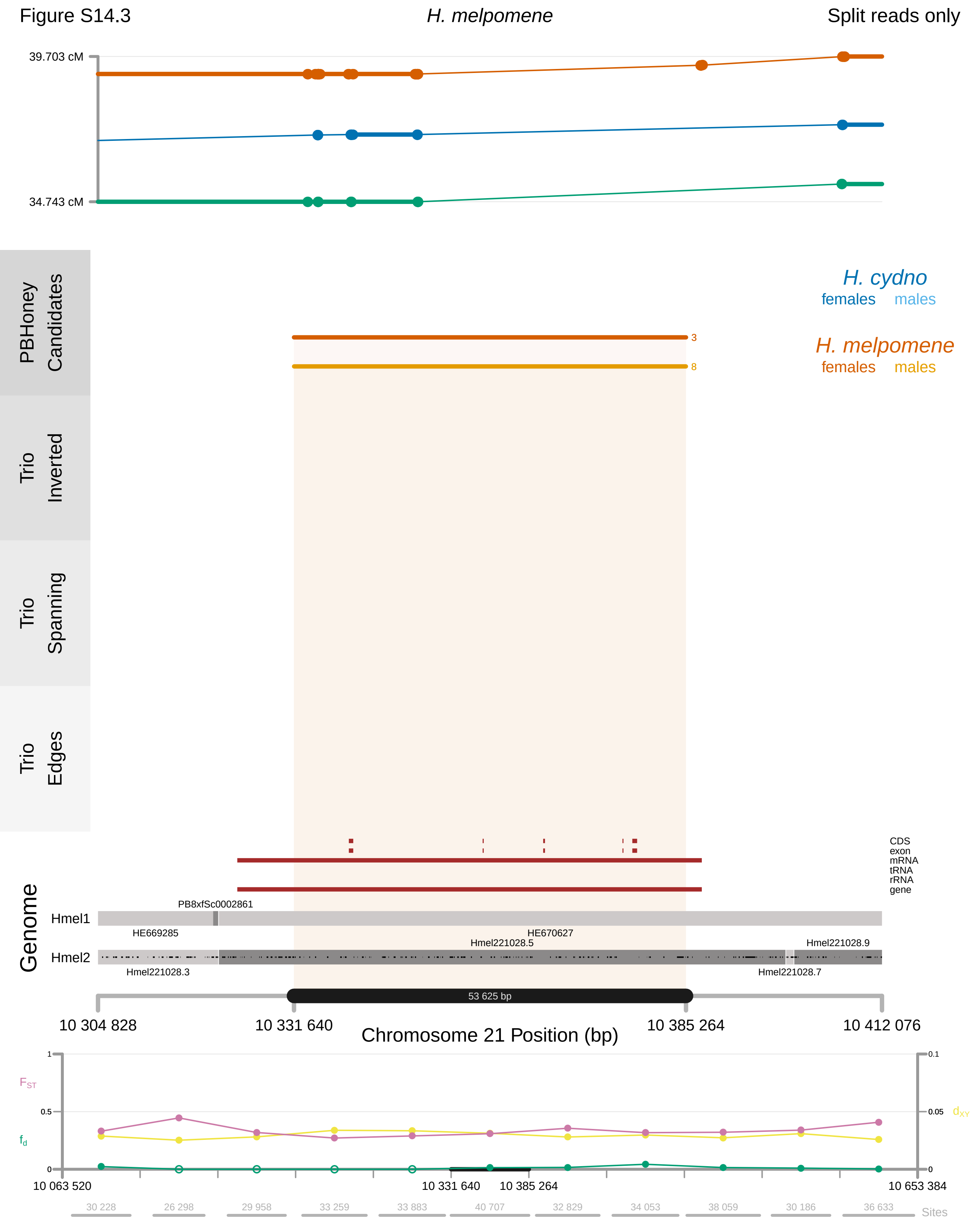

Figure S14.3
H. melpomene
Split reads only
39.703 cM
34.743 cM
PBHoney Candidates
Trio Inverted
Trio Spanning
Trio Edges
H. cydno
females
males
H. melpomene
females
males
3
8
CDS
exon
mRNA
tRNA
rRNA
gene
Genome
PB8xfSc0002861
Hmel1
HE669285
HE670627
Hmel221028.5
Hmel221028.9
Hmel2
Hmel221028.3
Hmel221028.7
53 625 bp
10 304 828
10 331 640
10 385 264
10 412 076
Chromosome 21 Position (bp)
1
0.5
0
FST
fd
0.1
0.05
0
dXY
10 063 520
10 331 640
10 385 264
10 653 384
30 228
26 298
29 958
33 259
33 883
40 707
32 829
34 053
38 059
30 186
36 633
Sites

Figure S14.4 *H. melpomene* Split reads only

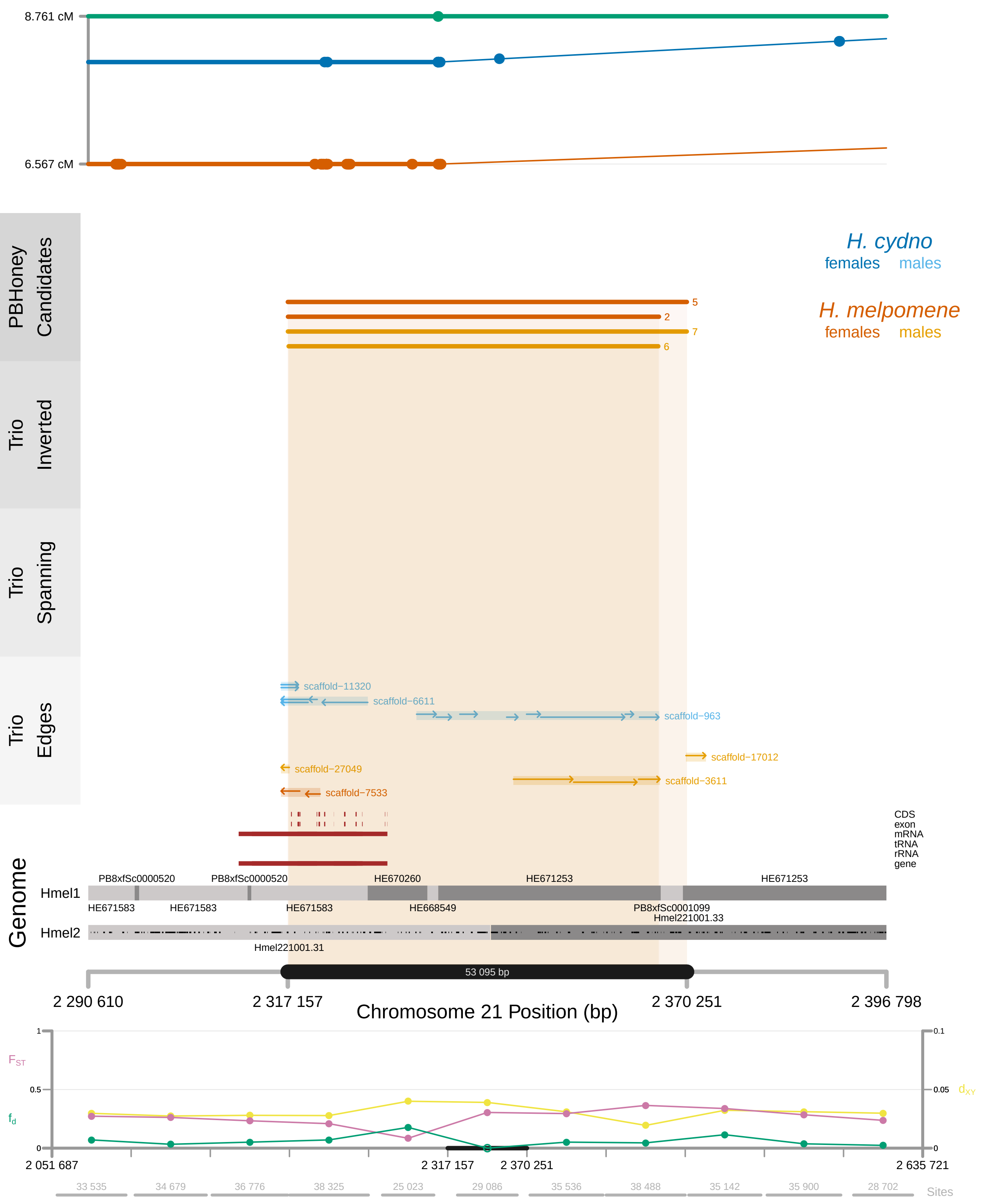

Figure S14.5 *H. melpomene* Split reads only

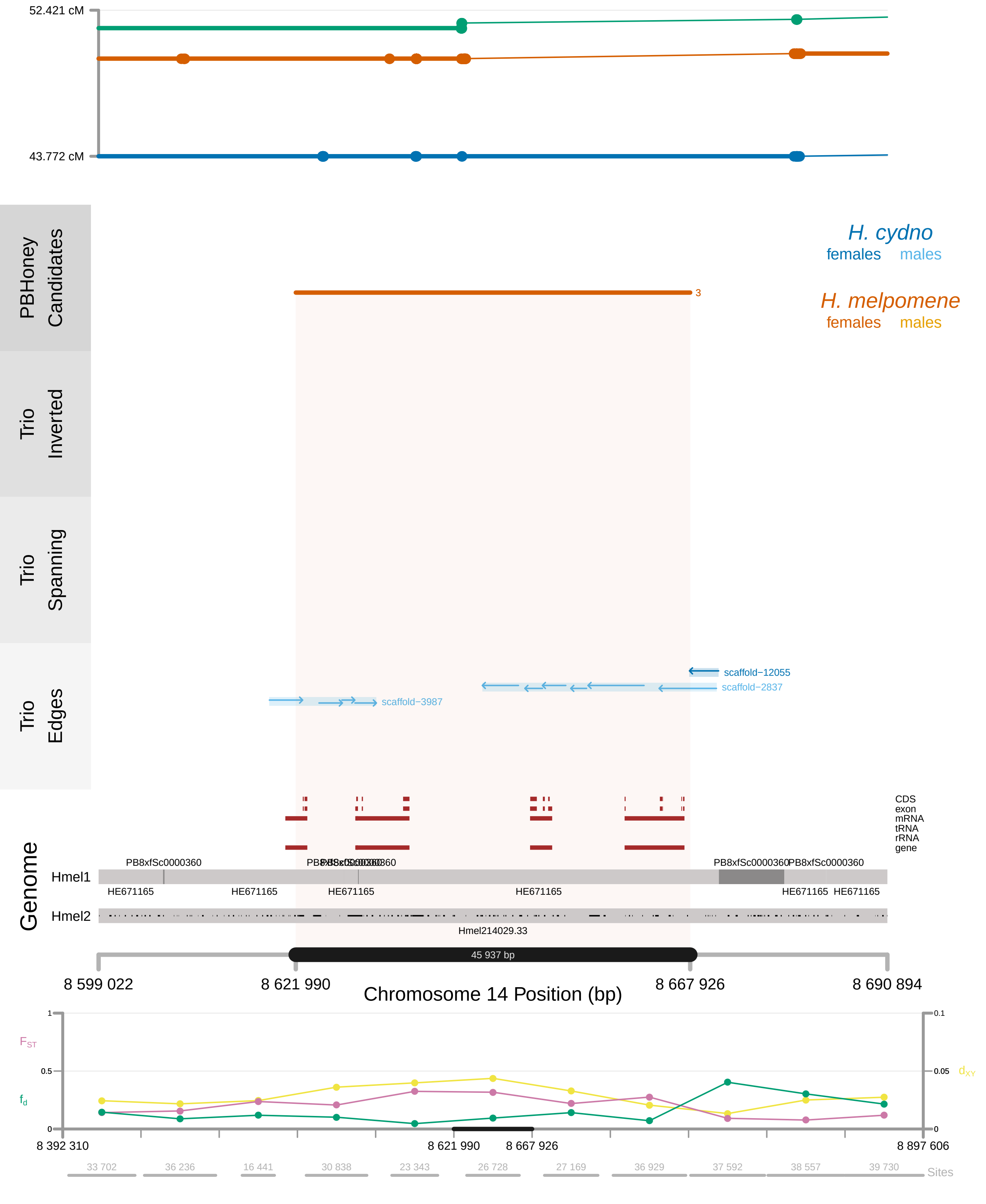

Figure S14.6

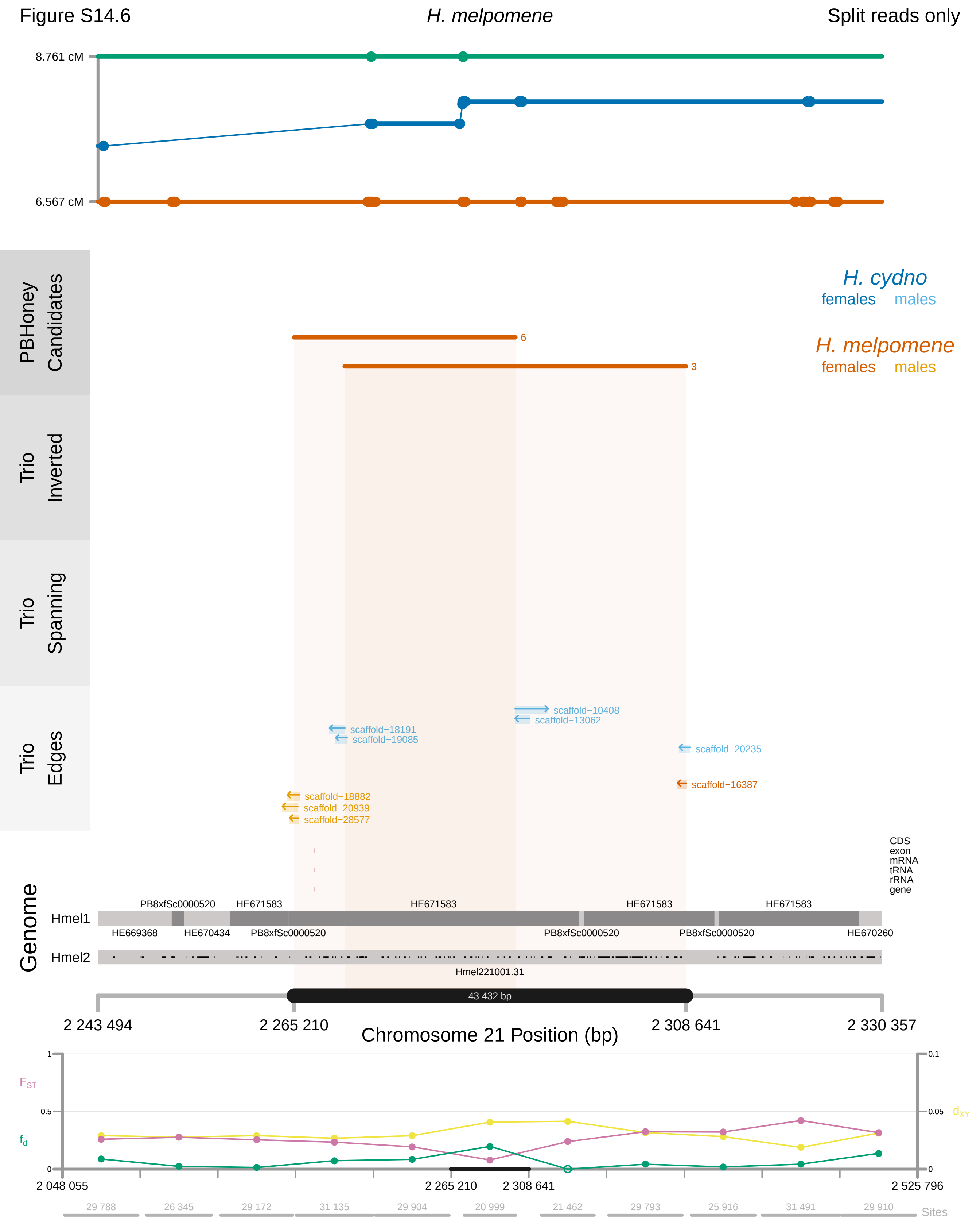
H. melpomene
Split reads only
8.761 cM
6.567 cM
PBHoney Candidates
Trio Inverted
Trio Spanning
Trio Edges
H. cydno
females males
H. melpomene
females males
6
3
scaffold−10408
scaffold−13062
scaffold−18191
scaffold−19085
scaffold−20235
scaffold−16387
scaffold−18882
scaffold−20939
scaffold−28577
CDS
exon
mRNA
tRNA
rRNA
gene
Genome
Hmel1
Hmel2
PB8xfSc0000520
HE671583
HE671583
HE671583
HE671583
HE669368
HE670434
PB8xfSc0000520
PB8xfSc0000520
PB8xfSc0000520
HE670260
Hmel221001.31
43 432 bp
2 243 494
2 265 210
2 308 641
2 330 357
Chromosome 21 Position (bp)
F_ST
f_d
d_XY
1
0.5
0
0.1
0.05
0
2 048 055
2 265 210
2 308 641
2 525 796
29 788
26 345
29 172
31 135
29 904
20 999
21 462
29 793
25 916
31 491
29 910
Sites

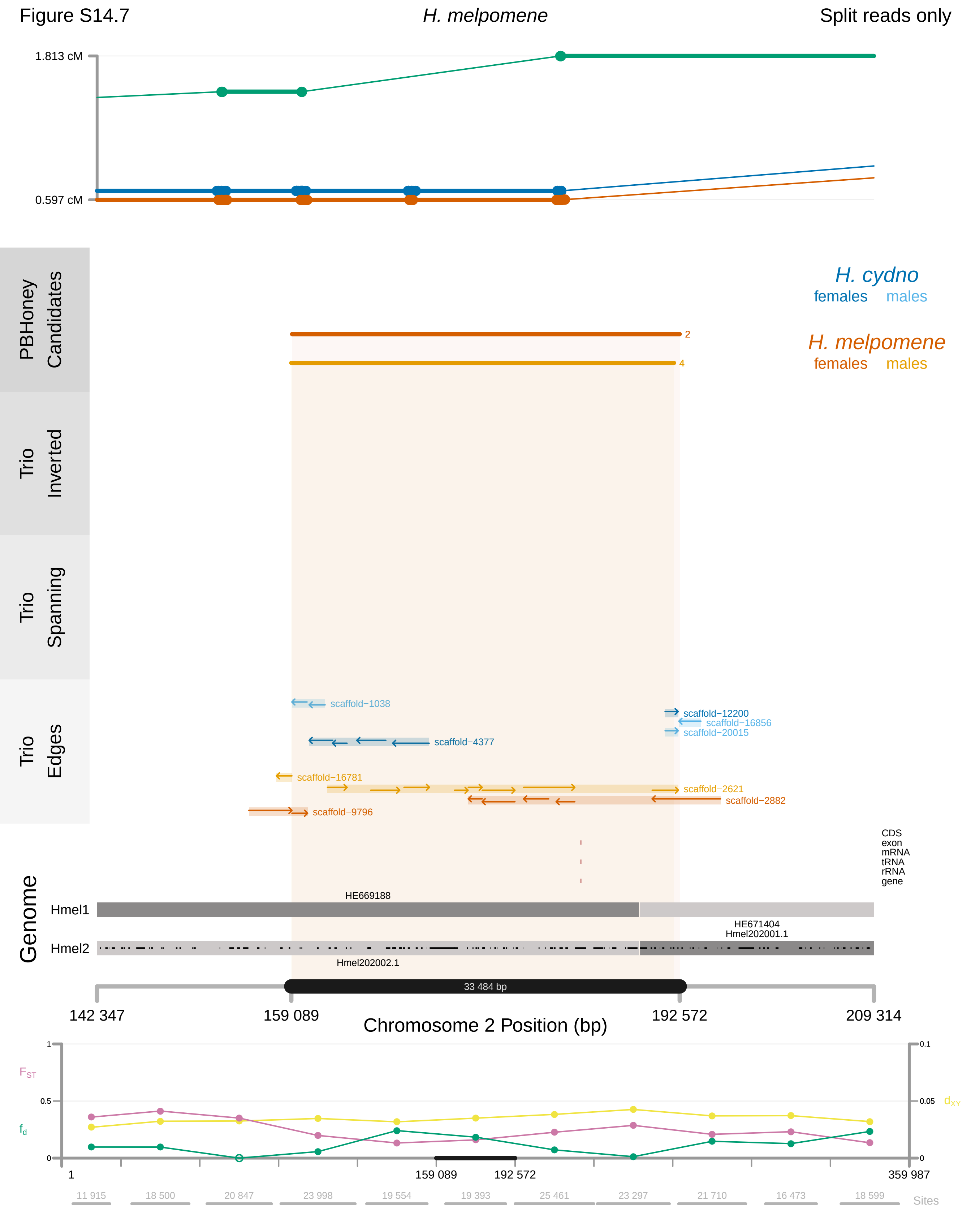

Figure S14.7
H. melpomene
Split reads only
1.813 cM
0.597 cM
PBHoney Candidates
Trio Inverted
Trio Spanning
Trio Edges
H. cydno
females males
H. melpomene
females males
2
4
scaffold−1038
scaffold−12200
scaffold−16856
scaffold−20015
scaffold−4377
scaffold−16781
scaffold−2621
scaffold−2882
scaffold−9796
CDS
exon
mRNA
tRNA
rRNA
gene
Genome
HE669188
Hmel1
HE671404
Hmel202001.1
Hmel2
Hmel202002.1
33 484 bp
142 347
159 089
192 572
209 314
Chromosome 2 Position (bp)
1
0.5
0
FST
fd
0.1
0.05
0
dXY
1
159 089
192 572
359 987
11 915
18 500
20 847
23 998
19 554
19 393
25 461
23 297
21 710
16 473
18 599
Sites

Figure S14.8 *H. melpomene* Split reads only

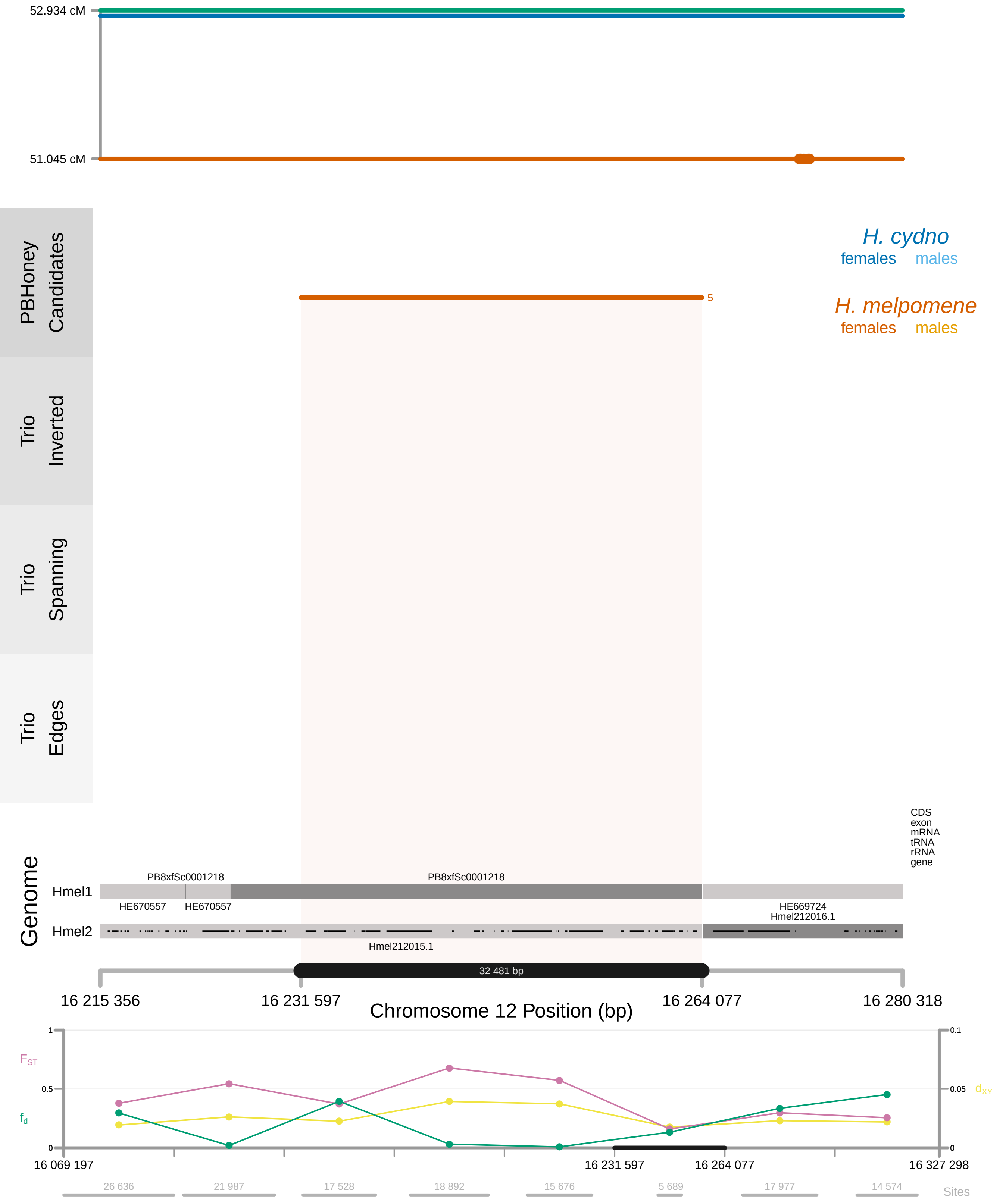

Figure S14.9 *H. melpomene* Split reads only

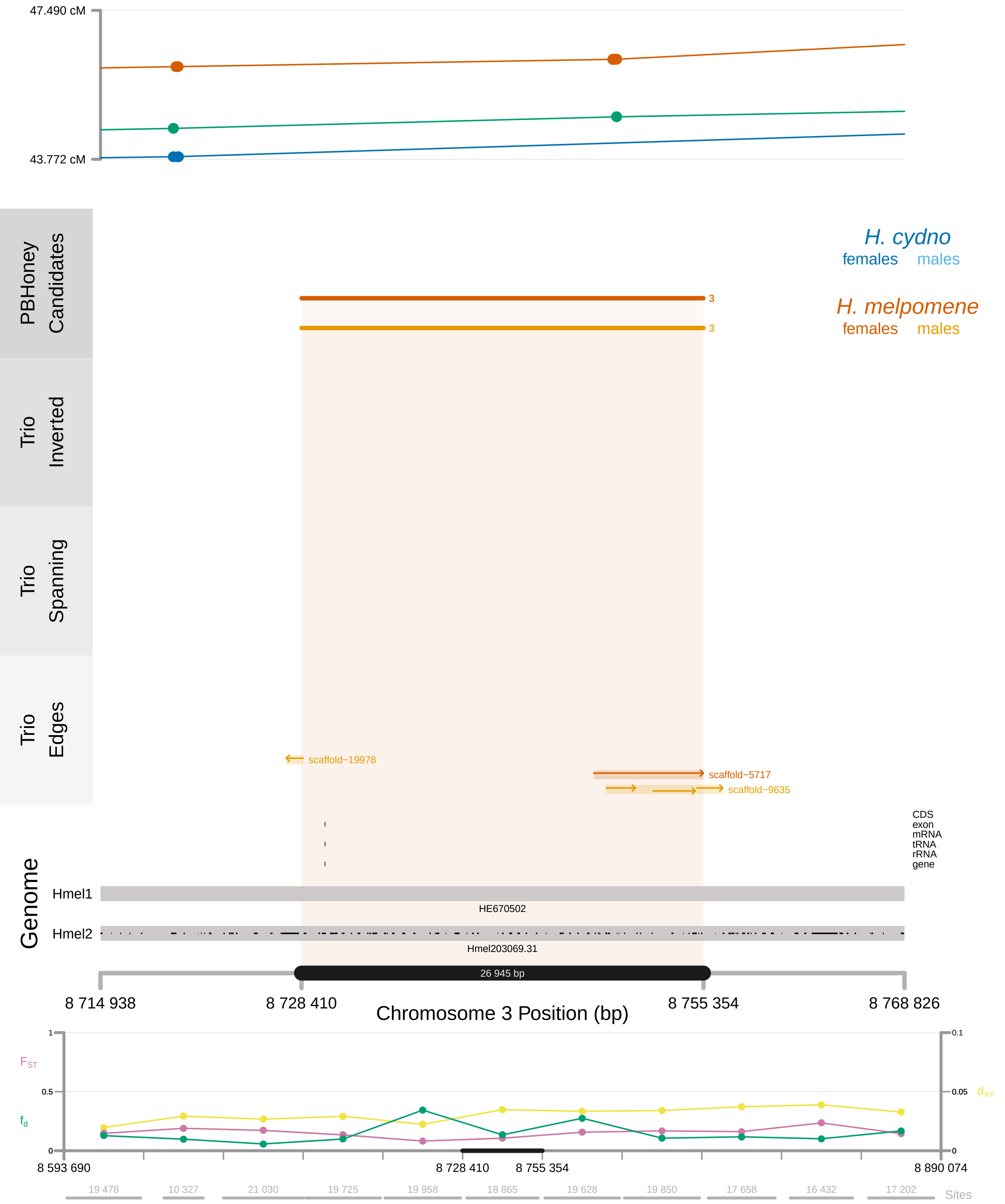

Figure S14.10 *H. melpomene* Split reads only

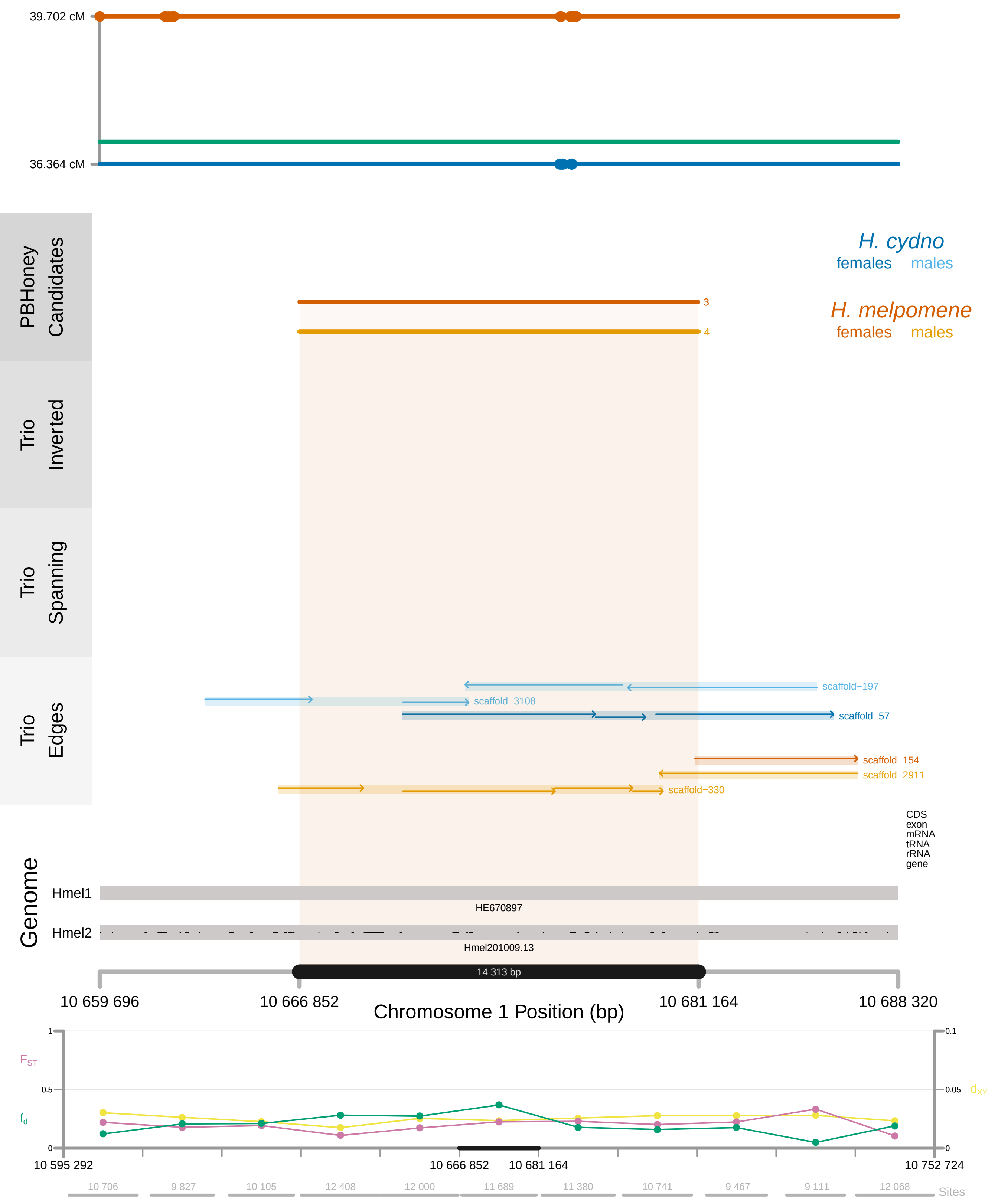

Figure S14.11 *H. melpomene* Split reads only

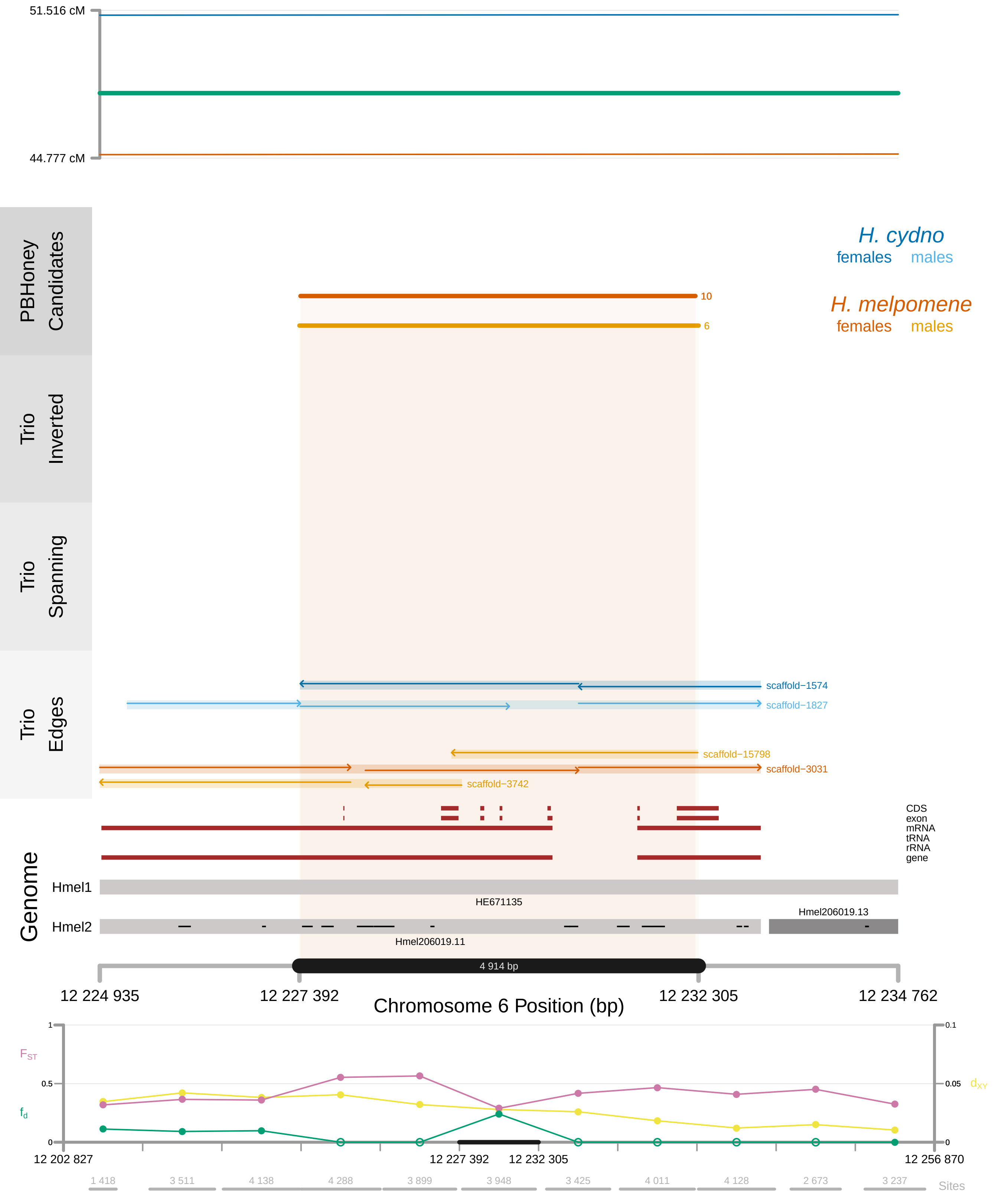

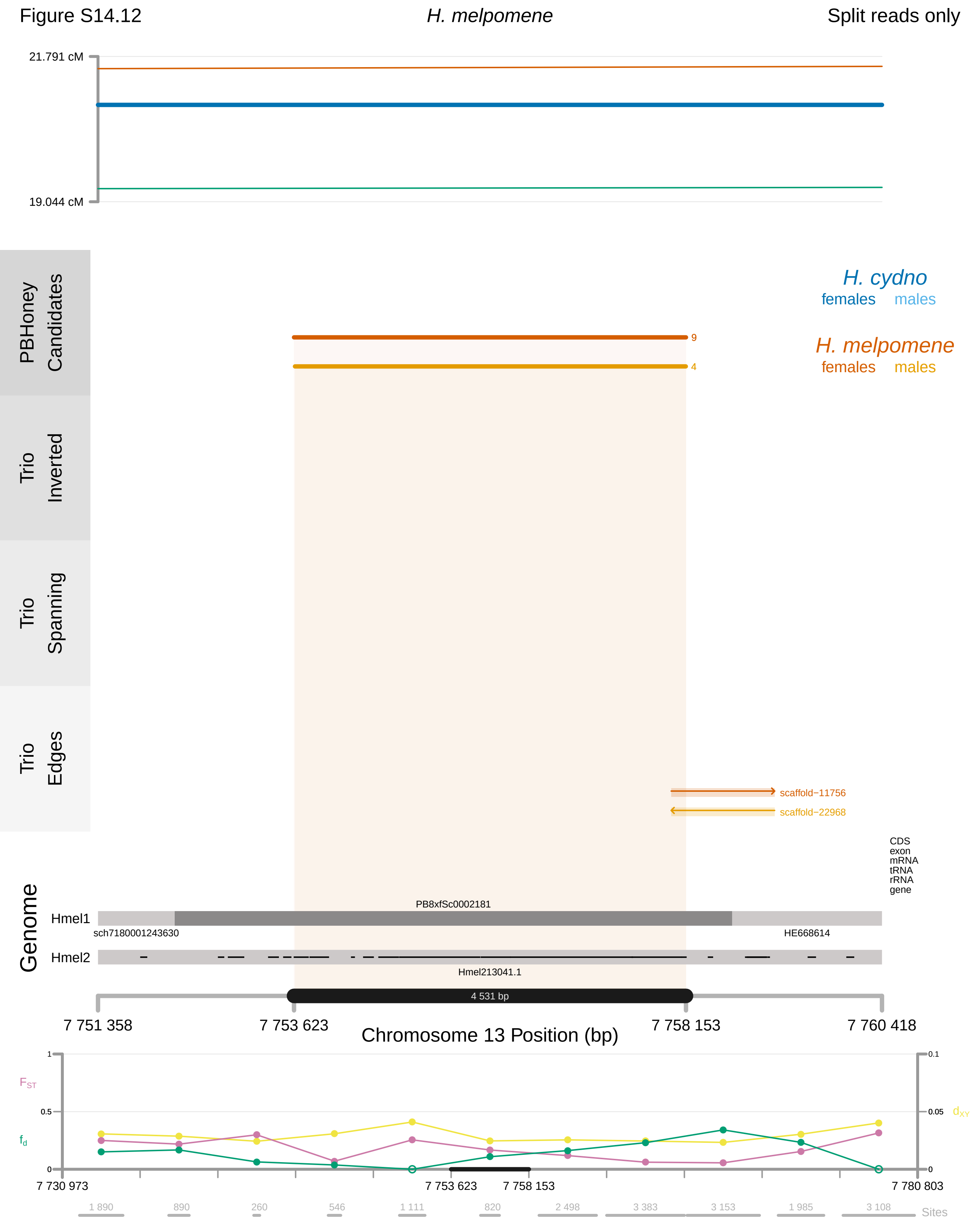

Figure S14.12
H. melpomene
Split reads only
21.791 cM
19.044 cM
PBHoney Candidates
Trio Inverted
Trio Spanning
Trio Edges
Genome
H. cydno
females
males
H. melpomene
females
males
9
4
scaffold−11756
scaffold−22968
CDS
exon
mRNA
tRNA
rRNA
gene
PB8xfSc0002181
Hmel1
sch7180001243630
HE668614
Hmel2
Hmel213041.1
4 531 bp
7 751 358
7 753 623
7 758 153
7 760 418
Chromosome 13 Position (bp)
1
0.5
0
FST
fd
0.1
0.05
0
dXY
7 730 973
7 753 623
7 758 153
7 780 803
1 890
890
260
546
1 111
820
2 498
3 383
3 153
1 985
3 108
Sites

Figure S14.13 *H. melpomene* Split reads only

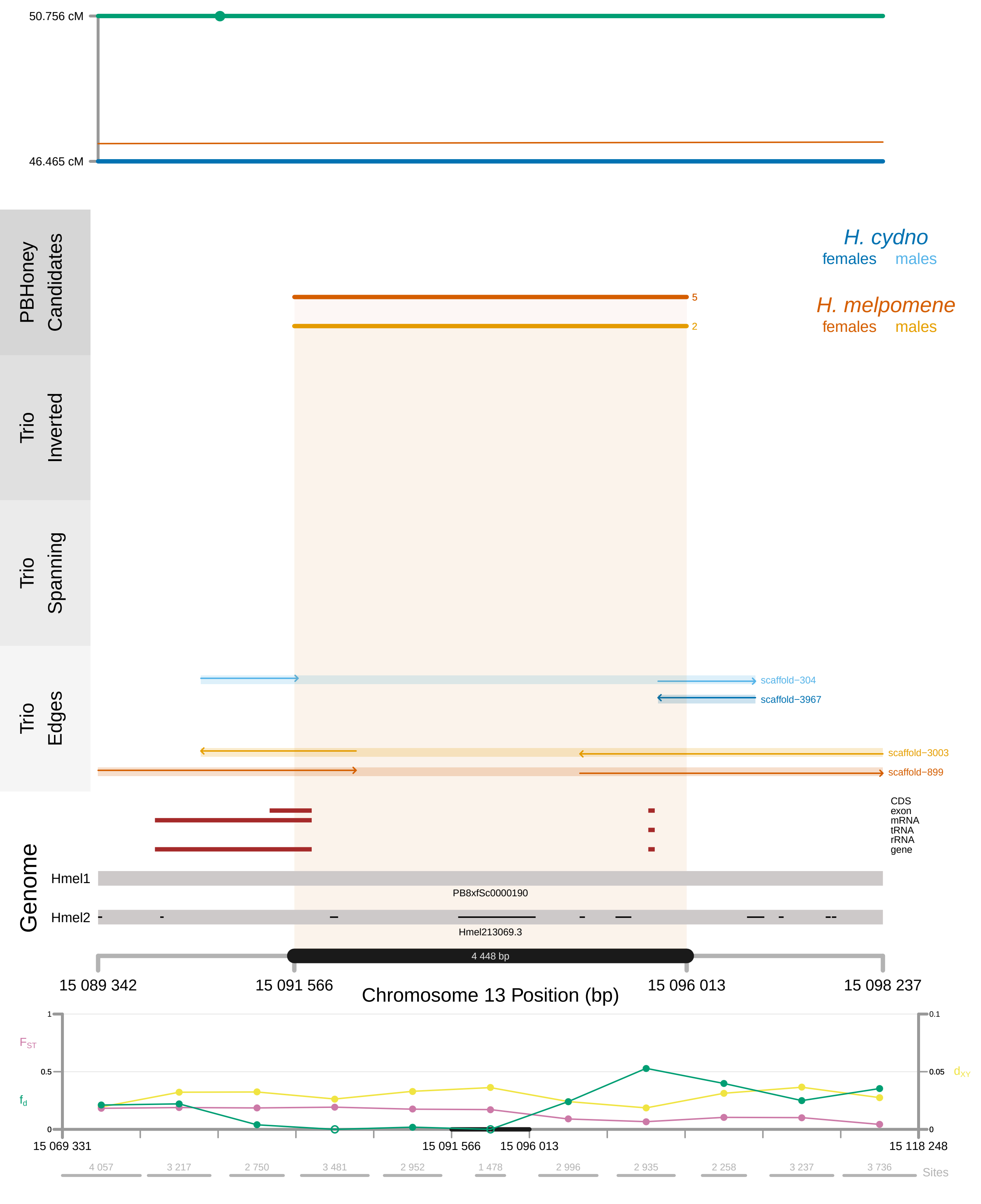

Figure S14.14 *H. melpomene* Split reads only

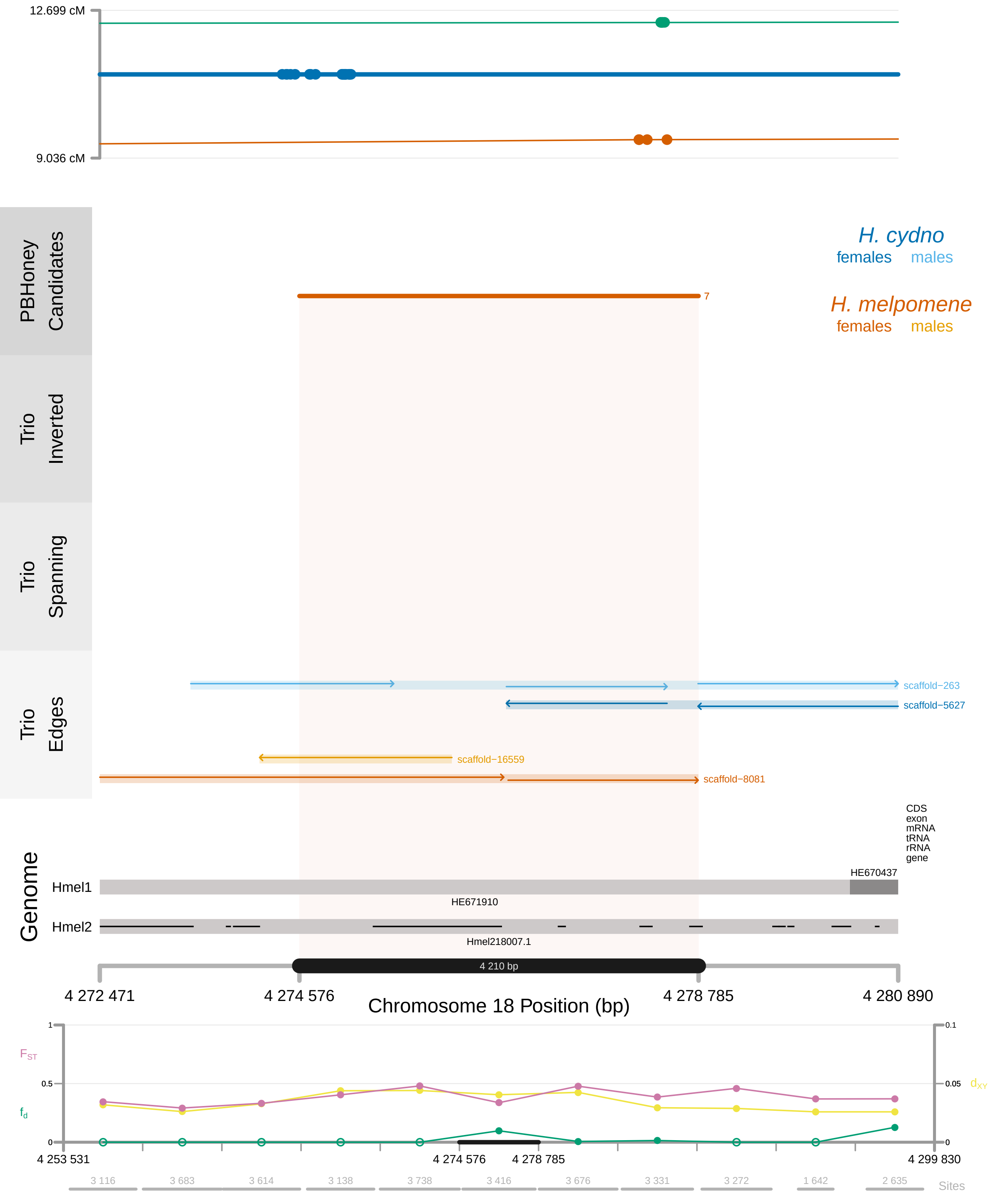

Figure S14.15

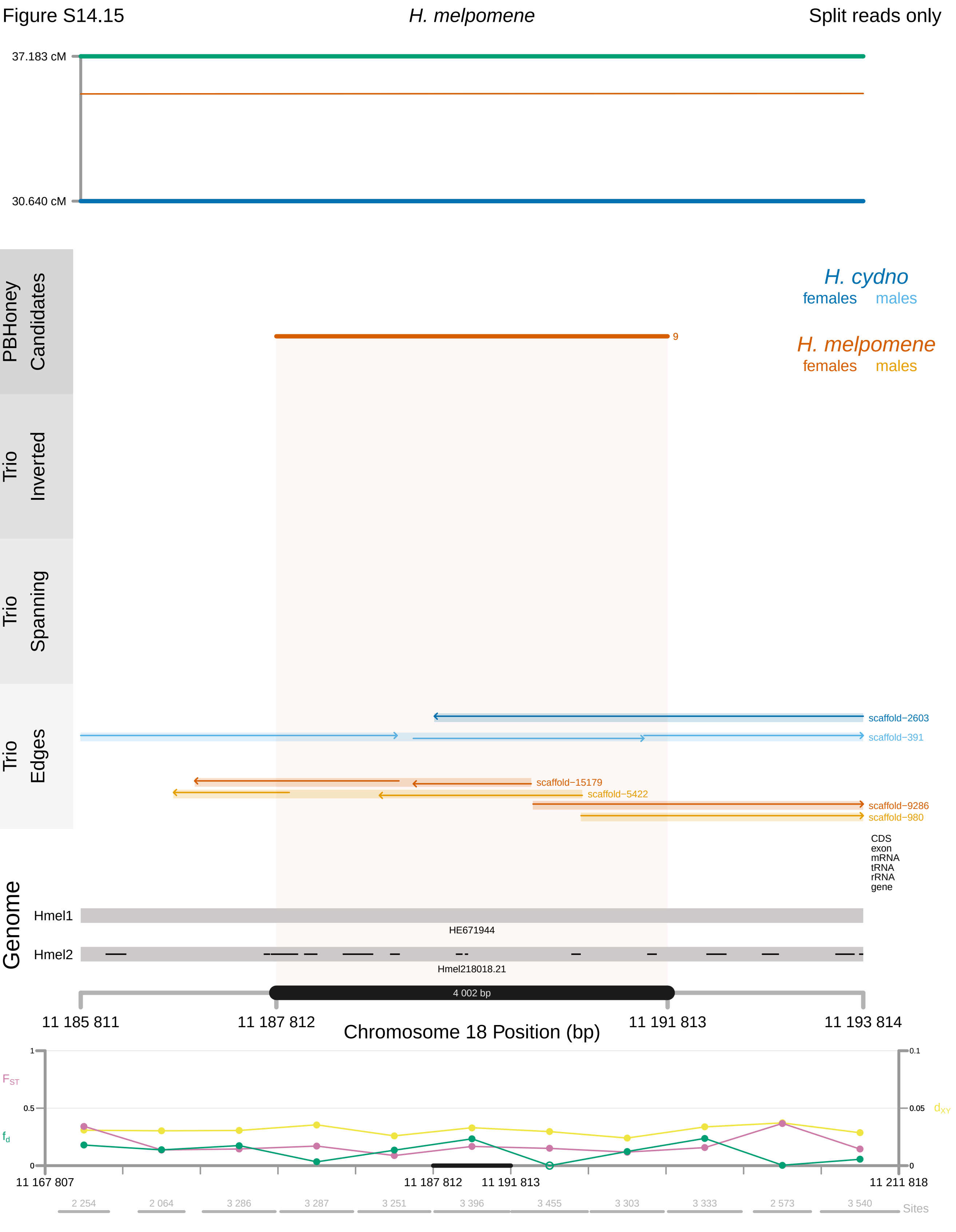

Figure S14.16 *H. melpomene* Split reads only

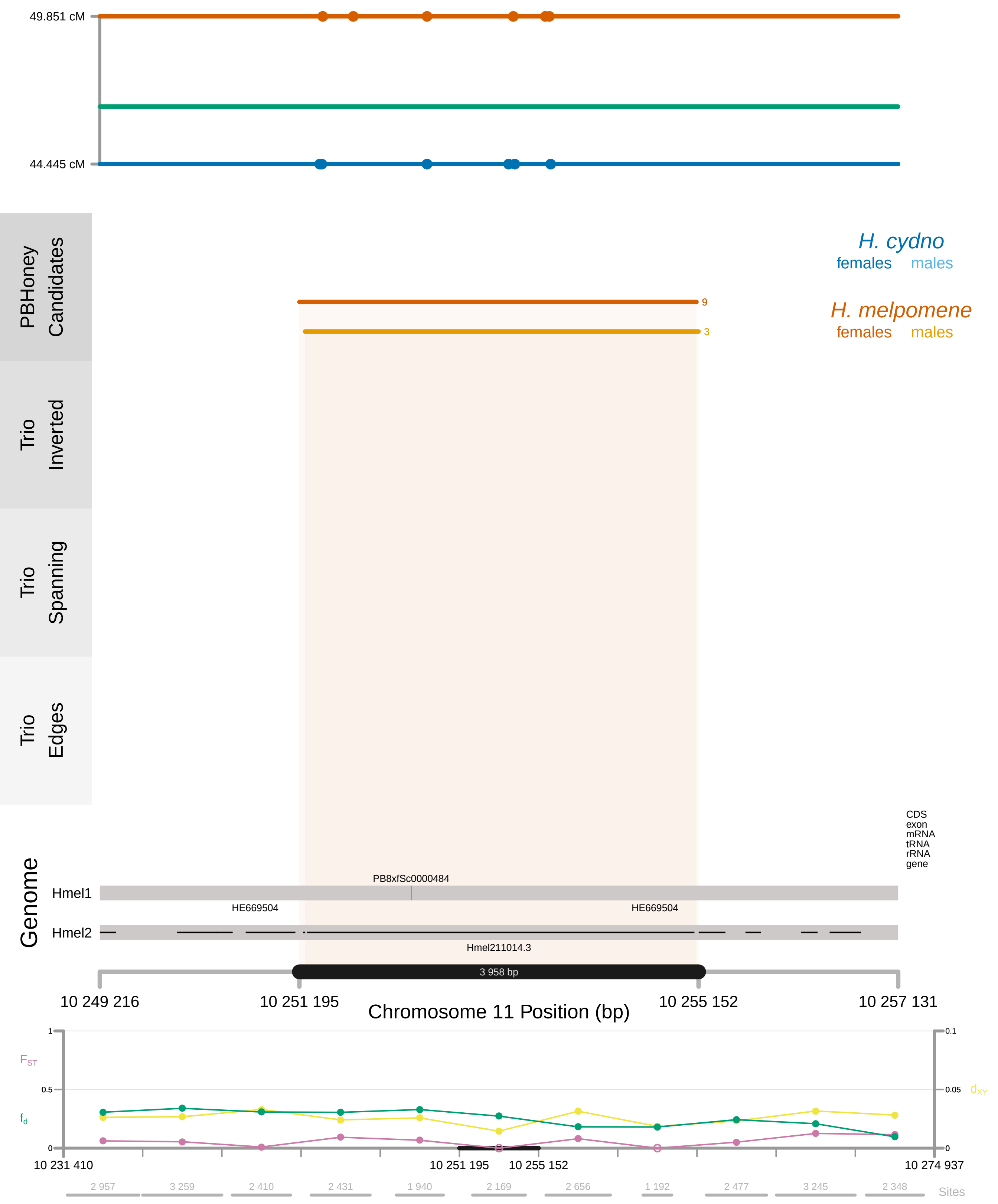

Figure S14.17 *H. melpomene* Split reads only

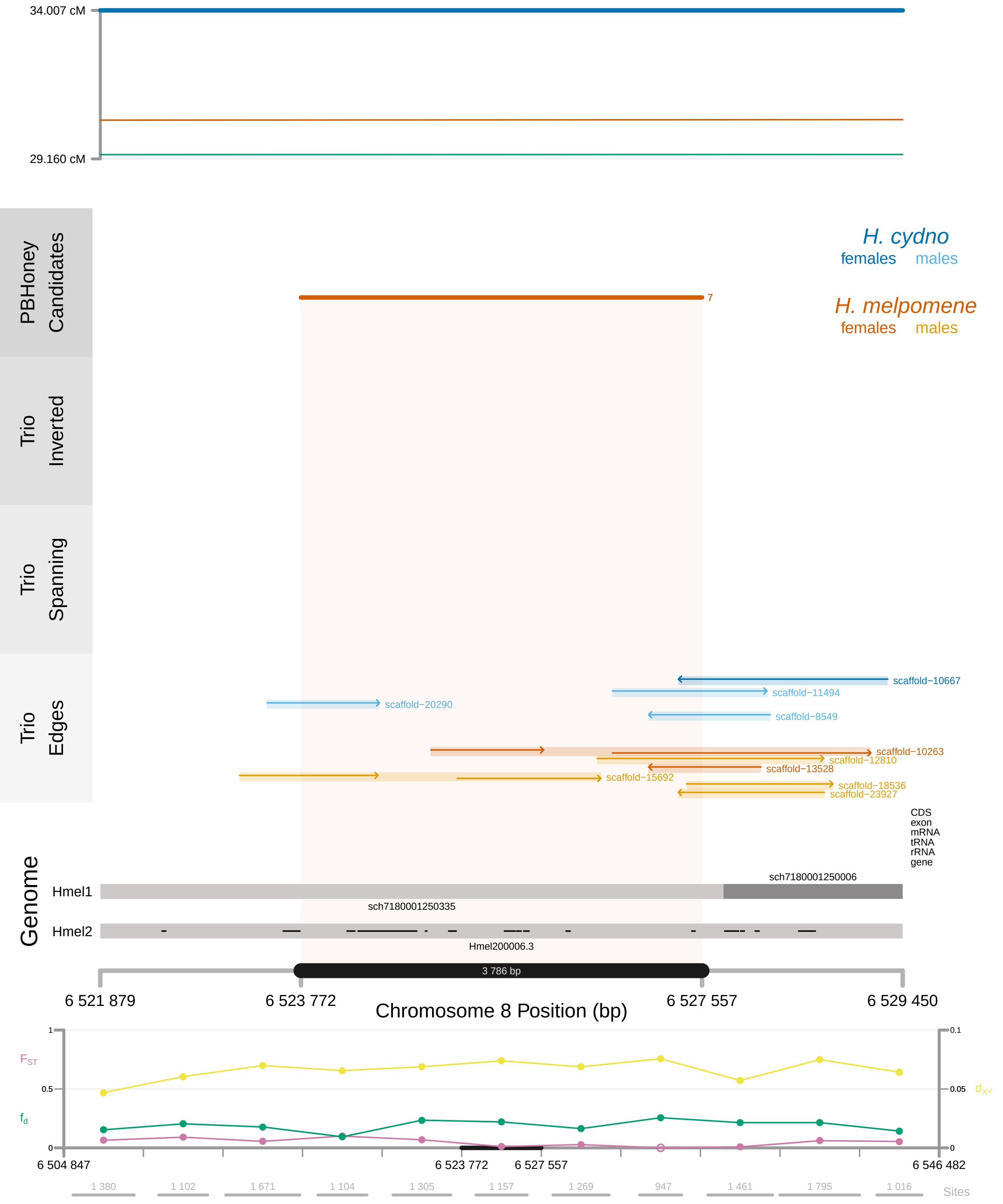

Figure S14.18 *H. melpomene* Split reads only

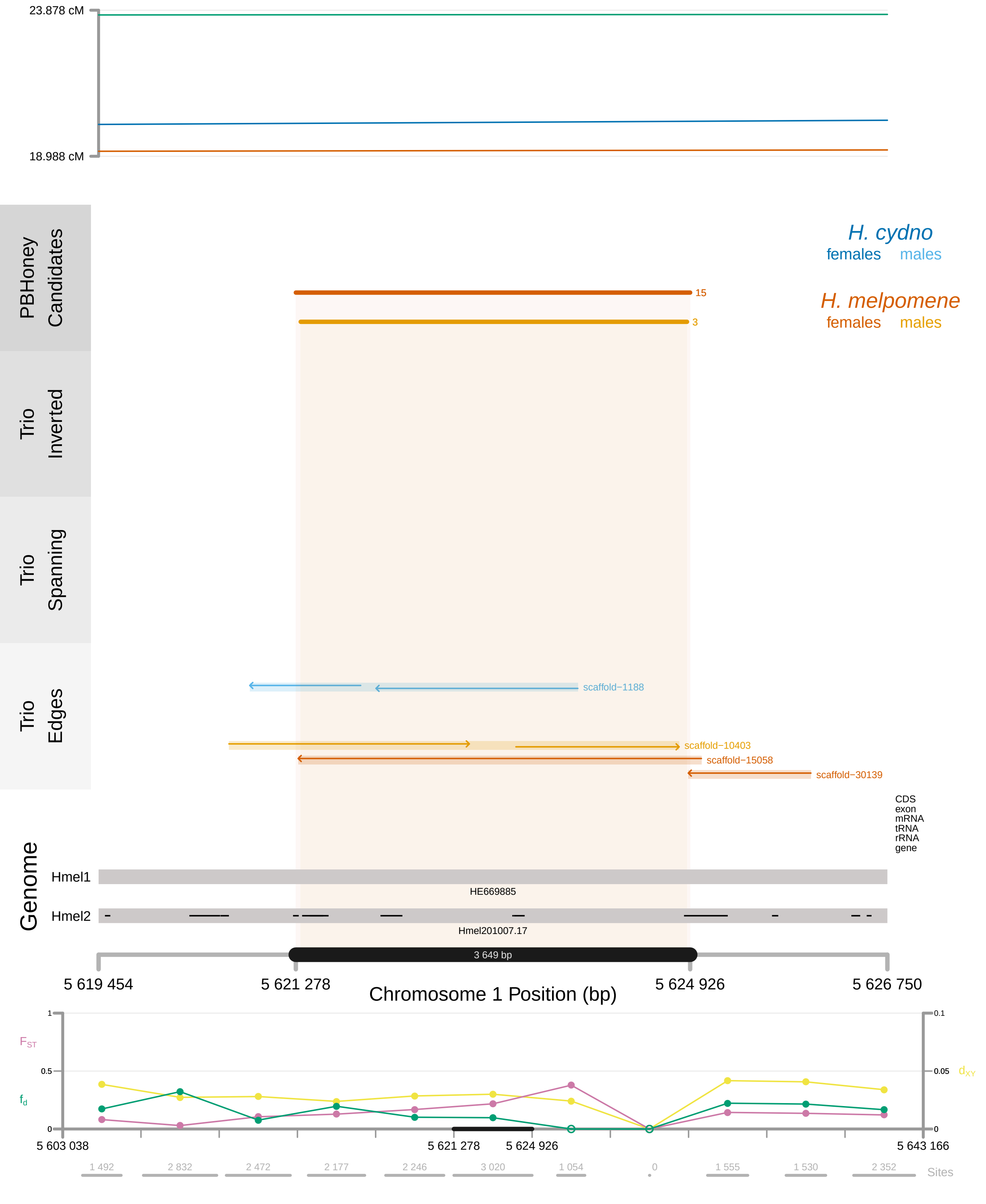

Figure S14.19

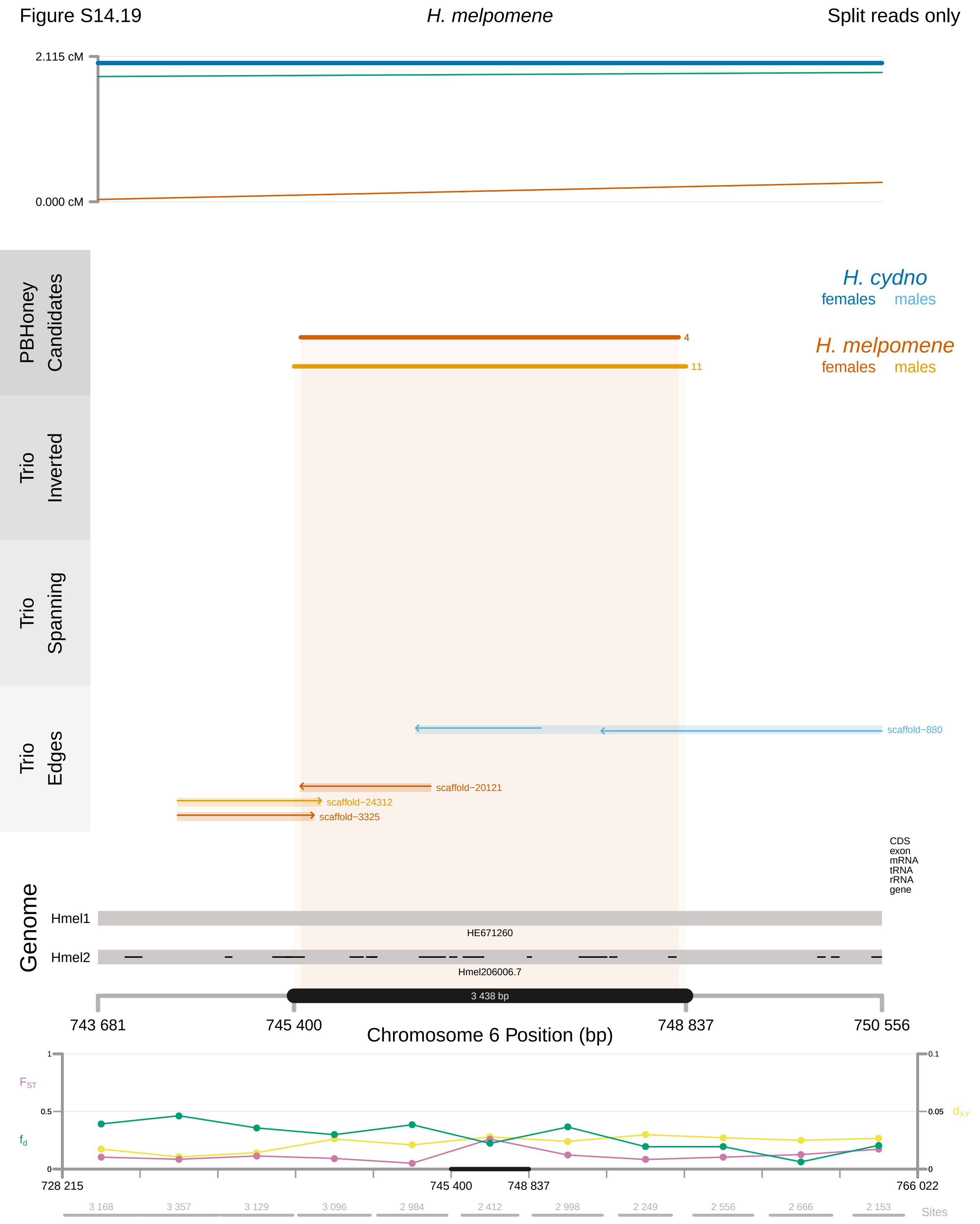

Figure S14.20 *H. melpomene* Split reads only

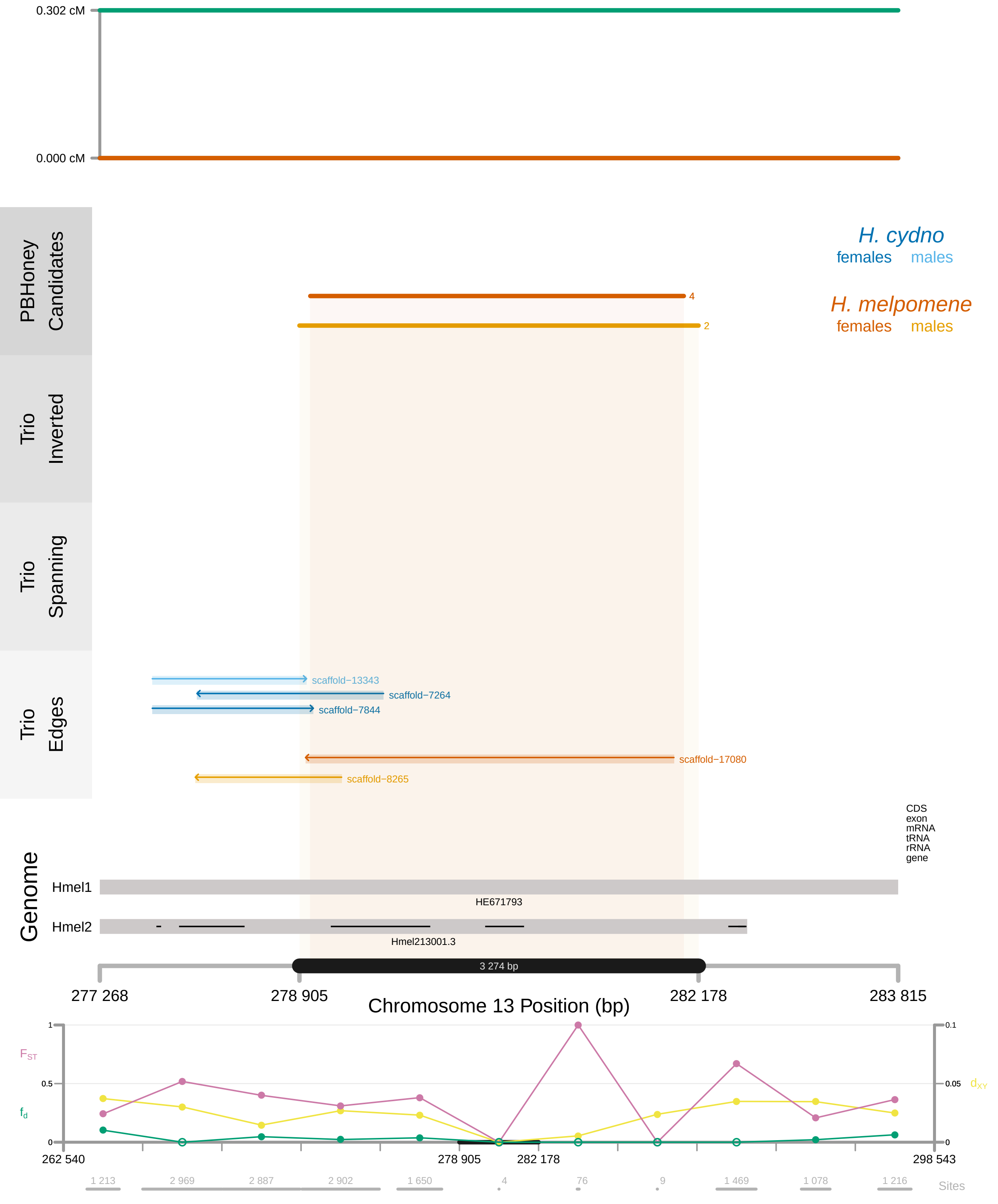

Figure S14.21 *H. melpomene* Split reads only

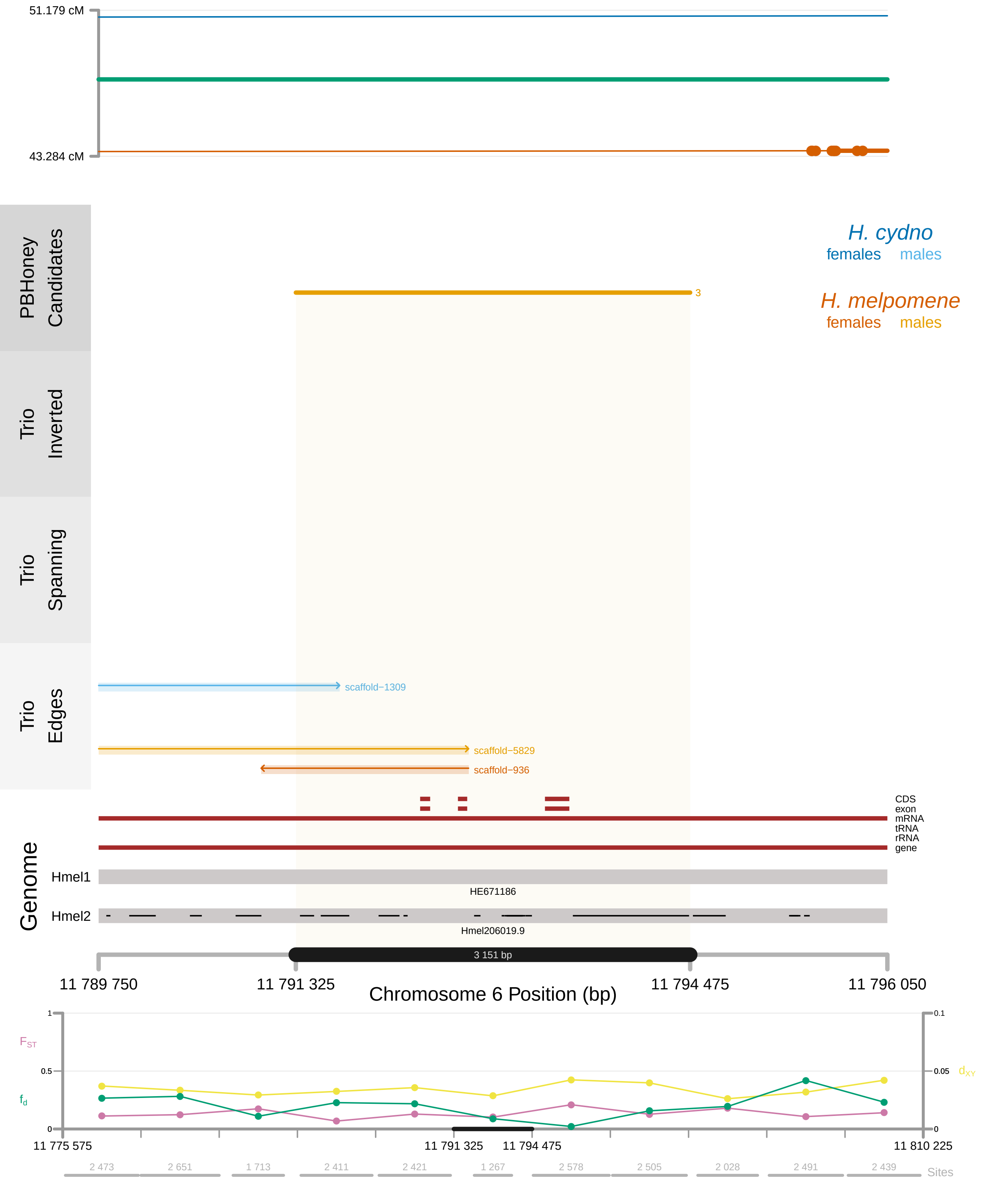

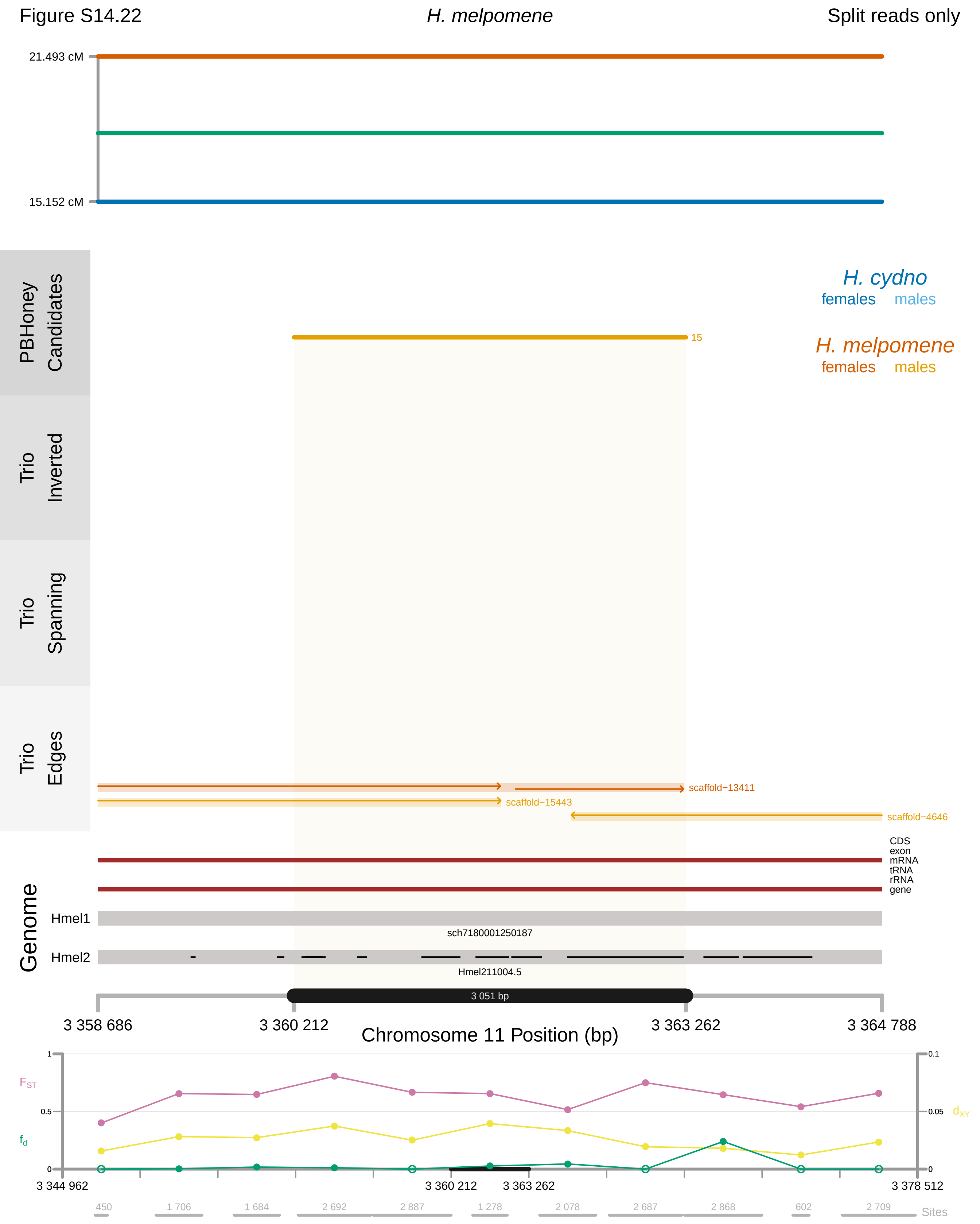

Figure S14.22
H. melpomene
Split reads only
21.493 cM
15.152 cM
PBHoney Candidates
Trio Inverted
Trio Spanning
Trio Edges
Genome
H. cydno
females males
H. melpomene
females males
15
scaffold−13411
scaffold−15443
scaffold−4646
CDS
exon
mRNA
tRNA
rRNA
gene
Hmel1
sch7180001250187
Hmel2
Hmel211004.5
3 051 bp
3 358 686
3 360 212
3 363 262
3 364 788
Chromosome 11 Position (bp)
1
0.5
0
FST
fd
0.1
0.05
0
dXY
3 344 962
3 360 212
3 363 262
3 378 512
450
1 706
1 684
2 692
2 887
1 278
2 078
2 687
2 868
602
2 709
Sites

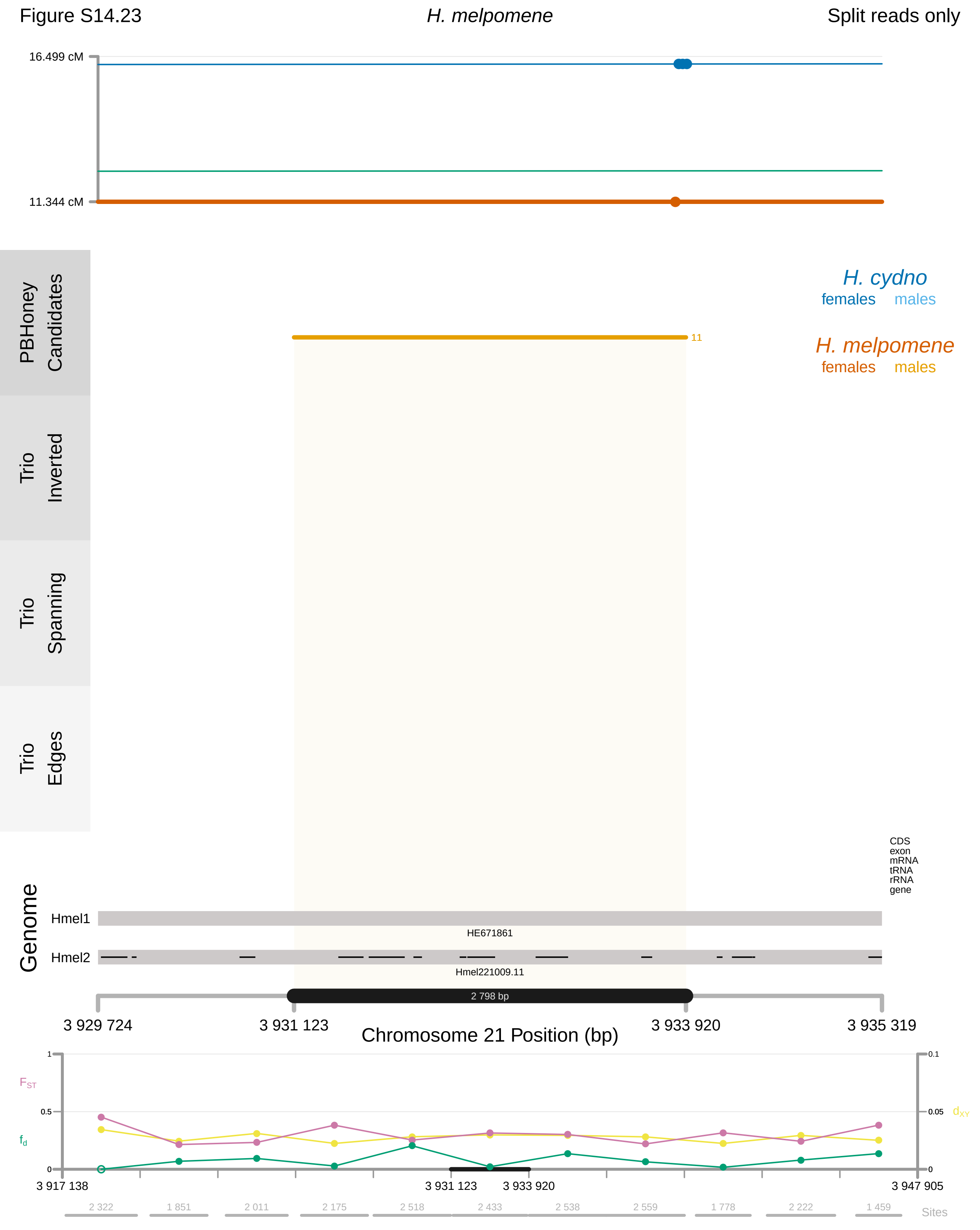

Figure S14.23
H. melpomene
Split reads only
16.499 cM
11.344 cM
PBHoney Candidates
Trio Inverted
Trio Spanning
Trio Edges
11
H. cydno
females males
H. melpomene
females males
CDS
exon
mRNA
tRNA
rRNA
gene
Genome
Hmel1
HE671861
Hmel2
Hmel221009.11
2 798 bp
3 929 724
3 931 123
3 933 920
3 935 319
Chromosome 21 Position (bp)
1
0.5
0
FST
fd
0.1
0.05
0
dXY
3 917 138
3 931 123
3 933 920
3 947 905
2 322
1 851
2 011
2 175
2 518
2 433
2 538
2 559
1 778
2 222
1 459
Sites

Figure S14.24 *H. melpomene* Split reads only

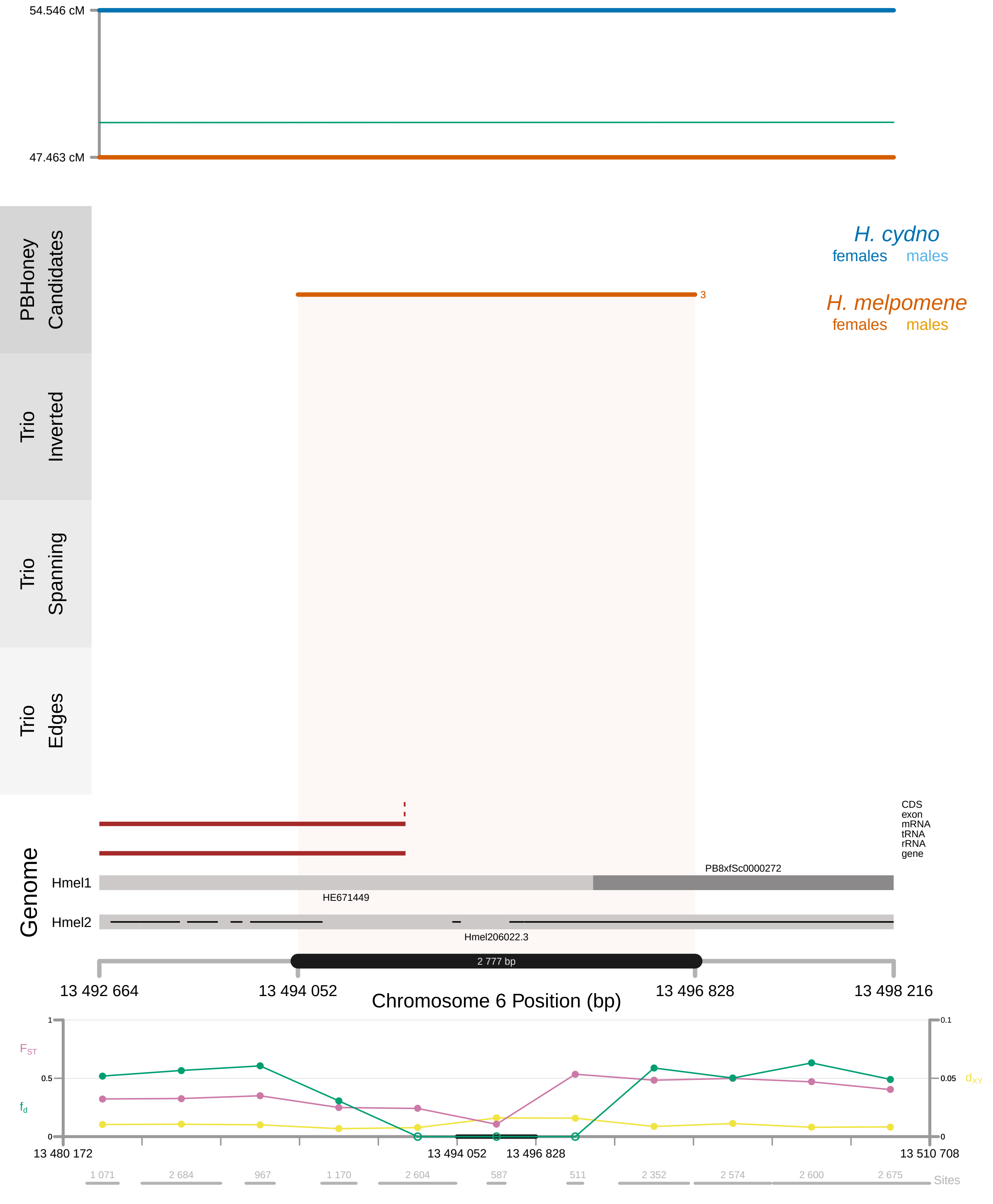

Figure S14.25 *H. melpomene* Split reads only

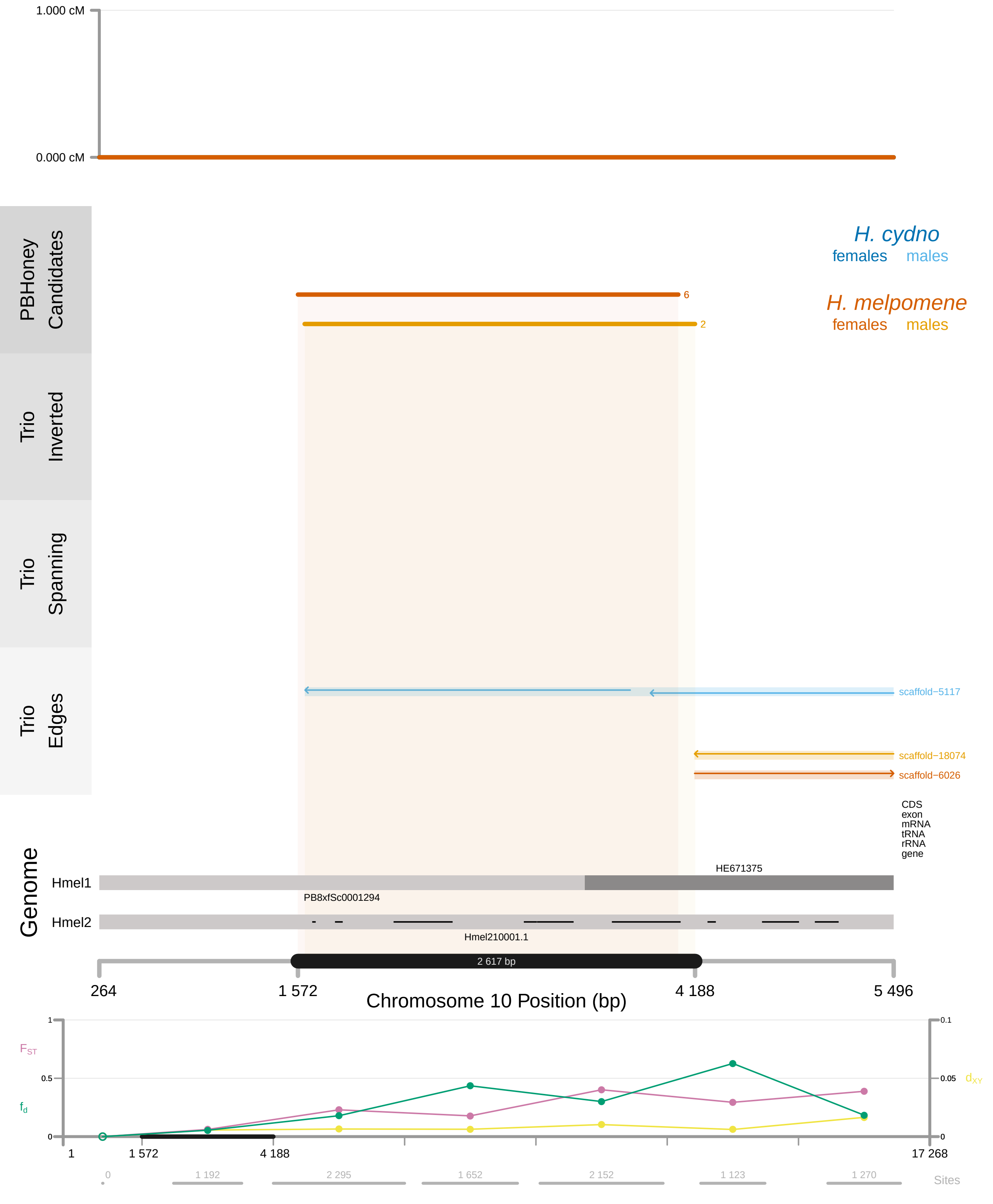

Figure S14.26 *H. melpomene* Split reads only

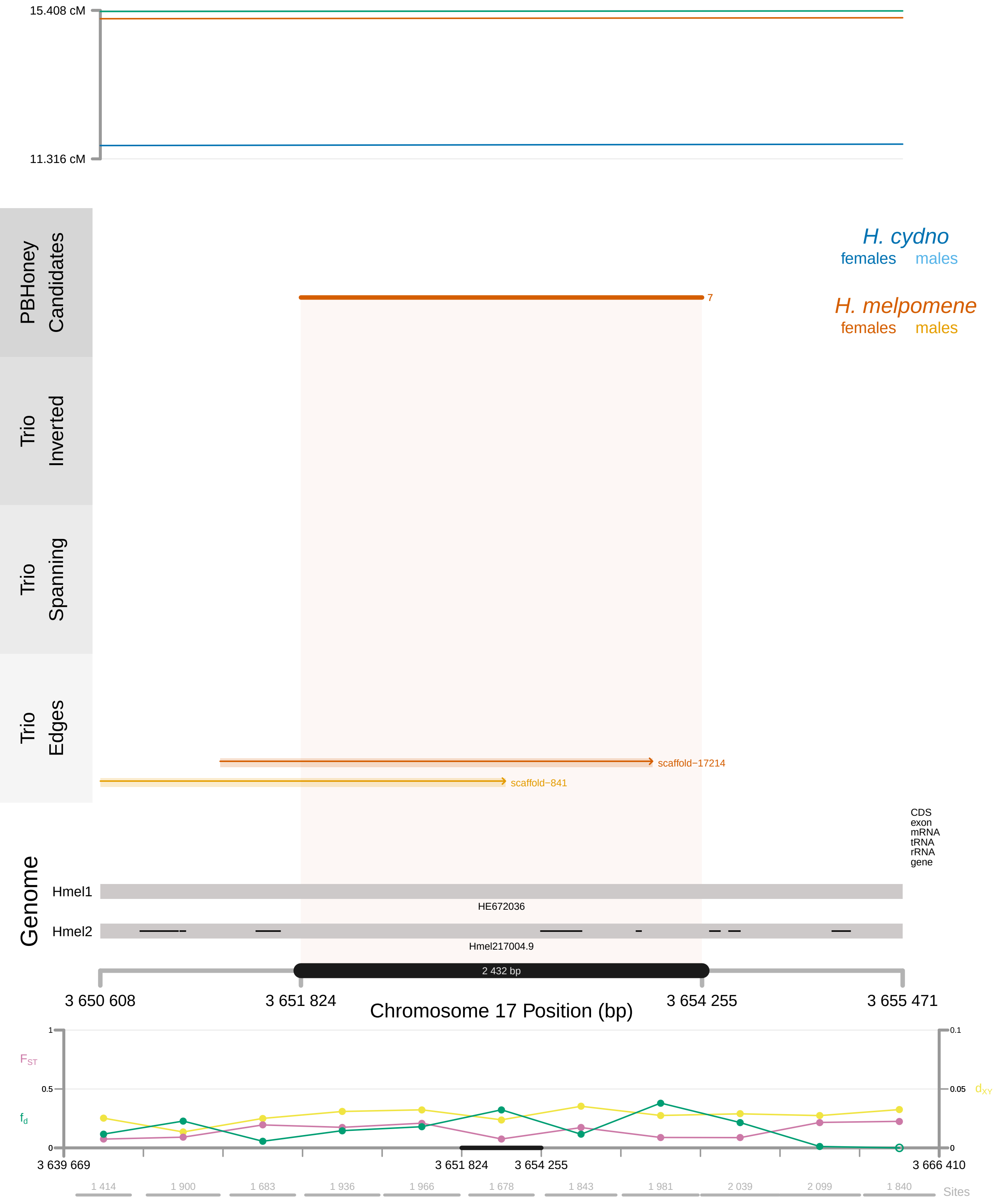

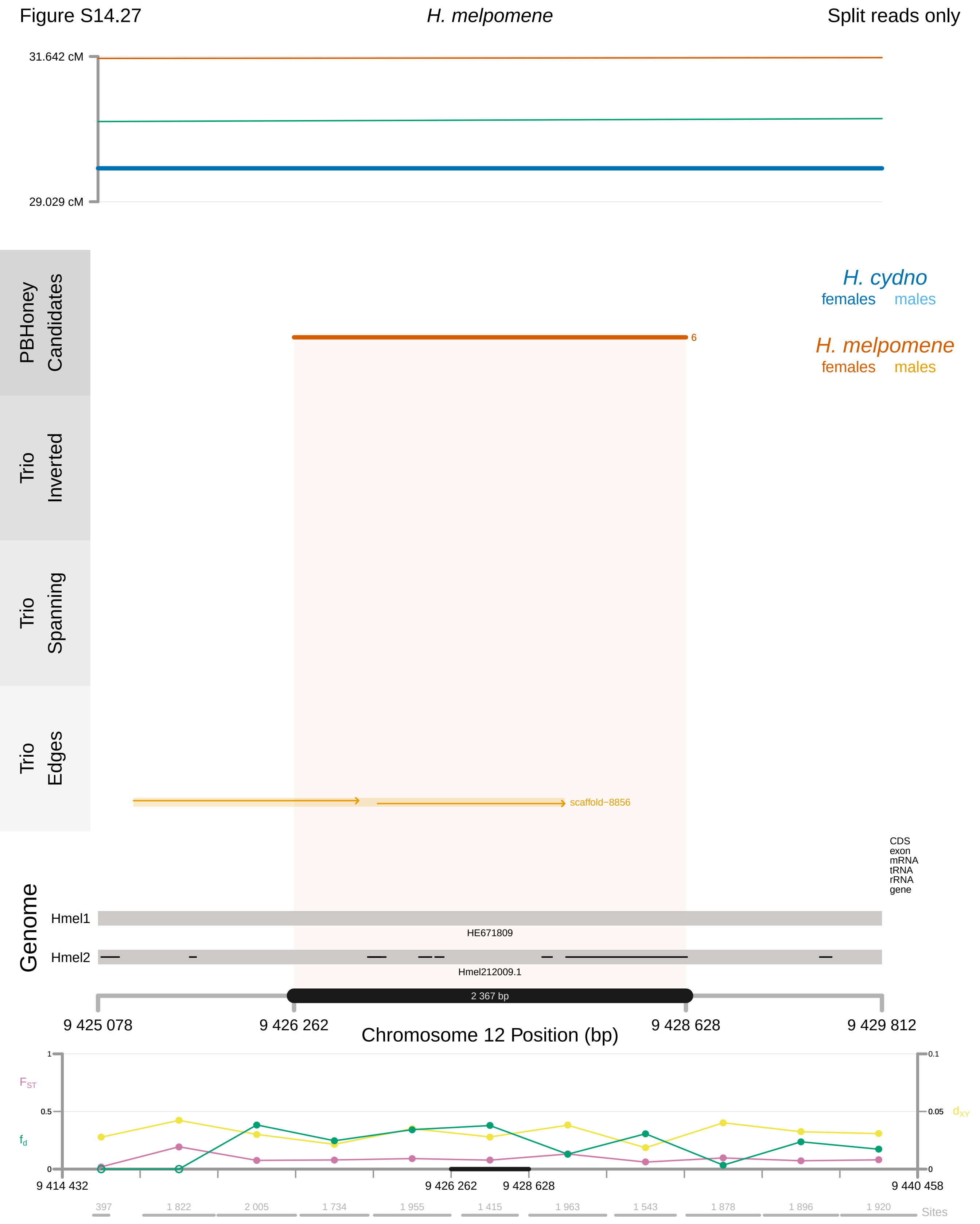

Figure S14.27
H. melpomene
Split reads only
31.642 cM
29.029 cM
PBHoney Candidates
Trio Inverted
Trio Spanning
Trio Edges
H. cydno
females
males
H. melpomene
females
males
6
scaffold−8856
CDS
exon
mRNA
tRNA
rRNA
gene
Genome
Hmel1
HE671809
Hmel2
Hmel212009.1
2 367 bp
9 425 078
9 426 262
9 428 628
9 429 812
Chromosome 12 Position (bp)
1
0.5
0
F_ST
f_d
0.1
0.05
0
d_XY
9 414 432
9 426 262
9 428 628
9 440 458
397
1 822
2 005
1 734
1 955
1 415
1 963
1 543
1 878
1 896
1 920
Sites

Figure S14.28

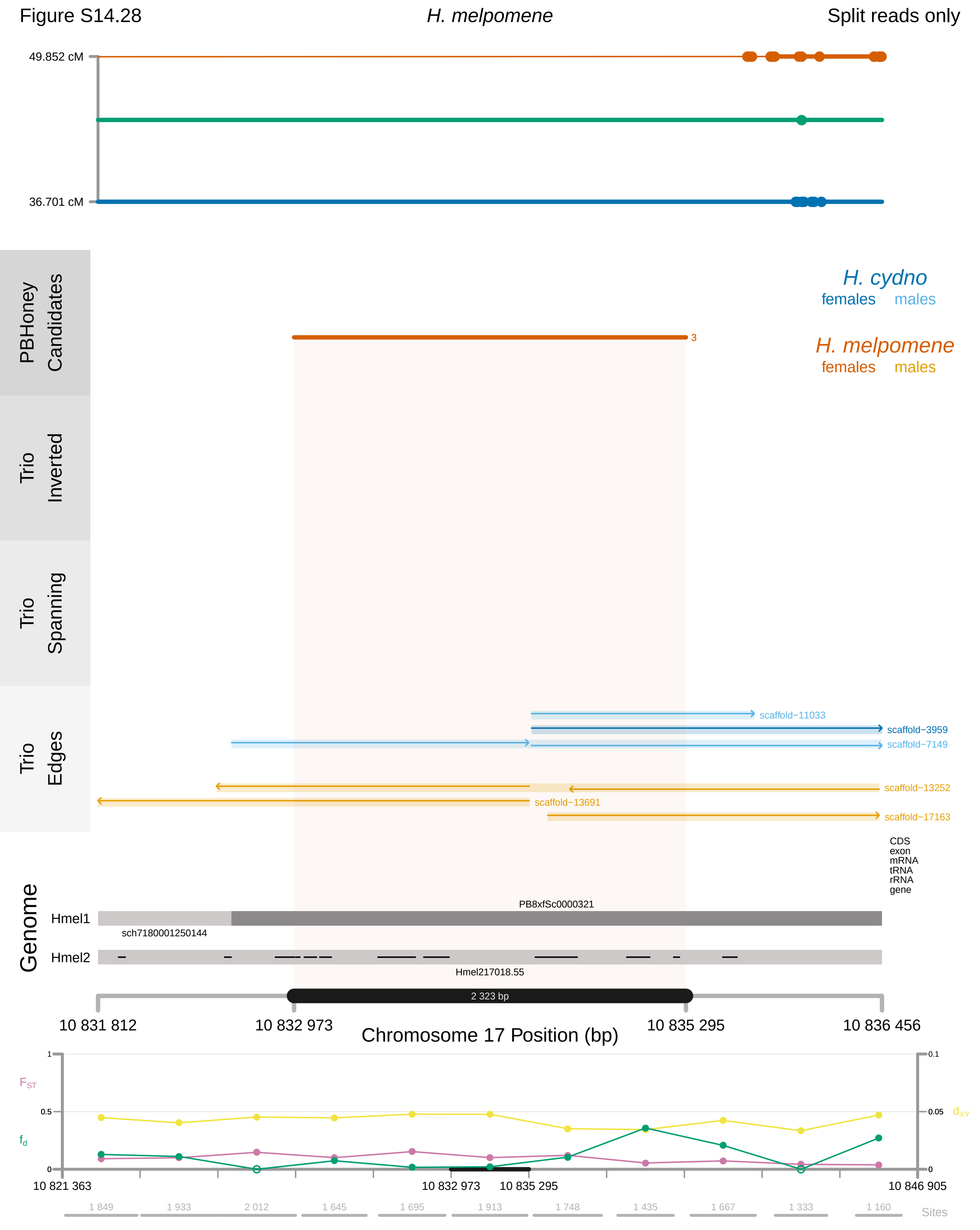

Figure S14.29

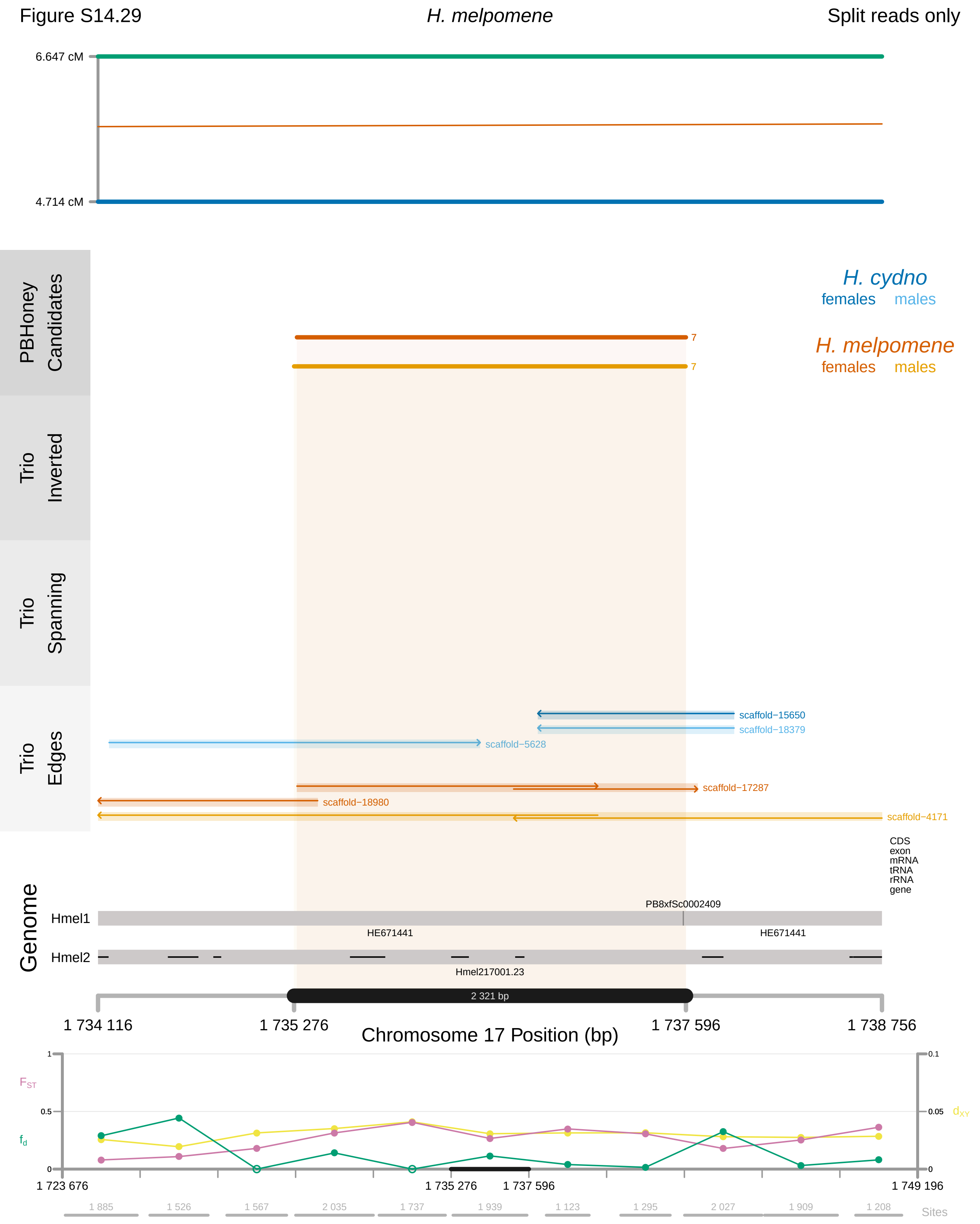

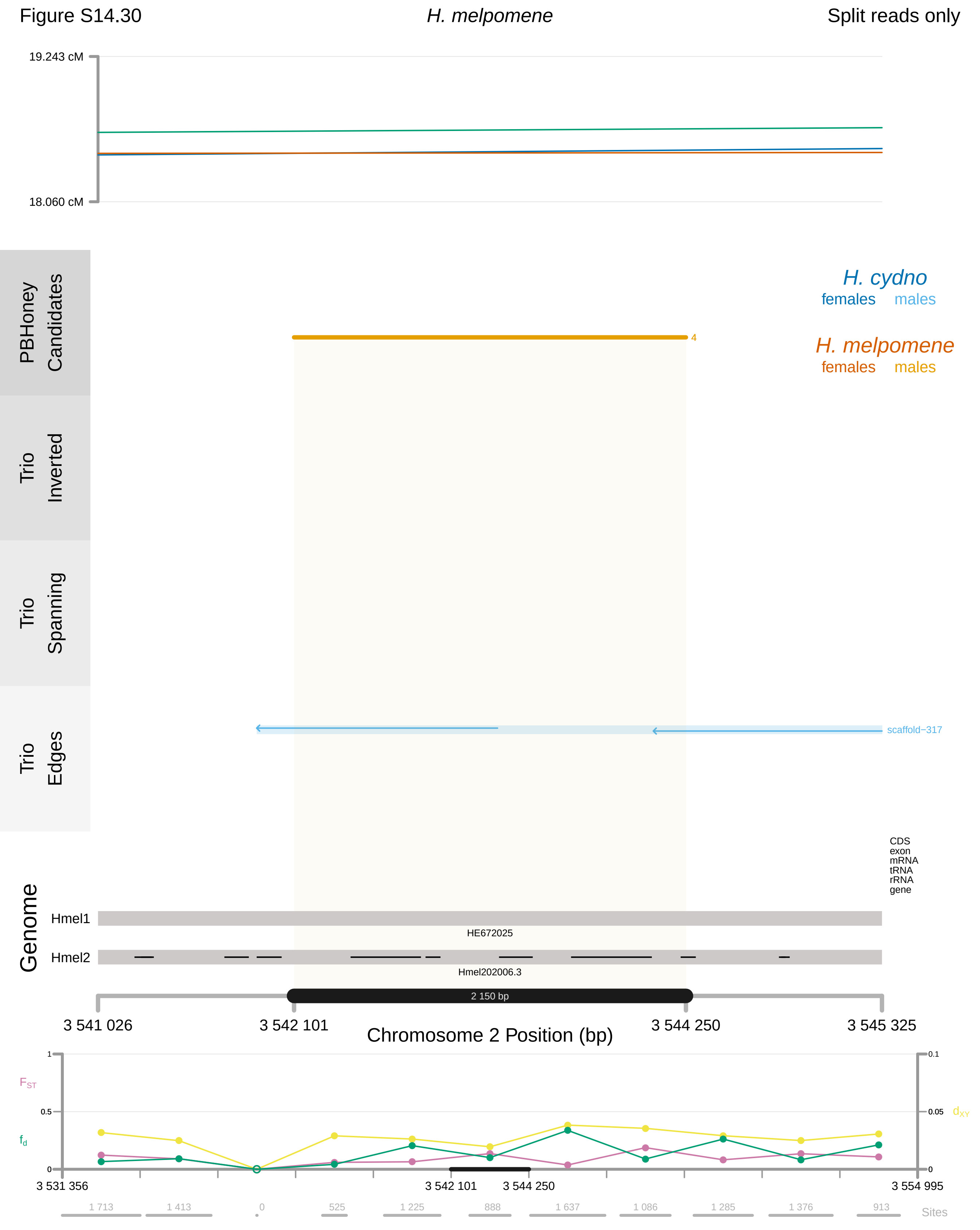

Figure S14.30
H. melpomene
Split reads only
19.243 cM
18.060 cM
PBHoney Candidates
Trio Inverted
Trio Spanning
Trio Edges
Genome
H. cydno
females males
H. melpomene
females males
4
scaffold−317
CDS
exon
mRNA
tRNA
rRNA
gene
Hmel1
HE672025
Hmel2
Hmel202006.3
2 150 bp
3 541 026
3 542 101
3 544 250
3 545 325
Chromosome 2 Position (bp)
F_ST
f_d
d_XY
1
0.5
0
0.1
0.05
0
3 531 356
3 542 101
3 544 250
3 554 995
1 713
1 413
0
525
1 225
888
1 637
1 086
1 285
1 376
913
Sites

Figure S14.31 *H. melpomene* Split reads only

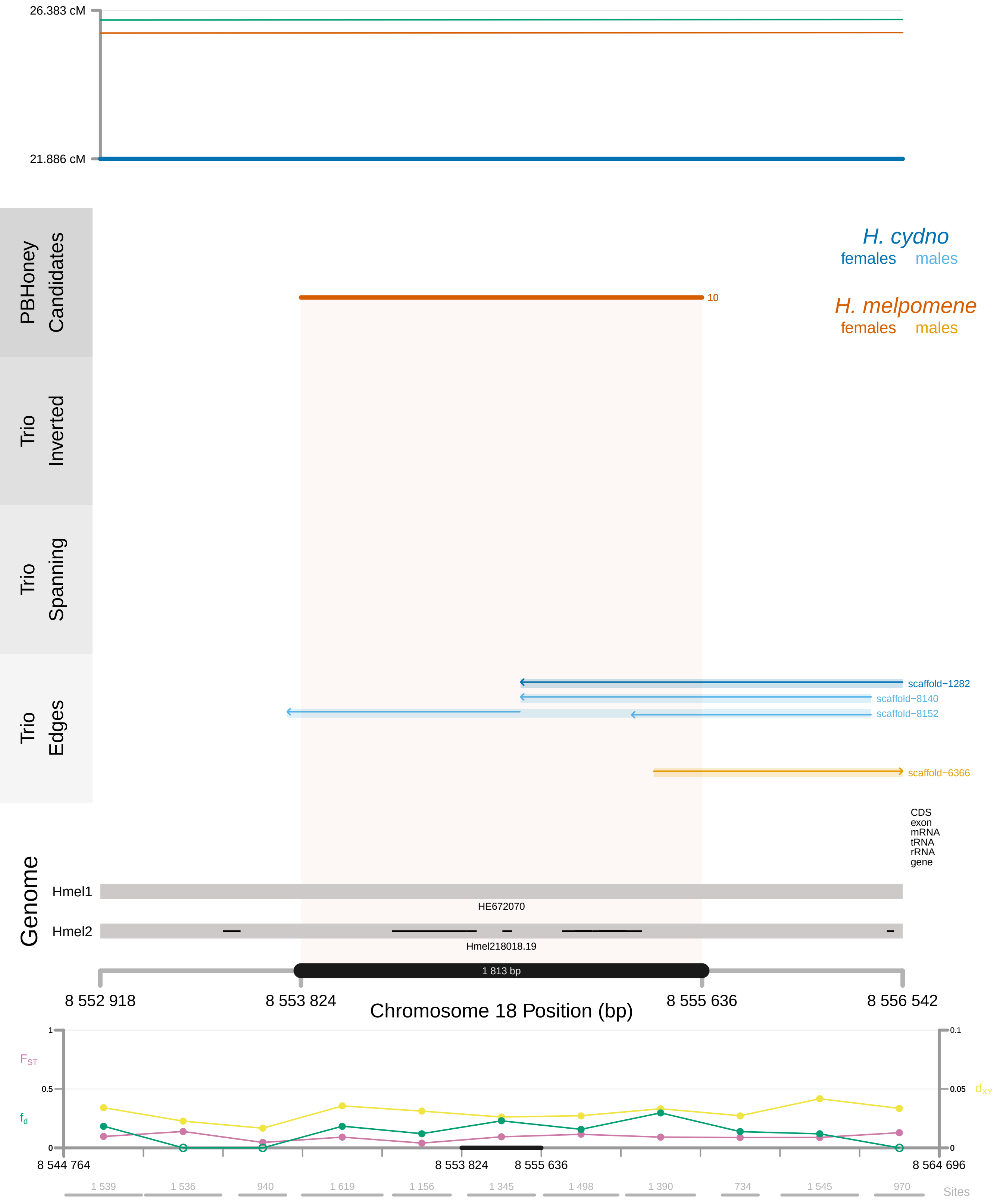

Figure S14.32

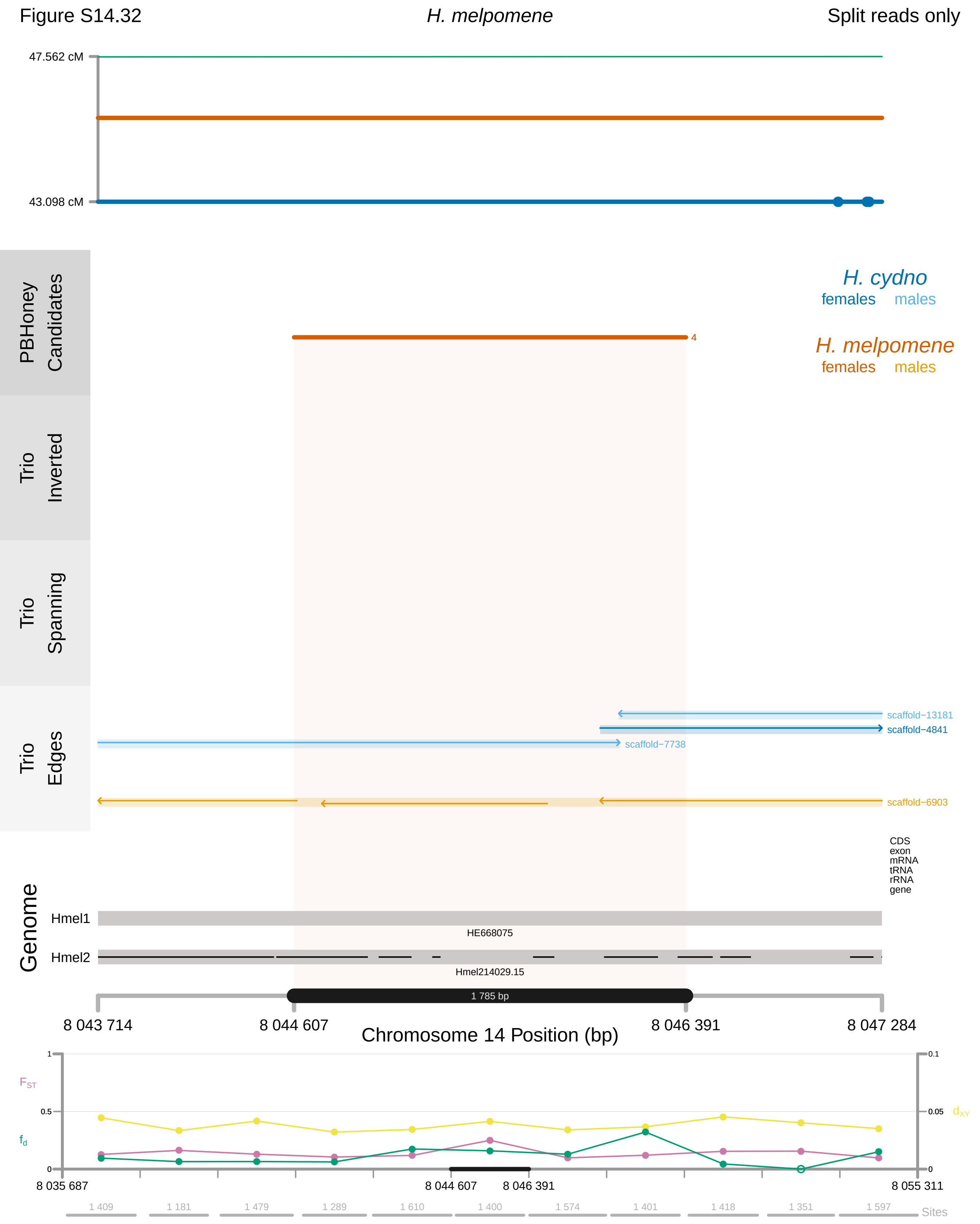

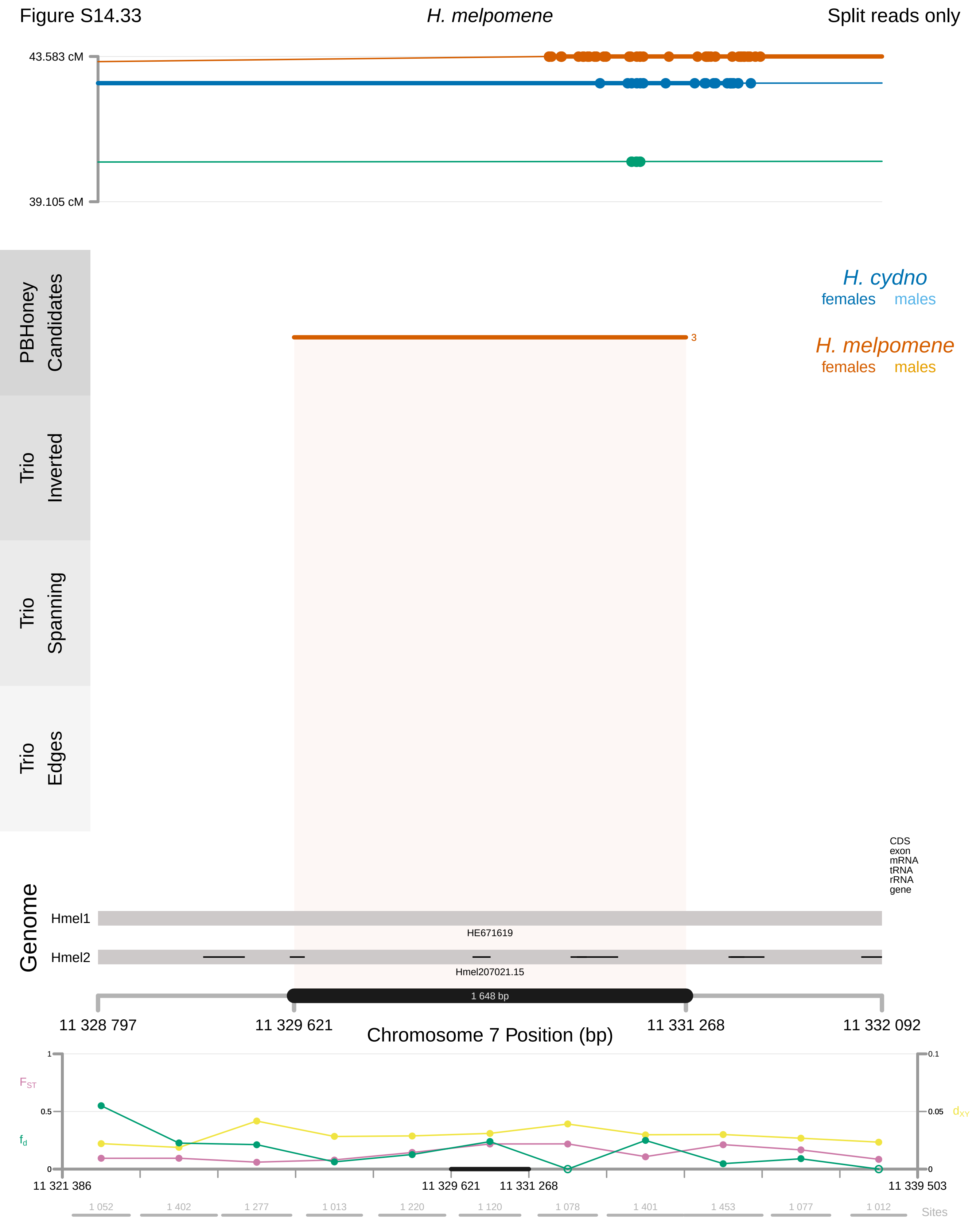

Figure S14.33
H. melpomene
Split reads only
43.583 cM
39.105 cM
PBHoney Candidates
Trio Inverted
Trio Spanning
Trio Edges
3
H. cydno
females
males
H. melpomene
females
males
CDS
exon
mRNA
tRNA
rRNA
gene
Genome
Hmel1
HE671619
Hmel2
Hmel207021.15
1 648 bp
11 328 797
11 329 621
11 331 268
11 332 092
Chromosome 7 Position (bp)
FST
fd
dXY
1
0.5
0
0.1
0.05
0
11 321 386
11 329 621
11 331 268
11 339 503
1 052
1 402
1 277
1 013
1 220
1 120
1 078
1 401
1 453
1 077
1 012
Sites

Figure S14.34 *H. melpomene* Split reads only

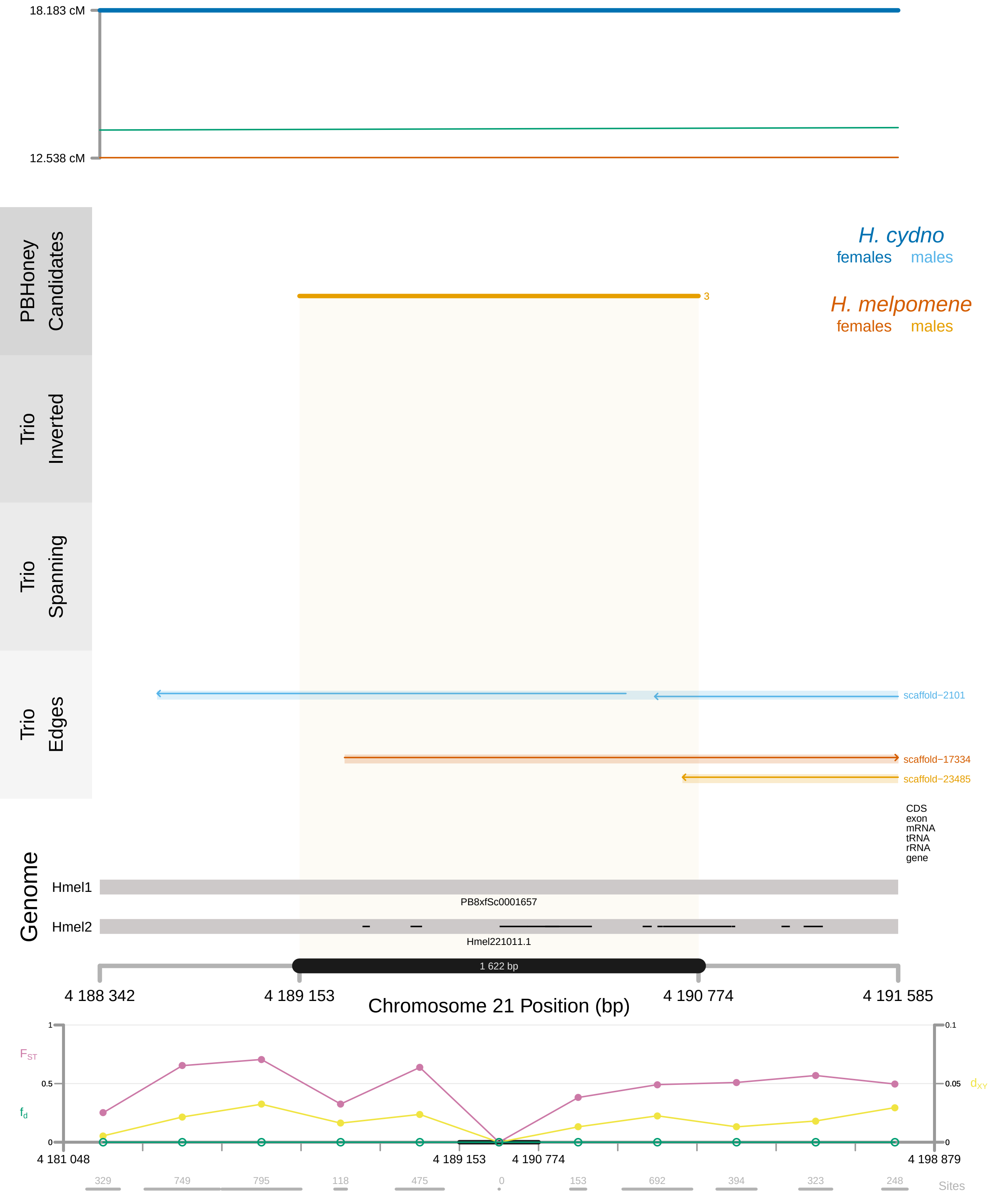

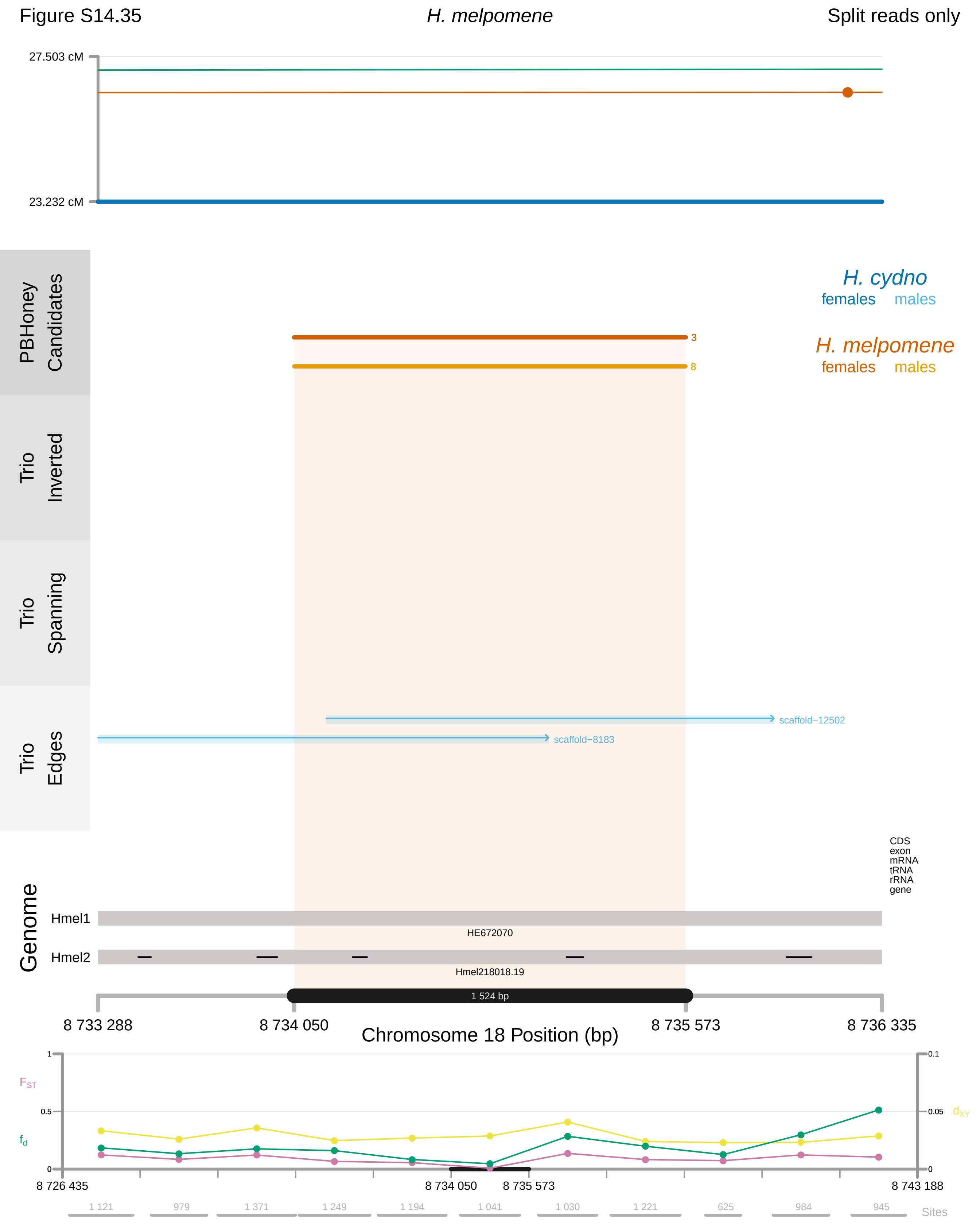

Figure S14.35
H. melpomene
Split reads only
27.503 cM
23.232 cM
PBHoney Candidates
Trio Inverted
Trio Spanning
Trio Edges
H. cydno
females
males
H. melpomene
females
males
3
8
scaffold−12502
scaffold−8183
CDS
exon
mRNA
tRNA
rRNA
gene
Genome
Hmel1
HE672070
Hmel2
Hmel218018.19
1 524 bp
8 733 288
8 734 050
8 735 573
8 736 335
Chromosome 18 Position (bp)
1
0.5
0
F_{ST}
f_d
0.1
0.05
0
d_{XY}
8 726 435
8 734 050
8 735 573
8 743 188
1 121
979
1 371
1 249
1 194
1 041
1 030
1 221
625
984
945
Sites

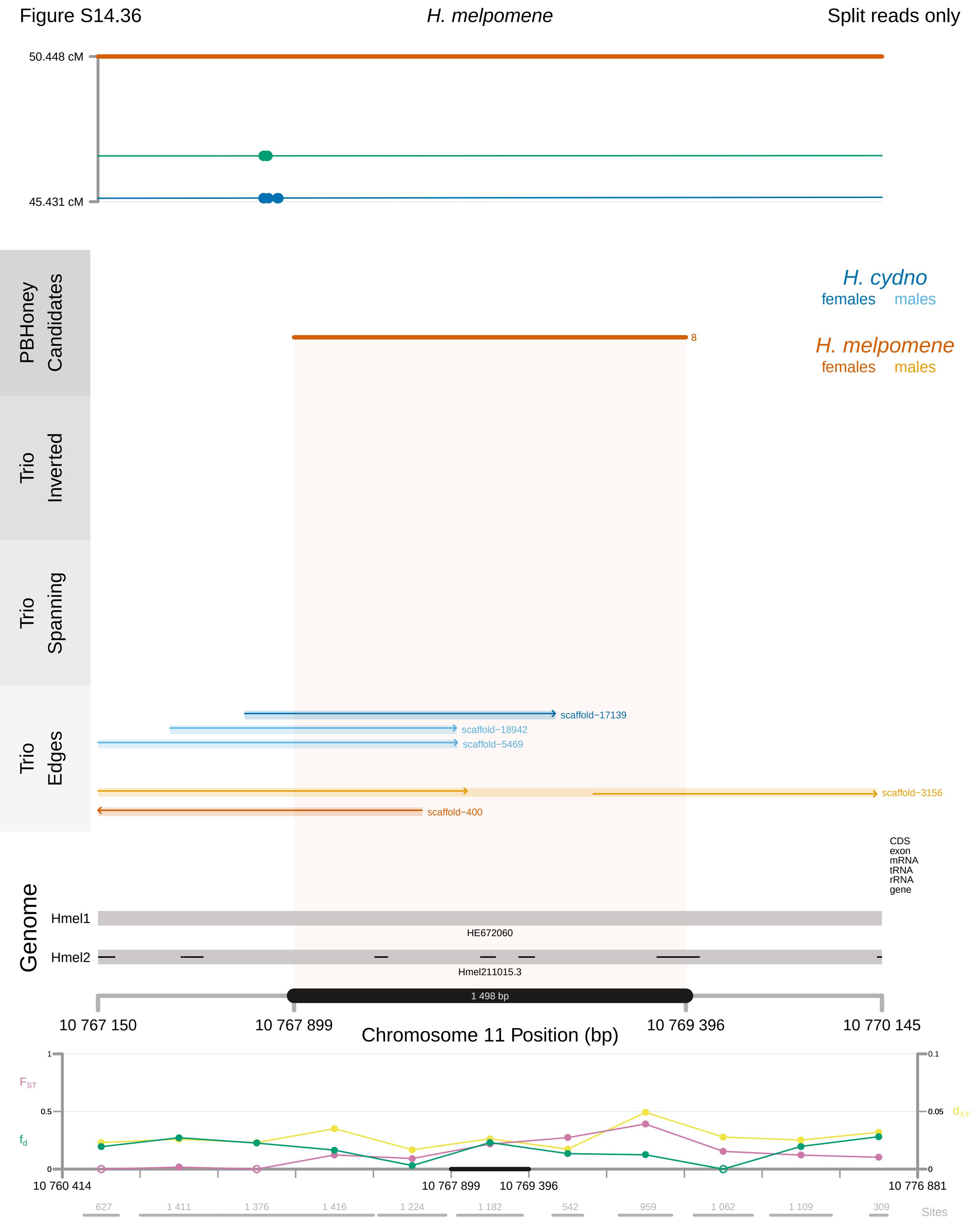

Figure S14.36
H. melpomene
Split reads only
50.448 cM
45.431 cM
PBHoney Candidates
Trio Inverted
Trio Spanning
Trio Edges
Genome
H. cydno
females
males
H. melpomene
females
males
8
scaffold−17139
scaffold−18942
scaffold−5469
scaffold−3156
scaffold−400
CDS
exon
mRNA
tRNA
rRNA
gene
Hmel1
HE672060
Hmel2
Hmel211015.3
1 498 bp
10 767 150
10 767 899
10 769 396
10 770 145
Chromosome 11 Position (bp)
F_ST
f_d
d_XY
1
0.5
0
0.1
0.05
0
10 760 414
10 767 899
10 769 396
10 776 881
627
1 411
1 376
1 416
1 224
1 182
542
959
1 062
1 109
309
Sites

Figure S14.37

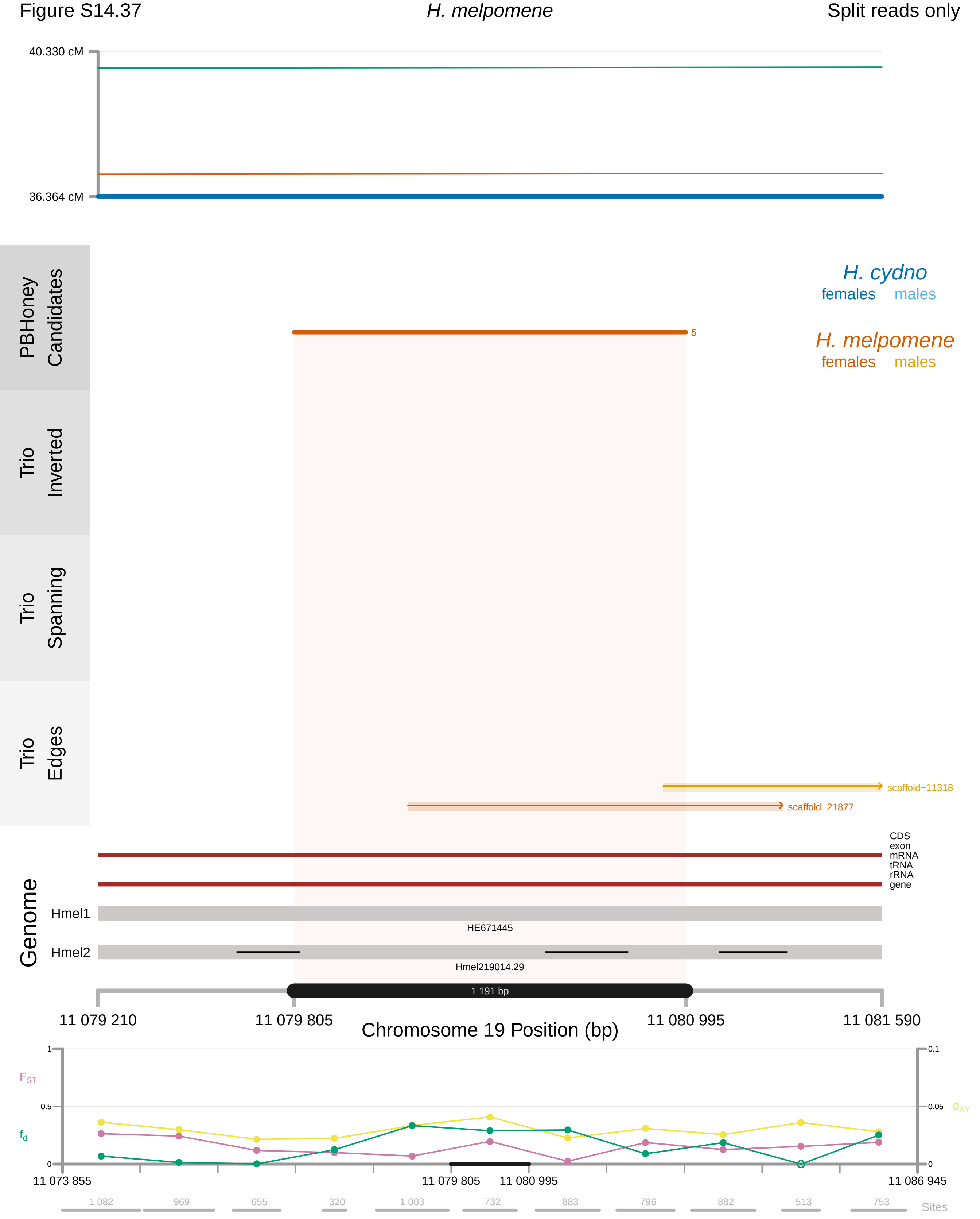

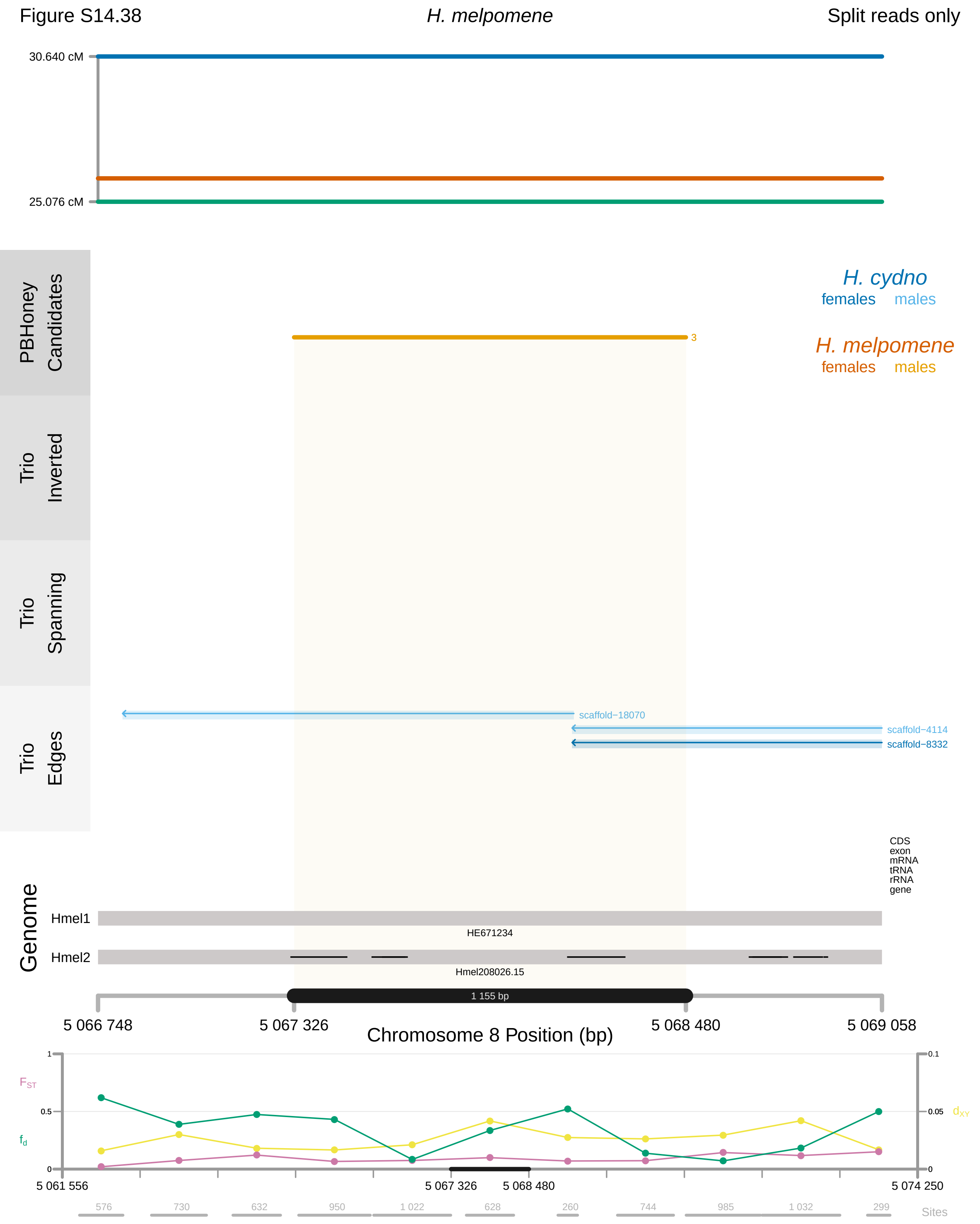

Figure S14.38
H. melpomene
Split reads only
30.640 cM
25.076 cM
PBHoney Candidates
Trio Inverted
Trio Spanning
Trio Edges
Genome
H. cydno
females males
H. melpomene
females males
3
scaffold−18070
scaffold−4114
scaffold−8332
CDS
exon
mRNA
tRNA
rRNA
gene
Hmel1
HE671234
Hmel2
Hmel208026.15
1 155 bp
5 066 748
5 067 326
5 068 480
5 069 058
Chromosome 8 Position (bp)
FST
fd
dXY
1
0.5
0
0.1
0.05
0
5 061 556
5 067 326
5 068 480
5 074 250
576
730
632
950
1 022
628
260
744
985
1 032
299
Sites

Figure S14.39 *H. melpomene* Split reads only

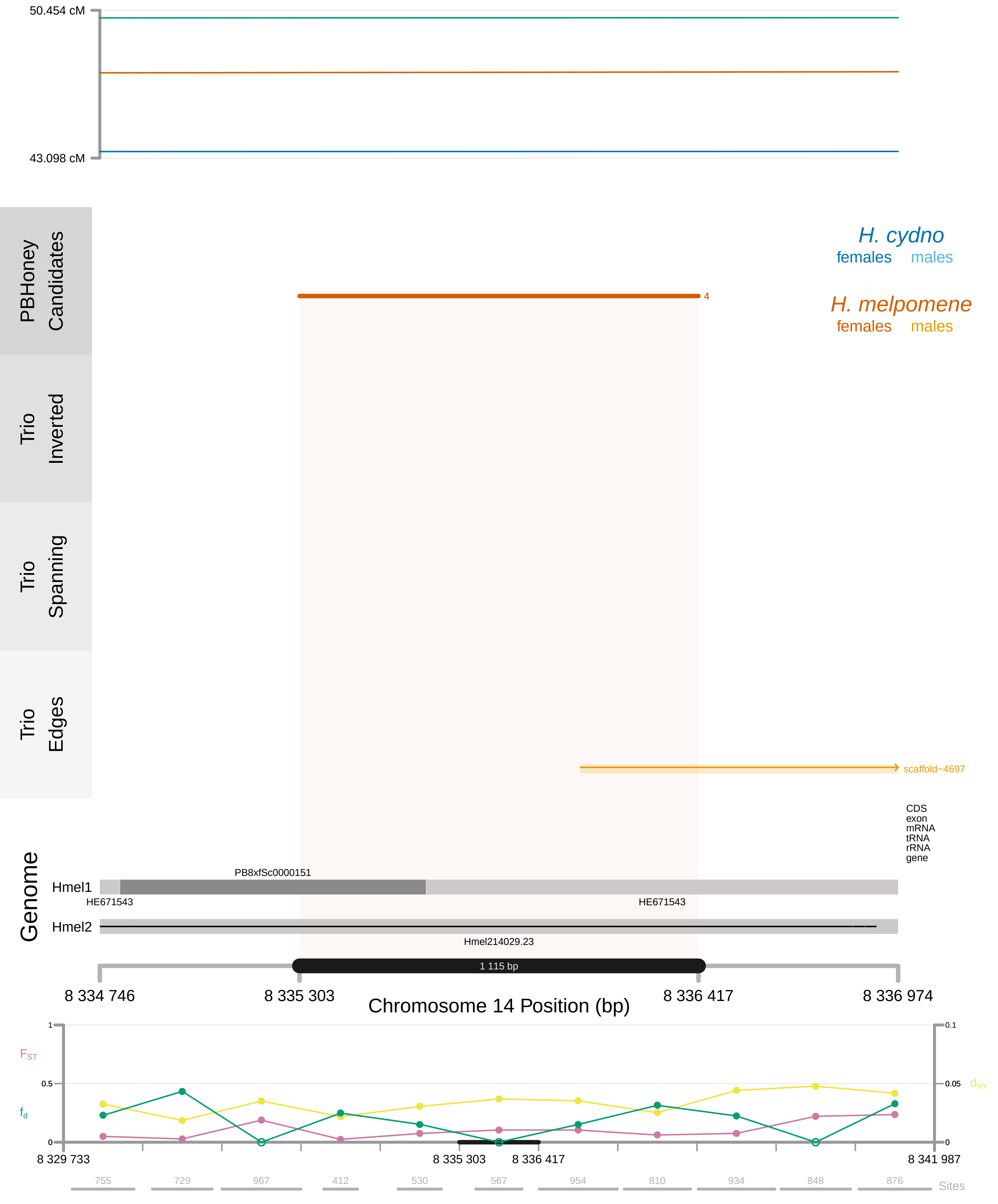

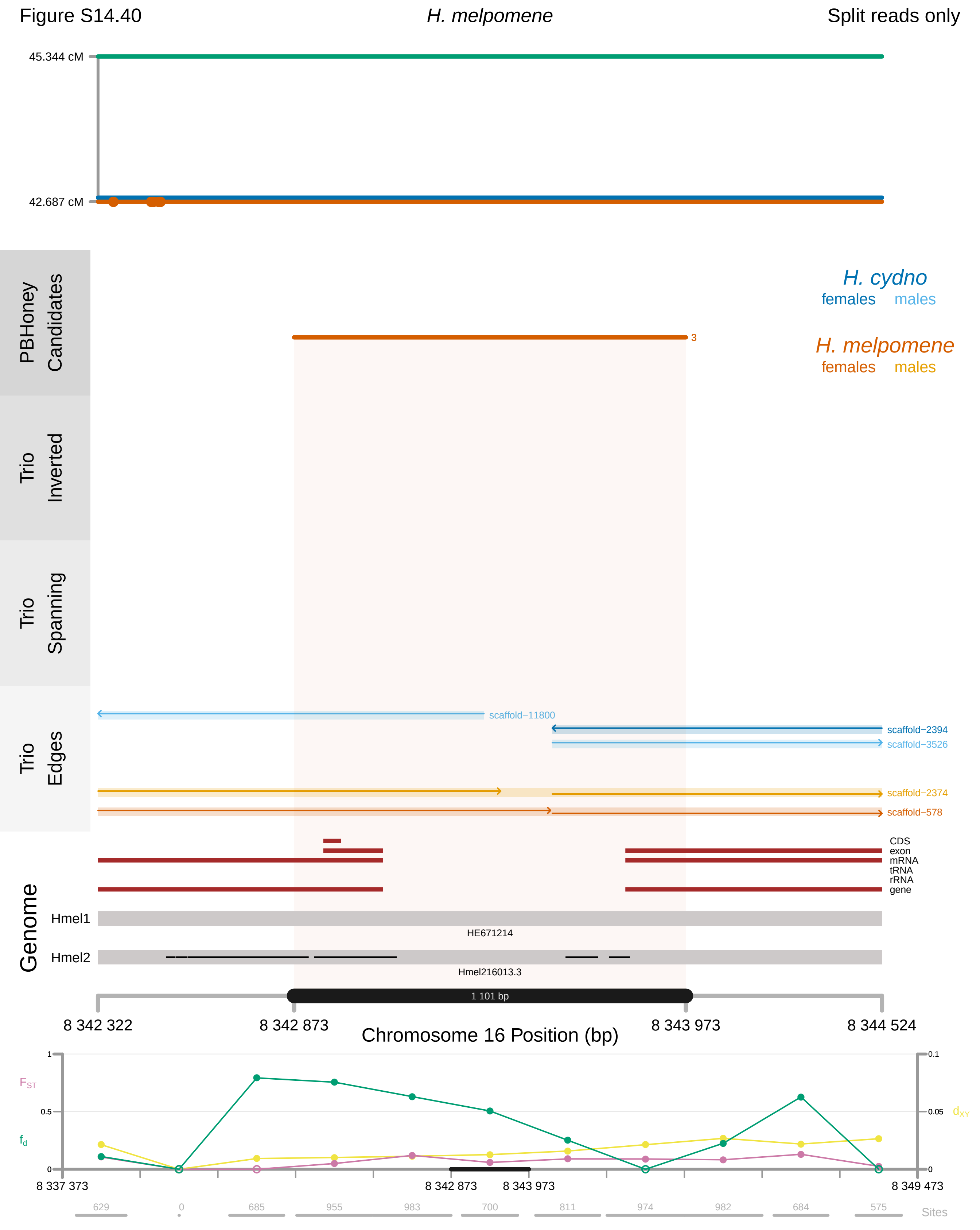

Figure S14.40
H. melpomene
Split reads only
45.344 cM
42.687 cM
PBHoney Candidates
Trio Inverted
Trio Spanning
Trio Edges
Genome
H. cydno
females
males
H. melpomene
females
males
3
scaffold−11800
scaffold−2394
scaffold−3526
scaffold−2374
scaffold−578
CDS
exon
mRNA
tRNA
rRNA
gene
Hmel1
HE671214
Hmel2
Hmel216013.3
1 101 bp
8 342 322
8 342 873
8 343 973
8 344 524
Chromosome 16 Position (bp)
FST
fd
dXY
1
0.5
0
0.1
0.05
0
8 337 373
8 342 873
8 343 973
8 349 473
629
0
685
955
983
700
811
974
982
684
575
Sites

Figure S14.41

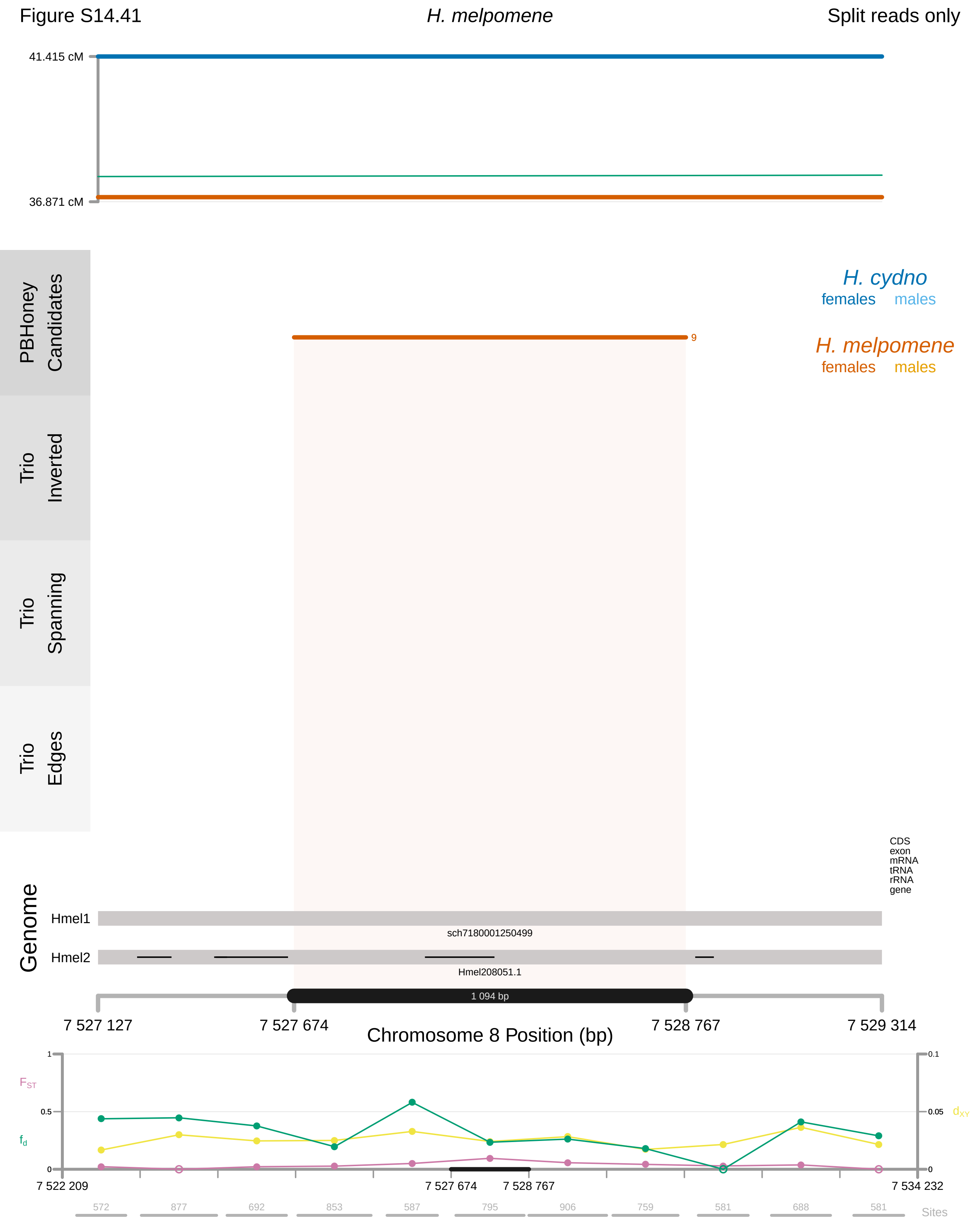

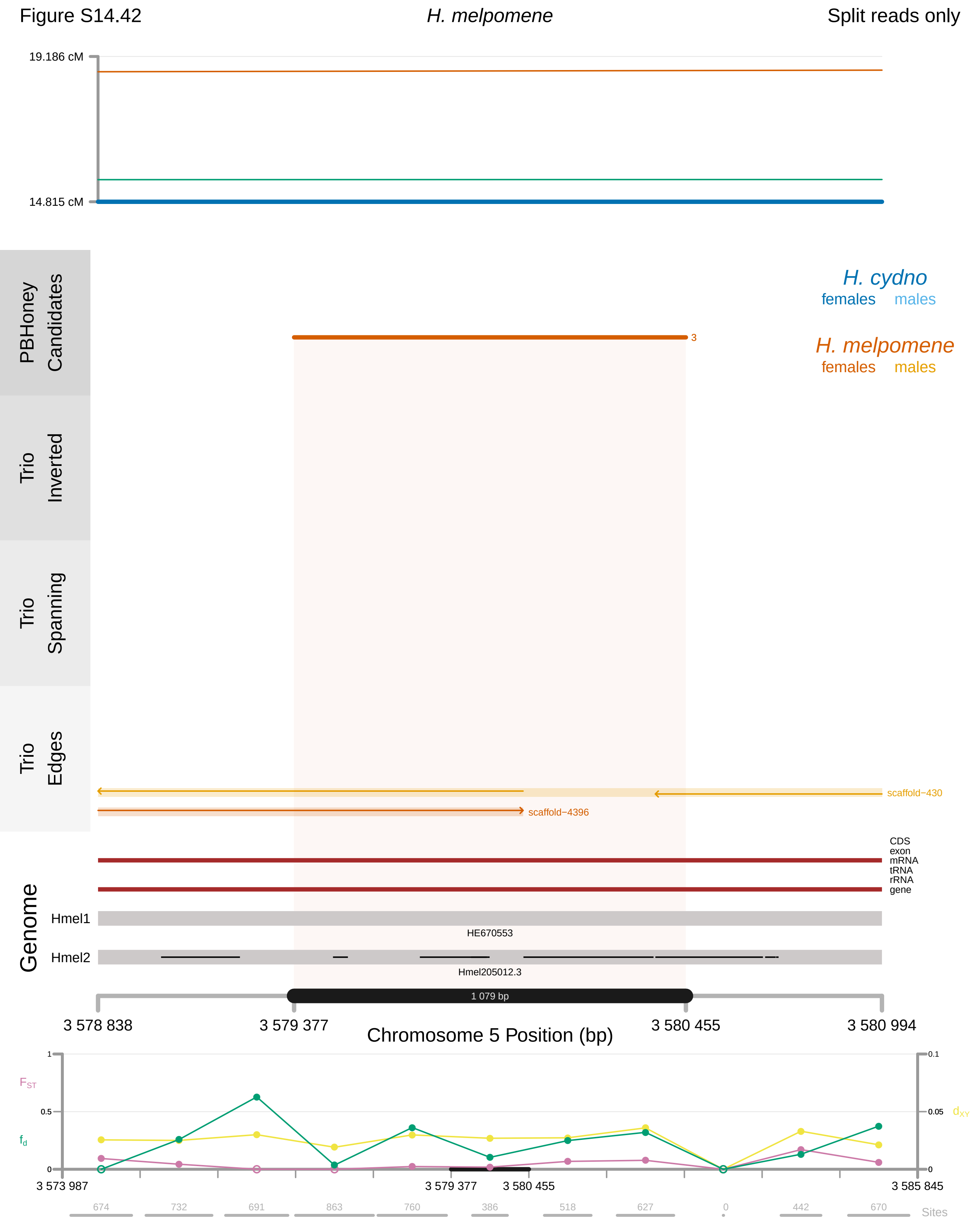
Figure S14.42
H. melpomene
Split reads only
19.186 cM
14.815 cM
PBHoney Candidates
Trio Inverted
Trio Spanning
Trio Edges
Genome
H. cydno
females males
H. melpomene
females males
3
scaffold−430
scaffold−4396
CDS
exon
mRNA
tRNA
rRNA
gene
Hmel1
HE670553
Hmel2
Hmel205012.3
1 079 bp
3 578 838
3 579 377
3 580 455
3 580 994
Chromosome 5 Position (bp)
F_{ST}
f_d
d_{XY}
1
0.5
0
0.1
0.05
3 573 987
3 579 377
3 580 455
3 585 845
674
732
691
863
760
386
518
627
0
442
670
Sites

Figure S14.43

H. melpomene

Split reads only

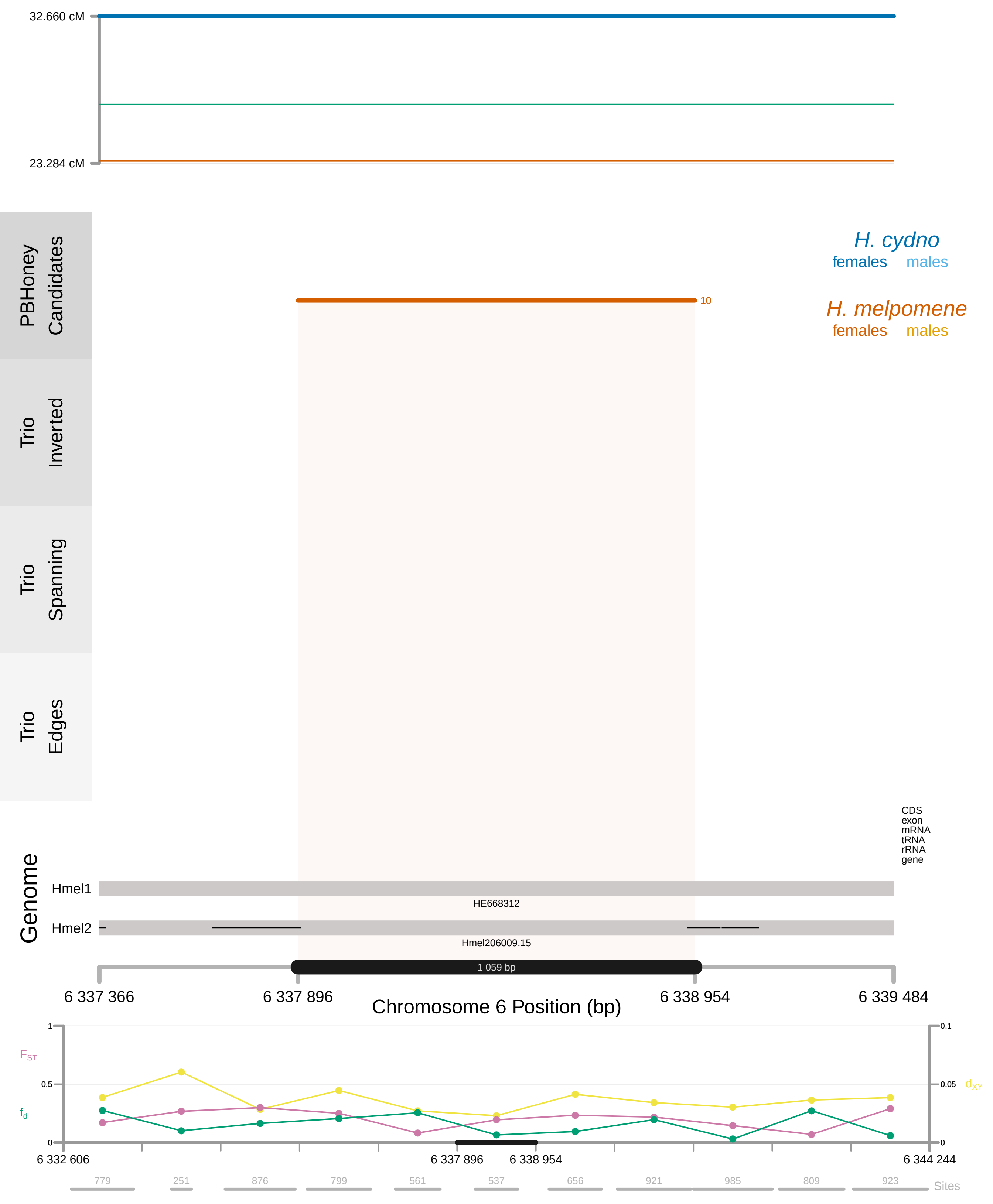

H. melpomene

Split reads only

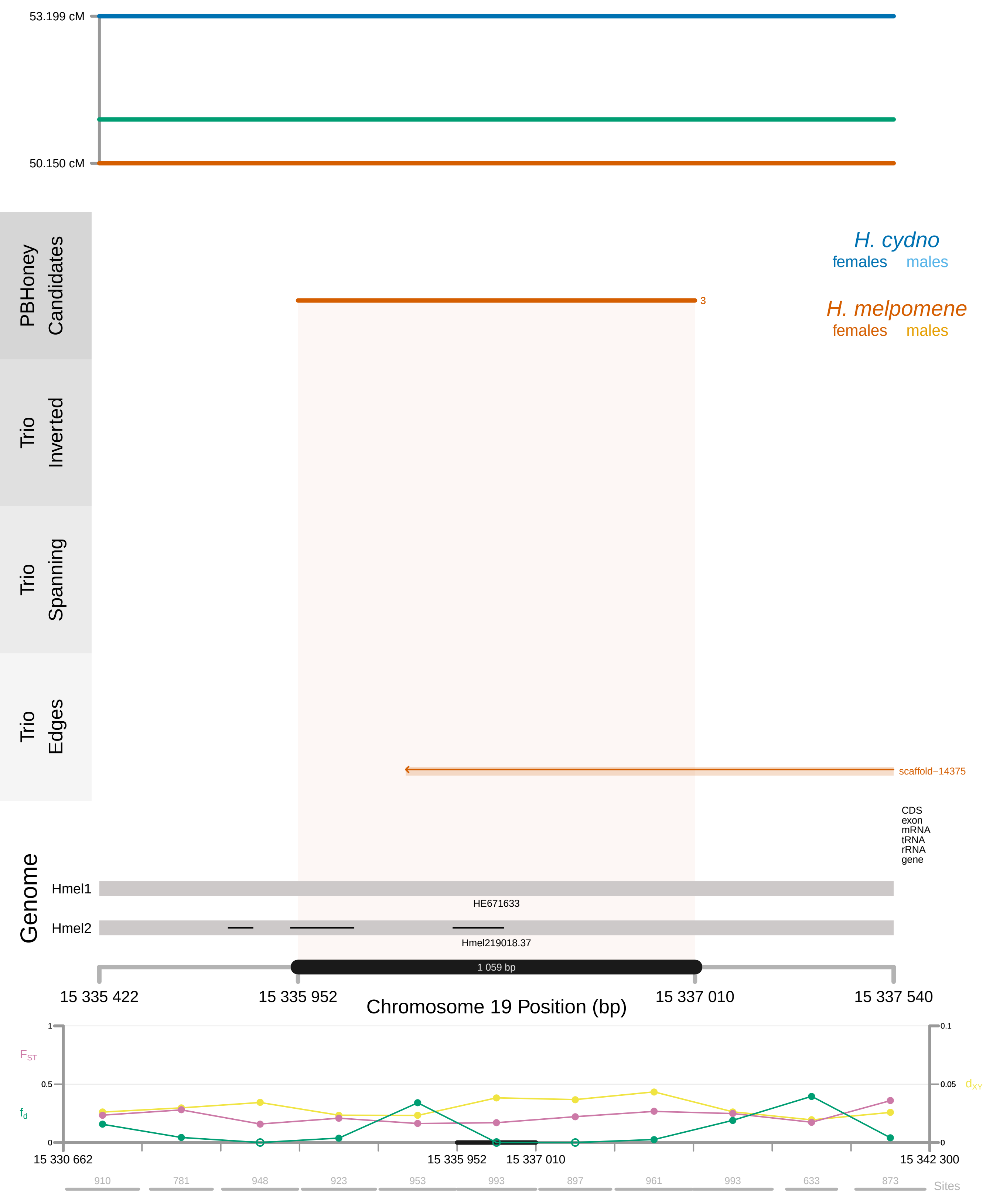

Figure S14.45

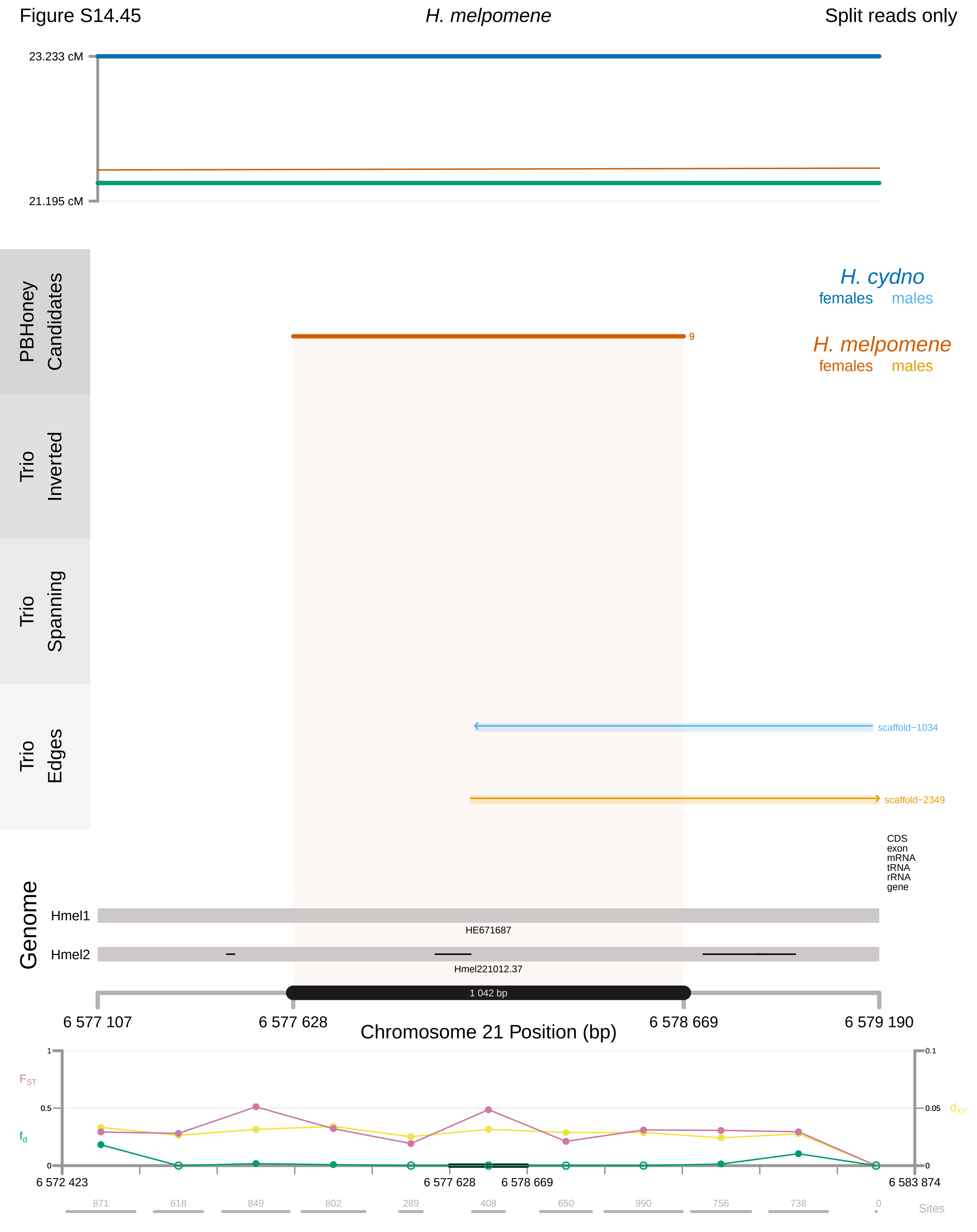

Figure S14.46 *H. melpomene* Split reads only

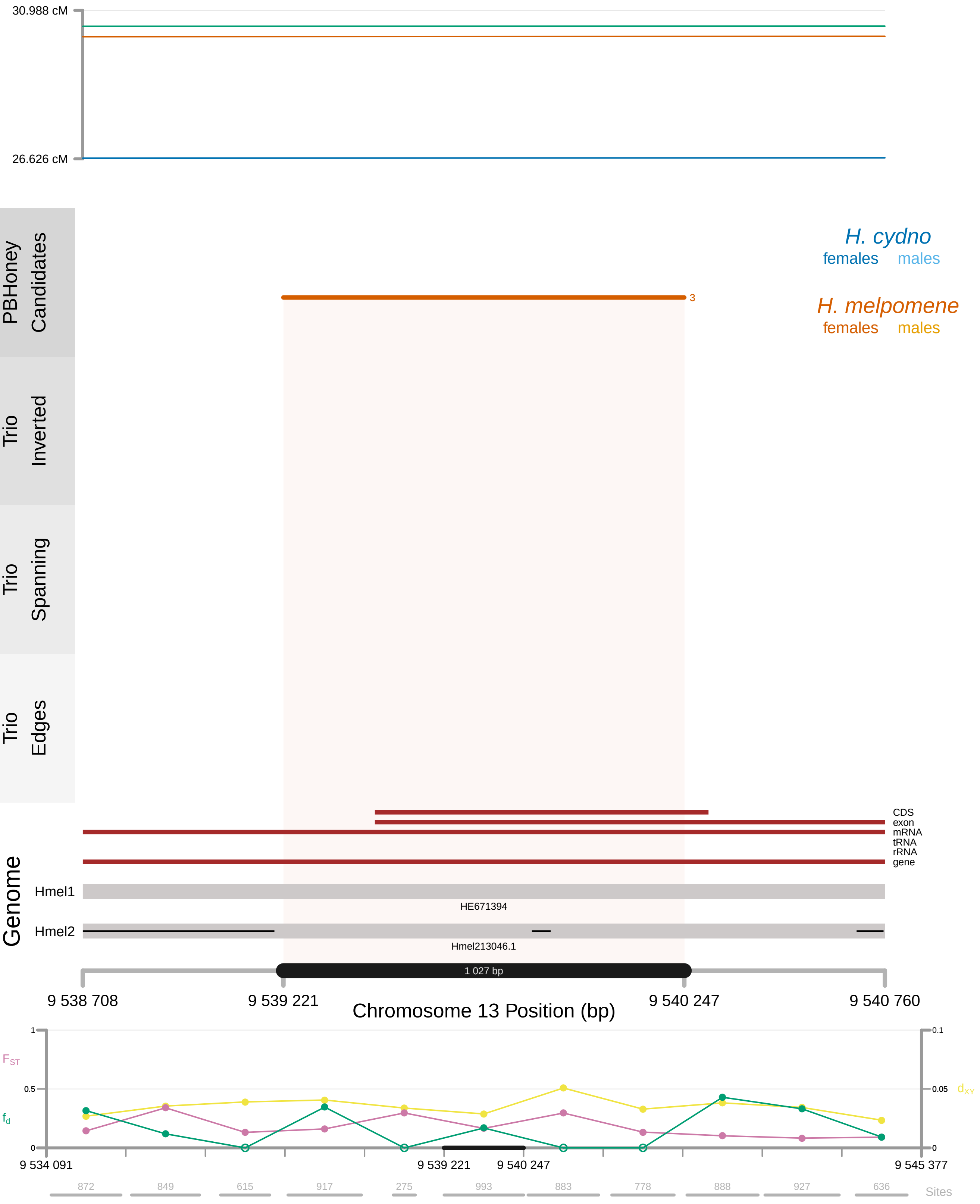

Supplement: Supplementary file 15 — S14, H. melpomene, split reads only. [file EVL3-1-138-s015.pdf]
