# Supplementary material for: No evidence for maintenance of a sympatric Heliconius species barrier by chromosomal inversions
Source: Evol Lett. 2017 Jun 14;1(3):138–54. doi: 10.1002/evl3.12 (PMC6122123; doi:10.1002/evl3.12)

Figure S15.1

Both species

Split reads and trio assembly

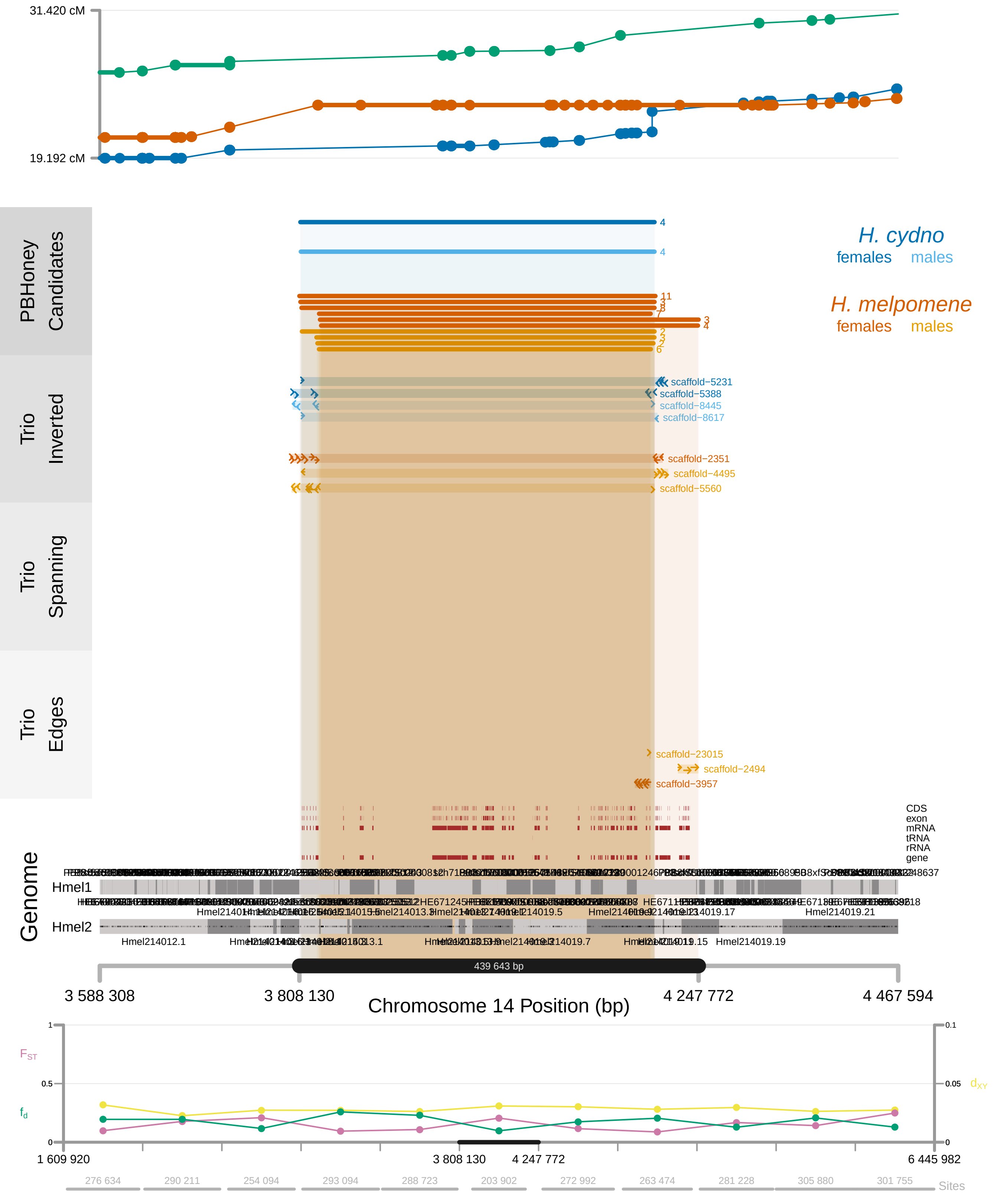

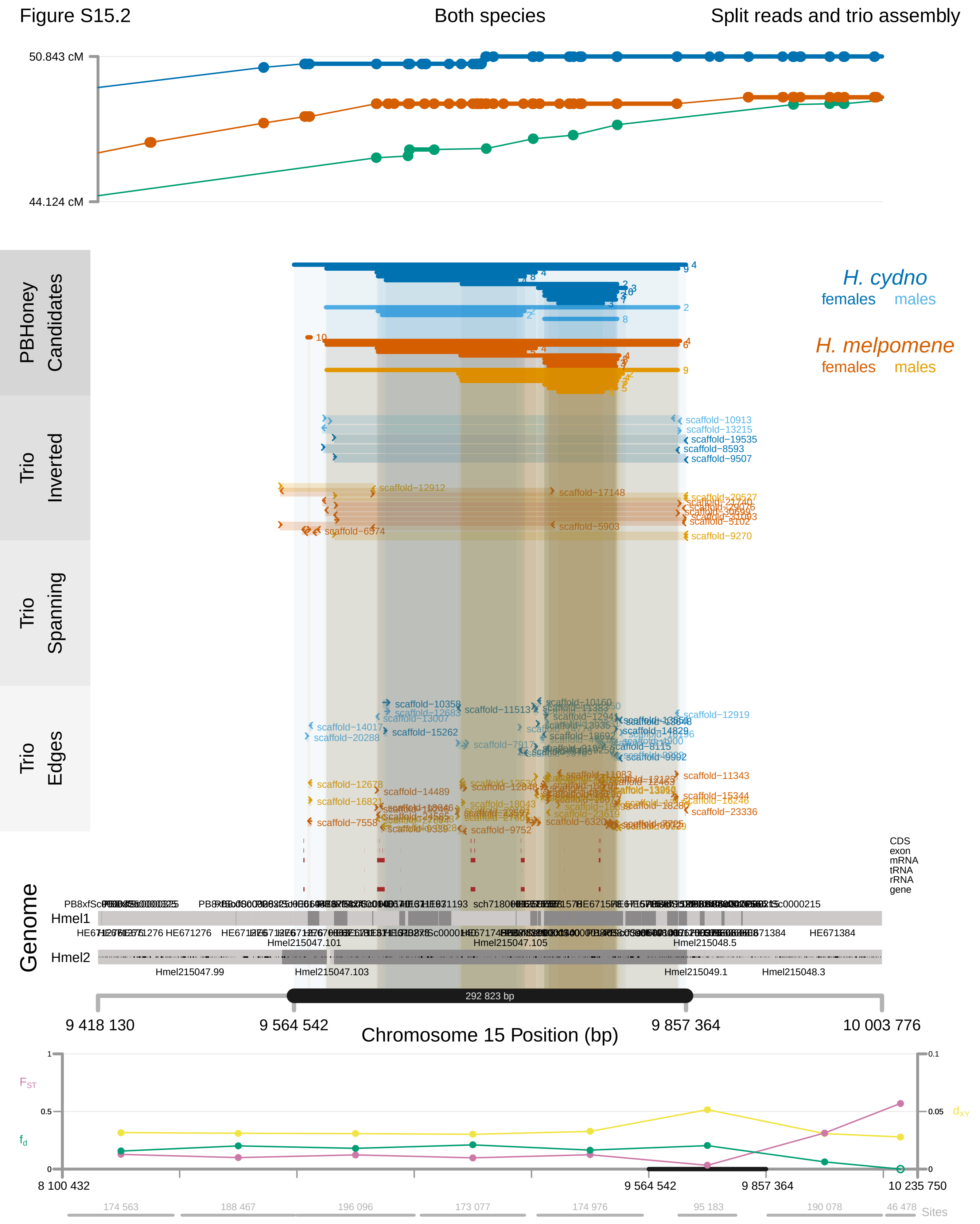

Figure S15.2
Both species
Split reads and trio assembly
50.843 cM
44.124 cM
PBHoney Candidates
Trio Inverted
Trio Spanning
Trio Edges
Genome
H. cydno
females males
H. melpomene
females males
CDS
exon
mRNA
tRNA
rRNA
gene
Hmel1
Hmel2
292 823 bp
9 418 130
9 564 542
9 857 364
10 003 776
Chromosome 15 Position (bp)
F_ST
f_d
d_XY
8 100 432
9 564 542
9 857 364
10 235 750
174 563
188 467
196 096
173 077
174 976
95 183
190 078
46 478
Sites

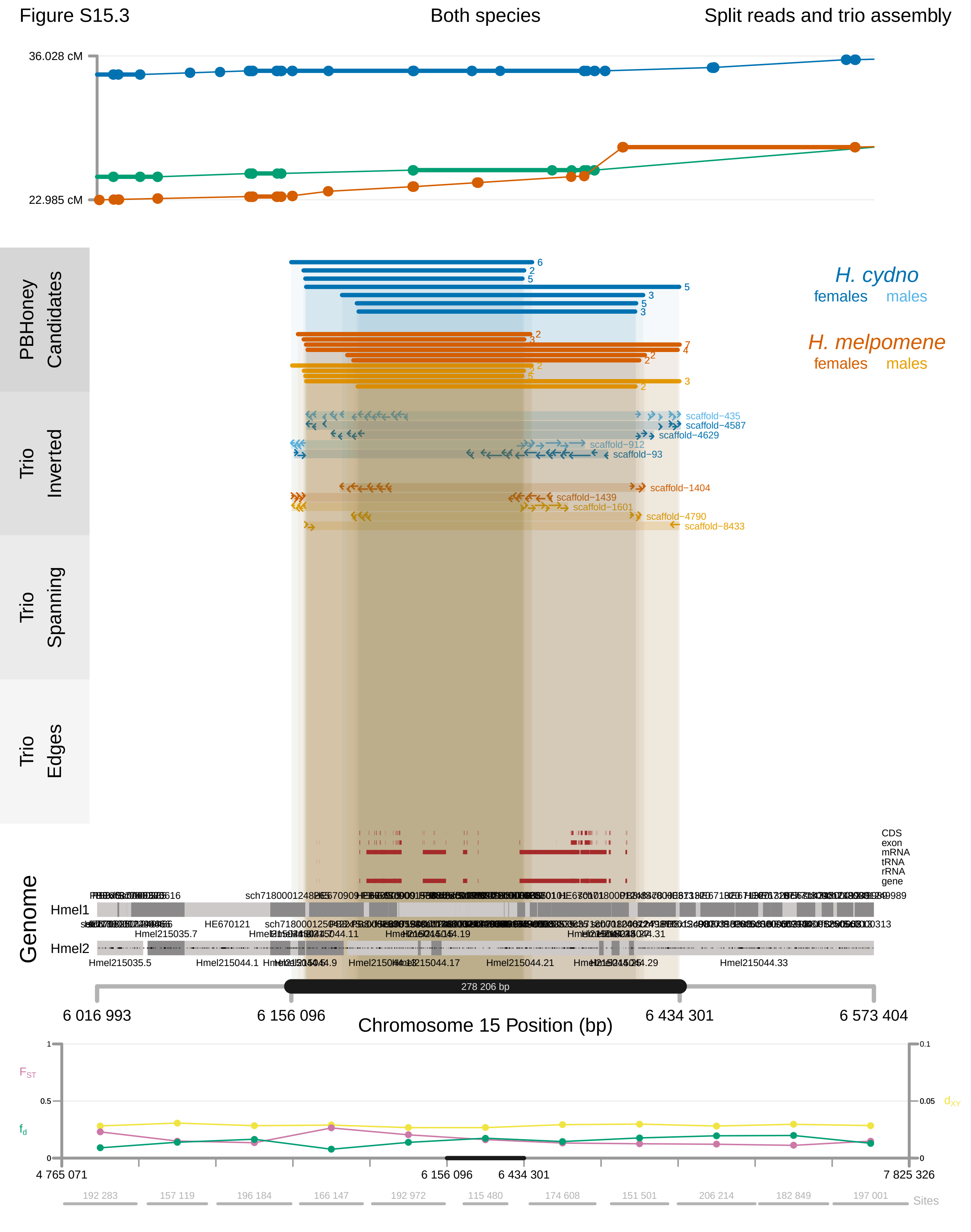

Figure S15.3
Both species
Split reads and trio assembly
36.028 cM
22.985 cM
PBHoney Candidates
Trio Inverted
Trio Spanning
Trio Edges
Genome
H. cydno
females males
H. melpomene
females males
scaffold−435
scaffold−4587
scaffold−4629
scaffold−912
scaffold−93
scaffold−1404
scaffold−1439
scaffold−1601
scaffold−4790
scaffold−8433
CDS
exon
mRNA
tRNA
rRNA
gene
Hmel1
Hmel2
Hmel215035.7
HE670121
Hmel215035.5
Hmel215044.1
Hmel215044.17
Hmel215044.21
Hmel215044.33
278 206 bp
6 016 993
6 156 096
6 434 301
6 573 404
Chromosome 15 Position (bp)
F_ST
f_d
d_XY
4 765 071
7 825 326
192 283
157 119
196 184
166 147
192 972
115 480
174 608
151 501
206 214
182 849
197 001
Sites

Figure S15.4

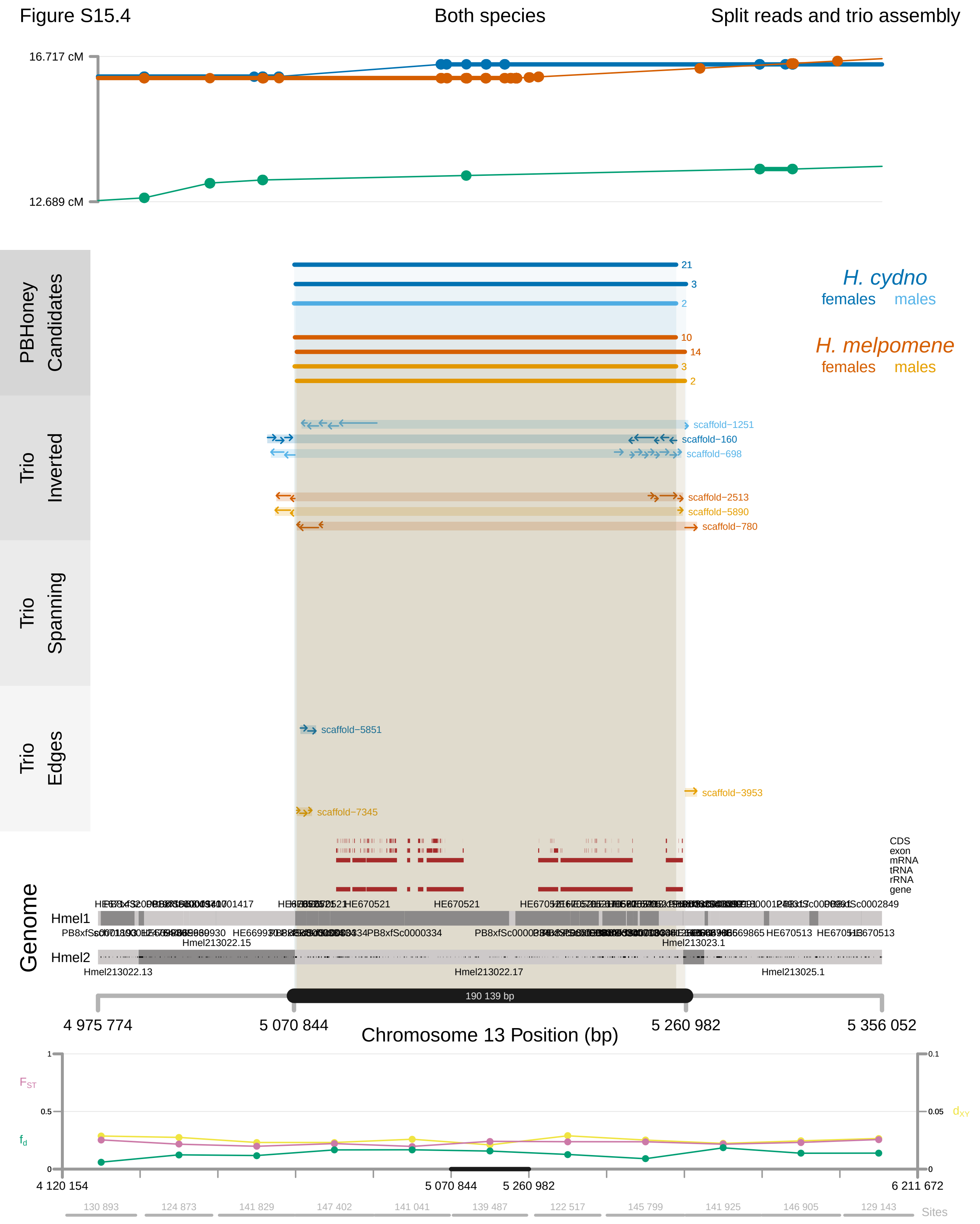
Both species
Split reads and trio assembly
16.717 cM
12.689 cM
PBHoney Candidates
Trio Inverted
Trio Spanning
Trio Edges
Genome
H. cydno
females
males
H. melpomene
females
males
21
3
2
10
14
3
2
scaffold−1251
scaffold−160
scaffold−698
scaffold−2513
scaffold−5890
scaffold−780
scaffold−5851
scaffold−3953
scaffold−7345
CDS
exon
mRNA
tRNA
rRNA
gene
Hmel1
Hmel2
Hmel213022.15
Hmel213023.1
Hmel213022.13
Hmel213022.17
Hmel213025.1
190 139 bp
4 975 774
5 070 844
5 260 982
5 356 052
Chromosome 13 Position (bp)
FST
fd
dXY
1
0.5
0
0.1
0.05
0
4 120 154
5 070 844
5 260 982
6 211 672
130 893
124 873
141 829
147 402
141 041
139 487
122 517
145 799
141 925
146 905
129 143
Sites

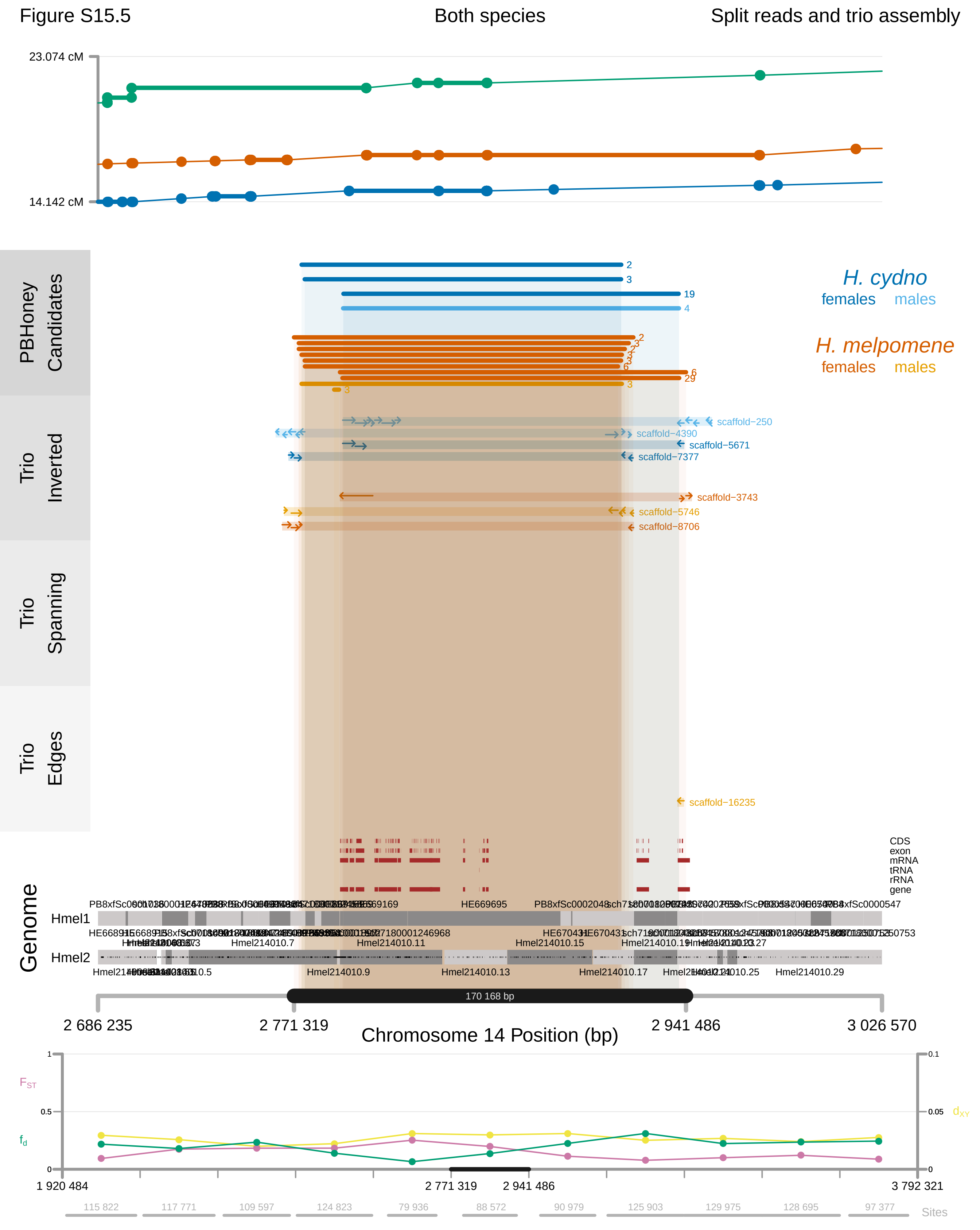

Figure S15.5
Both species
Split reads and trio assembly
23.074 cM
14.142 cM
PBHoney Candidates
Trio Inverted
Trio Spanning
Trio Edges
Genome
H. cydno
females males
H. melpomene
females males
scaffold−250
scaffold−4390
scaffold−5671
scaffold−7377
scaffold−3743
scaffold−5746
scaffold−8706
scaffold−16235
CDS
exon
mRNA
tRNA
rRNA
gene
Hmel1
Hmel2
HE669695
Hmel214010.7
Hmel214010.11
Hmel214010.15
Hmel214010.9
Hmel214010.13
Hmel214010.17
Hmel214010.29
170 168 bp
2 686 235
2 771 319
2 941 486
3 026 570
Chromosome 14 Position (bp)
FST
fd
dXY
1 920 484
2 771 319
2 941 486
3 792 321
115 822
117 771
109 597
124 823
79 936
88 572
90 979
125 903
129 975
128 695
97 377
Sites

Figure S15.6 Both species Split reads and trio assembly

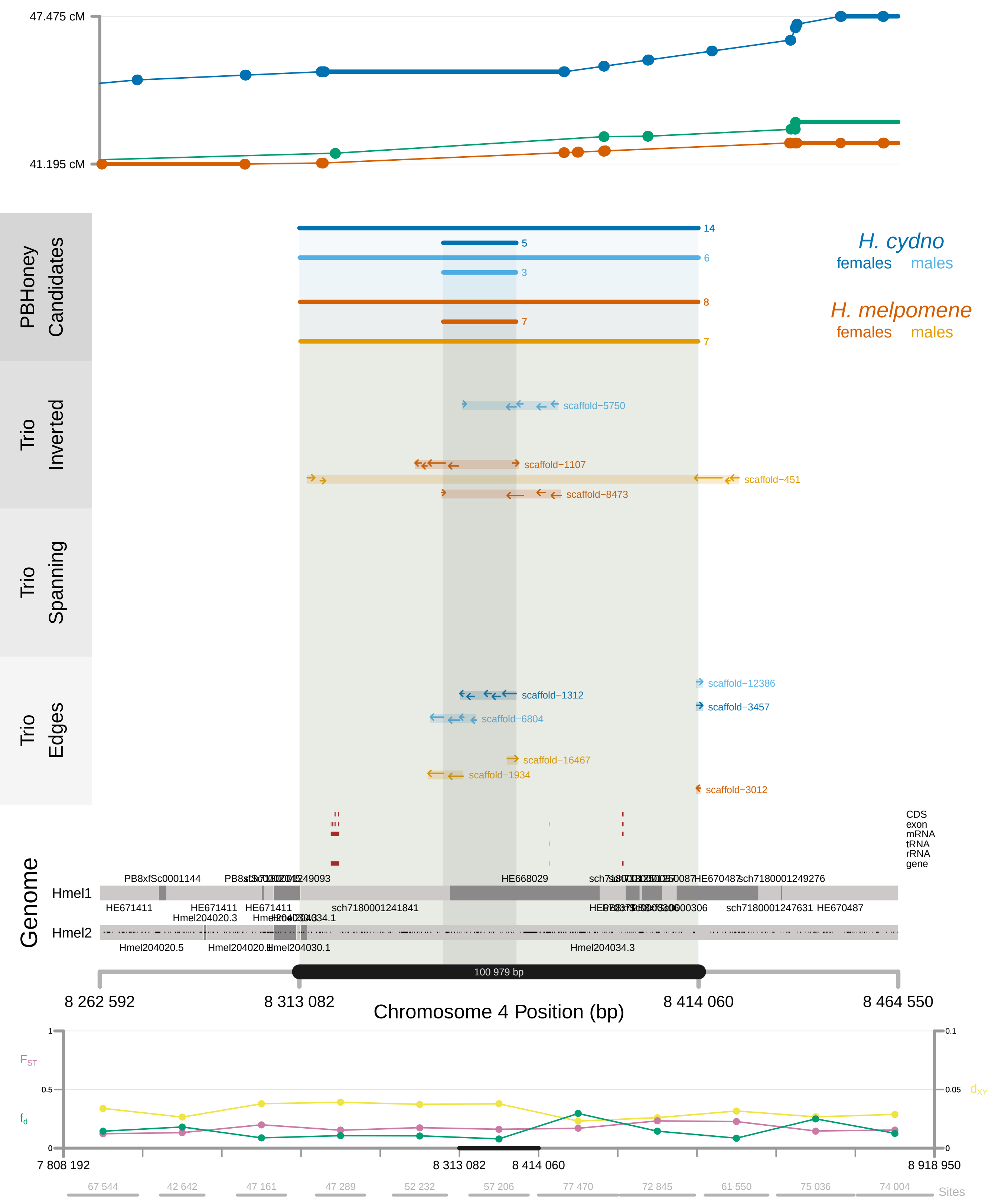

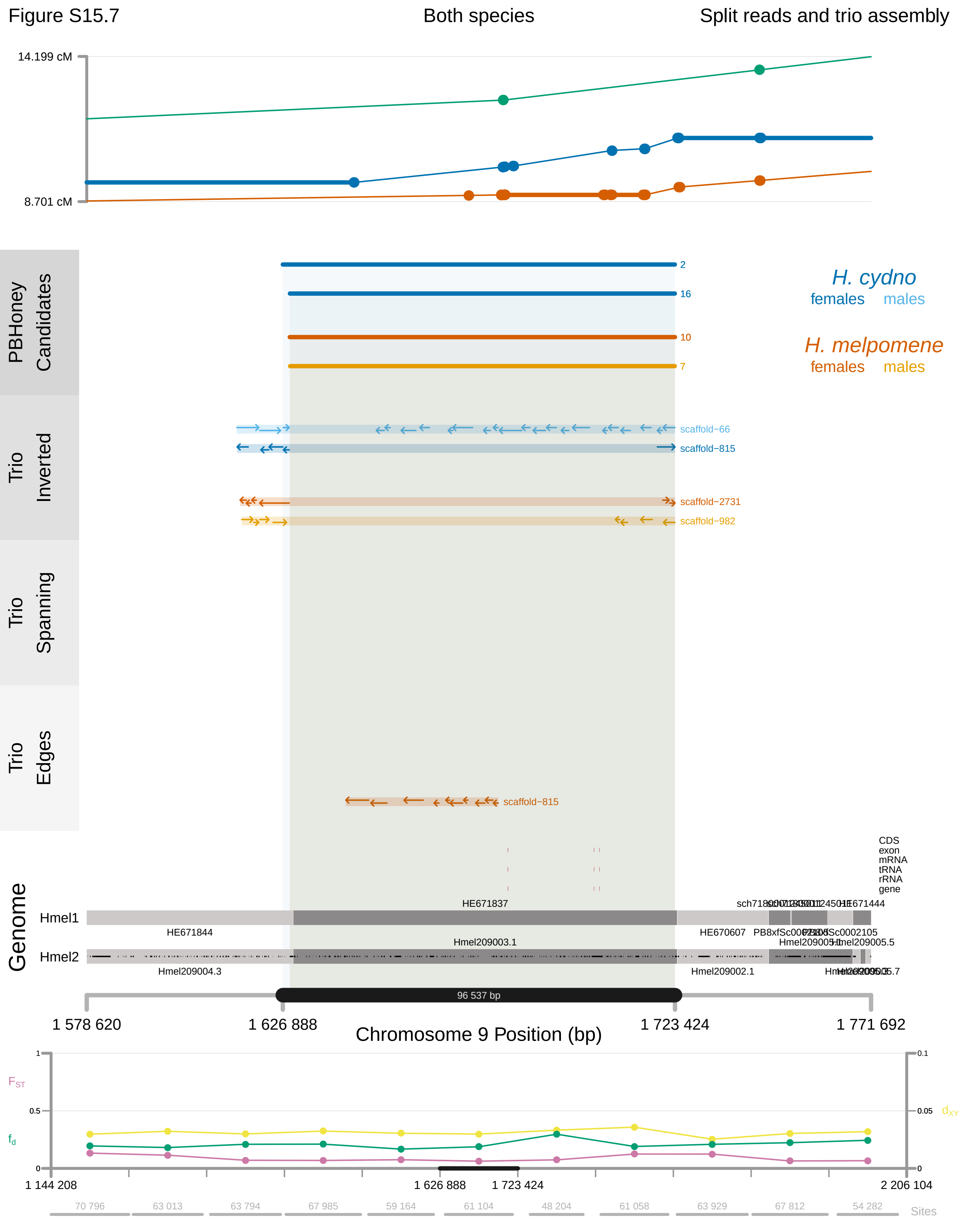

Figure S15.7
Both species
Split reads and trio assembly
14.199 cM
8.701 cM
PBHoney Candidates
Trio Inverted
Trio Spanning
Trio Edges
Genome
H. cydno
females
males
H. melpomene
females
males
2
16
10
7
scaffold−66
scaffold−815
scaffold−2731
scaffold−982
scaffold−815
CDS
exon
mRNA
tRNA
rRNA
gene
Hmel1
Hmel2
HE671837
HE671844
HE670607
HE671444
Hmel209003.1
Hmel209004.3
Hmel209002.1
96 537 bp
1 578 620
1 626 888
1 723 424
1 771 692
Chromosome 9 Position (bp)
FST
fd
dXY
1
0.5
0
0.1
0.05
1 144 208
2 206 104
70 796
63 013
63 794
67 985
59 164
61 104
48 204
61 058
63 929
67 812
54 282
Sites

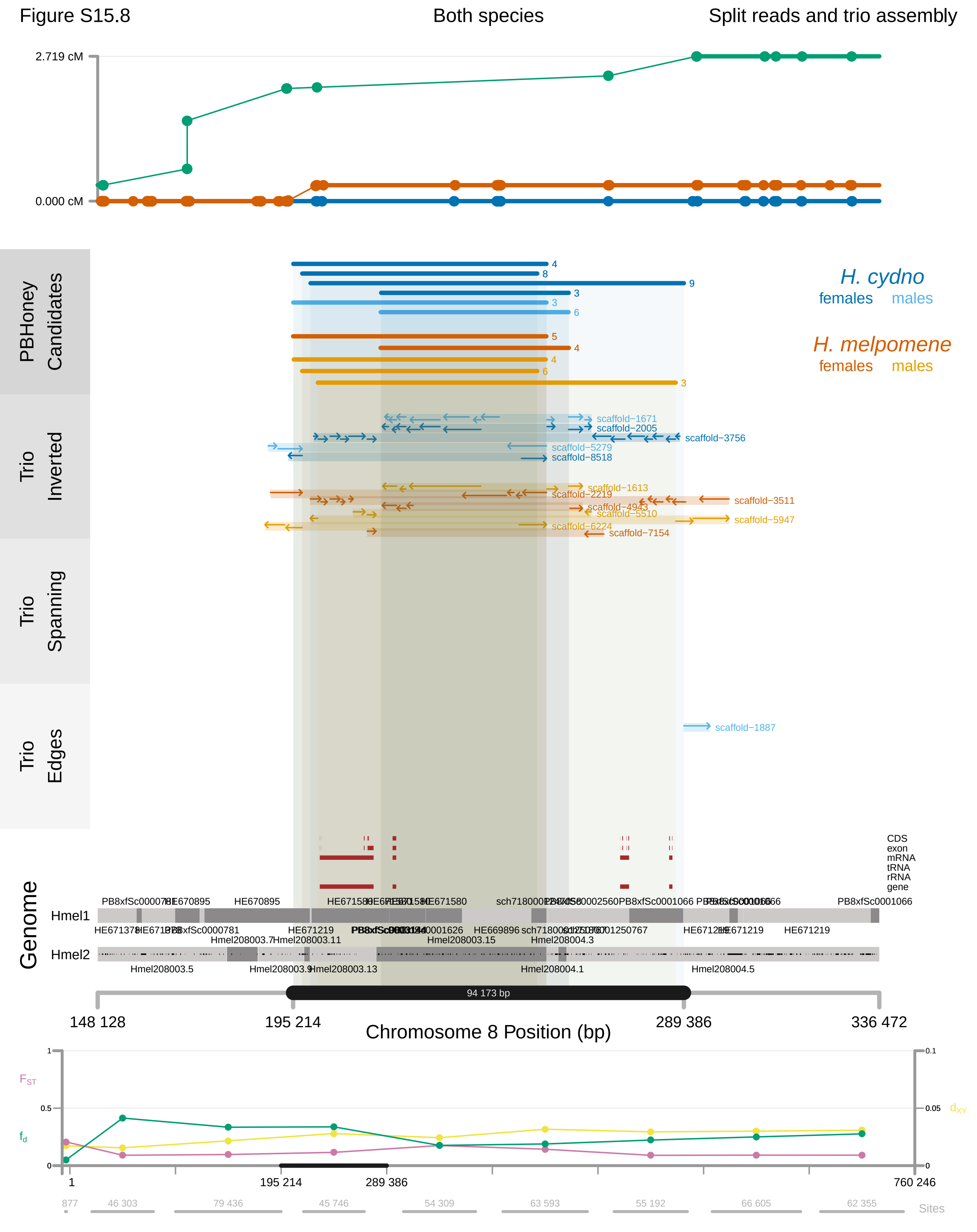
Figure S15.8
Both species
Split reads and trio assembly
2.719 cM
0.000 cM
PBHoney Candidates
Trio Inverted
Trio Spanning
Trio Edges
Genome
H. cydno
females males
H. melpomene
females males
scaffold−1671
scaffold−2005
scaffold−3756
scaffold−5279
scaffold−8518
scaffold−1613
scaffold−2219
scaffold−3511
scaffold−4943
scaffold−5510
scaffold−5947
scaffold−6224
scaffold−7154
scaffold−1887
CDS
exon
mRNA
tRNA
rRNA
gene
Hmel1
Hmel2
94 173 bp
148 128
195 214
289 386
336 472
Chromosome 8 Position (bp)
FST
fd
dXY
1
0.5
0
0.1
0.05
760 246
Sites

Figure S15.9 Both species Split reads and trio assembly

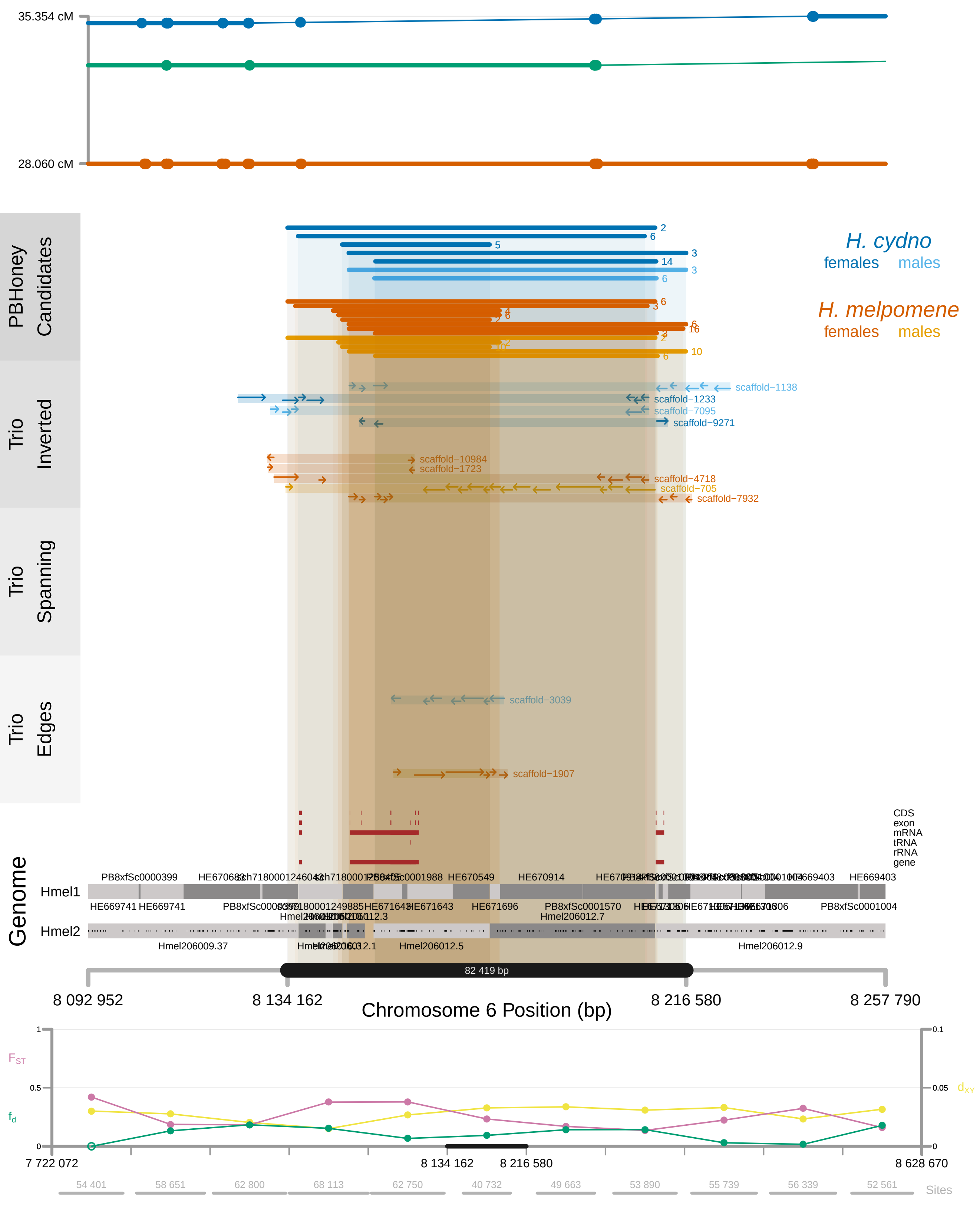

Figure S15.10 Both species Split reads and trio assembly

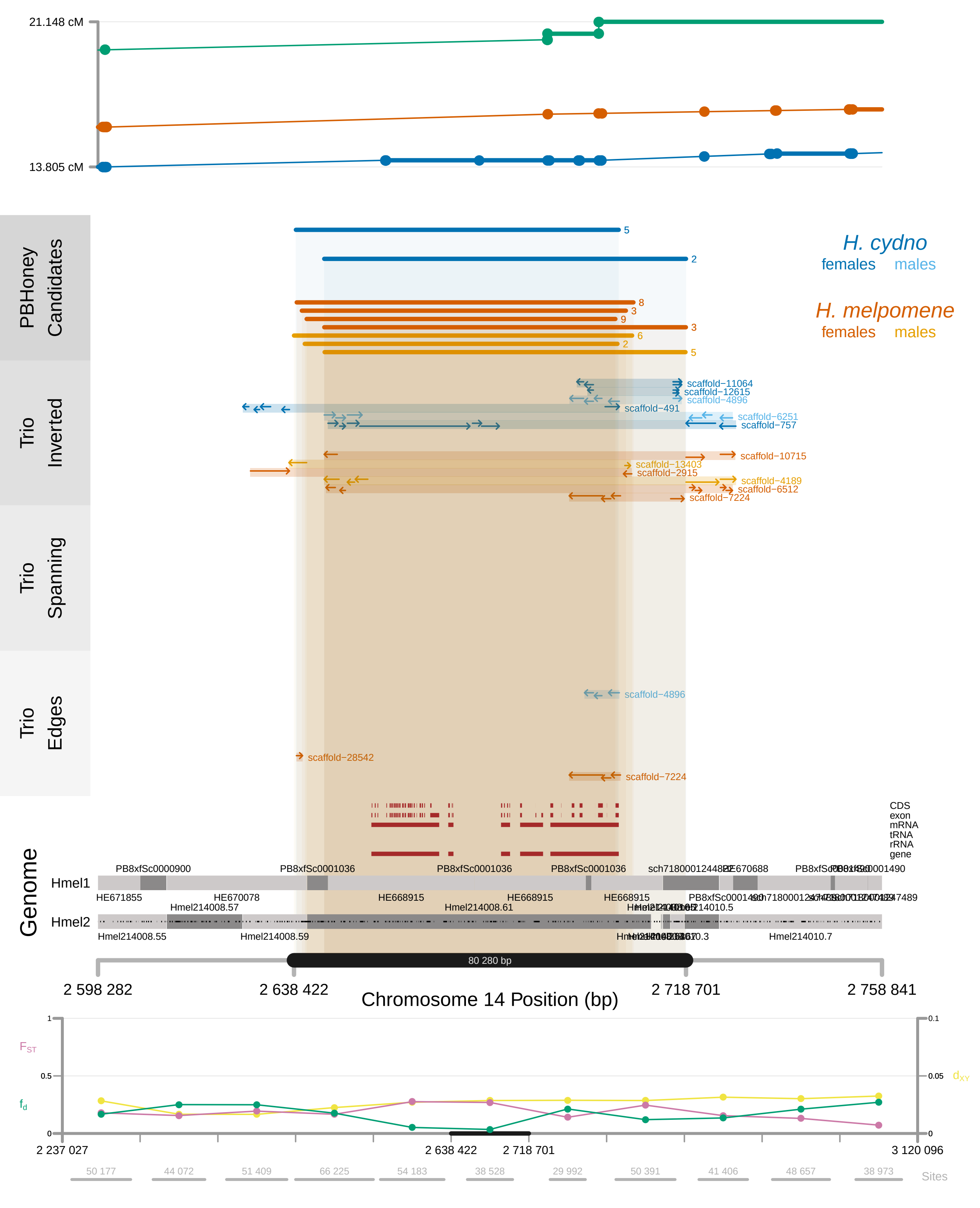

Figure S15.11 Both species Split reads and trio assembly

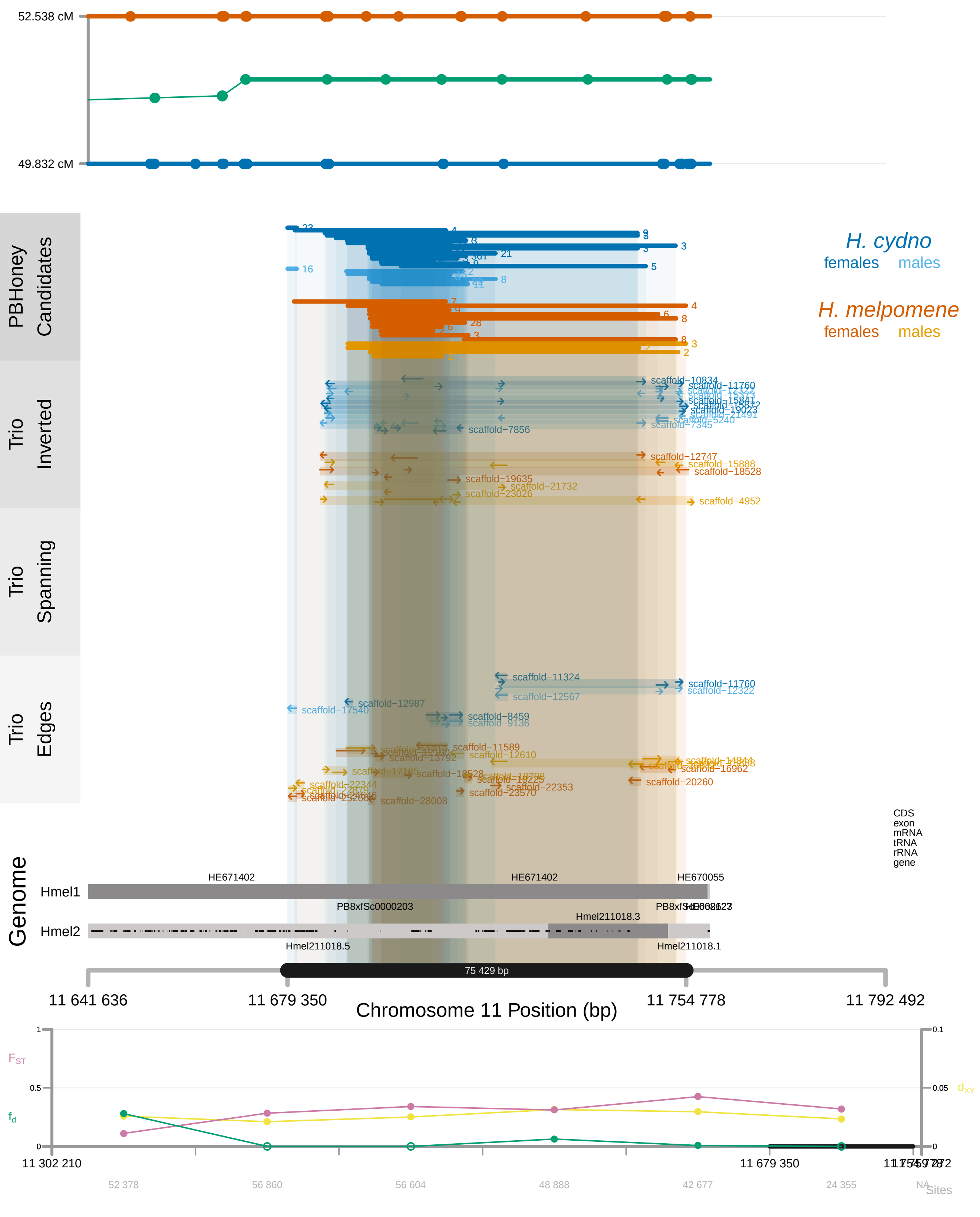

Figure S15.12 Both species Split reads and trio assembly

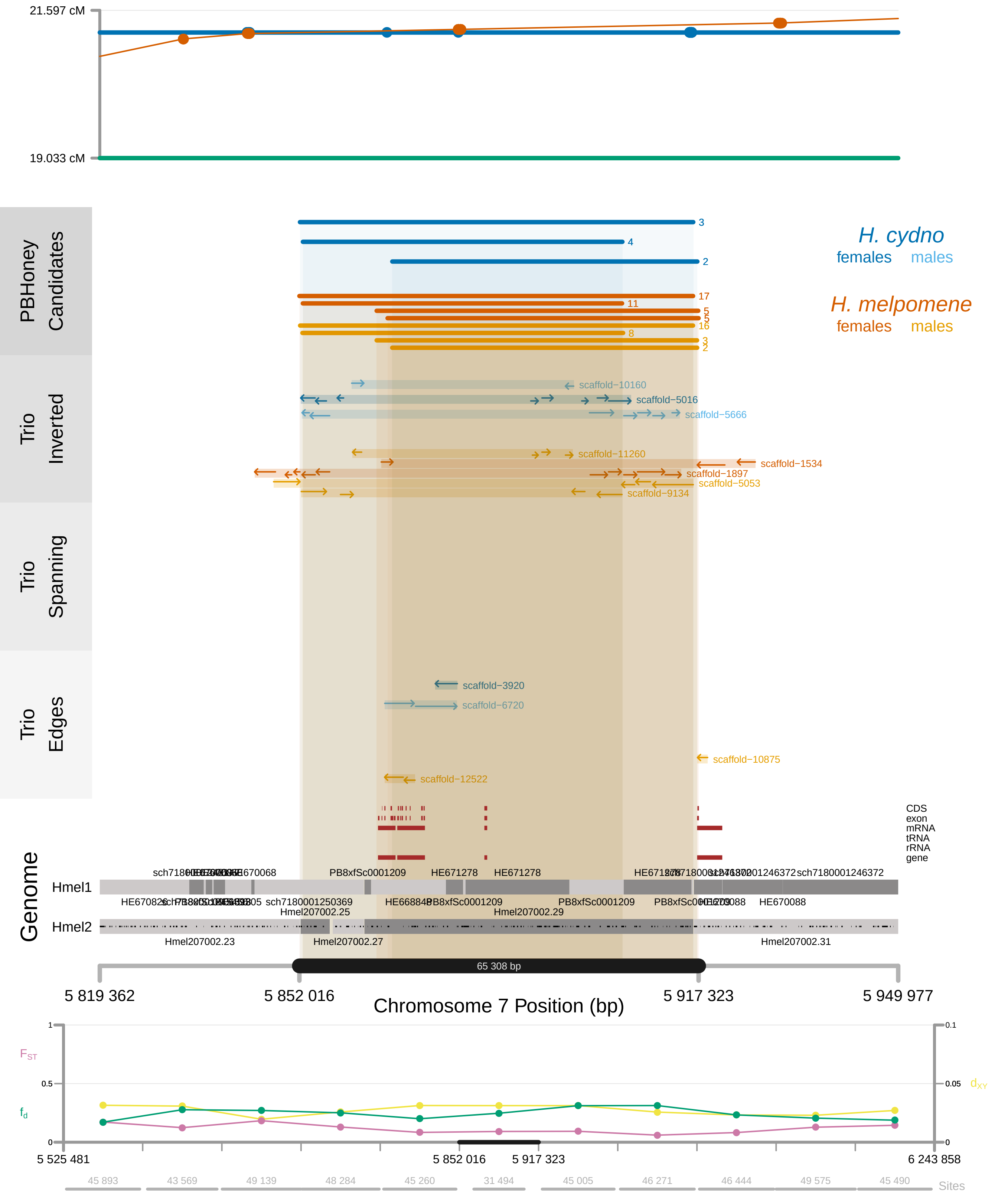

Figure S15.13

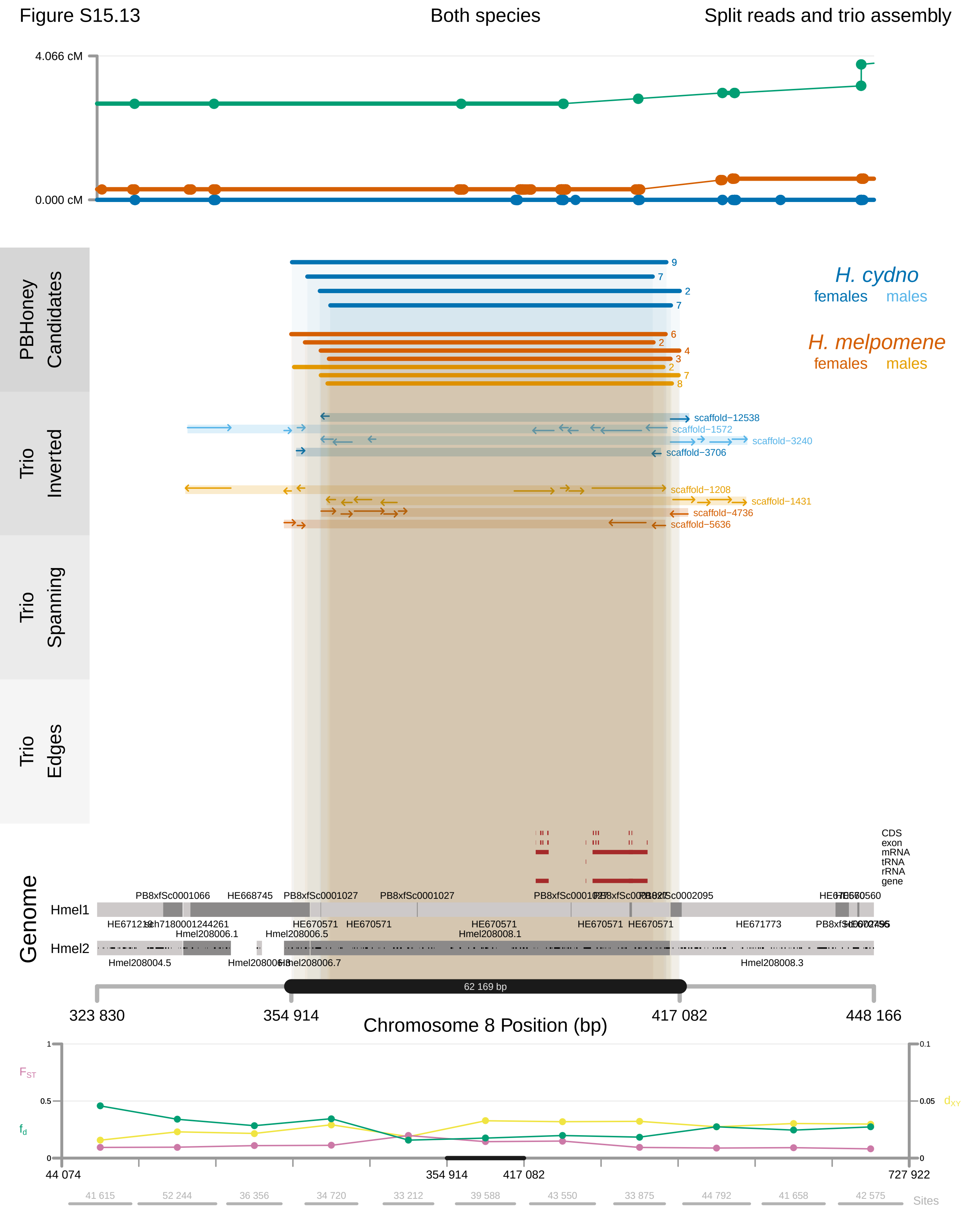

Figure S15.14 Both species Split reads and trio assembly

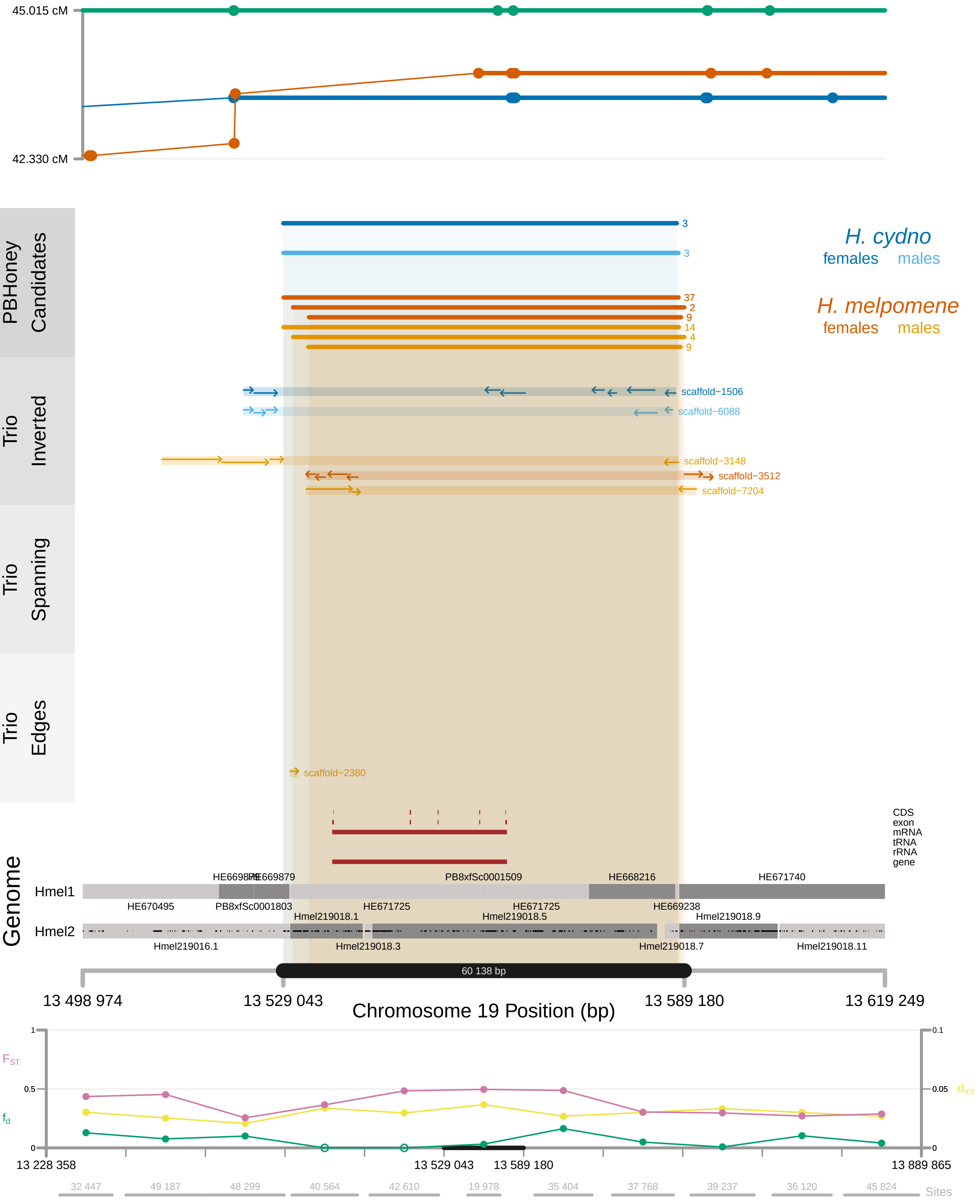

Figure S15.15

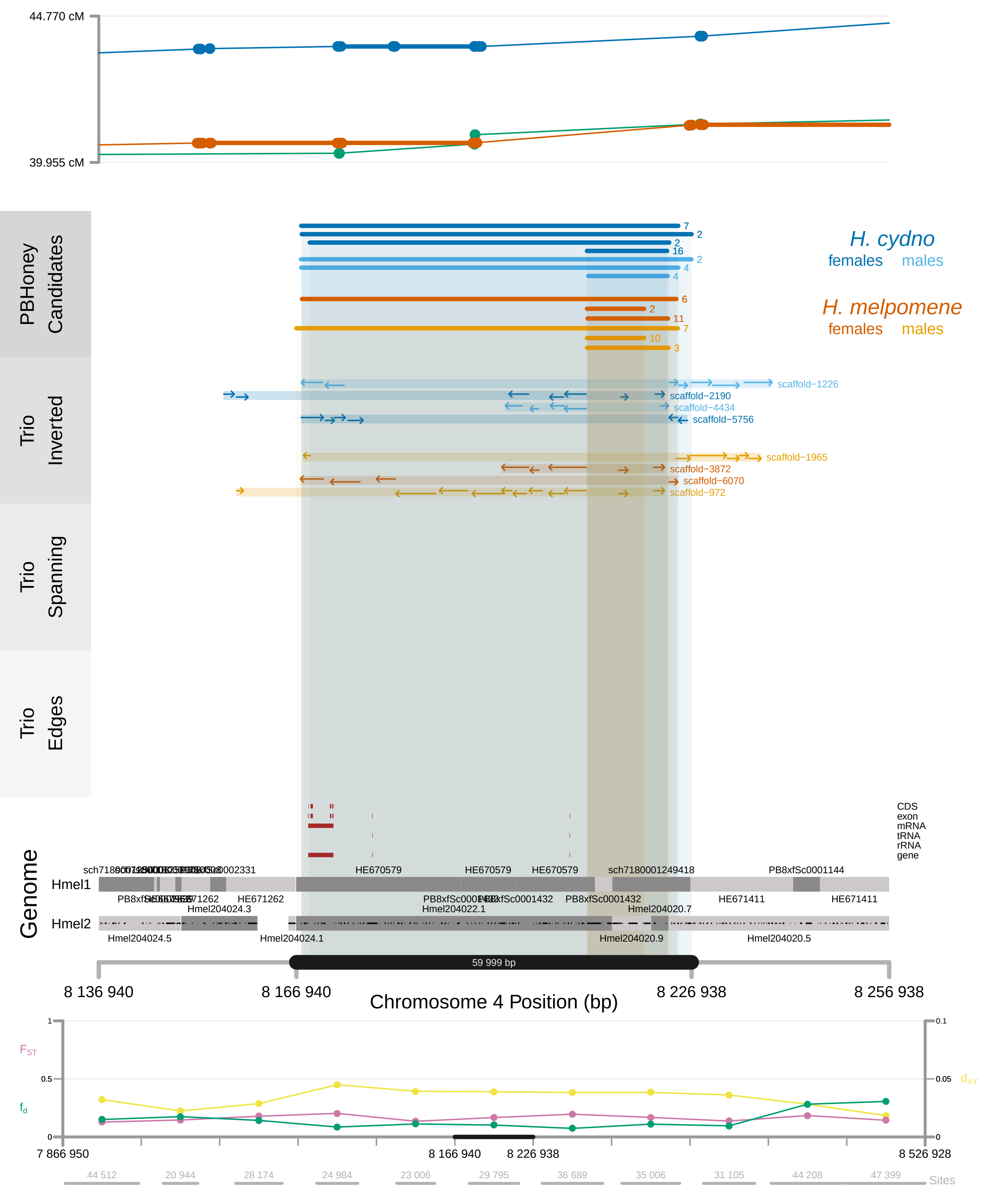

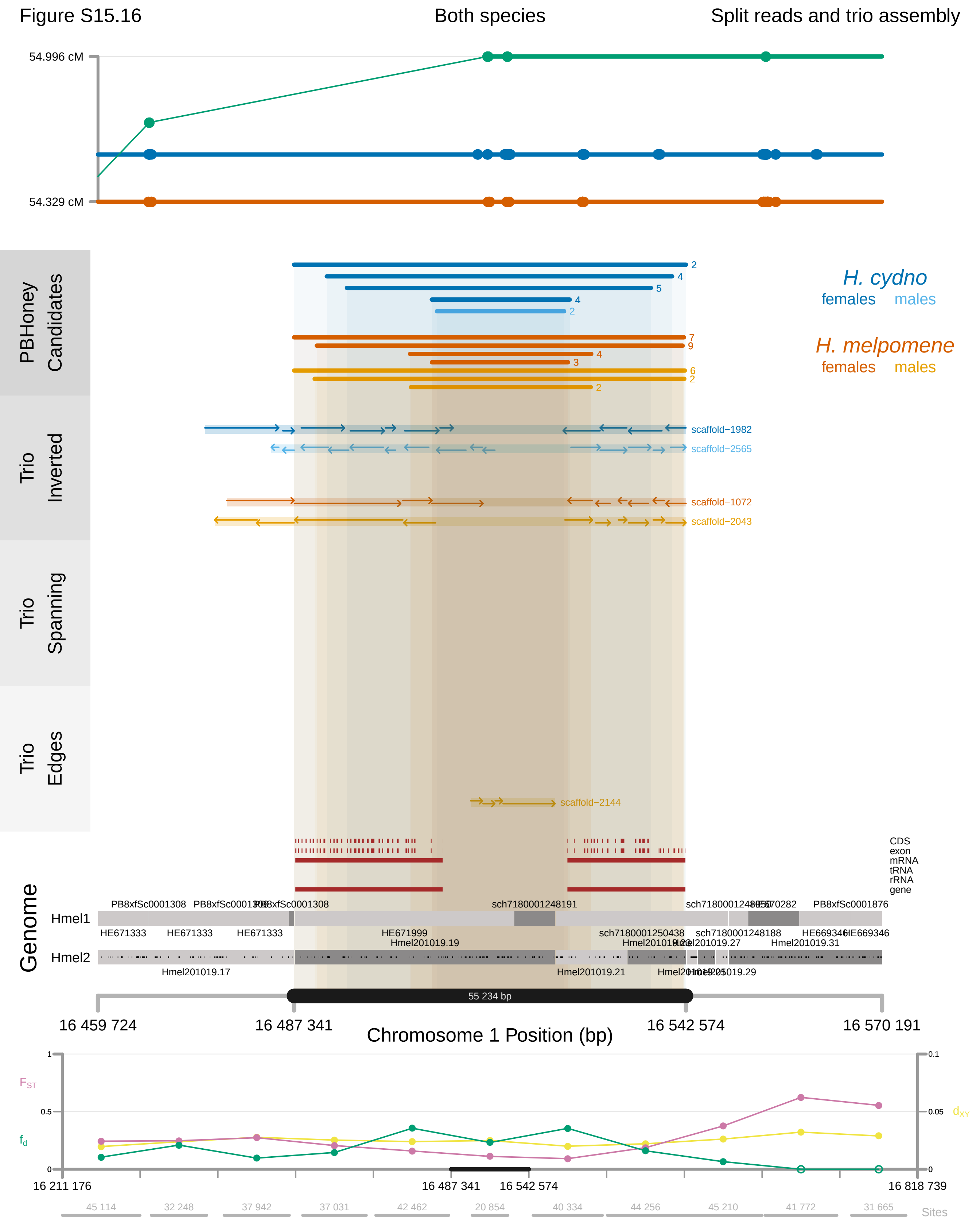
Figure S15.16
Both species
Split reads and trio assembly
54.996 cM
54.329 cM
PBHoney Candidates
Trio Inverted
Trio Spanning
Trio Edges
Genome
H. cydno
females males
H. melpomene
females males
scaffold−1982
scaffold−2565
scaffold−1072
scaffold−2043
scaffold−2144
CDS
exon
mRNA
tRNA
rRNA
gene
Hmel1
Hmel2
55 234 bp
16 459 724
16 487 341
16 542 574
16 570 191
Chromosome 1 Position (bp)
F_{ST}
f_d
d_{XY}
16 211 176
16 487 341
16 542 574
16 818 739
Sites

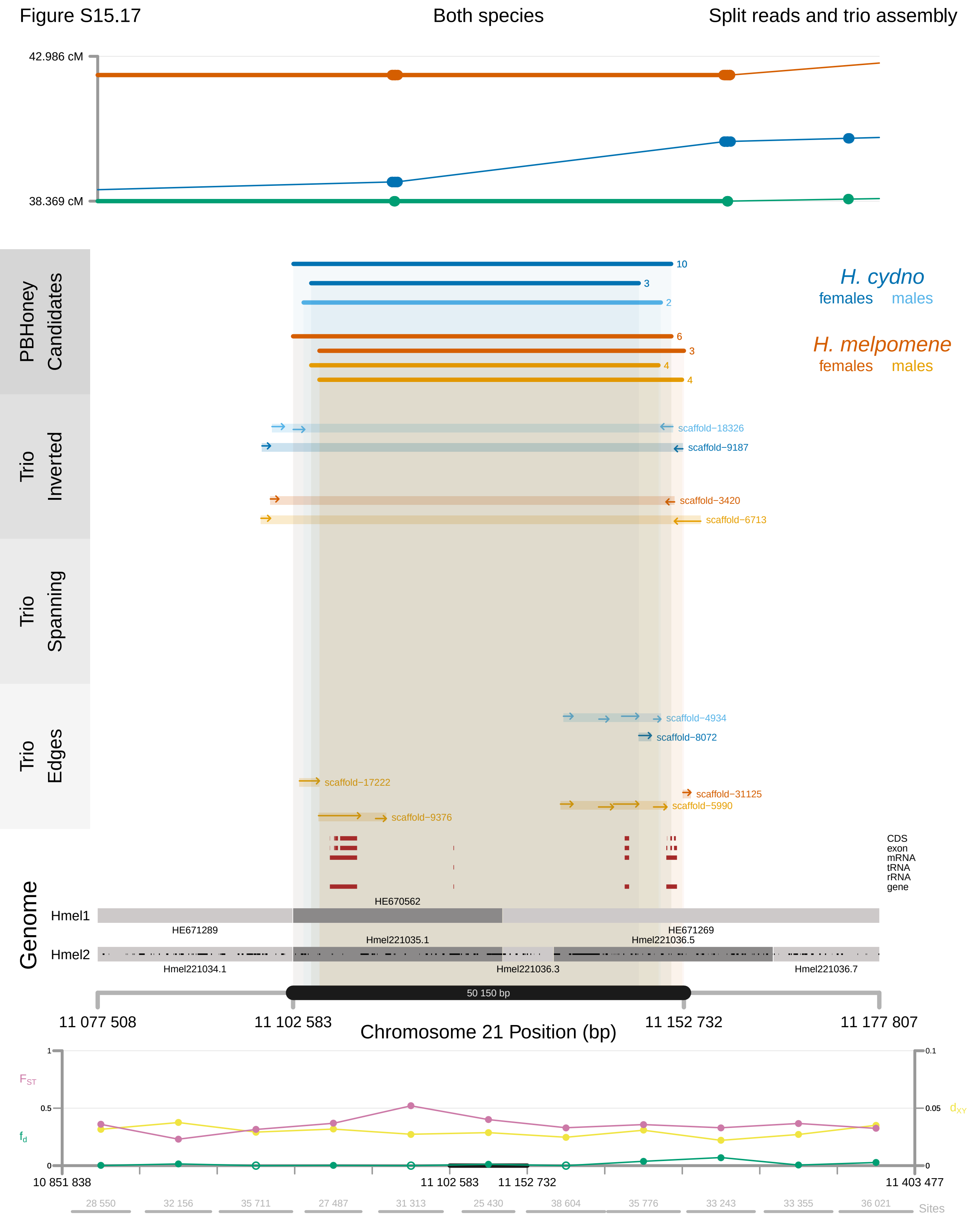

Figure S15.17
Both species
Split reads and trio assembly
42.986 cM
38.369 cM
PBHoney Candidates
Trio Inverted
Trio Spanning
Trio Edges
Genome
H. cydno
females
males
H. melpomene
females
males
10
3
2
6
3
4
4
scaffold−18326
scaffold−9187
scaffold−3420
scaffold−6713
scaffold−4934
scaffold−8072
scaffold−17222
scaffold−31125
scaffold−5990
scaffold−9376
CDS
exon
mRNA
tRNA
rRNA
gene
HE670562
Hmel1
HE671289
HE671269
Hmel221035.1
Hmel221036.5
Hmel2
Hmel221034.1
Hmel221036.3
Hmel221036.7
50 150 bp
11 077 508
11 102 583
11 152 732
11 177 807
Chromosome 21 Position (bp)
FST
fd
dXY
1
0.5
0
0.1
0.05
0
10 851 838
11 102 583
11 152 732
11 403 477
28 550
32 156
35 711
27 487
31 313
25 430
38 604
35 776
33 243
33 355
36 021
Sites

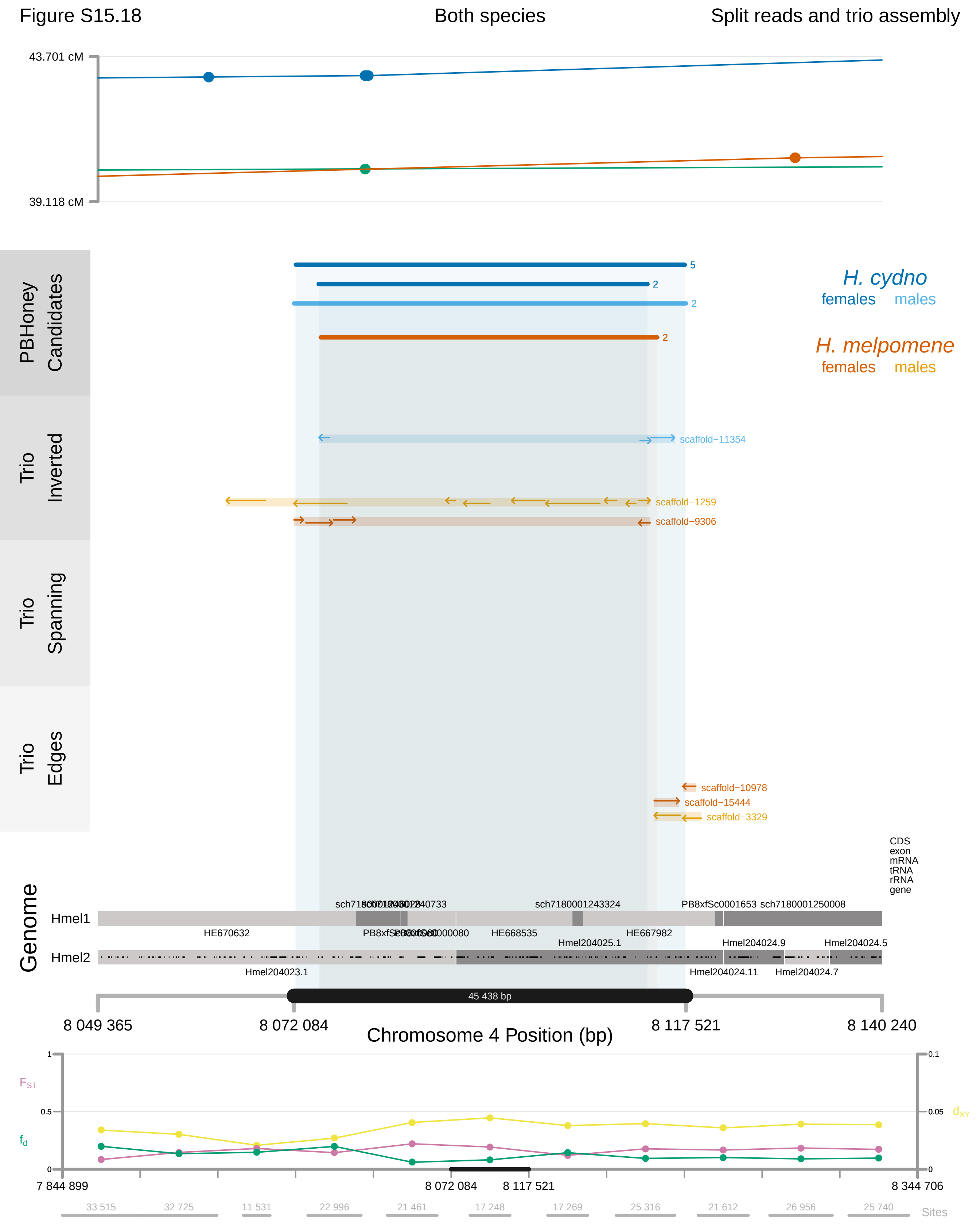

Figure S15.18
Both species
Split reads and trio assembly
43.701 cM
39.118 cM
PBHoney Candidates
Trio Inverted
Trio Spanning
Trio Edges
Genome
H. cydno
females
males
H. melpomene
females
males
5
2
2
2
scaffold−11354
scaffold−1259
scaffold−9306
scaffold−10978
scaffold−15444
scaffold−3329
CDS
exon
mRNA
tRNA
rRNA
gene
Hmel1
Hmel2
HE670632
HE668535
HE667982
PB8xfSc0001653
sch7180001250008
sch7180001243324
Hmel204023.1
Hmel204025.1
Hmel204024.9
Hmel204024.5
Hmel204024.11
Hmel204024.7
45 438 bp
8 049 365
8 072 084
8 117 521
8 140 240
Chromosome 4 Position (bp)
FST
fd
dXY
1
0.5
0
0.1
0.05
0
7 844 899
8 072 084
8 117 521
8 344 706
33 515
32 725
11 531
22 996
21 461
17 248
17 269
25 316
21 612
26 956
25 740
Sites

Figure S15.19 Both species Split reads and trio assembly

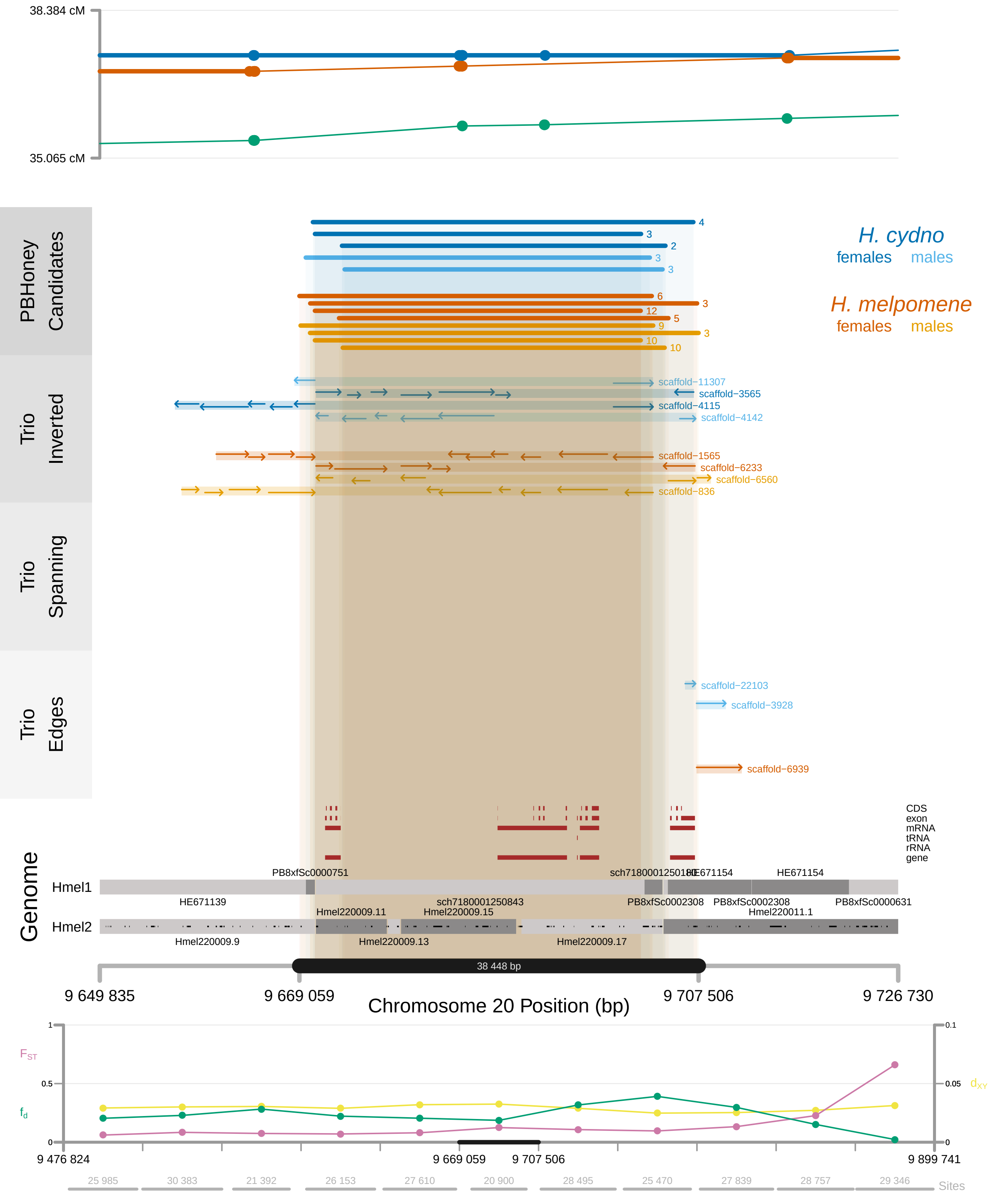

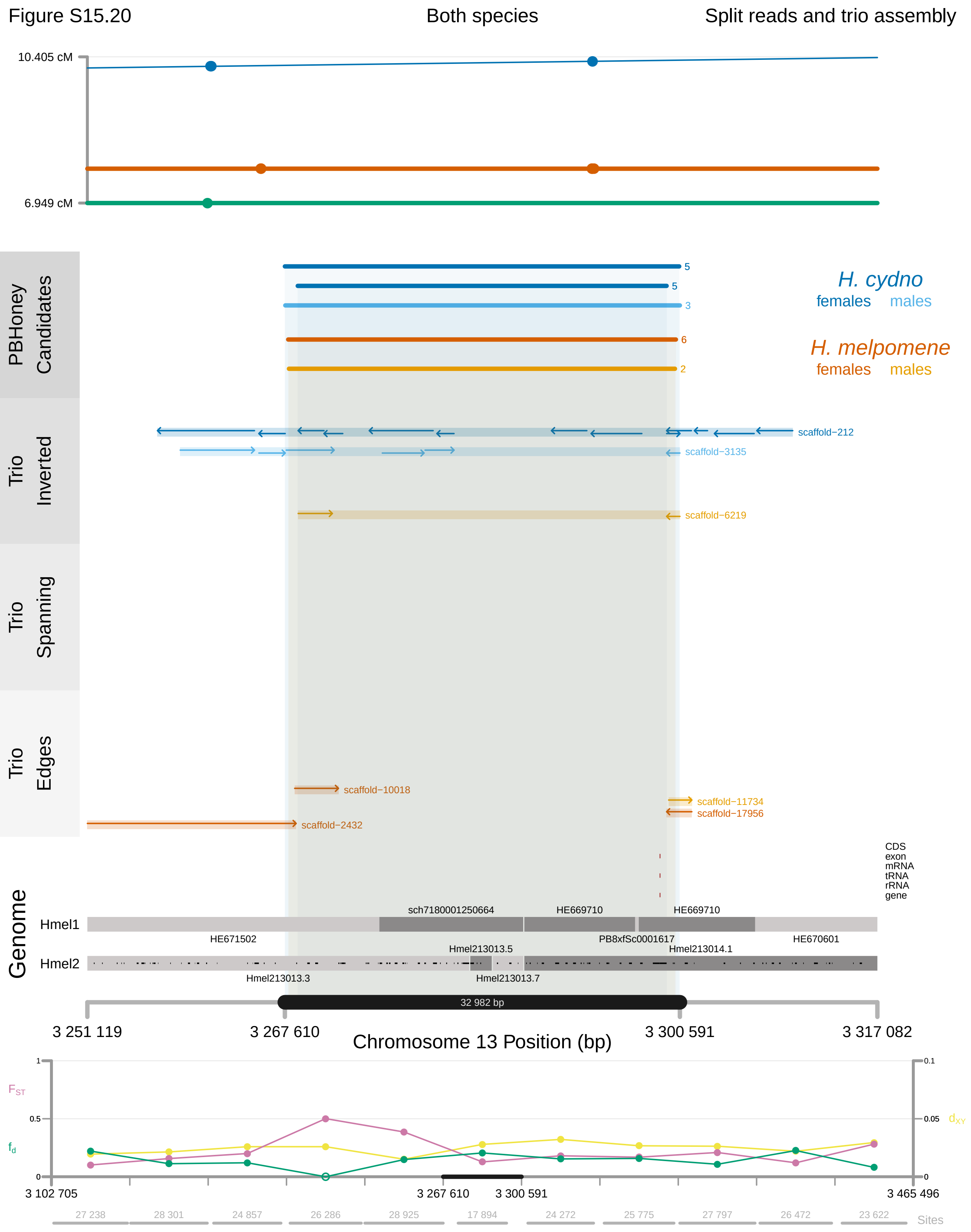

Figure S15.20
Both species
Split reads and trio assembly
10.405 cM
6.949 cM
PBHoney Candidates
5
5
3
6
2
H. cydno
females males
H. melpomene
females males
Trio Inverted
scaffold−212
scaffold−3135
scaffold−6219
Trio Spanning
Trio Edges
scaffold−10018
scaffold−11734
scaffold−17956
scaffold−2432
Genome
CDS
exon
mRNA
tRNA
rRNA
gene
Hmel1
sch7180001250664
HE669710
HE669710
HE671502
PB8xfSc0001617
HE670601
Hmel2
Hmel213013.5
Hmel213014.1
Hmel213013.3
Hmel213013.7
32 982 bp
3 251 119
3 267 610
3 300 591
3 317 082
Chromosome 13 Position (bp)
1
0.5
0
0.1
0.05
0
F_{ST}
f_d
d_{XY}
3 102 705
3 267 610
3 300 591
3 465 496
27 238
28 301
24 857
26 286
28 925
17 894
24 272
25 775
27 797
26 472
23 622
Sites

Figure S15.21 Both species Split reads and trio assembly

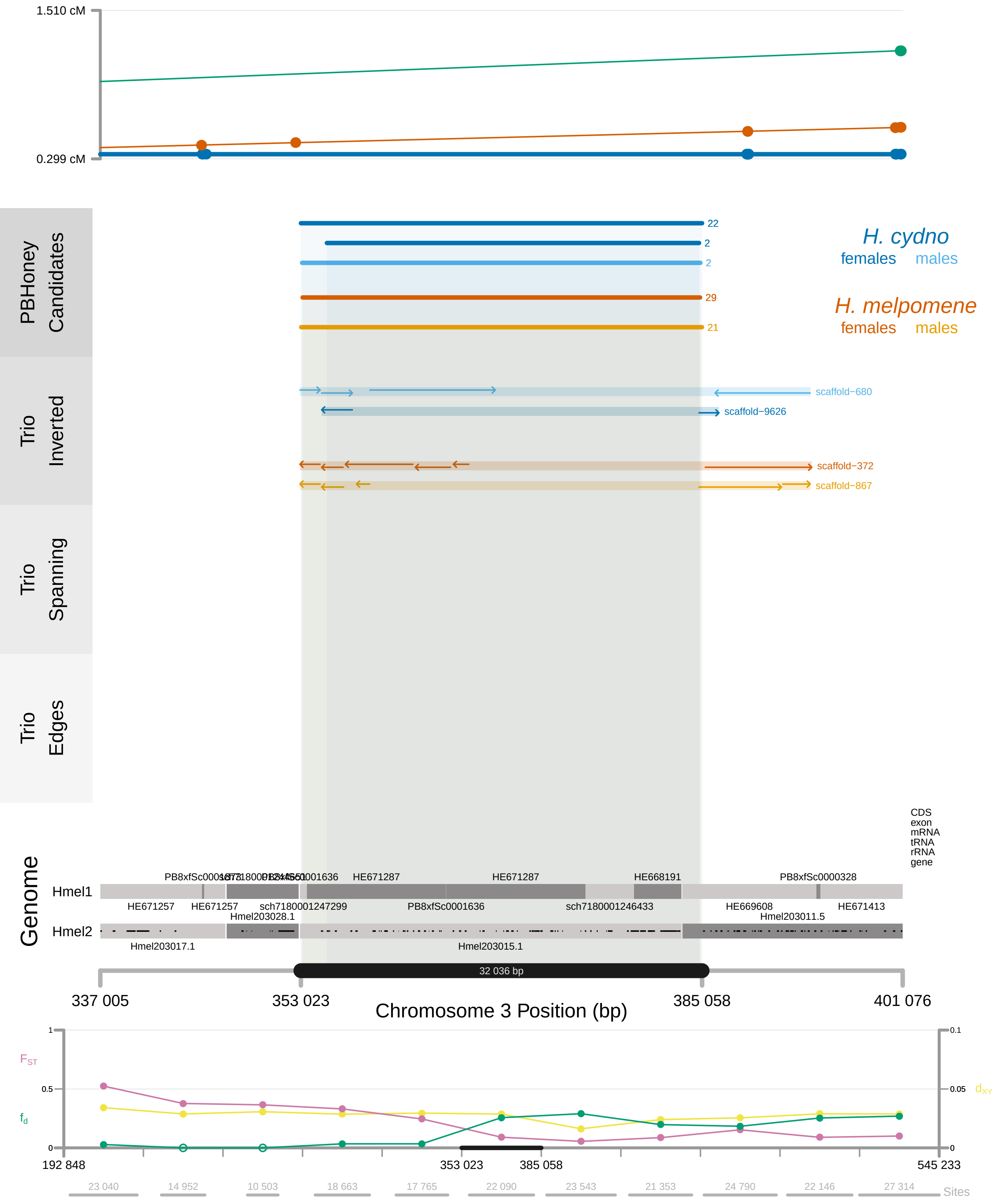

Figure S15.22

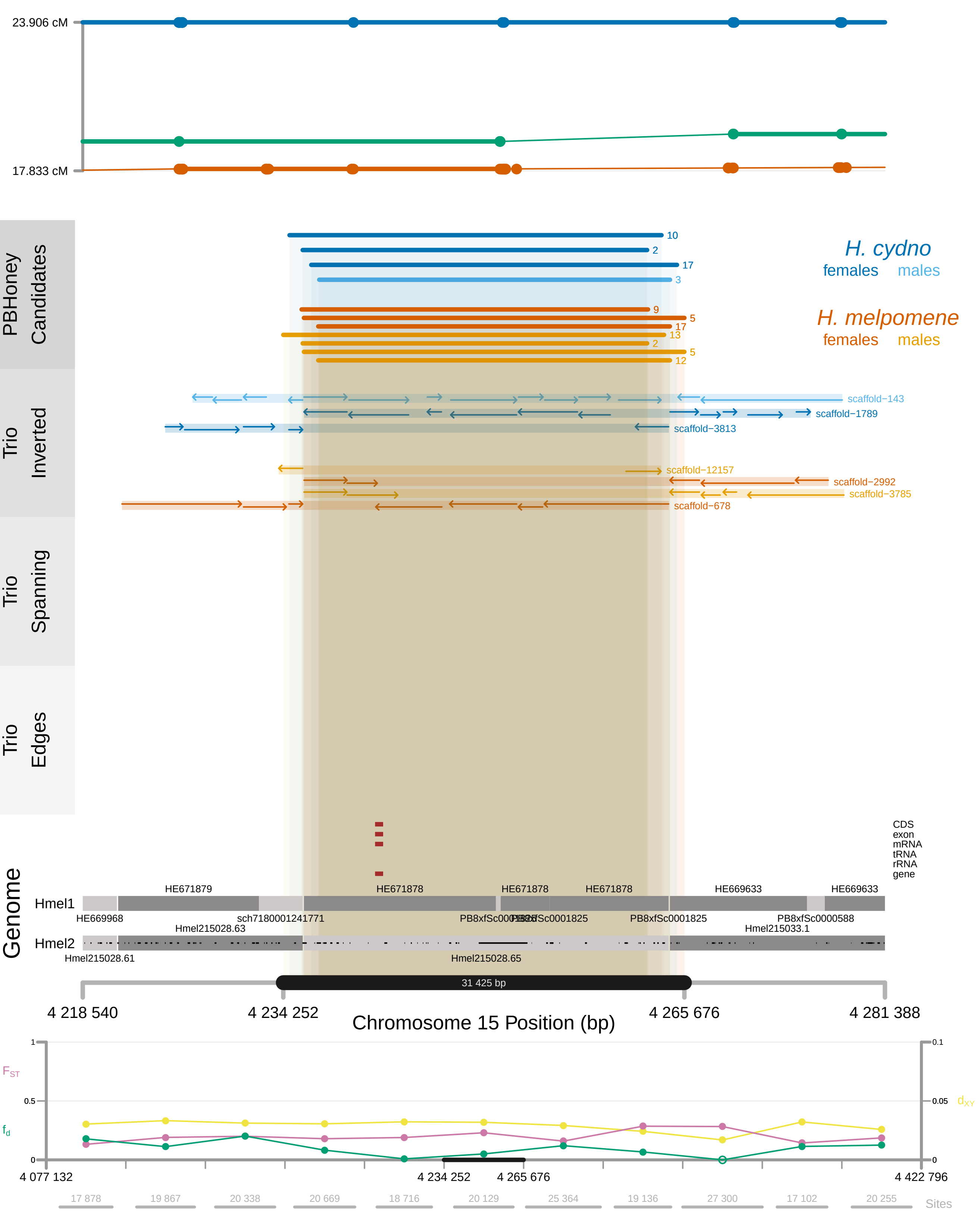

Figure S15.23

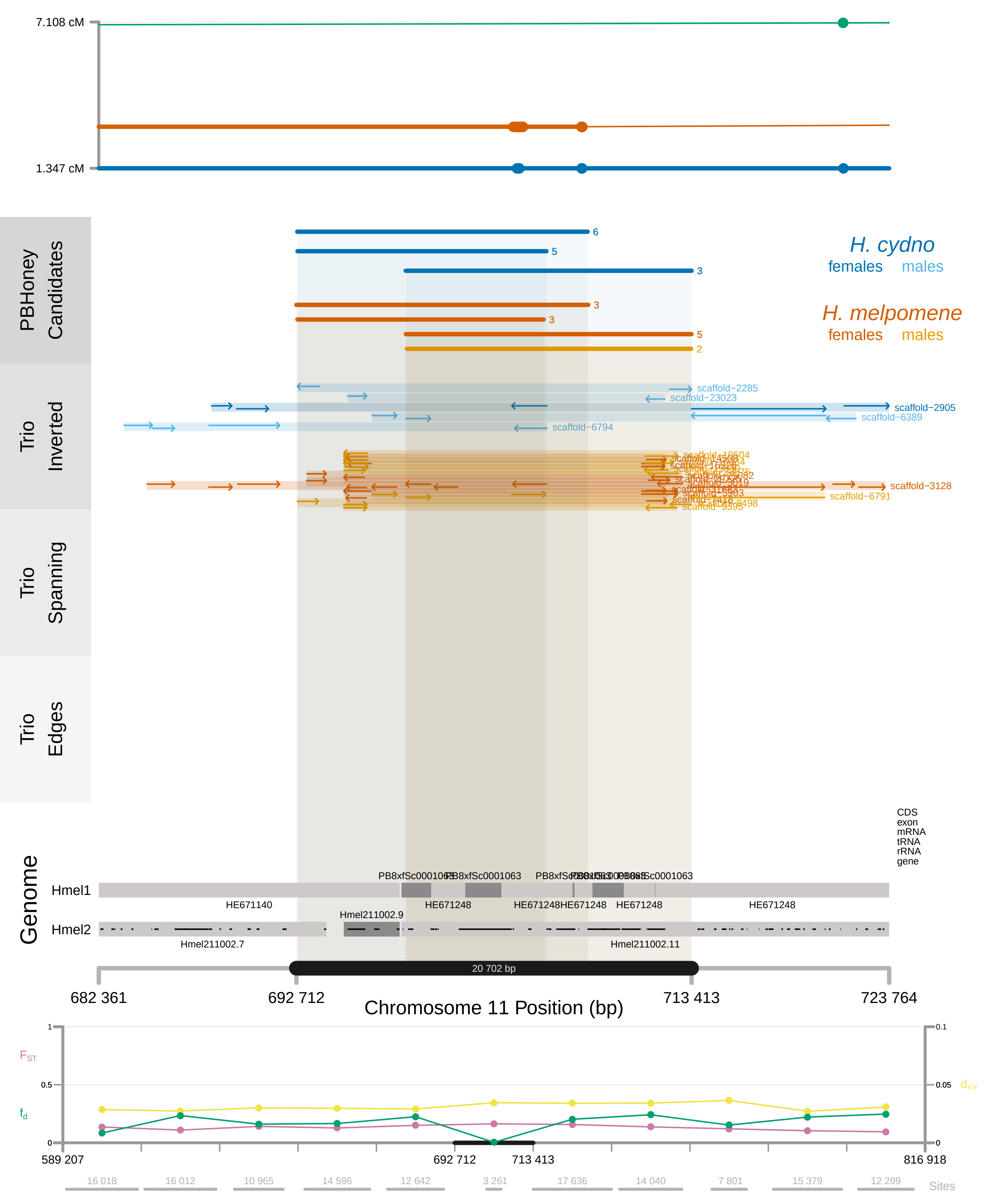

Figure S15.24

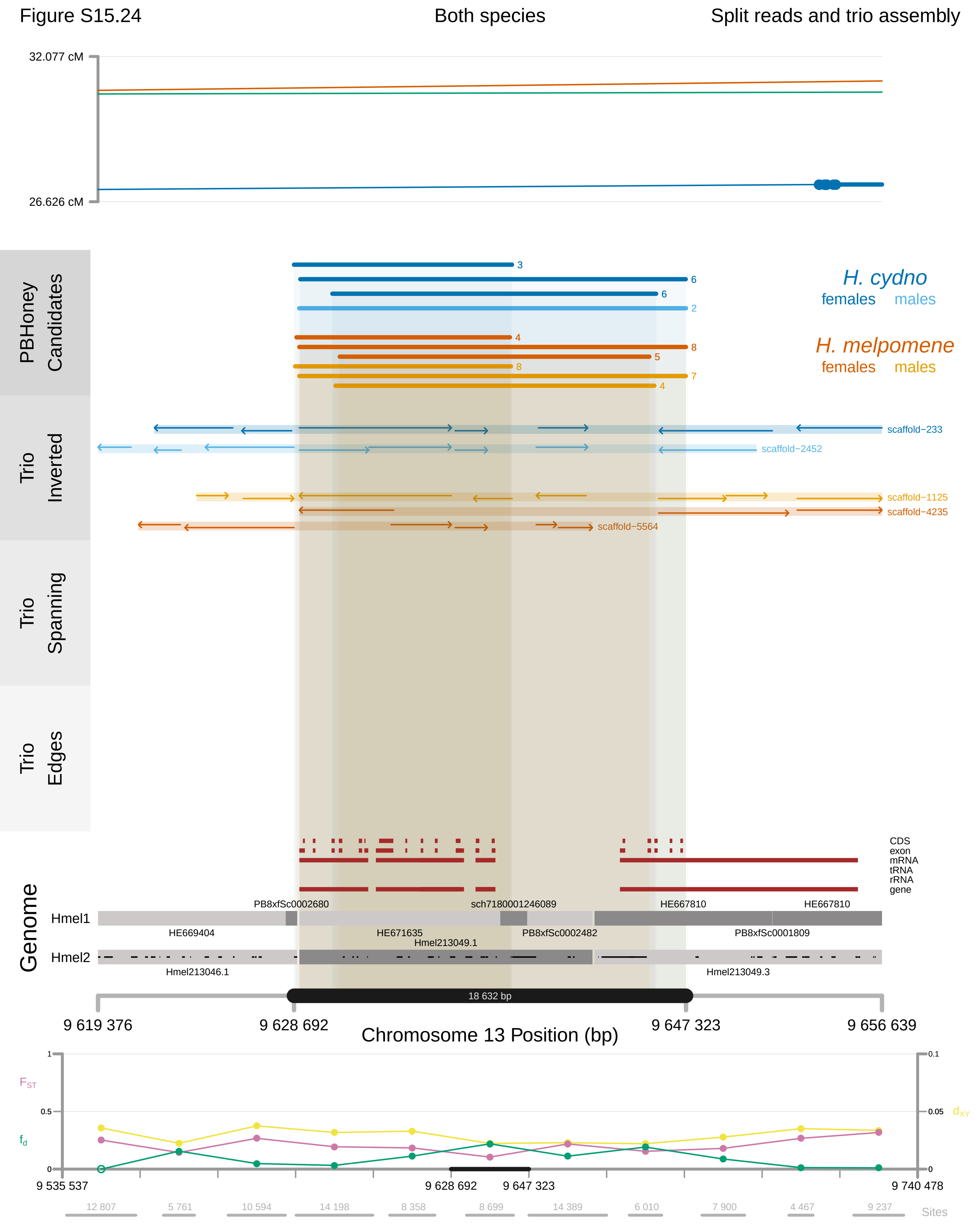

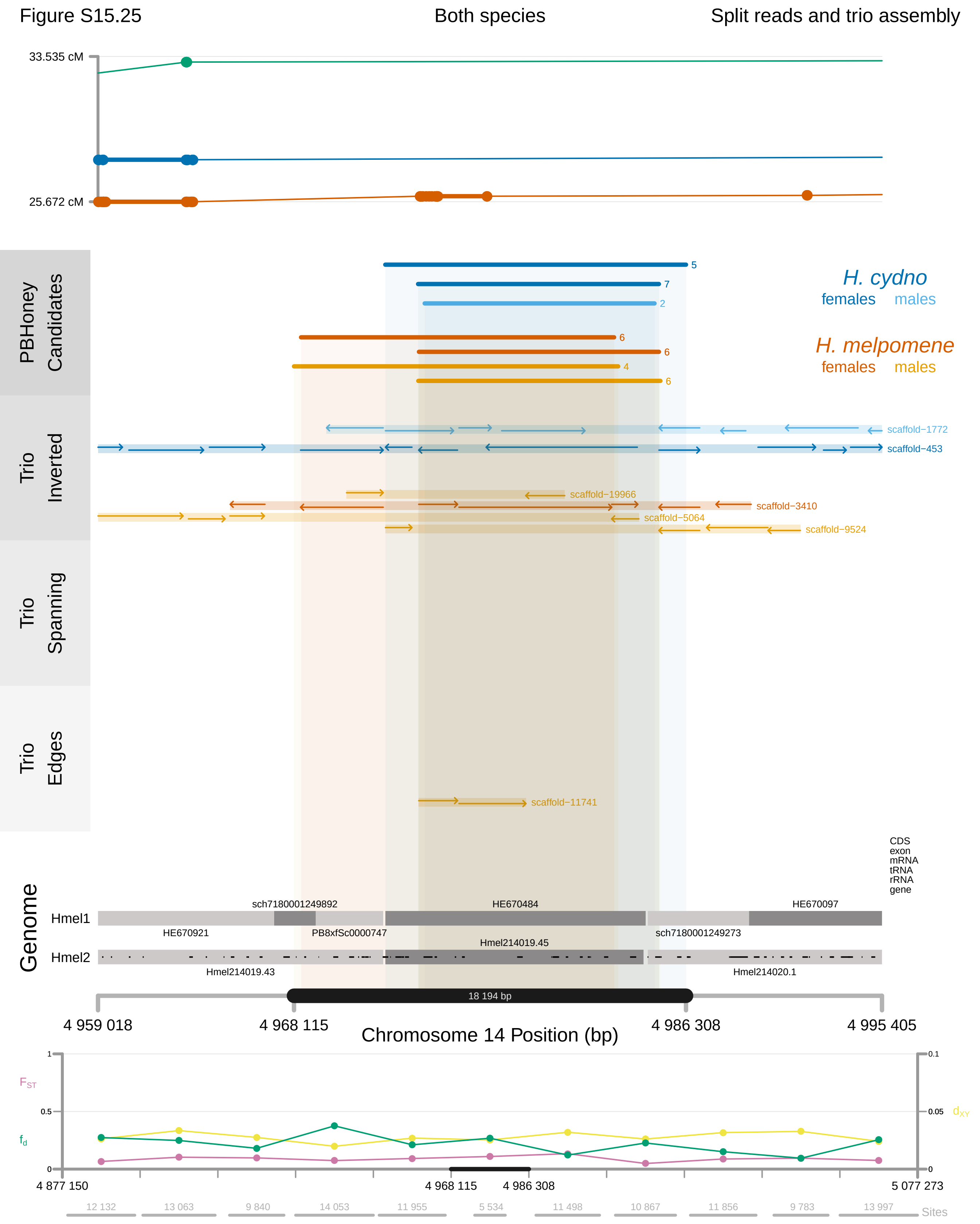

Figure S15.25
Both species
Split reads and trio assembly
33.535 cM
25.672 cM
PBHoney Candidates
Trio Inverted
Trio Spanning
Trio Edges
H. cydno
females males
H. melpomene
females males
scaffold−1772
scaffold−453
scaffold−19966
scaffold−3410
scaffold−5064
scaffold−9524
scaffold−11741
CDS
exon
mRNA
tRNA
rRNA
gene
Genome
Hmel1
Hmel2
sch7180001249892
HE670484
HE670097
HE670921
PB8xfSc0000747
sch7180001249273
Hmel214019.45
Hmel214019.43
Hmel214020.1
18 194 bp
4 959 018
4 968 115
4 986 308
4 995 405
Chromosome 14 Position (bp)
F_ST
f_d
d_XY
4 877 150
4 968 115
4 986 308
5 077 273
12 132
13 063
9 840
14 053
11 955
5 534
11 498
10 867
11 856
9 783
13 997
Sites

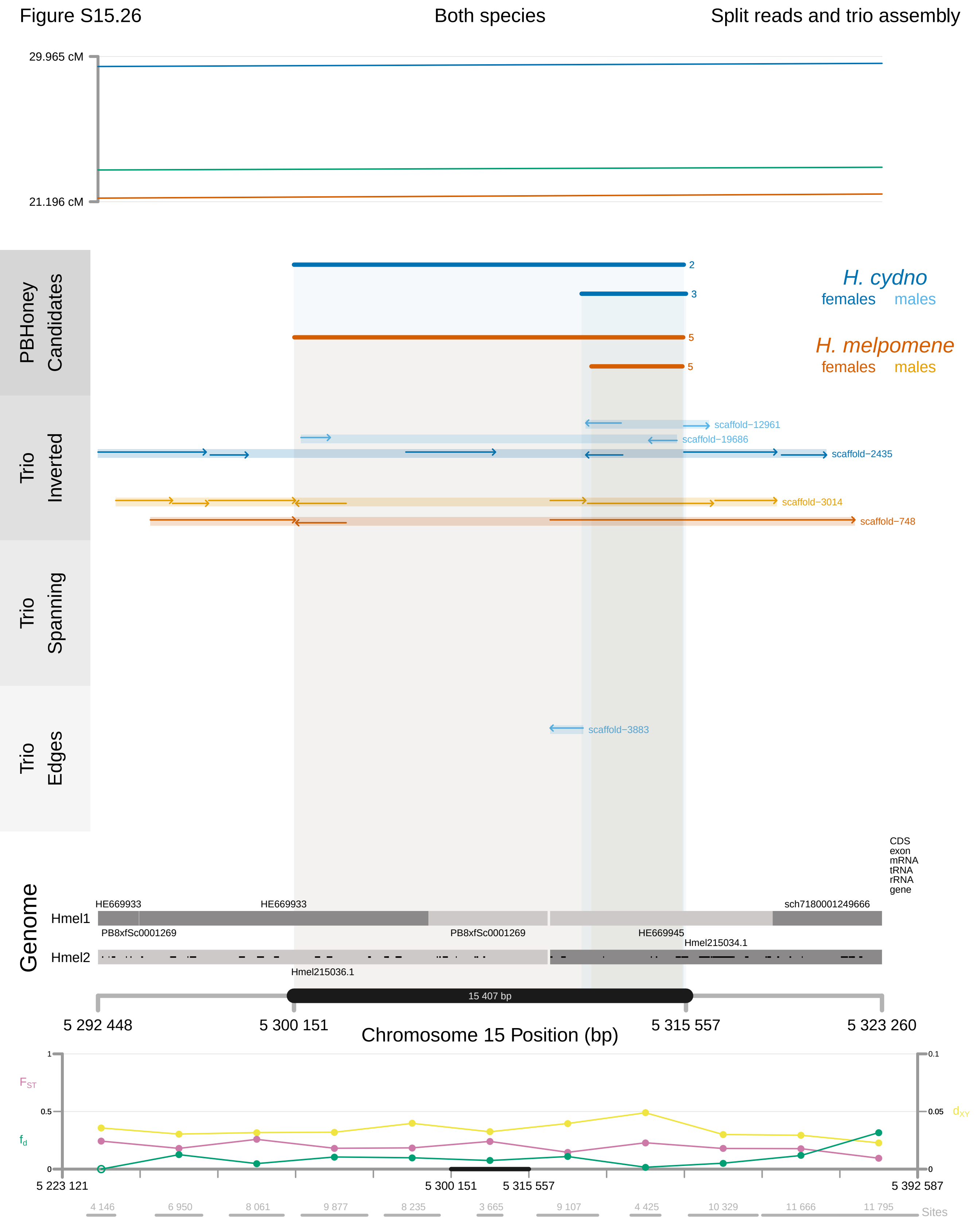

Figure S15.26
Both species
Split reads and trio assembly
29.965 cM
21.196 cM
PBHoney Candidates
Trio Inverted
Trio Spanning
Trio Edges
H. cydno
females males
H. melpomene
females males
scaffold−12961
scaffold−19686
scaffold−2435
scaffold−3014
scaffold−748
scaffold−3883
Genome
CDS
exon
mRNA
tRNA
rRNA
gene
Hmel1
Hmel2
HE669933
HE669933
sch7180001249666
PB8xfSc0001269
PB8xfSc0001269
HE669945
Hmel215034.1
Hmel215036.1
15 407 bp
5 292 448
5 300 151
5 315 557
5 323 260
Chromosome 15 Position (bp)
FST
fd
dXY
Sites
5 223 121
5 392 587
4 146
6 950
8 061
9 877
8 235
3 665
9 107
4 425
10 329
11 666
11 795

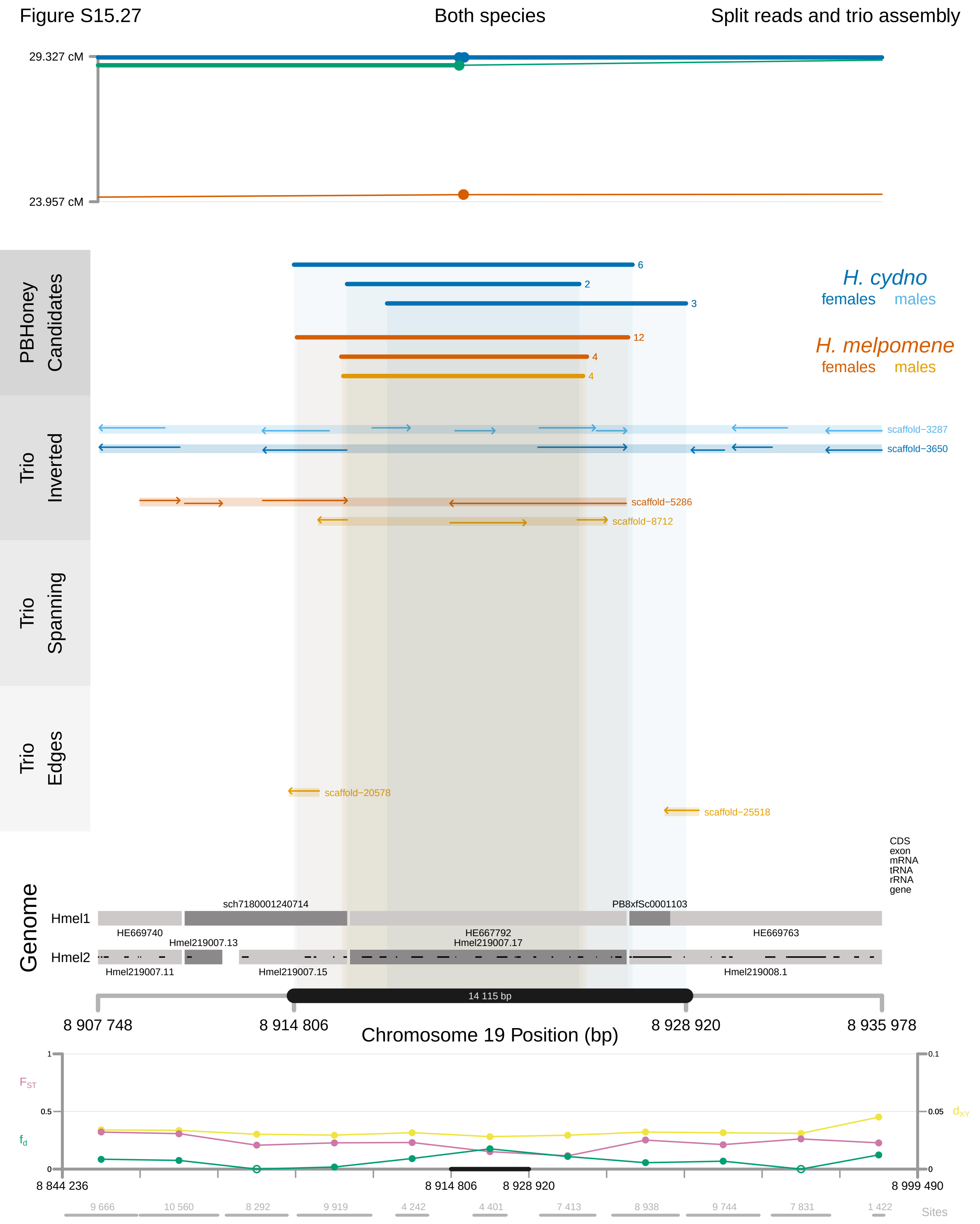

Figure S15.27
Both species
Split reads and trio assembly
29.327 cM
23.957 cM
PBHoney Candidates
6
2
3
12
4
4
H. cydno
females
males
H. melpomene
females
males
Trio Inverted
scaffold−3287
scaffold−3650
scaffold−5286
scaffold−8712
Trio Spanning
Trio Edges
scaffold−20578
scaffold−25518
CDS
exon
mRNA
tRNA
rRNA
gene
Genome
sch7180001240714
PB8xfSc0001103
Hmel1
HE669740
HE667792
HE669763
Hmel219007.13
Hmel219007.17
Hmel2
Hmel219007.11
Hmel219007.15
Hmel219008.1
14 115 bp
8 907 748
8 914 806
8 928 920
8 935 978
Chromosome 19 Position (bp)
1
0.5
0
0.1
0.05
0
F_{ST}
f_d
d_{XY}
8 844 236
8 914 806
8 928 920
8 999 490
9 666
10 560
8 292
9 919
4 242
4 401
7 413
8 938
9 744
7 831
1 422
Sites

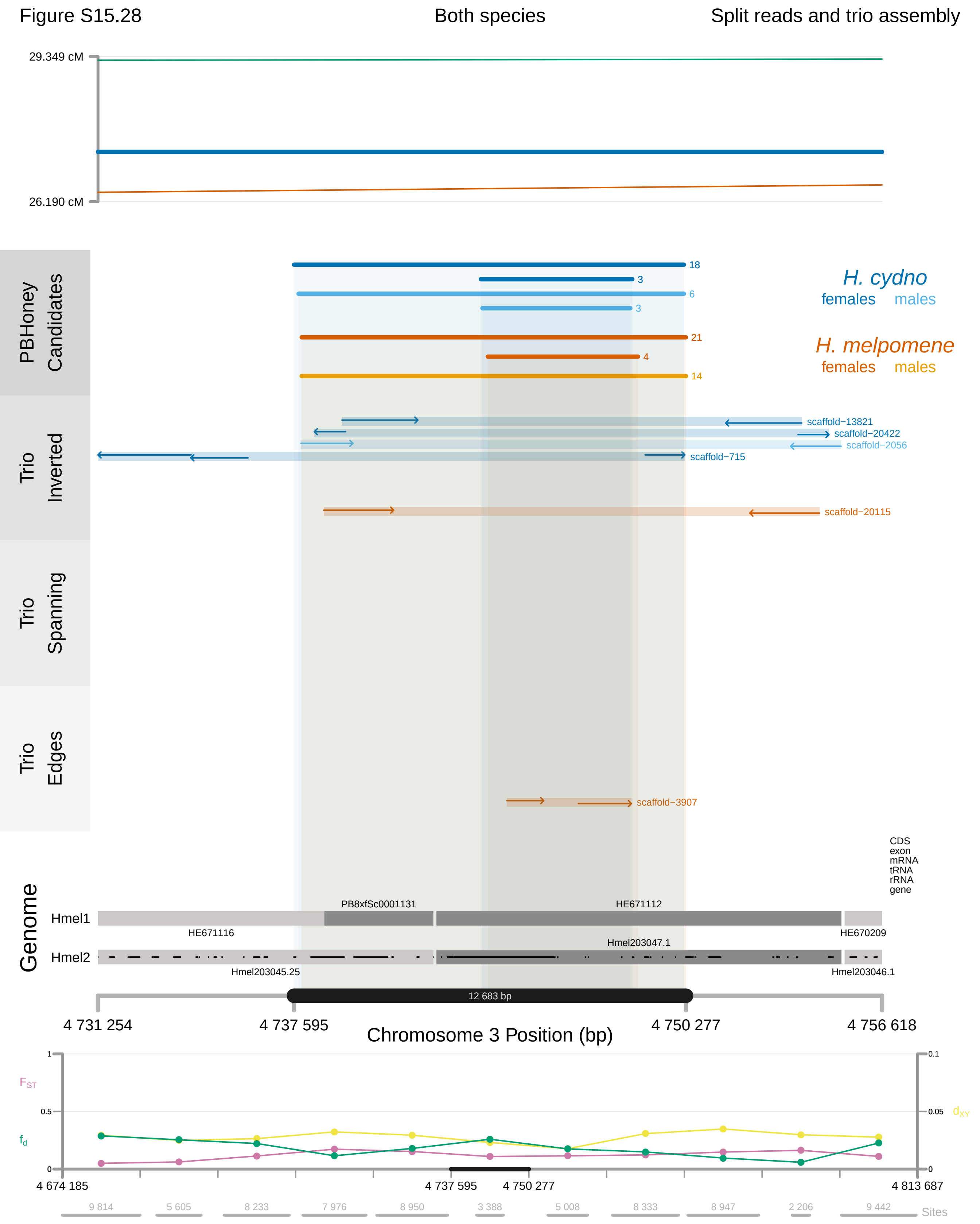
Figure S15.28
Both species
Split reads and trio assembly
29.349 cM
26.190 cM
PBHoney Candidates
Trio Inverted
Trio Spanning
Trio Edges
Genome
18
3
6
3
21
4
14
H. cydno
females males
H. melpomene
females males
scaffold−13821
scaffold−20422
scaffold−2056
scaffold−715
scaffold−20115
scaffold−3907
CDS
exon
mRNA
tRNA
rRNA
gene
Hmel1
Hmel2
PB8xfSc0001131
HE671112
HE671116
HE670209
Hmel203047.1
Hmel203045.25
Hmel203046.1
12 683 bp
4 731 254
4 737 595
4 750 277
4 756 618
Chromosome 3 Position (bp)
FST
fd
dXY
1
0.5
0
0.1
0.05
0
4 674 185
4 737 595
4 750 277
4 813 687
9 814
5 605
8 233
7 976
8 950
3 388
5 008
8 333
8 947
2 206
9 442
Sites

Figure S15.29 Both species Split reads and trio assembly

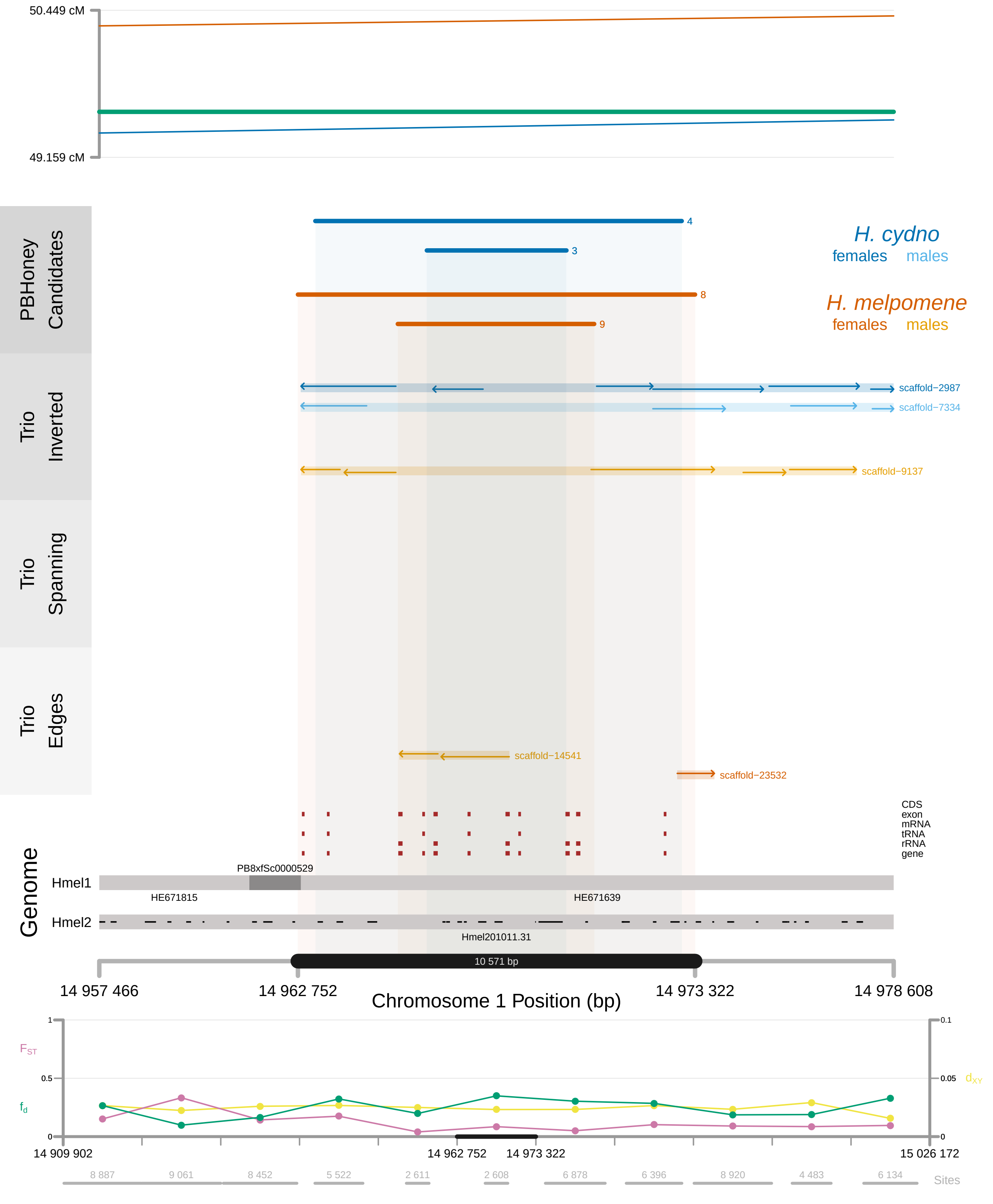

Figure S15.30 Both species Split reads and trio assembly

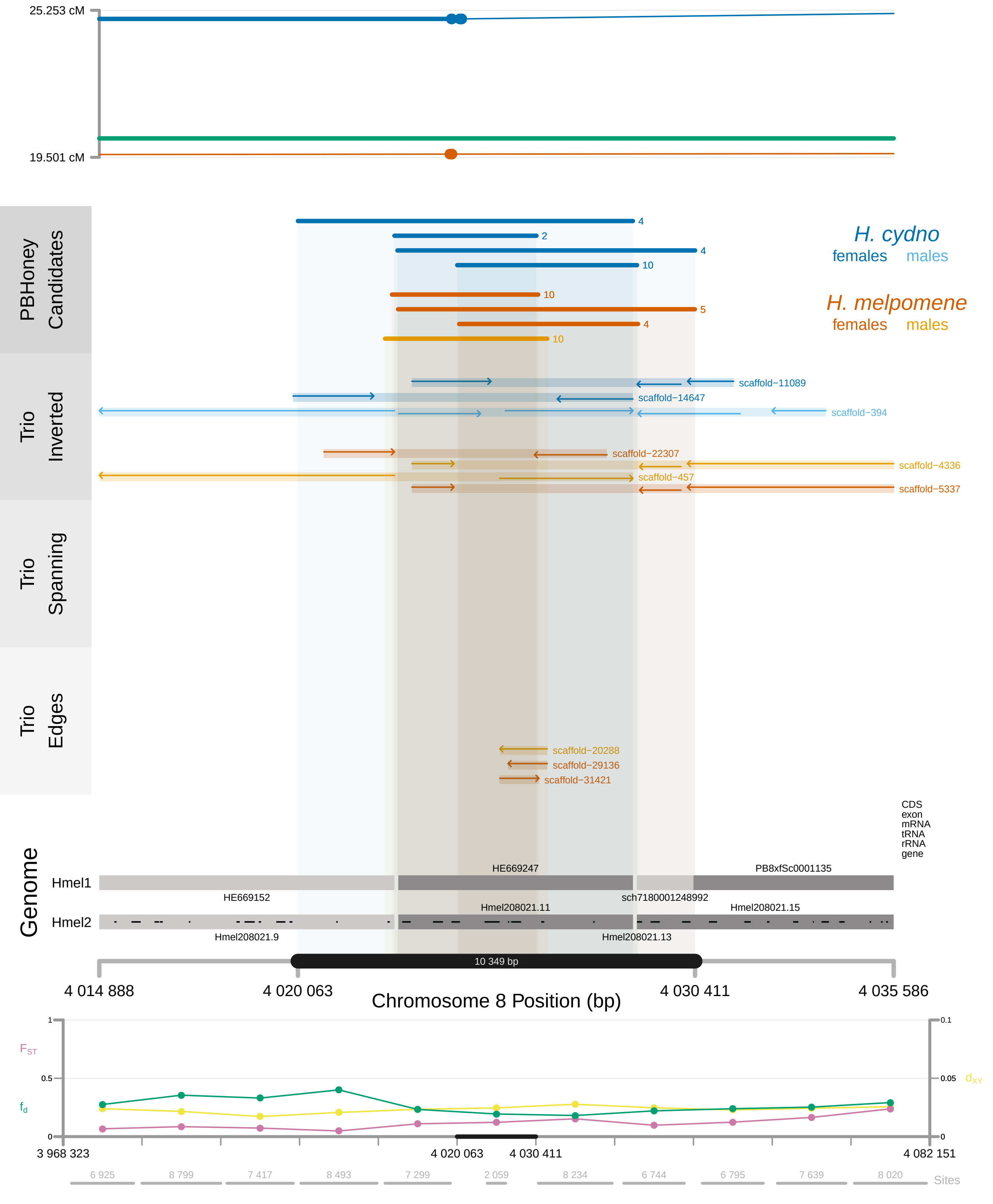

Figure S15.31

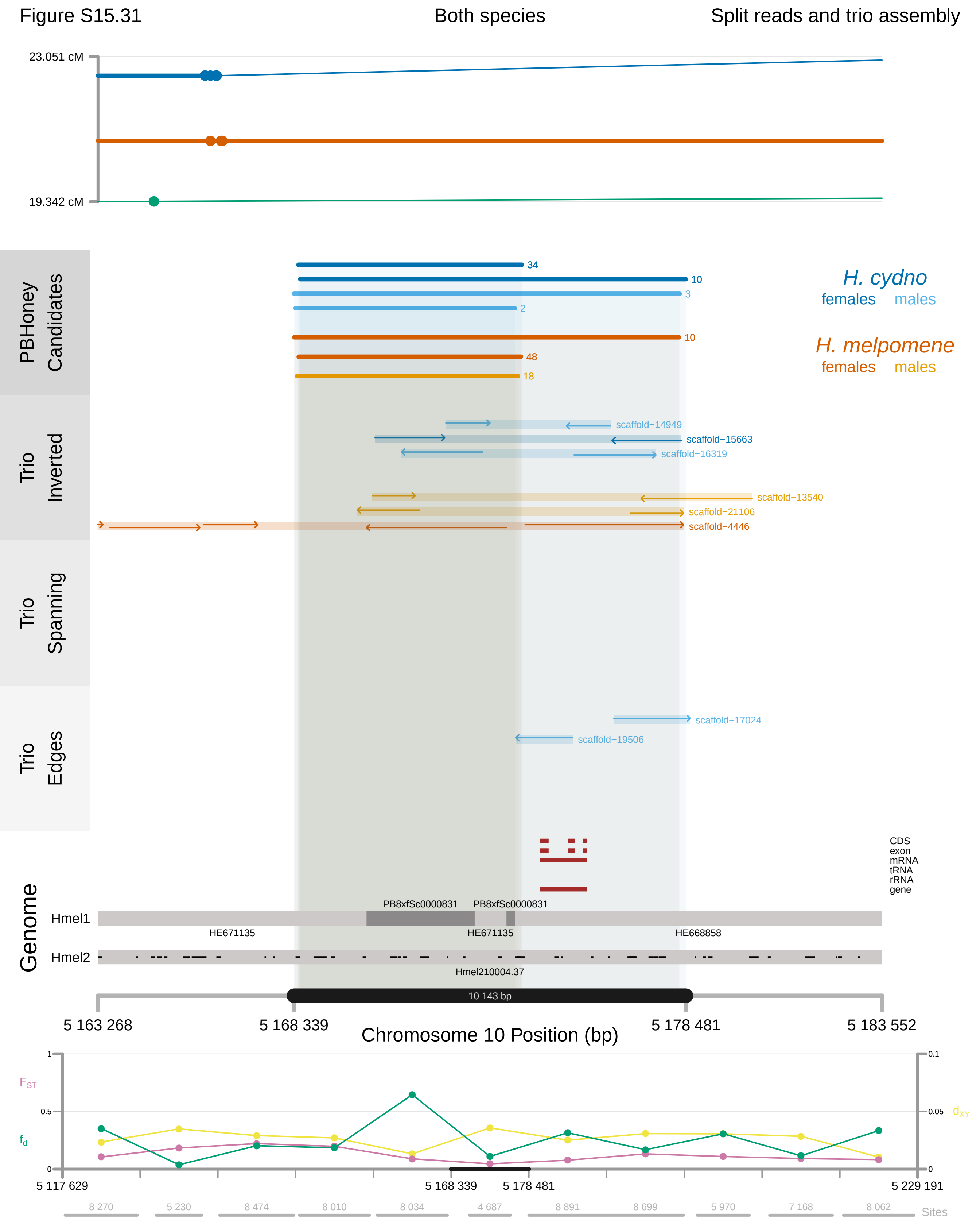

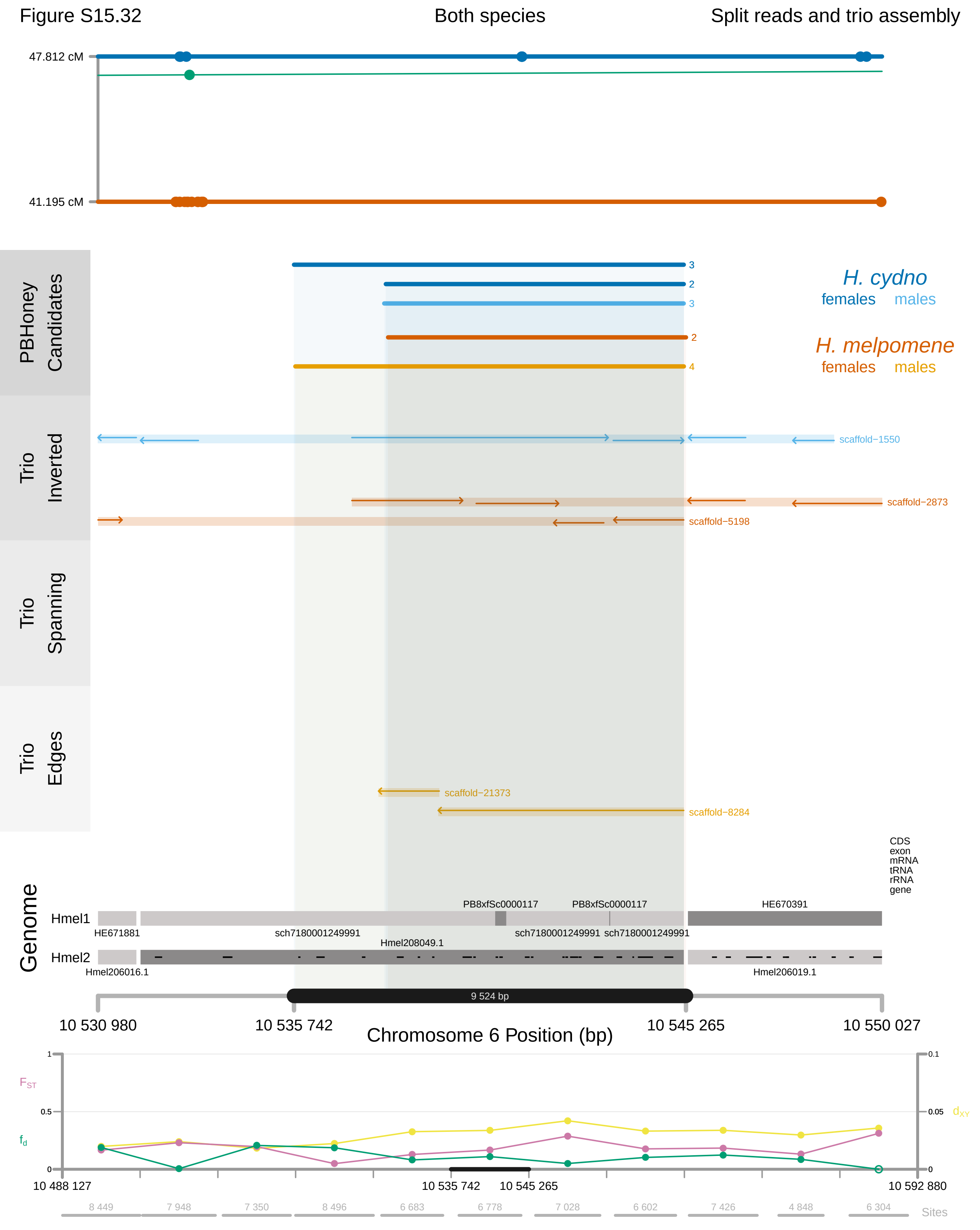

Figure S15.32
Both species
Split reads and trio assembly
47.812 cM
41.195 cM
PBHoney Candidates
Trio Inverted
Trio Spanning
Trio Edges
Genome
H. cydno
females
males
H. melpomene
females
males
scaffold−1550
scaffold−2873
scaffold−5198
scaffold−21373
scaffold−8284
CDS
exon
mRNA
tRNA
rRNA
gene
Hmel1
Hmel2
PB8xfSc0000117
PB8xfSc0000117
HE670391
HE671881
sch7180001249991
sch7180001249991
sch7180001249991
Hmel208049.1
Hmel206016.1
Hmel206019.1
9 524 bp
10 530 980
10 535 742
10 545 265
10 550 027
Chromosome 6 Position (bp)
F_ST
f_d
d_XY
10 488 127
10 535 742
10 545 265
10 592 880
8 449
7 948
7 350
8 496
6 683
6 778
7 028
6 602
7 426
4 848
6 304
Sites

Figure S15.33 Both species Split reads and trio assembly

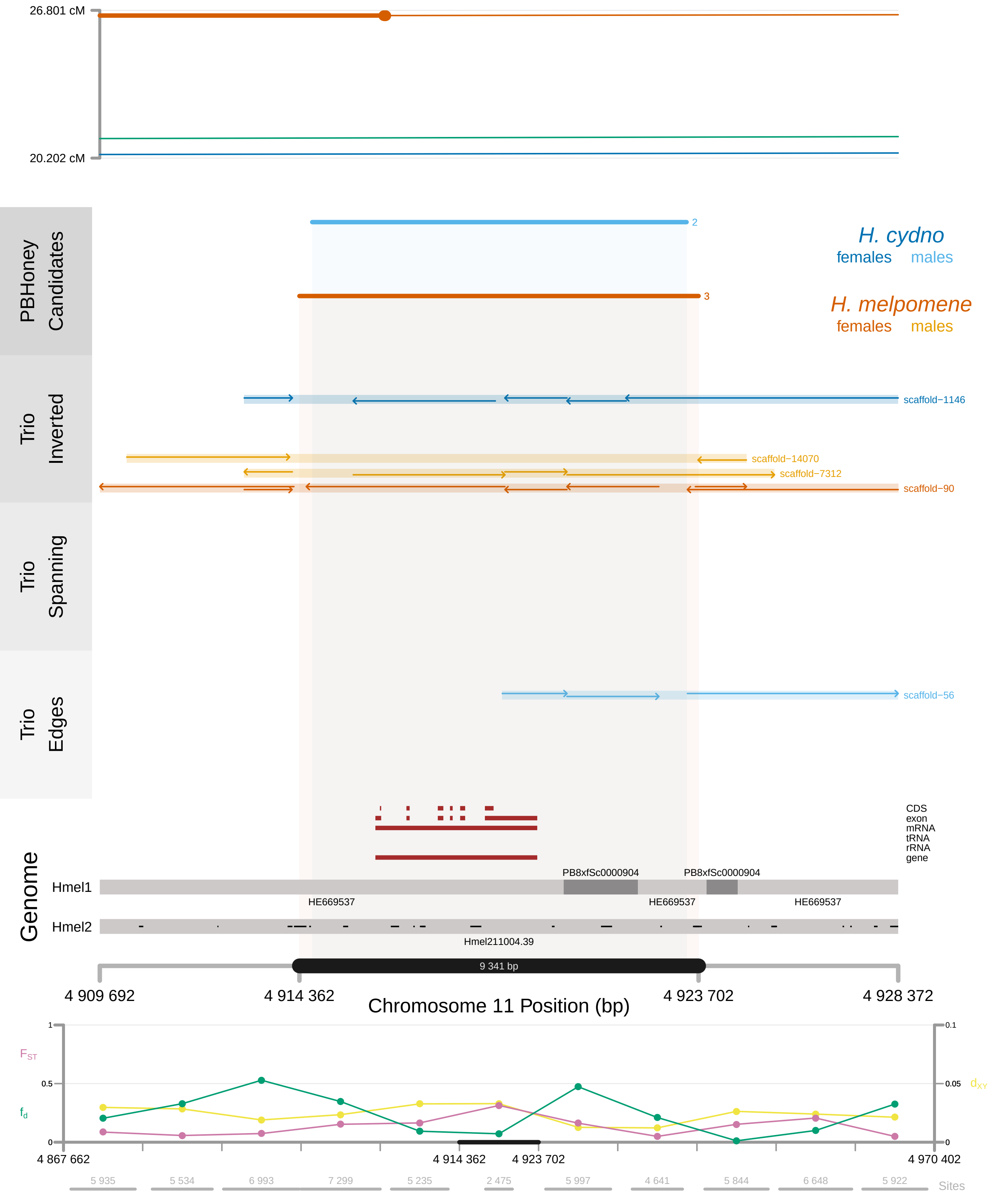

Figure S15.34 Both species Split reads and trio assembly

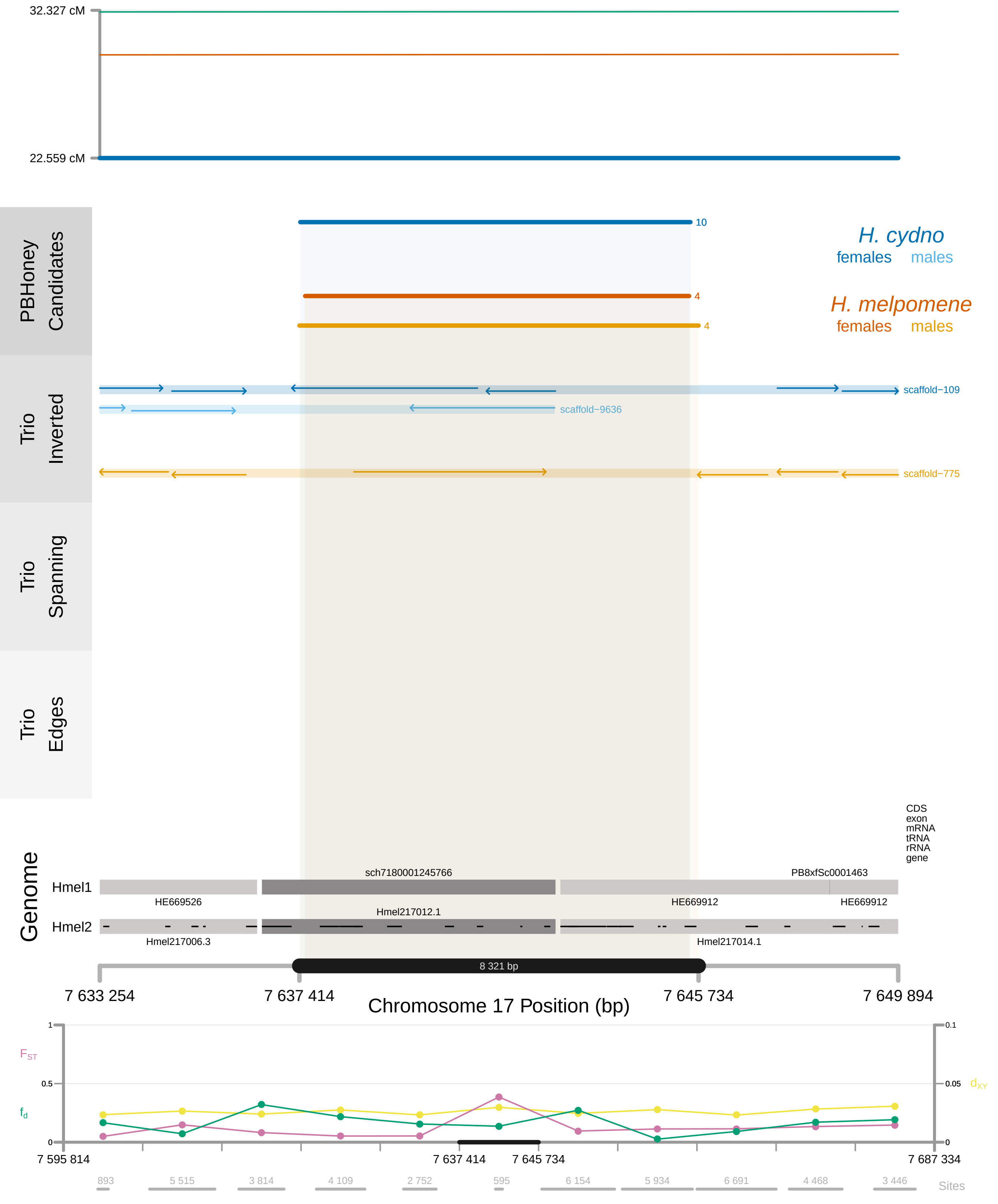

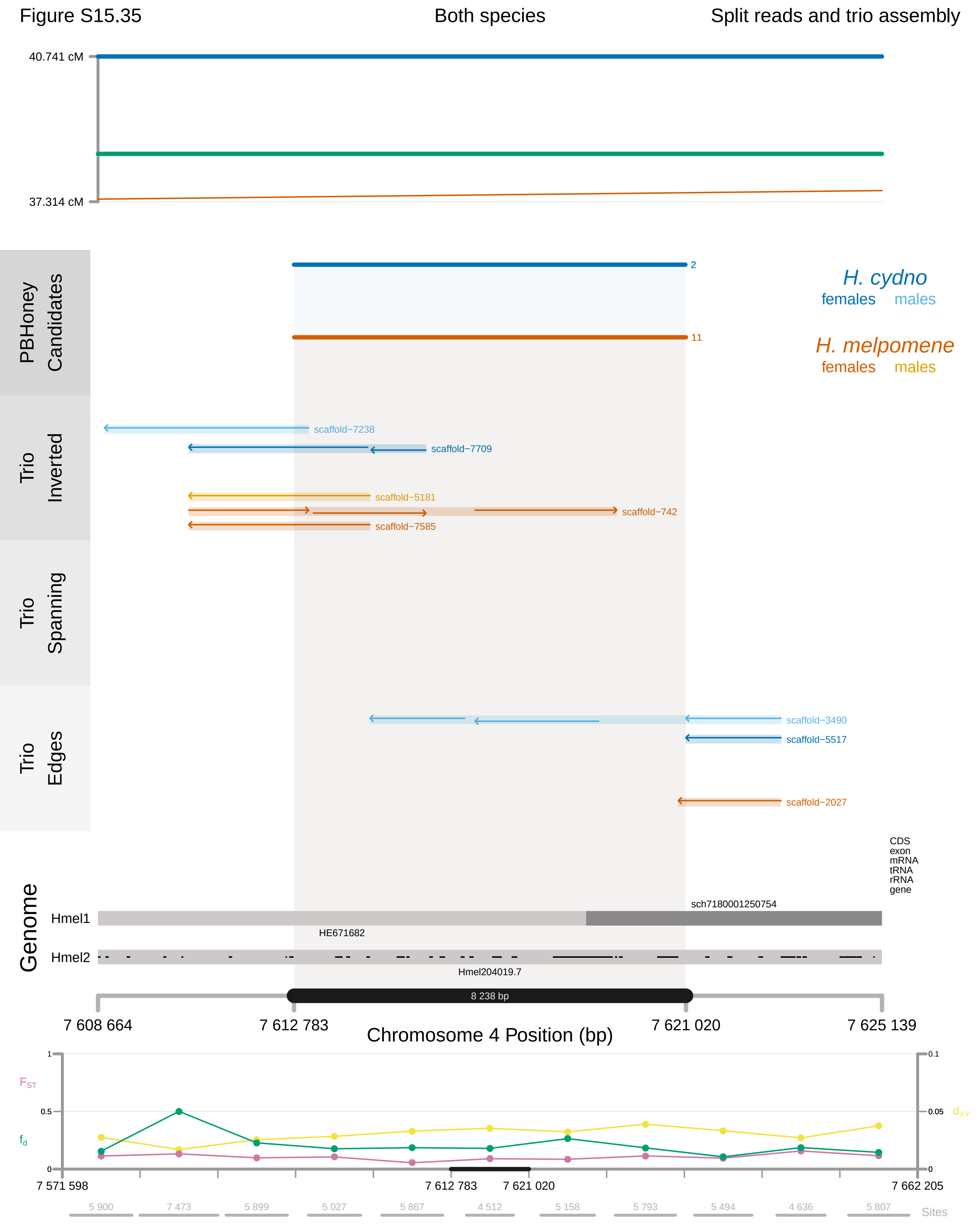

Figure S15.35
Both species
Split reads and trio assembly
40.741 cM
37.314 cM
PBHoney Candidates
Trio Inverted
Trio Spanning
Trio Edges
Genome
2
11
H. cydno
females males
H. melpomene
females males
scaffold−7238
scaffold−7709
scaffold−5181
scaffold−742
scaffold−7585
scaffold−3490
scaffold−5517
scaffold−2027
CDS
exon
mRNA
tRNA
rRNA
gene
sch7180001250754
Hmel1
HE671682
Hmel2
Hmel204019.7
8 238 bp
7 608 664
7 612 783
7 621 020
7 625 139
Chromosome 4 Position (bp)
1
0.5
0
F_{ST}
f_d
0.1
0.05
0
d_{XY}
7 571 598
7 612 783
7 621 020
7 662 205
5 900
7 473
5 899
5 027
5 867
4 512
5 158
5 793
5 494
4 636
5 807
Sites

Figure S15.36 Both species Split reads and trio assembly

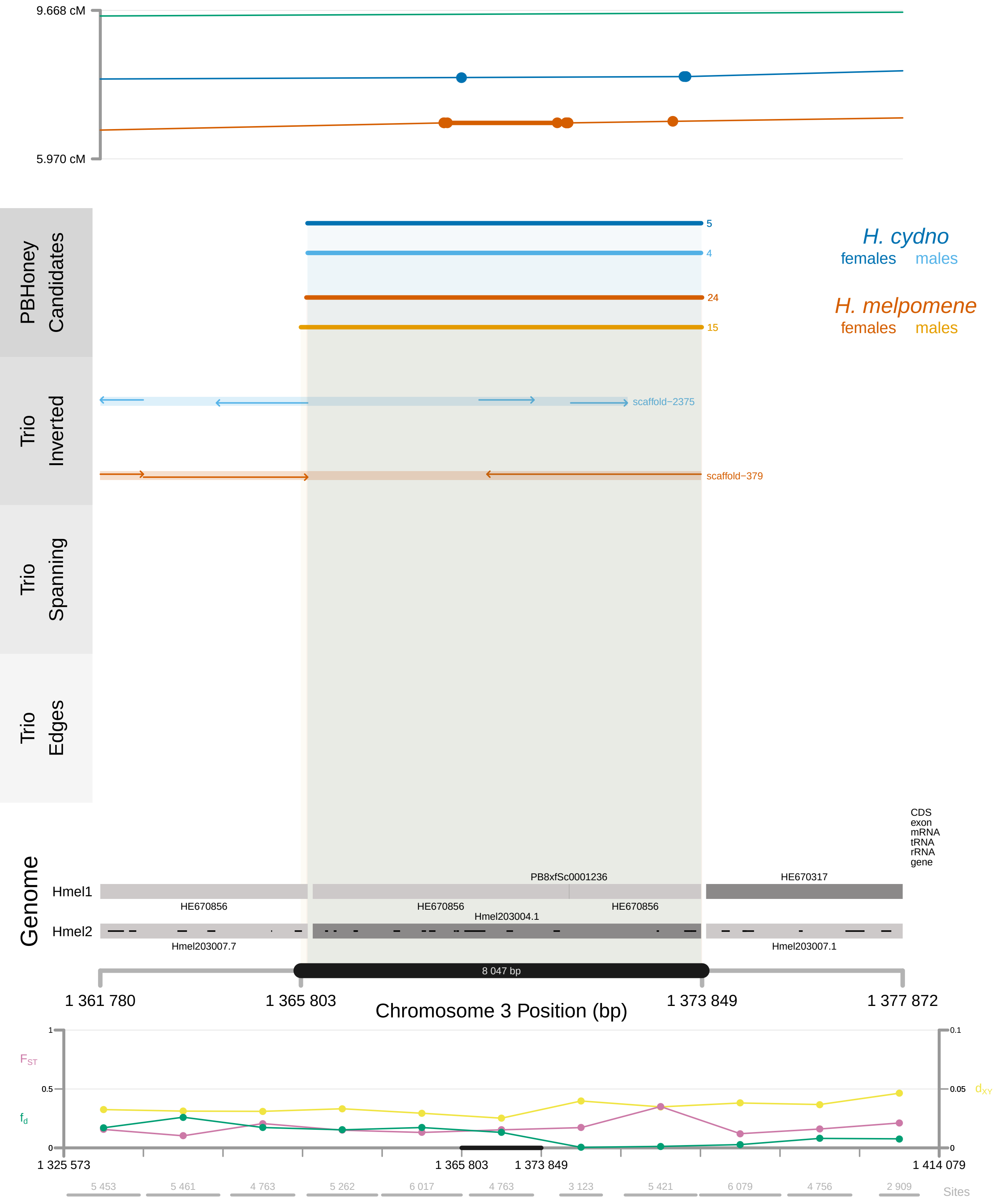

Figure S15.37 Both species Split reads and trio assembly

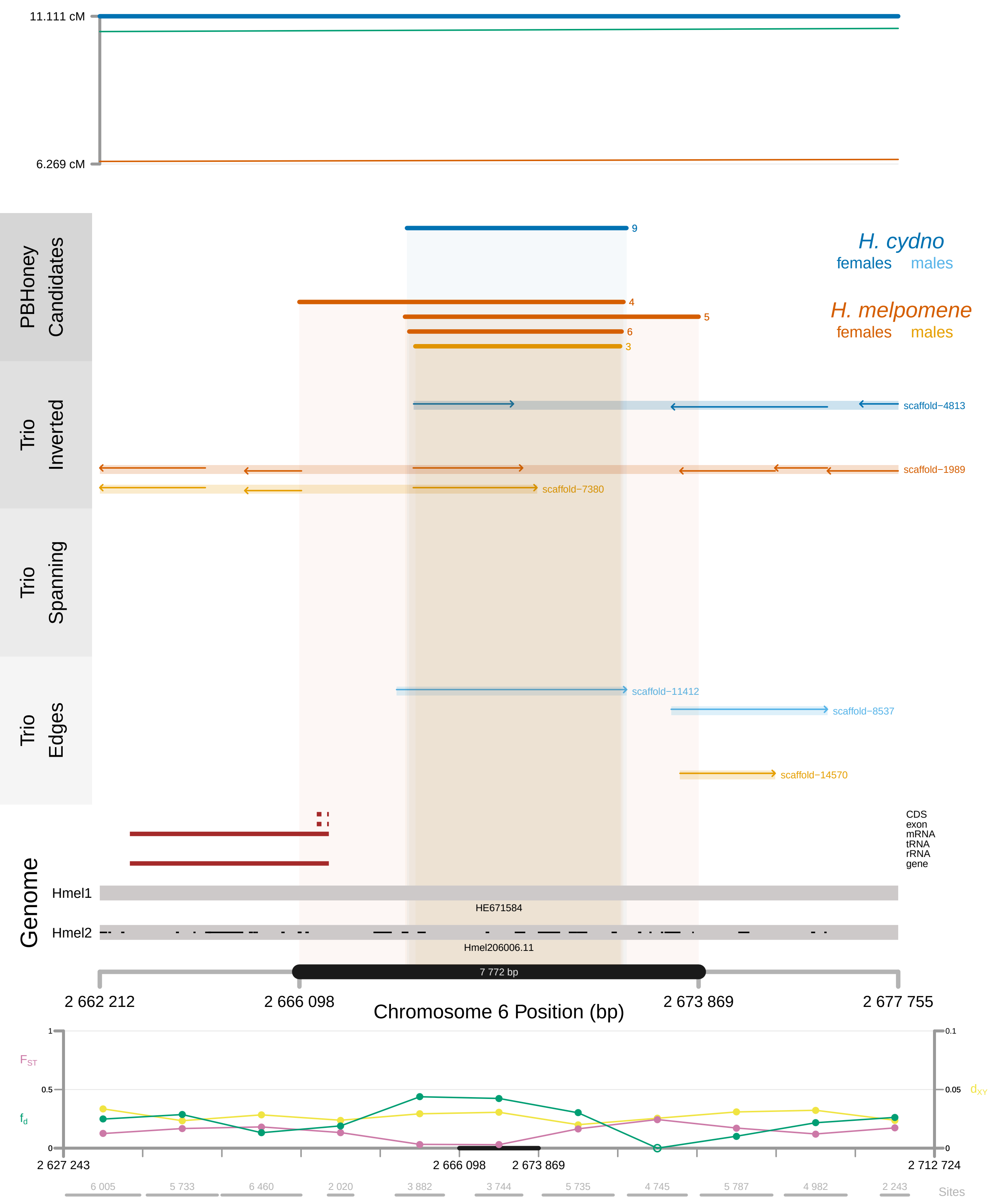

Figure S15.38 Both species Split reads and trio assembly

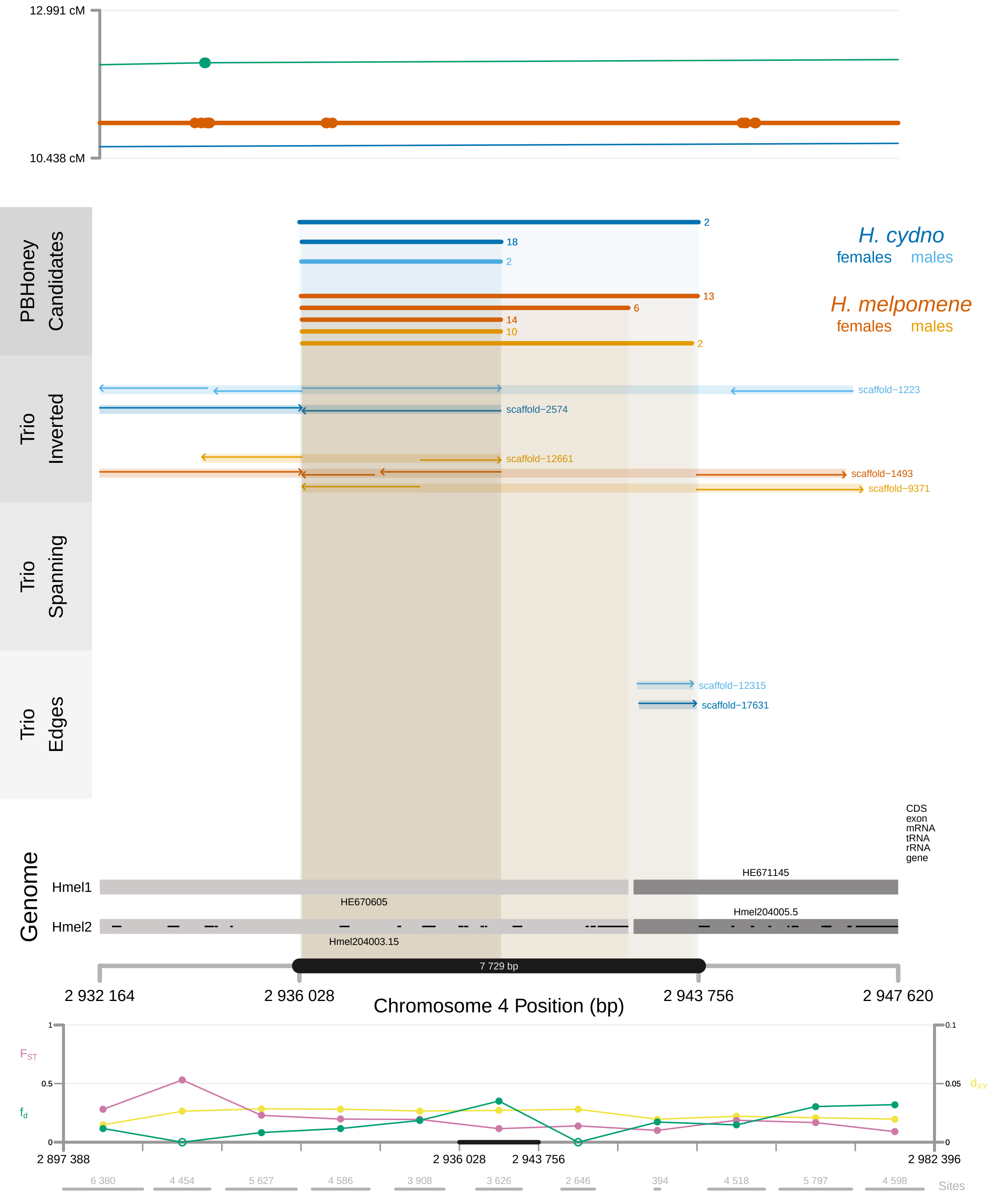

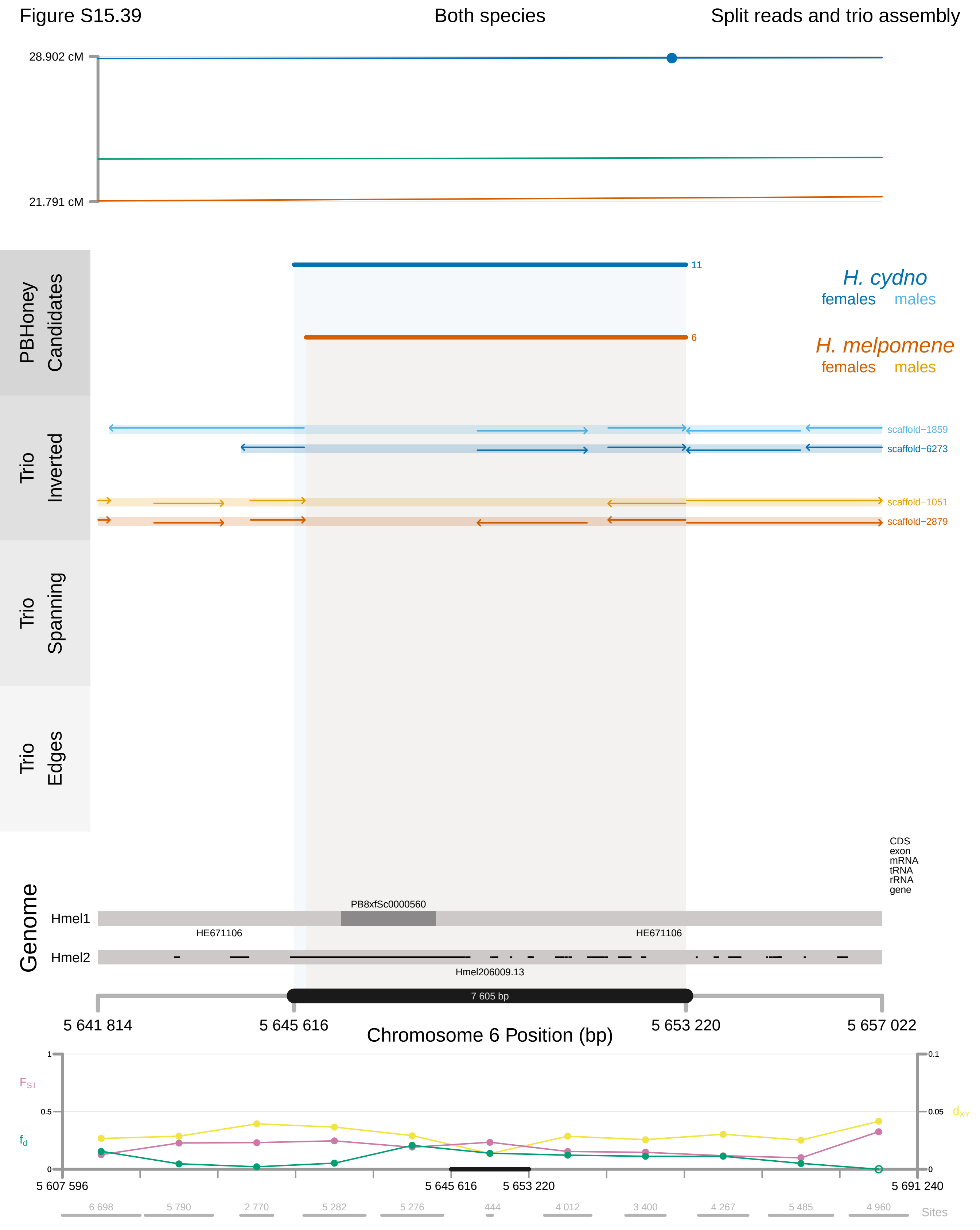

Figure S15.39
Both species
Split reads and trio assembly
28.902 cM
21.791 cM
PBHoney Candidates
11
6
H. cydno
females
males
H. melpomene
females
males
Trio Inverted
scaffold−1859
scaffold−6273
scaffold−1051
scaffold−2879
Trio Spanning
Trio Edges
Genome
CDS
exon
mRNA
tRNA
rRNA
gene
PB8xfSc0000560
Hmel1
HE671106
HE671106
Hmel2
Hmel206009.13
7 605 bp
5 641 814
5 645 616
5 653 220
5 657 022
Chromosome 6 Position (bp)
FST
fd
dXY
1
0.5
0
0.1
0.05
0
5 607 596
5 645 616
5 653 220
5 691 240
6 698
5 790
2 770
5 282
5 276
444
4 012
3 400
4 267
5 485
4 960
Sites

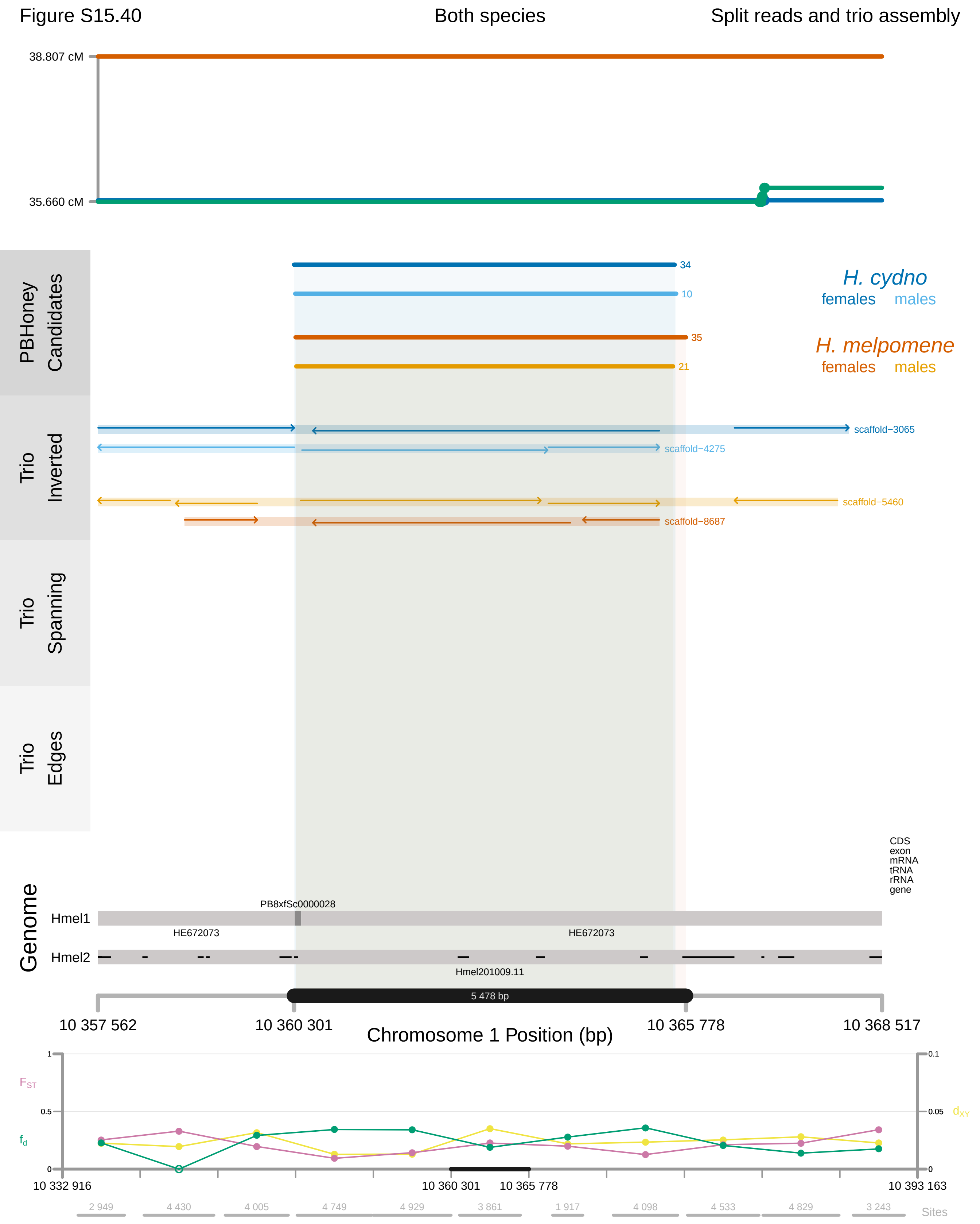

Figure S15.40
Both species
Split reads and trio assembly
38.807 cM
35.660 cM
PBHoney Candidates
34
10
35
21
H. cydno
females
males
H. melpomene
females
males
Trio Inverted
scaffold−3065
scaffold−4275
scaffold−5460
scaffold−8687
Trio Spanning
Trio Edges
Genome
CDS
exon
mRNA
tRNA
rRNA
gene
Hmel1
PB8xfSc0000028
HE672073
HE672073
Hmel2
Hmel201009.11
5 478 bp
10 357 562
10 360 301
10 365 778
10 368 517
Chromosome 1 Position (bp)
FST
fd
dXY
1
0.5
0
0.1
0.05
0
10 332 916
10 360 301
10 365 778
10 393 163
2 949
4 430
4 005
4 749
4 929
3 861
1 917
4 098
4 533
4 829
3 243
Sites

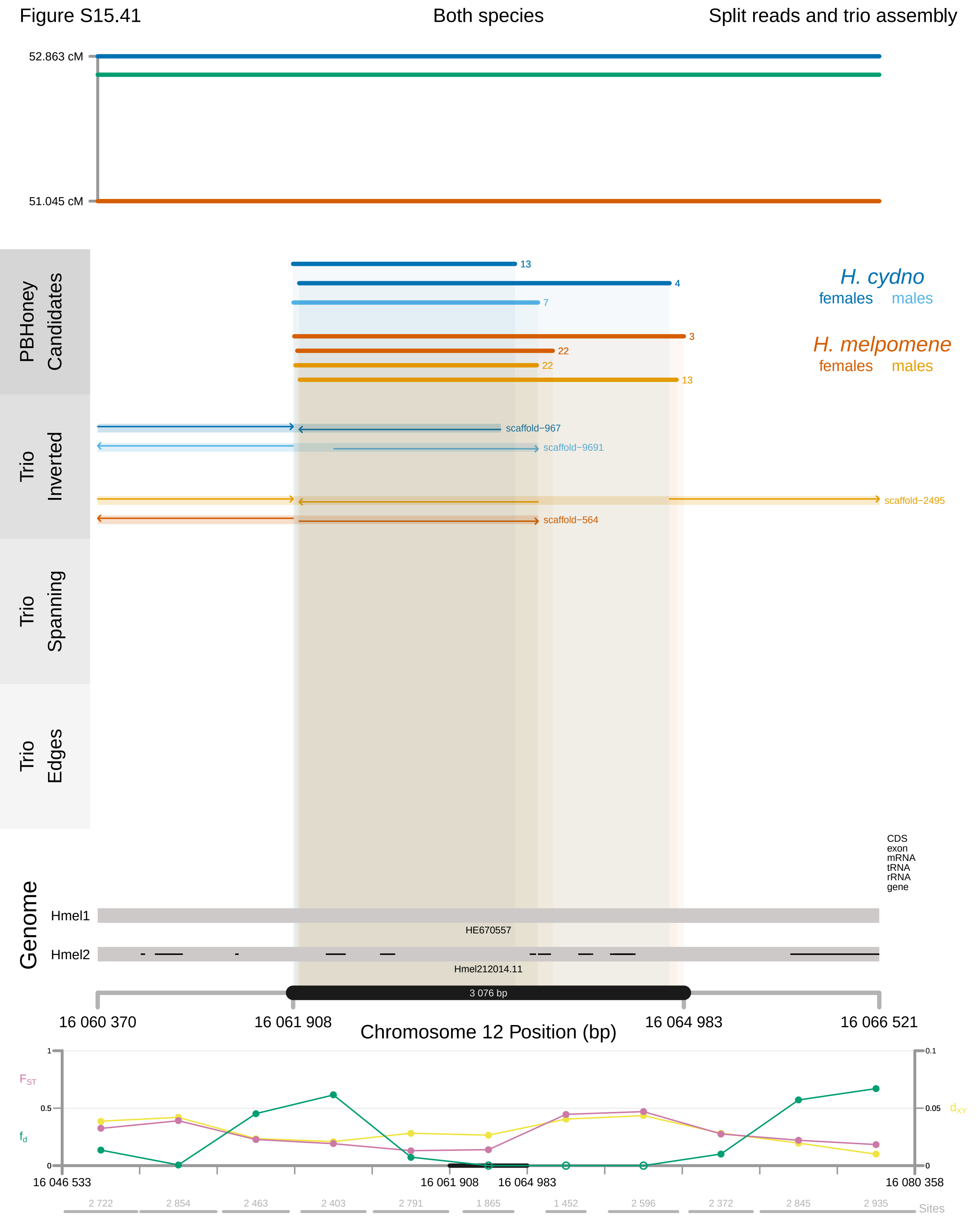
Figure S15.41
Both species
Split reads and trio assembly
52.863 cM
51.045 cM
PBHoney Candidates
13
4
7
3
22
22
13
H. cydno
females
males
H. melpomene
females
males
Trio Inverted
scaffold−967
scaffold−9691
scaffold−2495
scaffold−564
Trio Spanning
Trio Edges
CDS
exon
mRNA
tRNA
rRNA
gene
Genome
Hmel1
HE670557
Hmel2
Hmel212014.11
3 076 bp
16 060 370
16 061 908
16 064 983
16 066 521
Chromosome 12 Position (bp)
FST
fd
dXY
1
0.5
0
0.1
0.05
0
16 046 533
16 061 908
16 064 983
16 080 358
2 722
2 854
2 463
2 403
2 791
1 865
1 452
2 596
2 372
2 845
2 935
Sites

Figure S15.42

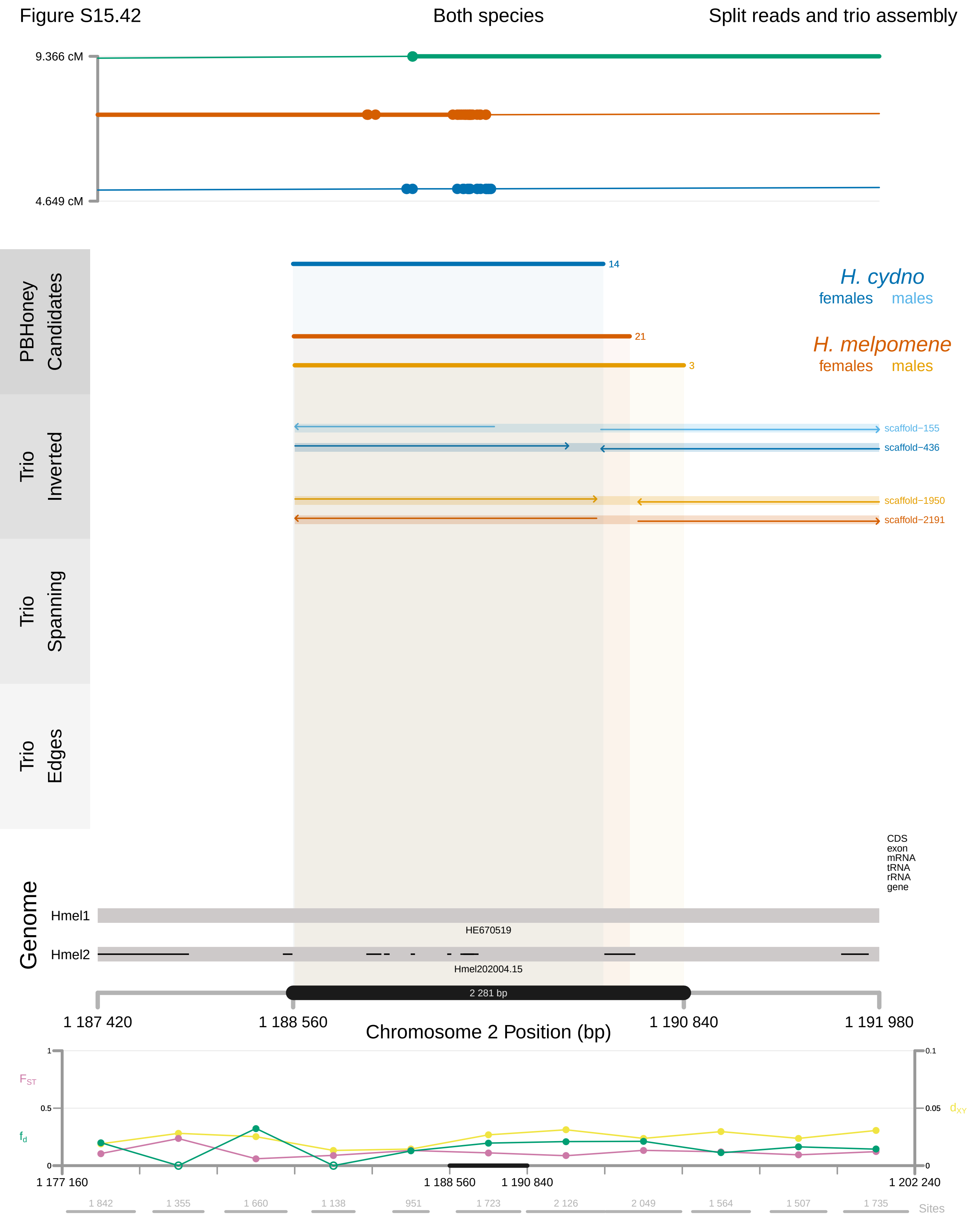

Supplement: Supplementary file 16 — S15, Both species, split reads and trio assembly. [file EVL3-1-138-s016.pdf]
