# Supplementary material for: No evidence for maintenance of a sympatric Heliconius species barrier by chromosomal inversions
Source: Evol Lett. 2017 Jun 14;1(3):138–54. doi: 10.1002/evl3.12 (PMC6122123; doi:10.1002/evl3.12)
Supplement: Supplementary file 17 — S16, Both species, split reads only. [file EVL3-1-138-s017.pdf]

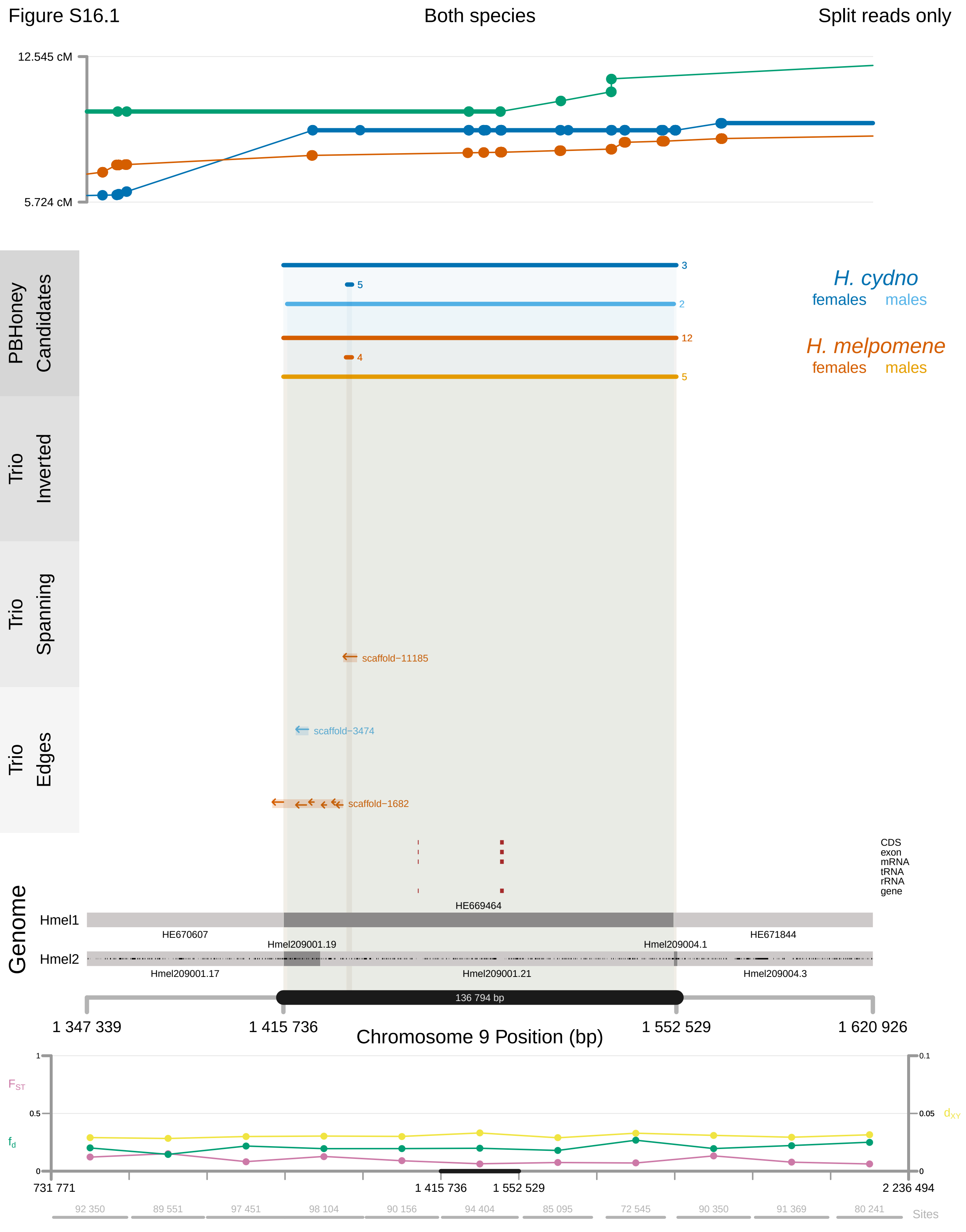

Figure S16.1
Both species
Split reads only
12.545 cM
5.724 cM
PBHoney Candidates
Trio Inverted
Trio Spanning
Trio Edges
Genome
H. cydno
females
males
H. melpomene
females
males
3
5
2
12
4
5
scaffold−11185
scaffold−3474
scaffold−1682
CDS
exon
mRNA
tRNA
rRNA
gene
HE669464
Hmel1
HE670607
HE671844
Hmel209001.19
Hmel209004.1
Hmel2
Hmel209001.17
Hmel209001.21
Hmel209004.3
136 794 bp
1 347 339
1 415 736
1 552 529
1 620 926
Chromosome 9 Position (bp)
1
0.5
0
F_{ST}
f_d
0.1
0.05
0
d_{XY}
731 771
1 415 736
1 552 529
2 236 494
92 350
89 551
97 451
98 104
90 156
94 404
85 095
72 545
90 350
91 369
80 241
Sites

Figure S16.2

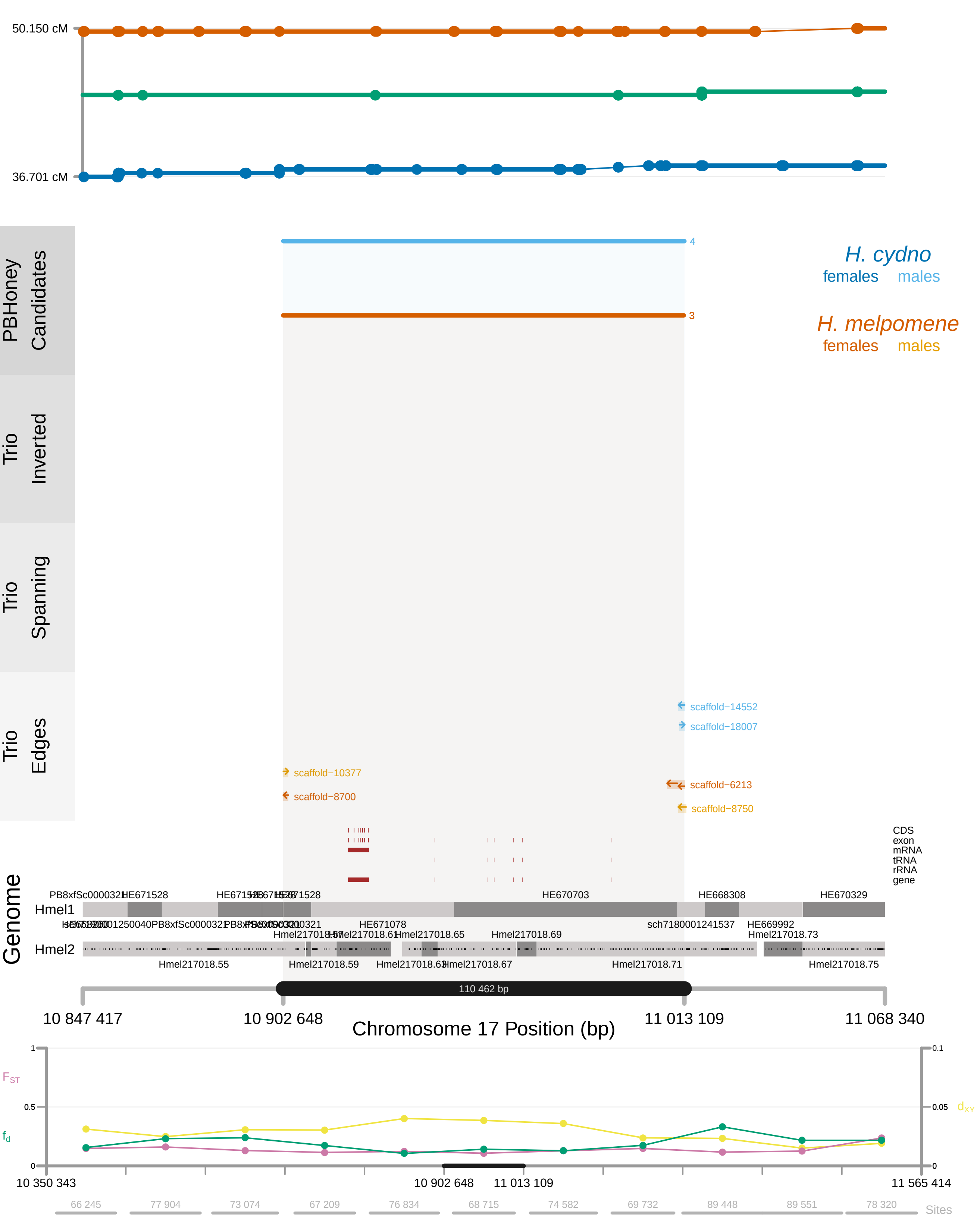
Both species
Split reads only
50.150 cM
36.701 cM
PBHoney Candidates
Trio Inverted
Trio Spanning
Trio Edges
H. cydno
females males
H. melpomene
females males
scaffold−14552
scaffold−18007
scaffold−10377
scaffold−8700
scaffold−6213
scaffold−8750
CDS
exon
mRNA
tRNA
rRNA
gene
Genome
Hmel1
Hmel2
HE670703
HE668308
HE670329
HE671078
sch7180001241537
HE669992
Hmel217018.55
Hmel217018.59
Hmel217018.65
Hmel217018.67
Hmel217018.69
Hmel217018.71
Hmel217018.73
Hmel217018.75
110 462 bp
10 847 417
10 902 648
11 013 109
11 068 340
Chromosome 17 Position (bp)
FST
fd
dXY
10 350 343
10 902 648
11 013 109
11 565 414
66 245
77 904
73 074
67 209
76 834
68 715
74 582
69 732
89 448
89 551
78 320
Sites

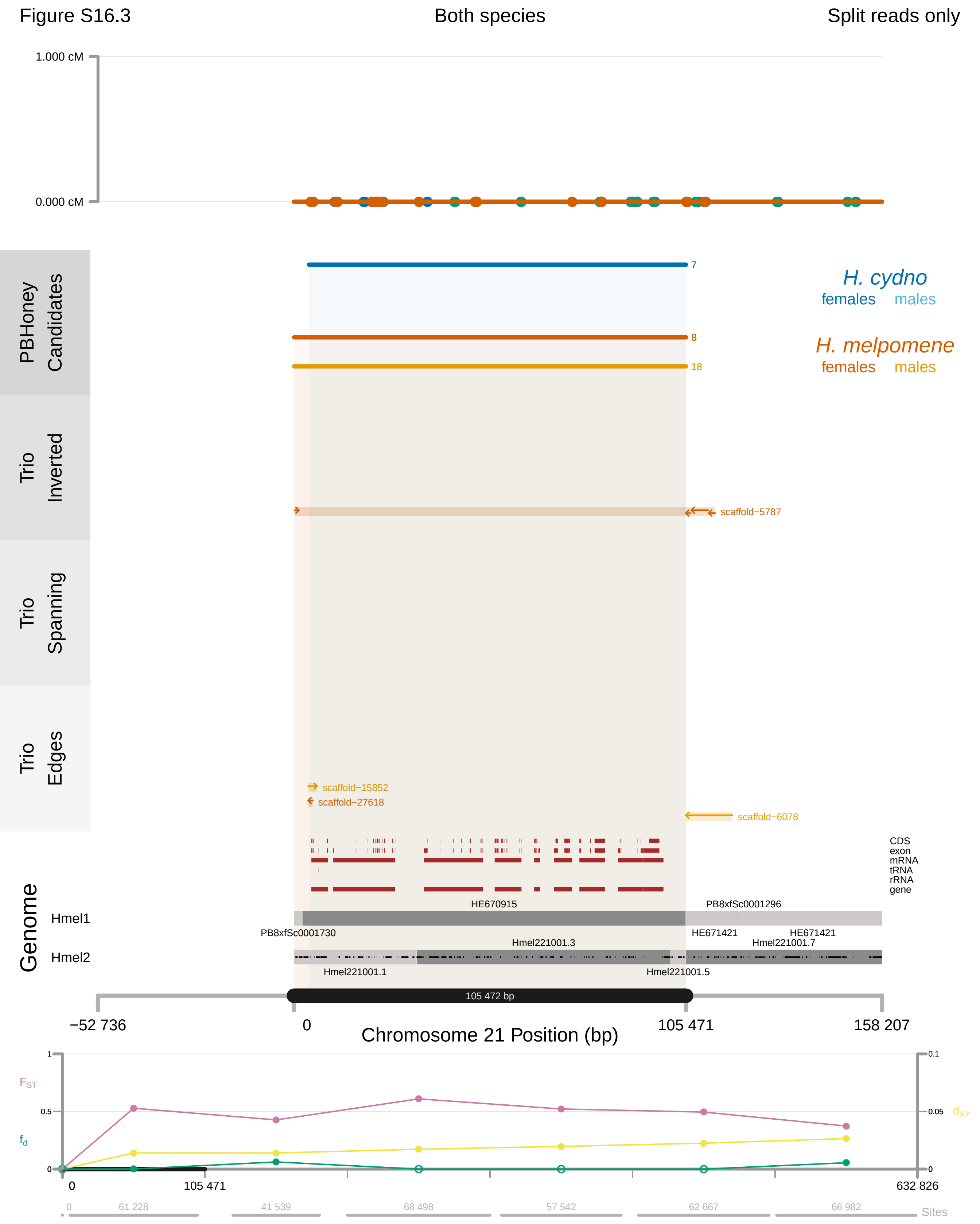

Figure S16.3
Both species
Split reads only
1.000 cM
0.000 cM
PBHoney Candidates
7
8
18
H. cydno
females males
H. melpomene
females males
Trio Inverted
scaffold−5787
Trio Spanning
Trio Edges
scaffold−15852
scaffold−27618
scaffold−6078
CDS
exon
mRNA
tRNA
rRNA
gene
Genome
HE670915
PB8xfSc0001296
Hmel1
PB8xfSc0001730
HE671421
HE671421
Hmel221001.3
Hmel221001.7
Hmel2
Hmel221001.1
Hmel221001.5
105 472 bp
−52 736
0
105 471
158 207
Chromosome 21 Position (bp)
1
0.5
0
0.1
0.05
0
F_{ST}
f_d
d_{XY}
0
105 471
632 826
0
61 228
41 539
68 498
57 542
62 667
66 982
Sites

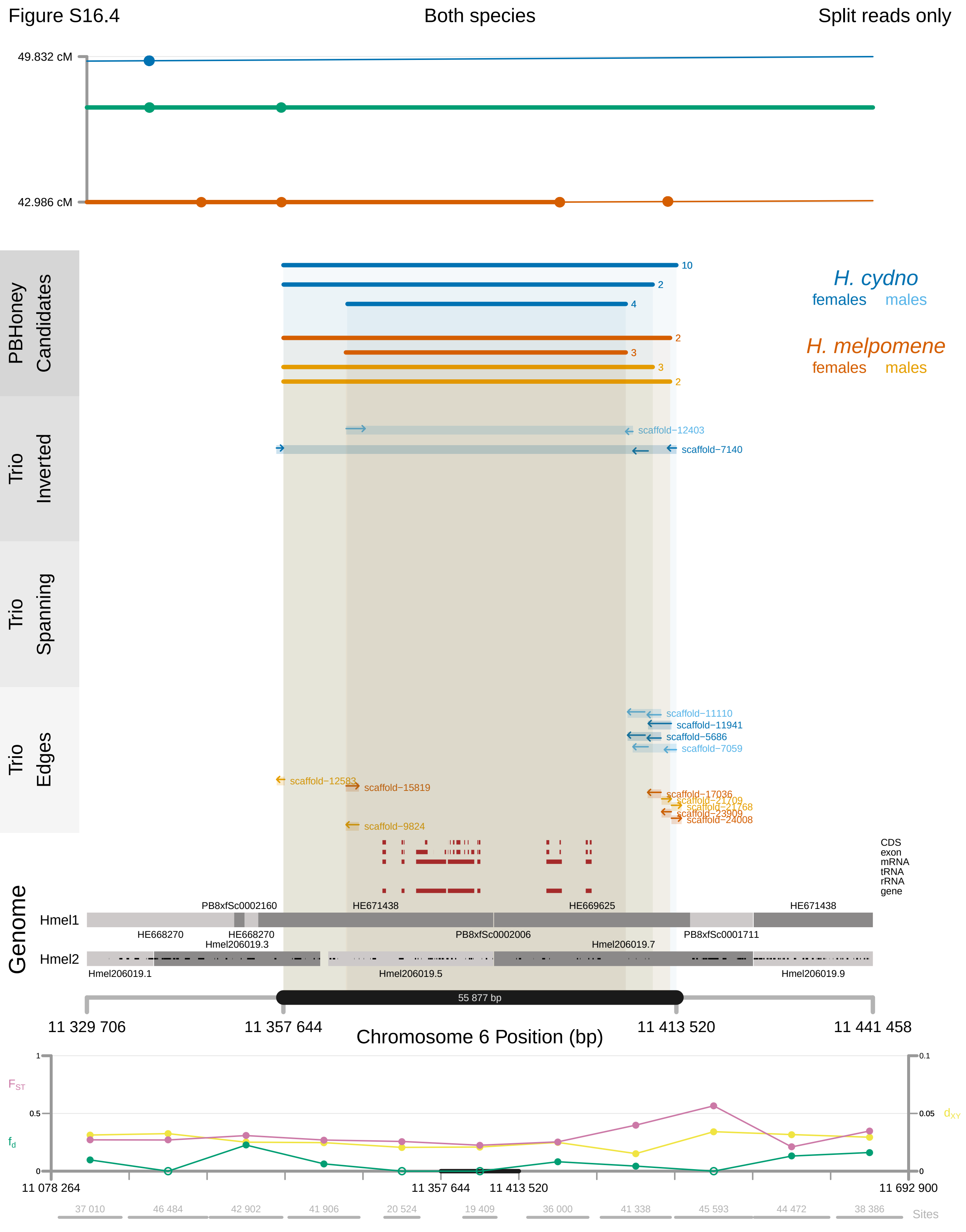
Figure S16.4
Both species
Split reads only
49.832 cM
42.986 cM
PBHoney Candidates
Trio Inverted
Trio Spanning
Trio Edges
Genome
H. cydno
females
males
H. melpomene
females
males
10
2
4
2
3
3
2
scaffold−12403
scaffold−7140
scaffold−11110
scaffold−11941
scaffold−5686
scaffold−7059
scaffold−12583
scaffold−15819
scaffold−9824
scaffold−17036
scaffold−21709
scaffold−21768
scaffold−23909
scaffold−24008
CDS
exon
mRNA
tRNA
rRNA
gene
Hmel1
Hmel2
PB8xfSc0002160
HE671438
HE669625
HE671438
HE668270
HE668270
PB8xfSc0002006
PB8xfSc0001711
Hmel206019.3
Hmel206019.7
Hmel206019.1
Hmel206019.5
Hmel206019.9
55 877 bp
11 329 706
11 357 644
11 413 520
11 441 458
Chromosome 6 Position (bp)
1
0.5
0
0.1
0.05
0
F_{ST}
f_d
d_{XY}
11 078 264
11 357 644
11 413 520
11 692 900
37 010
46 484
42 902
41 906
20 524
19 409
36 000
41 338
45 593
44 472
38 386
Sites

Figure S16.5

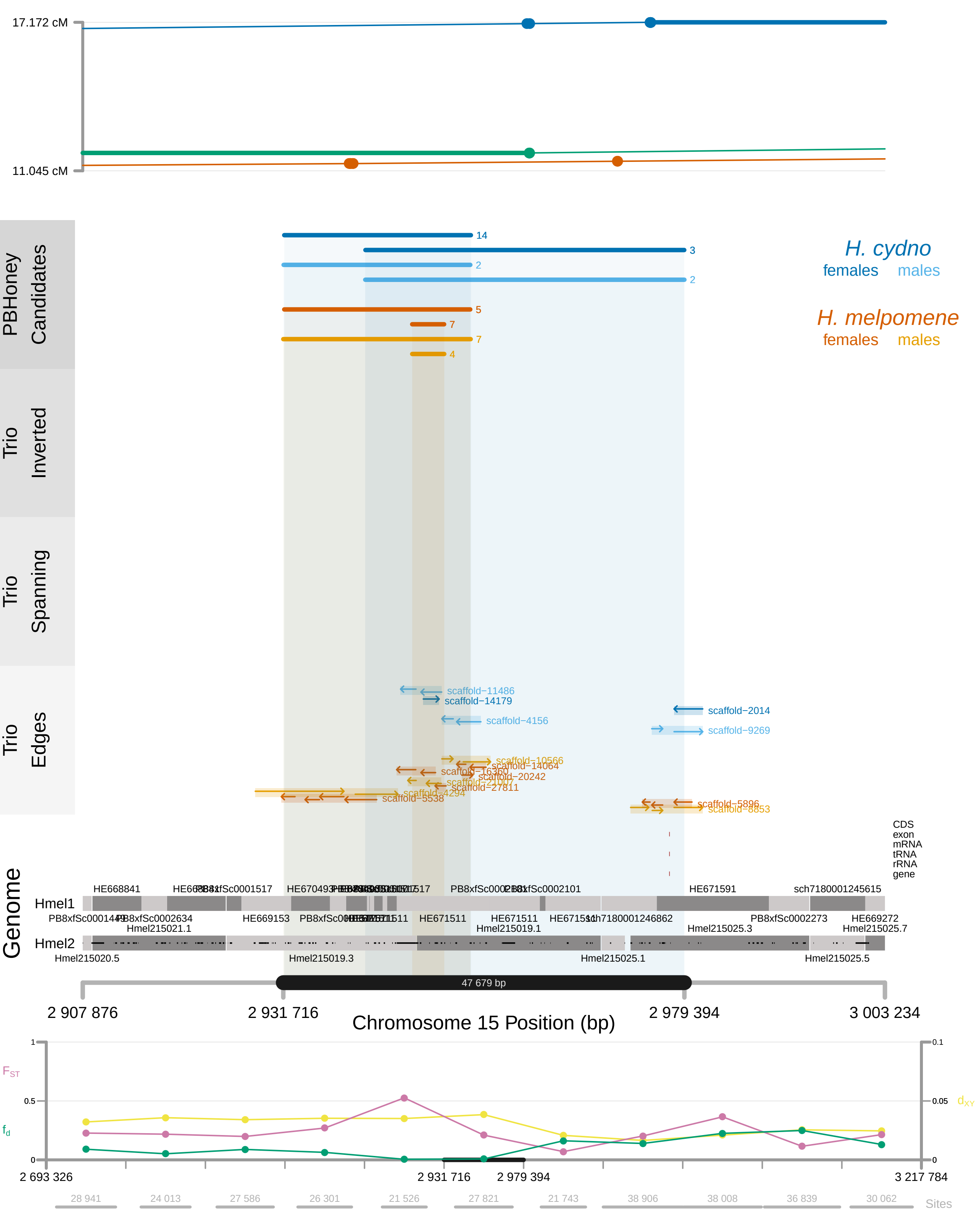

Both species
Split reads only
17.172 cM
11.045 cM
PBHoney Candidates
Trio Inverted
Trio Spanning
Trio Edges
Genome
H. cydno
females
males
H. melpomene
females
males
14
3
2
2
5
7
7
4
scaffold−11486
scaffold−14179
scaffold−4156
scaffold−2014
scaffold−9269
scaffold−10566
scaffold−14064
scaffold−16360
scaffold−20242
scaffold−21007
scaffold−27811
scaffold−4294
scaffold−5538
scaffold−5896
scaffold−8853
CDS
exon
mRNA
tRNA
rRNA
gene
Hmel1
Hmel2
HE668841
PB8xfSc0001517
HE671591
sch7180001245615
PB8xfSc0002101
HE669153
HE671511
Hmel215021.1
Hmel215019.1
sch7180001246862
PB8xfSc0002273
HE669272
Hmel215025.3
Hmel215025.7
Hmel215020.5
Hmel215019.3
Hmel215025.1
Hmel215025.5
47 679 bp
2 907 876
2 931 716
2 979 394
3 003 234
Chromosome 15 Position (bp)
F_{ST}
f_d
d_{XY}
1
0.5
0
0.1
0.05
0
2 693 326
2 931 716
2 979 394
3 217 784
28 941
24 013
27 586
26 301
21 526
27 821
21 743
38 906
38 008
36 839
30 062
Sites

Figure S16.6

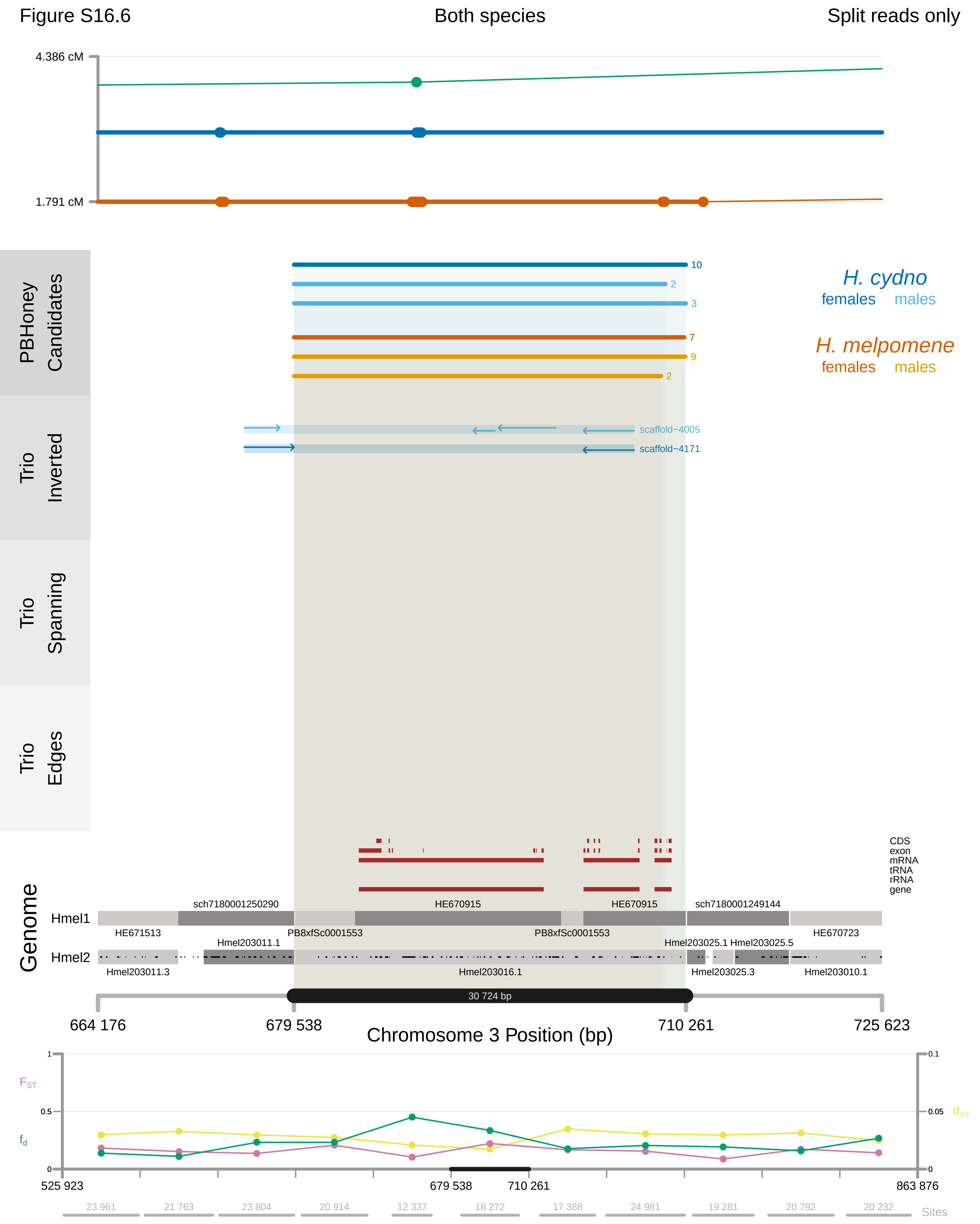

Both species
Split reads only
4.386 cM
1.791 cM
PBHoney Candidates
Trio Inverted
Trio Spanning
Trio Edges
Genome
H. cydno
females
males
H. melpomene
females
males
10
2
3
7
9
2
scaffold−4005
scaffold−4171
CDS
exon
mRNA
tRNA
rRNA
gene
Hmel1
Hmel2
sch7180001250290
HE670915
HE670915
sch7180001249144
HE671513
PB8xfSc0001553
PB8xfSc0001553
HE670723
Hmel203011.1
Hmel203025.1 Hmel203025.5
Hmel203011.3
Hmel203016.1
Hmel203025.3
Hmel203010.1
30 724 bp
664 176
679 538
710 261
725 623
Chromosome 3 Position (bp)
1
0.5
0
F_{ST}
f_d
0.1
0.05
0
d_{XY}
525 923
679 538
710 261
863 876
23 961
21 763
23 804
20 914
12 337
18 272
17 388
24 981
19 281
20 792
20 232
Sites

Figure S16.7

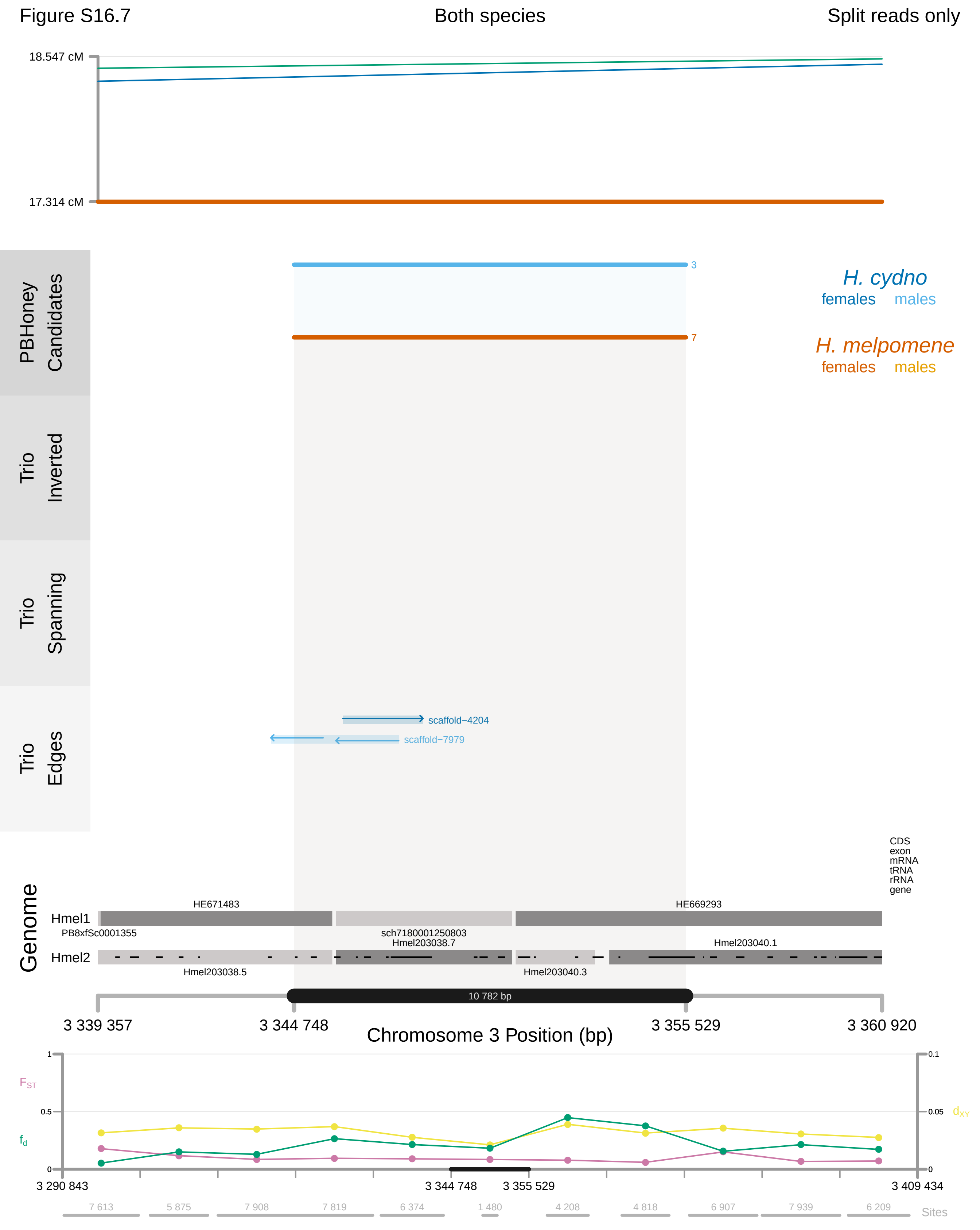

Both species
Split reads only
18.547 cM
17.314 cM
PBHoney Candidates
Trio Inverted
Trio Spanning
Trio Edges
3
7
H. cydno
females
males
H. melpomene
females
males
scaffold−4204
scaffold−7979
CDS
exon
mRNA
tRNA
rRNA
gene
Genome
HE671483
HE669293
Hmel1
PB8xfSc0001355
sch7180001250803
Hmel203038.7
Hmel203040.1
Hmel2
Hmel203038.5
Hmel203040.3
10 782 bp
3 339 357
3 344 748
3 355 529
3 360 920
Chromosome 3 Position (bp)
1
0.5
0
F_{ST}
f_d
0.1
0.05
0
d_{XY}
3 290 843
3 344 748
3 355 529
3 409 434
7 613
5 875
7 908
7 819
6 374
1 480
4 208
4 818
6 907
7 939
6 209
Sites

Figure S16.8

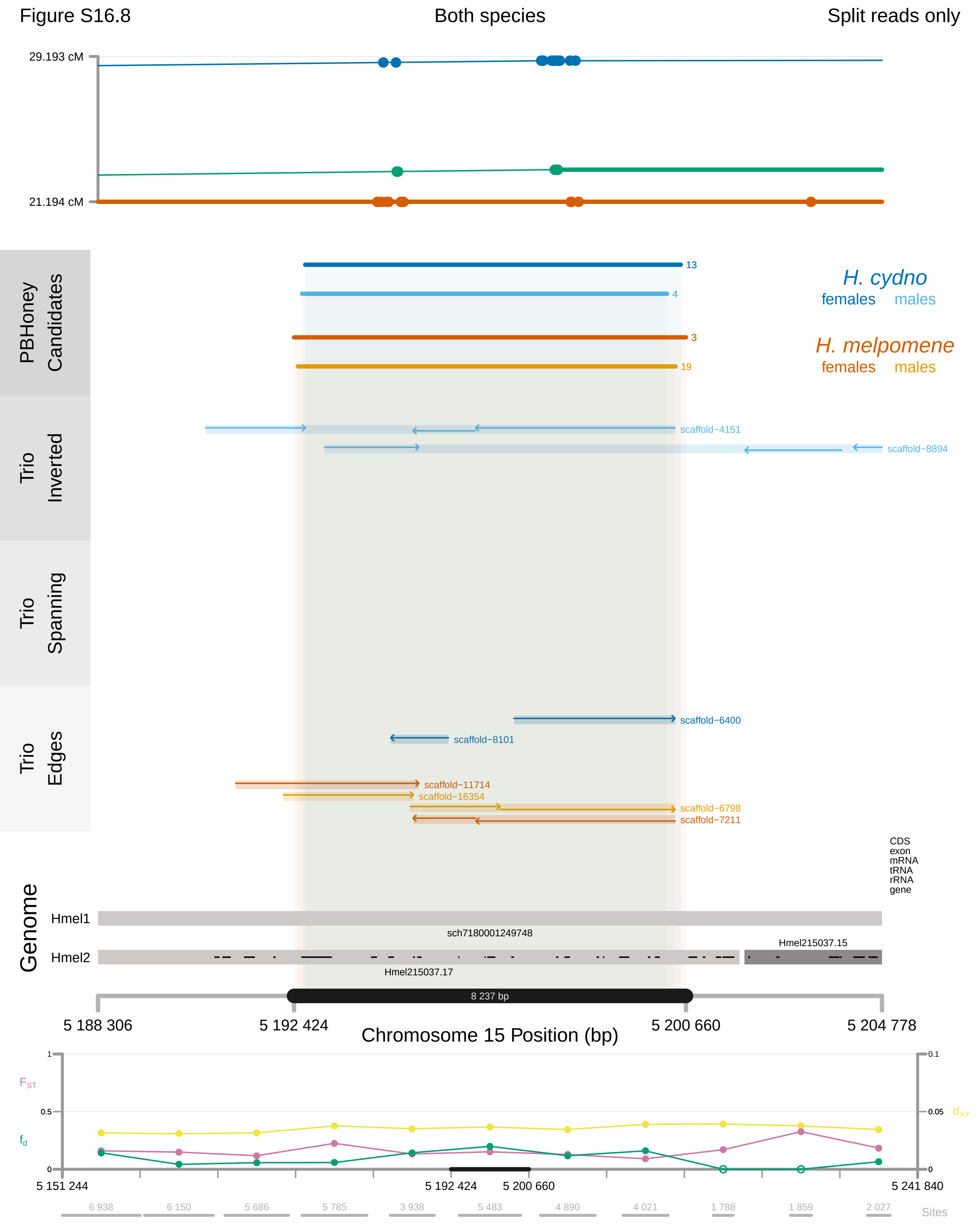

Both species
Split reads only
29.193 cM
21.194 cM
PBHoney Candidates
13
4
3
19
H. cydno
females males
H. melpomene
females males
Trio Inverted
scaffold−4151
scaffold−8894
Trio Spanning
Trio Edges
scaffold−6400
scaffold−8101
scaffold−11714
scaffold−16354
scaffold−6798
scaffold−7211
CDS
exon
mRNA
tRNA
rRNA
gene
Genome
Hmel1
sch7180001249748
Hmel2
Hmel215037.15
Hmel215037.17
8 237 bp
5 188 306
5 192 424
5 200 660
5 204 778
Chromosome 15 Position (bp)
1
0.5
0
F_ST
f_d
0.1
0.05
0
d_XY
5 151 244
5 192 424
5 200 660
5 241 840
6 938
6 150
5 686
5 785
3 938
5 483
4 890
4 021
1 788
1 859
2 027
Sites

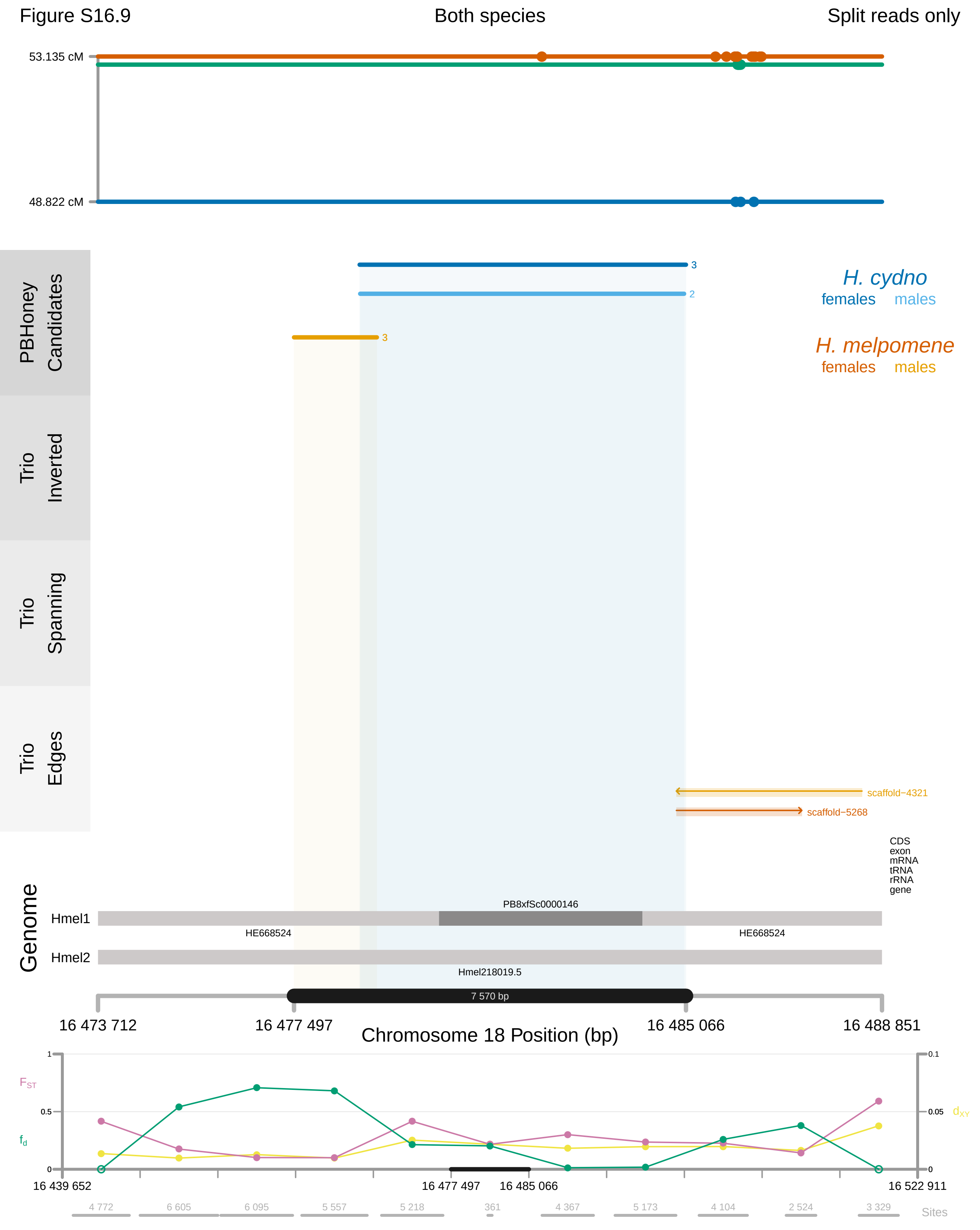

Figure S16.9
Both species
Split reads only
53.135 cM
48.822 cM
PBHoney Candidates
Trio Inverted
Trio Spanning
Trio Edges
Genome
H. cydno
females
males
H. melpomene
females
males
3
2
3
scaffold−4321
scaffold−5268
CDS
exon
mRNA
tRNA
rRNA
gene
Hmel1
Hmel2
PB8xfSc0000146
HE668524
HE668524
Hmel218019.5
7 570 bp
16 473 712
16 477 497
16 485 066
16 488 851
Chromosome 18 Position (bp)
F_ST
f_d
d_XY
1
0.5
0
0.1
0.05
0
16 439 652
16 477 497
16 485 066
16 522 911
4 772
6 605
6 095
5 557
5 218
361
4 367
5 173
4 104
2 524
3 329
Sites

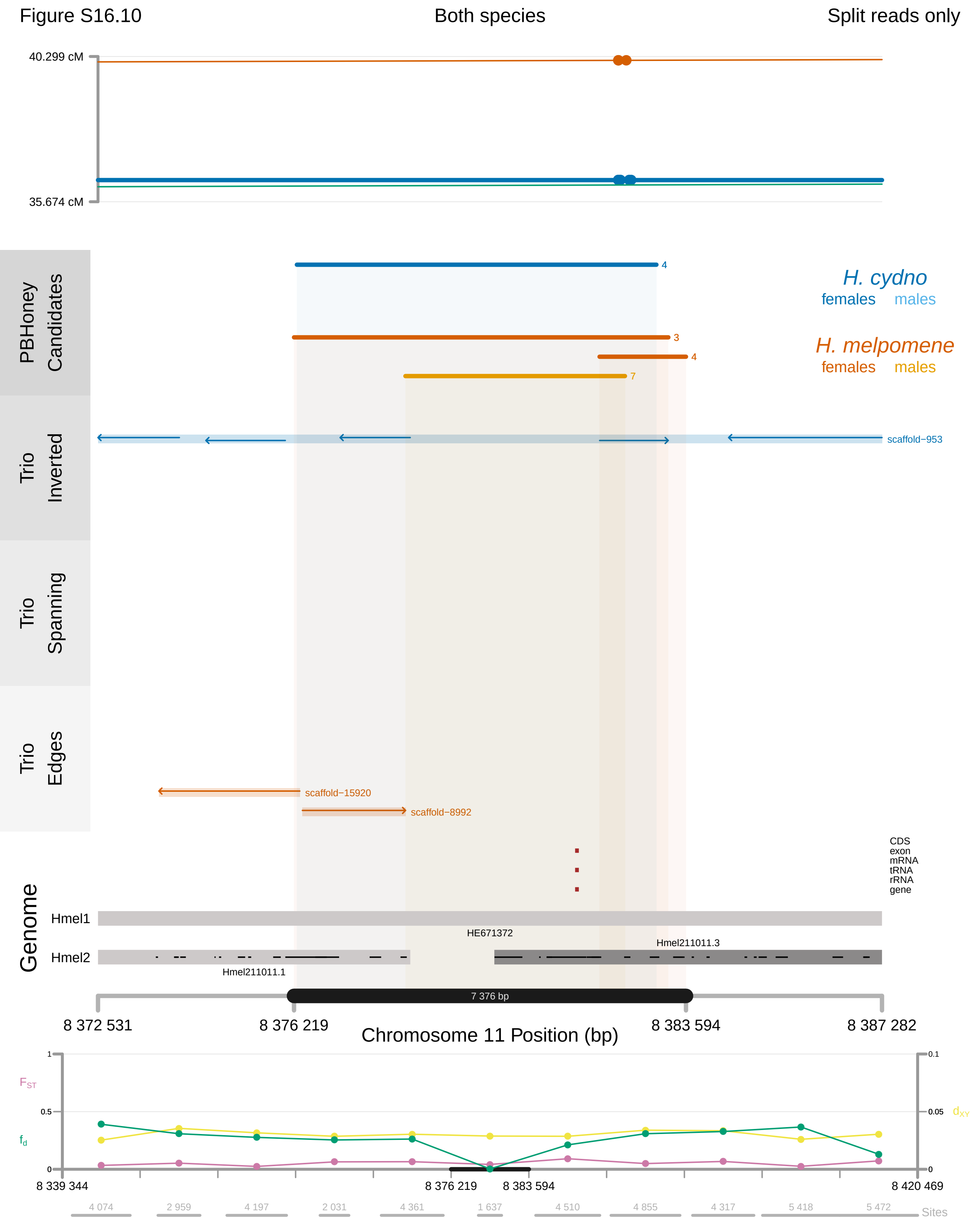

Figure S16.10
Both species
Split reads only
40.299 cM
35.674 cM
PBHoney Candidates
Trio Inverted
Trio Spanning
Trio Edges
Genome
H. cydno
females
males
H. melpomene
females
males
4
3
4
7
scaffold−953
scaffold−15920
scaffold−8992
CDS
exon
mRNA
tRNA
rRNA
gene
Hmel1
Hmel2
HE671372
Hmel211011.3
Hmel211011.1
7 376 bp
8 372 531
8 376 219
8 383 594
8 387 282
Chromosome 11 Position (bp)
F_ST
f_d
d_XY
1
0.5
0
0.1
0.05
0
8 339 344
8 376 219
8 383 594
8 420 469
4 074
2 959
4 197
2 031
4 361
1 637
4 510
4 855
4 317
5 418
5 472
Sites

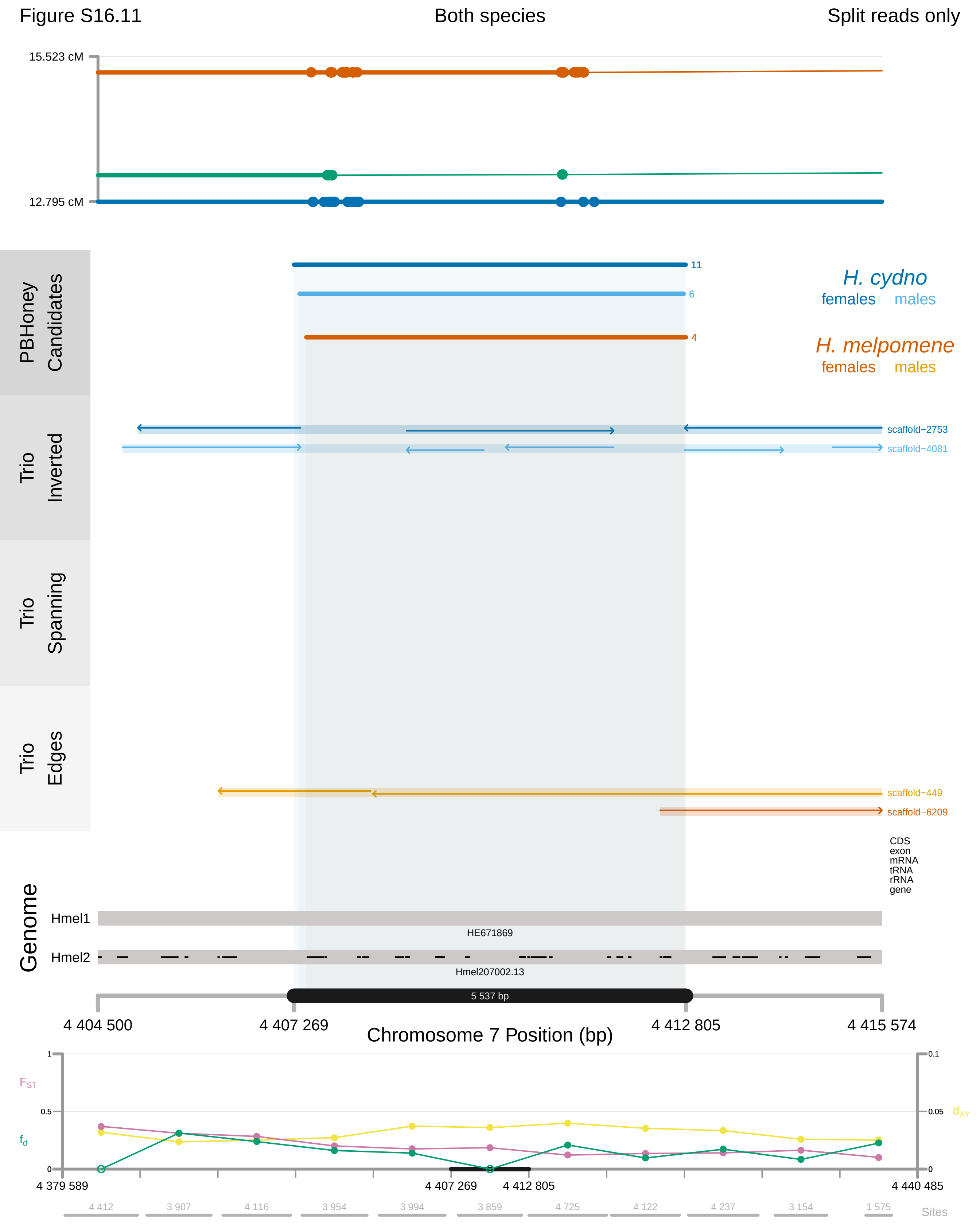

Figure S16.11
Both species
Split reads only
15.523 cM
12.795 cM
PBHoney Candidates
11
6
4
H. cydno
females males
H. melpomene
females males
Trio Inverted
scaffold−2753
scaffold−4081
Trio Spanning
Trio Edges
scaffold−449
scaffold−6209
CDS
exon
mRNA
tRNA
rRNA
gene
Genome
Hmel1
HE671869
Hmel2
Hmel207002.13
5 537 bp
4 404 500
4 407 269
4 412 805
4 415 574
Chromosome 7 Position (bp)
1
0.5
0
FST
fd
0.1
0.05
0
dXY
4 379 589
4 407 269
4 412 805
4 440 485
4 412
3 907
4 116
3 954
3 994
3 859
4 725
4 122
4 237
3 154
1 575
Sites

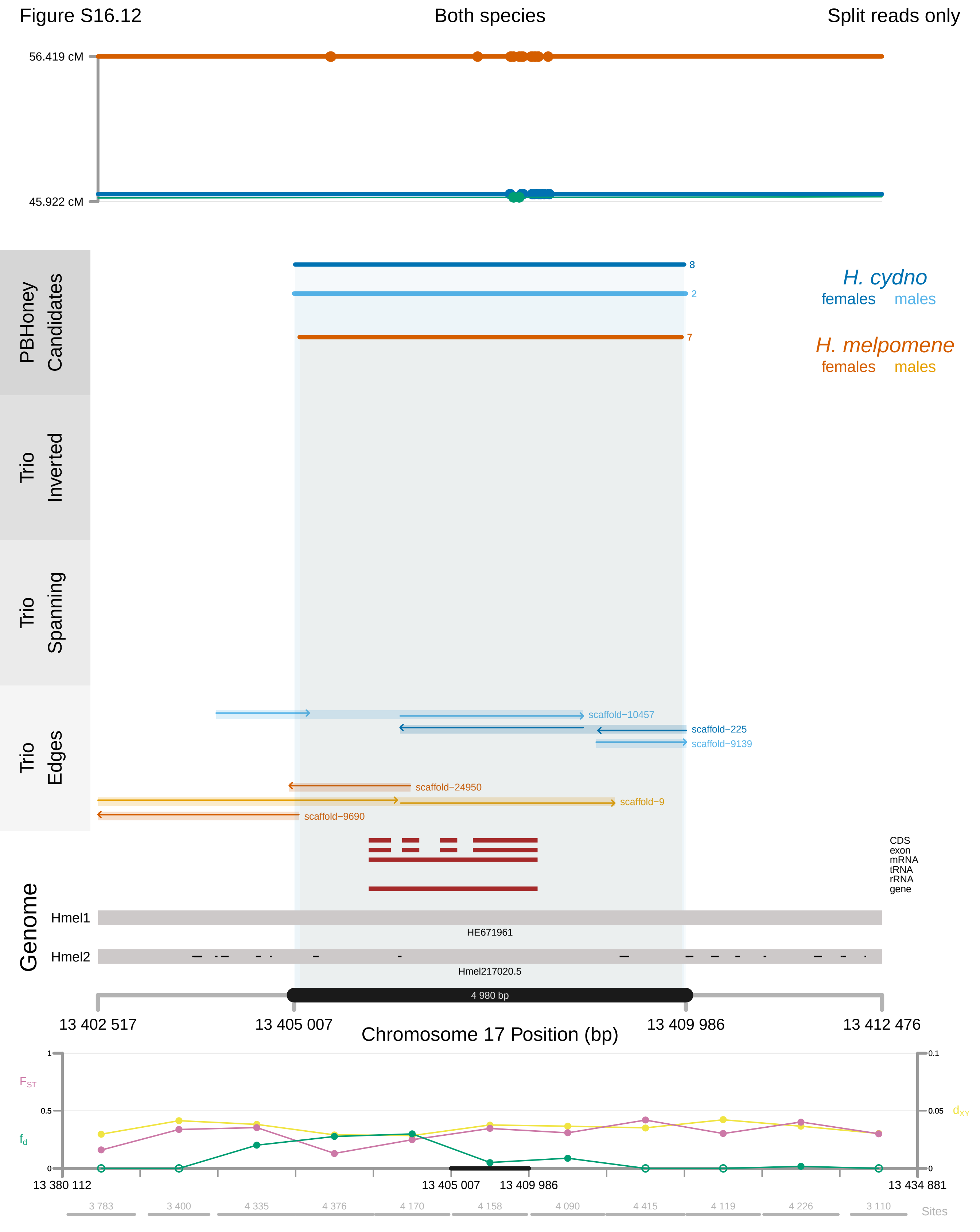

Figure S16.12
Both species
Split reads only
56.419 cM
45.922 cM
PBHoney Candidates
Trio Inverted
Trio Spanning
Trio Edges
Genome
8
2
7
H. cydno
females
males
H. melpomene
females
males
scaffold−10457
scaffold−225
scaffold−9139
scaffold−24950
scaffold−9
scaffold−9690
CDS
exon
mRNA
tRNA
rRNA
gene
Hmel1
HE671961
Hmel2
Hmel217020.5
4 980 bp
13 402 517
13 405 007
13 409 986
13 412 476
Chromosome 17 Position (bp)
F_{ST}
f_d
d_{XY}
13 380 112
13 405 007
13 409 986
13 434 881
3 783
3 400
4 335
4 376
4 170
4 158
4 090
4 415
4 119
4 226
3 110
Sites

Figure S16.13

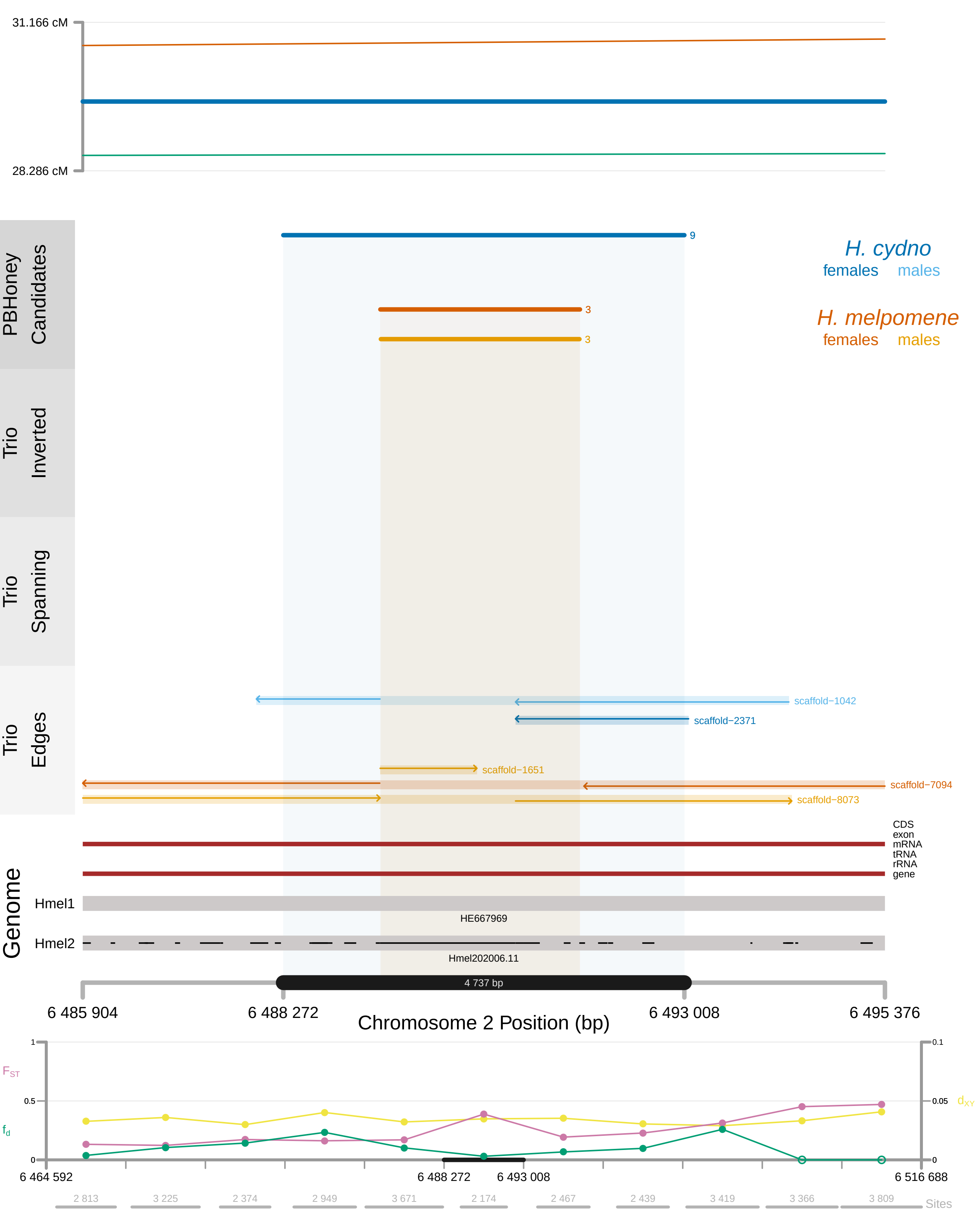

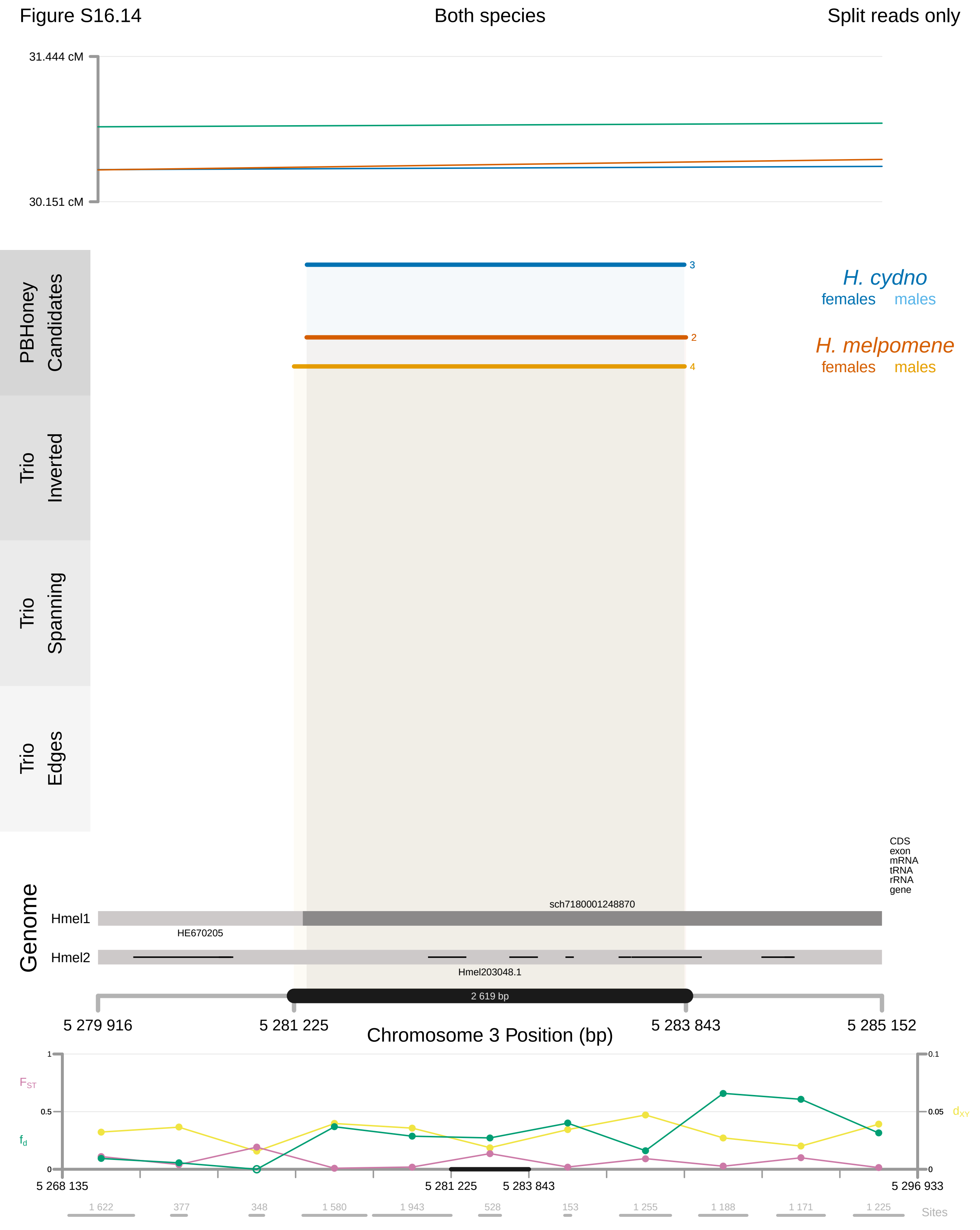
Figure S16.14
Both species
Split reads only
31.444 cM
30.151 cM
PBHoney Candidates
Trio Inverted
Trio Spanning
Trio Edges
3
2
4
H. cydno
females
males
H. melpomene
females
males
CDS
exon
mRNA
tRNA
rRNA
gene
Genome
Hmel1
Hmel2
sch7180001248870
HE670205
Hmel203048.1
2 619 bp
5 279 916
5 281 225
5 283 843
5 285 152
Chromosome 3 Position (bp)
F_ST
f_d
d_XY
1
0.5
0
0.1
0.05
0
5 268 135
5 281 225
5 283 843
5 296 933
1 622
377
348
1 580
1 943
528
153
1 255
1 188
1 171
1 225
Sites

Figure S16.15 Both species Split reads only

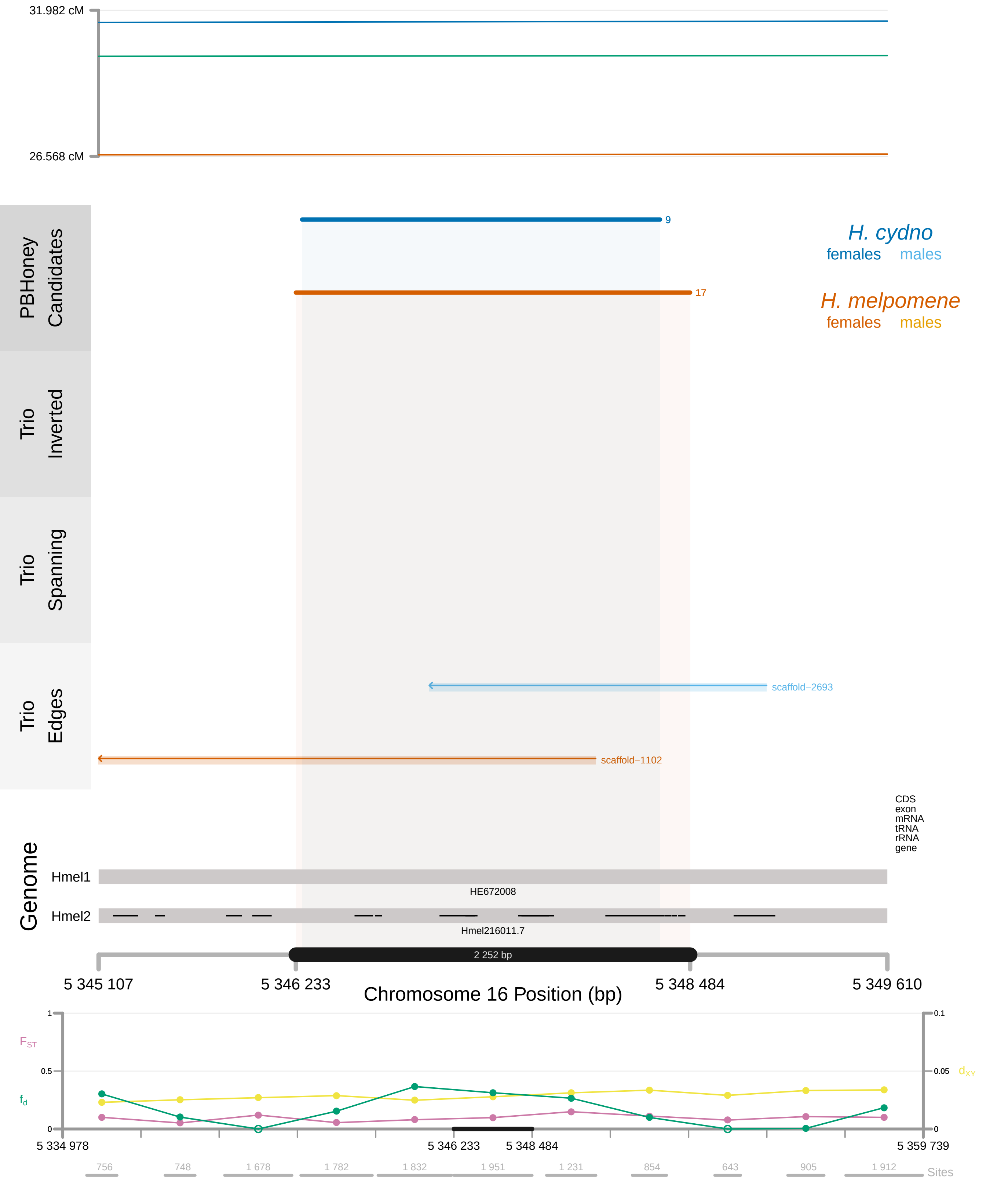

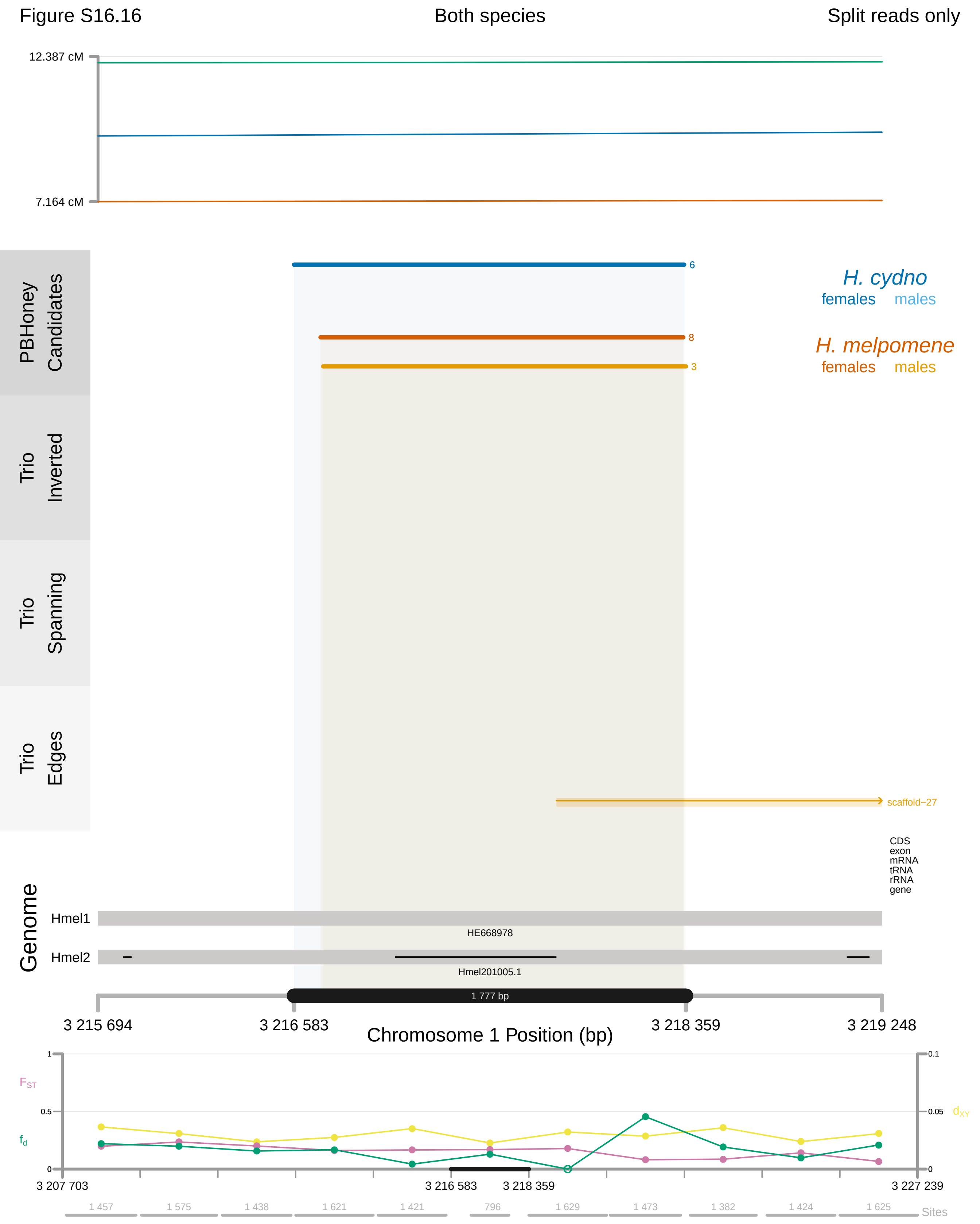

Figure S16.16
Both species
Split reads only
12.387 cM
7.164 cM
PBHoney Candidates
Trio Inverted
Trio Spanning
Trio Edges
6
8
3
H. cydno
females males
H. melpomene
females males
scaffold−27
CDS
exon
mRNA
tRNA
rRNA
gene
Genome
Hmel1
HE668978
Hmel2
Hmel201005.1
1 777 bp
3 215 694
3 216 583
3 218 359
3 219 248
Chromosome 1 Position (bp)
1
0.5
0
F_ST
f_d
0.1
0.05
0
d_XY
3 207 703
3 216 583
3 218 359
3 227 239
1 457
1 575
1 438
1 621
1 421
796
1 629
1 473
1 382
1 424
1 625
Sites

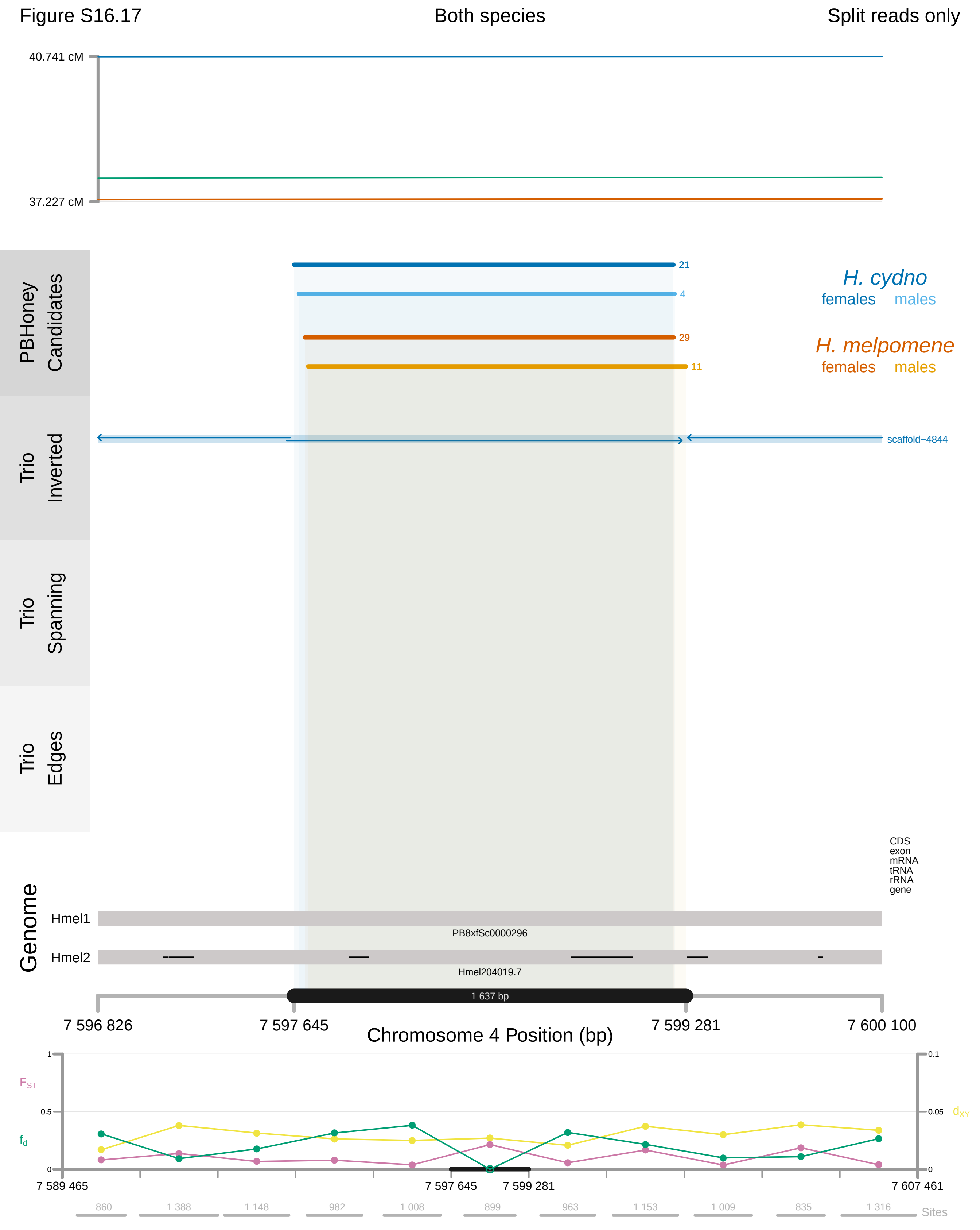

Figure S16.17
Both species
Split reads only
40.741 cM
37.227 cM
PBHoney Candidates
Trio Inverted
Trio Spanning
Trio Edges
Genome
21
4
29
11
H. cydno
females
males
H. melpomene
females
males
scaffold−4844
CDS
exon
mRNA
tRNA
rRNA
gene
Hmel1
PB8xfSc0000296
Hmel2
Hmel204019.7
1 637 bp
7 596 826
7 597 645
7 599 281
7 600 100
Chromosome 4 Position (bp)
F_ST
f_d
d_XY
1
0.5
0
0.1
0.05
0
7 589 465
7 597 645
7 599 281
7 607 461
860
1 388
1 148
982
1 008
899
963
1 153
1 009
835
1 316
Sites
