# Supplementary material for: No evidence for maintenance of a sympatric Heliconius species barrier by chromosomal inversions
Source: Evol Lett. 2017 Jun 14;1(3):138–54. doi: 10.1002/evl3.12 (PMC6122123; doi:10.1002/evl3.12)

Figure S17.1

Both species Split reads in one species, trio assembly in both

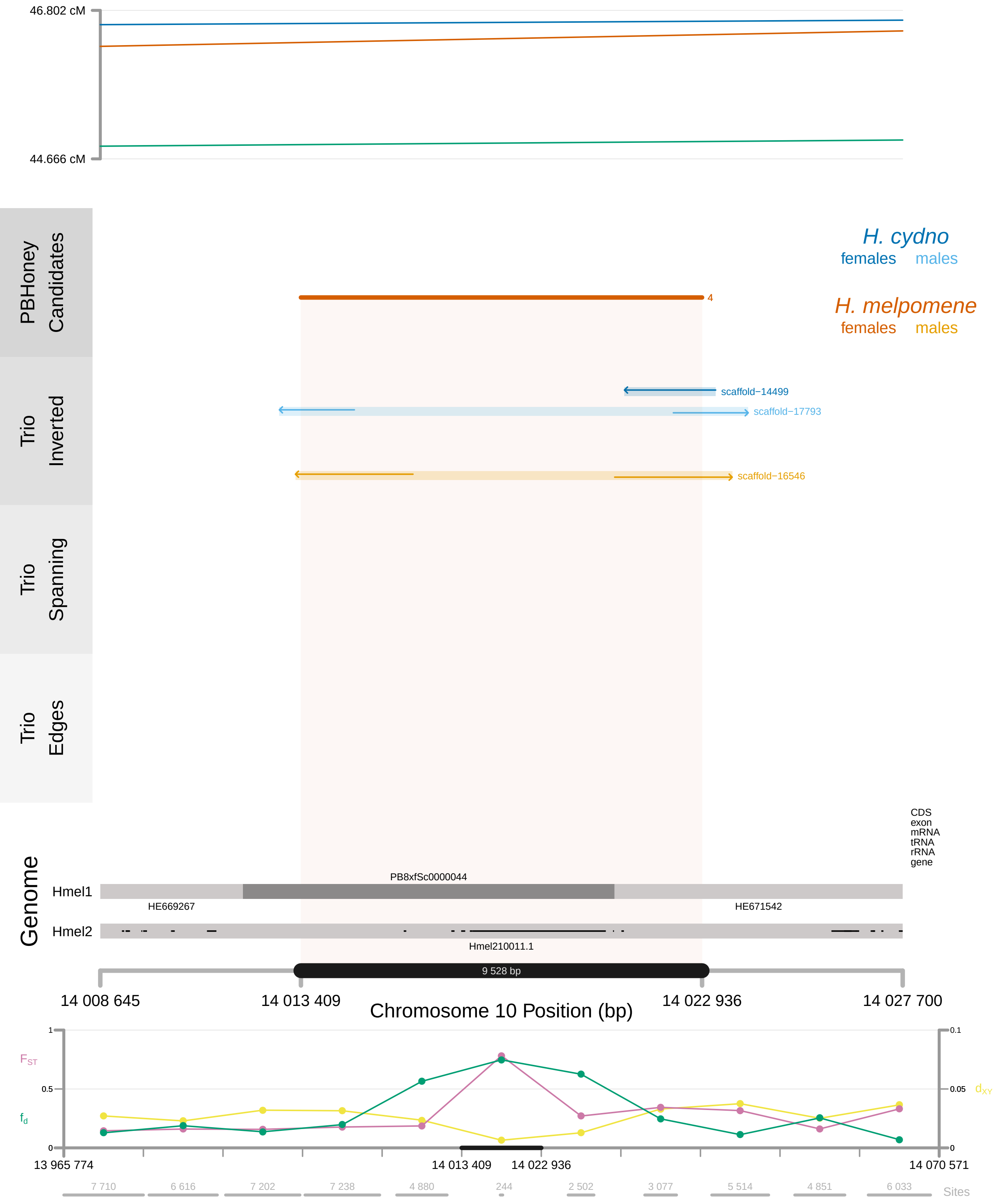

Figure S17.2

Both species Split reads in one species, trio assembly in both

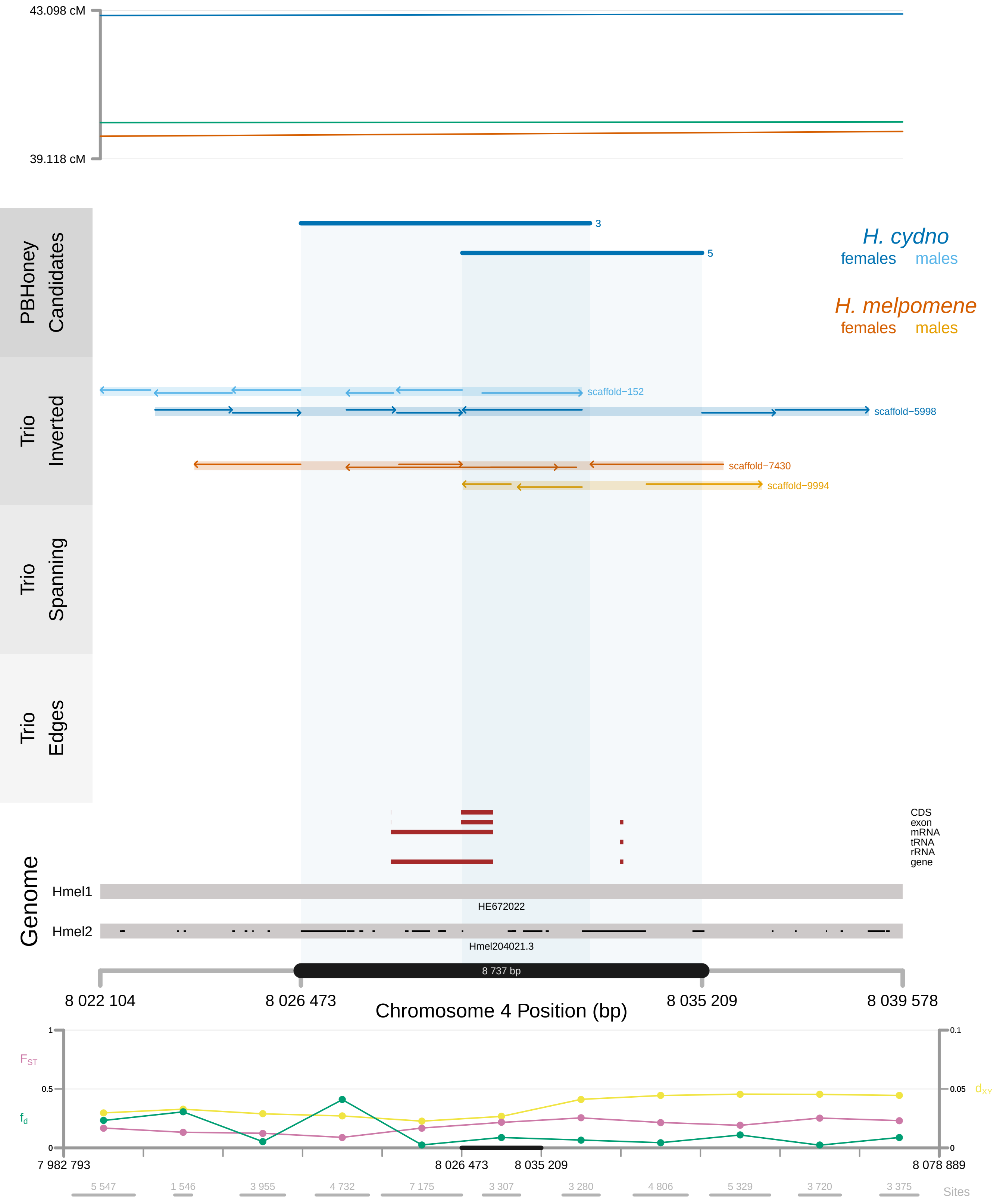

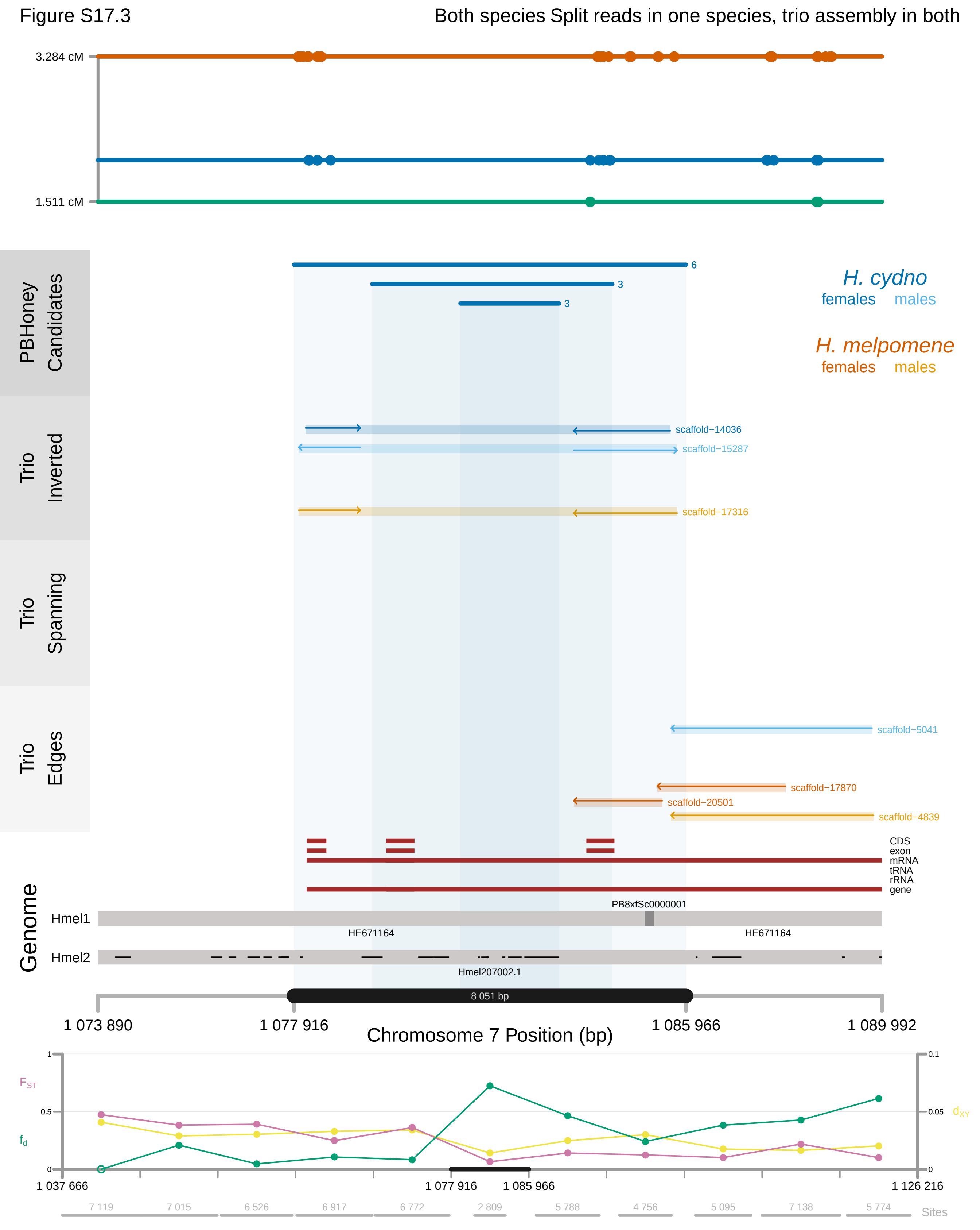

Figure S17.3
Both species Split reads in one species, trio assembly in both
3.284 cM
1.511 cM
PBHoney Candidates
Trio Inverted
Trio Spanning
Trio Edges
Genome
6
3
3
H. cydno
females males
H. melpomene
females males
scaffold−14036
scaffold−15287
scaffold−17316
scaffold−5041
scaffold−17870
scaffold−20501
scaffold−4839
CDS
exon
mRNA
tRNA
rRNA
gene
PB8xfSc0000001
Hmel1
HE671164
HE671164
Hmel2
Hmel207002.1
8 051 bp
1 073 890
1 077 916
1 085 966
1 089 992
Chromosome 7 Position (bp)
F_{ST}
f_d
d_{XY}
1
0.5
0
0.1
0.05
0
1 037 666
1 077 916
1 085 966
1 126 216
7 119
7 015
6 526
6 917
6 772
2 809
5 788
4 756
5 095
7 138
5 774
Sites

Figure S17.4

Both species Split reads in one species, trio assembly in both

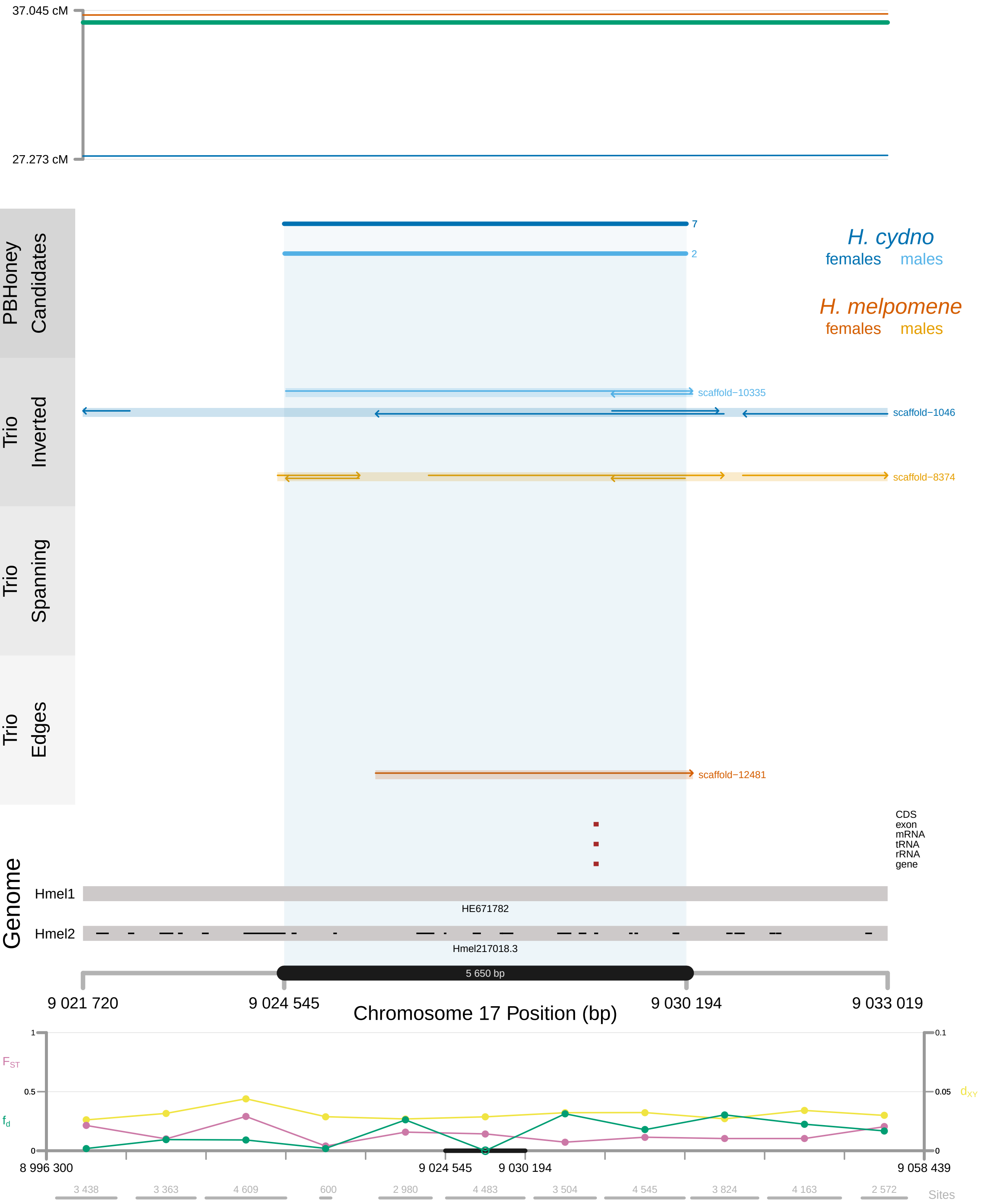

Figure S17.5 Both species Split reads in one species, trio assembly in both

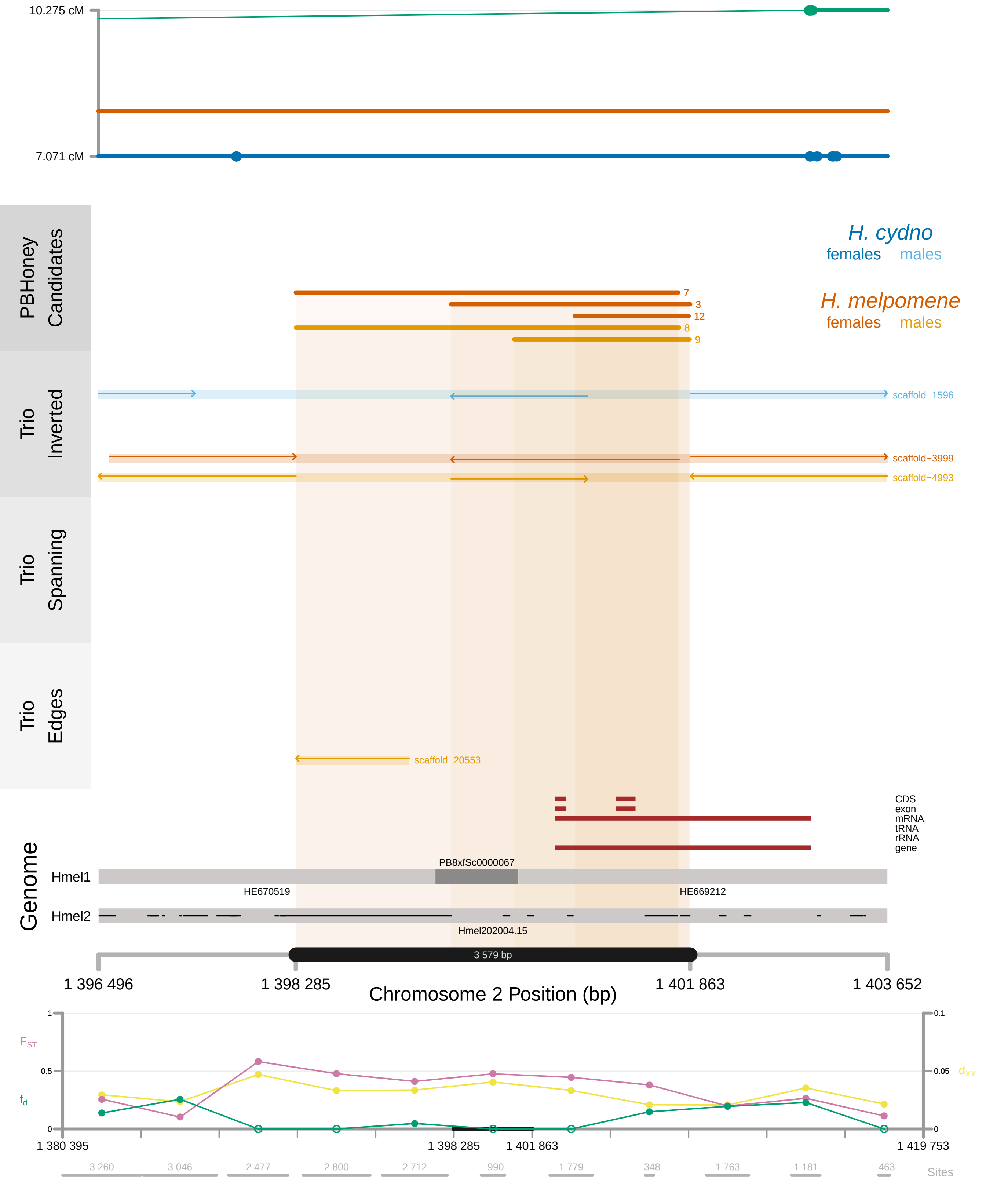

Figure S17.6

Both species Split reads in one species, trio assembly in both

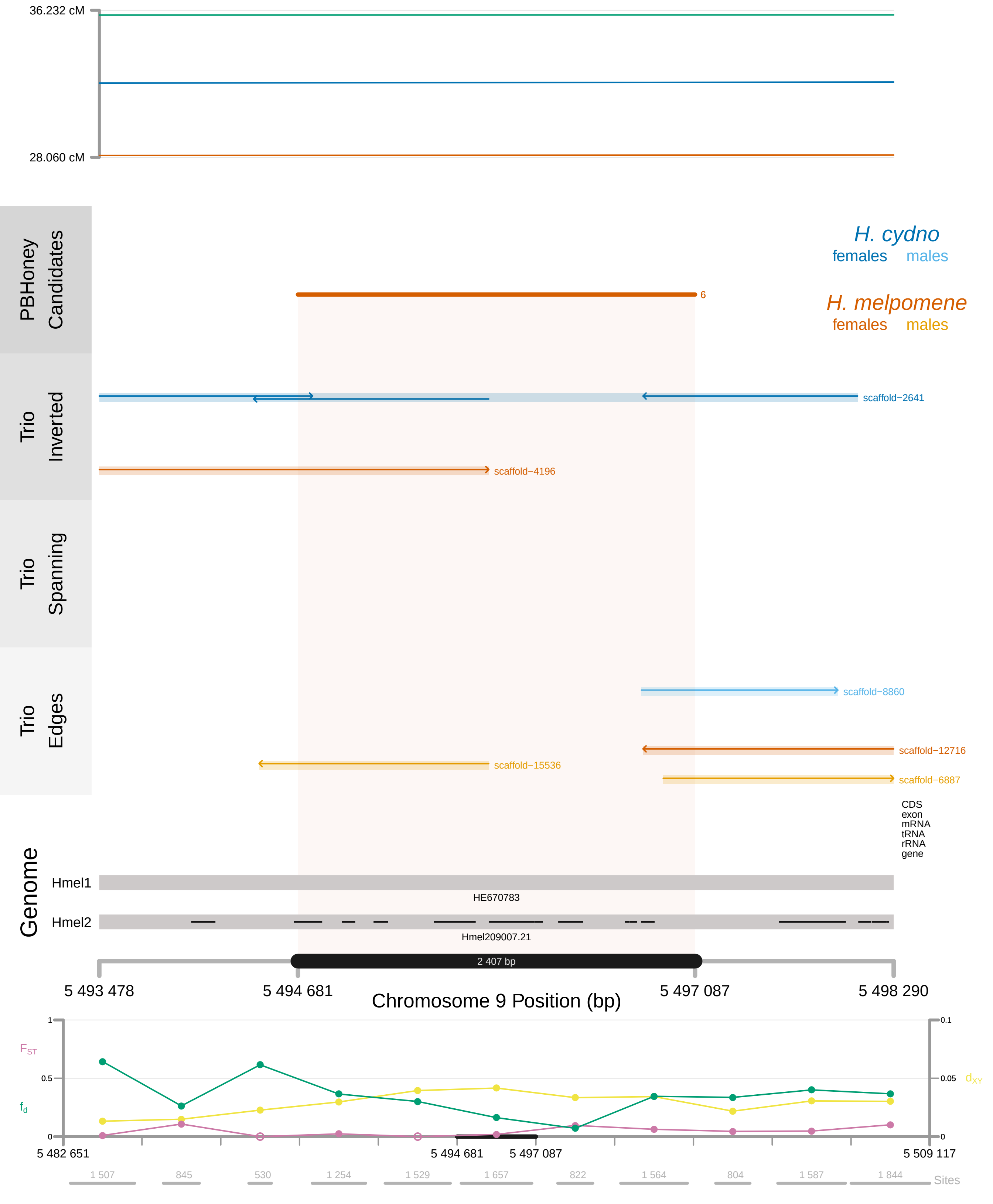

Supplement: Supplementary file 18 — S17, Both species, split reads in one species, trio assembly in both. [file EVL3-1-138-s018.pdf]
