# Supplementary material for: No evidence for maintenance of a sympatric Heliconius species barrier by chromosomal inversions
Source: Evol Lett. 2017 Jun 14;1(3):138–54. doi: 10.1002/evl3.12 (PMC6122123; doi:10.1002/evl3.12)
Supplement: Supplementary file 19 — Figure S18. Oxford grids for ordered Hmel2 scaffolds and H. erato scaffolds. [file EVL3-1-138-s019.pdf]

1

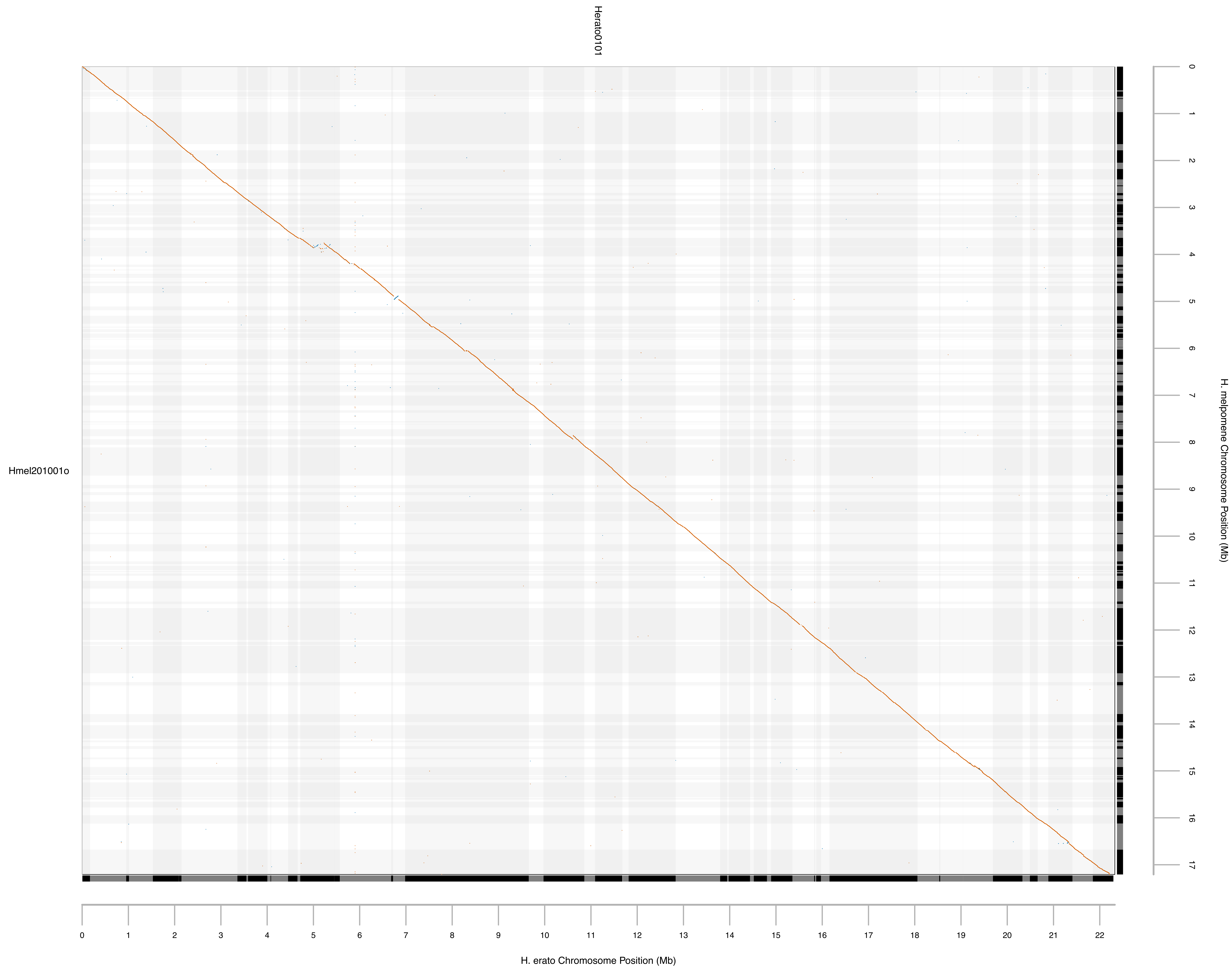
Herato0101
Hmel201001o
H. melpomene Chromosome Position (Mb)
H. erato Chromosome Position (Mb)
0 1 2 3 4 5 6 7 8 9 10 11 12 13 14 15 16 17 18 19 20 21 22
0 1 2 3 4 5 6 7 8 9 10 11 12 13 14 15 16 17

2

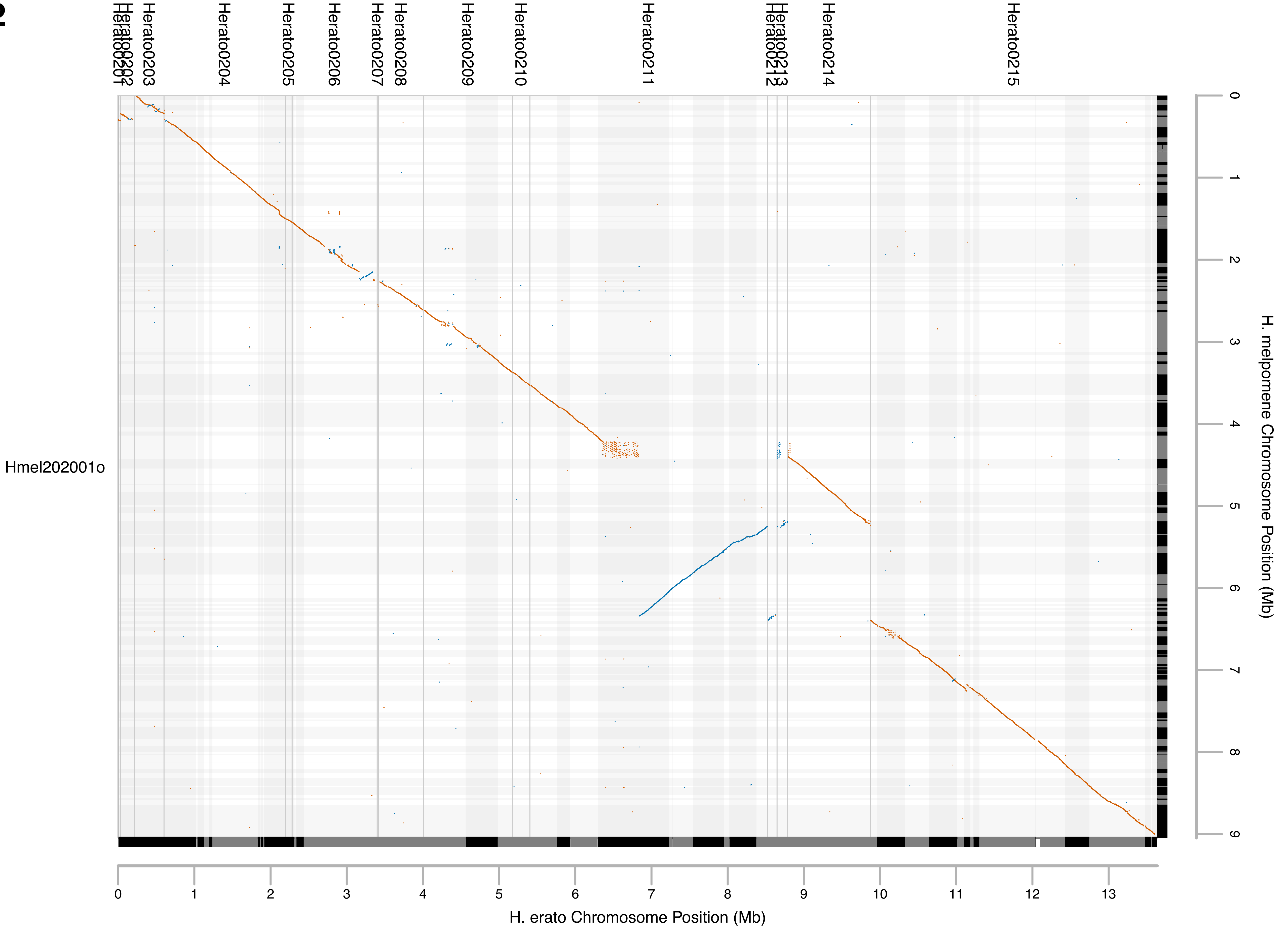
Herato0203
Herato0204
Herato0205
Herato0206
Herato0207
Herato0208
Herato0209
Herato0210
Herato0211
Herato0214
Herato0215
Hmel202001o
H. melpomene Chromosome Position (Mb)
H. erato Chromosome Position (Mb)
0
1
2
3
4
5
6
7
8
9
10
11
12
13

3

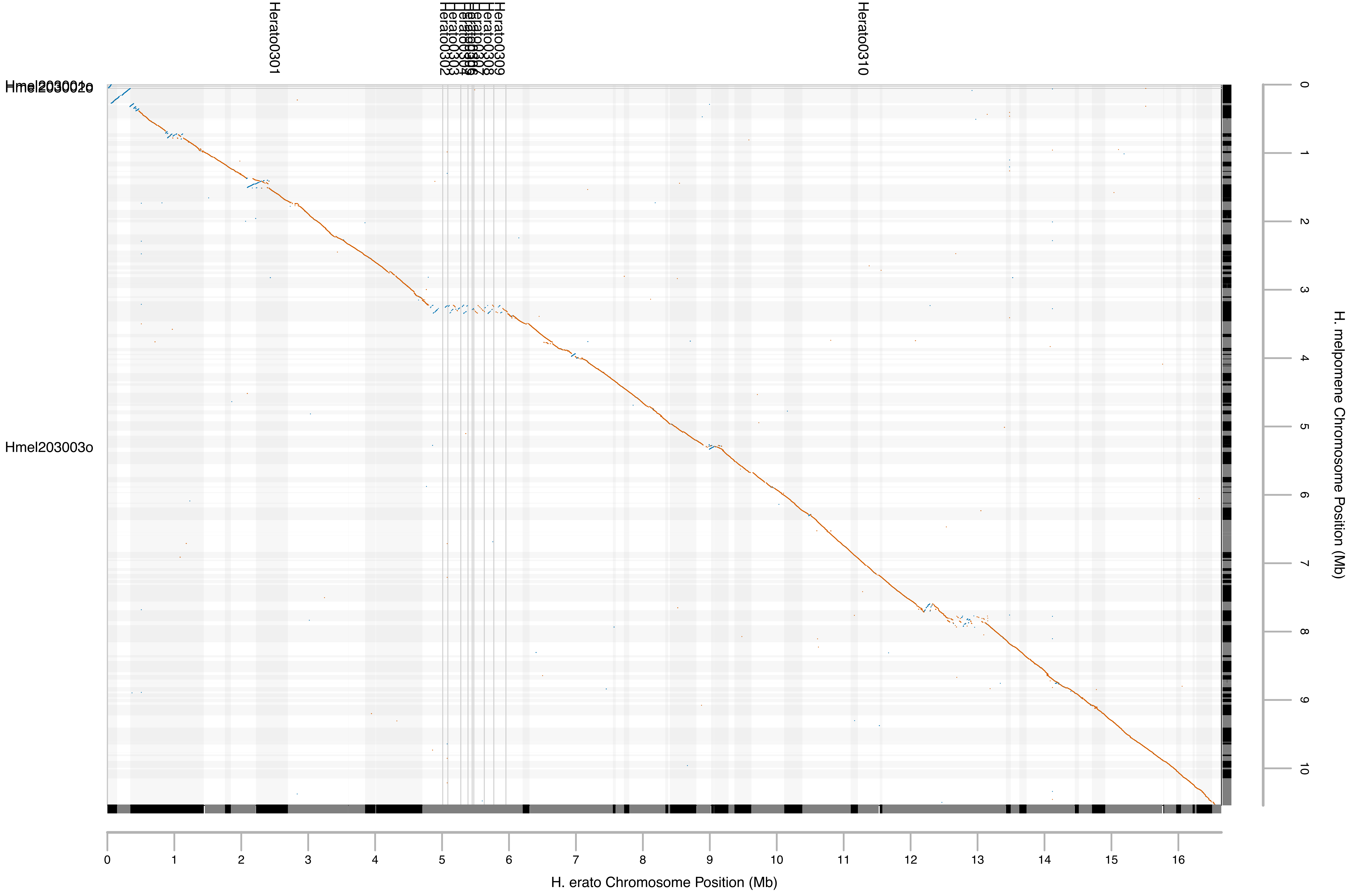
Herato0301
Herato0302
Herato0303
Herato0304
Herato0305
Herato0306
Herato0307
Herato0308
Herato0309
Herato0310
Hmel203001o
Hmel203002o
Hmel203003o
H. erato Chromosome Position (Mb)
H. melpomene Chromosome Position (Mb)
0
1
2
3
4
5
6
7
8
9
10
11
12
13
14
15
16

4

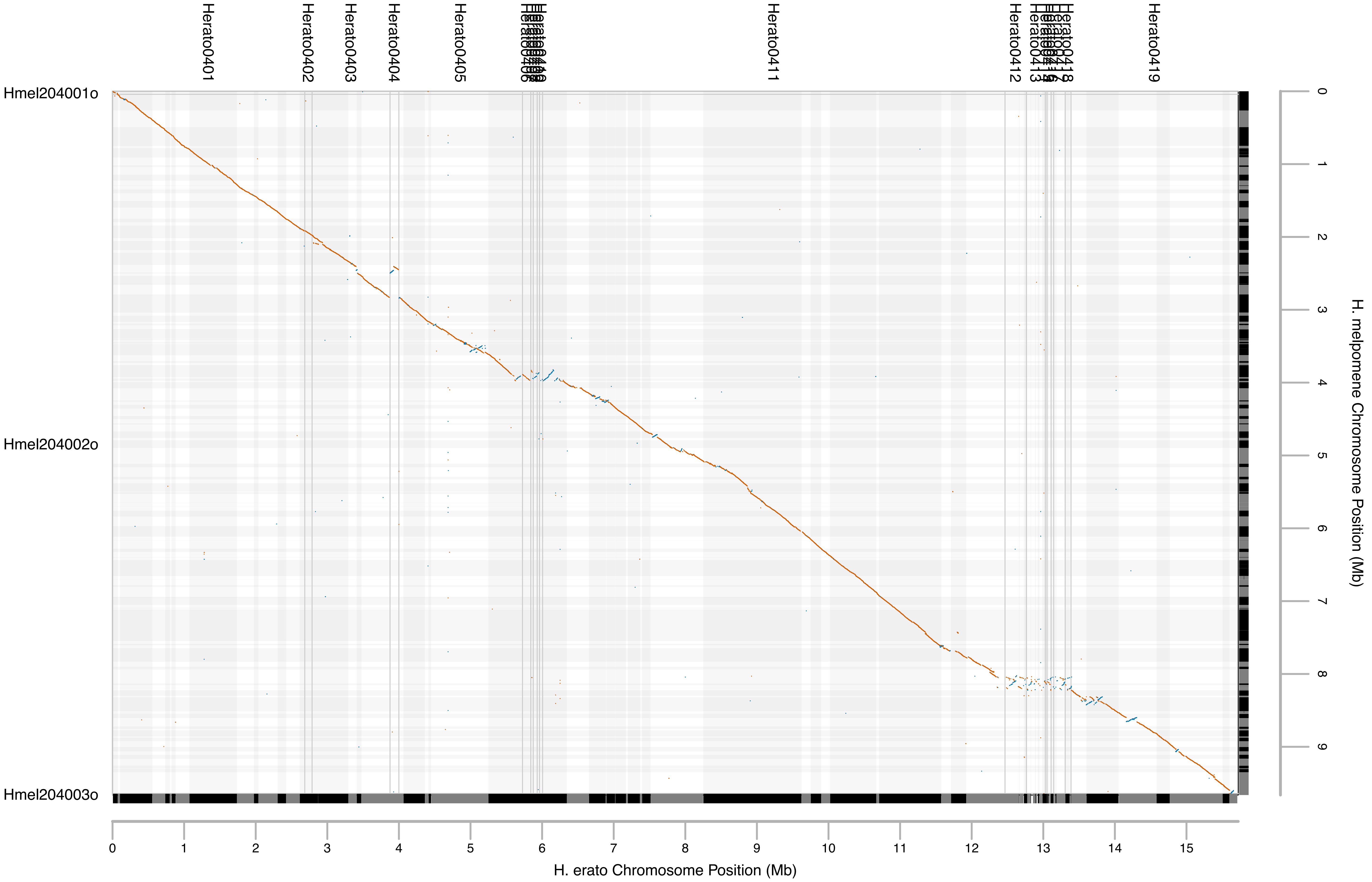
Herato0401
Herato0402
Herato0403
Herato0404
Herato0405
Herato0411
Herato0412
Herato0413
Herato0419
Hmel204001o
Hmel204002o
Hmel204003o
0
1
2
3
4
5
6
7
8
9
10
11
12
13
14
15
H. erato Chromosome Position (Mb)
H. melpomene Chromosome Position (Mb)

5

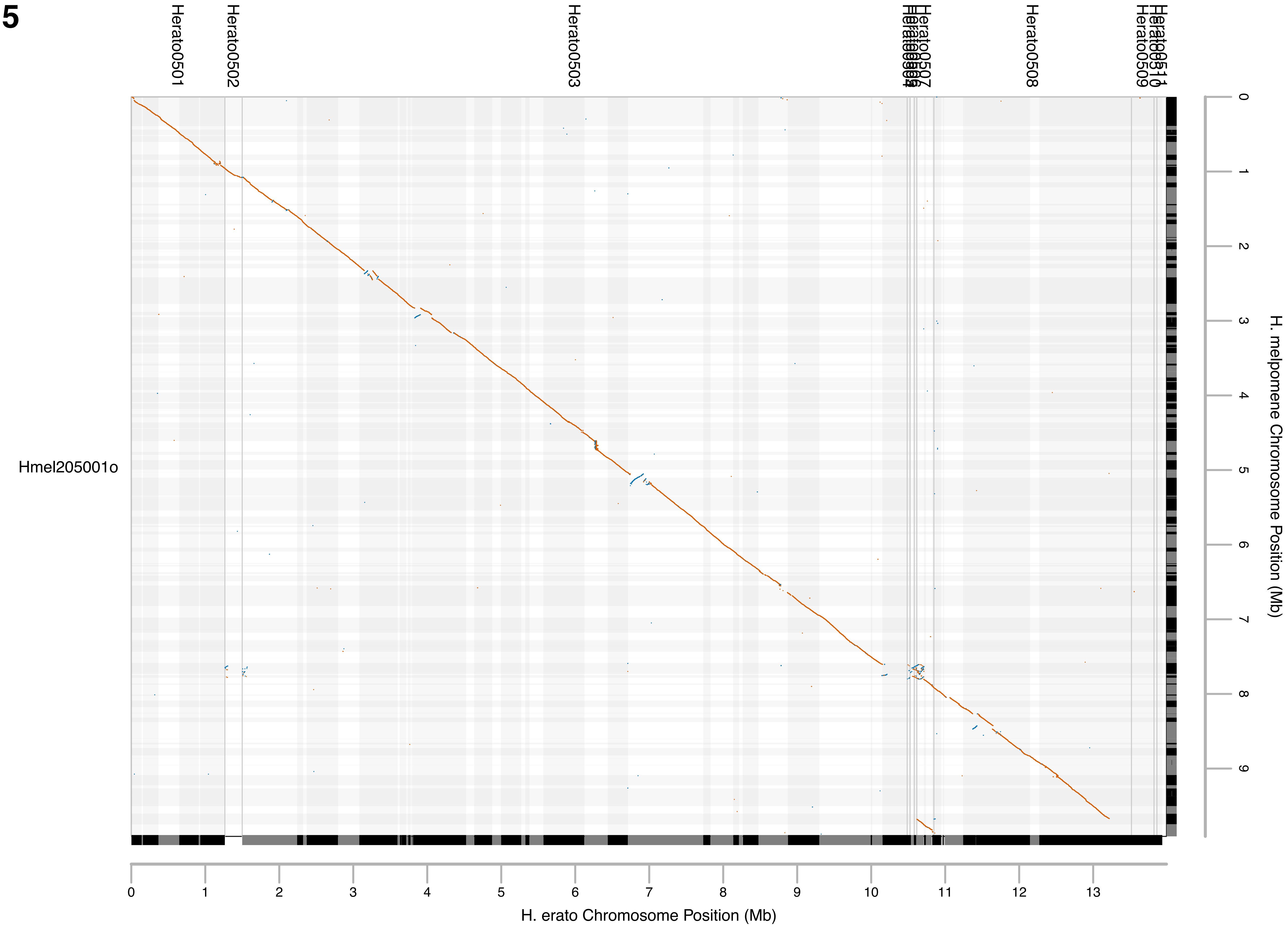
Herato0501
Herato0502
Herato0503
Herato0507
Herato0508
Herato0509
Hmel205001o
H. melpomene Chromosome Position (Mb)
H. erato Chromosome Position (Mb)
0
1
2
3
4
5
6
7
8
9
10
11
12
13

6

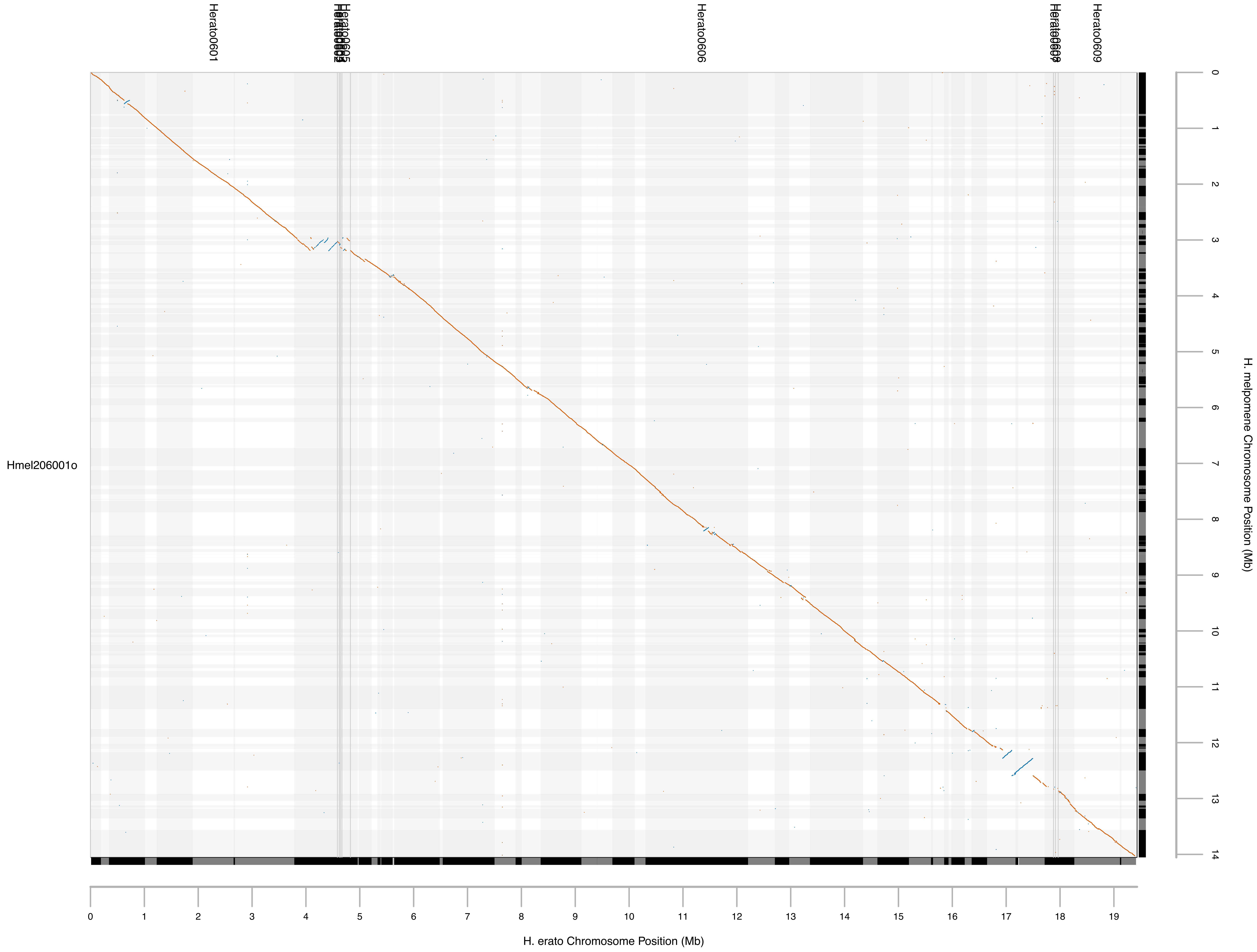
Herato0601
Herato0606
Herato0609
Hmel206001o
H. melpomene Chromosome Position (Mb)
H. erato Chromosome Position (Mb)

7

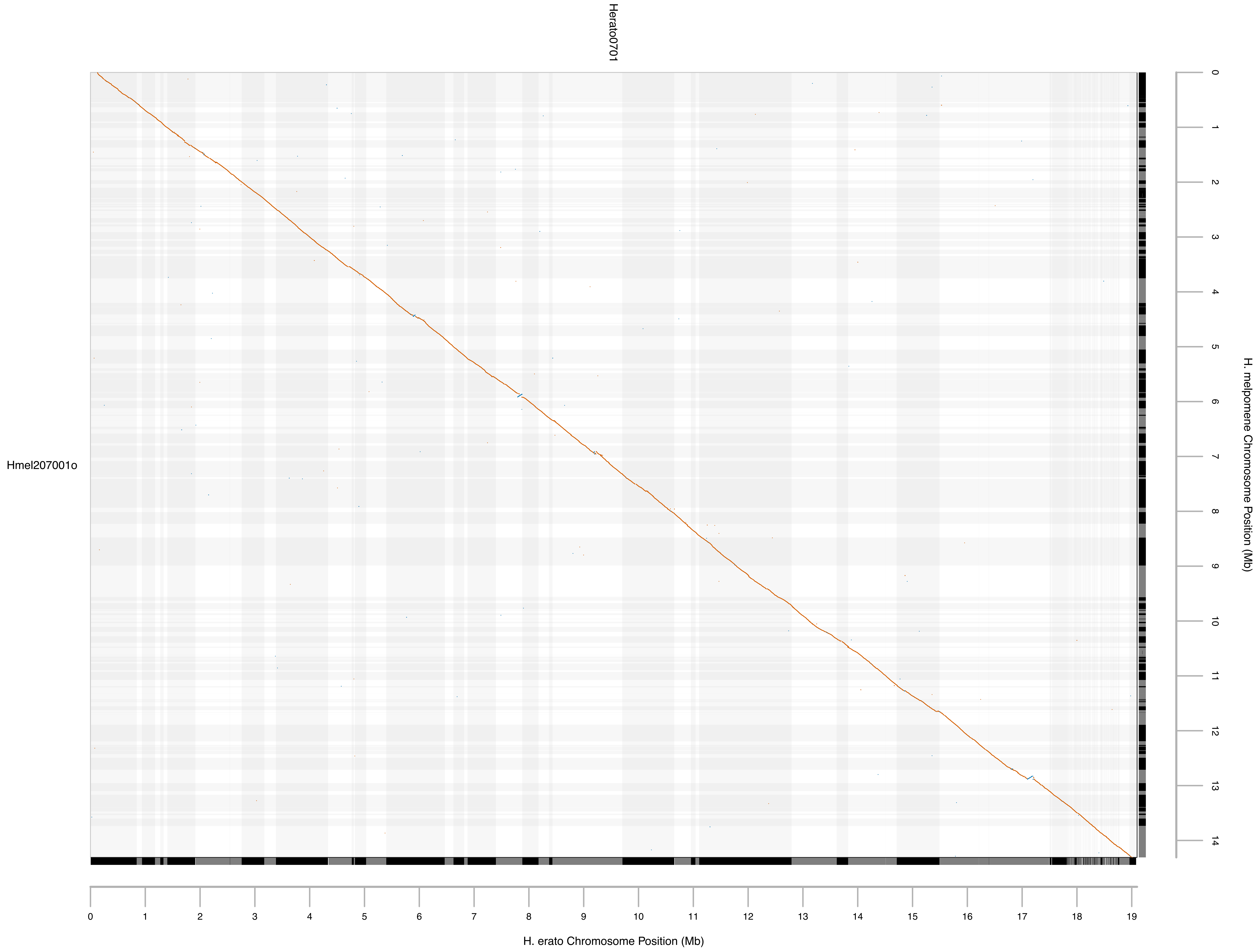
Herato0701
Hmel207001o
H. melpomene Chromosome Position (Mb)
0
1
2
3
4
5
6
7
8
9
10
11
12
13
14
0
1
2
3
4
5
6
7
8
9
10
11
12
13
14
15
16
17
18
19
H. erato Chromosome Position (Mb)

8

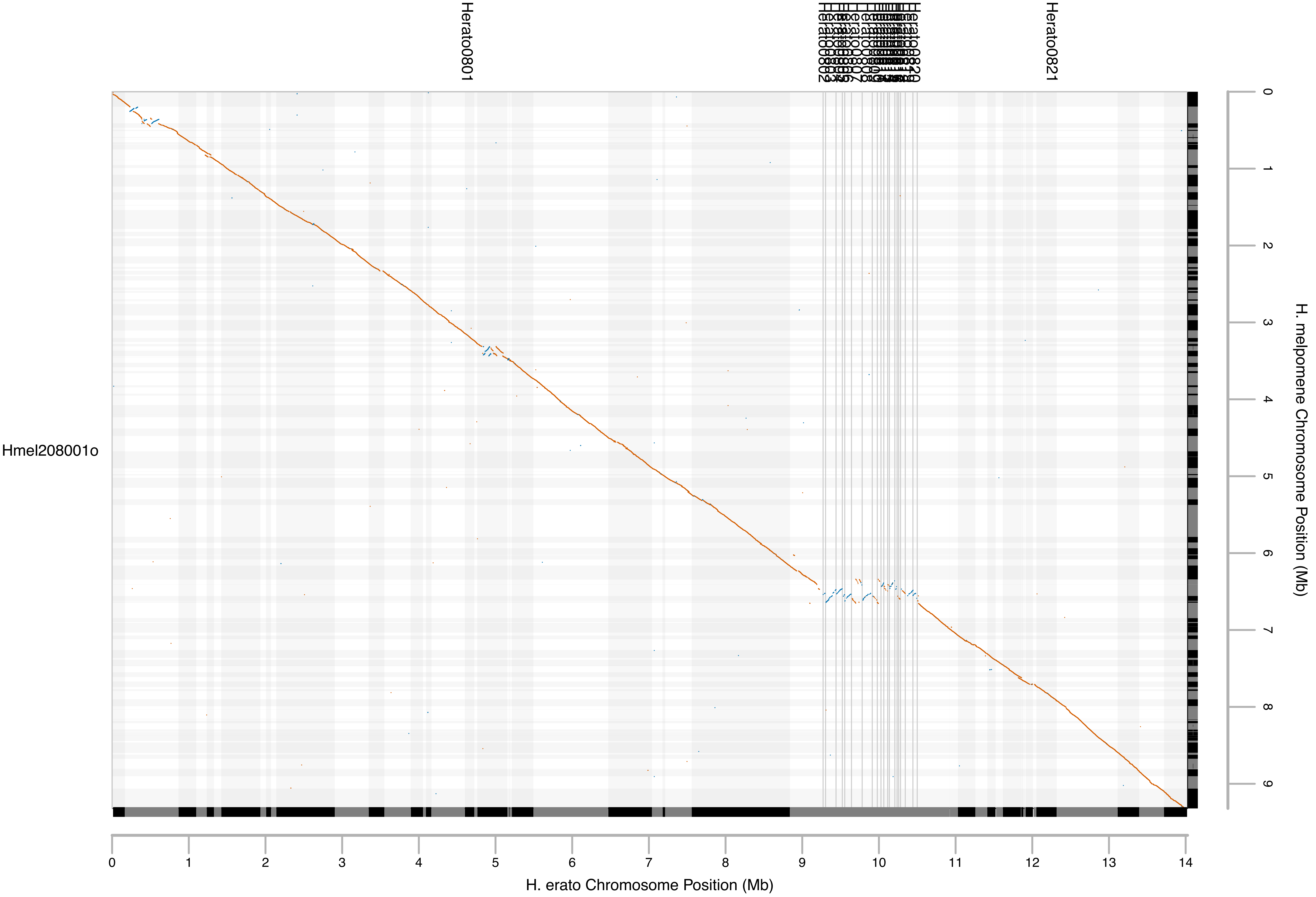
Herato0801
Herato0821
Hmel208001o
H. melpomene Chromosome Position (Mb)
H. erato Chromosome Position (Mb)
0
1
2
3
4
5
6
7
8
9
10
11
12
13
14

9

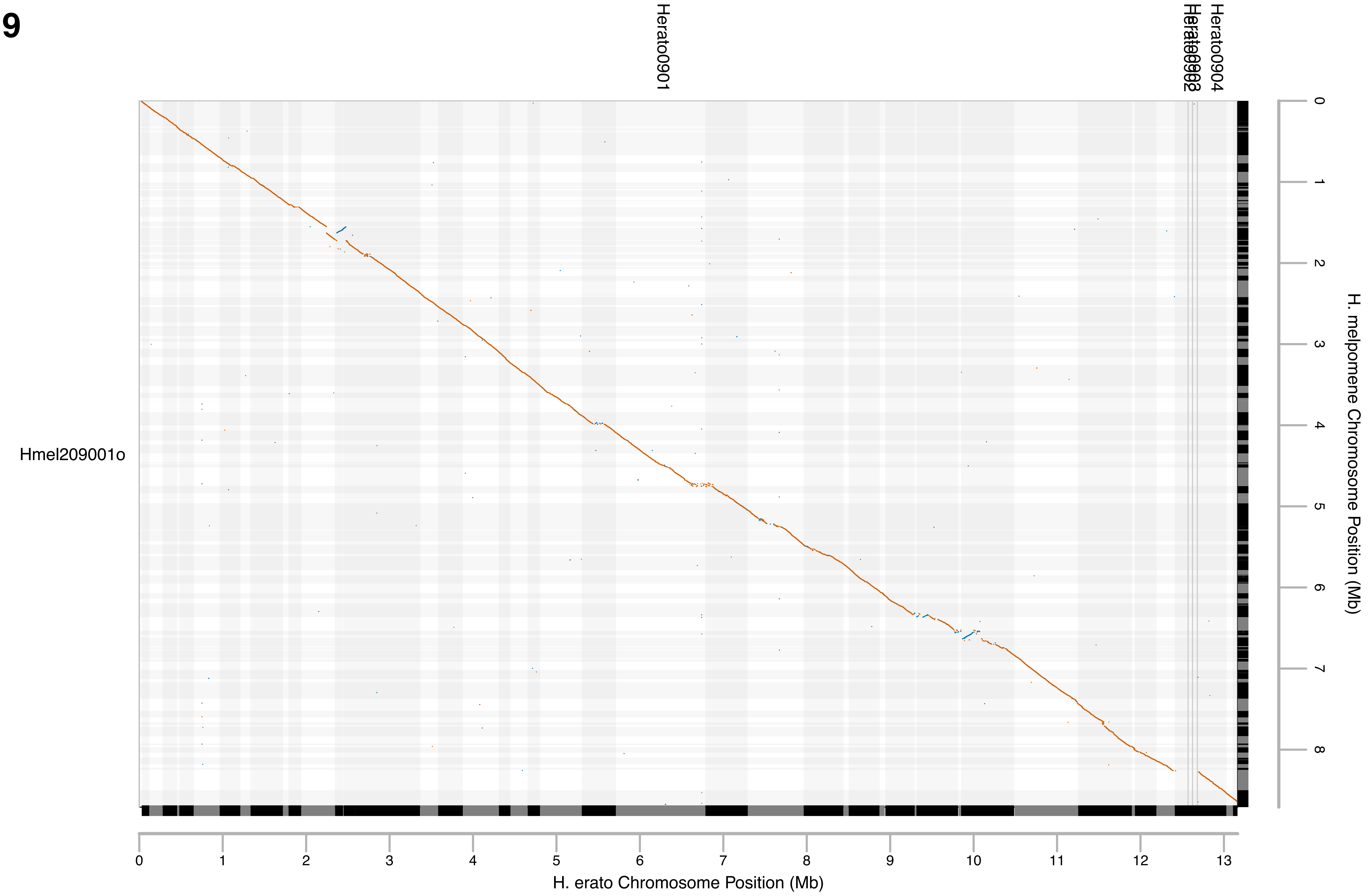
Herato0901
Herato0902
Herato0903
Herato0904
Hmel209001o
H. melpomene Chromosome Position (Mb)
0
1
2
3
4
5
6
7
8
H. erato Chromosome Position (Mb)
0
1
2
3
4
5
6
7
8
9
10
11
12
13

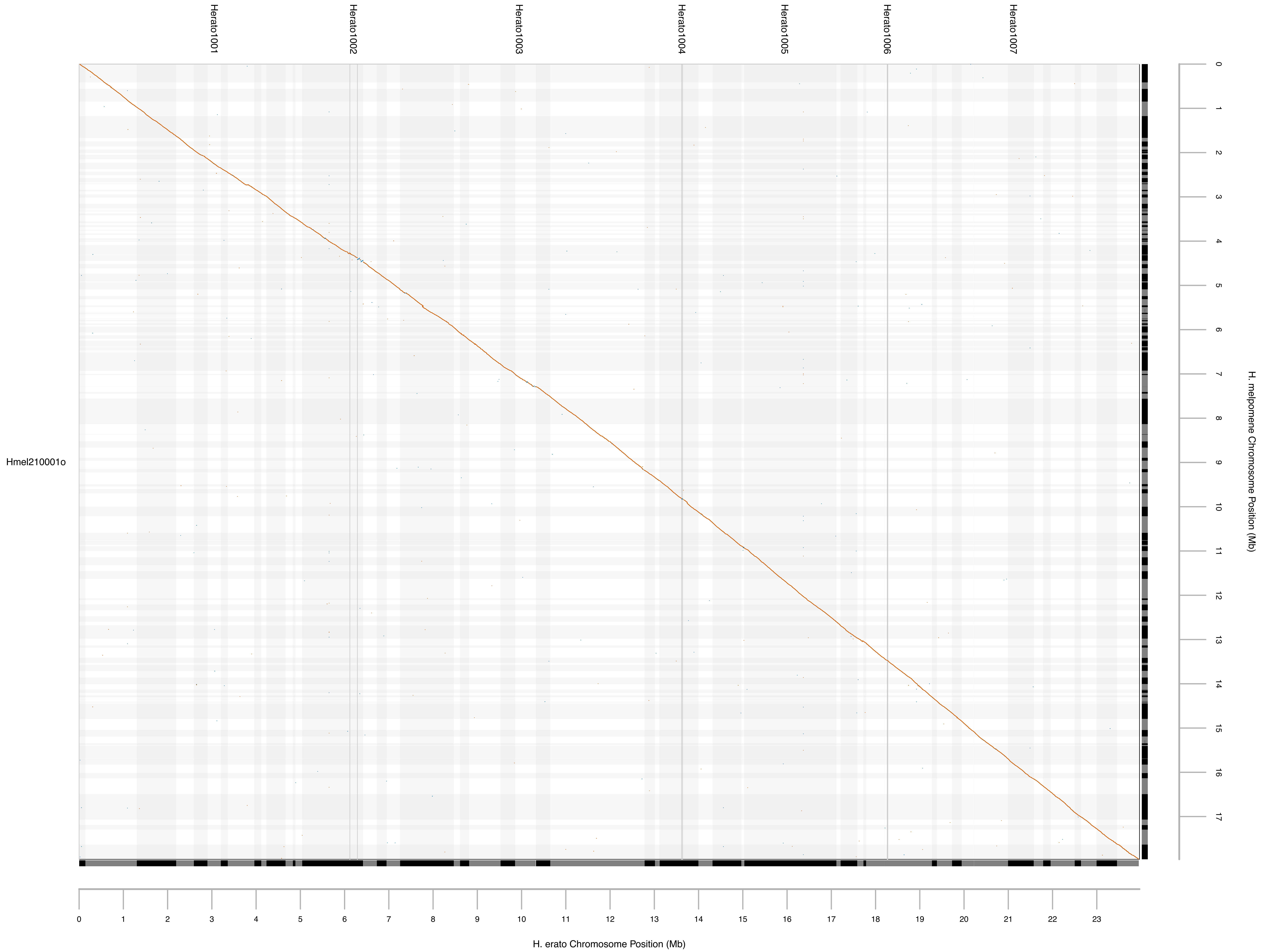

Herato1001
Herato1002
Herato1003
Herato1004
Herato1005
Herato1006
Herato1007
Hmel210001o
H. melpomene Chromosome Position (Mb)
H. erato Chromosome Position (Mb)

11

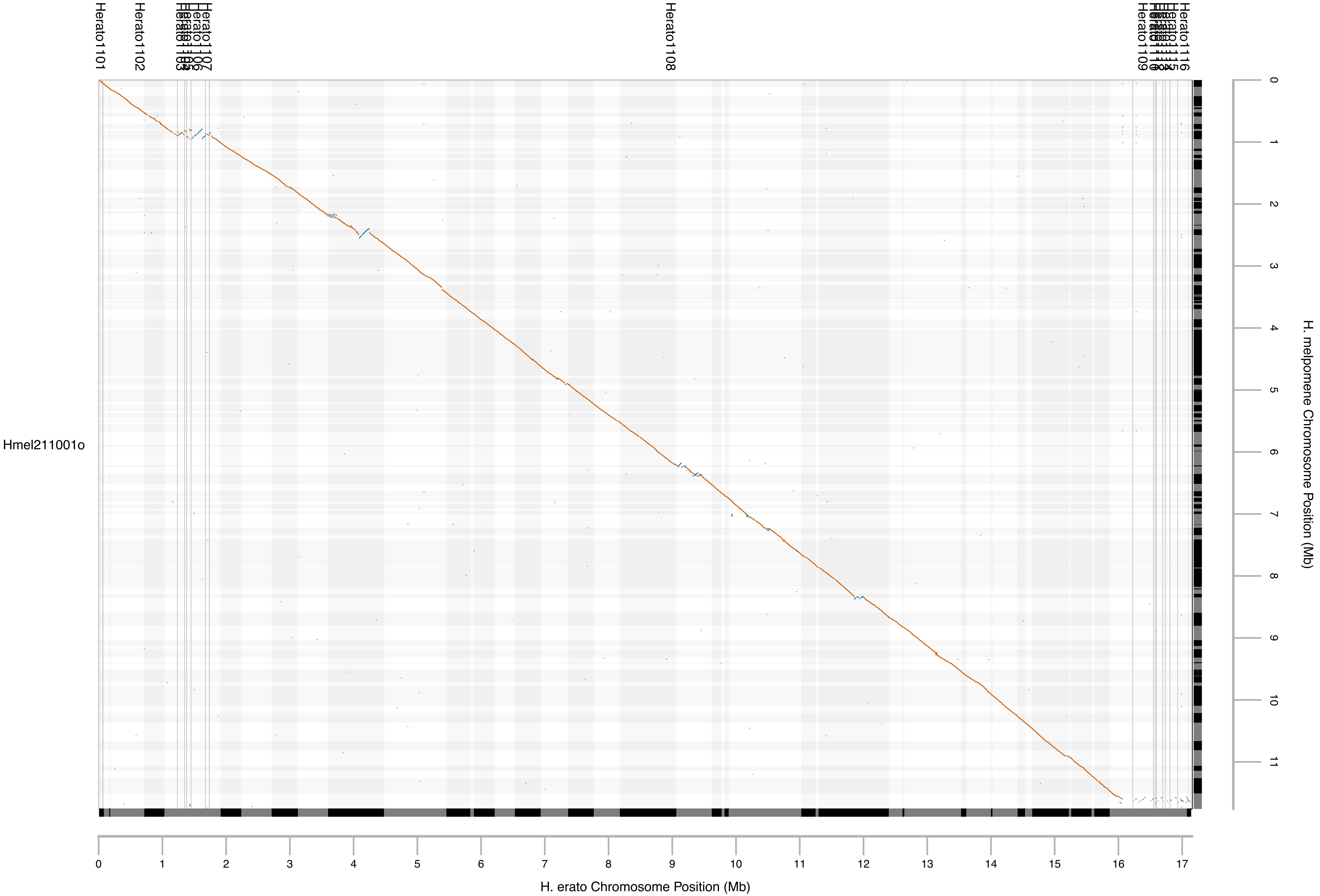
Herato1101
Herato1102
Herato1107
Herato1108
Herato1109
Herato1116
Hmel211001o
H. melpomene Chromosome Position (Mb)
H. erato Chromosome Position (Mb)
0
1
2
3
4
5
6
7
8
9
10
11
12
13
14
15
16
17

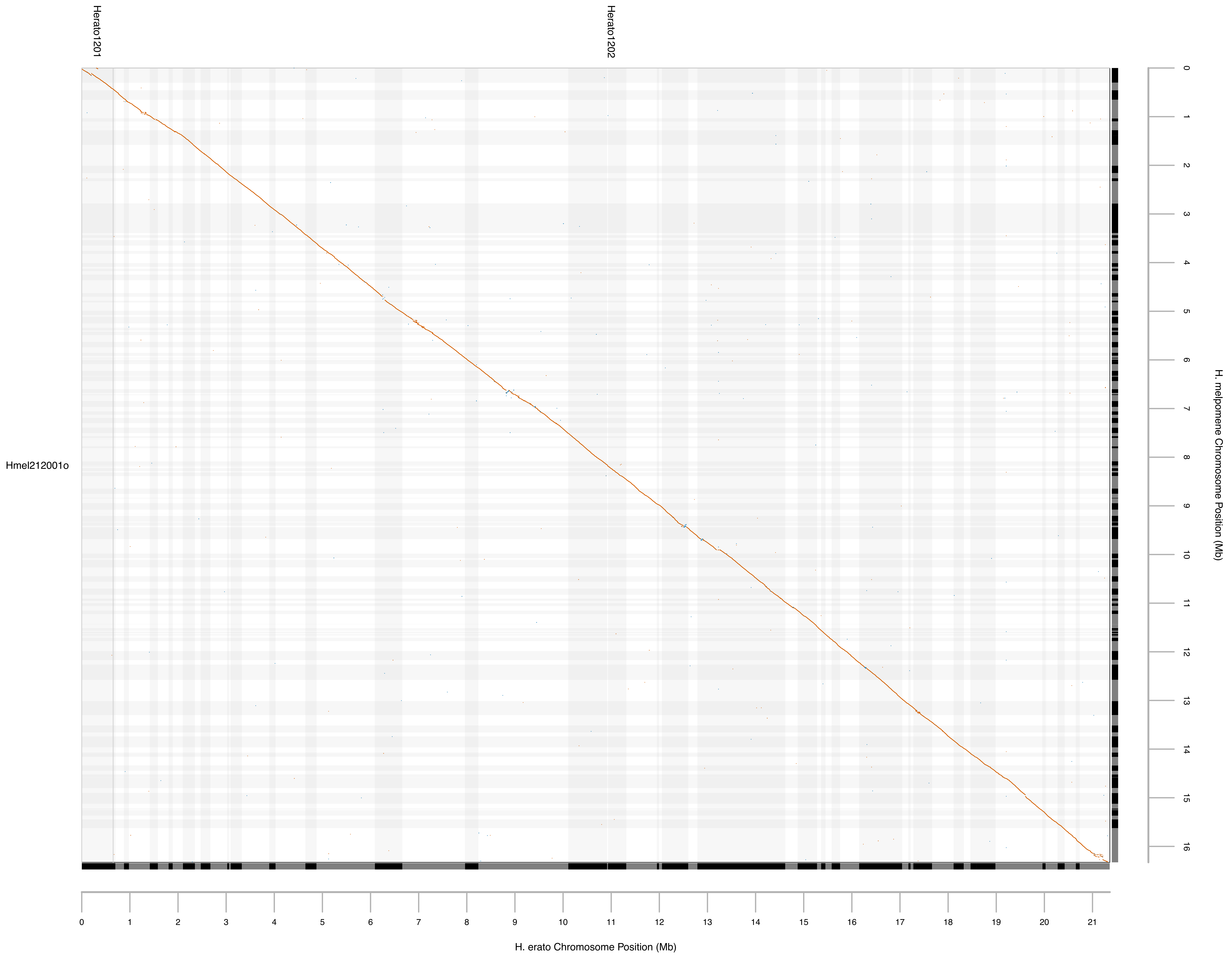
Herato1201
Herato1202
Hmel212001o
H. melpomene Chromosome Position (Mb)
H. erato Chromosome Position (Mb)

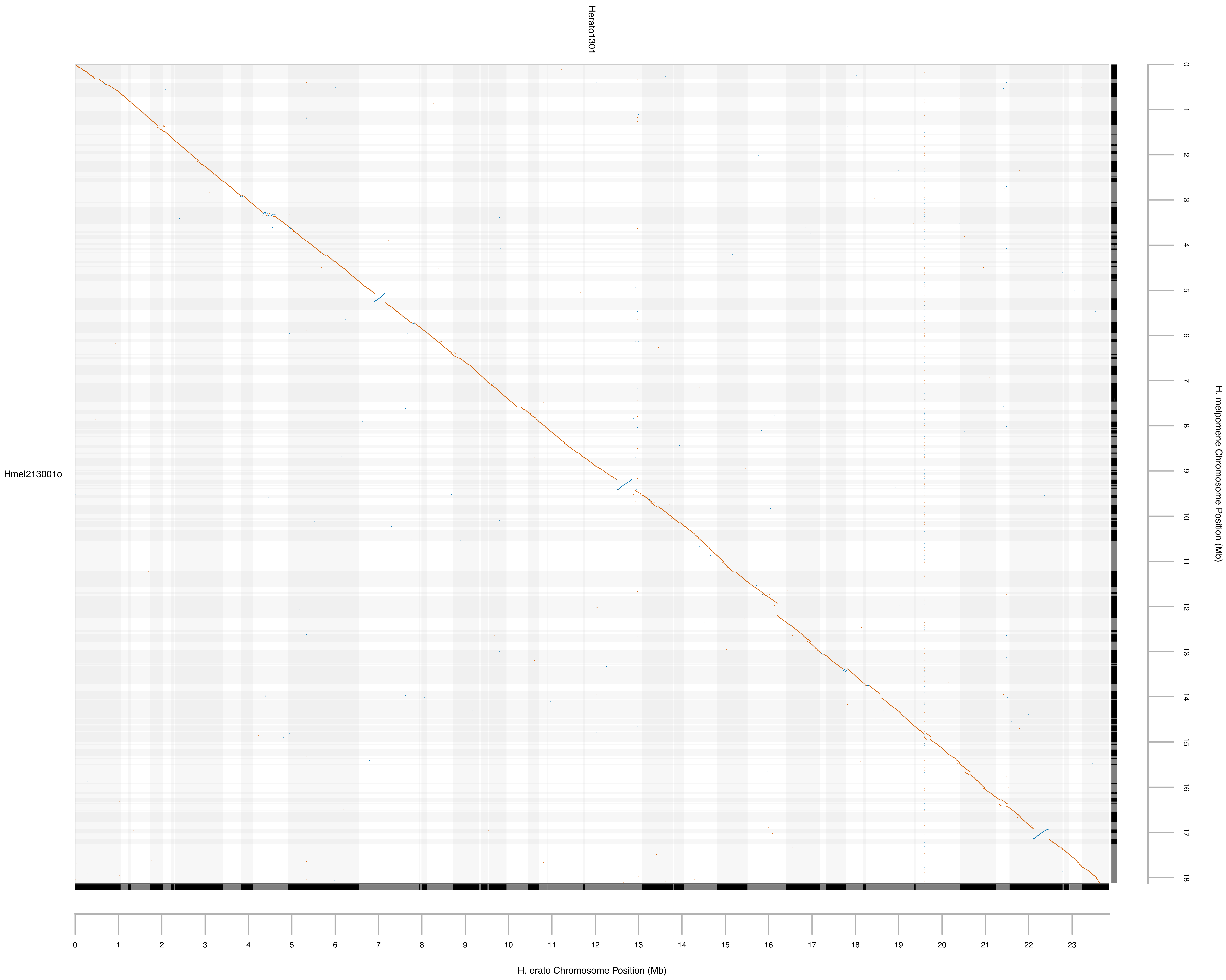

Herato1301
Hmel213001o
H. melpomene Chromosome Position (Mb)
0
1
2
3
4
5
6
7
8
9
10
11
12
13
14
15
16
17
18
0
1
2
3
4
5
6
7
8
9
10
11
12
13
14
15
16
17
18
19
20
21
22
23
H. erato Chromosome Position (Mb)

14

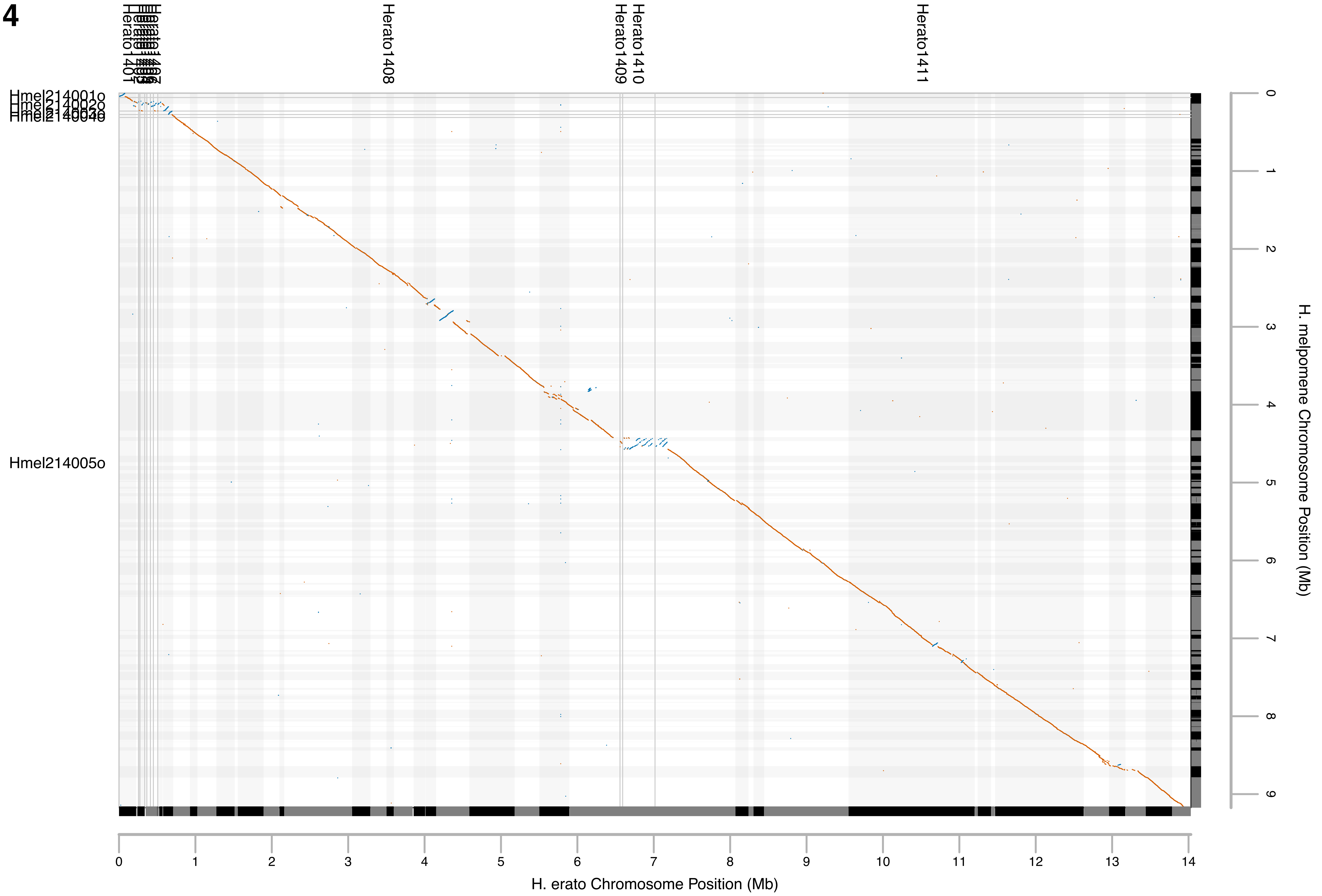

Herato1401
Herato1408
Herato1409
Herato1410
Herato1411
Hmel214001o
Hmel214002o
Hmel214005o
0
1
2
3
4
5
6
7
8
9
10
11
12
13
14
H. erato Chromosome Position (Mb)
H. melpomene Chromosome Position (Mb)

15

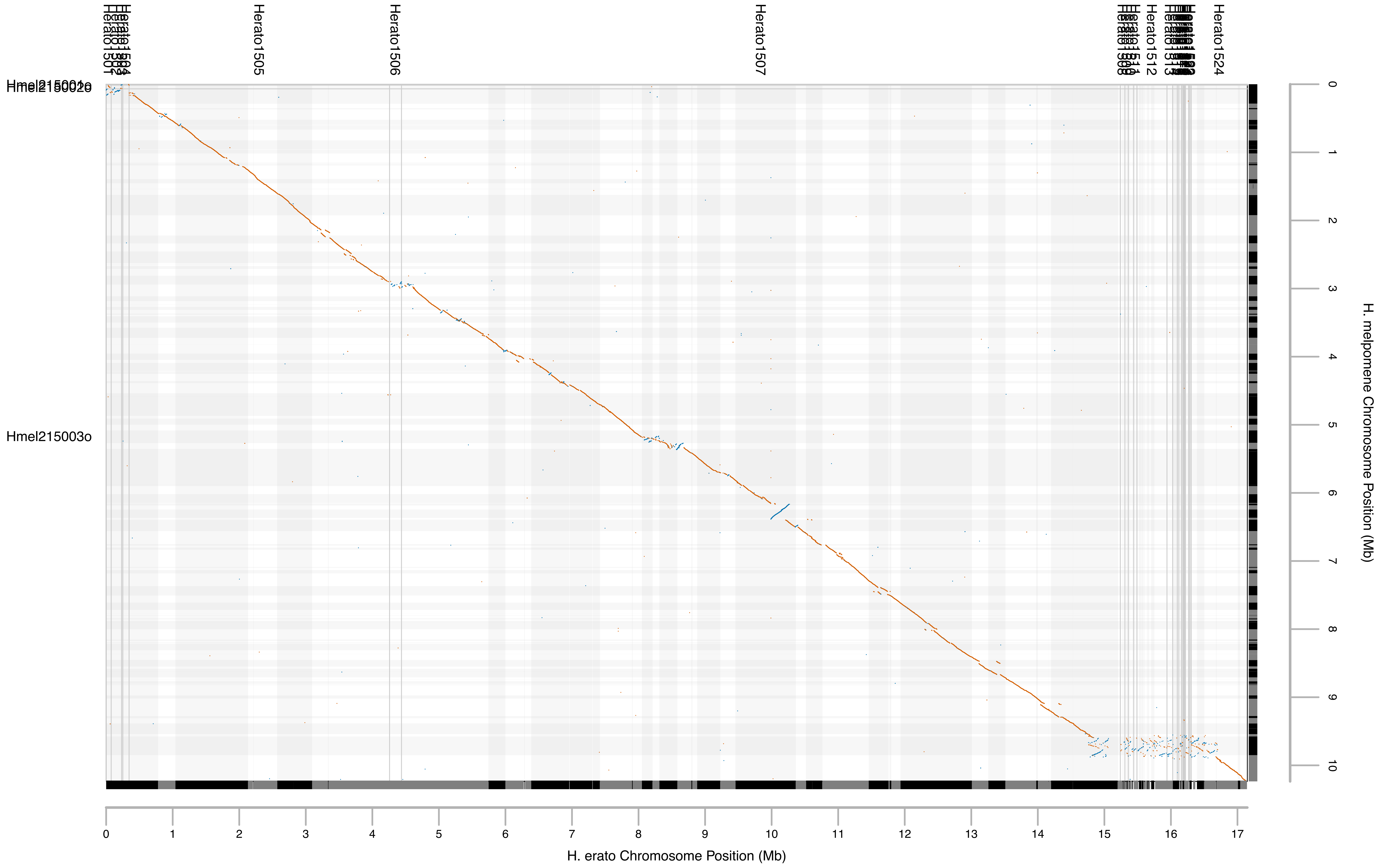
Herato1505
Herato1506
Herato1507
Herato1512
Herato1524
Hmel215003o
H. melpomene Chromosome Position (Mb)
0
1
2
3
4
5
6
7
8
9
10
H. erato Chromosome Position (Mb)
0
1
2
3
4
5
6
7
8
9
10
11
12
13
14
15
16
17

16

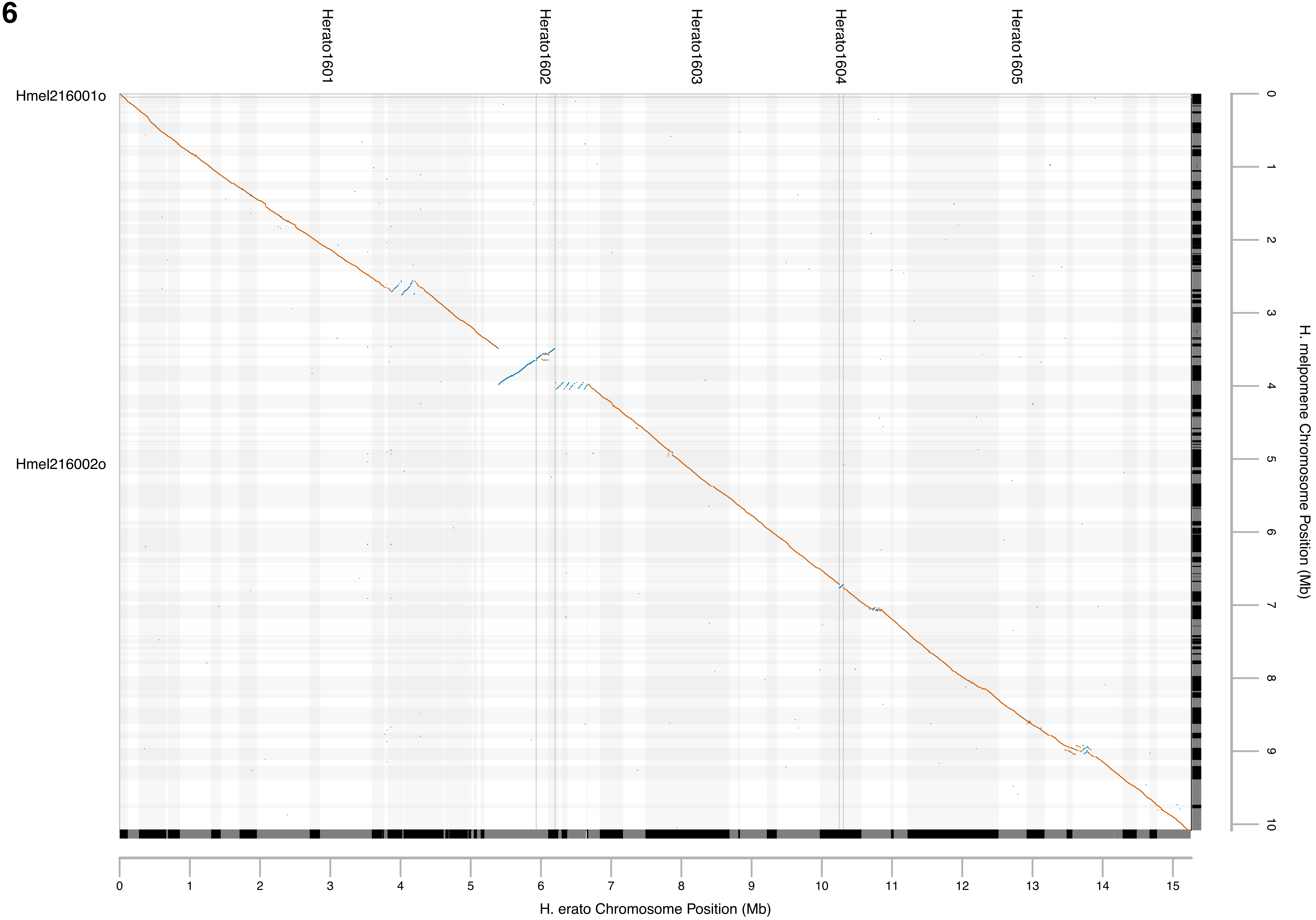

Herato1601
Herato1602
Herato1603
Herato1604
Herato1605
Hmel216001o
Hmel216002o
0
1
2
3
4
5
6
7
8
9
10
11
12
13
14
15
H. erato Chromosome Position (Mb)
H. melpomene Chromosome Position (Mb)

17

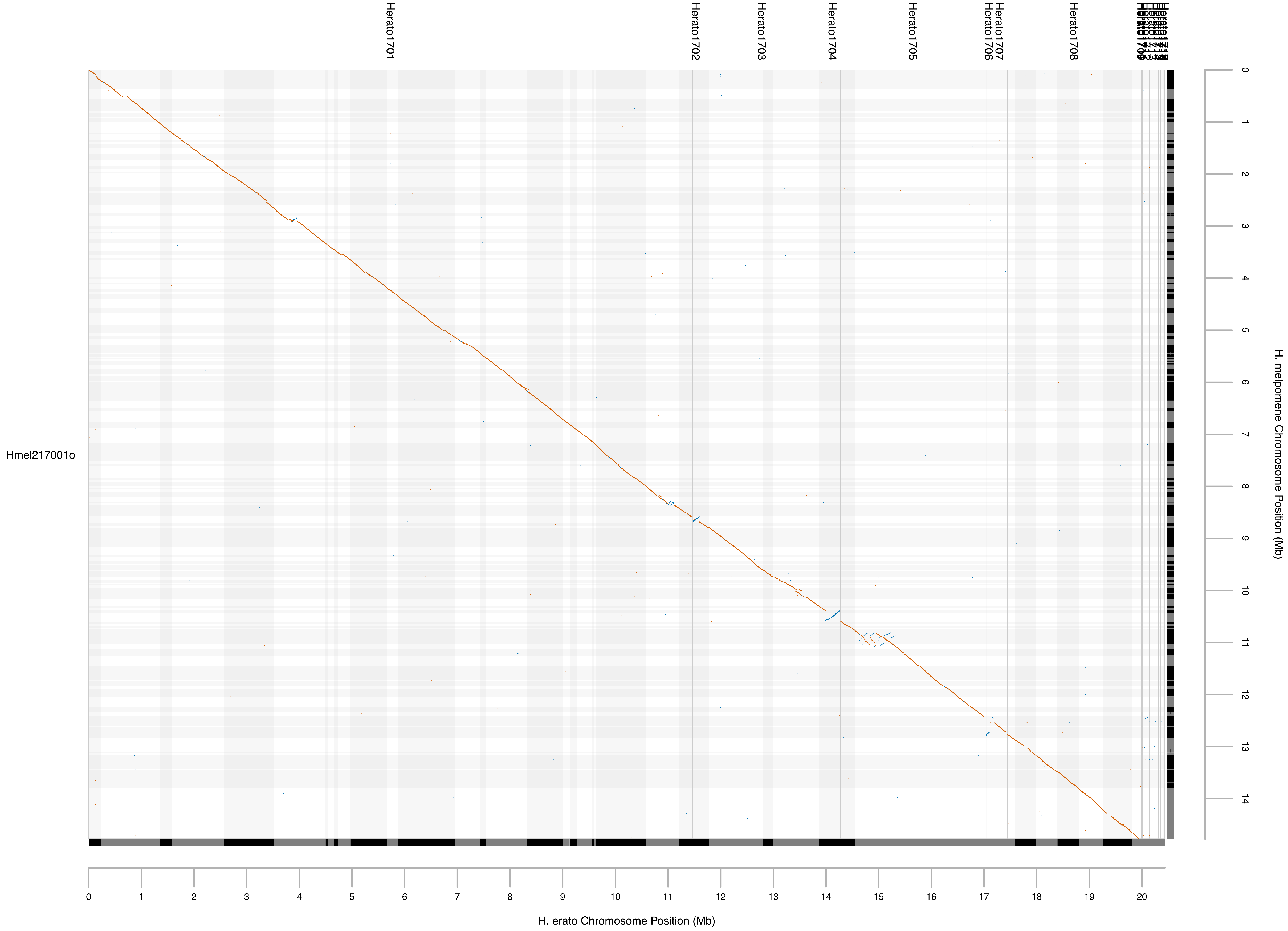
Herato1701
Herato1702
Herato1703
Herato1704
Herato1705
Herato1706
Herato1707
Herato1708
Hmel217001o
H. melpomene Chromosome Position (Mb)
0
1
2
3
4
5
6
7
8
9
10
11
12
13
14
H. erato Chromosome Position (Mb)
0
1
2
3
4
5
6
7
8
9
10
11
12
13
14
15
16
17
18
19
20

18

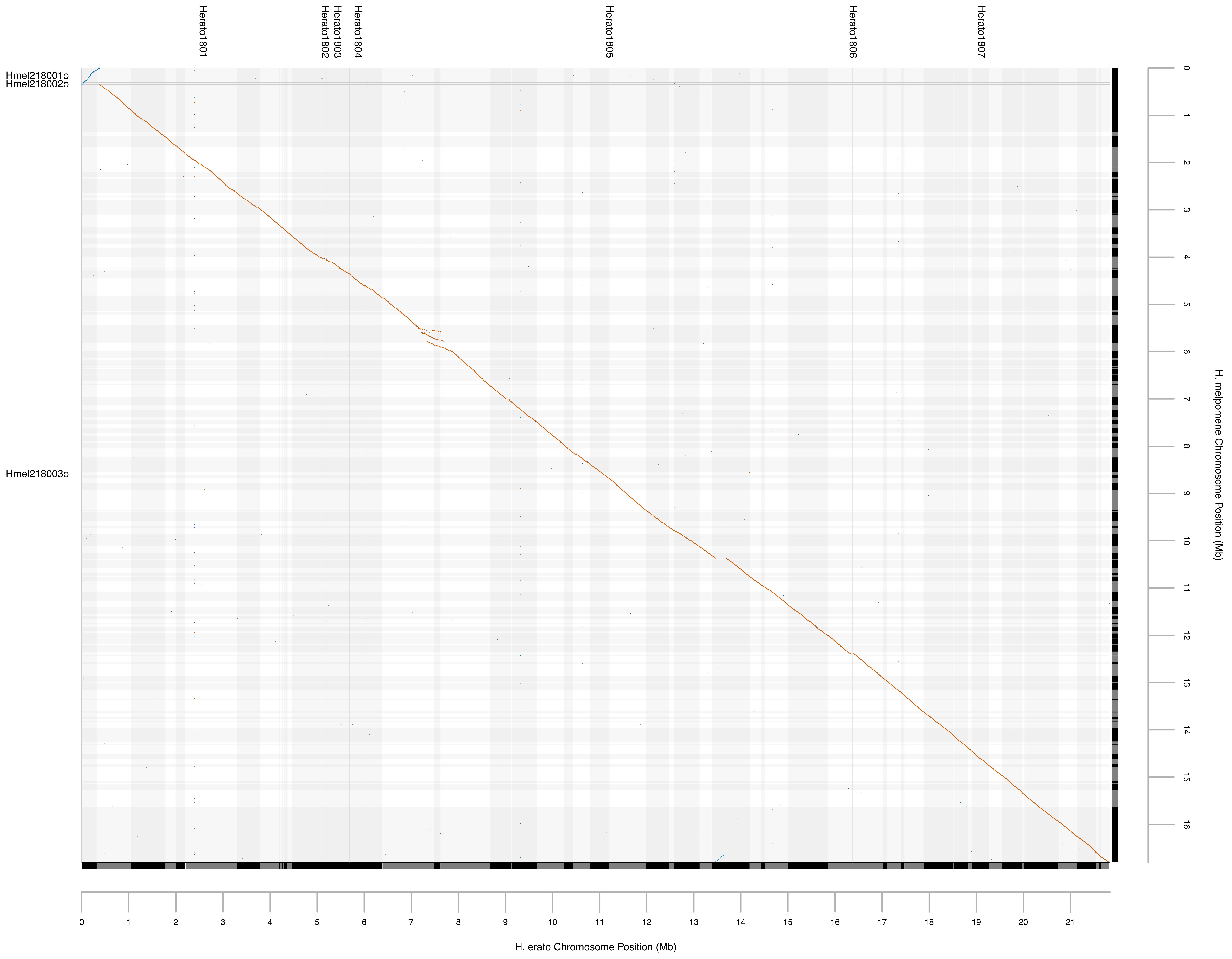

Herato1801
Herato1802
Herato1803
Herato1804
Herato1805
Herato1806
Herato1807
Hmel218001o
Hmel218002o
Hmel218003o
H. melpomene Chromosome Position (Mb)
H. erato Chromosome Position (Mb)

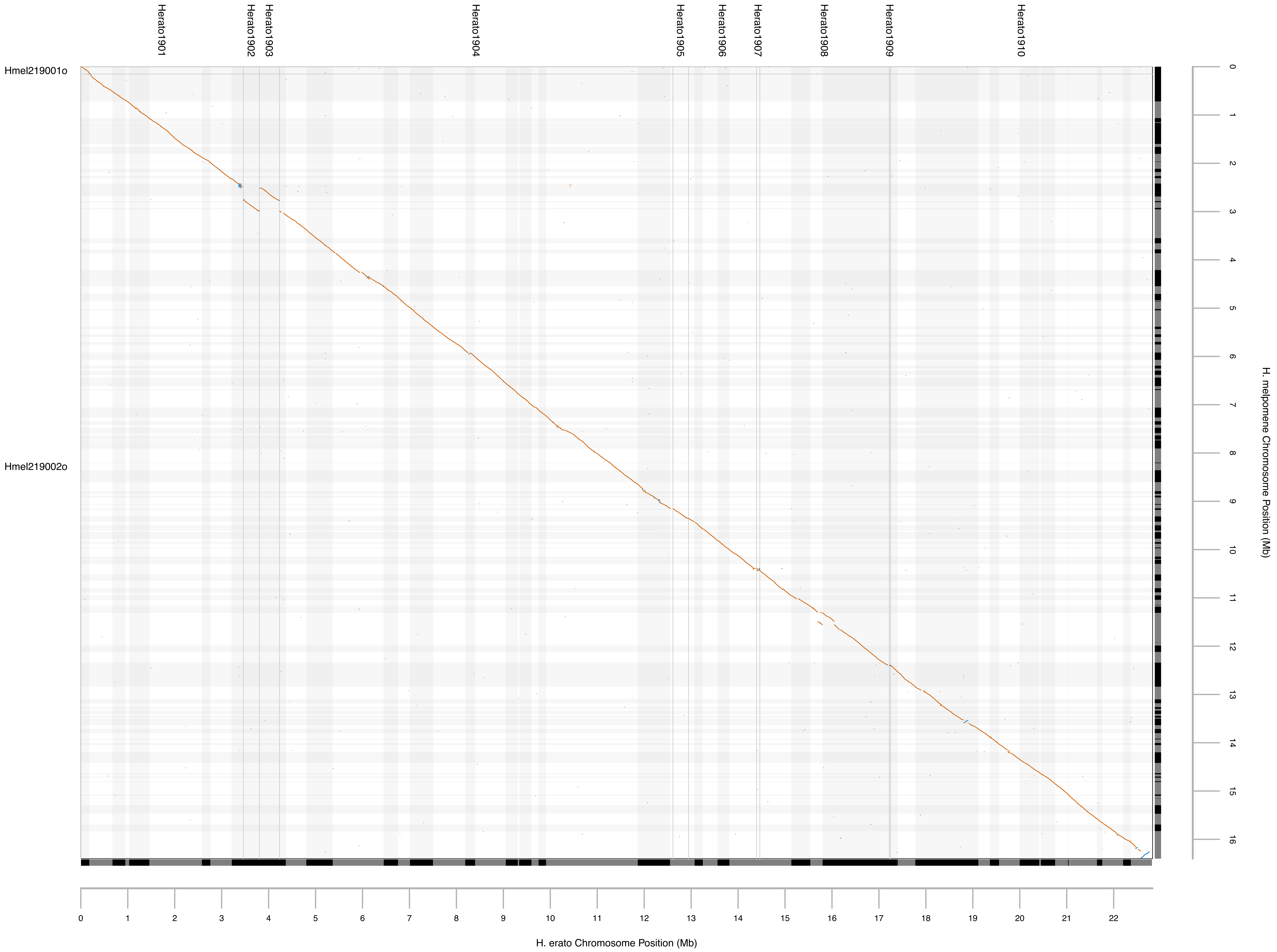
Herato1901
Herato1902
Herato1903
Herato1904
Herato1905
Herato1906
Herato1907
Herato1908
Herato1909
Herato1910
Hmel219001o
Hmel219002o
H. melpomene Chromosome Position (Mb)
H. erato Chromosome Position (Mb)

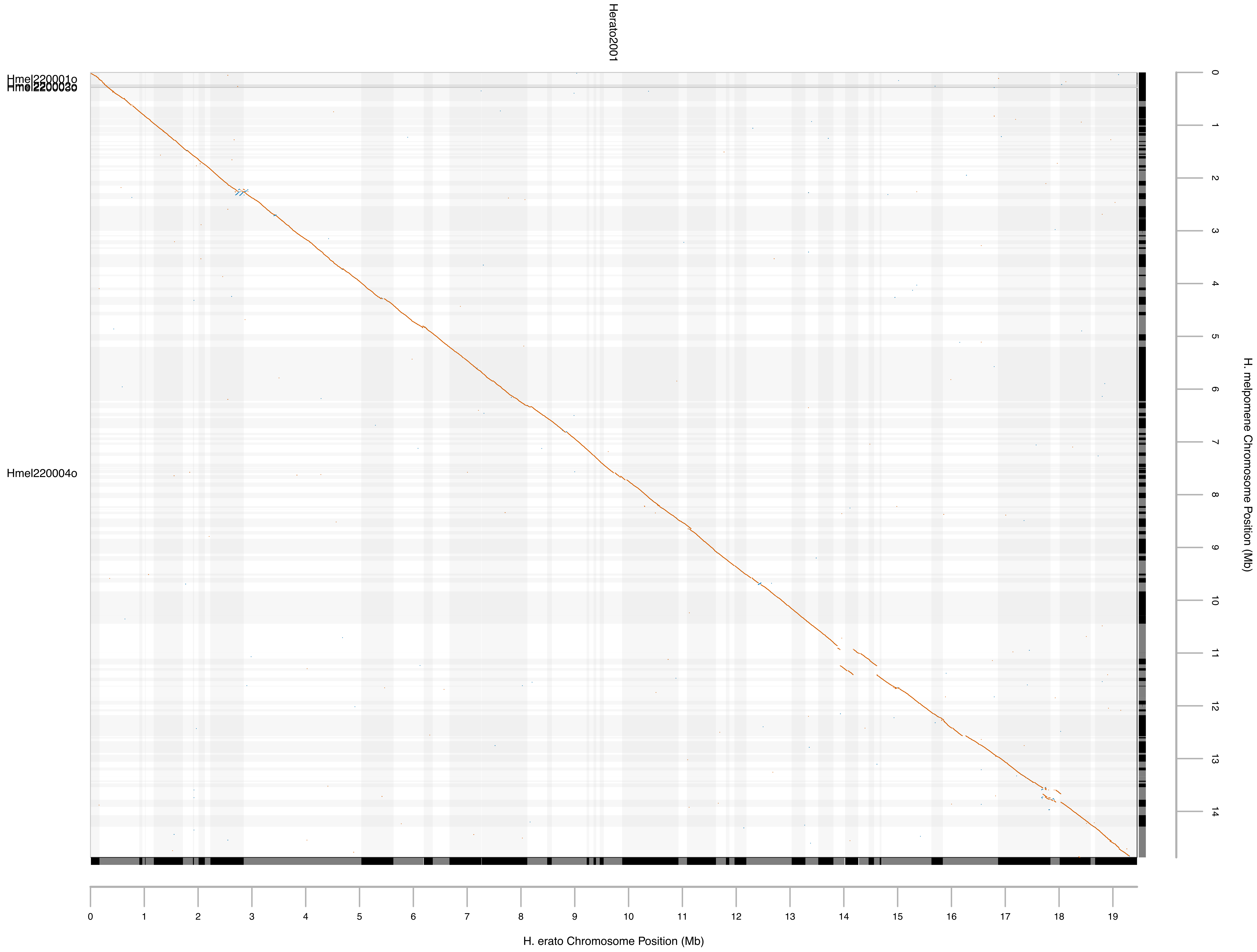
Herato2001
Hmel220001o
Hmel220003o
Hmel220004o
H. melpomene Chromosome Position (Mb)
H. erato Chromosome Position (Mb)

21

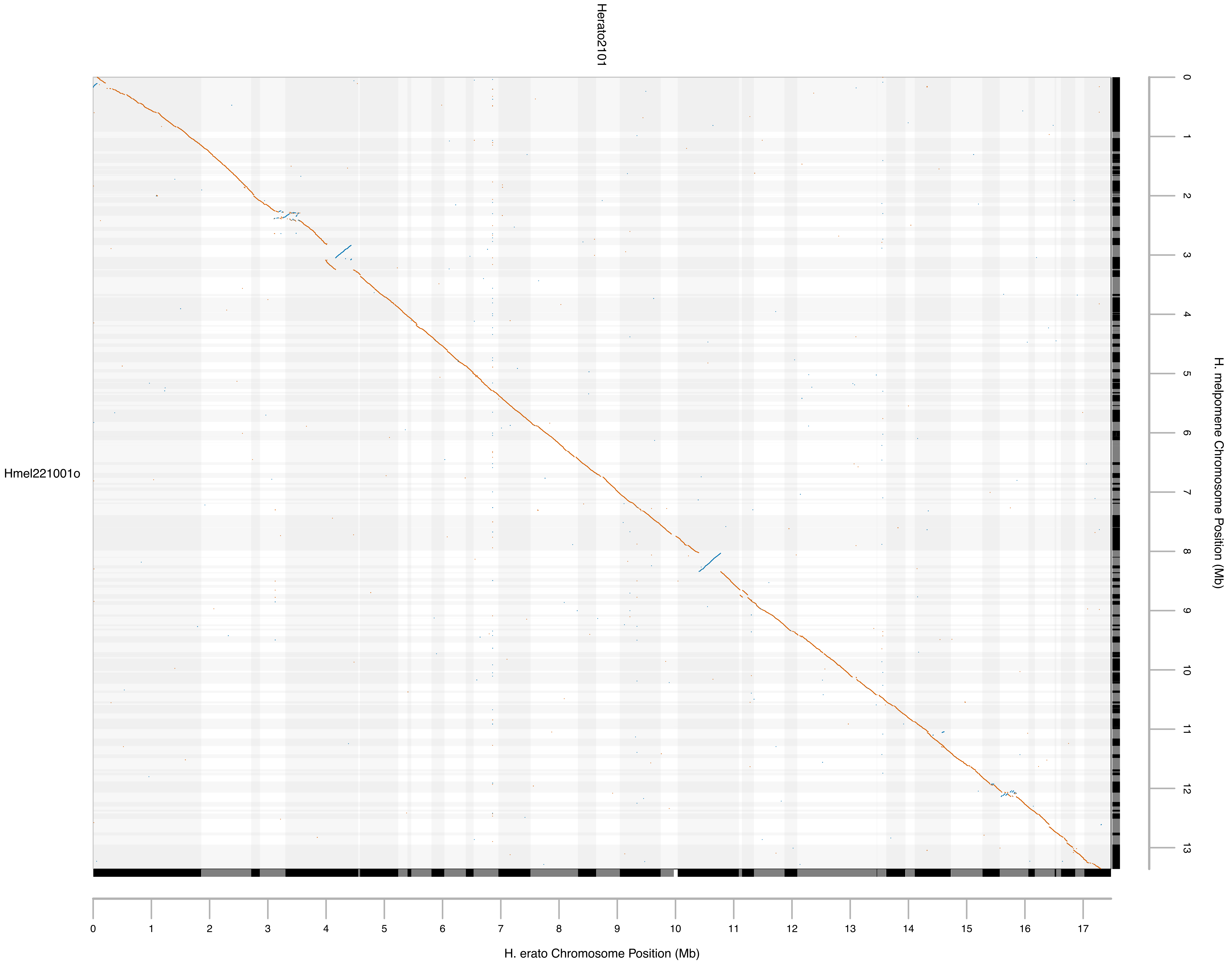

Herato2101
Hmel221001o
H. melpomene Chromosome Position (Mb)
0
1
2
3
4
5
6
7
8
9
10
11
12
13
H. erato Chromosome Position (Mb)
0
1
2
3
4
5
6
7
8
9
10
11
12
13
14
15
16
17
